# The functional genome of CA1 and CA3 neurons under native conditions and in response to ischemia

**DOI:** 10.1186/1471-2164-8-370

**Published:** 2007-10-15

**Authors:** Dieter Newrzella, Payam S Pahlavan, Carola Krüger, Christine Boehm, Oliver Sorgenfrei, Helmut Schröck, Gisela Eisenhardt, Nadine Bischoff, Gerhard Vogt, Oliver Wafzig, Moritz Rossner, Martin H Maurer, Holger Hiemisch, Alfred Bach, Wolfgang Kuschinsky, Armin Schneider

**Affiliations:** 1 Sygnis Bioscience, Im Neuenheimer Feld 515, 69120 Heidelberg, Germany; 2 Department of Physiology, University of Heidelberg, Im Neuenheimer Feld 326, Heidelberg, Germany; 3 MPI for Experimental Medicine, Hermann-Rein-Str. 3, Göttingen, 69120, Germany

## Abstract

**Background:**

The different physiological repertoire of CA3 and CA1 neurons in the hippocampus, as well as their differing behaviour after noxious stimuli are ultimately based upon differences in the expressed genome. We have compared CA3 and CA1 gene expression in the uninjured brain, and after cerebral ischemia using laser microdissection (LMD), RNA amplification, and array hybridization.

**Results:**

Profiling in CA1 vs. CA3 under normoxic conditions detected more than 1000 differentially expressed genes that belong to different, physiologically relevant gene ontology groups in both cell types. The comparison of each region under normoxic and ischemic conditions revealed more than 5000 ischemia-regulated genes for each individual cell type. Surprisingly, there was a high co-regulation in both regions. In the ischemic state, only about 100 genes were found to be differentially expressed in CA3 and CA1. The majority of these genes were also different in the native state. A minority of interesting genes (e.g. inhibinbetaA) displayed divergent expression preference under native and ischemic conditions with partially opposing directions of regulation in both cell types.

**Conclusion:**

The differences found in two morphologically very similar cell types situated next to each other in the CNS are large providing a rational basis for physiological differences. Unexpectedly, the genomic response to ischemia is highly similar in these two neuron types, leading to a substantial attenuation of functional genomic differences in these two cell types. Also, the majority of changes that exist in the ischemic state are not generated de novo by the ischemic stimulus, but are preexistant from the genomic repertoire in the native situation. This unexpected influence of a strong noxious stimulus on cell-specific gene expression differences can be explained by the activation of a cell-type independent conserved gene-expression program. Our data generate both novel insights into the relation of the quiescent and stimulus-induced transcriptome in different cells, and provide a large dataset to the research community, both for mapping purposes, as well as for physiological and pathophysiological research.

## Background

The brain harbours a large variety of neuron types and sub-types, besides a large number of glial cells. These neurons can differ strongly in their physiological tasks and capacities, and can be very specialized. In addition, different neuron types also display a quite specialized response to pathophysiological influences. This is exemplified in neurodegenerative disorders, where often mutations in broadly expressed genes only cause pathology in one type of neurons (e.g. SOD1 mutations only affect motoneurons in ALS, Parkin mutations only affect dopaminergic neurons of the substantia nigra in PD). Ultimately, the cause of both differing physiology, as well as different vulnerability to pathophysiologic stimuli must lie in the expressed genome in different cell type.

There are a number of approaches aimed to establish a molecular brain atlas. This is mostly done by high-throughput in-situ hybridizations, and automated image acquisition systems (e.g. [1-4]). Alternatively or complementarily, systematic generation of mice expressing marker proteins under control of large genomic elements may be used [5]. However, these strategies approach the problem on a gene-by-gene basis, and will not allow to explore functional genomic differences between two cell types on a statistical level.

At present, differences in global gene expression between different neuron types in the quiescent brain are not well studied. Moreover, there are hardly any systematic data available that examine how common stimuli change the functional genome in different neuron types. The main reasons for this have been technical difficulties. However, with the advent of laser microdissection, RNA amplification, and high-density DNA-array systems such approaches are now feasible [6,7].

Two very interesting neuron types for an exemplary study of these questions are situated in the hippocampal subfields CA (cornu ammonis)3 and CA1. First, the hippocampus is an intensively studied part of the brain, and a lot of data on the physiological behaviour of these cell types is available. Second, these cells are homogenously placed in cell bands that are easily identifiable, and there is a relatively low degree of contamination by other cells (interneurons, glial cells). Third, these cells display a strong difference in vulnerability to different disease stimuli, the most prominent being cerebral ischemia [8-11].

We have therefore performed a genome-wide comparison of CA3 and CA1 gene expression in the uninjured brain, and after cerebral ischemia using laser microdissection (LMD), RNA amplification, and array hybridization.

## Results

### Experimental setup

**Figure 1:**
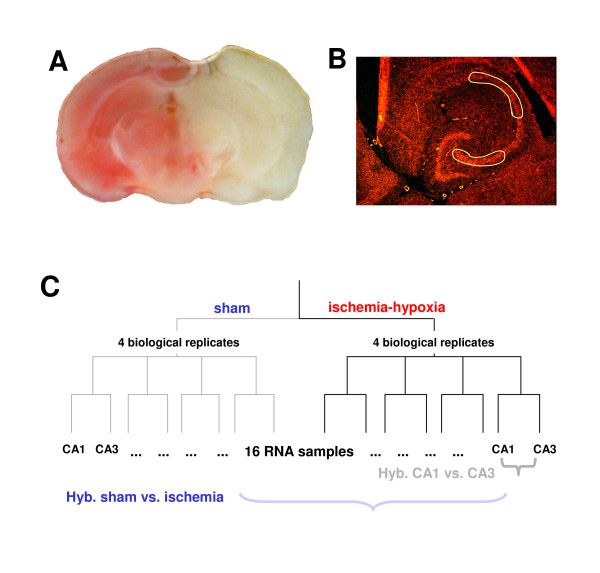
**Experimental model and strategy**. Male C57/bl6 mice were subjected to a combined ischemia/hypoxia model which consistently yields full hemispheric infarcts. A, TTC-stained coronal section 24 h after induction of ischemia/hypoxia demonstrate a full hemispheric infarct which fully covers the hippocampal region. B, The cutting outlines used for Laser microdissection from coronal cryosections are demonstrated on a section stained with an antibody against NeuN and a secondary Cy3-coupled antibody. Actual Laser microdissections in the experiments were performed on Thionin-stained sections. C, Scheme showing the strategy used for detection of differentially regulated genes from amplified RNA. Samples from CA3 and CA1 regions were hybridized on two-color oligonucleotide arrays (Agilent). Direct competitive hybridizations were performed for all combinations: CA3 sham vs, CA1 sham, CA3 ischemia vs CA1 ischemia, CA3 ischemia vs CA3 sham, and CA1 ischemia vs CA1 sham. All experiments were also dye-swapped, and means of the two corresponding values used for further analyses. Arrays were statistically analyzed using linear modelling (limma, R).

We used competitive hybridizations on Agilent G4121 DNA arrays displaying app. 21318 gene products as 60 mer-oligonucleotides. The ischemic model we used was a modified hypoxia/ischemia model as introduced by Vannucci and colleagues [12,13] (Fig. 1A). We dissected CA3 and CA1 hippocampal subfields by laser microdissection from Thionin-stained cryosections (exemplary outline shown on a NeuN stained section in Fig. 1B), and amplified the RNA. There was no difference in size distribution of amplified RNA from different animals, different subfields, or the experimental group (sham vs. ischemia) (see Additional file 1). Gene regulation in CA1 vs. CA3 regions from uninjured and ischemic animals (n = 4 each) was determined in a total of 32 combinatorial hybridizations, including dye-swap controls (Fig. 1C). Microarray datasets generated in this study have been submitted in MIAME-compliant format to the EBI database [14].

### Comparison of differential gene expression in the normoxic state

**Figure 2:**
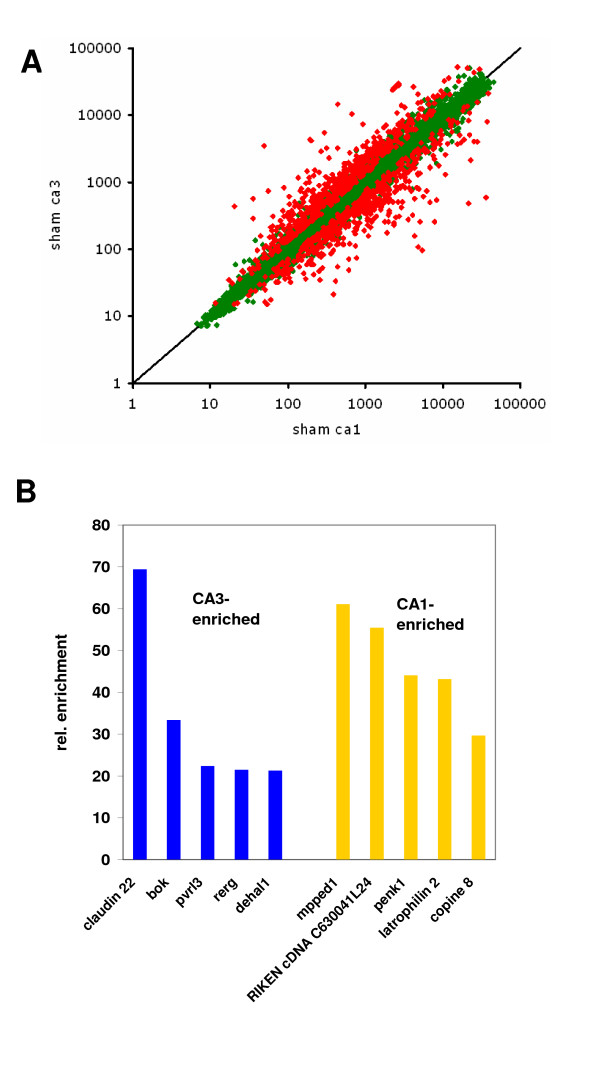
**CA1 and CA3 neurons display a different gene expression repertoire in the native state**. Result of the comparison of CA3 and CA1 regions in the native animal. A, We detect 1035 genes that are differentially expressed in the CA3 and CA1 region in sham-treated animals, 566 are significantly enriched in CA3, 487 in CA1 (p_fdr _< 0.05; differentially regulated genes in red). B, Bar graph showing enrichment factors for the 5 most differentially expressed genes in CA3 and CA1. The enrichment factors reflect relative abundance of gene expression in CA3 over CA1 or vice versa, and were calculated by dividing the mean of the gene abundance in CA3 by CA1 or vice versa. Bok, Bcl-2 related ovarian killer; pvrl3, poliovirus receptor-related 3; rerg, RAS-like, estrogen-regulated; dehal1, Iodotyrosine dehalogenase 1; mpped1, metallophosphoesterase domain containing 1; penk1, preproenkephalin 1.

We searched for genes with stronger expression in CA3 or CA1 by direct competitive hybridization of 4 samples each. A total of 1035 genes were found with 566 being stronger expressed in CA3, and 487 in CA1 (Fig. 2A; full list in Additional file 2) (p_fdr _< 0.05).

The five most prominent genes overexpressed in CA3 or CA1 are given in Fig. 2B: The gene with the strongest differential expression in CA3 was claudin 22, a protein of the claudin family which is involved in tight junction formation [15]. There are indeed indications that puncta adhaerentia at the mossy fiber – CA3 dendrite synaptic sites contain ZO-1, a protein that connects claudin to the actin cytoskeleton [16]. It is thus likely, that claudin 22 plays a CA3-specific role at these synapses.

Bok (Bcl-2 related ovarian killer) is a pro-apoptotic member of the Bcl family. In dividing cells, Bok is regulated by the cell cycle [17-19]. Its function has however been primarily studied in reproductive tissue, and nothing is known about its role in neurons.

Pvrl3 (or PRR3 or Nectin3) is the poliovirus-virus related receptor 3. This group of genes is involved in cell-cell interactions [20,21], and Nectin3 has been shown to be critical for the formation of puncta adherens junctions (PAJs) between CA3 dendrites and mossy fibres [16,22,23]. It is interesting to find two genes belonging to the same protein complex among the 5 most enriched genes in CA3 vs. CA1.

Rerg is a RAS-related tumor suppressor gene first identified in breast cancer [24,25]. Its role in the brain and the CA3 region is completely unknown at present. Finally, DEHAL1 (Iodotyrosine dehalogenase 1) is a gene involved in deiodination of mono and diiodotyrosines [16,22,26]. The potential function of this protein in CA3 is completely enigmatic at present.

The gene most exclusively expressed in CA1 is mpped1 (metallophosphoesterase domain containing 1; NM_172610). There is no information available on this protein in the databases. Also, the second gene found here is an unknown EST (NM_183136). A gene enriched by a factor of 44-fold was preproenkephalin (NM_001002927). The CA1-selective expression of this gene is again confirmed on the in-situ level by the systematic gene-brain-mapping database BGEM. Latrophilin 2 is one of the receptors for alpha-latrotoxin [27]. While latrophilin 1 seems to be expressed in all hippocampal subfields there is not much information available on the role of latrophilin 2. Copines finally are a class of proteins with Ca^2+ ^and phospholipid-binding properties that are thought to be involved in membrane trafficking [28]. However, very little specific information on their role in the brain is available.

### GO analysis of CA3 vs. CA1 in the native state

**Figure 3:**
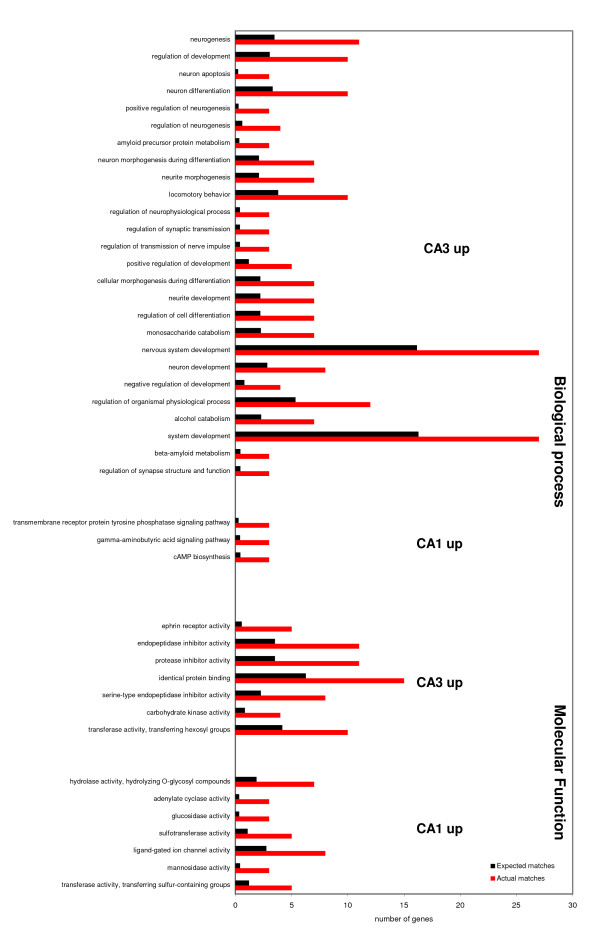
**Gene ontology analysis for CA3- and CA1-enriched genes in the native state**. Bar graph depicting gene ontology (GO) analysis of the genes significantly overexpressed in CA3 or CA1. The upper part of the graph shows the GO category "Biological process", the lower part "Molecular function". GO analysis were performed using the web-based L2L tool [29]. For "Biological process" there is a strong emphasis on terms related to neuron differentiation, synaptic function, and energy metabolism in CA3, and to GABA-signaling in CA1. For "Molecular function" we find enrichment of groups like ephrin receptors, serin-type protease inhibitors, and carbohydrate moiety transfer activities. In contrast in CA1 we observe an enrichment in deglycosylating activity, ligand-gated ion channels, and sulfotransferase activity. Black, expected matches; red, observed matches.

We next performed a gene ontology analysis in the categories biological process and molecular function to systematically assess relevant funtional gene groups. Gene ontology analysis with the L2l-tool [29] demonstrated a number of functional groups that were significantly overrepresented in the CA3-enriched genes (Fig. 3). Several main topics appear evident when looking at biological process categories: First, there are several large gene groups relating to differentiation: "neuron differentiation", "nervous system development", "regulation of differentiation". Second, there is a small group of genes classified as "regulation of synaptic transmission" and similar categories. Third, there are two groups related to cellular catabolism: "alcohol catabolism" and "monosaccharide catabolism". Interestingly, there are also two categories relating to "amyloid precursor protein metabolism". For the genes relatively upregulated in CA1, we find only three categories in the biological process category enriched, most notably the "gamma-aminobutyric acid signaling pathway", with the GABA-A receptor subunits alpha 1 and 3, and beta 2, "cAMP biogenesis", and "transmembrane receptor tyrosine phosphatase signaling pathway".

This differential enrichment of gene categories suggests a much higher degree of plasticity mechanisms of CA3 neurons in the hippocampus compared to CA1 that are reflected by large groups of genes in categories related to "differentiation" or "neurite morphogenesis", and by "regulation of synaptic transmission". The second theme cluster refers to energy generation (e.g. "monosaccharide catabolism") harbouring genes like hexokinase 2, phosphofructokinase, or fructose-bisphosphate aldolase. This suggests a higher enrichment of genes involved in energy generation, and may provide one explanation for a higher resistance against hypoxic/ischemic events in CA3 neurons.

For the GO category "Molecular function" we also find a remarkable difference between CA3 and CA1 (Fig. 3, lower part): CA3-enriched categories include "ephrin receptor activity", "endopeptidase inhibitor activity", "carbohydrate kinase activity", and "transferase activity, transferring hexosyl groups". Particularly interesting are the "endopeptidase inhibitor activity" that harbours among others 5 different serpin-type endopeptidase inhibitors (SerpinB9, SerpinF1, SerpinA7, SerpinG1, and Spink1). A number of reports finds neuroprotective functions of different serpins in models of cerebral ischemia [30,31]. "Carbohydrate kinase activity" again identifies genes involved in energy metabolism such as 6-phosphofructo-2-kinase/fructose-2,6-biphosphatase 3, hexokinase 2, or phosphofructokinase. The group "transferase activity, transferring hexosyl groups" contains a total of 10 genes involved in carbohadrate transfer and proteoglykan modifikation (e.g. N-acetylgalactosaminyltransferase 14 (GalNAc-T14), fucosyltransferase 8, mannosyl (alpha-1,6-)-glycoprotein beta-1,6-N-acetyl-glucosaminyltransferase), and suggests a high acitivity in CA3 neurons in modulating the cell surface and the extracellular space. Finally, the category "ephrin receptor activity" (contains EPHA3, EPHA4, EPHA5, EPHA7, EPHB1) underlines the notion of a high degree of plasticity potential in CA3, e.g. in axon guidance.

CA1-enriched categories include: "hydrolase activity, hydrolyzing O-glycosyl compounds", "adenylate cyclase activity","glucosidase activity","sulfotransferase activity", "ligand-gated ion channel activity", and "mannosidase activity". Enrichment of "Adenylate cyclase activity" is derived from CA1-overexpression of adenylate cyclases 1, 6 and 8. "Ligand-gated ion channel activity" contains the already mentioned GABA-A receptor subunits, the alpha 5 subunit of the nicotinic cholinergic receptor, or the TRP channels, subtype C, members 3 and 4. Interestingly, while CA3 has an enrichment in carbohydrate moiety addition, CA1 is enriched in genes that remove carbohydrate chains, such as glucosidases and mannosidases. In addition, CA1 is significantly enriched in a number of sulfotransferases.

We then asked the question whether the differential gene expression pattern observed here has any potential commonalities to gene lists in the literature. Using the L2L tool we discovered significant overlaps with 10 out of 811 lists. First, there is a significant overlap with genes down-regulated in the hippocampus with aging (two lists detected) [32]. Among these genes were synapsin II and the Eph receptor A4, likely relating to declining plasticity mechanisms in CA3. Second, there was a remarkable overlap with hypoxia-induced genes [33]: BCL2/adenovirus E1B 19 kDa interacting protein 3 (BNIP3), hexokinase 2, phosphoribosyl pyrophosphate synthetase 1, platelet phosphofructokinase, enolase 1 (alpha), endothelin 2, fructose-bisphosphate aldolase A, and the prostaglandin-endoperoxide synthase 2 (prostaglandin G/H synthase and cyclooxygenase). This overlap encourages the notion from above that genes are enriched in CA3 whose relative overexpression in the native state of the pyramidal cells offers protection against subsequent hypoxia. The relative overexpression of genes involved in glcolysis (hexokinase2, phosphofructokinase, and fructose-bisphosphate aldolase) supports the enrichment of genes related to ATP generation found in the gene ontology analysis above. It seems that CA3 neurons have a higher reserve of energy generation in contrast to CA1 which provides an attractive hypothesis for their relative resistance to hypoxia.

### Confirmation of differential gene expression

**Figure 4:**
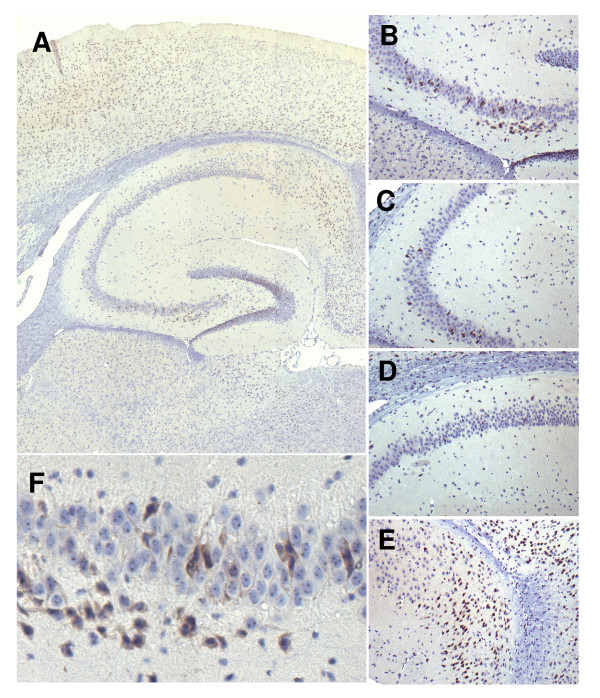
**The Bok-protein is localized to CA3**. Micrographs showing immunohistochemical detection of Bok (Bcl-2 related ovarian killer) protein expression in the hippocampus of C57/bl6 mice. A, Overview of the hippocampal formation (original magnification (OM) 10×). Bok preferentially localizes to th CA3 subfield, with some staining in the dentate gyrus. Apart from the hippocampus stining is visible in the cortex. B,C,D,E Staining in the CA3 subfield (B), in the CA3-CA2 transition (C), in the CA1 field (D), and in the CA1-subiculum transition (E) (OM 20×). In CA2 only few single neurons are detectable that stain positive for Bok, in CA1 staining is absent and is evident again in the subiculum. F, Close-up of CA3 neurons (OM 60×). Although quite specifically localized to CA3 Bok displays a patchy staining of neurons within this subfield.

To exemplarily confirm the regional-specific gene expression found we performed immunostaining for the Bcl-family member Bok (Fig. 4). In the hippocampus we find strong, but cellularly heterogenous expression in the CA3 field. There are single positive cells to be found in the CA2 region, and none in CA1. Bok is expressed again in the subiculum, and in the dentate gyrus. Apart from the hippocampal formation, Bok is expressed in the cerebral cortex. Thus, we could confirm the differential expression pattern of one exemplary gene on the protein level.

A second, more systematic approach to verifying our results would be a comparison to databases harbouring hippocampal expression patterns. One project that uses a systematic approach towards localization of gene expression in the brain is BGEM (Brain Gene Expression Map [4]). This repository provides a selection of in-situ hybridization of over 2400 genes, which have been selected by neuroscientists or by their expression in the brain [4]. For our purposes, the selection of genes can however be regarded as random. We have manually compared selected overrepresented genes in CA3 or CA1 to this database.

In the list of the 5 most overrepresented genes in CA3 we find Bok, pvrl3 and for CA1 penk. All genes showed the selective hippocampal subfield expression predicted by our data. Additional file 3 shows a selection of genes that are found among the 1035 differentially expressed genes and the BGEM database, and confirm the expected subfield distribution between CA3 and CA1. The in-situ hybridizations also nicely correlate with the induction factor found with respect to the relative staining intensities of both areas.

Similar confirmation of our data could be obtained by querying another web-based in-situ-hybridization repository of the brain, the Allen brain atlas [34]. We confirmed cell-type specific enrichment for a number of regulated genes that were not contained in the BGEM set, for example the GABA-A receptor subunit alpha1 and others (Additional file 3). These comparisons are admittedly hampered by the difficulty in querying these databases, and extracting informations about the coverage of our gene lists by these collections, as well as by the need to visually inspect all images. Thus we cannot estimate the likely false positive or negative rate for our lists from these comparisons at the moment. However, it is noteworthy that we did not detect any false positives by this manual approach.

Another approach to systematically check the validity of our data is comparison to similar studies. Indeed, a number of data have been published on differences of gene expression in the native state in the hippocampus, the most thorough by Lein and colleagues [35]. There is a high degree of overlap in genes identified as being overrepresented in either CA3 or CA1. We identified the nephroblastoma-overexpressed gene (Nov) in our gene lists as an CA1-enriched gene (CA1/CA1 sham ratio 2.29) which was verified by Lein et al. using in-situ hybridization. In CA3, one of the highest hits in our list was the Bcl2-related ovarian killer (Bok; relative regulation 33.4-fold compared to CA1) that was also found and verified by Lein et al Other genes that were commonly identified by both approaches were the transforming growth factor beta 2 (Tgfb2), protein kinase C, delta (Prkcd), myosin Vb (Myo5b), copine 9 (NM_170673.3) (versus copine 6 in the Lein screen). In summary, we could demonstrate a high reliability of our approach based on RNA-amplification.

### Genes regulated in CA3 and CA1 by hypoxia/ischemia

**Figure 5:**
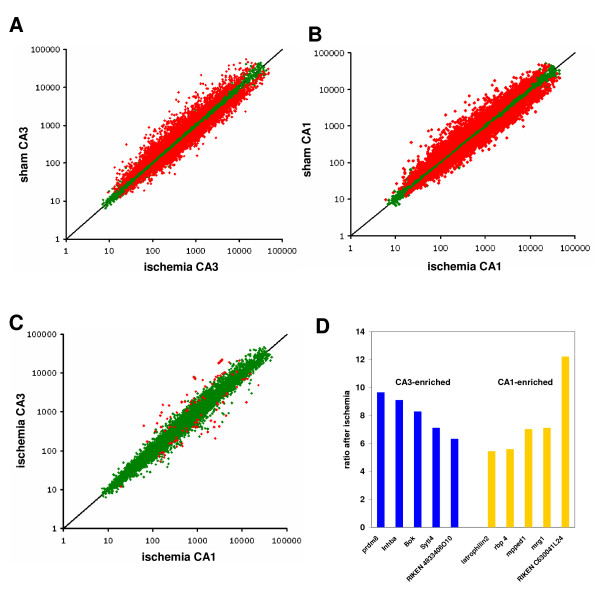
**Gene expression in CA3 versus CA1 after ischemia**. A,B Scatterplots showing the results of direct competitive hybridizations of both subfields from ischemic animals and the non-ischemic controls (means of n = 4 experiments for each region). In each of the hippocampal subregions more than 5000 genes could be identified exhibiting differential expression upon ischemia. A. 5243 differentially expressed genes were detected in the CA3 region. B. 5511 differentially expressed genes were detected in the CA1 region. Red: significantly regulated genes (p_fdr _< 0.05; differentially regulated genes in blue). C, A total of 97 genes is significantly different between ischemic CA3 and ischemic CA1 regions (scatterplot; blue are significantly regulated genes p_fdr _< 0.05). D, Bar graph showing the 5 most different genes with preference for CA3 or CA1 with their relative enrichment factors (prdma, pr-domain containing protein 8; Inhba, Inhibin beta A; Bok, Bcl-2 related ovarian killer; Sytl4, synaptotagmin-like 4; rbp 4, retinol binding protein 4; mpped1, metallophosphoesterase domain containing 1; mrg1, myeloid ecotropic viral integration site-related gene 1, alternative names: meis2, stra10).

Comparing ischemic samples from CA3 or CA1 to their respective normoxic controls yielded a large number of regulated genes. In CA3, a total of 5243 genes were found to be regulated by ischemia (p_fdr _< 0.05; see Additional file 4). Of those, 2559 were upregulated by ischemia, and 2619 downregulated (Fig. 5A). GO analysis revealed only two terms in biological process that were overrepresented,"microspike biogenesis" and "response to inorganic substance". Comparison to regulated gene lists found overlaps with a variety of stress-induced paradigms (e.g. heatshock, UV-irradiation), and genes induced by TNFa-treatment, NF-kB or p53. The list of overlapping genes with p53 induction harbours genes such as cyclin-dependent kinase inhibitor 1A (p21, Cip1), Fas ligand, immediate early response 3, or the growth arrest and DNA-damage-inducible, alpha (GADD45A). In CA1 we find 5511 genes altered, 2649 of those upregulated by ischemia (Fig. 5B; see Additional file 5).

Comparing both lists of significantly regulated genes, we find a surprising overlap of 4070 genes at p_fdr _< 0.05, 1173 only regulated in CA3, and 1441 only regulated in CA1. However, the significance cutoff at p_fdr _< 0.05 is arbitrary, and the exclusiveness of genes in the non-overlapping set is relative. Indeed, the impression of a co-regulation of genes in CA1 and CA3 by hypoxia/ischemia is overwhelming, with a high correlation (Fig. 5C; R^2 ^of 0.84). The slope of the correlation equation is essentially 1, implying that there is no systematic difference in the gene regulation of CA1 and CA3 neurons confronted with the ischemic stimulus. Using direct comparison of ischemic CA3 or CA1 samples, only 97 genes were found to be differentially expressed in CA3 or CA1 under ischemic conditions (red, p_fdr _< 0.05; Fig. 5C; full list in Additional file 6). Of those, 38 were stronger regulated in CA3 24 h after ischemia, and 59 in CA1. Fig. 5D shows the 5 most strongly enriched genes in CA3 and CA1, respectively.

Interestingly, the majority of genes different in the ischemic state were genes that had already been detected as different in the normoxic state (total of 65 genes), while 51 genes overlapped with genes induced by ischemia in CA3 and/or CA1.

In order to further validate the lists of genes identified we compared regulation factors for genes for the sham or ischemia group obtained from the array analyses to quantitative PCR (see Additional file 7). We find a good correlation between both assessment methods (Pearson correlation coefficient 0.75). We also performed immunohistochemistry on one gene regulated in both sham and ischemia (Sytl4), and confirmed the distribution preference to CA3 (see Additional file 8).

Gene ontology overrepresentation analysis revealed that functional gene groups that were relatively upregulated in CA3 include categories such as "synaptic transmission", "cell-cell signaling", "neurotransmitter secretion", but also "axon guidance" and "nervous system development" under biological process. Under molecular function, there are two enriched categories, "peroxidase function" and "calcium binding". One of the two genes contained in "peroxidase function" is interestingly a peroxidasin homologue (A_51_P300456, AK122223), a protein that has so far not been studied in a mammalian setting [36]. This gene category may be relevant for counteracting oxygen radical formation, one pathomechanism in cerebral ischemia. In the CA1 overrepresented genes we only found one category enriched, "retinol binding" under molecular function. This category contained only 2 genes, retinol-binding protein 1 and 4.

Thus, there is a major difference in the groups of genes that are relatively overrepresented in the two hippocampal regions after ischemia, however, the number of differentially expressed genes in CA1 and CA3 under ischemic conditions is unexpectedly low.

### Ischemia attenuates gene expression differences between CA3 and CA1

**Figure 6:**
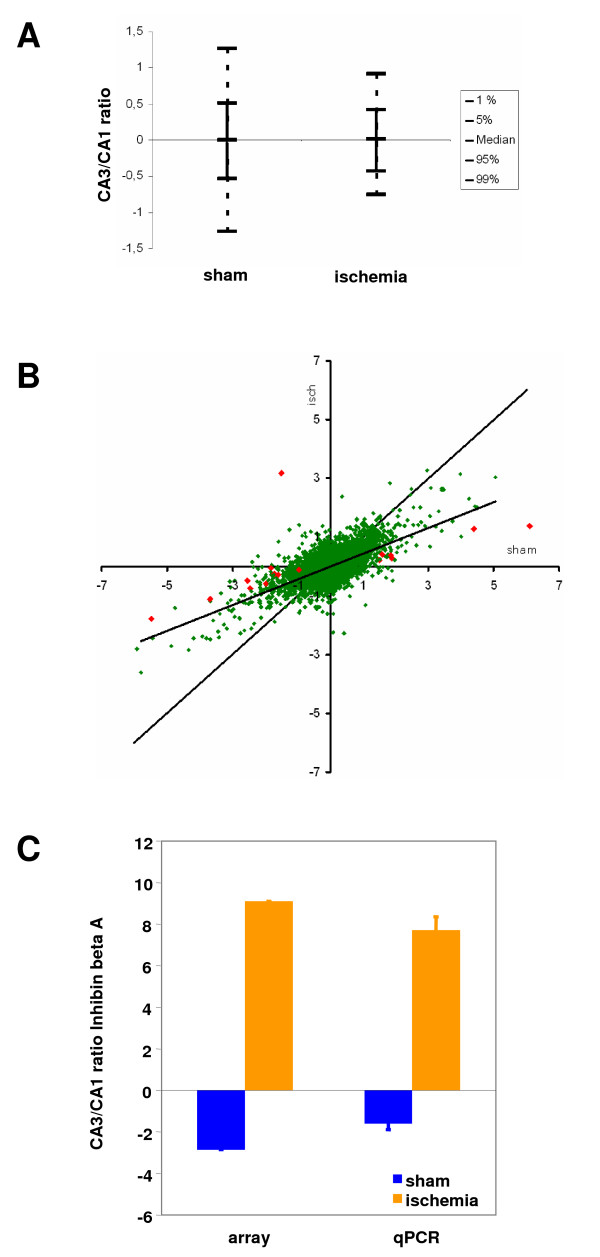
**Ischemia blunts the gene expression differences in CA3 and CA1**. A, Box-Whisker plot showing the narrowing of CA3/CA1 ratios in ischemic vs. naive animals. B, Plotting CA3/CA1 ratios in ischemia vs. sham reveals deviation from the diagonal. Difference ratios are attenuated by ischemia regardless of preferential CA3 or CA1 expression (red: p_fdr _< 0.05). C, Inhibin beta A displays the highest change in preference between the native and ischemic state. The gene expression preference actually reverses from higher expression in CA1 in the native brain to higher expression in CA3 in the ischemic state. Verification with quantitative PCR shows comparable values to the array-derived ratios.

We asked what the reasons for this apparent overall disappearance of individualitistic gene expression profiles of these two cell types under ischemic conditions were, while the ischemic stimulus itself produced massive gene expression changes in both cell types. One technical reason could have been a strong increase in statistical variance in the ischemic samples caused by adding the experimental variance to the basic biological fluctuation between individual mice, and thus strongly decreasing power for the detection of differences. However, the distribution of standard deviations in both groups was highly similar, and cannot account for a 10-fold decrease in power (97 vs. 1035 genes detected at the same significance level) [37] (see Additional file 9). Indeed, the spread of CA3/CA1-ratios was broader in the normoxic than in the ischemic situation (Fig. 6A), suggesting that the major cause of reduction in the number of cell-type-specifically enriched genes was truely caused by a relative convergence of gene expression in these two cell types. We contrasted CA3/CA1 ratios in the normoxic and ischemic state (Fig. 6B). The regression line deviates strongly from the diagonal (slope 0.44; R^2 ^= 0.34), reflecting the broader spread of CA3/CA1 ratios in the normoxic situation. The vertical compressive deformation of the scatter plot also appears symmetrical, implying that the loss of relative gene expression enrichment is equally affecting CA3 and CA1. While this compression effect under ischemic conditions affects the broad majority of genes, only 15 discrete genes demonstrate a significantly divergent behaviour under normoxic and hypoxic conditions with regard to their CA3 or CA1 preference (see Additional file 10). In nearly all cases, this relative shift is caused by attenuation of the extent of differential preference under ischemia. Several genes display a particularly impressive shift: Proenkephalin is 44-fold more abundant in CA1 in the native state, but looses this strong preference in ischemia (only 3-fold more abundant in CA1). Proenkephalin has been characterized as an immediate-early gene in the hippocampus in various seizure models [38-40], we did however not find data concerning effects of proenkephalin on neuronal survival or possible functions in cerebral ischemia. The gene with the most prominent change in the other direction is claudin-22. This gene looses CA3-specificity with ischemia (69-fold overexpression in CA3 in the normoxic vs. 2.6-fold overexpression in the ischemic state). Claudin-22 is also the gene with the highest overall specificity to the CA3 region, and has been implicated in puncta adhaerentia junction formation between CA3 pyramidal cells and mossy fibers (see above). There are no links to ischemia at present. The most interesting, however singular, case is Inhibin beta A (Inhba) (A_51_P239750; NM_008380), where ischemia led to a full reversal of the expression preference from CA1- to CA3-neurons (from a relative overexpression of 2.86-fold in the CA1-area under normoxic conditions to an overexpression of 9.1-fold in the CA3-region under ischemic conditions (total relative change 26-fold)). This change was caused by a concomitant upregulation in CA3 (5.02-fold), and a downregulation in CA1 (0.55-fold) by ischemia. This is also the highest overall change in the relative distribution of gene expression noted in CA3 and CA1. We verified this behaviour by quantitative PCR for Inhibin beta A (Fig. 6C), and find good correlation. Inhibin/Activin beta A induction has indeed been previously observed in global gene expression analysis [41-43]. Induction of activin appears to be involved in the neuroprotective effects of bFGF [44], and thus the reversal of expression from CA1 to CA3 after an ischemic stimulus is one attractive candidate for mediating the ischemia tolerance of CA3 neurons.

In summary, contrary to our expectations, and despite massive induction of gene expression changes by ischemia in two distinct cell types, individual gene expression differences between CA3 and CA1 are strongly diminished under ischemic conditions by non-directional changes in CA3 and CA1.

## Discussion

### Characteristics of our approach

We have aimed at comparing the functional genome in two subsections of the hippocampus harbouring diverse neuron types in the native state and after a strong transcriptional stimulus using laser microdissection and RNA amplification with a highly linear protocol [6,7,45]. We have chosen CA3 and CA1 fields as these fields are well-studied concerning their differing physiology, are situated in close proximity in the brain, and are readily separable from each other (e.g. without using immunohistochemical markers). Although we have not excised single neurons from the two areas, we have carefully excised the pyramidal cell layers that harbour neurons at high density with relatively few non-neuronal cell types (e.g. astrocytes or endothelial cells). About 10% of the neurons in the pyramidal layer are estimated to be interneurons. We therefore assume that the vast majority of genes identified as differeing between the two regions are of neuronal origin. This assumption is backed for example by the clearly neuronal origin of the majority of functional gene ontology groups identified. Also, although a small percentage of non-neuronal cell types are present, these are unlikely to differ in their gene expression between CA1 and CA3 as is expected from the neurons, and therefore are unlikely to contribute to the differences observed here. Comparable studies using even less sophisticated approaches (e.g. manual dissection such as Lein et al. [35]) have yielded a set of purely neuronal genes.

As strong transcriptional stimulus we have chosen hypoxia/ischemia, as these two hippocampal fields are known to display differing vulnerabilities to this insult, and ischemia is one of the strongest stimuli known in the brain, thus likely providing statistically robust differences. We used two-color hybridization, and directly cross-compared samples from CA3 and CA1 in the native and ischemic state in all combinations.

The main questions we sought to approach with this study was how the functional genome of two neuron types differs in the native state, changes after an ischemic stimulus, and if and how these changes are related.

### Validation of datasets

Regulated datasets have been extracted from the array experiment using stringent statistical criteria (false discovery rate correction by the Benjamini-Hochberg method). We sought to show the validity of the extracted genes by three approaches: First, we compared genes regulated in the non-ischemic situation by comparison to online in-situ-hybridization databases, where we have validated the differential expression of over 10% of the genes detected in this dataset by visual inspection. Second, we have verified the differential expression of a total of 9 genes from the non-ischemic and ischemic datasets by quantitative PCR, with generally good accordance of observed direction and extent of regulation. Third, we have performed immunohistochemistry for two genes. Overall we believe that these approaches provide an excellent trust basis for our lists. It has to be noted however that all verification approaches are based on exemplary and random sampling of genes, and do not provide proof in a systematic and statistically valid way that all genes in the lists are differentially regulated. Indeed, we expect 5% false positives in our lists according to the Benjamini-Hochberg method. Also, we have not approached the more difficult question of false negatives. This does however not impact on the validity of the general conclusions drawn from the study, since false negatives and positives are expected to be governed by random factors.

### Ischemic model

We chose the Vannucci model of a combined global hypoxia and hemispheric ischemia, as this is more robust than other models (e.g. filament model MCAO), and reliably affects the whole hippocampus. We have carefully optimized hypoxia times (see methods section) to produce a highly reproducible hemispheric ischemia with hippocampal involvement and tolerable mortality. This indeed resulted in an acceptable variance between ischemic samples. In contrast to global ischemic model (e.g. 2-vessel occlusion with hypotonia [46]) this model also produces a full infarct that is readily detectable by TTC-staining.

### Comparison to similar approaches

We have conducted a detailed gene expression analysis of CA1 and CA3 gene expression in the native state, and the first systematic analysis of gene expression under ischemia. Moreover, our study is the first attempt to dissect the status quo of gene expression in two similar hippocampal structures harbouring different neurons under native conditions and after a strong transcriptional stimulus such as hypoxia/ischemia, and relate these to changes in the individual regions.

Regarding gene expression differences in the native state, a number of labs have undertaken systematic approaches to dissect gene expression in hippocampal subfields in man and rodents [35,47-51]. Ginsberg and Che have studied CA3 and CA1 differences in human post-mortem tissue [50]. Similar to our approach, they have microdissected neurons from CA3 or CA1 using LMD. They report a total of 20 genes changed, among these GABRA1 and GABRA2 as overexpressed genes in CA1, corroborating our data for the human situation. The authors have however used a very limited DNA array (~120 genes contained). A similar study was undertaken by Torres-Munoz and colleagues, also by using microdissection of single neurons from CA3 or CA1 from post-mortem brains [49]. The authors report a total of 60 genes significantly overrepresented in CA3 or CA1. The study by Bonaventure and colleagues compares microdissected nuclei and CA1 and CA3 in Wistar rats [47]. It is however unclear from the paper how many genes were found to be statistically different between CA1 and CA3. Of the five genes given in the paper as most enriched in CA3 or CA1, we find RGS14 in our list of 1035 significantly changed genes also as an CA1-enriched gene product. A comprehensive analysis was performed by Lein et al. [35,52]. Lein et al. have manually dissected hippocampal subregions from the mouse (CA1, CA2, CA3, DG), and categorized gene expression according to the preferential expression. A total of 104 genes were selected according to their relative rank in pattern formation and their preferential distribution to different regions of the hippocampus verified by in-situ-hybridization. As the main purpose of this study was to find prominent marker genes for the hippocampal subregions, the authors have not used rigorous parametric statistics to determine the totality of genes that is changed, but rather used manual inspections of patterns for selection of the 104 most different genes. In contrast, we define the population of genes that are significantly changed in the functional genome by stringent statistical criteria (p_fdr _< 0.05). Also, we use LMD for dissection which is more accurate than manual dissection. There is a high degree of overlap in the genes reported by the authors and in our study, cross-validating both approaches. A very similar approach to ours was taken by Datson et al. [51]. The authors compared gene expression in the dentate gyrus to the CA3 region using laser microdissection and Affymetrix chip hybridization with rigorous statistical testing. They report a similar number of differentially expressed genes (724) to our study (1035).

Regarding the question of differences under ischemia, a number of labs have looked at single gene product distributions to the hippocampal subfields CA3 and CA1 after ischemia [53-62], however, no one has undertaken a systematic attempt to define differences in CA1 and CA3 in the ischemic state.

### Ischemia dampens genomic differences between neuronal cell types

In the native state, there is a substantial degree of difference in gene expression (>1000 genes differentially expressed between CA3 and CA1) which likely forms the basis for the differing physiological and connective properties of these cells. Our lists of differentially expressed genes now provides a large dataset for generating and testing new hypotheses on the origin of differing properties of these cell types.

We have used a strong transcriptional stimulus to study the behaviour of the functional genome of two similar neuronal cell types to a common perturbation. The ischemic paradigm is particularly well-suited because it is one of the strongest stimuli known for changing gene expression in the brain, and because these two cell types display differential vulnerability to ischemic insults, with the CA3 neurons being more resistant. The number of genes detected as significantly differing in their abundance in CA3 vs CA1 was unexpectedly lower in the ischemic state (97 genes) than under normoxic conditions (1035 genes). The majority of the genes detected was also preferentially enriched in CA3 or CA1 under native conditions. This dampening of cell-type specific gene expression profiles appears counterintuitive in view of the massive induction of gene expression changes in each individual neuron type by the ischemic stimulus (both > 5000 genes changed, i.e. app. 25% of the functional genome present on the DNA array). However, this massive induction is indeed the explanation for this behaviour: Obviously ischemia evokes a conserved, largely (neuronal) cell-type-independent program in gene expression changes, which attenuates pre-existing differences.

An alternative explanation for the disappearance of cell-type specificity in gene expression in the hippocampus would be centered on a general shut-down of transcription in damaged neurons that would thus eliminate differences. However, we and others have done numerous studies in different ischemic paradigms, and it is believed that ischemia is one of the strongest transcriptional stimuli known in the brain and in neurons. This is indeed underlined by the strong ischemia-induced regulation of gene expression in this study (close to 25% of the scanned genome was altered, both up- and downregulated). One could however argue that these changes are not derived from neurons, but from non-neuronal cells that contaminate the pyramidal layers. This would consequently imply that one would primarily detect regulated genes of non-neuronal origin under ischemic conditions. However, the major functional groups (GO-term) regulated are neuronal, such as "synaptic transmission", "neurotransmitter secretion", "axon guidance" or "nervous system development". And among the most differing genes in CA3 and CA1 under ischemic conditions are genes like synaptotagmin-like 4, or latrophilin-2 which are exclusively neuronal in origin. We therefore believe that the explanation for the observed dampening of field-specific differences is indeed due to the activation of a common transcriptional program rather than to shutting down transcription.

To broaden this important conclusion it will be necessary in the future to examine a much larger number of different neuron types. These results also may have implications for therapeutic strategies in ischemic diseases of the brain: It would be advantageous to select target mechanisms for pharmacological interference from the pathways and elements that are common to different (neuronal) cell types.

Returning to one initial question of the genomic basis for the physiological and pathophysiological differences between two types of neurons, there is a large repertoire of functional genomic differences genes and pathways in the native state that can form the basis for correlating physiological properties of these two cell types. However, single gene candidates for the differing susceptibility of CA3 and CA1 neurons are limited (<100), and these are certainly attractive entry points for detecting new targets in ischemic diseases of the brain.

## Conclusion

By using laser microdissection and RNA amplification we could identify the differential functional genome under normoxic and hypoxic conditions in the two hippocampal subfields likely forming the basis of differing physiological and pathophysiological behaviour of these cells. Our approach was successful, and lends itself to replication with different paradigms. We have provided the most thorough and rigid catalogue to date of gene expression differences in the CA3 and CA1 subfield of the hippocampus, both under native conditions, as well as after a strong ischemic stimulus. These data will be both useful for brain mapping projects, as well as for researchers studying the physiology of the hippocampus (e.g. in learning and memory paradigms). Moreover, our datasets are of considerable value for research into ischemic pathophysiology.

The most surprising outcome of this analyses is however that the strong transcriptional stimulus hypoxia/ischemia leads to a substantial reduction in pre-existing cell-type-specific gene expression patterns, instead of generating new or enhancing existing differences.

## Methods

### Ischemic model

Hypoxia-ischemia was induced in 3-month- old male C57BL6 mice by a modification of the procedure developed by Vannucci and co-workers [12,13]. Animals were anaesthetized with halothane (4% in 70% N_2_O: 30% O_2_). A small incision was made in the neck and the right common carotid artery was exposed and ligated with 4–0 surgical silk in three separate locations. The incision was closed, and the animal allowed to recover with access to food and water for 3 hours. Prior to exposure to hypoxia, mice were placed in a 35.5°C water bath for 10 minutes followed by hypoxic exposure which comprised 10 min (6% O_2_/94%N_2_) and 25 min (8% O_2_/92%N_2_). At the end of the hypoxic interval, animals were allowed to recover in room air for 30 min and then return to their cage with free access to food and water for 24 houres. Animals were re-anaesthetized with Halothane, transcardially perfused with HBSS, decapitated, and brains were frozen on dry ice. During establishing of the protocol a number of animals were examined by TTC-staining to determine ischemic damage. The morphology of the pyramidal cell layer after the Vannucci model remains intact after 24 hrs. Animal procedures adhered to German ethical standards and were approved by the relevant regulatory authorities (Regierungspräsidium Karlsruhe).

### Laser Microdissection

Mouse brains were cut into 8 μm thick horizontal sections using a cryotome (Leica). For identification of the pyramidal cell layers sections were stained with Thionin (0.5% in a 0.066 M Na-acetate buffer). Air-dried sections were immersed for 30 seconds, and rinsed once with water. For laser microdissection we selected three consecutive horizontal sections (Bregma as zero reference: -5.64; interaural connection as zero reference: 4.36). Laser microdissection (Leica LMD) was used to dissect the CA1 and CA3 pyramidal layers identified by comparison to stereotaxic atlas data. Excised regions from 3 consecutive sections were collected together, and further processed. N = 4 brains per group were used.

### RNA amplification

Each RNA sample was linearly amplified by two consecutive rounds of T7 polymerase-driven amplification [63]. The precipitated total RNA of each LMD sample was reverse transcribed with a first-strand synthesis mix comprising 10 U Superscript III (Invitrogen, Karlsruhe, Germany), 1 U Superasin (Ambion, Huntingdon, UK) and 0.5 pmol of T7-T18V primer by incubating for 2 h at 50°C after second-strand synthesis with 6 U DNA polymerase I, 2 h at 22°C with 0.2 U RNaseH, and 15 min at 16°C with 4 U T4 DNA polymerase I (Invitrogen). The T7 promoter-tagged cDNA template was purified by standard phenol extraction and ethanol precipitation. This cDNA was used for first round amplified antisense RNA production applying a MegaScript kit (Ambion) and purified with an RNeasy Mini Kit (Qiagen) according to the manufacturer's instructions. Second round amplification was performed accordingly but using T7-tagged random primer for first-strand cDNA synthesis and T18V primer for second-strand synthesis, yielding amplified sense RNA. UV measurements at 280 and 260 nm and 2100 Bioanalyzer runs using the RNA-6000 Nano-LabChip (Agilent, Böblingen, Germany) were used to ensure the quality and quantity of the sense RNA produced. The yield varied from 40 to 80 μg. As RNA-marker we used the RNA-ladder 6000 (Ambion), with the following sizes: 0.2, 0.5, 1, 2, 4, and 6 kb.

### Microarray analysis

The sense RNA samples were fluorescently labelled using the CyScribe post-labelling kit (Amersham Biosciences, Freiburg, Germany) according to the manufacturer's instructions. Subsequently, the corresponding CA1 and CA3 samples were hybridized pairwise to DNA arrays harbouring 21.318 gene probes as 60 mer-oligonucleotides (#G4121A, Agilent). Accordingly, the corresponding sham and ischemia samples were also hybridised pairwise. Hybridization was performed following the instructions of the array manufacturer but using 2 μg labelled probe. All arrays were analysed with the Agilent DNA array scanner and the ARRAYVISION V6.0 image software (Imaging Research, St Catharines, Canada).

### Statistical analysis

Data were log-transformed and normalized using the Lowess algorithm. Statistical evaluation of the expression changes was performed in the "R" statistical environment (version 1.091) by using the BioConductor packages "marray" [64] for data access and primary analysis and the package "limma" which implements a moderate t-test based on Bayesian statistic. This moderate t-test allows for a comparably reliable estimation of the SD even if only few replicates are available. The resulting p-value was corrected for multiple testing using the false discovery rate method (FDR) of Benjamini and Hochberg [65]. P-values were adjusted to a false-discovery rate of 5 %.

### Quantitative PCR

RNA of microdissected brain regions was amplified as described [45]. cDNA was synthesized from 5 μg amplified RNA (2 rounds) using oligodT primers, superscript II reverse transcriptase (Gibco) using standard conditions. Quantitative PCR was performed using the Lightcycler system (Roche Diagnostics, Mannheim, Germany) with SYBR-green staining of DNA double-strands. Cycling conditions were as follows: 5 min 95°C, 5 sec 95°C, 10 sec 60°C, 30 sec 72°C; 10 sec 84°C for 45 cycles for Inhibin beta-2as and cyclophilin. The same annealing temperature was used for the amplification of Protocadherin 8 (Pcdh8), Neuritin 1 (Nrn1), and Spondin 1 (Spon1), but measurement was carried out at 76°C melting temperature. An annealing temperature of 62°C and a melting temperature of 79°C was used for Calbindin 28 K (Calb1) and RAS-like, estrogen-regulated, growth inhibitor (Rerg). As cycling conditions for measurement of Synaptotagmin-like 4 (Sytl4) and retinol binding protein 4 (Rbp4) served an annealing temperature of 66°C and a measuring temperature of 82°C and 88°C, respectively. Melting curves were determined using the following parameters: 95°C cooling to 50°C; ramping to 99°C at 0.2°C/sec. The following primer pairs were used: "mInhibin beta-2s" GGA GGG CTG GAA GAG GAA AAG GAA, "mmInhibin beta-2as" TAA GGT TGG CAA AGG GGC TGT GAC (product 328 bp), "mmProtocad 8_3734s" GCT AAG TGG AAG TTA CTG CCA AAG, "mmProtocad 8_3974as" AGT GAC AGA GCT TAC AGA GAA CCG (product 240 bp), "mmNrn 1_1098s" TCC ACG TGG GAA TCA GCC GGT G, "mmNrn 1_1352as" TGG ACC TGA ACG AGG GGC ATC G (product 254 bp), "mmSpon 1_3770s" GAG GAT GTT GAT CTC AGC TGT GAG, "mmSpon 1_4016as" TGA ACA GGC TCT CGT GTG TTA AGG (product 246 bp), "mm Calb1-1293s" CTT CTA TCT GGC GGA AGG GAT GG, "mm Calb1-1533as" ACT CTC TGG CAG TAG ACT TCT TGG (product 240 bp), "mm Rerg-1710s" CAG TAA GTT GGC CAC ATG CCT TGT, "mm Rerg-1918as" CCA AAT CTC AGT ATA TGG GGC AGG (product 208 bp), "mm Sytl4-3242s" GTG GAT GGA ATT CCC CAG CCA TTC, "mm Sytl4-3656as" GAA CAA GTC TGA GCC TGG GCT CTC (product 414 bp), "mmRbp4-567s" GGA AGT GTG TGC AGA CAT GGT GGG, "mmRbp4-903as" GGA GGG CCT GCT TTG ACA GTA ACC (product 336 bp), "mmNpy2r-2752s" GTT GCA CTT CTC TGG ATG CTG ACG, "mmNpy2r-3122as" GCT CCT TCA GAA TGA CTG TGC AGA (product 370 bp). Specificity of products was ensured by melting point analysis and agarose gel electrophoresis. cDNA content of samples was normalized to the expression level of Cyclophilin (primers: "cyc5" ACC CCA CCG TGT TCT TCG AC, "acyc300" CAT TTG CCA TGG ACA AGA TG). Relative regulation levels were derived after normalization to cyclophilin.

### Immunohistochemistry

For immunohistochemistry, sections of paraffin-embedded tissues (2 μm) were deparaffinated and microwaved (citrate buffer at 500 W for 10 min). Afterwards, sections were incubated at room temperature with the respective antisera (α-Bok: #4521, Cell Signaling, 1:100; α-Synapsin II: #VAS-SV061, Stressgen, 1:100) over night at 4°C in a humid chamber. Staining was visualized using the ABC technique with DAB as chromogen (DAKO).

## Authors' contributions

DN, GV, OW, OS, AS carried out data analysis and statistical testing, PP, WK, HS, MM, AS designed, established and performed the ischemic model used, DN carried out array hybridizations, CK performed and evaluated immunohistochemical stainings, WK, HH, DN and AS conceived of the study, and were involved in the overall design, AB helped in the discussion of the results, CB, NS carried out laser microdissection, GE, MR performed RNA amplification and probe labeling, GE performed quantitative PCR, AS, DN, WK drafted the manuscript. All authors read and approved the final manuscript.

## Supplementary Material

Additional file 1 **Size distribution of amplified RNA from CA1 and CA3 areas**. A, Shown are electropherograms generated with the Agilent Bioanalyzer from 2^nd ^round amplified RNA from CA1 or CA3 of 4 animals (2 sham, 2 ischemia-treated). B, Marker lane with corresponding sizes (nt, nucleotides), and corresponding curve. C, Shown are the corresponding graphs. There is no difference in size distribution between CA1 and CA3, nor between sham- and ischemia-treated animals. The bulk of the amplified RNA ranges between 200 to 2000 nt. Click here for file

Additional file 2 **List of all significantly regulated genes in CA3 vs CA1 (native state)**. HTML file containing all significantly regulated genes in CA3 vs CA1 in the native state, with Agilent probe numbers, accession numbers, gene names, enrichment factors ("M (CA3/CA1")), and false-discovery-rate corrected p-values. M indicates direction and extent of enrichment: Numbers >1 indicate preferential expression in CA3, numbers <1 in CA1. Click here for file

Additional file 3 **Genes with differential expression in CA3 and CA1 under native conditions: Verification by detection in brain mapping projects**. Shown are genes which had significantly differing distribution to CA3 and CA1. Selected genes with matches in brain mapping projects (BGEM or Allen Brain Atlas) are given with the gene symbol, gene name, Refseq accession number, Agilent probe number, preference of enrichment, and enrichment factor, and location in or link to microgrpahs of in situ hybridizations. All genes that were found are confirmed by the in-situ hybridizations. There is also general good correlation of the enrichment factors and visual impressions of the staining intensities in CA3 or CA1. Click here for file

Additional file 4 **List of all significantly regulated genes in CA3 ischemic vs. sham**. HTML file containing all genes significantly regulated in CA3 between the ischemic and the sham group. Given are Agilent probe numbers, accession numbers, gene names, enrichment factors ("M (ICA3/SCA3")), and false-discovery-rate corrected p-values. Click here for file

Additional file 5 **List of all significantly regulated genes in CA1 ischemic vs. sham**. HTML file containing all genes significantly regulated in CA1 between the ischemic and the sham group. Given are Agilent probe numbers, accession numbers, gene names, enrichment factors ("M (ICA1/SCA1")), and false-discovery-rate corrected p-values. Click here for file

Additional file 6 **List of all significantly regulated genes in CA3 vs CA1 (ischemic state)**. HTML file containing all significantly regulated genes in CA3 vs CA1 in the ischemic state. Given are Agilent probe numbers, accession numbers, gene names, enrichment factors ("M (CA3/CA1")), and false-discovery-rate corrected p-values. Click here for file

Additional file 7 **Comparison of regulation factors derived from array analysis and quantitative PCR**. Shown are 8 genes that show differential expression between CA3 and CA1 in the native or ischemic state with the regulation factor derived from array analysis, and quantitative PCR: There is a good correlation between both assessments. The Pearson correlation coefficient from these 8 comparisons is 0.75. Click here for file

Additional file 8 **Immunohistochemical detection of Synatotagmin-like 4 in the hippocampus**. A, Distribution of Sytl4 gene product in the hippocampus of a sham-operated rat (overview), B, and in an animal from the ischemic group. C,D magnification of the CA3 field of a sham (C), and ischemic (D) animal (original magnification 40×). Sytl4 localizes preferentially to the CA3 field (stratum lucidum) under both conditions. Click here for file

Additional file 9 **Distribution of standard deviations in the ischemic and sham groups**. Plotted are the cumulative number of genes in each group versus the standard deviation. Both distributions are highly similar, although the ischemic group is slightly right-shifted. Click here for file

Additional file 10 **Genes that show a significantly different CA3/CA1 distribution under normoxic and ischemic conditions**. Shown are 15 genes which had significantly differing distribution to CA3 and CA1 in the normoxic versus the ischemic state. Given are the accession number, gene name, the relative CA3/CA1 ratio shift from normoxic to ischemic conditions (negative values indicate the reverse CA1/CA3 ratio), the raw P values and the FDR-corrected P-values for the siginificance of different behaviour under the two conditions), and the CA3/CA1 ratios under normoxic and ischemic conditions with the respective FDR-corrected P-values. Changes in most genes are caused by an attenuation of differential expression by ischemia. The only gene which shows a true reversal of distribution preference is Inhibin beta A (from CA1 in sham to CA3 in ischemia). Click here for file

## References

[B1] Visel A, Thaller C, Eichele G (2004). GenePaint.org: an atlas of gene expression patterns in the mouse embryo. Nucleic Acids Res.

[B2] Carson JP, Ju T, Lu HC, Thaller C, Xu M, Pallas SL, Crair MC, Warren J, Chiu W, Eichele G (2005). A digital atlas to characterize the mouse brain transcriptome. PLoS Comput Biol.

[B3] Gray PA, Fu H, Luo P, Zhao Q, Yu J, Ferrari A, Tenzen T, Yuk DI, Tsung EF, Cai Z, Alberta JA, Cheng LP, Liu Y, Stenman JM, Valerius MT, Billings N, Kim HA, Greenberg ME, McMahon AP, Rowitch DH, Stiles CD, Ma Q (2004). Mouse brain organization revealed through direct genome-scale TF expression analysis. Science.

[B4] Magdaleno S, Jensen P, Brumwell CL, Seal A, Lehman K, Asbury A, Cheung T, Cornelius T, Batten DM, Eden C, Norland SM, Rice DS, Dosooye N, Shakya S, Mehta P, Curran T (2006). BGEM: an in situ hybridization database of gene expression in the embryonic and adult mouse nervous system. PLoS Biol.

[B5] Gong S, Zheng C, Doughty ML, Losos K, Didkovsky N, Schambra UB, Nowak NJ, Joyner A, Leblanc G, Hatten ME, Heintz N (2003). A gene expression atlas of the central nervous system based on bacterial artificial chromosomes. Nature.

[B6] Bohm C, Newrzella D, Sorgenfrei O (2005). Laser microdissection in CNS research. Drug Discov Today.

[B7] Rossner MJ, Hirrlinger J, Wichert SP, Boehm C, Newrzella D, Hiemisch H, Eisenhardt G, Stuenkel C, von Ahsen O, Nave KA (2006). Global transcriptome analysis of genetically identified neurons in the adult cortex. J Neurosci.

[B8] Yang G, Kitagawa K, Ohtsuki T, Kuwabara K, Mabuchi T, Yagita Y, Takazawa K, Tanaka S, Yanagihara T, Hori M, Matsumoto M (2000). Regional difference of neuronal vulnerability in the murine hippocampus after transient forebrain ischemia. Brain Res.

[B9] Wilde GJ, Pringle AK, Wright P, Iannotti F (1997). Differential vulnerability of the CA1 and CA3 subfields of the hippocampus to superoxide and hydroxyl radicals in vitro. J Neurochem.

[B10] Davolio C, Greenamyre JT (1995). Selective vulnerability of the CA1 region of hippocampus to the indirect excitotoxic effects of malonic acid. Neurosci Lett.

[B11] Ordy JM, Wengenack TM, Bialobok P, Coleman PD, Rodier P, Baggs RB, Dunlap WP, Kates B (1993). Selective vulnerability and early progression of hippocampal CA1 pyramidal cell degeneration and GFAP-positive astrocyte reactivity in the rat four-vessel occlusion model of transient global ischemia. Exp Neurol.

[B12] Vannucci SJ, Seaman LB, Vannucci RC (1996). Effects of hypoxia-ischemia on GLUT1 and GLUT3 glucose transporters in immature rat brain. J Cereb Blood Flow Metab.

[B13] Vannucci SJ, Willing LB, Goto S, Alkayed NJ, Brucklacher RM, Wood TL, Towfighi J, Hurn PD, Simpson IA (2001). Experimental stroke in the female diabetic, db/db, mouse. J Cereb Blood Flow Metab.

[B14] http://www.ebi.ac.uk/microarray-as/aer/result?queryFor=Experiment&eAccession=E-MEXP-1253.

[B15] Tsukita S, Furuse M (1998). Overcoming barriers in the study of tight junction functions: from occludin to claudin. Genes Cells.

[B16] Inagaki M, Irie K, Deguchi-Tawarada M, Ikeda W, Ohtsuka T, Takeuchi M, Takai Y (2003). Nectin-dependent localization of ZO-1 at puncta adhaerentia junctions between the mossy fiber terminals and the dendrites of the pyramidal cells in the CA3 area of adult mouse hippocampus. J Comp Neurol.

[B17] Hsu SY, Kaipia A, McGee E, Lomeli M, Hsueh AJ (1997). Bok is a pro-apoptotic Bcl-2 protein with restricted expression in reproductive tissues and heterodimerizes with selective anti-apoptotic Bcl-2 family members. Proc Natl Acad Sci USA.

[B18] Yakovlev AG, Di Giovanni S, Wang G, Liu W, Stoica B, Faden AI (2004). BOK and NOXA are essential mediators of p53-dependent apoptosis. J Biol Chem.

[B19] Rodriguez JM, Glozak MA, Ma Y, Cress WD (2006). Bok, Bcl-2 related ovarian killer, is cell cycle regulated and sensitizes to stress-induced apoptosis. J Biol Chem.

[B20] Reymond N, Borg JP, Lecocq E, Adelaide J, Campadelli-Fiume G, Dubreuil P, Lopez M (2000). Human nectin3/PRR3: a novel member of the PVR/PRR/nectin family that interacts with afadin. Gene.

[B21] Takahashi K, Nakanishi H, Miyahara M, Mandai K, Satoh K, Satoh A, Nishioka H, Aoki J, Nomoto A, Mizoguchi A, Takai Y (1999). Nectin/PRR: an immunoglobulin-like cell adhesion molecule recruited to cadherin-based adherens junctions through interaction with Afadin, a PDZ domain-containing protein. J Cell Biol.

[B22] Honda T, Sakisaka T, Yamada T, Kumazawa N, Hoshino T, Kajita M, Kayahara T, Ishizaki H, Tanaka-Okamoto M, Mizoguchi A, Manabe T, Miyoshi J, Takai Y (2006). Involvement of nectins in the formation of puncta adherentia junctions and the mossy fiber trajectory in the mouse hippocampus. Mol Cell Neurosci.

[B23] Yamada A, Irie K, Deguchi-Tawarada M, Ohtsuka T, Takai Y (2003). Nectin-dependent localization of synaptic scaffolding molecule (S-SCAM) at the puncta adherentia junctions formed between the mossy fibre terminals and the dendrites of pyramidal cells in the CA3 area of the mouse hippocampus. Genes Cells.

[B24] Finlin BS, Gau CL, Murphy GA, Shao H, Kimel T, Seitz RS, Chiu YF, Botstein D, Brown PO, Der CJ, Tamanoi F, Andres DA, Perou CM (2001). RERG is a novel ras-related, estrogen-regulated and growth-inhibitory gene in breast cancer. J Biol Chem.

[B25] Key MD, Andres DA, Der CJ, Repasky GA (2005). Characterization of RERG: An Estrogen-Regulated Tumor Suppressor Gene. Methods Enzymol.

[B26] Gnidehou S, Caillou B, Talbot M, Ohayon R, Kaniewski J, Noel-Hudson MS, Morand S, Agnangji D, Sezan A, Courtin F, Virion A, Dupuy C (2004). Iodotyrosine dehalogenase 1 (DEHAL1) is a transmembrane protein involved in the recycling of iodide close to the thyroglobulin iodination site. Faseb J.

[B27] Sudhof TC (2001). alpha-Latrotoxin and its receptors: neurexins and CIRL/latrophilins. Annu Rev Neurosci.

[B28] Tomsig JL, Creutz CE (2002). Copines: a ubiquitous family of Ca(2+)-dependent phospholipid-binding proteins. Cell Mol Life Sci.

[B29] Newman JC, Weiner AM (2005). L2L: a simple tool for discovering the hidden significance in microarray expression data. Genome Biol.

[B30] Cinelli P, Madani R, Tsuzuki N, Vallet P, Arras M, Zhao CN, Osterwalder T, Rulicke T, Sonderegger P (2001). Neuroserpin, a neuroprotective factor in focal ischemic stroke. Mol Cell Neurosci.

[B31] Weaver M, Leshley K, Sands H, Gritman KR, Legos JJ, Tuma RF (2002). LEX032, a novel recombinant serpin, protects the brain after transient focal ischemia. Microvasc Res.

[B32] Carter TA, Greenhall JA, Yoshida S, Fuchs S, Helton R, Swaroop A, Lockhart DJ, Barlow C (2005). Mechanisms of aging in senescence-accelerated mice. Genome Biol.

[B33] Harris AL (2002). Hypoxia–a key regulatory factor in tumour growth. Nat Rev Cancer.

[B34] Lein ES, Hawrylycz MJ, Ao N, Ayres M, Bensinger A, Bernard A, Boe AF, Boguski MS, Brockway KS, Byrnes EJ, Chen L, Chen L, Chen TM, Chin MC, Chong J, Crook BE, Czaplinska A, Dang CN, Datta S, Dee NR, Desaki AL, Desta T, Diep E, Dolbeare TA, Donelan MJ, Dong HW, Dougherty JG, Duncan BJ, Ebbert AJ, Eichele G (2007). Genome-wide atlas of gene expression in the adult mouse brain. Nature.

[B35] Lein ES, Zhao X, Gage FH (2004). Defining a molecular atlas of the hippocampus using DNA microarrays and high-throughput in situ hybridization. J Neurosci.

[B36] Nelson RE, Fessler LI, Takagi Y, Blumberg B, Keene DR, Olson PF, Parker CG, Fessler JH (1994). Peroxidasin: a novel enzyme-matrix protein of Drosophila development. Embo J.

[B37] Wei C, Li J, Bumgarner RE (2004). Sample size for detecting differentially expressed genes in microarray experiments. BMC genomics.

[B38] Lason W, Przewlocka B, Van Luijtelaar G, Coenen A (1994). Proenkephalin and prodynorphin mRNA level in brain of rats with absence epilepsy. Neuropeptides.

[B39] Noh HS, Kim DW, Kang SS, Kim YH, Cho GJ, Choi WS (2006). Ketogenic diet decreases the level of proenkephalin mRNA induced by kainic acid in the mouse hippocampus. Neurosci Lett.

[B40] Przewlocka B, Lason W, Machelska H, Przewlocki R (1994). The effects of cocaine-induced seizures on the proenkephalin mRNA level in the mouse hippocampus: a possible involvement of the nitric oxide pathway. Neurosci Lett.

[B41] Bottner M, Dubal DB, Rau SW, Suzuki S, Wise PM (2006). Stroke injury in rats causes an increase in activin A gene expression which is unaffected by oestradiol treatment. J Neuroendocrinol.

[B42] Soriano MA, Tessier M, Certa U, Gill R (2000). Parallel gene expression monitoring using oligonucleotide probe arrays of multiple transcripts with an animal model of focal ischemia. J Cereb Blood Flow Metab.

[B43] Lai M, Sirimanne E, Williams CE, Gluckman PD (1996). Sequential patterns of inhibin subunit gene expression following hypoxic-ischemic injury in the rat brain. Neuroscience.

[B44] Tretter YP, Hertel M, Munz B, ten Bruggencate G, Werner S, Alzheimer C (2000). Induction of activin A is essential for the neuroprotective action of basic fibroblast growth factor in vivo. Nat Med.

[B45] Bohm C, Newrzella D, Herberger S, Schramm N, Eisenhardt G, Schenk V, Sonntag-Buck V, Sorgenfrei O (2006). Effects of antidepressant treatment on gene expression profile in mouse brain: cell type-specific transcription profiling using laser microdissection and microarray analysis. J Neurochem.

[B46] Brambrink AM, Schneider A, Noga H, Astheimer A, Gotz B, Korner I, Heimann A, Welschof M, Kempski O (2000). Tolerance-Inducing dose of 3-nitropropionic acid modulates bcl-2 and bax balance in the rat brain: a potential mechanism of chemical preconditioning. J Cereb Blood Flow Metab.

[B47] Bonaventure P, Guo H, Tian B, Liu X, Bittner A, Roland B, Salunga R, Ma XJ, Kamme F, Meurers B, Bakker M, Jurzak M, Leysen JE, Erlander MG (2002). Nuclei and subnuclei gene expression profiling in mammalian brain. Brain Res.

[B48] Robles Y, Vivas-Mejia PE, Ortiz-Zuazaga HG, Felix J, Ramos X, Pena de Ortiz S (2003). Hippocampal gene expression profiling in spatial discrimination learning. Neurobiol Learn Mem.

[B49] Torres-Munoz JE, Van Waveren C, Keegan MG, Bookman RJ, Petito CK (2004). Gene expression profiles in microdissected neurons from human hippocampal subregions. Brain Res Mol Brain Res.

[B50] Ginsberg SD, Che S (2005). Expression profile analysis within the human hippocampus: comparison of CA1 and CA3 pyramidal neurons. J Comp Neurol.

[B51] Datson NA, Meijer L, Steenbergen PJ, Morsink MC, van der Laan S, Meijer OC, de Kloet ER (2004). Expression profiling in laser-microdissected hippocampal subregions in rat brain reveals large subregion-specific differences in expression. Eur J Neurosci.

[B52] Zhao X, Lein ES, He A, Smith SC, Aston C, Gage FH (2001). Transcriptional profiling reveals strict boundaries between hippocampal subregions. J Comp Neurol.

[B53] Jung BP, Zhang G, Ho W, Francis J, Eubanks JH (2002). Transient forebrain ischemia alters the mRNA expression of methyl DNA-binding factors in the adult rat hippocampus. Neuroscience.

[B54] Jin K, Graham SH, Nagayama T, Goldsmith PC, Greenberg DA, Zhou A, Simon RP (2001). Altered expression of the neuropeptide-processing enzyme carboxypeptidase E in the rat brain after global ischemia. J Cereb Blood Flow Metab.

[B55] Sun HB, Yokota H, Chi XX, Xu ZC (2000). Differential expression of neurexin mRNA in CA1 and CA3 hippocampal neurons in response to ischemic insult. Brain Res Mol Brain Res.

[B56] Timsit S, Rivera S, Ouaghi P, Guischard F, Tremblay E, Ben-Ari Y, Khrestchatisky M (1999). Increased cyclin D1 in vulnerable neurons in the hippocampus after ischaemia and epilepsy: a modulator of in vivo programmed cell death?. Eur J Neurosci.

[B57] Francis J, Zhang Y, Ho W, Wallace MC, Zhang L, Eubanks JH (1999). Decreased hippocampal expression, but not functionality, of GABA(B) receptors after transient cerebral ischemia in rats. J Neurochem.

[B58] Chen J, Uchimura K, Stetler RA, Zhu RL, Nakayama M, Jin K, Graham SH, Simon RP (1998). Transient global ischemia triggers expression of the DNA damage-inducible gene GADD45 in the rat brain. J Cereb Blood Flow Metab.

[B59] Hsu JC, Zhang L, Wallace MC, Eubanks JH (1996). Cerebral ischemia alters the regional hippocampal expression of the rat m1 muscarinic acetylcholine receptor gene. Neurosci Lett.

[B60] Chen J, Zhu RL, Nakayama M, Kawaguchi K, Jin K, Stetler RA, Simon RP, Graham SH (1996). Expression of the apoptosis-effector gene, Bax, is up-regulated in vulnerable hippocampal CA1 neurons following global ischemia. J Neurochem.

[B61] Tortosa A, Ferrer I (1993). Parvalbumin immunoreactivity in the hippocampus of the gerbil after transient forebrain ischaemia: a qualitative and quantitative sequential study. Neuroscience.

[B62] Gass P, Muelhardt C, Sommer C, Becker CM, Kiessling M (1993). NMDA and glycine receptor mRNA expression following transient global ischemia in the gerbil brain. J Cereb Blood Flow Metab.

[B63] Van Gelder RN, von Zastrow ME, Yool A, Dement WC, Barchas JD, Eberwine JH (1990). Amplified RNA synthesized from limited quantities of heterogeneous cDNA. Proc Natl Acad Sci USA.

[B64] http://www.bioconductor.org.

[B65] Benjamini Y, Hochberg Y (1995). Controlling the false discovery rate – a practical and powerful approach to multiple testing. J Roy Stat Soc B Met.

