# Supplementary figures and images for: The functional genome of CA1 and CA3 neurons under native conditions and in response to ischemia
Source: BMC Genomics. 2007 Oct 15;8:370. doi: 10.1186/1471-2164-8-370 (PMC2194787; doi:10.1186/1471-2164-8-370)

**A**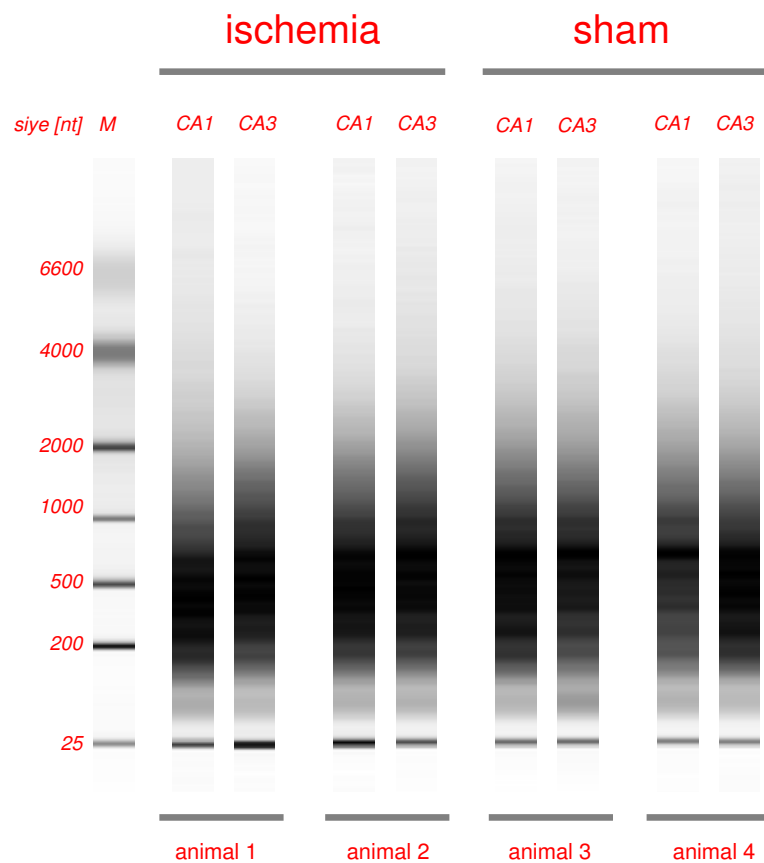**B**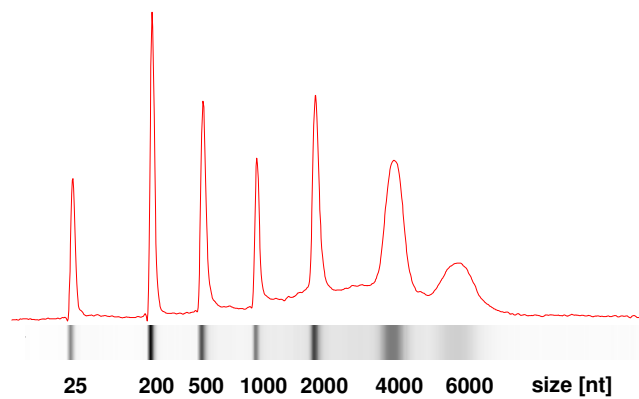**C**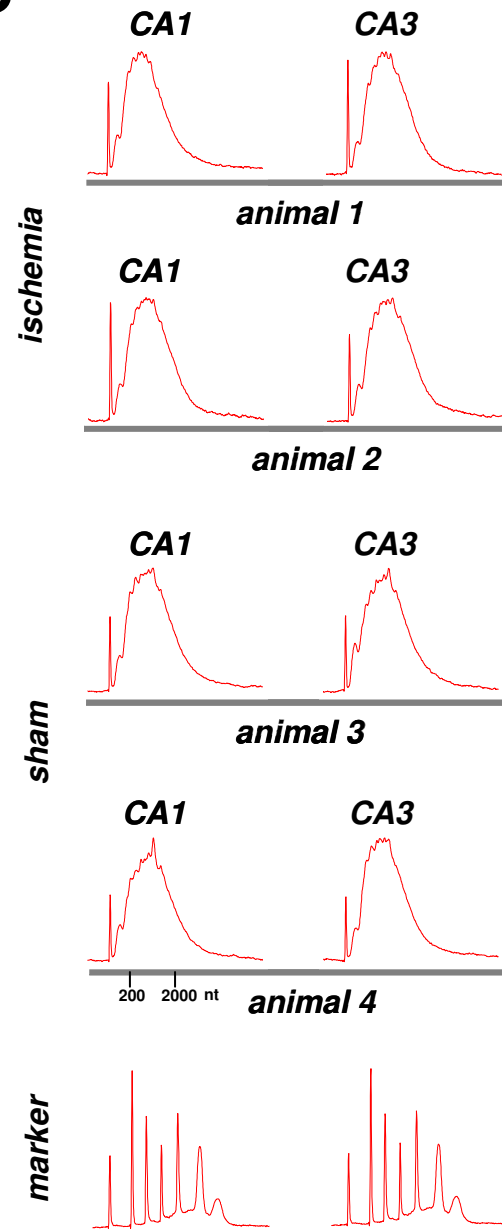

Supplement: Additional file 1 — Size distribution of amplified RNA from CA1 and CA3 areas. A, Shown are electropherograms generated with the Agilent Bioanalyzer from 2nd round amplified RNA from CA1 or CA3 of 4 animals (2 sham, 2 ischemia-treated). B, Marker lane with corresponding sizes (nt, nucleotides), and corresponding curve. C, Shown are the corresponding graphs. There is no difference in size distribution between CA1 and CA3, nor between sham- and ischemia-treated animals. The bulk of the amplified RNA ranges between 200 to 2000 nt. [file 1471-2164-8-370-S1.pdf]

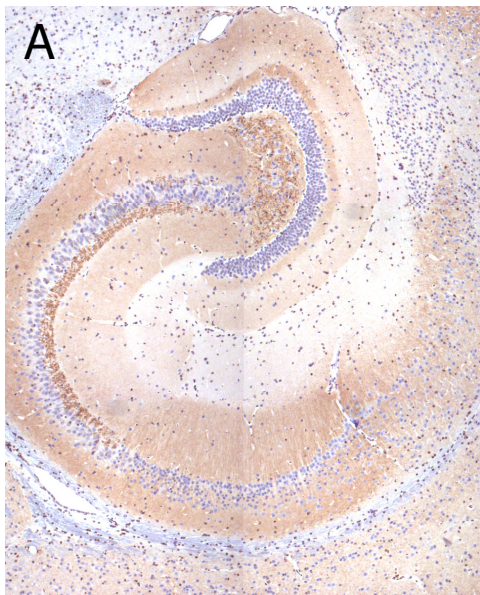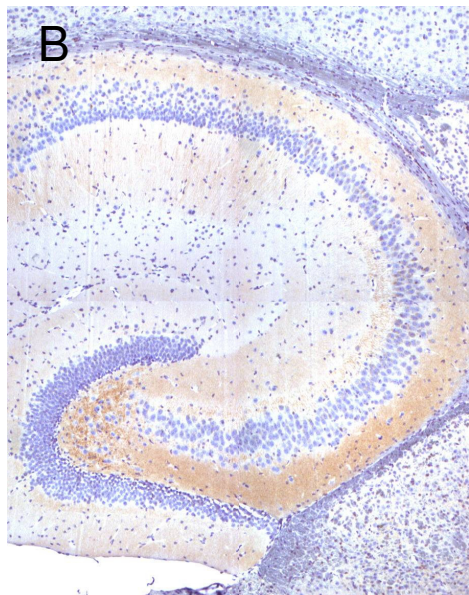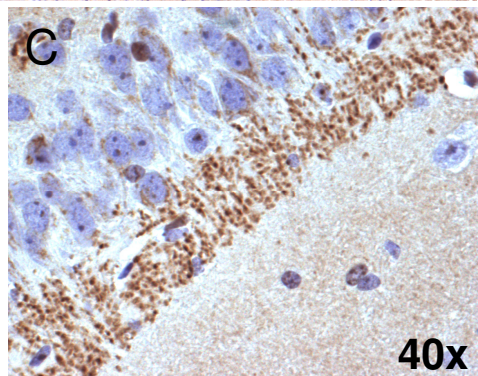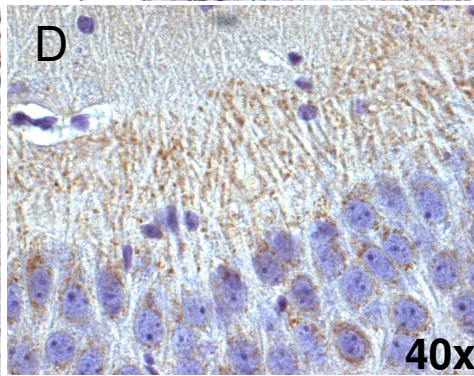

Supplement: Additional file 8 — Immunohistochemical detection of Synatotagmin-like 4 in the hippocampus. A, Distribution of Sytl4 gene product in the hippocampus of a sham-operated rat (overview), B, and in an animal from the ischemic group. C,D magnification of the CA3 field of a sham (C), and ischemic (D) animal (original magnification 40×). Sytl4 localizes preferentially to the CA3 field (stratum lucidum) under both conditions. [file 1471-2164-8-370-S8.pdf]

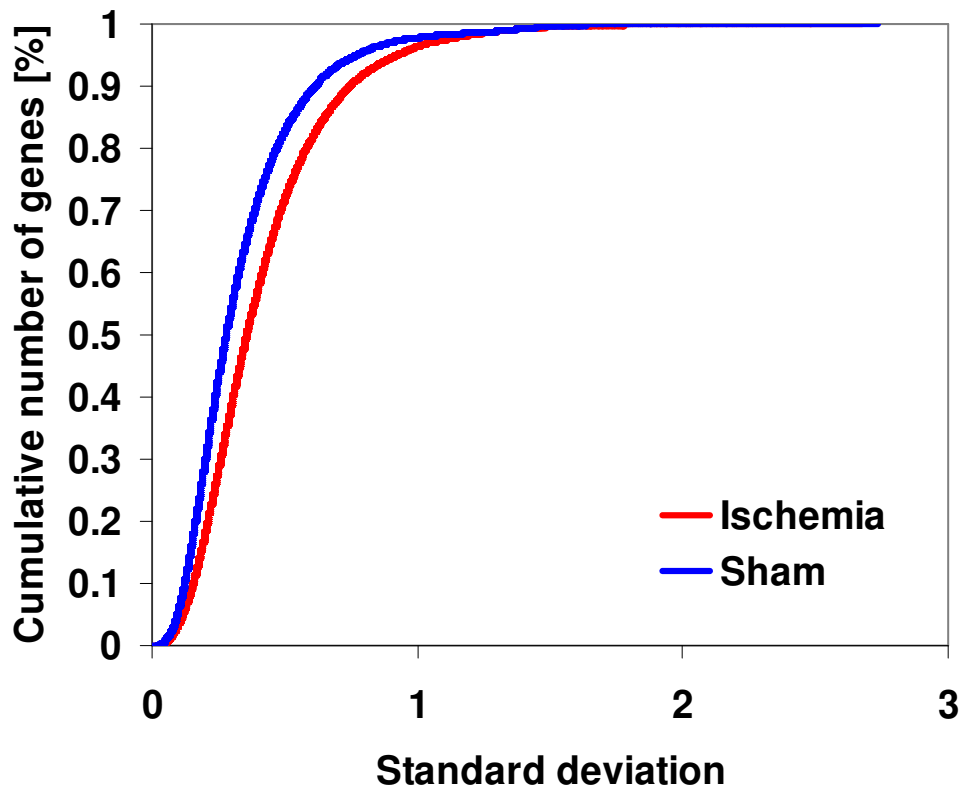

Supplement: Additional file 9 — Distribution of standard deviations in the ischemic and sham groups. Plotted are the cumulative number of genes in each group versus the standard deviation. Both distributions are highly similar, although the ischemic group is slightly right-shifted. [file 1471-2164-8-370-S9.pdf]
