# Supplementary material for: The functional genome of CA1 and CA3 neurons under native conditions and in response to ischemia
Source: BMC Genomics. 2007 Oct 15;8:370. doi: 10.1186/1471-2164-8-370 (PMC2194787; doi:10.1186/1471-2164-8-370)
Supplement: Additional file 2 — List of all significantly regulated genes in CA3 vs CA1 (native state). HTML file containing all significantly regulated genes in CA3 vs CA1 in the native state, with Agilent probe numbers, accession numbers, gene names, enrichment factors ("M (CA3/CA1")), and false-discovery-rate corrected p-values. M indicates direction and extent of enrichment: Numbers >1 indicate preferential expression in CA3, numbers <1 in CA1. [file 1471-2164-8-370-S2.htm]

| Agilent probe# | Accession# | gene name | M (CA3/CA1) | P.fdr |  |  |
| A\_51\_P132530 | AK008821 | Mus musculus adult male stomach cDNA, RIKEN full-length enriched library, clone:2210404A22 product:hypothetical PMP-22/ | 69.40 | 0.00000 |  | |
| A\_51\_P373082 | NM\_016778 | Mus musculus Bcl-2-related ovarian killer protein (Bok), mRNA | 33.39 | 0.00001 |  | |
| A\_51\_P125009 | NM\_021495 | Mus musculus poliovirus receptor-related 3 (Pvrl3), transcript variant alpha, mRNA | 22.39 | 0.00000 |  | |
| A\_51\_P305508 | NM\_181988 | Mus musculus RAS-like, estrogen-regulated, growth-inhibitor (Rerg), mRNA | 21.49 | 0.00002 |  | |
| A\_51\_P411200 | NM\_027391 | Mus musculus RIKEN cDNA 0610009A07 gene (0610009A07Rik), mRNA | 21.28 | 0.00000 |  | |
| A\_51\_P147766 | NM\_175079 | Mus musculus RIKEN cDNA A730016F12 gene (A730016F12Rik), transcript variant 2, mRNA | 18.94 | 0.00006 |  | |
| A\_51\_P340699 | XM\_485698 | PREDICTED: RAS-like family 11 member A | 16.05 | 0.00009 |  | |
| A\_51\_P105157 | NM\_028719 | Mus musculus copine IV (Cpne4), mRNA | 15.73 | 0.00196 |  | |
| A\_51\_P423465 | NM\_153098 | Mus musculus CD109 antigen (Cd109), mRNA | 13.49 | 0.00004 |  | |
| A\_51\_P191669 | NM\_007694 | Mus musculus chromogranin B (Chgb), mRNA | 11.11 | 0.00007 |  | |
| A\_51\_P513032 | AK046585 | Mus musculus 4 days neonate male adipose cDNA, RIKEN full-length enriched library, clone:B430110C06 product:hypothetical | 10.66 | 0.00020 |  | |
| A\_51\_P291713 | NM\_134094 | Mus musculus neurocalcin delta (Ncald), mRNA | 10.09 | 0.00042 |  | |
| A\_51\_P237856 | NM\_008328 | Mus musculus interferon activated gene 203 (Ifi203), mRNA | 9.29 | 0.00001 |  | |
| A\_51\_P260288 | AK016705 | Mus musculus adult male testis cDNA, RIKEN full-length enriched library, clone:4933406O10 product:RIKEN cDNA 4933406O | 8.52 | 0.00051 |  | |
| A\_51\_P320454 | NM\_146241 | Mus musculus TRH-degrading enzyme (Trhde), mRNA | 8.29 | 0.00016 |  | |
| A\_51\_P173114 | NM\_130878 | Mus musculus protocadherin 21 (Pcdh21), mRNA | 8.21 | 0.00007 |  | |
| A\_51\_P142972 | NM\_029947 | Mus musculus PR domain containing 8 (Prdm8), mRNA | 7.81 | 0.00496 |  | |
| A\_51\_P267986 | NM\_145951 | Mus musculus cytosolic ovarian carcinoma antigen 1 (Cova1), mRNA | 7.40 | 0.00003 |  | |
| A\_51\_P224164 | NM\_011867 | Mus musculus solute carrier family 26, member 4 (Slc26a4), mRNA | 6.81 | 0.00002 |  | |
| A\_51\_P384993 | NM\_175481 | Mus musculus glutamate receptor, ionotropic, kainate 4 (Grik4), mRNA | 6.79 | 0.00063 |  | |
| A\_51\_P376656 | NM\_028052 | Mus musculus synaptoporin (Synpr), mRNA | 6.46 | 0.00130 |  | |
| A\_51\_P165507 | NM\_007855 | Mus musculus twist homolog 2 (Drosophila) (Twist2), mRNA | 6.14 | 0.00003 |  | |
| A\_51\_P487298 | NM\_139307 | Mus musculus Slit-like 2 (Drosophila) (Slitl2), mRNA | 5.88 | 0.00016 |  | |
| A\_51\_P311919 | NM\_008731 | Mus musculus neuropeptide Y receptor Y2 (Npy2r), mRNA | 5.78 | 0.00064 |  | |
| A\_51\_P358485 | NM\_173447 | Mus musculus Eph receptor B1 (Ephb1), mRNA | 5.59 | 0.00305 |  | |
| A\_51\_P254855 | NM\_011198 | Mus musculus prostaglandin-endoperoxide synthase 2 (Ptgs2), mRNA | 5.54 | 0.00229 |  | |
| A\_51\_P496569 | NM\_178804 | Mus musculus slit homolog 2 (Drosophila) (Slit2), mRNA | 5.46 | 0.00265 |  | |
| A\_51\_P199580 | NM\_028185 | Mus musculus U7 snRNP-specific Sm-like protein LSM11 (Lsm11), mRNA | 5.19 | 0.00036 |  | |
| A\_51\_P171613 | NM\_009518 | Mus musculus wingless related MMTV integration site 10a (Wnt10a), mRNA | 5.05 | 0.00001 |  | |
| A\_51\_P349546 | NM\_153098 | Mus musculus CD109 antigen (Cd109), mRNA | 4.98 | 0.00004 |  | |
| A\_51\_P308844 | NM\_153529 | Mus musculus neuritin 1 (Nrn1), mRNA | 4.81 | 0.00010 |  | |
| A\_51\_P155190 | NM\_010231 | Mus musculus flavin containing monooxygenase 1 (Fmo1), mRNA | 4.55 | 0.02008 |  | |
| A\_51\_P407025 | NM\_007607 | Mus musculus carbonic anhydrase 4 (Car4), mRNA | 4.46 | 0.00007 |  | |
| A\_51\_P408653 | NM\_145463 | Mus musculus transmembrane protein 46 (Tmem46), mRNA | 4.37 | 0.00829 |  | |
| A\_51\_P276943 | NM\_016696 | Mus musculus glypican 1 (Gpc1), mRNA | 4.35 | 0.00060 |  | |
| A\_51\_P347312 | BC019747 | Mus musculus opioid growth factor receptor-like 1, mRNA (cDNA clone MGC:30347 IMAGE:4485439), complete cds. | 4.28 | 0.00018 |  | |
| A\_51\_P370423 | NM\_022319 | Mus musculus calsyntenin 2 (Clstn2), mRNA | 4.22 | 0.00004 |  | |
| A\_51\_P337230 | NM\_027864 | Mus musculus UDP-N-acetyl-alpha-D-galactosamine:polypeptide N-acetylgalactosaminyltransferase 14 (Galnt14), mRNA | 4.21 | 0.00048 |  | |
| A\_51\_P101460 | AK077574 | Mus musculus 8 days embryo whole body cDNA, RIKEN full-length enriched library, clone:5730453H04 product:desmoplakin, d | 4.21 | 0.01998 |  | |
| A\_51\_P318580 | NM\_028021 | Mus musculus myosin, heavy polypeptide 14 (Myh14), mRNA | 4.11 | 0.00006 |  | |
| A\_51\_P414078 | NM\_001025246 | Mus musculus Trp53 inducible protein 11 (Trp53i11), mRNA | 4.02 | 0.00094 |  | |
| A\_51\_P137079 | NM\_028841 | Mus musculus tetraspanin 17 (Tspan17), mRNA | 4.00 | 0.00051 |  | |
| A\_51\_P144601 | NM\_017379 | Mus musculus tubulin, alpha 8 (Tuba8), mRNA | 3.92 | 0.00019 |  | |
| A\_51\_P171200 | NM\_027307 | Mus musculus golgi phosphoprotein 2 (Golph2), mRNA | 3.81 | 0.00004 |  | |
| A\_51\_P303919 | NM\_019754 | Mus musculus transgelin 3 (Tagln3), mRNA | 3.80 | 0.00031 |  | |
| A\_51\_P212491 | NM\_133232 | Mus musculus 6-phosphofructo-2-kinase/fructose-2,6-biphosphatase 3 (Pfkfb3), transcript variant 1, mRNA | 3.78 | 0.00027 |  | |
| A\_51\_P206144 | NM\_133733 | Mus musculus RIKEN cDNA 9030425E11 gene (9030425E11Rik), mRNA | 3.75 | 0.00037 |  | |
| A\_51\_P460734 | NM\_011103 | Mus musculus protein kinase C, delta (Prkcd), mRNA | 3.75 | 0.01413 |  | |
| A\_51\_P487360 | NM\_016677 | Mus musculus hippocalcin-like 1 (Hpcal1), mRNA | 3.71 | 0.00002 |  | |
| A\_51\_P488819 | AK016591 | Mus musculus adult male testis cDNA, RIKEN full-length enriched library, clone:4933400F03 product:hypothetical Arginine-rich | 3.69 | 0.00139 |  | |
| A\_51\_P379552 | XM\_132047 | PREDICTED: RIKEN cDNA B830017A01 gene | 3.65 | 0.00051 |  | |
| A\_51\_P433228 | NM\_009829 | Mus musculus cyclin D2 (Ccnd2), mRNA | 3.63 | 0.00505 |  | |
| A\_51\_P335702 | NM\_029070 | Mus musculus RIKEN cDNA 4930511J11 gene (4930511J11Rik), mRNA | 3.63 | 0.00002 |  | |
| A\_51\_P234404 | NM\_013769 | Mus musculus tight junction protein 3 (Tjp3), mRNA | 3.62 | 0.00007 |  | |
| A\_51\_P436596 | NM\_011286 | Mus musculus rabphilin 3A (Rph3a), mRNA | 3.61 | 0.00713 |  | |
| A\_51\_P316311 | AK173053 | Mus musculus mRNA for mKIAA0897 protein | 3.61 | 0.00184 |  | |
| A\_51\_P408071 | XM\_132322 | PREDICTED: similar to mKIAA0166 protein | 3.59 | 0.00001 |  | |
| A\_51\_P417998 | NM\_013757 | Mus musculus synaptotagmin-like 4 (Sytl4), mRNA | 3.57 | 0.01714 |  | |
| A\_51\_P129694 | NM\_172492 | Mus musculus expressed sequence AI841794 (AI841794), mRNA | 3.52 | 0.00094 |  | |
| A\_51\_P199168 | NM\_007702 | Mus musculus cell death-inducing DNA fragmentation factor, alpha subunit-like effector A (Cidea), mRNA | 3.51 | 0.00100 |  | |
| A\_51\_P152918 | NM\_009472 | Mus musculus unc-5 homolog C (C. elegans) (Unc5c), mRNA | 3.48 | 0.00043 |  | |
| A\_51\_P517075 | NM\_011340 | Mus musculus serine (or cysteine) proteinase inhibitor, clade F, member 1 (Serpinf1), mRNA | 3.41 | 0.00692 |  | |
| A\_51\_P444437 | NM\_025968 | Mus musculus leukotriene B4 12-hydroxydehydrogenase (Ltb4dh), mRNA | 3.40 | 0.00345 |  | |
| A\_51\_P481494 | NM\_011248 | Mus musculus roundabout homolog 3 (Drosophila) (Robo3), mRNA | 3.39 | 0.00029 |  | |
| A\_51\_P477121 | NM\_021451 | Mus musculus phorbol-12-myristate-13-acetate-induced protein 1 (Pmaip1), mRNA | 3.39 | 0.00114 |  | |
| A\_51\_P360615 | NM\_008730 | Mus musculus neuronal pentraxin 1 (Nptx1), mRNA | 3.38 | 0.00030 |  | |
| A\_51\_P467869 | NM\_007389 | Mus musculus cholinergic receptor, nicotinic, alpha polypeptide 1 (muscle) (Chrna1), mRNA | 3.35 | 0.00011 |  | |
| A\_51\_P306527 | NM\_011738 | Mus musculus tyrosine 3-monooxygenase/tryptophan 5-monooxygenase activation protein, eta polypeptide (Ywhah), mRNA | 3.35 | 0.00406 |  | |
| A\_51\_P376238 | NM\_009776 | Mus musculus serine (or cysteine) proteinase inhibitor, clade G, member 1 (Serping1), mRNA | 3.29 | 0.00204 |  | |
| A\_51\_P130773 | NM\_009613 | Mus musculus a disintegrin and metalloprotease domain 11 (Adam11), mRNA | 3.28 | 0.00367 |  | |
| A\_51\_P372702 | NM\_010551 | Mus musculus interleukin 16 (Il16), mRNA | 3.22 | 0.00755 |  | |
| A\_51\_P258848 | AK016951 | Mus musculus adult male testis cDNA, RIKEN full-length enriched library, clone:4933427G17 product:hypothetical protein, full i | 3.21 | 0.00051 |  | |
| A\_51\_P269103 | NM\_021546 | Mus musculus amyloid beta (A4) precursor protein-binding, family A, member 1 binding protein (Apba2bp), mRNA | 3.17 | 0.00505 |  | |
| A\_51\_P462658 | NM\_008431 | Mus musculus potassium channel, subfamily K, member 4 (Kcnk4), mRNA | 3.17 | 0.00003 |  | |
| A\_51\_P384318 | NM\_023143 | Mus musculus complement component 1, r subcomponent (C1r), mRNA | 3.14 | 0.03993 |  | |
| A\_51\_P229613 | NM\_008792 | Mus musculus proprotein convertase subtilisin/kexin type 2 (Pcsk2), mRNA | 3.13 | 0.00674 |  | |
| A\_51\_P137236 | NM\_019498 | Mus musculus olfactomedin 1 (Olfm1), mRNA | 3.11 | 0.00350 |  | |
| A\_51\_P517198 | NM\_009959 | Mus musculus protocadherin alpha 5 (Pcdha5), mRNA | 3.08 | 0.00004 |  | |
| A\_51\_P159962 | NM\_010141 | Mus musculus Eph receptor A7 (Epha7), mRNA | 3.08 | 0.01487 |  | |
| A\_51\_P230904 | NM\_019877 | Mus musculus coatomer protein complex, subunit zeta 2 (Copz2), mRNA | 3.08 | 0.00614 |  | |
| A\_51\_P500656 | NM\_183204 | Mus musculus ring finger protein 182 (Rnf182), mRNA | 3.06 | 0.03608 |  | |
| A\_51\_P134045 | NM\_008792 | Mus musculus proprotein convertase subtilisin/kexin type 2 (Pcsk2), mRNA | 3.05 | 0.00457 |  | |
| A\_51\_P436669 | NM\_011995 | Mus musculus piccolo (presynaptic cytomatrix protein) (Pclo), mRNA | 3.03 | 0.02267 |  | |
| A\_51\_P267634 | NM\_178751 | Mus musculus RIKEN cDNA A730041O15 gene (A730041O15Rik), mRNA | 3.00 | 0.00369 |  | |
| A\_51\_P126437 | NM\_007930 | Mus musculus ectodermal-neural cortex 1 (Enc1), mRNA | 2.98 | 0.02291 |  | |
| A\_51\_P103901 | NM\_058212 | Mus musculus D4, zinc and double PHD fingers, family 3 (Dpf3), mRNA | 2.98 | 0.02270 |  | |
| A\_51\_P213691 | NM\_011324 | Mus musculus sodium channel, nonvoltage-gated, type I, alpha (Scnn1a), mRNA | 2.97 | 0.00001 |  | |
| A\_51\_P520879 | NM\_012059 | Mus musculus SH3 domain protein D19 (Sh3d19), mRNA | 2.97 | 0.00385 |  | |
| A\_51\_P142421 | NM\_138683 | Mus musculus thrombospondin type 1 domain containing gene (Rspondin), mRNA | 2.97 | 0.00119 |  | |
| A\_51\_P346488 | BC085129 | Mus musculus synapsin II, mRNA (cDNA clone MGC:113746 IMAGE:5701861), complete cds. | 2.95 | 0.00018 |  | |
| A\_51\_P280597 | NM\_013469 | Mus musculus annexin A11 (Anxa11), mRNA | 2.95 | 0.01624 |  | |
| A\_51\_P476489 | BC049364 | Mus musculus hypothetical LOC433022, mRNA (cDNA clone IMAGE:6490701) | 2.94 | 0.00060 |  | |
| A\_51\_P394190 | NM\_010723 | Mus musculus LIM domain only 4 (Lmo4), mRNA | 2.93 | 0.00407 |  | |
| A\_51\_P274259 | AK053807 | Mus musculus 0 day neonate eyeball cDNA, RIKEN full-length enriched library, clone:E130309L01 product:ADENYLATE KINA | 2.89 | 0.04125 |  | |
| A\_51\_P302450 | NM\_172589 | Mus musculus lipoma HMGIC fusion partner-like 2 (Lhfpl2), mRNA | 2.89 | 0.00402 |  | |
| A\_51\_P486560 | AK017182 | Mus musculus 11 days pregnant adult female ovary and uterus cDNA, RIKEN full-length enriched library, clone:5033421C21 pr | 2.88 | 0.00345 |  | |
| A\_51\_P376959 | NM\_019790 | Mus musculus transmembrane protein with EGF-like and two follistatin-like domains 2 (Tmeff2), mRNA | 2.87 | 0.00125 |  | |
| A\_51\_P249749 | NM\_031402 | Mus musculus cysteine-rich secretory protein LCCL domain containing 1 (Crispld1), mRNA | 2.85 | 0.00029 |  | |
| A\_51\_P406478 | TC1461965 | Q80U35 (Q80U35) MKIAA0337 protein (Fragment), complete | 2.84 | 0.00051 |  | |
| A\_51\_P248629 | NM\_175476 | Mus musculus Rho GTPase activating protein 25 (Arhgap25), mRNA | 2.84 | 0.00847 |  | |
| A\_51\_P319213 | NM\_175406 | Mus musculus ATPase, H+ transporting, V0 subunit D, isoform 2 (Atp6v0d2), mRNA | 2.83 | 0.03705 |  | |
| A\_51\_P469285 | NM\_008737 | Mus musculus neuropilin 1 (Nrp1), mRNA | 2.81 | 0.00248 |  | |
| A\_51\_P337412 | NM\_146015 | Mus musculus epidermal growth factor-containing fibulin-like extracellular matrix protein 1 (Efemp1), mRNA | 2.80 | 0.02325 |  | |
| A\_51\_P204080 | NM\_013820 | Mus musculus hexokinase 2 (Hk2), mRNA | 2.80 | 0.00002 |  | |
| A\_51\_P209902 | NM\_177814 | Mus musculus DNA segment, Chr 14, ERATO Doi 171, expressed (D14Ertd171e), mRNA | 2.80 | 0.00844 |  | |
| A\_51\_P269029 | NM\_009548 | Mus musculus zinc finger protein 179 (Zfp179), mRNA | 2.79 | 0.02386 |  | |
| A\_51\_P125727 | NM\_017400 | Mus musculus SH3-domain GRB2-like 3 (Sh3gl3), mRNA | 2.79 | 0.00173 |  | |
| A\_51\_P351194 | BC059093 | Mus musculus cornifelin, mRNA (cDNA clone MGC:70195 IMAGE:30293557), complete cds. | 2.78 | 0.00664 |  | |
| A\_51\_P396331 | NM\_013681 | Mus musculus synapsin II (Syn2), mRNA | 2.78 | 0.00302 |  | |
| A\_51\_P189962 | NM\_026380 | Mus musculus regulator of G-protein signaling 8 (Rgs8), mRNA | 2.76 | 0.00069 |  | |
| A\_51\_P268234 | NM\_029646 | Mus musculus RIKEN cDNA 2010004A03 gene (2010004A03Rik), mRNA | 2.75 | 0.00664 |  | |
| A\_51\_P485945 | NM\_010191 | Mus musculus farnesyl diphosphate farnesyl transferase 1 (Fdft1), mRNA | 2.74 | 0.00229 |  | |
| A\_51\_P398683 | NM\_026555 | Mus musculus reticulocalbin 3, EF-hand calcium binding domain (Rcn3), mRNA | 2.73 | 0.00005 |  | |
| A\_51\_P464238 | NM\_009716 | Mus musculus activating transcription factor 4 (Atf4), mRNA | 2.73 | 0.02291 |  | |
| A\_51\_P285137 | BC023075 | Mus musculus RIKEN cDNA 6430411K14 gene, mRNA (cDNA clone IMAGE:5361628), containing frame-shift errors. | 2.71 | 0.01175 |  | |
| A\_51\_P419116 | NM\_134050 | Mus musculus RAB15, member RAS oncogene family (Rab15), mRNA | 2.71 | 0.01635 |  | |
| A\_51\_P185248 | NM\_145741 | Mus musculus growth differentiation factor 10 (Gdf10), mRNA | 2.70 | 0.00011 |  | |
| A\_51\_P184261 | NM\_133832 | Mus musculus retinol dehydrogenase 10 (all-trans) (Rdh10), mRNA | 2.69 | 0.01937 |  | |
| A\_51\_P420600 | NM\_178738 | Mus musculus protease, serine, 35 (Prss35), mRNA | 2.69 | 0.00253 |  | |
| A\_51\_P510485 | BC046305 | Mus musculus RIKEN cDNA 2310047I15 gene, mRNA (cDNA clone MGC:54840 IMAGE:6493676), complete cds. | 2.69 | 0.02879 |  | |
| A\_51\_P176472 | AK010466 | Mus musculus ES cells cDNA, RIKEN full-length enriched library, clone:2410012C07 product:unknown EST, full insert sequenc | 2.69 | 0.00255 |  | |
| A\_51\_P398164 | NM\_178633 | Mus musculus kelch-like 2, Mayven (Drosophila) (Klhl2), mRNA | 2.68 | 0.01005 |  | |
| A\_51\_P297802 | NM\_001015046 | Mus musculus GTPase activating RANGAP domain-like 4 (Garnl4), mRNA | 2.68 | 0.00599 |  | |
| A\_51\_P323531 | AK006229 | Mus musculus adult male testis cDNA, RIKEN full-length enriched library, clone:1700021P22 product:hypothetical protein, full i | 2.67 | 0.00802 |  | |
| A\_51\_P222882 | NM\_010595 | Mus musculus potassium voltage-gated channel, shaker-related subfamily, member 1 (Kcna1), mRNA | 2.65 | 0.02066 |  | |
| A\_51\_P242978 | XM\_284451 | PREDICTED: similar to hypothetical protein | 2.65 | 0.00004 |  | |
| A\_51\_P313862 | NAP108668-1 | Unknown | 2.65 | 0.04794 |  | |
| A\_51\_P402818 | NM\_008506 | Mus musculus lung carcinoma myc related oncogene 1 (Lmyc1), mRNA | 2.64 | 0.00032 |  | |
| A\_51\_P166623 | NM\_177135 | Mus musculus RIKEN cDNA D830030K20 gene (D830030K20Rik), mRNA | 2.63 | 0.01102 |  | |
| A\_51\_P221762 | NM\_007936 | Mus musculus Eph receptor A4 (Epha4), mRNA | 2.62 | 0.00051 |  | |
| A\_51\_P137544 | NM\_010661 | Mus musculus keratin complex 1, acidic, gene 12 (Krt1-12), mRNA | 2.62 | 0.00494 |  | |
| A\_51\_P217226 | BI079946 | BI079946 602875955F1 NCI\_CGAP\_Mam2 Mus musculus cDNA clone IMAGE:5007924 5', mRNA sequence | 2.62 | 0.02379 |  | |
| A\_51\_P109709 | NM\_146750 | Mus musculus olfactory receptor 689 (Olfr689), mRNA | 2.61 | 0.01629 |  | |
| A\_51\_P178063 | NM\_009025 | Mus musculus RAS p21 protein activator 3 (Rasa3), mRNA | 2.61 | 0.00589 |  | |
| A\_51\_P452779 | NM\_133198 | Mus musculus liver glycogen phosphorylase (Pygl), mRNA | 2.61 | 0.00041 |  | |
| A\_51\_P339305 | NM\_016812 | Mus musculus Btg3 associated nuclear protein (Banp), mRNA | 2.60 | 0.00931 |  | |
| A\_51\_P246133 | NM\_010140 | Mus musculus Eph receptor A3 (Epha3), mRNA | 2.60 | 0.00936 |  | |
| A\_51\_P392943 | NM\_178728 | Mus musculus cDNA sequence AB112350 (AB112350), mRNA | 2.60 | 0.00001 |  | |
| A\_51\_P156108 | NM\_018827 | Mus musculus cytokine receptor-like factor 1 (Crlf1), mRNA | 2.60 | 0.01586 |  | |
| A\_51\_P395686 | AK017899 | Mus musculus adult male thymus cDNA, RIKEN full-length enriched library, clone:5830405M20 product:similar to RIBOSOMAL | 2.60 | 0.00688 |  | |
| A\_51\_P202592 | NM\_172628 | Mus musculus SH3 domain and tetratricopeptide repeats 2 (Sh3tc2), mRNA | 2.59 | 0.04020 |  | |
| A\_51\_P331886 | AK038316 | Mus musculus 16 days neonate thymus cDNA, RIKEN full-length enriched library, clone:A130095G14 product:inferred: human | 2.58 | 0.03582 |  | |
| A\_51\_P411645 | NM\_021500 | Mus musculus macrophage erythroblast attacher (Maea), mRNA | 2.58 | 0.03999 |  | |
| A\_51\_P288009 | NM\_009604 | Mus musculus cholinergic receptor, nicotinic, gamma polypeptide (Chrng), mRNA | 2.58 | 0.04332 |  | |
| A\_51\_P346543 | NM\_011600 | Mus musculus transducin-like enhancer of split 4, homolog of Drosophila E(spl) (Tle4), mRNA | 2.57 | 0.02661 |  | |
| A\_51\_P450487 | NM\_009270 | Mus musculus squalene epoxidase (Sqle), mRNA | 2.57 | 0.03904 |  | |
| A\_51\_P139320 | NM\_025273 | Mus musculus pterin 4 alpha carbinolamine dehydratase/dimerization cofactor of hepatocyte nuclear factor 1 alpha (TCF1) 1 (P | 2.57 | 0.00114 |  | |
| A\_51\_P237865 | NM\_021283 | Mus musculus interleukin 4 (Il4), mRNA | 2.56 | 0.01426 |  | |
| A\_51\_P482711 | AK010192 | Mus musculus adult male tongue cDNA, RIKEN full-length enriched library, clone:2310076D10 product:hypothetical protein, full | 2.55 | 0.00587 |  | |
| A\_51\_P267447 | NM\_009616 | Mus musculus a disintegrin and metalloproteinase domain 19 (meltrin beta) (Adam19), mRNA | 2.55 | 0.00878 |  | |
| A\_51\_P164995 | NM\_025285 | Mus musculus stathmin-like 2 (Stmn2), mRNA | 2.55 | 0.00652 |  | |
| A\_51\_P345159 | NM\_026024 | Mus musculus ubiquitin-conjugating enzyme E2T (putative) (Ube2t), mRNA | 2.54 | 0.00798 |  | |
| A\_51\_P237154 | NM\_023689 | Mus musculus sparc/osteonectin, cwcv and kazal-like domains proteoglycan 3 (Spock3), mRNA | 2.52 | 0.01460 |  | |
| A\_51\_P312465 | NM\_007459 | Mus musculus adaptor protein complex AP-2, alpha 2 subunit (Ap2a2), mRNA | 2.51 | 0.00786 |  | |
| A\_51\_P386964 | XM\_485063 | PREDICTED: hypothetical protein LOC70354 | 2.50 | 0.03517 |  | |
| A\_51\_P284686 | XM\_485800 | PREDICTED: AP2 associated kinase 1 | 2.50 | 0.00847 |  | |
| A\_51\_P502975 | NM\_146372 | Mus musculus olfactory receptor 509 (Olfr509), mRNA | 2.49 | 0.01371 |  | |
| A\_51\_P224424 | NM\_172781 | Mus musculus kelch-like 4 (Drosophila) (Klhl4), mRNA | 2.49 | 0.03017 |  | |
| A\_51\_P298837 | NM\_009262 | Mus musculus sparc/osteonectin, cwcv and kazal-like domains proteoglycan 1 (Spock1), mRNA | 2.49 | 0.04080 |  | |
| A\_51\_P188574 | XM\_146997 | PREDICTED: Mus musculus RIKEN cDNA 9930029P06 gene (9930029P06Rik), mRNA | 2.48 | 0.00110 |  | |
| A\_51\_P103594 | AK020485 | Mus musculus 12 days embryo embryonic body between diaphragm region and neck cDNA, RIKEN full-length enriched library, | 2.48 | 0.00688 |  | |
| A\_51\_P256747 | NM\_173029 | Mus musculus soluble adenylyl cyclase (Sacy), mRNA | 2.46 | 0.03574 |  | |
| A\_51\_P249494 | NM\_016807 | Mus musculus syndecan binding protein (Sdcbp), mRNA | 2.46 | 0.00029 |  | |
| A\_51\_P397154 | NAP063254-1 | Unknown | 2.46 | 0.00669 |  | |
| A\_51\_P427624 | NM\_025973 | Mus musculus progastricsin (pepsinogen C) (Pgc), mRNA | 2.45 | 0.02147 |  | |
| A\_51\_P178735 | NM\_146108 | Mus musculus 3-hydroxyisobutyryl-Coenzyme A hydrolase (Hibch), mRNA | 2.43 | 0.02860 |  | |
| A\_51\_P229875 | AK122457 | Mus musculus mRNA for mKIAA1147 protein | 2.43 | 0.00017 |  | |
| A\_51\_P359237 | NM\_025439 | Mus musculus transmembrane protein 9 (Tmem9), mRNA | 2.43 | 0.00388 |  | |
| A\_51\_P272363 | NM\_134160 | Mus musculus mucolipin 3 (Mcoln3), mRNA | 2.42 | 0.00491 |  | |
| A\_51\_P196207 | NM\_029341 | Mus musculus calcyphosine-like (Capsl), mRNA | 2.42 | 0.01575 |  | |
| A\_51\_P246124 | NM\_010113 | Mus musculus epidermal growth factor (Egf), mRNA | 2.42 | 0.02740 |  | |
| A\_51\_P359919 | NM\_177445 | Mus musculus aspartyl-tRNA synthetase (Dars), mRNA | 2.42 | 0.02049 |  | |
| A\_51\_P144850 | NM\_178685 | Mus musculus protocadherin 20 (Pcdh20), mRNA | 2.42 | 0.02357 |  | |
| A\_51\_P317211 | NM\_146256 | Mus musculus cDNA sequence BC034099 (BC034099), mRNA | 2.42 | 0.00075 |  | |
| A\_51\_P317640 | NM\_009367 | Mus musculus transforming growth factor, beta 2 (Tgfb2), mRNA | 2.41 | 0.03978 |  | |
| A\_51\_P318620 | NM\_019535 | Mus musculus SH3-domain GRB2-like 2 (Sh3gl2), mRNA | 2.40 | 0.00763 |  | |
| A\_51\_P240384 | NM\_032000 | Mus musculus trichorhinophalangeal syndrome I (human) (Trps1), mRNA | 2.38 | 0.00350 |  | |
| A\_51\_P475049 | NM\_011670 | Mus musculus ubiquitin carboxy-terminal hydrolase L1 (Uchl1), mRNA | 2.38 | 0.00517 |  | |
| A\_51\_P142299 | NM\_178382 | Mus musculus fibronectin leucine rich transmembrane protein 3 (Flrt3), mRNA | 2.38 | 0.01319 |  | |
| A\_51\_P157381 | AY523601 | Mus musculus CaMKII inhibitor protein alpha mRNA, complete cds. | 2.38 | 0.04918 |  | |
| A\_51\_P511439 | NM\_172718 | Mus musculus RUN and TBC1 domain containing 2 (Rutbc2), mRNA | 2.37 | 0.00116 |  | |
| A\_51\_P522004 | NM\_134448 | Mus musculus dystonin (Dst), transcript variant b, mRNA | 2.37 | 0.04874 |  | |
| A\_51\_P212038 | NM\_133764 | Mus musculus ATPase, H+ transporting, lysosomal, V0 subunit E isoform 2 (Atp6v0e2), mRNA | 2.36 | 0.00614 |  | |
| A\_51\_P110830 | NM\_013906 | Mus musculus a disintegrin-like and metalloprotease (reprolysin type) with thrombospondin type 1 motif, 8 (Adamts8), mRNA | 2.36 | 0.01098 |  | |
| A\_51\_P470715 | NM\_009895 | Mus musculus cytokine inducible SH2-containing protein (Cish), mRNA | 2.36 | 0.04361 |  | |
| A\_51\_P497295 | BC020023 | Mus musculus glutaminyl-peptide cyclotransferase (glutaminyl cyclase), mRNA (cDNA clone MGC:27858 IMAGE:3491756), co | 2.34 | 0.03505 |  | |
| A\_51\_P242622 | NM\_177350 | Mus musculus collomin (Colm), mRNA | 2.34 | 0.02867 |  | |
| A\_51\_P241074 | NM\_008927 | Mus musculus mitogen activated protein kinase kinase 1 (Map2k1), mRNA | 2.33 | 0.00340 |  | |
| A\_51\_P148814 | NM\_008529 | Mus musculus lymphocyte antigen 6 complex, locus E (Ly6e), mRNA | 2.33 | 0.01709 |  | |
| A\_51\_P164344 | AK015544 | Mus musculus adult male testis cDNA, RIKEN full-length enriched library, clone:4930471A21 product:unknown EST, full insert | 2.33 | 0.00079 |  | |
| A\_51\_P447248 | NM\_175105 | Mus musculus aquaporin 11 (Aqp11), mRNA | 2.32 | 0.00407 |  | |
| A\_51\_P234728 | NM\_175116 | Mus musculus purinergic receptor P2Y, G-protein coupled, 5 (P2ry5), mRNA | 2.32 | 0.02522 |  | |
| A\_51\_P444379 | NM\_198294 | Mus musculus RIKEN cDNA 1200003E16 gene (1200003E16Rik), mRNA | 2.30 | 0.00360 |  | |
| A\_51\_P146103 | NM\_008390 | Mus musculus interferon regulatory factor 1 (Irf1), mRNA | 2.30 | 0.03767 |  | |
| A\_51\_P275827 | NM\_144887 | Mus musculus zinc finger, DHHC domain containing 5 (Zdhhc5), mRNA | 2.30 | 0.03525 |  | |
| A\_51\_P296694 | NM\_023119 | Mus musculus enolase 1, alpha non-neuron (Eno1), mRNA | 2.30 | 0.00619 |  | |
| A\_51\_P189704 | NM\_025732 | Mus musculus RIKEN cDNA 4921520G13 gene (4921520G13Rik), mRNA | 2.29 | 0.01345 |  | |
| A\_51\_P330298 | NM\_031999 | Mus musculus transmembrane 7 superfamily member 1 (Tm7sf1), mRNA | 2.28 | 0.00060 |  | |
| A\_51\_P273626 | NM\_012061 | Mus musculus Ca<2+>dependent activator protein for secretion (Cadps), mRNA | 2.28 | 0.04155 |  | |
| A\_51\_P209372 | NM\_025436 | Mus musculus sterol-C4-methyl oxidase-like (Sc4mol), mRNA | 2.28 | 0.04576 |  | |
| A\_51\_P501793 | NM\_008237 | Mus musculus hairy and enhancer of split 3 (Drosophila) (Hes3), mRNA | 2.27 | 0.03829 |  | |
| A\_51\_P181676 | NM\_008398 | Mus musculus integrin alpha 7 (Itga7), mRNA | 2.26 | 0.00246 |  | |
| A\_51\_P317620 | NM\_182807 | Mus musculus expressed sequence AI851790 (AI851790), mRNA | 2.25 | 0.02704 |  | |
| A\_51\_P178692 | NM\_007930 | Mus musculus ectodermal-neural cortex 1 (Enc1), mRNA | 2.25 | 0.02267 |  | |
| A\_51\_P182471 | NM\_010160 | Mus musculus CUG triplet repeat, RNA binding protein 2 (Cugbp2), mRNA | 2.25 | 0.02724 |  | |
| A\_51\_P497039 | NM\_007863 | Mus musculus membrane protein, palmitoylated 3 (MAGUK p55 subfamily member 3) (Mpp3), mRNA | 2.24 | 0.01736 |  | |
| A\_51\_P329975 | NM\_016718 | Mus musculus ninjurin 2 (Ninj2), mRNA | 2.23 | 0.00004 |  | |
| A\_51\_P502919 | NAP057073-1 | Unknown | 2.23 | 0.02053 |  | |
| A\_51\_P355151 | AK013788 | Mus musculus adult male hippocampus cDNA, RIKEN full-length enriched library, clone:2900075A18 product:CAM-KII INHIBIT | 2.23 | 0.02386 |  | |
| A\_51\_P344249 | NM\_009685 | Mus musculus amyloid beta (A4) precursor protein-binding, family B, member 1 (Apbb1), mRNA | 2.23 | 0.01472 |  | |
| A\_51\_P460048 | NM\_029861 | Mus musculus RIKEN cDNA 1500041B16 gene (1500041B16Rik), mRNA | 2.22 | 0.03673 |  | |
| A\_51\_P215922 | NM\_009811 | Mus musculus caspase 6 (Casp6), mRNA | 2.22 | 0.00361 |  | |
| A\_51\_P424561 | NM\_009567 | Mus musculus zinc finger protein 93 (Zfp93), mRNA | 2.21 | 0.04937 |  | |
| A\_51\_P498267 | XM\_129028 | PREDICTED: similar to ankyrin repeat domain 29 | 2.21 | 0.01965 |  | |
| A\_51\_P148684 | NM\_175006 | Mus musculus POU domain, class 6, transcription factor 2 (Pou6f2), mRNA | 2.21 | 0.00786 |  | |
| A\_51\_P186601 | M29546 | Mouse MOD-1 null malic enzyme mRNA, partial cds. | 2.20 | 0.02855 |  | |
| A\_51\_P302823 | NM\_019686 | Mus musculus calcium and integrin binding family member 2 (Cib2), mRNA | 2.20 | 0.00530 |  | |
| A\_51\_P451428 | NM\_178410 | Mus musculus MARVEL (membrane-associating) domain containing 2 (Marveld2), mRNA | 2.20 | 0.00385 |  | |
| A\_51\_P419666 | BC054536 | Mus musculus a disintegrin and metalloprotease domain 11, mRNA (cDNA clone MGC:62319 IMAGE:6400084), complete cds | 2.20 | 0.04630 |  | |
| A\_51\_P490204 | NM\_026398 | Mus musculus processing of precursor 5, ribonuclease P/MRP family (S. cerevisiae) (Pop5), mRNA | 2.19 | 0.03096 |  | |
| A\_51\_P491147 | BC022600 | Mus musculus RIKEN cDNA 2810402K13 gene, mRNA (cDNA clone MGC:31102 IMAGE:4159269), complete cds. | 2.19 | 0.00664 |  | |
| A\_51\_P208472 | NM\_029901 | Mus musculus aldo-keto reductase family 1, member C21 (Akr1c21), mRNA | 2.19 | 0.00031 |  | |
| A\_51\_P195244 | NM\_029568 | Mus musculus microfibrillar-associated protein 4 (Mfap4), mRNA | 2.19 | 0.00658 |  | |
| A\_51\_P508289 | NM\_010145 | Mus musculus epoxide hydrolase 1, microsomal (Ephx1), mRNA | 2.18 | 0.02298 |  | |
| A\_51\_P343002 | NM\_172124 | Mus musculus beta-1,3-glucuronyltransferase 2 (glucuronosyltransferase S) (B3gat2), mRNA | 2.17 | 0.02147 |  | |
| A\_51\_P134741 | NM\_008180 | Mus musculus glutathione synthetase (Gss), mRNA | 2.17 | 0.03849 |  | |
| A\_51\_P429335 | NM\_019429 | Mus musculus protease, serine, 16 (thymus) (Prss16), mRNA | 2.17 | 0.02379 |  | |
| A\_51\_P431885 | NM\_198412 | Mus musculus DnaJ (Hsp40) homolog, subfamily C, member 6 (Dnajc6), mRNA | 2.16 | 0.02061 |  | |
| A\_51\_P156526 | AK032322 | Mus musculus adult male olfactory brain cDNA, RIKEN full-length enriched library, clone:6430519O13 product:PLASMA MEM | 2.16 | 0.01076 |  | |
| A\_51\_P496720 | NM\_019448 | Mus musculus DNA (cytosine-5-)-methyltransferase 3-like (Dnmt3l), mRNA | 2.16 | 0.01229 |  | |
| A\_51\_P246816 | NM\_025898 | Mus musculus N-ethylmaleimide sensitive fusion protein attachment protein alpha (Napa), mRNA | 2.16 | 0.01655 |  | |
| A\_51\_P217946 | AK083761 | Mus musculus 12 days embryo spinal ganglion cDNA, RIKEN full-length enriched library, clone:D130004H04 product:unknown | 2.16 | 0.00444 |  | |
| A\_51\_P465838 | NM\_021318 | Mus musculus four and a half LIM domains 5 (Fhl5), mRNA | 2.15 | 0.04803 |  | |
| A\_51\_P417025 | NM\_013837 | Mus musculus protein-tyrosine sulfotransferase 1 (Tpst1), mRNA | 2.15 | 0.00367 |  | |
| A\_51\_P279384 | NM\_016893 | Mus musculus fucosyltransferase 8 (Fut8), mRNA | 2.15 | 0.01965 |  | |
| A\_51\_P488024 | AK031899 | Mus musculus adult male medulla oblongata cDNA, RIKEN full-length enriched library, clone:6330442B14 product:weakly simil | 2.14 | 0.03582 |  | |
| A\_51\_P122232 | NM\_025657 | Mus musculus RIKEN cDNA 2810002D13 gene (2810002D13Rik), mRNA | 2.14 | 0.00367 |  | |
| A\_51\_P400555 | NM\_026906 | Mus musculus cathepsin 3 (Cts3), mRNA | 2.13 | 0.00859 |  | |
| A\_51\_P223085 | AK014830 | Mus musculus adult male testis cDNA, RIKEN full-length enriched library, clone:4921507A02 product:unclassifiable, full insert | 2.13 | 0.02746 |  | |
| A\_51\_P379953 | BC038619 | Mus musculus cDNA clone IMAGE:4219727, partial cds. | 2.13 | 0.02554 |  | |
| A\_51\_P469332 | AK034607 | Mus musculus 12 days embryo embryonic body between diaphragm region and neck cDNA, RIKEN full-length enriched library, | 2.13 | 0.00125 |  | |
| A\_51\_P461331 | XM\_487374 | PREDICTED: Mus musculus cadherin 9 (Cdh9), mRNA | 2.12 | 0.00181 |  | |
| A\_51\_P133229 | NM\_028072 | Mus musculus sulfatase 2 (Sulf2), mRNA | 2.12 | 0.04358 |  | |
| A\_51\_P214423 | BC049153 | Mus musculus RIKEN cDNA 2610024B07 gene, mRNA (cDNA clone IMAGE:6312095), partial cds | 2.11 | 0.00942 |  | |
| A\_51\_P277345 | NM\_017375 | Mus musculus osteoclast stimulating factor 1 (Ostf1), mRNA | 2.10 | 0.00077 |  | |
| A\_51\_P232281 | NM\_011109 | Mus musculus phospholipase A2, group IID (Pla2g2d), mRNA | 2.10 | 0.00587 |  | |
| A\_51\_P153812 | AK220368 | Mus musculus mRNA for mKIAA1063 protein | 2.09 | 0.00371 |  | |
| A\_51\_P214360 | NM\_133251 | Mus musculus vestigial like 1 homolog (Drosophila) (Vgll1), mRNA | 2.09 | 0.02828 |  | |
| A\_51\_P432764 | BC062197 | Mus musculus protein tyrosine phosphatase, non-receptor type substrate 1, mRNA (cDNA clone MGC:70224 IMAGE:5368250) | 2.09 | 0.04687 |  | |
| A\_51\_P362089 | NM\_146052 | Mus musculus leucine rich repeat containing 3B (Lrrc3b), mRNA | 2.08 | 0.00544 |  | |
| A\_51\_P240019 | NM\_013755 | Mus musculus glycogenin 1 (Gyg1), mRNA | 2.07 | 0.00120 |  | |
| A\_51\_P490700 | NM\_175430 | Mus musculus RIKEN cDNA B930008I02 gene (B930008I02Rik), mRNA | 2.07 | 0.04119 |  | |
| A\_51\_P263503 | NM\_011949 | Mus musculus mitogen activated protein kinase 1 (Mapk1), mRNA | 2.07 | 0.00511 |  | |
| A\_51\_P423527 | NM\_175007 | Mus musculus amphiphysin (Amph), mRNA | 2.06 | 0.00378 |  | |
| A\_51\_P251736 | NM\_007874 | Mus musculus deleted in polyposis 1 (Dp1), mRNA | 2.06 | 0.02150 |  | |
| A\_51\_P201444 | NAP057019-1 | Unknown | 2.06 | 0.01507 |  | |
| A\_51\_P461416 | XM\_129704 | PREDICTED: potassium voltage-gated channel, subfamily Q, member 5 | 2.06 | 0.00739 |  | |
| A\_51\_P295037 | NAP057010-1 | Unknown | 2.05 | 0.00244 |  | |
| A\_51\_P483168 | AW536275 | G0102E08-3 NIA Mouse E7.5 Embryonic Portion cDNA Library Mus musculus cDNA clone G0102E08 3'. | 2.05 | 0.02032 |  | |
| A\_51\_P208132 | NM\_175204 | Mus musculus RIKEN cDNA 5830406J20 gene (5830406J20Rik), mRNA | 2.04 | 0.00146 |  | |
| A\_51\_P355481 | NM\_025385 | Mus musculus RIKEN cDNA 1110020C13 gene (1110020C13Rik), mRNA | 2.03 | 0.01632 |  | |
| A\_51\_P275273 | NM\_010575 | Mus musculus integrin alpha 2b (Itga2b), mRNA | 2.03 | 0.01273 |  | |
| A\_51\_P312437 | NM\_025522 | Mus musculus dehydrogenase/reductase (SDR family) member 7 (Dhrs7), mRNA | 2.02 | 0.03806 |  | |
| A\_51\_P202332 | NM\_011126 | Mus musculus palate, lung, and nasal epithelium carcinoma associated (Plunc), mRNA | 2.02 | 0.04577 |  | |
| A\_51\_P308308 | NM\_172958 | Mus musculus phosphatidylinositol-3-phosphatase associated protein (Pip3ap), mRNA | 2.02 | 0.00786 |  | |
| A\_51\_P265145 | NM\_172751 | Mus musculus Rho guanine nucleotide exchange factor (GEF) 10 (Arhgef10), mRNA | 2.02 | 0.04558 |  | |
| A\_51\_P159874 | NM\_133853 | Mus musculus membrane associated guanylate kinase, WW and PDZ domain containing 3 (Magi3), mRNA | 2.02 | 0.02529 |  | |
| A\_51\_P237599 | NM\_009691 | Mus musculus amyloid beta (A4) precursor-like protein 2 (Aplp2), mRNA | 2.01 | 0.00646 |  | |
| A\_51\_P354262 | NM\_009256 | Mus musculus serine (or cysteine) proteinase inhibitor, clade B, member 9 (Serpinb9), mRNA | 2.01 | 0.00385 |  | |
| A\_51\_P373187 | NM\_025646 | Mus musculus RIKEN cDNA 0610009I22 gene (0610009I22Rik), transcript variant 2, mRNA | 2.01 | 0.00009 |  | |
| A\_51\_P388696 | NM\_010887 | Mus musculus NADH dehydrogenase (ubiquinone) Fe-S protein 4 (Ndufs4), mRNA | 2.01 | 0.03829 |  | |
| A\_51\_P190845 | NM\_007509 | Mus musculus ATPase, H+ transporting, V1 subunit B, isoform 2 (Atp6v1b2), mRNA | 2.01 | 0.02409 |  | |
| A\_51\_P517216 | NM\_016719 | Mus musculus growth factor receptor bound protein 14 (Grb14), mRNA | 2.00 | 0.01100 |  | |
| A\_51\_P265106 | NM\_145614 | Mus musculus dihydrolipoamide S-acetyltransferase (E2 component of pyruvate dehydrogenase complex) (Dlat), mRNA | 2.00 | 0.01718 |  | |
| A\_51\_P376525 | BU526046 | BU526046 AGENCOURT\_10296645 NIH\_MGC\_144 Mus musculus cDNA clone IMAGE:6534654 5', mRNA sequence | 2.00 | 0.04812 |  | |
| A\_51\_P116946 | NM\_080467 | Mus musculus ATPase, H+ transporting, lysosomal V0 subunit A isoform 4 (Atp6v0a4), mRNA | 1.99 | 0.00061 |  | |
| A\_51\_P167313 | NM\_171824 | Mus musculus piggyBac transposable element derived 5 (Pgbd5), mRNA | 1.98 | 0.01458 |  | |
| A\_51\_P233304 | NM\_027519 | Mus musculus RIKEN cDNA 6330406I15 gene (6330406I15Rik), mRNA | 1.98 | 0.01107 |  | |
| A\_51\_P136848 | AK050977 | Mus musculus 9 days embryo whole body cDNA, RIKEN full-length enriched library, clone:D030049E01 product:weakly similar | 1.98 | 0.00871 |  | |
| A\_51\_P428582 | NM\_009647 | Mus musculus adenylate kinase 3 alpha-like 1 (Ak3l1), mRNA | 1.97 | 0.00196 |  | |
| A\_51\_P328817 | NM\_183152 | Mus musculus RIKEN cDNA 6330514A18 gene (6330514A18Rik), mRNA | 1.97 | 0.03196 |  | |
| A\_51\_P200447 | L02241 | Mouse protein kinase inhibitor (testicular isoform) mRNA, complete cds. | 1.97 | 0.00501 |  | |
| A\_51\_P281586 | NM\_033569 | Mus musculus cyclin M2 (Cnnm2), mRNA | 1.97 | 0.00460 |  | |
| A\_51\_P300986 | NP377814 | GB|NM\_008580.1|NP\_032606.1 mitogen activated protein kinase kinase kinase 5; MEK kinase 5 | 1.97 | 0.00611 |  | |
| A\_51\_P280125 | NM\_176860 | Mus musculus RIKEN cDNA 2810457I06 gene (2810457I06Rik), mRNA | 1.96 | 0.00356 |  | |
| A\_51\_P490415 | NM\_025566 | Mus musculus tumor necrosis factor, alpha-induced protein 8-like 1 (Tnfaip8l1), mRNA | 1.96 | 0.03352 |  | |
| A\_51\_P466613 | NM\_011984 | Mus musculus homer homolog 3 (Drosophila) (Homer3), mRNA | 1.96 | 0.01234 |  | |
| A\_51\_P140742 | NM\_012043 | Mus musculus immunoglobulin superfamily containing leucine-rich repeat (Islr), mRNA | 1.96 | 0.01385 |  | |
| A\_51\_P133247 | AK076014 | Mus musculus 10 days embryo whole body cDNA, RIKEN full-length enriched library, clone:2610030J19 product:solute carrier f | 1.96 | 0.00006 |  | |
| A\_51\_P347965 | NM\_007427 | Mus musculus agouti related protein (Agrp), mRNA | 1.95 | 0.03744 |  | |
| A\_51\_P136124 | NM\_018871 | Mus musculus 3-monooxygenase/tryptophan 5-monooxygenase activation protein, gamma polypeptide (Ywhag), mRNA | 1.95 | 0.03009 |  | |
| A\_51\_P120636 | NM\_007891 | Mus musculus E2F transcription factor 1 (E2f1), mRNA | 1.95 | 0.02151 |  | |
| A\_51\_P320105 | NM\_026796 | Mus musculus SET and MYND domain containing 2 (Smyd2), mRNA | 1.95 | 0.02955 |  | |
| A\_51\_P138538 | NM\_026892 | Mus musculus RIKEN cDNA 1500010M16 gene (1500010M16Rik), mRNA | 1.94 | 0.02037 |  | |
| A\_51\_P211221 | NM\_025364 | Mus musculus RIKEN cDNA 1110005A23 gene (1110005A23Rik), mRNA | 1.94 | 0.03078 |  | |
| A\_51\_P145948 | NM\_144946 | Mus musculus neuropilin (NRP) and tolloid (TLL)-like 1 (Neto1), mRNA | 1.94 | 0.01062 |  | |
| A\_51\_P175736 | NM\_026553 | Mus musculus Yip1 interacting factor homolog (S. cerevisiae) (Yif1), mRNA | 1.94 | 0.03105 |  | |
| A\_51\_P267690 | NM\_153397 | Mus musculus a disintegrin and metalloprotease domain 32 (Adam32), mRNA | 1.93 | 0.04558 |  | |
| A\_51\_P333831 | CA451816 | UI-M-FZ0-ccq-e-15-0-UI.r1 NIH\_BMAP\_FZ0 Mus musculus cDNA clone IMAGE: 6823000 5', mRNA sequence | 1.93 | 0.00740 |  | |
| A\_51\_P387648 | NM\_008307 | Mus musculus HpaII tiny fragments locus 9c (Htf9c), mRNA | 1.92 | 0.00394 |  | |
| A\_51\_P119597 | NM\_009680 | Mus musculus adaptor-related protein complex 3, beta 1 subunit (Ap3b1), mRNA | 1.92 | 0.00569 |  | |
| A\_51\_P289464 | AK029184 | Mus musculus 0 day neonate head cDNA, RIKEN full-length enriched library, clone:4831410G10 product:DJ631M13.5.2 (NOVE | 1.92 | 0.01005 |  | |
| A\_51\_P174314 | NM\_019413 | Mus musculus roundabout homolog 1 (Drosophila) (Robo1), mRNA | 1.91 | 0.03843 |  | |
| A\_51\_P506792 | NM\_009412 | Mus musculus tumor protein D52 (Tpd52), transcript variant 5, mRNA | 1.90 | 0.00886 |  | |
| A\_51\_P186118 | NM\_024201 | Mus musculus RIKEN cDNA 0610011N22 gene (0610011N22Rik), mRNA | 1.90 | 0.02008 |  | |
| A\_51\_P461020 | NM\_009223 | Mus musculus stannin (Snn), mRNA | 1.90 | 0.01175 |  | |
| A\_51\_P413539 | NM\_021606 | Mus musculus NIMA (never in mitosis gene a)-related expressed kinase 6 (Nek6), mRNA | 1.90 | 0.04665 |  | |
| A\_51\_P379702 | NM\_022007 | Mus musculus FXYD domain-containing ion transport regulator 7 (Fxyd7), mRNA | 1.89 | 0.02066 |  | |
| A\_51\_P499653 | NM\_021369 | Mus musculus cholinergic receptor, nicotinic, alpha polypeptide 6 (Chrna6), mRNA | 1.88 | 0.02938 |  | |
| A\_51\_P268033 | BC026638 | Mus musculus mannoside acetylglucosaminyltransferase 4, isoenzyme B, mRNA (cDNA clone MGC:37502 IMAGE:4984907), | 1.88 | 0.04285 |  | |
| A\_51\_P168768 | A\_51\_P168768 | Unknown | 1.88 | 0.02049 |  | |
| A\_51\_P105837 | NM\_010828 | Mus musculus Cbp/p300-interacting transactivator, with Glu/Asp-rich carboxy-terminal domain, 2 (Cited2), mRNA | 1.88 | 0.03832 |  | |
| A\_51\_P360492 | NM\_008567 | Mus musculus minichromosome maintenance deficient 6 (MIS5 homolog, S. pombe) (S. cerevisiae) (Mcm6), mRNA | 1.88 | 0.01683 |  | |
| A\_51\_P514300 | NM\_009447 | Mus musculus tubulin, alpha 4 (Tuba4), mRNA | 1.87 | 0.02669 |  | |
| A\_51\_P134835 | NM\_207685 | Mus musculus ELAV (embryonic lethal, abnormal vision, Drosophila)-like 2 (Hu antigen B) (Elavl2), transcript variant 1, mRNA | 1.87 | 0.02042 |  | |
| A\_51\_P330481 | NM\_010714 | Mus musculus LIM homeobox protein 9 (Lhx9), transcript variant 2, mRNA | 1.86 | 0.00511 |  | |
| A\_51\_P372418 | NM\_026521 | Mus musculus RIKEN cDNA 3110006P09 gene (3110006P09Rik), mRNA | 1.85 | 0.03475 |  | |
| A\_51\_P406090 | AK020899 | Mus musculus adult retina cDNA, RIKEN full-length enriched library, clone:A930028L21 product:CDNA FLJ11219 FIS, CLONE | 1.85 | 0.00197 |  | |
| A\_51\_P295355 | NM\_011957 | Mus musculus cAMP responsive element binding protein 3-like 1 (Creb3l1), mRNA | 1.85 | 0.03687 |  | |
| A\_51\_P378856 | NM\_019703 | Mus musculus phosphofructokinase, platelet (Pfkp), mRNA | 1.85 | 0.03843 |  | |
| A\_51\_P335000 | NM\_010211 | Mus musculus four and a half LIM domains 1 (Fhl1), mRNA | 1.85 | 0.00425 |  | |
| A\_51\_P270960 | BC031532 | Mus musculus RIKEN cDNA 3222402P14 gene, mRNA (cDNA clone MGC:29057 IMAGE:3673874), complete cds. | 1.85 | 0.00114 |  | |
| A\_51\_P182257 | AK006134 | Mus musculus adult male testis cDNA, RIKEN full-length enriched library, clone:1700019N12 product:hypothetical protein, full i | 1.84 | 0.00057 |  | |
| A\_51\_P137317 | NM\_025873 | Mus musculus tRNA isopentenyltransferase 1 (Trit1), mRNA | 1.84 | 0.01339 |  | |
| A\_51\_P484869 | NM\_010255 | Mus musculus guanidinoacetate methyltransferase (Gamt), mRNA | 1.84 | 0.01871 |  | |
| A\_51\_P113403 | NM\_178743 | Mus musculus solute carrier family 26, member 11 (Slc26a11), mRNA | 1.84 | 0.03330 |  | |
| A\_51\_P308681 | AK018089 | Mus musculus 11 days embryo head cDNA, RIKEN full-length enriched library, clone:6230416A05 product:hypothetical protein, | 1.84 | 0.01511 |  | |
| A\_51\_P428903 | AK016770 | Mus musculus adult male testis cDNA, RIKEN full-length enriched library, clone:4933411G06 product:similar to UBIQUITIN-LIK | 1.84 | 0.02983 |  | |
| A\_51\_P492437 | NM\_024286 | Mus musculus popeye domain containing 3 (Popdc3), mRNA | 1.83 | 0.01937 |  | |
| A\_51\_P510957 | NM\_009679 | Mus musculus adaptor protein complex AP-2, mu1 (Ap2m1), mRNA | 1.83 | 0.03512 |  | |
| A\_51\_P429308 | BC068168 | Mus musculus neuropilin (NRP) and tolloid (TLL)-like 2, mRNA (cDNA clone IMAGE:30544936), with apparent retained intron | 1.82 | 0.04677 |  | |
| A\_51\_P247665 | NM\_178811 | Mus musculus tetratricopeptide repeat domain 15 (Ttc15), mRNA | 1.82 | 0.03866 |  | |
| A\_51\_P108173 | NAP099398-001 | Unknown | 1.81 | 0.00774 |  | |
| A\_51\_P162373 | AK079828 | Mus musculus 0 day neonate thymus cDNA, RIKEN full-length enriched library, clone:A430078A13 product:unknown EST, full i | 1.81 | 0.04812 |  | |
| A\_51\_P109766 | NM\_009503 | Mus musculus valosin containing protein (Vcp), mRNA | 1.80 | 0.02386 |  | |
| A\_51\_P160514 | NM\_021328 | Mus musculus bridging integrator 3 (Bin3), mRNA | 1.80 | 0.00012 |  | |
| A\_51\_P450839 | NM\_027307 | Mus musculus golgi phosphoprotein 2 (Golph2), mRNA | 1.79 | 0.04131 |  | |
| A\_51\_P281612 | NM\_147082 | Mus musculus olfactory receptor 609 (Olfr609), mRNA | 1.79 | 0.00886 |  | |
| A\_51\_P293110 | NM\_010818 | Mus musculus Cd200 antigen (Cd200), mRNA | 1.79 | 0.00144 |  | |
| A\_51\_P351371 | NM\_021288 | Mus musculus thymidylate synthase (Tyms), mRNA | 1.79 | 0.01062 |  | |
| A\_51\_P267194 | NM\_011810 | Mus musculus Fas apoptotic inhibitory molecule (Faim), mRNA | 1.78 | 0.00335 |  | |
| A\_51\_P383949 | AK220560 | Mus musculus mRNA for mKIAA4233 protein | 1.78 | 0.03096 |  | |
| A\_51\_P273686 | NM\_028803 | Mus musculus glucan (1,4-alpha-), branching enzyme 1 (Gbe1), mRNA | 1.78 | 0.04611 |  | |
| A\_51\_P444483 | NM\_007755 | Mus musculus cytoplasmic polyadenylation element binding protein 1 (Cpeb1), mRNA | 1.78 | 0.00944 |  | |
| A\_51\_P363258 | NM\_133731 | Mus musculus protease, serine, 22 (Prss22), mRNA | 1.77 | 0.00051 |  | |
| A\_51\_P123805 | XM\_355637 | PREDICTED: sno, strawberry notch homolog 1 | 1.77 | 0.01780 |  | |
| A\_51\_P398191 | AK028848 | Mus musculus 10 days neonate skin cDNA, RIKEN full-length enriched library, clone:4732462B05 product:hypothetical Histidin | 1.76 | 0.02036 |  | |
| A\_51\_P212682 | NM\_027290 | Mus musculus minichromosome maintenance deficient 10 (S. cerevisiae) (Mcm10), mRNA | 1.76 | 0.01571 |  | |
| A\_51\_P483180 | BC087942 | Mus musculus sorting nexin 7, mRNA (cDNA clone MGC:107328 IMAGE:30303462), complete cds. | 1.75 | 0.00074 |  | |
| A\_51\_P493558 | NM\_145417 | Mus musculus arginyl aminopeptidase (aminopeptidase B) (Rnpep), mRNA | 1.74 | 0.02890 |  | |
| A\_51\_P177744 | NM\_028864 | Mus musculus zinc finger CCCH type, antiviral 1 (Zc3hav1), mRNA | 1.74 | 0.03806 |  | |
| A\_51\_P278840 | NM\_177321 | Mus musculus melanoma inhibitory activity 2 (Mia2), mRNA | 1.74 | 0.02196 |  | |
| A\_51\_P431985 | NM\_031202 | Mus musculus tyrosinase-related protein 1 (Tyrp1), mRNA | 1.74 | 0.01268 |  | |
| A\_51\_P289341 | AK030947 | Mus musculus adult male thymus cDNA, RIKEN full-length enriched library, clone:5830467P10 product:unknown EST, full inser | 1.74 | 0.00494 |  | |
| A\_51\_P233947 | NM\_144522 | Mus musculus TBC1 domain family, member 10b (Tbc1d10b), mRNA | 1.74 | 0.00299 |  | |
| A\_51\_P210395 | NM\_053108 | Mus musculus glutaredoxin 1 (thioltransferase) (Glrx1), mRNA | 1.74 | 0.03698 |  | |
| A\_51\_P280404 | NM\_134065 | Mus musculus ependymin related protein 2 (zebrafish) (Epdr2), mRNA | 1.73 | 0.03809 |  | |
| A\_51\_P340226 | NM\_013709 | Mus musculus Sh3 domain YSC-like 1 (Sh3yl1), mRNA | 1.73 | 0.02314 |  | |
| A\_51\_P468388 | NM\_011235 | Mus musculus RAD51-like 3 (S. cerevisiae) (Rad51l3), mRNA | 1.72 | 0.01511 |  | |
| A\_51\_P231979 | NM\_013472 | Mus musculus annexin A6 (Anxa6), mRNA | 1.72 | 0.03764 |  | |
| A\_51\_P410744 | AK049335 | Mus musculus ES cells cDNA, RIKEN full-length enriched library, clone:C330025F22 product:diacylglycerol kinase, epsilon, ful | 1.71 | 0.02124 |  | |
| A\_51\_P281033 | NM\_178893 | Mus musculus coronin, actin binding protein 2A (Coro2a), mRNA | 1.71 | 0.01228 |  | |
| A\_51\_P378545 | NM\_028030 | Mus musculus RNA binding protein with multiple splicing 2 (Rbpms2), mRNA | 1.70 | 0.04358 |  | |
| A\_51\_P232355 | NM\_008950 | Mus musculus protease (prosome, macropain) 26S subunit, ATPase 5 (Psmc5), mRNA | 1.70 | 0.01770 |  | |
| A\_51\_P357422 | NM\_025556 | Mus musculus RIKEN cDNA 2410022L05 gene (2410022L05Rik), mRNA | 1.70 | 0.03461 |  | |
| A\_51\_P259902 | NM\_022992 | Mus musculus ADP-ribosylation factor-like 6 interacting protein 5 (Arl6ip5), mRNA | 1.69 | 0.00038 |  | |
| A\_51\_P193011 | NM\_008450 | Mus musculus kinesin 2 (Kns2), transcript variant a, mRNA | 1.69 | 0.00857 |  | |
| A\_51\_P305140 | NM\_008181 | Mus musculus glutathione S-transferase, alpha 1 (Ya) (Gsta1), mRNA | 1.69 | 0.04780 |  | |
| A\_51\_P421477 | AK173135 | Mus musculus mRNA for mKIAA1238 protein | 1.69 | 0.02867 |  | |
| A\_51\_P381178 | AK038975 | Mus musculus adult male hypothalamus cDNA, RIKEN full-length enriched library, clone:A230080K23 product:similar to DJ583 | 1.69 | 0.00543 |  | |
| A\_51\_P184006 | NM\_018867 | Mus musculus carboxypeptidase X 2 (M14 family) (Cpxm2), mRNA | 1.69 | 0.01314 |  | |
| A\_51\_P473252 | NM\_011777 | Mus musculus zyxin (Zyx), mRNA | 1.69 | 0.00252 |  | |
| A\_51\_P440267 | AI851444 | UI-M-BH0-akj-e-08-0-UI.s1 NIH\_BMAP\_M\_S1 Mus musculus cDNA clone UI-M-BH0-akj-e-08-0-UI 3'. | 1.68 | 0.02036 |  | |
| A\_51\_P434979 | NM\_029098 | Mus musculus DNA segment, Chr 15, ERATO Doi 735, expressed (D15Ertd735e), mRNA | 1.68 | 0.00837 |  | |
| A\_51\_P499776 | NM\_026603 | Mus musculus density-regulated protein (Denr), mRNA | 1.68 | 0.02518 |  | |
| A\_51\_P464576 | NM\_008943 | Mus musculus presenilin 1 (Psen1), mRNA | 1.67 | 0.03512 |  | |
| A\_51\_P164014 | AK049676 | Mus musculus 12 days embryo spinal cord cDNA, RIKEN full-length enriched library, clone:C530022J18 product:hypothetical Li | 1.67 | 0.01699 |  | |
| A\_51\_P327141 | NM\_009539 | Mus musculus zeta-chain (TCR) associated protein kinase (Zap70), mRNA | 1.67 | 0.00035 |  | |
| A\_51\_P232399 | NM\_027857 | Mus musculus aspartoacylase (aminoacylase) 3 (Acy3), mRNA | 1.67 | 0.02794 |  | |
| A\_51\_P507130 | NM\_025411 | Mus musculus RIKEN cDNA 1110049F12 gene (1110049F12Rik), mRNA | 1.66 | 0.01209 |  | |
| A\_51\_P310225 | NM\_011664 | Mus musculus ubiquitin B (Ubb), mRNA | 1.66 | 0.03542 |  | |
| A\_51\_P269728 | NM\_011186 | Mus musculus proteasome (prosome, macropain) subunit, beta type 5 (Psmb5), mRNA | 1.66 | 0.00878 |  | |
| A\_51\_P319234 | XM\_485461 | PREDICTED: Mus musculus ubiquitin specific protease 48 (Usp48), mRNA | 1.66 | 0.02843 |  | |
| A\_51\_P392546 | NM\_146159 | Mus musculus cDNA sequence BC023882 (BC023882), mRNA | 1.66 | 0.01655 |  | |
| A\_51\_P304397 | AK017670 | Mus musculus 8 days embryo whole body cDNA, RIKEN full-length enriched library, clone:5730456K23 product:hypothetical Zi | 1.66 | 0.01592 |  | |
| A\_51\_P313067 | AK035153 | Mus musculus 12 days embryo embryonic body between diaphragm region and neck cDNA, RIKEN full-length enriched library, | 1.66 | 0.01718 |  | |
| A\_51\_P114459 | NM\_027537 | Mus musculus RIKEN cDNA 6330404A07 gene (6330404A07Rik), mRNA | 1.65 | 0.03753 |  | |
| A\_51\_P295575 | NM\_025530 | Mus musculus cutC copper transporter homolog (E.coli) (Cutc), mRNA | 1.65 | 0.04408 |  | |
| A\_51\_P114002 | AK002213 | Mus musculus adult male kidney cDNA, RIKEN full-length enriched library, clone:0610005A07 product:GLUTATHIONE S-TRAN | 1.65 | 0.00786 |  | |
| A\_51\_P387006 | AI587890 | mp54h10.y1 Soares\_thymus\_2NbMT Mus musculus cDNA clone IMAGE:573091 5'. | 1.65 | 0.00852 |  | |
| A\_51\_P447600 | AK078630 | Mus musculus adult male adrenal gland cDNA, RIKEN full-length enriched library, clone:7330410C13 product:signal peptide, C | 1.65 | 0.02323 |  | |
| A\_51\_P354681 | NM\_026243 | Mus musculus RIKEN cDNA 9130411I17 gene (9130411I17Rik), mRNA | 1.65 | 0.02036 |  | |
| A\_51\_P289647 | NM\_173386 | Mus musculus RIKEN cDNA E330016A19 gene (E330016A19Rik), mRNA | 1.65 | 0.01803 |  | |
| A\_51\_P514776 | NM\_009771 | Mus musculus beta-transducin repeat containing protein (Btrc), mRNA | 1.65 | 0.02481 |  | |
| A\_51\_P501963 | NM\_021463 | Mus musculus phosphoribosyl pyrophosphate synthetase 1 (Prps1), mRNA | 1.64 | 0.01468 |  | |
| A\_51\_P193302 | NM\_025305 | Mus musculus mitchondrial ribosomal protein S7 (Mrps7), mRNA | 1.64 | 0.03828 |  | |
| A\_51\_P229196 | NM\_026220 | Mus musculus microfibrillar-associated protein 1 (Mfap1), mRNA | 1.64 | 0.02740 |  | |
| A\_51\_P438002 | NM\_026523 | Mus musculus neuromedin B (Nmb), mRNA | 1.64 | 0.03674 |  | |
| A\_51\_P409468 | XM\_619748 | PREDICTED: Mus musculus similar to olfactory receptor Olfr367 (LOC545417), mRNA | 1.64 | 0.01948 |  | |
| A\_51\_P267441 | NM\_025416 | Mus musculus RIKEN cDNA 1110038F21 gene (1110038F21Rik), mRNA | 1.63 | 0.01189 |  | |
| A\_51\_P226645 | AK047238 | Mus musculus 10 days neonate cerebellum cDNA, RIKEN full-length enriched library, clone:B930041E13 product:hypothetical | 1.63 | 0.00012 |  | |
| A\_51\_P216905 | NM\_007438 | Mus musculus aldolase 1, A isoform (Aldoa), mRNA | 1.63 | 0.01948 |  | |
| A\_51\_P360531 | NM\_138662 | Mus musculus protocadherin alpha 3 (Pcdha3), mRNA | 1.63 | 0.03926 |  | |
| A\_51\_P450088 | XM\_484897 | PREDICTED: procollagen, type VI, alpha 3 | 1.62 | 0.01339 |  | |
| A\_51\_P182104 | NM\_152823 | Mus musculus unc-5 homolog C (C. elegans)-like (Unc5cl), mRNA | 1.62 | 0.03311 |  | |
| A\_51\_P400366 | NM\_021375 | Mus musculus Rhesus blood group-associated B glycoprotein (Rhbg), mRNA | 1.62 | 0.00204 |  | |
| A\_51\_P297346 | NM\_029153 | Mus musculus secretory carrier membrane protein 1 (Scamp1), mRNA | 1.62 | 0.01413 |  | |
| A\_51\_P178646 | NM\_026308 | Mus musculus ribonuclease P 21 subunit (human) (Rpp21), mRNA | 1.62 | 0.01418 |  | |
| A\_51\_P129260 | BI662359 | BI662359 603305916F1 NCI\_CGAP\_Mam4 Mus musculus cDNA clone IMAGE:5351666 5', mRNA sequence | 1.61 | 0.00300 |  | |
| A\_51\_P474408 | NM\_025868 | Mus musculus RIKEN cDNA 2310042M24 gene (2310042M24Rik), mRNA | 1.61 | 0.01751 |  | |
| A\_51\_P402165 | NM\_011880 | Mus musculus regulator of G protein signaling 7 (Rgs7), mRNA | 1.61 | 0.02359 |  | |
| A\_51\_P138044 | NM\_019739 | Mus musculus forkhead box O1 (Foxo1), mRNA | 1.61 | 0.04125 |  | |
| A\_51\_P189105 | AK077275 | Mus musculus 11 days pregnant adult female ovary and uterus cDNA, RIKEN full-length enriched library, clone:5033401M15 pr | 1.60 | 0.02529 |  | |
| A\_51\_P112223 | NM\_010357 | Mus musculus glutathione S-transferase, alpha 4 (Gsta4), mRNA | 1.60 | 0.02575 |  | |
| A\_51\_P326932 | AK018172 | Mus musculus adult male medulla oblongata cDNA, RIKEN full-length enriched library, clone:6330414G02 product:unknown ES | 1.59 | 0.04965 |  | |
| A\_51\_P184198 | NM\_031878 | Mus musculus SWI/SNF related, matrix associated, actin dependent regulator of chromatin, subfamily d, member 2 (Smarcd2) | 1.59 | 0.01592 |  | |
| A\_51\_P321531 | AF479672 | Mus musculus Pur-gamma B-form (Purg) mRNA, complete cds; alternatively spliced. | 1.59 | 0.03113 |  | |
| A\_51\_P477066 | NM\_020507 | Mus musculus transducer of ERBB2, 2 (Tob2), mRNA | 1.59 | 0.00847 |  | |
| A\_51\_P396983 | NM\_175525 | Mus musculus RIKEN cDNA D630042P16 gene (D630042P16Rik), mRNA | 1.59 | 0.02337 |  | |
| A\_51\_P367011 | NM\_207236 | Mus musculus olfactory receptor 1283 (Olfr1283), mRNA | 1.59 | 0.00105 |  | |
| A\_51\_P383063 | NM\_147060 | Mus musculus olfactory receptor 667 (Olfr667), mRNA | 1.59 | 0.01507 |  | |
| A\_51\_P278464 | AK013920 | Mus musculus 13 days embryo head cDNA, RIKEN full-length enriched library, clone:3100002J23 product:hypothetical protein, | 1.58 | 0.00609 |  | |
| A\_51\_P388281 | NM\_009835 | Mus musculus chemokine (C-C motif) receptor 6 (Ccr6), mRNA | 1.58 | 0.00232 |  | |
| A\_51\_P211519 | NM\_030728 | Mus musculus RIKEN cDNA 9930013L23 gene (9930013L23Rik), mRNA | 1.58 | 0.01678 |  | |
| A\_51\_P242779 | NM\_025975 | Mus musculus t-complex-associated-testis-expressed 1-like (Tcte1l), mRNA | 1.58 | 0.03986 |  | |
| A\_51\_P381387 | NM\_153415 | Mus musculus protein-O-mannosyltransferase 2 (Pomt2), mRNA | 1.58 | 0.00745 |  | |
| A\_51\_P205390 | NM\_028491 | Mus musculus RIKEN cDNA 1700040L02 gene (1700040L02Rik), mRNA | 1.58 | 0.03311 |  | |
| A\_51\_P262773 | NM\_007664 | Mus musculus cadherin 2 (Cdh2), mRNA | 1.58 | 0.03837 |  | |
| A\_51\_P393602 | NM\_023721 | Mus musculus ATPase, H+ transporting, V1 subunit D (Atp6v1d), mRNA | 1.57 | 0.02879 |  | |
| A\_51\_P117439 | NM\_011847 | Mus musculus DnaJ (Hsp40) homolog, subfamily B, member 6 (Dnajb6), mRNA | 1.57 | 0.02574 |  | |
| A\_51\_P466558 | XM\_131619 | PREDICTED: similar to BC022150 protein | 1.57 | 0.02105 |  | |
| A\_51\_P367395 | NM\_138583 | Mus musculus DNA segment, Chr 16, human D22S680E, expressed (D16H22S680E), mRNA | 1.56 | 0.00840 |  | |
| A\_51\_P382204 | NM\_177605 | Mus musculus hypothetical protein 9130207N01 (9130207N01), mRNA | 1.56 | 0.00704 |  | |
| A\_51\_P492366 | NM\_007557 | Mus musculus bone morphogenetic protein 7 (Bmp7), mRNA | 1.56 | 0.04937 |  | |
| A\_51\_P155234 | NM\_009760 | Mus musculus BCL2/adenovirus E1B 19kDa-interacting protein 1, NIP3 (Bnip3), mRNA | 1.55 | 0.01221 |  | |
| A\_51\_P380772 | NM\_178224 | Mus musculus cystathionine beta-synthase (Cbs), transcript variant 2, mRNA | 1.55 | 0.02727 |  | |
| A\_51\_P348151 | NM\_145128 | Mus musculus mannoside acetylglucosaminyltransferase 5 (Mgat5), mRNA | 1.55 | 0.00248 |  | |
| A\_51\_P447326 | NM\_026646 | Mus musculus solute carrier family 25 (mitochondrial carrier, glutamate), member 22 (Slc25a22), mRNA | 1.55 | 0.01533 |  | |
| A\_51\_P397876 | NM\_010134 | Mus musculus engrailed 2 (En2), mRNA | 1.54 | 0.03843 |  | |
| A\_51\_P438841 | NM\_009819 | Mus musculus catenin (cadherin associated protein), alpha 2 (Ctnna2), transcript variant 2, mRNA | 1.54 | 0.00361 |  | |
| A\_51\_P384033 | NM\_011183 | Mus musculus presenilin 2 (Psen2), mRNA | 1.54 | 0.03767 |  | |
| A\_51\_P300456 | AK122223 | Mus musculus mRNA for mKIAA0230 protein | 1.54 | 0.00019 |  | |
| A\_51\_P369850 | NM\_133364 | Mus musculus proline rich membrane anchor 1 (Prima1), transcript variant I, mRNA | 1.54 | 0.00745 |  | |
| A\_51\_P500814 | NM\_010708 | Mus musculus lectin, galactose binding, soluble 9 (Lgals9), mRNA | 1.54 | 0.01941 |  | |
| A\_51\_P229536 | NM\_020282 | Mus musculus NAD(P)H dehydrogenase, quinone 2 (Nqo2), mRNA | 1.54 | 0.02327 |  | |
| A\_51\_P139370 | NM\_146769 | Mus musculus olfactory receptor 1110 (Olfr1110), mRNA | 1.53 | 0.04677 |  | |
| A\_51\_P342387 | NM\_013475 | Mus musculus apolipoprotein H (Apoh), mRNA | 1.53 | 0.02288 |  | |
| A\_51\_P163306 | NM\_016918 | Mus musculus nudix (nucleoside diphosphate linked moiety X)-type motif 5 (Nudt5), mRNA | 1.53 | 0.02095 |  | |
| A\_51\_P457584 | AK090159 | Mus musculus 13 days embryo spinal cord cDNA, RIKEN full-length enriched library, clone:G630008C16 product:FLAVOHEM | 1.53 | 0.01998 |  | |
| A\_51\_P325624 | NM\_024219 | Mus musculus heat shock factor binding protein 1 (Hsbp1), mRNA | 1.53 | 0.00664 |  | |
| A\_51\_P373379 | NM\_009975 | Mus musculus casein kinase II, beta subunit (Csnk2b), mRNA | 1.53 | 0.01280 |  | |
| A\_51\_P515349 | AK049359 | Mus musculus ES cells cDNA, RIKEN full-length enriched library, clone:C330027M21 product:PHOSPHATIDYLGLYCEROPHO | 1.52 | 0.00798 |  | |
| A\_51\_P320843 | NM\_025921 | Mus musculus RIKEN cDNA 2610002M06 gene (2610002M06Rik), mRNA | 1.51 | 0.03165 |  | |
| A\_51\_P403578 | AK007907 | Mus musculus 10 day old male pancreas cDNA, RIKEN full-length enriched library, clone:1810059H22 product:hypothetical pro | 1.51 | 0.03092 |  | |
| A\_51\_P362671 | AK031890 | Mus musculus adult male medulla oblongata cDNA, RIKEN full-length enriched library, clone:6330441F13 product:potassium v | 1.51 | 0.02453 |  | |
| A\_51\_P124345 | NM\_153166 | Mus musculus copine V (Cpne5), mRNA | 1.51 | 0.04803 |  | |
| A\_51\_P326152 | NM\_011377 | Mus musculus single-minded homolog 2 (Drosophila) (Sim2), mRNA | 1.51 | 0.02004 |  | |
| A\_51\_P470751 | NM\_033354 | Mus musculus leucine zipper transcription regulator 2 (Lztr2), mRNA | 1.50 | 0.01040 |  | |
| A\_51\_P187612 | NM\_134095 | Mus musculus DNA segment, Chr 15, Wayne State University 75, expressed (D15Wsu75e), mRNA | 1.50 | 0.02895 |  | |
| A\_51\_P327828 | NM\_011149 | Mus musculus peptidylprolyl isomerase B (Ppib), mRNA | 1.50 | 0.00619 |  | |
| A\_51\_P187093 | NM\_010270 | Mus musculus mitochondrial ribosomal protein S33 (Mrps33), nuclear gene encoding mitochondrial protein, transcript variant 1, | 1.50 | 0.01529 |  | |
| A\_51\_P474902 | AK049089 | Mus musculus 0 day neonate cerebellum cDNA, RIKEN full-length enriched library, clone:C230097P10 product:similar to ACTI | 1.50 | 0.02447 |  | |
| A\_51\_P445765 | TC1519807 | Unknown | 1.49 | 0.00611 |  | |
| A\_51\_P141071 | NM\_025958 | Mus musculus cullin-associated and neddylation-dissociated 2 (putative) (Cand2), mRNA | 1.49 | 0.02032 |  | |
| A\_51\_P126584 | NAP057195-1 | Unknown | 1.49 | 0.00808 |  | |
| A\_51\_P108266 | NM\_033268 | Mus musculus actinin alpha 2 (Actn2), mRNA | 1.48 | 0.00619 |  | |
| A\_51\_P108645 | NM\_010587 | Mus musculus intersectin 1 (SH3 domain protein 1A) (Itsn1), mRNA | 1.48 | 0.02024 |  | |
| A\_51\_P409311 | NM\_199303 | Mus musculus bactericidal/permeability-increasing protein-like 3 (Bpil3), mRNA | 1.47 | 0.01060 |  | |
| A\_51\_P484290 | NM\_178798 | Mus musculus solute carrier family 7 (cationic amino acid transporter, y+ system), member 6 (Slc7a6), mRNA | 1.47 | 0.03687 |  | |
| A\_51\_P176912 | NM\_139145 | Mus musculus holocarboxylase synthetase (biotin- | 1.47 | 0.04600 |  | |
| A\_51\_P461191 | NM\_029674 | Mus musculus RIKEN cDNA 1700083M11 gene (1700083M11Rik), mRNA | 1.47 | 0.02610 |  | |
| A\_51\_P185181 | NM\_007499 | Mus musculus ataxia telangiectasia mutated homolog (human) (Atm), mRNA | 1.47 | 0.01062 |  | |
| A\_51\_P157462 | NM\_009060 | Mus musculus regucalcin (Rgn), mRNA | 1.46 | 0.04345 |  | |
| A\_51\_P159612 | NM\_019487 | Mus musculus heme binding protein 2 (Hebp2), mRNA | 1.46 | 0.01795 |  | |
| A\_51\_P116407 | NM\_013753 | Mus musculus cDNA sequence X99384 (X99384), mRNA | 1.45 | 0.02434 |  | |
| A\_51\_P271160 | NM\_147068 | Mus musculus olfactory receptor 166 (Olfr166), mRNA | 1.45 | 0.01255 |  | |
| A\_51\_P399866 | NM\_133255 | Mus musculus hook homolog 2 (Drosophila) (Hook2), mRNA | 1.45 | 0.02632 |  | |
| A\_51\_P442097 | NM\_027868 | Mus musculus solute carrier family 41, member 3 (Slc41a3), mRNA | 1.45 | 0.01780 |  | |
| A\_51\_P438039 | NM\_183272 | Mus musculus RIKEN cDNA 1700063I17 gene (1700063I17Rik), mRNA | 1.44 | 0.01529 |  | |
| A\_51\_P179757 | NM\_026256 | Mus musculus RIKEN cDNA 4921537P18 gene (4921537P18Rik), mRNA | 1.44 | 0.04621 |  | |
| A\_51\_P207988 | NM\_008965 | Mus musculus prostaglandin E receptor 4 (subtype EP4) (Ptger4), mRNA | 1.44 | 0.01780 |  | |
| A\_51\_P518893 | NM\_001002787 | Mus musculus calicin (Ccin), mRNA | 1.44 | 0.01685 |  | |
| A\_51\_P103890 | AK045394 | Mus musculus adult male corpora quadrigemina cDNA, RIKEN full-length enriched library, clone:B230112L11 product:hypotheti | 1.43 | 0.01829 |  | |
| A\_51\_P205480 | NM\_144843 | Mus musculus myotubularin related protein 6 (Mtmr6), mRNA | 1.43 | 0.03341 |  | |
| A\_51\_P162786 | NM\_024475 | Mus musculus ubiquitin-like domain containing CTD phosphatase 1 (Ublcp1), mRNA | 1.43 | 0.04833 |  | |
| A\_51\_P413445 | AK016098 | Mus musculus adult male testis cDNA, RIKEN full-length enriched library, clone:4930552N02 product:unclassifiable, full insert | 1.43 | 0.02419 |  | |
| A\_51\_P155514 | NM\_011164 | Mus musculus prolactin (Prl), mRNA | 1.42 | 0.01376 |  | |
| A\_51\_P242024 | NM\_018753 | Mus musculus tyrosine 3-monooxygenase/tryptophan 5-monooxygenase activation protein, beta polypeptide (Ywhab), mRNA | 1.42 | 0.01043 |  | |
| A\_51\_P420655 | NM\_180588 | Mus musculus RIKEN cDNA 2700029E10 gene (2700029E10Rik), mRNA | 1.42 | 0.03113 |  | |
| A\_51\_P366079 | NM\_146391 | Mus musculus olfactory receptor 1058 (Olfr1058), mRNA | 1.41 | 0.01339 |  | |
| A\_51\_P352052 | NM\_008819 | Mus musculus phosphatidylethanolamine N-methyltransferase (Pemt), mRNA | 1.41 | 0.02447 |  | |
| A\_51\_P399545 | NM\_175251 | Mus musculus AT rich interactive domain 2 (Arid-rfx like) (Arid2), mRNA | 1.41 | 0.04713 |  | |
| A\_51\_P351937 | NM\_145147 | Mus musculus GTP binding protein 6 (putative) (Gtpbp6), mRNA | 1.41 | 0.01533 |  | |
| A\_51\_P276939 | NM\_198101 | Mus musculus Gem-interacting protein (Gmip), mRNA | 1.41 | 0.00664 |  | |
| A\_51\_P414927 | NM\_027388 | Mus musculus phosphatidylinositol glycan, class W (Pigw), mRNA | 1.40 | 0.04611 |  | |
| A\_51\_P467751 | NM\_133970 | Mus musculus DNA segment, Chr 8, ERATO Doi 354, expressed (D8Ertd354e), mRNA | 1.40 | 0.02409 |  | |
| A\_51\_P519811 | NM\_018751 | Mus musculus sulfotransferase family, cytosolic, 1C, member 1 (Sult1c1), mRNA | 1.40 | 0.00633 |  | |
| A\_51\_P128174 | NM\_007937 | Mus musculus Eph receptor A5 (Epha5), mRNA | 1.40 | 0.01464 |  | |
| A\_51\_P485542 | NM\_018737 | Mus musculus cytidine 5'-triphosphate synthase 2 (Ctps2), mRNA | 1.39 | 0.00465 |  | |
| A\_51\_P213031 | NM\_134147 | Mus musculus RIKEN cDNA D930010J01 gene (D930010J01Rik), mRNA | 1.39 | 0.01676 |  | |
| A\_51\_P247542 | NM\_172799 | Mus musculus RIKEN cDNA 4932418K24 gene (4932418K24Rik), mRNA | 1.39 | 0.03843 |  | |
| A\_51\_P404236 | NM\_033617 | Mus musculus ATPase, H+ transporting, V0 subunit B (Atp6v0b), mRNA | 1.39 | 0.04386 |  | |
| A\_51\_P138909 | AK014573 | Mus musculus 0 day neonate skin cDNA, RIKEN full-length enriched library, clone:4632413I24 product:weakly similar to HISTO | 1.38 | 0.04779 |  | |
| A\_51\_P373737 | NAP057059-1 | Unknown | 1.38 | 0.03129 |  | |
| A\_51\_P138807 | AK006001 | Mus musculus adult male testis cDNA, RIKEN full-length enriched library, clone:1700015L13 product:hypothetical protein, full i | 1.38 | 0.03608 |  | |
| A\_51\_P464234 | NM\_008020 | Mus musculus FK506 binding protein 2 (Fkbp2), mRNA | 1.37 | 0.02834 |  | |
| A\_51\_P334174 | NM\_145383 | Mus musculus rhodopsin (Rho), mRNA | 1.37 | 0.02966 |  | |
| A\_51\_P493940 | AK006268 | Mus musculus adult male testis cDNA, RIKEN full-length enriched library, clone:1700023D19 product:hypothetical protein, full i | 1.36 | 0.01998 |  | |
| A\_51\_P268154 | NM\_019477 | Mus musculus acyl-CoA synthetase long-chain family member 4 (Acsl4), transcript variant 2, mRNA | 1.36 | 0.03329 |  | |
| A\_51\_P483639 | AK039115 | Mus musculus adult male hypothalamus cDNA, RIKEN full-length enriched library, clone:A230098A12 product:hypothetical Fibr | 1.36 | 0.03475 |  | |
| A\_51\_P294255 | NM\_178413 | Mus musculus cDNA sequence BC051244 (BC051244), mRNA | 1.35 | 0.03587 |  | |
| A\_51\_P259879 | NM\_173430 | Mus musculus fukutin related protein (Fkrp), mRNA | 1.35 | 0.02504 |  | |
| A\_51\_P161043 | AK220167 | Mus musculus mRNA for mKIAA4025 protein | 1.35 | 0.04265 |  | |
| A\_51\_P148509 | NM\_011652 | Mus musculus titin (Ttn), mRNA | 1.35 | 0.04865 |  | |
| A\_51\_P182116 | NM\_019466 | Mus musculus Down syndrome critical region homolog 1 (human) (Dscr1), mRNA | 1.35 | 0.04345 |  | |
| A\_51\_P482123 | NM\_007902 | Mus musculus endothelin 2 (Edn2), mRNA | 1.35 | 0.04918 |  | |
| A\_51\_P103396 | NM\_016879 | Mus musculus keratin complex 2, basic, gene 18 (Krt2-18), mRNA | 1.34 | 0.02692 |  | |
| A\_51\_P221755 | NM\_010116 | Mus musculus kallikrein 9 (Klk9), mRNA | 1.34 | 0.02283 |  | |
| A\_51\_P438756 | AK079064 | Mus musculus adult male diencephalon cDNA, RIKEN full-length enriched library, clone:9330120A15 product:unknown EST, ful | 1.34 | 0.01230 |  | |
| A\_51\_P469551 | AK035644 | Mus musculus adult male urinary bladder cDNA, RIKEN full-length enriched library, clone:9530080J18 product:reserpine-sensiti | 1.34 | 0.03017 |  | |
| A\_51\_P185693 | NM\_031197 | Mus musculus solute carrier family 2 (facilitated glucose transporter), member 2 (Slc2a2), mRNA | 1.33 | 0.01575 |  | |
| A\_51\_P307831 | NM\_183124 | Mus musculus defensin beta 41 (Defb41), mRNA | 1.33 | 0.04611 |  | |
| A\_51\_P329094 | BC087920 | Mus musculus RIKEN cDNA 4930550L24 gene, mRNA (cDNA clone MGC:107266 IMAGE:30281980), complete cds. | 1.33 | 0.02025 |  | |
| A\_51\_P249173 | NM\_010643 | Mus musculus kallikrein 24 (Klk24), mRNA | 1.33 | 0.02684 |  | |
| A\_51\_P488739 | NM\_030701 | Mus musculus G protein-coupled receptor 109B (Gpr109b), mRNA | 1.33 | 0.03229 |  | |
| A\_51\_P362423 | NM\_010841 | Mus musculus metallothionein-like 5, testis-specific (tesmin) (Mtl5), mRNA | 1.32 | 0.01756 |  | |
| A\_51\_P381611 | NM\_029413 | Mus musculus microrchidia 4 (Morc4), mRNA | 1.32 | 0.02975 |  | |
| A\_51\_P137125 | NM\_016749 | Mus musculus myosin binding protein H (Mybph), mRNA | 1.32 | 0.03062 |  | |
| A\_51\_P485421 | AK088666 | Mus musculus 2 days neonate thymus thymic cells cDNA, RIKEN full-length enriched library, clone:E430023B15 product:imm | 1.32 | 0.03330 |  | |
| A\_51\_P162955 | NM\_177920 | Mus musculus serine (or cysteine) proteinase inhibitor, clade A (alpha-1 antiproteinase, antitrypsin), member 7 (Serpina7), mR | 1.32 | 0.04623 |  | |
| A\_51\_P211210 | AK122456 | Mus musculus mRNA for mKIAA1146 protein | 1.32 | 0.01822 |  | |
| A\_51\_P348183 | BC048537 | Mus musculus DNA segment, Chr 2, ERATO Doi 217, expressed, mRNA (cDNA clone MGC:58482 IMAGE:6532162), complet | 1.32 | 0.04519 |  | |
| A\_51\_P455371 | NM\_178263 | Mus musculus ankyrin repeat domain 27 (VPS9 domain) (Ankrd27), transcript variant 2, mRNA | 1.32 | 0.04266 |  | |
| A\_51\_P175180 | XM\_283487 | PREDICTED: serine protease inhibitor, Kazal type 5 | 1.31 | 0.02492 |  | |
| A\_51\_P130567 | NM\_020291 | Mus musculus olfactory receptor 480 (Olfr480), mRNA | 1.31 | 0.02409 |  | |
| A\_51\_P113585 | NM\_146050 | Mus musculus oncoprotein induced transcript 1 (Oit1), mRNA | 1.31 | 0.03770 |  | |
| A\_51\_P264194 | A\_51\_P264194 | Unknown | 1.31 | 0.03820 |  | |
| A\_51\_P427964 | AK030676 | Mus musculus 6 days neonate head cDNA, RIKEN full-length enriched library, clone:5430424A14 product:similar to CDNA FLJ | 1.31 | 0.01797 |  | |
| A\_51\_P406433 | AK016466 | Mus musculus adult male testis cDNA, RIKEN full-length enriched library, clone:4931420C21 product:hypothetical SEC7-like d | 1.31 | 0.03770 |  | |
| A\_51\_P179921 | NM\_008988 | Mus musculus putative neuronal cell adhesion molecule (Punc), mRNA | 1.31 | 0.04777 |  | |
| A\_51\_P267617 | NM\_008125 | Mus musculus gap junction membrane channel protein beta 2 (Gjb2), mRNA | 1.30 | 0.03859 |  | |
| A\_51\_P446696 | NM\_134183 | Mus musculus vomeronasal 1 receptor, C28 (V1rc28), mRNA | 1.30 | 0.04577 |  | |
| A\_51\_P164762 | NAP048022-1 | Unknown | 1.30 | 0.01888 |  | |
| A\_51\_P315837 | AK036347 | Mus musculus 16 days neonate cerebellum cDNA, RIKEN full-length enriched library, clone:9630059K23 product:similar to MA | 1.29 | 0.03525 |  | |
| A\_51\_P381086 | NM\_178699 | Mus musculus RIKEN cDNA B930041F14 gene (B930041F14Rik), mRNA | 1.29 | 0.03809 |  | |
| A\_51\_P302538 | NM\_145458 | Mus musculus PX domain containing serine/threonine kinase (Pxk), mRNA | 1.29 | 0.04812 |  | |
| A\_51\_P508959 | NM\_030024 | Mus musculus RIKEN cDNA E130201N16 gene (E130201N16Rik), mRNA | 1.28 | 0.03840 |  | |
| A\_51\_P391560 | NM\_198927 | Mus musculus submandibular gland protein C (Smgc), mRNA | 1.28 | 0.04713 |  | |
| A\_51\_P253207 | BC024441 | Mus musculus RIKEN cDNA D630035O19 gene, mRNA (cDNA clone MGC:37279 IMAGE:4973857), complete cds. | 1.28 | 0.04119 |  | |
| A\_51\_P370315 | NM\_011937 | Mus musculus glucosamine-6-phosphate deaminase 1 (Gnpda1), mRNA | 1.27 | 0.02323 |  | |
| A\_51\_P520956 | AK006959 | Mus musculus adult male testis cDNA, RIKEN full-length enriched library, clone:1700080O16 product:hypothetical MAGE famil | 1.26 | 0.02662 |  | |
| A\_51\_P105424 | NM\_029831 | Mus musculus RIKEN cDNA 1700127D06 gene (1700127D06Rik), mRNA | 1.25 | 0.02860 |  | |
| A\_51\_P406193 | AK020389 | Mus musculus adult male diencephalon cDNA, RIKEN full-length enriched library, clone:9330179O15 product:hypothetical prot | 1.25 | 0.04862 |  | |
| A\_51\_P298688 | AK030411 | Mus musculus adult male pituitary gland cDNA, RIKEN full-length enriched library, clone:5330408N05 product:hypothetical prot | 1.25 | 0.03843 |  | |
| A\_51\_P521176 | NM\_025886 | Mus musculus RIKEN cDNA 2400009B11 gene (2400009B11Rik), mRNA | 1.23 | 0.04937 |  | |
| A\_51\_P277174 | NM\_011161 | Mus musculus mitogen-activated protein kinase 11 (Mapk11), mRNA | 1.23 | 0.04408 |  | |
| A\_51\_P383703 | NM\_007500 | Mus musculus atonal homolog 1 (Drosophila) (Atoh1), mRNA | 0.77 | 0.04751 |  | |
| A\_51\_P336161 | NM\_010764 | Mus musculus mannosidase 2, alpha B1 (Man2b1), mRNA | 0.76 | 0.03254 |  | |
| A\_51\_P518919 | NM\_172853 | Mus musculus cadherin 7, type 2 (Cdh7), mRNA | 0.76 | 0.04713 |  | |
| A\_51\_P120912 | AK048960 | Mus musculus 0 day neonate cerebellum cDNA, RIKEN full-length enriched library, clone:C230085N17 product:ZETA-SARCOG | 0.76 | 0.03417 |  | |
| A\_51\_P188605 | NM\_198424 | Mus musculus cDNA sequence BC061259 (BC061259), mRNA | 0.76 | 0.03461 |  | |
| A\_51\_P185563 | NM\_145742 | Mus musculus DEAH (Asp-Glu-Ala-His) box polypeptide 35 (Dhx35), mRNA | 0.75 | 0.01655 |  | |
| A\_51\_P425944 | NM\_008468 | Mus musculus karyopherin (importin) alpha 6 (Kpna6), mRNA | 0.75 | 0.01948 |  | |
| A\_51\_P480578 | NM\_008797 | Mus musculus pyruvate carboxylase (Pcx), mRNA | 0.75 | 0.03285 |  | |
| A\_51\_P289697 | NM\_011849 | Mus musculus NIMA (never in mitosis gene a)-related expressed kinase 4 (Nek4), mRNA | 0.74 | 0.04027 |  | |
| A\_51\_P474151 | NM\_177746 | Mus musculus diacylglycerol O-acyltransferase 2-like 4 (Dgat2l4), mRNA | 0.73 | 0.04007 |  | |
| A\_51\_P491240 | AK011092 | Mus musculus 13 days embryo liver cDNA, RIKEN full-length enriched library, clone:2510042H12 product:weakly similar to RA | 0.73 | 0.04577 |  | |
| A\_51\_P309534 | NM\_019781 | Mus musculus peroxisomal biogenesis factor 14 (Pex14), mRNA | 0.73 | 0.04253 |  | |
| A\_51\_P264013 | NM\_026979 | Mus musculus C1q and tumor necrosis factor related protein 2 (C1qtnf2), mRNA | 0.73 | 0.01530 |  | |
| A\_51\_P460929 | NM\_153176 | Mus musculus spastic paraplegia 7 homolog (human) (Spg7), mRNA | 0.73 | 0.04265 |  | |
| A\_51\_P512541 | AK049709 | Mus musculus 12 days embryo spinal cord cDNA, RIKEN full-length enriched library, clone:C530043L06 product:similar to HC | 0.72 | 0.03391 |  | |
| A\_51\_P136143 | NM\_011786 | Mus musculus arachidonate lipoxygenase 3 (Aloxe3), mRNA | 0.72 | 0.04212 |  | |
| A\_51\_P222071 | NM\_153088 | Mus musculus CTD (carboxy-terminal domain, RNA polymerase II, polypeptide A) small phosphatase 1 (Ctdsp1), mRNA | 0.72 | 0.02762 |  | |
| A\_51\_P348855 | NM\_010127 | Mus musculus POU domain, class 6, transcription factor 1 (Pou6f1), mRNA | 0.72 | 0.01674 |  | |
| A\_51\_P344758 | AK005698 | Mus musculus adult male testis cDNA, RIKEN full-length enriched library, clone:1700007D05 product:hypothetical protein, full i | 0.71 | 0.00692 |  | |
| A\_51\_P518503 | BC068305 | Mus musculus Sloan-Kettering viral oncogene homolog, mRNA (cDNA clone MGC:76530 IMAGE:30093626), complete cds | 0.71 | 0.04976 |  | |
| A\_51\_P275382 | TC1538535 | Unknown | 0.71 | 0.03829 |  | |
| A\_51\_P346214 | NM\_013529 | Mus musculus glutamine fructose-6-phosphate transaminase 2 (Gfpt2), mRNA | 0.71 | 0.04865 |  | |
| A\_51\_P325102 | NAP057186-1 | Unknown | 0.71 | 0.01937 |  | |
| A\_51\_P218948 | NM\_030168 | Mus musculus RIKEN cDNA 4921505C17 gene (4921505C17Rik), mRNA | 0.70 | 0.02008 |  | |
| A\_51\_P115738 | NM\_053248 | Mus musculus solute carrier family 5 (sodium iodide symporter), member 5 (Slc5a5), mRNA | 0.70 | 0.01175 |  | |
| A\_51\_P207310 | U85089 | Mus musculus thioredoxin mRNA, nuclear gene encoding mitochondrial protein, complete cds. | 0.70 | 0.01632 |  | |
| A\_51\_P102782 | NM\_199195 | Mus musculus branched chain ketoacid dehydrogenase E1, beta polypeptide (Bckdhb), mRNA | 0.70 | 0.02314 |  | |
| A\_51\_P129937 | NM\_052973 | Mus musculus striatin, calmodulin binding protein 3 (Strn3), mRNA | 0.70 | 0.01949 |  | |
| A\_51\_P417643 | NM\_008259 | Mus musculus forkhead box A1 (Foxa1), mRNA | 0.70 | 0.02481 |  | |
| A\_51\_P227165 | NM\_025865 | Mus musculus RIKEN cDNA 2310030G06 gene (2310030G06Rik), mRNA | 0.70 | 0.01780 |  | |
| A\_51\_P518576 | NM\_145919 | Mus musculus abhydrolase domain containing 14A (Abhd14a), mRNA | 0.70 | 0.00599 |  | |
| A\_51\_P157737 | NM\_172483 | Mus musculus zinc finger protein 180 (Zfp180), mRNA | 0.70 | 0.04610 |  | |
| A\_51\_P428157 | NM\_028351 | Mus musculus thrombospondin, type I, domain 2 (Thsd2), mRNA | 0.70 | 0.02130 |  | |
| A\_51\_P472393 | AK015205 | Mus musculus adult male testis cDNA, RIKEN full-length enriched library, clone:4930426D05 product:hypothetical protein, full i | 0.70 | 0.01644 |  | |
| A\_51\_P206563 | AK008751 | Mus musculus adult male stomach cDNA, RIKEN full-length enriched library, clone:2210018M11 product:CDNA FLJ13589 FIS, | 0.69 | 0.01291 |  | |
| A\_51\_P294195 | A\_51\_P294195 | Unknown | 0.69 | 0.04577 |  | |
| A\_51\_P489003 | AK129355 | Mus musculus mRNA for mKIAA1422 protein | 0.69 | 0.01616 |  | |
| A\_51\_P356045 | AK122358 | Mus musculus mRNA for mKIAA0734 protein | 0.69 | 0.03148 |  | |
| A\_51\_P488383 | XM\_135197 | PREDICTED: protein tyrosine phosphatase, non-receptor type 23 | 0.69 | 0.00282 |  | |
| A\_51\_P141288 | NM\_010693 | Mus musculus lymphocyte protein tyrosine kinase (Lck), mRNA | 0.68 | 0.04965 |  | |
| A\_51\_P273489 | AK029819 | Mus musculus adult male testis cDNA, RIKEN full-length enriched library, clone:4930580E03 product:weakly similar to CENTA | 0.68 | 0.01677 |  | |
| A\_51\_P479929 | NM\_024176 | Mus musculus Dr1 associated protein 1 (negative cofactor 2 alpha) (Drap1), mRNA | 0.68 | 0.04713 |  | |
| A\_51\_P501429 | AK081839 | Mus musculus 16 days embryo head cDNA, RIKEN full-length enriched library, clone:C130081C23 product:CDNA FLJ13096 FI | 0.68 | 0.00614 |  | |
| A\_51\_P142760 | NM\_029102 | Mus musculus glycosyltransferase 8 domain containing 2 (Glt8d2), mRNA | 0.68 | 0.03475 |  | |
| A\_51\_P103209 | NM\_028639 | Mus musculus tetratricopeptide repeat domain 7 (Ttc7), mRNA | 0.68 | 0.00859 |  | |
| A\_51\_P456816 | NM\_172932 | Mus musculus neuroligin 3 (Nlgn3), mRNA | 0.68 | 0.04577 |  | |
| A\_51\_P137254 | NM\_025727 | Mus musculus kelch-like 10 (Drosophila) (Klhl10), mRNA | 0.68 | 0.02575 |  | |
| A\_51\_P416308 | XM\_110968 | PREDICTED: dynein, axonemal, heavy polypeptide 9 | 0.67 | 0.03525 |  | |
| A\_51\_P381218 | BB138485 | BB138485 RIKEN full-length enriched, adult male bone Mus musculus cDNA clone 9830148A07 3'. | 0.67 | 0.00931 |  | |
| A\_51\_P233059 | NM\_008477 | Mus musculus kinectin 1 (Ktn1), mRNA | 0.67 | 0.00830 |  | |
| A\_51\_P350996 | NM\_080461 | Mus musculus zinc finger protein 358 (Zfp358), mRNA | 0.67 | 0.04855 |  | |
| A\_51\_P416660 | NM\_172587 | Mus musculus CDC14 cell division cycle 14 homolog B (S. cerevisiae) (Cdc14b), mRNA | 0.66 | 0.00801 |  | |
| A\_51\_P124132 | NM\_029562 | Mus musculus cytochrome P450, family 2, subfamily d, polypeptide 26 (Cyp2d26), mRNA | 0.66 | 0.00342 |  | |
| A\_51\_P266191 | AK018108 | Mus musculus adult male medulla oblongata cDNA, RIKEN full-length enriched library, clone:6330403N15 product:hypothetical | 0.66 | 0.02968 |  | |
| A\_51\_P420100 | NM\_025736 | Mus musculus RIKEN cDNA 4921531G14 gene (4921531G14Rik), mRNA | 0.66 | 0.02968 |  | |
| A\_51\_P216303 | NM\_008608 | Mus musculus matrix metalloproteinase 14 (membrane-inserted) (Mmp14), mRNA | 0.66 | 0.03843 |  | |
| A\_51\_P196056 | NM\_212437 | Mus musculus widely-interspaced zinc finger motifs (Wiz), transcript variant 3, mRNA | 0.66 | 0.04577 |  | |
| A\_51\_P121797 | A\_51\_P121797 | Unknown | 0.66 | 0.01632 |  | |
| A\_51\_P440865 | NM\_173426 | Mus musculus RIKEN cDNA 1700012H17 gene (1700012H17Rik), mRNA | 0.66 | 0.02573 |  | |
| A\_51\_P203062 | NM\_010135 | Mus musculus enabled homolog (Drosophila) (Enah), mRNA | 0.65 | 0.04558 |  | |
| A\_51\_P333159 | NM\_026653 | Mus musculus replication protein A1 (Rpa1), mRNA | 0.65 | 0.04972 |  | |
| A\_51\_P110010 | NM\_027946 | Mus musculus WD repeat domain 68 (Wdr68), mRNA | 0.65 | 0.01709 |  | |
| A\_51\_P223462 | NM\_025994 | Mus musculus EF hand domain containing 2 (Efhd2), mRNA | 0.65 | 0.04566 |  | |
| A\_51\_P507500 | TC1478257 | JC4947 ADP-ribosylation factor 3 - mouse {Mus musculus;} , complete | 0.65 | 0.02879 |  | |
| A\_51\_P220934 | NM\_026955 | Mus musculus RIKEN cDNA 2200002K05 gene (2200002K05Rik), mRNA | 0.65 | 0.01511 |  | |
| A\_51\_P399653 | NM\_009953 | Mus musculus corticotropin releasing hormone receptor 2 (Crhr2), mRNA | 0.64 | 0.04630 |  | |
| A\_51\_P109539 | NM\_001015681 | Mus musculus RIKEN cDNA E130308A19 gene (E130308A19Rik), transcript variant 2, mRNA | 0.64 | 0.00833 |  | |
| A\_51\_P124467 | BC063268 | Mus musculus cDNA sequence BC033915, mRNA (cDNA clone MGC:68087 IMAGE:4187769), complete cds | 0.64 | 0.04938 |  | |
| A\_51\_P469661 | NM\_134115 | Mus musculus serine/threonine kinase 38 (Stk38), mRNA | 0.64 | 0.03950 |  | |
| A\_51\_P368262 | NM\_011630 | Mus musculus nuclear receptor subfamily 2, group C, member 2 (Nr2c2), mRNA | 0.64 | 0.03525 |  | |
| A\_51\_P469252 | NM\_172817 | Mus musculus zinc finger protein 647 (Zfp647), mRNA | 0.64 | 0.04345 |  | |
| A\_51\_P251737 | AK005439 | Mus musculus adult female placenta cDNA, RIKEN full-length enriched library, clone:1600013E24 product:weakly similar to UB | 0.64 | 0.00522 |  | |
| A\_51\_P173911 | NM\_008070 | Mus musculus gamma-aminobutyric acid (GABA-A) receptor, subunit beta 2 (Gabrb2), mRNA | 0.64 | 0.04611 |  | |
| A\_51\_P427151 | NM\_138671 | Mus musculus NAD kinase (Nadk), mRNA | 0.64 | 0.01240 |  | |
| A\_51\_P349783 | NM\_007388 | Mus musculus acid phosphatase 5, tartrate resistant (Acp5), mRNA | 0.64 | 0.00766 |  | |
| A\_51\_P101777 | NM\_026101 | Mus musculus hect domain and RLD 4 (Herc4), mRNA | 0.63 | 0.01746 |  | |
| A\_51\_P261359 | NM\_172692 | Mus musculus glucosidase beta 2 (Gba2), mRNA | 0.63 | 0.00742 |  | |
| A\_51\_P309731 | NM\_019779 | Mus musculus cytochrome P450, family 11, subfamily a, polypeptide 1 (Cyp11a1), mRNA | 0.63 | 0.00925 |  | |
| A\_51\_P494342 | NM\_212457 | Mus musculus brain expressed, X-linked 4 (Bex4), mRNA | 0.63 | 0.03770 |  | |
| A\_51\_P152404 | NM\_026166 | Mus musculus RIKEN cDNA 1200009F10 gene (1200009F10Rik), transcript variant 1, mRNA | 0.63 | 0.04345 |  | |
| A\_51\_P325318 | NM\_172702 | Mus musculus tumor differentially expressed 2-like (Tde2l), mRNA | 0.63 | 0.00669 |  | |
| A\_51\_P379478 | NM\_172484 | Mus musculus RIKEN cDNA E030049G20 gene (E030049G20Rik), mRNA | 0.63 | 0.01175 |  | |
| A\_51\_P409170 | NM\_008492 | Mus musculus lactate dehydrogenase 2, B chain (Ldh2), mRNA | 0.63 | 0.03350 |  | |
| A\_51\_P180091 | NM\_028775 | Mus musculus cytochrome P450, family 2, subfamily s, polypeptide 1 (Cyp2s1), mRNA | 0.62 | 0.00382 |  | |
| A\_51\_P508411 | NM\_177856 | Mus musculus hypothetical protein E130311K13 (E130311K13), mRNA | 0.62 | 0.02076 |  | |
| A\_51\_P237553 | NM\_009652 | Mus musculus thymoma viral proto-oncogene 1 (Akt1), mRNA | 0.62 | 0.02185 |  | |
| A\_51\_P517834 | AK012806 | Mus musculus 10, 11 days embryo whole body cDNA, RIKEN full-length enriched library, clone:2810025A12 product:hypotheti | 0.62 | 0.04847 |  | |
| A\_51\_P126136 | NM\_007751 | Mus musculus cytochrome c oxidase, subunit VIIIb (Cox8b), mRNA | 0.62 | 0.01575 |  | |
| A\_51\_P183853 | NM\_178602 | Mus musculus glutamate receptor, ionotropic, N-methyl D-aspartate-like 1A (Grinl1a), mRNA | 0.62 | 0.00669 |  | |
| A\_51\_P170176 | NM\_146184 | Mus musculus UDP-Gal:betaGal beta 1,3-galactosyltransferase polypeptide 7 (B7galt7), mRNA | 0.62 | 0.00772 |  | |
| A\_51\_P496640 | XM\_128751 | PREDICTED: cysteine-rich motor neuron 1 | 0.62 | 0.01062 |  | |
| A\_51\_P380069 | NM\_198962 | Mus musculus hypocretin (orexin) receptor 2 (Hcrtr2), mRNA | 0.62 | 0.01682 |  | |
| A\_51\_P207921 | NM\_008898 | Mus musculus P450 (cytochrome) oxidoreductase (Por), mRNA | 0.62 | 0.02151 |  | |
| A\_51\_P188772 | NM\_012026 | Mus musculus Rho-guanine nucleotide exchange factor (Rgnef), mRNA | 0.62 | 0.01716 |  | |
| A\_51\_P359800 | NM\_009131 | Mus musculus C-type lectin domain family 11, member a (Clec11a), mRNA | 0.62 | 0.02908 |  | |
| A\_51\_P463570 | NM\_008641 | Mus musculus microtubule associated serine/threonine kinase 2 (Mast2), mRNA | 0.61 | 0.04594 |  | |
| A\_51\_P483220 | NM\_008378 | Mus musculus imprinted and ancient (Impact), mRNA | 0.61 | 0.03165 |  | |
| A\_51\_P218091 | NM\_010195 | Mus musculus leucine rich repeat containing G protein coupled receptor 5 (Lgr5), mRNA | 0.61 | 0.00197 |  | |
| A\_51\_P425149 | BG294923 | BG294923 602392238F1 NIH\_MGC\_94 Mus musculus cDNA clone IMAGE:4504089 5', mRNA sequence | 0.61 | 0.02277 |  | |
| A\_51\_P118704 | NM\_007823 | Mus musculus cytochrome P450, family 4, subfamily b, polypeptide 1 (Cyp4b1), mRNA | 0.61 | 0.01511 |  | |
| A\_51\_P277005 | NM\_175140 | Mus musculus carbohydrate (N-acetylgalactosamine 4-0) sulfotransferase 8 (Chst8), mRNA | 0.61 | 0.00878 |  | |
| A\_51\_P487228 | NM\_172148 | Mus musculus cDNA sequence BC028440 (BC028440), mRNA | 0.61 | 0.00395 |  | |
| A\_51\_P164606 | NM\_010311 | Mus musculus guanine nucleotide binding protein, alpha z subunit (Gnaz), mRNA | 0.61 | 0.01605 |  | |
| A\_51\_P243138 | NM\_007405 | Mus musculus adenylate cyclase 6 (Adcy6), mRNA | 0.61 | 0.01626 |  | |
| A\_51\_P222153 | NM\_009419 | Mus musculus protein-tyrosine sulfotransferase 2 (Tpst2), mRNA | 0.60 | 0.00833 |  | |
| A\_51\_P259028 | NM\_025869 | Mus musculus dual specificity phosphatase 26 (putative) (Dusp26), mRNA | 0.60 | 0.04573 |  | |
| A\_51\_P139165 | NM\_025822 | Mus musculus arginine/serine-rich coiled-coil 1 (Rsrc1), mRNA | 0.60 | 0.01981 |  | |
| A\_51\_P401668 | NM\_010686 | Mus musculus lysosomal-associated protein transmembrane 5 (Laptm5), mRNA | 0.60 | 0.04558 |  | |
| A\_51\_P317695 | NM\_019510 | Mus musculus transient receptor potential cation channel, subfamily C, member 3 (Trpc3), mRNA | 0.60 | 0.01998 |  | |
| A\_51\_P216215 | NM\_007982 | Mus musculus PTK2 protein tyrosine kinase 2 (Ptk2), mRNA | 0.60 | 0.00853 |  | |
| A\_51\_P457989 | NM\_027491 | Mus musculus Ras-related GTP binding D (Rragd), mRNA | 0.60 | 0.04155 |  | |
| A\_51\_P306017 | NM\_007865 | Mus musculus delta-like 1 (Drosophila) (Dll1), mRNA | 0.60 | 0.03444 |  | |
| A\_51\_P414790 | NM\_178005 | Mus musculus leucine rich repeat transmembrane neuronal 2 (Lrrtm2), mRNA | 0.59 | 0.01871 |  | |
| A\_51\_P119776 | AK004418 | Mus musculus 18-day embryo whole body cDNA, RIKEN full-length enriched library, clone:1110069I04 product:unknown EST, f | 0.59 | 0.02855 |  | |
| A\_51\_P416689 | NM\_010162 | Mus musculus exostoses (multiple) 1 (Ext1), mRNA | 0.59 | 0.02880 |  | |
| A\_51\_P421279 | BC053094 | Mus musculus echinoderm microtubule associated protein like 1, mRNA (cDNA clone IMAGE:6400458), containing frame-shift | 0.59 | 0.03069 |  | |
| A\_51\_P331429 | NM\_177756 | Mus musculus glycosyltransferase 25 domain containing 2 (Glt25d2), mRNA | 0.59 | 0.00497 |  | |
| A\_51\_P503822 | NM\_175499 | Mus musculus SLIT and NTRK-like family, member 6 (Slitrk6), mRNA | 0.59 | 0.00577 |  | |
| A\_51\_P117739 | NM\_010216 | Mus musculus c-fos induced growth factor (Figf), mRNA | 0.59 | 0.02386 |  | |
| A\_51\_P220278 | NM\_028392 | Mus musculus protein phosphatase 2 (formerly 2A), regulatory subunit B (PR 52), beta isoform (Ppp2r2b), transcript variant 2, | 0.59 | 0.03994 |  | |
| A\_51\_P116940 | NM\_007940 | Mus musculus epoxide hydrolase 2, cytoplasmic (Ephx2), mRNA | 0.59 | 0.04386 |  | |
| A\_51\_P346803 | NM\_017380 | Mus musculus septin 9 (Sept9), mRNA | 0.59 | 0.01565 |  | |
| A\_51\_P384448 | AK041201 | Mus musculus adult male aorta and vein cDNA, RIKEN full-length enriched library, clone:A530090C09 product:MY014 PROTEI | 0.59 | 0.00888 |  | |
| A\_51\_P424168 | NM\_133217 | Mus musculus beta-carotene 9', 10'-dioxygenase 2 (Bcdo2), mRNA | 0.58 | 0.03001 |  | |
| A\_51\_P257769 | AK032954 | Mus musculus 12 days embryo male wolffian duct includes surrounding region cDNA, RIKEN full-length enriched library, clone: | 0.58 | 0.03849 |  | |
| A\_51\_P190244 | NM\_008729 | Mus musculus catenin (cadherin associated protein), delta 2 (Ctnnd2), mRNA | 0.58 | 0.02065 |  | |
| A\_51\_P384669 | NM\_133807 | Mus musculus expressed sequence AA959742 (AA959742), mRNA | 0.58 | 0.04332 |  | |
| A\_51\_P265906 | BC066035 | Mus musculus zinc finger, RAN-binding domain containing 3, mRNA (cDNA clone MGC:91303 IMAGE:6837116), complete cds | 0.58 | 0.01784 |  | |
| A\_51\_P100828 | NM\_007452 | Mus musculus peroxiredoxin 3 (Prdx3), mRNA | 0.58 | 0.00491 |  | |
| A\_51\_P386585 | NM\_153410 | Mus musculus G-protein signalling modulator 1 (AGS3-like, C. elegans) (Gpsm1), mRNA | 0.58 | 0.02359 |  | |
| A\_51\_P111164 | NM\_172612 | Mus musculus Rho family GTPase 1 (Rnd1), mRNA | 0.58 | 0.03522 |  | |
| A\_51\_P464398 | AK046394 | Mus musculus adult male corpora quadrigemina cDNA, RIKEN full-length enriched library, clone:B230379H01 product:SERINE/ | 0.58 | 0.01797 |  | |
| A\_51\_P194375 | NM\_021567 | Mus musculus poly(rC) binding protein 4 (Pcbp4), mRNA | 0.58 | 0.02263 |  | |
| A\_51\_P226791 | A\_51\_P226791 | Unknown | 0.58 | 0.00074 |  | |
| A\_51\_P471458 | NM\_020564 | Mus musculus sulfotransferase family 5A, member 1 (Sult5a1), mRNA | 0.58 | 0.00367 |  | |
| A\_51\_P131358 | NM\_009151 | Mus musculus selectin, platelet (p-selectin) ligand (Selpl), mRNA | 0.57 | 0.03475 |  | |
| A\_51\_P301508 | AK009316 | Mus musculus adult male tongue cDNA, RIKEN full-length enriched library, clone:2310012I02 product:SKI PROTO-ONCOGEN | 0.57 | 0.01100 |  | |
| A\_51\_P218975 | NM\_173417 | Mus musculus potassium voltage-gated channel, delayed-rectifier, subfamily S, member 3 (Kcns3), mRNA | 0.57 | 0.01062 |  | |
| A\_51\_P163694 | NM\_172557 | Mus musculus RUN and FYVE domain containing 1 (Rufy1), mRNA | 0.57 | 0.02225 |  | |
| A\_51\_P138548 | NM\_008253 | Mus musculus high mobility group box 3 (Hmgb3), mRNA | 0.57 | 0.00937 |  | |
| A\_51\_P160408 | NM\_178142 | Mus musculus RIKEN cDNA A830039H10 gene (A830039H10Rik), transcript variant 2, mRNA | 0.57 | 0.00669 |  | |
| A\_51\_P290059 | NM\_008083 | Mus musculus growth associated protein 43 (Gap43), mRNA | 0.57 | 0.00961 |  | |
| A\_51\_P412461 | NM\_172808 | Mus musculus RIKEN cDNA 1700112N15 gene (1700112N15Rik), mRNA | 0.57 | 0.01590 |  | |
| A\_51\_P449777 | AK077382 | Mus musculus 6 days neonate head cDNA, RIKEN full-length enriched library, clone:5430409C21 product:unknown EST, full in | 0.57 | 0.03362 |  | |
| A\_51\_P120075 | NM\_009093 | Mus musculus ribosomal protein S29 (Rps29), mRNA | 0.57 | 0.04306 |  | |
| A\_51\_P318683 | AF031816 | Mus musculus hybrid receptor gp250 precursor, mRNA, partial cds | 0.57 | 0.00647 |  | |
| A\_51\_P465042 | NM\_025882 | Mus musculus polymerase (DNA-directed), epsilon 4 (p12 subunit) (Pole4), mRNA | 0.57 | 0.00102 |  | |
| A\_51\_P370614 | NM\_009718 | Mus musculus neurogenin 2 (Neurog2), mRNA | 0.57 | 0.03986 |  | |
| A\_51\_P395470 | NM\_001001184 | Mus musculus hypothetical MGC86034 (MGC86034), mRNA | 0.57 | 0.00997 |  | |
| A\_51\_P451301 | NM\_178764 | Mus musculus RIKEN cDNA B930006L02 gene (B930006L02Rik), mRNA | 0.56 | 0.04967 |  | |
| A\_51\_P298190 | XM\_488538 | PREDICTED: SET-binding factor 2 | 0.56 | 0.03567 |  | |
| A\_51\_P171772 | NM\_021399 | Mus musculus B-cell leukemia/lymphoma 11B (Bcl11b), mRNA | 0.56 | 0.01886 |  | |
| A\_51\_P514898 | NM\_175160 | Mus musculus zinc finger, DHHC domain containing 1 (Zdhhc1), mRNA | 0.56 | 0.02879 |  | |
| A\_51\_P466478 | NM\_011213 | Mus musculus protein tyrosine phosphatase, receptor type, F (Ptprf), mRNA | 0.56 | 0.00039 |  | |
| A\_51\_P427825 | AK009836 | Mus musculus adult male tongue cDNA, RIKEN full-length enriched library, clone:2310046A06 product:hypothetical protein, full | 0.55 | 0.04726 |  | |
| A\_51\_P119857 | AK017680 | Mus musculus 8 days embryo whole body cDNA, RIKEN full-length enriched library, clone:5730461F13 product:unknown EST, | 0.55 | 0.01529 |  | |
| A\_51\_P376158 | NM\_024175 | Mus musculus ribosomal protein S23 (Rps23), mRNA | 0.55 | 0.04426 |  | |
| A\_51\_P498388 | NM\_145587 | Mus musculus SH3-binding kinase 1 (Sbk1), mRNA | 0.55 | 0.00894 |  | |
| A\_51\_P233101 | NM\_008520 | Mus musculus latent transforming growth factor beta binding protein 3 (Ltbp3), mRNA | 0.55 | 0.00418 |  | |
| A\_51\_P363519 | NM\_028596 | Mus musculus RIKEN cDNA 2410025L10 gene (2410025L10Rik), mRNA | 0.55 | 0.00902 |  | |
| A\_51\_P358152 | NM\_029100 | Mus musculus selenoprotein N, 1 (Sepn1), mRNA | 0.55 | 0.01575 |  | |
| A\_51\_P224023 | NM\_144846 | Mus musculus RIKEN cDNA 0910001A06 gene (0910001A06Rik), mRNA | 0.55 | 0.01546 |  | |
| A\_51\_P435922 | NM\_029338 | Mus musculus RIKEN cDNA 1700027N10 gene (1700027N10Rik), mRNA | 0.54 | 0.04184 |  | |
| A\_51\_P340038 | AK032598 | Mus musculus adult male olfactory brain cDNA, RIKEN full-length enriched library, clone:6430629P17 product:hypothetical Pho | 0.54 | 0.03753 |  | |
| A\_51\_P268983 | XM\_485520 | PREDICTED: similar to myomesin family, member 3 | 0.54 | 0.00158 |  | |
| A\_51\_P456857 | NM\_031177 | Mus musculus WD repeat domain 10 (Wdr10), mRNA | 0.54 | 0.01948 |  | |
| A\_51\_P108581 | TC1463173 | Q6QPD1 (Q6QPD1) E3 25.4 kDa, partial (10%) | 0.54 | 0.02945 |  | |
| A\_51\_P233982 | NM\_177089 | Mus musculus transforming, acidic coiled-coil containing protein 1 (Tacc1), transcript variant 1, mRNA | 0.54 | 0.01361 |  | |
| A\_51\_P296878 | NM\_173746 | Mus musculus hypothetical protein C130086A10 (C130086A10), mRNA | 0.54 | 0.00161 |  | |
| A\_51\_P195557 | NM\_026249 | Mus musculus RIKEN cDNA 4930429B21 gene (4930429B21Rik), mRNA | 0.54 | 0.02865 |  | |
| A\_51\_P373573 | NM\_174988 | Mus musculus cadherin 22 (Cdh22), mRNA | 0.53 | 0.01631 |  | |
| A\_51\_P428777 | NM\_030555 | Mus musculus pre-B-cell leukemia transcription factor 4 (Pbx4), transcript variant 2, mRNA | 0.53 | 0.02740 |  | |
| A\_51\_P208811 | AK030894 | Mus musculus adult male thymus cDNA, RIKEN full-length enriched library, clone:5830445K23 product:weakly similar to TES | 0.53 | 0.00591 |  | |
| A\_51\_P509997 | NM\_009943 | Mus musculus cytochrome c oxidase, subunit VI a, polypeptide 2 (Cox6a2), mRNA | 0.53 | 0.00385 |  | |
| A\_51\_P494675 | NM\_028071 | Mus musculus coactosin-like 1 (Dictyostelium) (Cotl1), mRNA | 0.53 | 0.00137 |  | |
| A\_51\_P490795 | AK031169 | Mus musculus 13 days embryo forelimb cDNA, RIKEN full-length enriched library, clone:5930420M08 product:Max dimerization | 0.53 | 0.01468 |  | |
| A\_51\_P173459 | NM\_175538 | Mus musculus RIKEN cDNA E130304F04 gene (E130304F04Rik), mRNA | 0.53 | 0.01234 |  | |
| A\_51\_P110759 | NM\_009199 | Mus musculus solute carrier family 1 (neuronal/epithelial high affinity glutamate transporter, system Xag), member 1 (Slc1a1), | 0.53 | 0.00878 |  | |
| A\_51\_P141600 | NM\_009098 | Mus musculus ribosomal protein S8 (Rps8), mRNA | 0.53 | 0.03136 |  | |
| A\_51\_P326994 | NM\_133746 | Mus musculus RIKEN cDNA 2810048G17 gene (2810048G17Rik), mRNA | 0.53 | 0.02879 |  | |
| A\_51\_P513661 | AK082224 | Mus musculus 0 day neonate cerebellum cDNA, RIKEN full-length enriched library, clone:C230026C11 product:unknown EST, f | 0.53 | 0.02817 |  | |
| A\_51\_P255853 | NM\_011527 | Mus musculus T-cell acute lymphocytic leukemia 1 (Tal1), mRNA | 0.53 | 0.01511 |  | |
| A\_51\_P310850 | BC025841 | Mus musculus cDNA clone IMAGE:5149318, partial cds. | 0.53 | 0.04124 |  | |
| A\_51\_P431491 | BC037132 | Mus musculus RAB11 family interacting protein 3 (class II), mRNA (cDNA clone MGC:47508 IMAGE:4973426), complete cds. | 0.52 | 0.02090 |  | |
| A\_51\_P159603 | NM\_207202 | Mus musculus DNA segment, Chr X, Immunex 50, expressed (DXImx50e), mRNA | 0.52 | 0.02320 |  | |
| A\_51\_P105262 | NM\_201355 | Mus musculus cDNA sequence BC047219 (BC047219), mRNA | 0.52 | 0.02554 |  | |
| A\_51\_P383194 | NM\_008804 | Mus musculus phosphodiesterase 9A (Pde9a), mRNA | 0.52 | 0.00078 |  | |
| A\_51\_P463860 | NM\_172581 | Mus musculus RIKEN cDNA 9830169C18 gene (9830169C18Rik), mRNA | 0.52 | 0.02790 |  | |
| A\_51\_P469688 | NM\_138649 | Mus musculus synaptotagmin XVII (Syt17), mRNA | 0.52 | 0.00865 |  | |
| A\_51\_P145541 | NM\_020296 | Mus musculus RNA binding motif, single stranded interacting protein 1 (Rbms1), mRNA | 0.52 | 0.01749 |  | |
| A\_51\_P139029 | NM\_023805 | Mus musculus solute carrier family 38, member 3 (Slc38a3), mRNA | 0.52 | 0.04094 |  | |
| A\_51\_P209460 | NM\_026585 | Mus musculus DNA segment, Chr 6, Wayne State University 116, expressed (D6Wsu116e), mRNA | 0.52 | 0.00011 |  | |
| A\_51\_P438235 | NM\_027591 | Mus musculus RIKEN cDNA 4921520P21 gene (4921520P21Rik), mRNA | 0.52 | 0.00184 |  | |
| A\_51\_P360840 | NM\_009569 | Mus musculus zinc finger protein, multitype 1 (Zfpm1), mRNA | 0.52 | 0.00879 |  | |
| A\_51\_P306308 | NM\_028220 | Mus musculus WD repeat domain 17 (Wdr17), mRNA | 0.52 | 0.04571 |  | |
| A\_51\_P270206 | NM\_029852 | Mus musculus RIKEN cDNA 4921537D05 gene (4921537D05Rik), mRNA | 0.52 | 0.03806 |  | |
| A\_51\_P268953 | AK035894 | Mus musculus 16 days neonate cerebellum cDNA, RIKEN full-length enriched library, clone:9630015D15 product:hypothetical | 0.51 | 0.03581 |  | |
| A\_51\_P435990 | NM\_022565 | Mus musculus N-deacetylase/N-sulfotransferase (heparin glucosaminyl) 4 (Ndst4), mRNA | 0.51 | 0.00005 |  | |
| A\_51\_P446825 | NM\_176952 | Mus musculus RIKEN cDNA 6430573F11 gene (6430573F11Rik), mRNA | 0.51 | 0.03673 |  | |
| A\_51\_P181705 | AK017277 | Mus musculus 6 days neonate head cDNA, RIKEN full-length enriched library, clone:5430405N12 product:unclassifiable, full in | 0.51 | 0.03525 |  | |
| A\_51\_P409827 | AB030187 | Mus musculus mRNA, complete cds, clone:1-73. | 0.51 | 0.03909 |  | |
| A\_51\_P116137 | NM\_008377 | Mus musculus leucine-rich repeats and immunoglobulin-like domains 1 (Lrig1), mRNA | 0.51 | 0.04030 |  | |
| A\_51\_P225056 | AK047799 | Mus musculus adult male corpus striatum cDNA, RIKEN full-length enriched library, clone:C030045O21 product:hypothetical pr | 0.51 | 0.00072 |  | |
| A\_51\_P338886 | NM\_026950 | Mus musculus OCIA domain containing 2 (Ociad2), mRNA | 0.51 | 0.00066 |  | |
| A\_51\_P352303 | NM\_011983 | Mus musculus homer homolog 2 (Drosophila) (Homer2), mRNA | 0.51 | 0.00077 |  | |
| A\_51\_P291078 | NM\_172710 | Mus musculus RIKEN cDNA 2310045A20 gene (2310045A20Rik), mRNA | 0.51 | 0.01837 |  | |
| A\_51\_P296682 | NM\_010098 | Mus musculus opsin (encephalopsin) (Opn3), mRNA | 0.51 | 0.00405 |  | |
| A\_51\_P436630 | NM\_173393 | Mus musculus cDNA sequence BC024502 (BC024502), mRNA | 0.51 | 0.01699 |  | |
| A\_51\_P133897 | AK080900 | Mus musculus 4 days neonate male adipose cDNA, RIKEN full-length enriched library, clone:B430203G01 product:NICOTINIC | 0.51 | 0.01049 |  | |
| A\_51\_P260721 | NM\_016902 | Mus musculus nephronophthisis 1 (juvenile) homolog (human) (Nphp1), mRNA | 0.50 | 0.00973 |  | |
| A\_51\_P253883 | NM\_029758 | Mus musculus DNA segment, Chr 12, ERATO Doi 553, expressed (D12Ertd553e), mRNA | 0.50 | 0.04665 |  | |
| A\_51\_P393654 | NM\_175514 | Mus musculus RIKEN cDNA D430039N05 gene (D430039N05Rik), mRNA | 0.50 | 0.01126 |  | |
| A\_51\_P509579 | NM\_028118 | Mus musculus WD repeat, SAM and U-box domain containing 1 (Wdsub1), mRNA | 0.50 | 0.03608 |  | |
| A\_51\_P438293 | BC038250 | Mus musculus, clone IMAGE:4507900, mRNA. | 0.50 | 0.00904 |  | |
| A\_51\_P115248 | NM\_175238 | Mus musculus Rap1 interacting factor 1 homolog (yeast) (Rif1), mRNA | 0.50 | 0.04677 |  | |
| A\_51\_P521304 | NM\_026167 | Mus musculus kelch-like 13 (Drosophila) (Klhl13), mRNA | 0.50 | 0.02656 |  | |
| A\_51\_P221997 | NM\_207237 | Mus musculus mannosidase, alpha, class 1C, member 1 (Man1c1), mRNA | 0.50 | 0.03195 |  | |
| A\_51\_P157255 | NM\_008304 | Mus musculus syndecan 2 (Sdc2), mRNA | 0.49 | 0.00863 |  | |
| A\_51\_P344661 | BC059060 | Mus musculus RIKEN cDNA 4933409K07 gene, mRNA (cDNA clone MGC:69869 IMAGE:6822098), complete cds. | 0.49 | 0.01240 |  | |
| A\_51\_P376454 | BI646741 | BI646741 603279769F1 NCI\_CGAP\_Mam3 Mus musculus cDNA clone IMAGE:5320025 5', mRNA sequence | 0.49 | 0.03495 |  | |
| A\_51\_P127035 | NM\_026486 | Mus musculus RIKEN cDNA 4432405B04 gene (4432405B04Rik), mRNA | 0.49 | 0.00299 |  | |
| A\_51\_P193034 | BU534286 | BU534286 AGENCOURT\_10198645 NIH\_MGC\_143 Mus musculus cDNA clone IMAGE:6561559 5', mRNA sequence | 0.49 | 0.01780 |  | |
| A\_51\_P100289 | NM\_020493 | Mus musculus serum response factor (Srf), mRNA | 0.49 | 0.01248 |  | |
| A\_51\_P298259 | NM\_025634 | Mus musculus RIKEN cDNA 2310042E22 gene (2310042E22Rik), mRNA | 0.49 | 0.02580 |  | |
| A\_51\_P338317 | NM\_145491 | Mus musculus ras homolog gene family, member Q (Rhoq), mRNA | 0.49 | 0.01085 |  | |
| A\_51\_P113195 | NM\_178924 | Mus musculus uroplakin 1B (Upk1b), mRNA | 0.49 | 0.02185 |  | |
| A\_51\_P120017 | BC057029 | Mus musculus phosphodiesterase 2A, cGMP-stimulated, mRNA (cDNA clone IMAGE:6832606), containing frame-shift errors | 0.49 | 0.00658 |  | |
| A\_51\_P303056 | NM\_009623 | Mus musculus adenylate cyclase 8 (Adcy8), mRNA | 0.48 | 0.00030 |  | |
| A\_51\_P217899 | NM\_146163 | Mus musculus myosin 1H (Myo1h), mRNA | 0.48 | 0.01480 |  | |
| A\_51\_P450924 | NM\_025882 | Mus musculus polymerase (DNA-directed), epsilon 4 (p12 subunit) (Pole4), mRNA | 0.48 | 0.00628 |  | |
| A\_51\_P367477 | AK045510 | Mus musculus adult male corpora quadrigemina cDNA, RIKEN full-length enriched library, clone:B230207O18 product:unknown | 0.48 | 0.03744 |  | |
| A\_51\_P115471 | AK046043 | Mus musculus adult male corpora quadrigemina cDNA, RIKEN full-length enriched library, clone:B230337H21 product:inferred: | 0.47 | 0.00007 |  | |
| A\_51\_P187665 | NM\_028904 | Mus musculus RIKEN cDNA 4932432N11 gene (4932432N11Rik), mRNA | 0.47 | 0.00012 |  | |
| A\_51\_P313921 | NM\_013696 | Mus musculus thyrotropin releasing hormone receptor (Trhr), mRNA | 0.47 | 0.01625 |  | |
| A\_51\_P179565 | NM\_023450 | Mus musculus RIKEN cDNA 2010204K13 gene (2010204K13Rik), mRNA | 0.47 | 0.00066 |  | |
| A\_51\_P422124 | NM\_053090 | Mus musculus down-regulated by Ctnnb1, a (Drctnnb1a), mRNA | 0.47 | 0.00178 |  | |
| A\_51\_P189251 | NM\_013823 | Mus musculus klotho (Kl), mRNA | 0.47 | 0.02401 |  | |
| A\_51\_P332140 | NM\_009900 | Mus musculus chloride channel 2 (Clcn2), mRNA | 0.47 | 0.02611 |  | |
| A\_51\_P206835 | NM\_010687 | Mus musculus like-glycosyltransferase (Large), mRNA | 0.47 | 0.01108 |  | |
| A\_51\_P217648 | NM\_019397 | Mus musculus EGF-like-domain, multiple 6 (Egfl6), mRNA | 0.47 | 0.00313 |  | |
| A\_51\_P452528 | BC035277 | Mus musculus DNA segment, Chr 1, ERATO Doi 471, expressed, mRNA (cDNA clone IMAGE:5064213), partial cds | 0.46 | 0.02196 |  | |
| A\_51\_P482618 | NM\_019789 | Mus musculus calsenilin, presenilin binding protein, EF hand transcription factor (Csen), mRNA | 0.46 | 0.00454 |  | |
| A\_51\_P227392 | NM\_133955 | Mus musculus ras homolog gene family, member U (Rhou), mRNA | 0.46 | 0.00122 |  | |
| A\_51\_P495986 | NM\_025508 | Mus musculus guanosine monophosphate reductase (Gmpr), mRNA | 0.46 | 0.00197 |  | |
| A\_51\_P509137 | NM\_007545 | Mus musculus BH3 interacting (with BCL2 family) domain, apoptosis agonist (Bid3), mRNA | 0.46 | 0.00376 |  | |
| A\_51\_P107991 | NM\_009550 | Mus musculus zinc finger protein 2 (Zfp2), transcript variant 1, mRNA | 0.46 | 0.00252 |  | |
| A\_51\_P204329 | AK049846 | Mus musculus adult male hippocampus cDNA, RIKEN full-length enriched library, clone:C630003C14 product:similar to CDNA | 0.46 | 0.00664 |  | |
| A\_51\_P222475 | NM\_053252 | Mus musculus tangerin (LOC114601), mRNA | 0.46 | 0.00388 |  | |
| A\_51\_P108757 | NM\_024243 | Mus musculus fucosidase, alpha-L- 1, tissue (Fuca1), mRNA | 0.46 | 0.00100 |  | |
| A\_51\_P397222 | A\_51\_P397222 | Unknown | 0.46 | 0.01280 |  | |
| A\_51\_P206153 | D13903 | Mus musculus mRNA for MPTPdelta, complete cds. | 0.45 | 0.00036 |  | |
| A\_51\_P416126 | XM\_484041 | PREDICTED: chromodomain helicase DNA binding protein 3 | 0.45 | 0.03840 |  | |
| A\_51\_P365153 | NM\_010474 | Mus musculus heparan sulfate (glucosamine) 3-O-sulfotransferase 1 (Hs3st1), mRNA | 0.45 | 0.03353 |  | |
| A\_51\_P226472 | AK046082 | Mus musculus adult male corpora quadrigemina cDNA, RIKEN full-length enriched library, clone:B230340M18 product:unclassif | 0.45 | 0.02343 |  | |
| A\_51\_P170816 | NM\_019867 | Mus musculus neuronal guanine nucleotide exchange factor (Ngef), mRNA | 0.45 | 0.00550 |  | |
| A\_51\_P442366 | AK083254 | Mus musculus adult male hippocampus cDNA, RIKEN full-length enriched library, clone:C630031G11 product:diaphanous hom | 0.45 | 0.01046 |  | |
| A\_51\_P348433 | NM\_013832 | Mus musculus RAS protein activator like 1 (GAP1 like) (Rasal1), mRNA | 0.45 | 0.01127 |  | |
| A\_51\_P480390 | NM\_008579 | Mus musculus meiosis expressed gene 1 (Meig1), mRNA | 0.45 | 0.00177 |  | |
| A\_51\_P283774 | NM\_028199 | Mus musculus plexin domain containing 1 (Plxdc1), mRNA | 0.45 | 0.00102 |  | |
| A\_51\_P513000 | NM\_030699 | Mus musculus netrin G1 (Ntng1), mRNA | 0.45 | 0.00712 |  | |
| A\_51\_P479590 | BC037006 | ENSMUST00000018993 | 0.45 | 0.00217 |  | |
| A\_51\_P428661 | NM\_009707 | Mus musculus Rho GTPase activating protein 6 (Arhgap6), mRNA | 0.45 | 0.01511 |  | |
| A\_51\_P336952 | NM\_030004 | Mus musculus crystallin, lamda 1 (Cryl1), mRNA | 0.45 | 0.00010 |  | |
| A\_51\_P146970 | NM\_145831 | Mus musculus doublesex and mab-3 related transcription factor 2 (Dmrt2), mRNA | 0.45 | 0.01631 |  | |
| A\_51\_P140182 | NM\_009656 | Mus musculus aldehyde dehydrogenase 2, mitochondrial (Aldh2), mRNA | 0.45 | 0.00388 |  | |
| A\_51\_P190198 | NM\_027438 | Mus musculus paraneoplastic antigen MA1 (Pnma1), mRNA | 0.44 | 0.00182 |  | |
| A\_51\_P103650 | NM\_053100 | Mus musculus tripartite motif protein 8 (Trim8), mRNA | 0.44 | 0.00189 |  | |
| A\_51\_P221100 | NM\_198214 | Mus musculus syntaphilin (Snph), mRNA | 0.44 | 0.03066 |  | |
| A\_51\_P416673 | NM\_008473 | Mus musculus keratin complex 2, basic, gene 1 (Krt2-1), mRNA | 0.44 | 0.00161 |  | |
| A\_51\_P144348 | A\_51\_P144348 | Unknown | 0.44 | 0.00980 |  | |
| A\_51\_P293059 | NM\_133722 | Mus musculus RIKEN cDNA 2210412D01 gene (2210412D01Rik), mRNA | 0.44 | 0.01649 |  | |
| A\_51\_P217509 | NM\_028709 | Mus musculus BTB (POZ) domain containing 11 (Btbd11), transcript variant 1, mRNA | 0.44 | 0.03744 |  | |
| A\_51\_P350252 | NM\_010179 | Mus musculus fat tumor suppressor homolog (Drosophila) (Fath), mRNA | 0.43 | 0.01780 |  | |
| A\_51\_P340601 | NM\_009770 | Mus musculus B-cell translocation gene 3 (Btg3), mRNA | 0.43 | 0.02409 |  | |
| A\_51\_P370510 | NM\_145450 | Mus musculus cDNA sequence BC022687 (BC022687), mRNA | 0.43 | 0.02518 |  | |
| A\_51\_P260312 | NM\_172753 | Mus musculus RIKEN cDNA 4732435N03 gene (4732435N03Rik), mRNA | 0.43 | 0.01169 |  | |
| A\_51\_P141580 | NM\_199021 | Mus musculus dipeptidylpeptidase 10 (Dpp10), mRNA | 0.43 | 0.02522 |  | |
| A\_51\_P148355 | NM\_175433 | Mus musculus RIKEN cDNA 5430400N05 gene (5430400N05Rik), mRNA | 0.43 | 0.00367 |  | |
| A\_51\_P380337 | NM\_027871 | Mus musculus Rho guanine nucleotide exchange factor (GEF) 3 (Arhgef3), mRNA | 0.43 | 0.00612 |  | |
| A\_51\_P263239 | NM\_008748 | Mus musculus dual specificity phosphatase 8 (Dusp8), mRNA | 0.43 | 0.03753 |  | |
| A\_51\_P202050 | NM\_008052 | Mus musculus deltex 1 homolog (Drosophila) (Dtx1), mRNA | 0.43 | 0.01220 |  | |
| A\_51\_P403704 | AK011787 | Mus musculus 10 days embryo whole body cDNA, RIKEN full-length enriched library, clone:2610100L16 product:unknown EST | 0.43 | 0.00012 |  | |
| A\_51\_P221677 | NM\_178110 | Mus musculus tripartite motif-containing 62 (Trim62), mRNA | 0.43 | 0.00664 |  | |
| A\_51\_P400160 | AK013705 | Mus musculus adult male hippocampus cDNA, RIKEN full-length enriched library, clone:2900056M07 product:hypothetical prot | 0.42 | 0.00502 |  | |
| A\_51\_P435968 | NM\_134131 | Mus musculus tumor necrosis factor, alpha-induced protein 8 (Tnfaip8), mRNA | 0.42 | 0.02817 |  | |
| A\_51\_P423000 | NM\_007417 | Mus musculus adrenergic receptor, alpha 2a (Adra2a), mRNA | 0.42 | 0.00075 |  | |
| A\_51\_P346747 | NM\_013919 | Mus musculus ubiquitin specific protease 21 (Usp21), mRNA | 0.42 | 0.00147 |  | |
| A\_51\_P415775 | NM\_198967 | Mus musculus cDNA sequence BC023818 (BC023818), mRNA | 0.42 | 0.01803 |  | |
| A\_51\_P425165 | A\_51\_P425165 | Unknown | 0.42 | 0.00021 |  | |
| A\_51\_P331805 | NM\_146188 | Mus musculus potassium channel tetramerisation domain containing 15 (Kctd15), mRNA | 0.42 | 0.00076 |  | |
| A\_51\_P406527 | NM\_019697 | Mus musculus potassium voltage-gated channel, Shal-related family, member 2 (Kcnd2), mRNA | 0.42 | 0.00623 |  | |
| A\_51\_P357533 | BC080307 | Mus musculus transmembrane protein 44, mRNA (cDNA clone MGC:90554 IMAGE:5721210), complete cds. | 0.42 | 0.00156 |  | |
| A\_51\_P411182 | NM\_144828 | Mus musculus protein phosphatase 1, regulatory (inhibitor) subunit 1B (Ppp1r1b), mRNA | 0.42 | 0.00859 |  | |
| A\_51\_P366306 | NM\_028325 | Mus musculus zinc finger, CCHC domain containing 12 (Zcchc12), mRNA | 0.41 | 0.00374 |  | |
| A\_51\_P424470 | NM\_028408 | Mus musculus cornichon homolog 3 (Drosophila) (Cnih3), mRNA | 0.41 | 0.01339 |  | |
| A\_51\_P397363 | NM\_009196 | Mus musculus solute carrier family 16 (monocarboxylic acid transporters), member 1 (Slc16a1), mRNA | 0.41 | 0.00105 |  | |
| A\_51\_P474431 | NM\_023117 | Mus musculus cell division cycle 25 homolog B (S. cerevisiae) (Cdc25b), mRNA | 0.41 | 0.00457 |  | |
| A\_51\_P354526 | NM\_025912 | Mus musculus RIKEN cDNA 2010011I20 gene (2010011I20Rik), mRNA | 0.41 | 0.01886 |  | |
| A\_51\_P415806 | NM\_026954 | Mus musculus tumor suppressor candidate 1 (Tusc1), mRNA | 0.41 | 0.00240 |  | |
| A\_51\_P104012 | AK009669 | Mus musculus adult male tongue cDNA, RIKEN full-length enriched library, clone:2310037P21 product:BRAIN SPECIFIC BINDI | 0.41 | 0.03350 |  | |
| A\_51\_P137094 | NM\_015772 | Mus musculus sal-like 2 (Drosophila) (Sall2), mRNA | 0.40 | 0.00599 |  | |
| A\_51\_P167535 | NM\_010174 | Mus musculus fatty acid binding protein 3, muscle and heart (Fabp3), mRNA | 0.40 | 0.00057 |  | |
| A\_51\_P202033 | NM\_026582 | Mus musculus RIKEN cDNA 5031439A09 gene (5031439A09Rik), mRNA | 0.40 | 0.02323 |  | |
| A\_51\_P416974 | NM\_016768 | Mus musculus pre B-cell leukemia transcription factor 3 (Pbx3), mRNA | 0.40 | 0.00106 |  | |
| A\_51\_P396284 | BC030435 | Mus musculus chromodomain helicase DNA binding protein 3, mRNA (cDNA clone MGC:40857 IMAGE:5369285), complete cd | 0.40 | 0.02975 |  | |
| A\_51\_P444633 | NM\_029653 | Mus musculus death associated protein kinase 1 (Dapk1), mRNA | 0.40 | 0.02294 |  | |
| A\_51\_P342871 | NM\_009112 | Mus musculus S100 calcium binding protein A10 (calpactin) (S100a10), mRNA | 0.40 | 0.02329 |  | |
| A\_51\_P297441 | NM\_054066 | Mus musculus phospholipase C, zeta 1 (Plcz1), mRNA | 0.40 | 0.00801 |  | |
| A\_51\_P410430 | AK006449 | Mus musculus adult male testis cDNA, RIKEN full-length enriched library, clone:1700028D05 product:nasopharyngeal epitheliu | 0.40 | 0.00244 |  | |
| A\_51\_P269375 | NM\_031158 | Mus musculus ankyrin 1, erythroid (Ank1), mRNA | 0.39 | 0.00036 |  | |
| A\_51\_P329842 | NM\_027998 | Mus musculus claudin 23 (Cldn23), mRNA | 0.39 | 0.00869 |  | |
| A\_51\_P215077 | NM\_025569 | Mus musculus microsomal glutathione S-transferase 3 (Mgst3), mRNA | 0.39 | 0.00005 |  | |
| A\_51\_P432180 | NM\_134038 | Mus musculus solute carrier family 16 (monocarboxylic acid transporters), member 6 (Slc16a6), transcript variant 2, mRNA | 0.39 | 0.00078 |  | |
| A\_51\_P267494 | NM\_026514 | Mus musculus CDC42 effector protein (Rho GTPase binding) 3 (Cdc42ep3), mRNA | 0.39 | 0.03525 |  | |
| A\_51\_P516861 | AK122234 | Mus musculus mRNA for mKIAA0277 protein | 0.39 | 0.03285 |  | |
| A\_51\_P348665 | NM\_016894 | Mus musculus receptor (calcitonin) activity modifying protein 1 (Ramp1), mRNA | 0.38 | 0.00116 |  | |
| A\_51\_P236270 | NM\_009183 | Mus musculus ST8 alpha-N-acetyl-neuraminide alpha-2,8-sialyltransferase 4 (St8sia4), mRNA | 0.38 | 0.00664 |  | |
| A\_51\_P402575 | NM\_172963 | Mus musculus RIKEN cDNA 1110012J17 gene (1110012J17Rik), mRNA | 0.38 | 0.00284 |  | |
| A\_51\_P478061 | NM\_207676 | Mus musculus immunoglobulin superfamily, member 4A (Igsf4a), transcript variant 2, mRNA | 0.38 | 0.00110 |  | |
| A\_51\_P327016 | NM\_016744 | Mus musculus phosphodiesterase 1A, calmodulin-dependent (Pde1a), mRNA | 0.38 | 0.02147 |  | |
| A\_51\_P410361 | AK030326 | Mus musculus 11 days pregnant adult female ovary and uterus cDNA, RIKEN full-length enriched library, clone:5031438B01 pr | 0.38 | 0.02182 |  | |
| A\_51\_P365369 | NM\_011261 | Mus musculus reelin (Reln), mRNA | 0.38 | 0.03589 |  | |
| A\_51\_P286378 | AK004276 | Mus musculus 18-day embryo whole body cDNA, RIKEN full-length enriched library, clone:1110056P05 product:unclassifiable, | 0.38 | 0.01418 |  | |
| A\_51\_P212592 | NM\_173749 | Mus musculus RIKEN cDNA E430002G05 gene (E430002G05Rik), mRNA | 0.37 | 0.03806 |  | |
| A\_51\_P504863 | NM\_008795 | Mus musculus PCTAIRE-motif protein kinase 3 (Pctk3), mRNA | 0.37 | 0.00633 |  | |
| A\_51\_P439842 | NM\_054077 | Mus musculus proline arginine-rich end leucine-rich repeat (Prelp), mRNA | 0.37 | 0.00569 |  | |
| A\_51\_P471648 | AK006215 | Mus musculus adult male testis cDNA, RIKEN full-length enriched library, clone:1700021K10 product:unknown EST, full insert | 0.37 | 0.00051 |  | |
| A\_51\_P367977 | AK053854 | Mus musculus 0 day neonate eyeball cDNA, RIKEN full-length enriched library, clone:E130314P20 product:unknown EST, full i | 0.37 | 0.01299 |  | |
| A\_51\_P425815 | AK043585 | Mus musculus 10 days neonate cortex cDNA, RIKEN full-length enriched library, clone:A830010I12 product:unclassifiable, full i | 0.37 | 0.00075 |  | |
| A\_51\_P218895 | NM\_008064 | Mus musculus glucosidase, alpha, acid (Gaa), mRNA | 0.37 | 0.00282 |  | |
| A\_51\_P207183 | NM\_178675 | Mus musculus solute carrier family 35, member F1 (Slc35f1), mRNA | 0.37 | 0.00602 |  | |
| A\_51\_P180492 | NM\_016974 | Mus musculus D site albumin promoter binding protein (Dbp), mRNA | 0.37 | 0.00125 |  | |
| A\_51\_P219444 | NM\_013880 | Mus musculus phospholipase C-like 2 (Plcl2), mRNA | 0.37 | 0.00037 |  | |
| A\_51\_P253824 | AK008211 | Mus musculus adult male small intestine cDNA, RIKEN full-length enriched library, clone:2010012L10 product:microsomal glut | 0.37 | 0.00010 |  | |
| A\_51\_P173735 | AK017170 | Mus musculus 11 days pregnant adult female ovary and uterus cDNA, RIKEN full-length enriched library, clone:5033414A21 pr | 0.37 | 0.01524 |  | |
| A\_51\_P448178 | NM\_175519 | Mus musculus potassium channel tetramerisation domain containing 8 (Kctd8), mRNA | 0.36 | 0.01795 |  | |
| A\_51\_P322877 | NM\_133816 | Mus musculus SH3-domain binding protein 4 (Sh3bp4), mRNA | 0.36 | 0.00221 |  | |
| A\_51\_P114693 | NM\_145562 | Mus musculus RIKEN cDNA 9130213B05 gene (9130213B05Rik), mRNA | 0.35 | 0.00944 |  | |
| A\_51\_P288419 | TC1448320 | Unknown | 0.35 | 0.00498 |  | |
| A\_51\_P333839 | NM\_019971 | Mus musculus platelet-derived growth factor, C polypeptide (Pdgfc), mRNA | 0.35 | 0.00007 |  | |
| A\_51\_P199407 | NM\_021371 | Mus musculus calneuron 1 (Caln1), mRNA | 0.35 | 0.01981 |  | |
| A\_51\_P293812 | AK002458 | Mus musculus adult male kidney cDNA, RIKEN full-length enriched library, clone:0610010D24 product:hypothetical protein, full | 0.35 | 0.00628 |  | |
| A\_51\_P355906 | NM\_021099 | Mus musculus kit oncogene (Kit), mRNA | 0.34 | 0.04125 |  | |
| A\_51\_P371174 | NM\_013863 | Mus musculus Bcl2-associated athanogene 3 (Bag3), mRNA | 0.34 | 0.00110 |  | |
| A\_51\_P264676 | AK083198 | Mus musculus adult male hippocampus cDNA, RIKEN full-length enriched library, clone:C630026G18 product:unknown EST, fu | 0.34 | 0.02357 |  | |
| A\_51\_P465082 | NM\_172913 | Mus musculus trinucleotide repeat containing 9 (Tnrc9), mRNA | 0.33 | 0.01632 |  | |
| A\_51\_P256246 | NM\_025359 | Mus musculus tetraspanin 13 (Tspan13), mRNA | 0.33 | 0.02357 |  | |
| A\_51\_P380750 | NM\_009824 | Mus musculus core-binding factor, runt domain, alpha subunit 2, translocated to, 3 homolog (human) (Cbfa2t3h), mRNA | 0.33 | 0.02337 |  | |
| A\_51\_P468231 | AK003303 | Mus musculus 18-day embryo whole body cDNA, RIKEN full-length enriched library, clone:1110002J03 product:unknown EST, f | 0.33 | 0.00314 |  | |
| A\_51\_P476538 | AK077391 | Mus musculus 6 days neonate head cDNA, RIKEN full-length enriched library, clone:5430417L22 product:unknown EST, full in | 0.33 | 0.00147 |  | |
| A\_51\_P520019 | NM\_011799 | Mus musculus cell division cycle 6 homolog (S. cerevisiae) (Cdc6), transcript variant 1, mRNA | 0.33 | 0.00003 |  | |
| A\_51\_P171437 | NM\_010930 | Mus musculus nephroblastoma overexpressed gene (Nov), mRNA | 0.33 | 0.00036 |  | |
| A\_51\_P247184 | AK046712 | Mus musculus 4 days neonate male adipose cDNA, RIKEN full-length enriched library, clone:B430320C24 product:weakly simil | 0.33 | 0.01877 |  | |
| A\_51\_P334876 | NM\_007384 | Mus musculus amiloride-sensitive cation channel 1, neuronal (degenerin) (Accn1), mRNA | 0.33 | 0.00048 |  | |
| A\_51\_P361150 | AY304481 | Mus musculus Purkinje cell protein 4-like 1 mRNA, complete cds. | 0.33 | 0.00022 |  | |
| A\_51\_P131744 | AK045478 | Mus musculus adult male corpora quadrigemina cDNA, RIKEN full-length enriched library, clone:B230206C19 product:unknown | 0.32 | 0.00173 |  | |
| A\_51\_P475785 | NM\_207264 | Mus musculus cDNA sequence BC052040 (BC052040), mRNA | 0.32 | 0.00024 |  | |
| A\_51\_P234843 | AK005609 | Mus musculus adult male testis cDNA, RIKEN full-length enriched library, clone:1700001L19 product:hypothetical protein, full i | 0.32 | 0.00196 |  | |
| A\_51\_P324303 | NM\_153789 | Mus musculus myosin regulatory light chain interacting protein (Mylip), mRNA | 0.32 | 0.00054 |  | |
| A\_51\_P326191 | AY862185 | Mus musculus serine proteinase inhibitor 2A mRNA, complete cds | 0.31 | 0.00282 |  | |
| A\_51\_P516148 | NM\_016984 | Mus musculus transient receptor potential cation channel, subfamily C, member 4 (Trpc4), mRNA | 0.31 | 0.01780 |  | |
| A\_51\_P480679 | AK052495 | Mus musculus 13 days embryo lung cDNA, RIKEN full-length enriched library, clone:D430036J16 product:unknown EST, full in | 0.31 | 0.00111 |  | |
| A\_51\_P262759 | NM\_007631 | Mus musculus cyclin D1 (Ccnd1), mRNA | 0.31 | 0.00230 |  | |
| A\_51\_P103819 | NM\_144551 | Mus musculus tribbles homolog 2 (Drosophila) (Trib2), mRNA | 0.31 | 0.03806 |  | |
| A\_51\_P406583 | NM\_181072 | Mus musculus myosin IE (Myo1e), mRNA | 0.31 | 0.00826 |  | |
| A\_51\_P114634 | NM\_173405 | Mus musculus RIKEN cDNA 6530401C20 gene (6530401C20Rik), mRNA | 0.30 | 0.00004 |  | |
| A\_51\_P309854 | NM\_080465 | Mus musculus potassium intermediate/small conductance calcium-activated channel, subfamily N, member 2 (Kcnn2), mRNA | 0.30 | 0.00213 |  | |
| A\_51\_P515046 | A\_51\_P515046 | Unknown | 0.30 | 0.00739 |  | |
| A\_51\_P112500 | A\_51\_P112500 | Unknown | 0.30 | 0.00020 |  | |
| A\_51\_P493671 | XM\_112440 | PREDICTED: heparan sulfate D-glucosaminyl 3-O-sulfotransferase 2 | 0.30 | 0.00036 |  | |
| A\_51\_P333622 | BC029089 | Mus musculus RIKEN cDNA 2010300C02 gene, mRNA (cDNA clone MGC:27999 IMAGE:3599818), complete cds. | 0.30 | 0.01507 |  | |
| A\_51\_P374572 | NM\_008344 | Mus musculus insulin-like growth factor binding protein 6 (Igfbp6), mRNA | 0.30 | 0.00822 |  | |
| A\_51\_P222633 | NM\_008067 | Mus musculus gamma-aminobutyric acid (GABA-A) receptor, subunit alpha 3 (Gabra3), mRNA | 0.30 | 0.00471 |  | |
| A\_51\_P425402 | NM\_021286 | Mus musculus seizure related gene 6 (Sez6), mRNA | 0.30 | 0.00114 |  | |
| A\_51\_P103237 | NM\_015755 | Mus musculus hormonally upregulated Neu-associated kinase (Hunk), mRNA | 0.30 | 0.01529 |  | |
| A\_51\_P515532 | NM\_029881 | Mus musculus RIKEN cDNA C030003D03 gene (C030003D03Rik), mRNA | 0.29 | 0.00947 |  | |
| A\_51\_P202911 | NM\_144882 | Mus musculus RIKEN cDNA 2810022L02 gene (2810022L02Rik), mRNA | 0.29 | 0.00060 |  | |
| A\_51\_P366344 | NM\_009365 | Mus musculus transforming growth factor beta 1 induced transcript 1 (Tgfb1i1), mRNA | 0.29 | 0.00050 |  | |
| A\_51\_P290576 | NM\_152804 | Mus musculus polo-like kinase 2 (Drosophila) (Plk2), mRNA | 0.29 | 0.00745 |  | |
| A\_51\_P191439 | NM\_178927 | Mus musculus expressed sequence AV344025 (AV344025), mRNA | 0.29 | 0.00822 |  | |
| A\_51\_P410260 | NM\_008846 | Mus musculus phosphatidylinositol-4-phosphate 5-kinase, type 1 alpha (Pip5k1a), mRNA | 0.29 | 0.00173 |  | |
| A\_51\_P178081 | NM\_009049 | Mus musculus regulated endocrine-specific protein 18 (Resp18), mRNA | 0.29 | 0.00069 |  | |
| A\_51\_P306183 | NM\_010757 | Mus musculus v-maf musculoaponeurotic fibrosarcoma oncogene family, protein K (avian) (Mafk), mRNA | 0.29 | 0.00009 |  | |
| A\_51\_P158018 | NM\_026279 | Mus musculus RIKEN cDNA 2310026E23 gene (2310026E23Rik), mRNA | 0.28 | 0.00128 |  | |
| A\_51\_P206489 | NM\_080433 | Mus musculus zinc finger protein 312 (Zfp312), mRNA | 0.28 | 0.01242 |  | |
| A\_51\_P383524 | NM\_181728 | Mus musculus ADP-ribosyltransferase 3 (Art3), mRNA | 0.28 | 0.00009 |  | |
| A\_51\_P239654 | NM\_010444 | Mus musculus nuclear receptor subfamily 4, group A, member 1 (Nr4a1), mRNA | 0.28 | 0.00886 |  | |
| A\_51\_P442704 | NM\_007873 | Mus musculus double C2, beta (Doc2b), mRNA | 0.28 | 0.00196 |  | |
| A\_51\_P168905 | NM\_015777 | Mus musculus immunoglobulin (CD79A) binding protein 1b (Igbp1b), mRNA | 0.28 | 0.00009 |  | |
| A\_51\_P416891 | AK013906 | Mus musculus 12 days embryo head cDNA, RIKEN full-length enriched library, clone:3021401C12 product:unknown EST, full in | 0.28 | 0.00106 |  | |
| A\_51\_P344113 | NM\_053202 | Mus musculus forkhead box P1 (Foxp1), mRNA | 0.28 | 0.00664 |  | |
| A\_51\_P263288 | NM\_198306 | Mus musculus UDP-N-acetyl-alpha-D-galactosamine:polypeptide N-acetylgalactosaminyltransferase 9 (Galnt9), mRNA | 0.28 | 0.00009 |  | |
| A\_51\_P434567 | AK046802 | Mus musculus 10 days neonate medulla oblongata cDNA, RIKEN full-length enriched library, clone:B830011M21 product:ALPH | 0.27 | 0.03522 |  | |
| A\_51\_P111049 | NM\_178711 | Mus musculus phospholipid scramblase 4 (Plscr4), mRNA | 0.27 | 0.00028 |  | |
| A\_51\_P494293 | AK020268 | Mus musculus adult male cecum cDNA, RIKEN full-length enriched library, clone:9130024F11 product:hypothetical protein, full | 0.27 | 0.00143 |  | |
| A\_51\_P264495 | NM\_018870 | Mus musculus phosphoglycerate mutase 2 (Pgam2), mRNA | 0.27 | 0.00020 |  | |
| A\_51\_P278653 | NM\_023396 | Mus musculus reprimo, TP53 dependent G2 arrest mediator candidate (Rprm), mRNA | 0.27 | 0.01631 |  | |
| A\_51\_P204740 | NM\_133654 | Mus musculus CD34 antigen (Cd34), mRNA | 0.27 | 0.00658 |  | |
| A\_51\_P265778 | AK019528 | Mus musculus 0 day neonate head cDNA, RIKEN full-length enriched library, clone:4833446K15 product:hypothetical protein, f | 0.27 | 0.00840 |  | |
| A\_51\_P347673 | AK007011 | Mus musculus adult male testis cDNA, RIKEN full-length enriched library, clone:1700085N21 product:unknown EST, full insert | 0.27 | 0.00057 |  | |
| A\_51\_P433026 | BC052412 | Mus musculus RIKEN cDNA 4932443D16 gene, mRNA (cDNA clone MGC:64622 IMAGE:5720146), complete cds. | 0.26 | 0.00042 |  | |
| A\_51\_P162437 | XM\_125706 | PREDICTED: breakpoint cluster region homolog | 0.26 | 0.00090 |  | |
| A\_51\_P273538 | NM\_027954 | Mus musculus RIKEN cDNA 1700013H19 gene (1700013H19Rik), mRNA | 0.26 | 0.00018 |  | |
| A\_51\_P143162 | NM\_080728 | Mus musculus myosin, heavy polypeptide 7, cardiac muscle, beta (Myh7), mRNA | 0.26 | 0.00528 |  | |
| A\_51\_P427080 | NM\_001004364 | Mus musculus development and differentiation enhancing factor 2 (Ddef2), mRNA | 0.26 | 0.02256 |  | |
| A\_51\_P513311 | NM\_009107 | Mus musculus retinoid X receptor gamma (Rxrg), mRNA | 0.26 | 0.00502 |  | |
| A\_51\_P504915 | NM\_173411 | Mus musculus cDNA sequence BC030500 (BC030500), mRNA | 0.25 | 0.00180 |  | |
| A\_51\_P247014 | NM\_010250 | Mus musculus gamma-aminobutyric acid (GABA-A) receptor, subunit alpha 1 (Gabra1), mRNA | 0.25 | 0.00251 |  | |
| A\_51\_P287418 | NM\_008548 | Mus musculus mannosidase 1, alpha (Man1a), mRNA | 0.25 | 0.00058 |  | |
| A\_51\_P340987 | NM\_178888 | Mus musculus GTPase activating RANGAP domain-like 3 (Garnl3), mRNA | 0.25 | 0.00000 |  | |
| A\_51\_P181341 | NM\_054095 | Mus musculus EF hand calcium binding protein 2 (Efcbp2), mRNA | 0.25 | 0.00022 |  | |
| A\_51\_P453817 | AK082735 | Mus musculus 0 day neonate cerebellum cDNA, RIKEN full-length enriched library, clone:C230098O21 product:unknown EST, f | 0.24 | 0.00010 |  | |
| A\_51\_P405606 | NM\_010884 | Mus musculus N-myc downstream regulated gene 1 (Ndrg1), mRNA | 0.24 | 0.00004 |  | |
| A\_51\_P314397 | NM\_024223 | Mus musculus cysteine rich protein 2 (Crip2), mRNA | 0.23 | 0.00019 |  | |
| A\_51\_P327451 | NM\_009653 | Mus musculus aminolevulinic acid synthase 2, erythroid (Alas2), mRNA | 0.23 | 0.02657 |  | |
| A\_51\_P388517 | NM\_010698 | Mus musculus LIM domain binding 2 (Ldb2), mRNA | 0.23 | 0.00166 |  | |
| A\_51\_P268496 | NM\_053195 | Mus musculus solute carrier family 24 (sodium/potassium/calcium exchanger), member 3 (Slc24a3), mRNA | 0.23 | 0.00242 |  | |
| A\_51\_P262489 | NM\_009215 | Mus musculus somatostatin (Sst), mRNA | 0.23 | 0.03872 |  | |
| A\_51\_P253964 | NM\_178399 | Mus musculus RIKEN cDNA 3110035E14 gene (3110035E14Rik), mRNA | 0.23 | 0.00178 |  | |
| A\_51\_P272993 | NM\_172290 | Mus musculus neurotrimin (Hnt), mRNA | 0.23 | 0.00002 |  | |
| A\_51\_P422685 | NM\_177086 | Mus musculus zinc finger, matrin type 4 (Zmat4), mRNA | 0.22 | 0.00146 |  | |
| A\_51\_P466910 | NM\_009783 | Mus musculus calcium channel, voltage-dependent, T type, alpha 1G subunit (Cacna1g), mRNA | 0.22 | 0.00027 |  | |
| A\_51\_P465445 | NM\_008633 | Mus musculus microtubule-associated protein 4 (Mtap4), mRNA | 0.22 | 0.00005 |  | |
| A\_51\_P365859 | NM\_183173 | Mus musculus ankyrin repeat domain 43 (Ankrd43), mRNA | 0.22 | 0.01158 |  | |
| A\_51\_P214837 | NM\_029277 | Mus musculus Rho GTPase activating protein 12 (Arhgap12), mRNA | 0.22 | 0.00079 |  | |
| A\_51\_P114576 | AK008716 | Mus musculus adult male stomach cDNA, RIKEN full-length enriched library, clone:2210012L08 product:unknown EST, full ins | 0.22 | 0.00028 |  | |
| A\_51\_P169730 | NM\_008218 | Mus musculus hemoglobin alpha, adult chain 1 (Hba-a1), mRNA | 0.22 | 0.01946 |  | |
| A\_51\_P345560 | NM\_028238 | Mus musculus Rab38, member of RAS oncogene family (Rab38), mRNA | 0.21 | 0.00442 |  | |
| A\_51\_P301636 | AK052809 | Mus musculus 10 days lactation, adult female mammary gland cDNA, RIKEN full-length enriched library, clone:D730020K15 pr | 0.21 | 0.00059 |  | |
| A\_51\_P131008 | NM\_010408 | Mus musculus hyperpolarization-activated, cyclic nucleotide-gated K+ 1 (Hcn1), mRNA | 0.20 | 0.00110 |  | |
| A\_51\_P270355 | NM\_019978 | Mus musculus double cortin and calcium/calmodulin-dependent protein kinase-like 1 (Dcamkl1), mRNA | 0.20 | 0.00027 |  | |
| A\_51\_P142744 | NM\_172294 | Mus musculus sulfatase 1 (Sulf1), mRNA | 0.20 | 0.00151 |  | |
| A\_51\_P501632 | NM\_009705 | Mus musculus arginase type II (Arg2), mRNA | 0.19 | 0.00388 |  | |
| A\_51\_P144319 | NM\_207654 | Mus musculus ephrin A5 (Efna5), transcript variant 1, mRNA | 0.19 | 0.00009 |  | |
| A\_51\_P497395 | NM\_145100 | Mus musculus Ly6/Plaur domain containing 1 (Lypdc1), mRNA | 0.19 | 0.00196 |  | |
| A\_51\_P161946 | NM\_175332 | Mus musculus RIKEN cDNA E130012A19 gene (E130012A19Rik), mRNA | 0.19 | 0.00006 |  | |
| A\_51\_P383369 | NM\_011857 | Mus musculus odd Oz/ten-m homolog 3 (Drosophila) (Odz3), mRNA | 0.18 | 0.00004 |  | |
| A\_51\_P221510 | NM\_029784 | Mus musculus RIKEN cDNA 6430514L14 gene (6430514L14Rik), mRNA | 0.18 | 0.00255 |  | |
| A\_51\_P267877 | NM\_139228 | Mus musculus rhomboid, veinlet-like 4 (Drosophila) (Rhbdl4), mRNA | 0.18 | 0.00001 |  | |
| A\_51\_P117226 | AK046533 | Mus musculus adult male adrenal gland cDNA, RIKEN full-length enriched library, clone:B330016H11 product:SMALL REC ho | 0.18 | 0.00009 |  | |
| A\_51\_P245895 | NM\_172632 | Mus musculus mitogen-activated protein kinase 4 (Mapk4), mRNA | 0.17 | 0.00001 |  | |
| A\_51\_P273213 | NM\_145890 | Mus musculus transcription factor CP2-like 2 (Tcfcp2l2), mRNA | 0.17 | 0.00114 |  | |
| A\_51\_P468362 | BC043115 | Mus musculus RIKEN cDNA 0610010D24 gene, mRNA (cDNA clone MGC:58041 IMAGE:6408630), complete cds | 0.16 | 0.00074 |  | |
| A\_51\_P368311 | NM\_011702 | Mus musculus vasoactive intestinal polypeptide (Vip), mRNA | 0.16 | 0.00299 |  | |
| A\_51\_P269663 | NM\_010585 | Mus musculus inositol 1,4,5-triphosphate receptor 1 (Itpr1), mRNA | 0.16 | 0.00019 |  | |
| A\_51\_P354706 | NM\_010094 | Mus musculus left right determination factor 1 (Lefty1), mRNA | 0.15 | 0.00004 |  | |
| A\_51\_P273609 | NM\_146125 | Mus musculus inositol 1,4,5-trisphosphate 3-kinase A (Itpka), mRNA | 0.15 | 0.00076 |  | |
| A\_51\_P307979 | NM\_007960 | Mus musculus ets variant gene 1 (Etv1), mRNA | 0.15 | 0.00003 |  | |
| A\_51\_P212543 | NM\_025696 | Mus musculus sortilin-related VPS10 domain containing receptor 3 (Sorcs3), mRNA | 0.14 | 0.00313 |  | |
| A\_51\_P237374 | NM\_021387 | Mus musculus RIKEN cDNA 2900093B09 gene (2900093B09Rik), mRNA | 0.14 | 0.00009 |  | |
| A\_51\_P256170 | NM\_134066 | Mus musculus aldo-keto reductase family 1, member C18 (Akr1c18), mRNA | 0.13 | 0.00283 |  | |
| A\_51\_P272553 | NM\_011498 | Mus musculus basic helix-loop-helix domain containing, class B2 (Bhlhb2), mRNA | 0.13 | 0.00020 |  | |
| A\_51\_P511269 | NM\_011141 | Mus musculus POU domain, class 3, transcription factor 1 (Pou3f1), mRNA | 0.13 | 0.00010 |  | |
| A\_51\_P396719 | NM\_144944 | Mus musculus G protein-coupled receptor 73-like 1 (Gpr73l1), mRNA | 0.12 | 0.00104 |  | |
| A\_51\_P293938 | AK004534 | Mus musculus 18-day embryo whole body cDNA, RIKEN full-length enriched library, clone:1190017B18 product:hypothetical P- | 0.12 | 0.00069 |  | |
| A\_51\_P196718 | NM\_009622 | Mus musculus adenylate cyclase 1 (Adcy1), mRNA | 0.12 | 0.00012 |  | |
| A\_51\_P325904 | XM\_148966 | PREDICTED: inhibin beta-B | 0.12 | 0.00039 |  | |
| A\_51\_P104985 | AI391295 | mb65f07.y1 Soares mouse p3NMF19.5 Mus musculus cDNA clone IMAGE:334309 5'. | 0.11 | 0.00002 |  | |
| A\_51\_P389957 | NM\_016758 | Mus musculus regulator of G-protein signaling 14 (Rgs14), mRNA | 0.11 | 0.00012 |  | |
| A\_51\_P216593 | NM\_011716 | Mus musculus Wolfram syndrome 1 homolog (human) (Wfs1), mRNA | 0.11 | 0.00060 |  | |
| A\_51\_P484111 | NM\_016762 | Mus musculus matrilin 2 (Matn2), mRNA | 0.10 | 0.00180 |  | |
| A\_51\_P233153 | NM\_153163 | Mus musculus Ca2+-dependent activator protein for secretion 2 (Cadps2), mRNA | 0.10 | 0.00072 |  | |
| A\_51\_P479688 | NM\_173370 | Mus musculus CDP-diacylglycerol synthase 1 (Cds1), mRNA | 0.10 | 0.00166 |  | |
| A\_51\_P353252 | NM\_178920 | Mus musculus mal, T-cell differentiation protein 2 (Mal2), mRNA | 0.09 | 0.00100 |  | |
| A\_51\_P151909 | NM\_145584 | Mus musculus spondin 1, (f-spondin) extracellular matrix protein (Spon1), mRNA | 0.09 | 0.00022 |  | |
| A\_51\_P491635 | NM\_027366 | Mus musculus lymphocyte antigen 6 complex, locus G6E (Ly6g6e), mRNA | 0.08 | 0.00030 |  | |
| A\_51\_P374752 | NM\_011255 | Mus musculus retinol binding protein 4, plasma (Rbp4), mRNA | 0.08 | 0.00028 |  | |
| A\_51\_P322115 | NM\_010483 | Mus musculus 5-hydroxytryptamine (serotonin) receptor 5B (Htr5b), mRNA | 0.08 | 0.00001 |  | |
| A\_51\_P289107 | NM\_133849 | Mus musculus histamine receptor H 3 (Hrh3), mRNA | 0.08 | 0.00012 |  | |
| A\_51\_P356052 | NM\_175012 | Mus musculus gastrin releasing peptide (Grp), mRNA | 0.07 | 0.00016 |  | |
| A\_51\_P335801 | NM\_009788 | Mus musculus calbindin-28K (Calb1), mRNA | 0.07 | 0.00605 |  | |
| A\_51\_P271417 | NM\_178887 | Mus musculus fibrinogen C domain containing 1 (Fibcd1), mRNA | 0.05 | 0.00051 |  | |
| A\_51\_P422429 | NM\_010825 | Mus musculus myeloid ecotropic viral integration site-related gene 1 (Mrg1), mRNA | 0.05 | 0.00015 |  | |
| A\_51\_P334104 | NM\_007833 | Mus musculus decorin (Dcn), mRNA | 0.04 | 0.00066 |  | |
| A\_51\_P427563 | NM\_025815 | Mus musculus copine VIII (Cpne8), mRNA | 0.03 | 0.00033 |  | |
| A\_51\_P284849 | XM\_131258 | PREDICTED: calcium-independent alpha-latrotoxin receptor homolog 2 | 0.02 | 0.00002 |  | |
| A\_51\_P102987 | NM\_001002927 | Mus musculus preproenkephalin 1 (Penk1), mRNA | 0.02 | 0.00000 |  | |
| A\_51\_P458638 | NM\_183136 | Mus musculus RIKEN cDNA C630041L24 gene (C630041L24Rik), mRNA | 0.02 | 0.00001 |  | |
| A\_51\_P296846 | NM\_172610 | Mus musculus DNA segment, Chr 15, Brigham & Women's Genetics 0669 expressed (D15Bwg0669e), mRNA | 0.02 | 0.00001 |  | |
|  |  |  |  |  |  |  |
