# Supplementary material for: The functional genome of CA1 and CA3 neurons under native conditions and in response to ischemia
Source: BMC Genomics. 2007 Oct 15;8:370. doi: 10.1186/1471-2164-8-370 (PMC2194787; doi:10.1186/1471-2164-8-370)
Supplement: Additional file 3 — Genes with differential expression in CA3 and CA1 under native conditions: Verification by detection in brain mapping projects. Shown are genes which had significantly differing distribution to CA3 and CA1. Selected genes with matches in brain mapping projects (BGEM or Allen Brain Atlas) are given with the gene symbol, gene name, Refseq accession number, Agilent probe number, preference of enrichment, and enrichment factor, and location in or link to microgrpahs of in situ hybridizations. All genes that were found are confirmed by the in-situ hybridizations. There is also general good correlation of the enrichment factors and visual impressions of the staining intensities in CA3 or CA1. [file 1471-2164-8-370-S3.htm]

| Gene symbol | Gene name | Acc. No. | Agilent probe No. | Enriched in | Factor | Link to BGEM |  |  |  |  |
| bok | bcl-2-related ovarian killer protein | NM\_016778 | A\_51\_P373082 | CA3 | 33.4 | http://www.stjudebgem.org/web/view/mediumImage/viewMediumImage.php?slideId=29509 |  |  |  |  |
| pvrl3 | poliovirus receptor-related 3 | NM\_021495 | A\_51\_P125009 | CA3 | 22.4 | http://www.stjudebgem.org/web/view/mediumImage/viewMediumImage.php?slideId=27022� |  |  |  |  |
| chgb | chromograninB | NM\_007694 | A\_51\_P191669 | CA3 | 11.1 | http://www.stjudebgem.org/web/view/mediumImage/viewMediumImage.php?slideId=16399 |  |  |  |  |
| npy2r | neuropeptide Y receptor 2 | NM\_008731 | A\_51\_P311919 | CA3 | 5.8 | http://www.stjudebgem.org/web/view/mediumImage/viewMediumImage.php?slideId=9746 |  |  |  |  |
| nrn1 | neuritin 1 | NM\_153529 | A\_51\_P308844 | CA3 | 4.8 | http://www.stjudebgem.org/web/view/mediumImage/viewMediumImage.php?slideId=8260� |  |  |  |  |
| hpcal1 | hippocalcin-like 1 | NM\_016677 | A\_51\_P487360 | CA3 | 3.7 | http://www.stjudebgem.org/web/view/mediumImage/viewMediumImage.php?slideId=11689 |  |  |  |  |
| rasa3 | RAS p21 protein activator 3� | NM\_009025 | A\_51\_P178063 | CA3 | 2.6 | http://www.stjudebgem.org/web/view/largeImage/viewLargeImage.php?slideId=30050 |  |  |  |  |
| epha4 | Eph receptor A4� | NM\_007936 | A\_51\_P221762 | CA3 | 2.6 | http://www.stjudebgem.org/web/view/largeImage/viewLargeImage.php?slideId=7565 |  |  |  |  |
| tgfb2 | transforming growth factor, beta 2 | NM\_009367 | A\_51\_P317640 | CA3 | 2.4 | http://www.stjudebgem.org/web/view/largeImage/viewLargeImage.php?slideId=53 |  |  |  |  |
| foxo1 | forkhead box O1 | NM\_019739 | A\_51\_P138044 | CA3 | 1.6 | http://www.stjudebgem.org/web/view/largeImage/viewLargeImage.php?slideId=4432 |  |  |  |  |
| psen2 | presenilin 2 | NM\_011183 | A\_51\_P384033 | CA3 | 1.5 | http://www.stjudebgem.org/web/view/largeImage/viewLargeImage.php?slideId=9823 |  |  |  |  |
|  |  |  |  |  |  |  |  |  |  |  |
| penk1 | preproenkephalin 1 | NM\_001002927 | A\_51\_P102987 | CA1 | 44.1 | http://www.stjudebgem.org/web/view/mediumImage/viewMediumImage.php?slideId=13851 |  |  |  |  |
| Wfs1 | Wolfram syndrome 1 homolog | NM\_011716 | A\_51\_P216593 | CA1 | 9.1 | http://www.stjudebgem.org/web/view/mediumImage/viewMediumImage.php?slideId=44856� |  |  |  |  |
| Gpr73l1 | G protein-coupled receptor 73-like 1 | NM\_144944 | A\_51\_P396719 | CA1 | 8.1 | http://www.stjudebgem.org/web/view/mediumImage/viewMediumImage.php?slideId=36643� |  |  |  |  |
| pou3f1 | POU domain, class 3, transcription factor 1 | NM\_011141 | A\_51\_P511269 | CA1 | 7.8 | http://www.stjudebgem.org/web/view/mediumImage/viewMediumImage.php?slideId=29255� |  |  |  |  |
| itpr1 | inositol 1,4,5-triphosphate receptor 1 | NM\_010585 | A\_51\_P269663 | CA1 | 6.3 | http://www.stjudebgem.org/web/view/mediumImage/viewMediumImage.php?slideId=29732� |  |  |  |  |
| lypdc1 | ly6/Plaur domain containing 1 | NM\_145100 | A\_51\_P497395 | CA1 | 5.4 | http://www.stjudebgem.org/web/view/mediumImage/viewMediumImage.php?slideId=44510� |  |  |  |  |
| foxP1 | forkhead box P1� | NM\_053202 | A\_51\_P344113 | CA1 | 3.6 | http://www.stjudebgem.org/web/view/mediumImage/viewMediumImage.php?slideId=25794� |  |  |  |  |
| Plk2 | polo-like kinase 2 | NM\_152804 | A\_51\_P290576 | CA1 | 3.4 | http://www.stjudebgem.org/web/view/largeImage/viewLargeImage.php?slideId=37325 |  |  |  |  |
| sez6 | seizure related gene 6� | NM\_021286 | A\_51\_P425402 | CA1 | 3.3 | http://www.stjudebgem.org/web/view/largeImage/viewLargeImage.php?slideId=2745 |  |  |  |  |
| dapk1 | death associated protein kinase 1 | NM\_029653 | A\_51\_P444633 | CA1 | 2.5 | http://www.stjudebgem.org/web/view/largeImage/viewLargeImage.php?slideId=11547 |  |  |  |  |
| homer2 | homer 2 | NM\_011983 | A\_51\_P352303 | CA1 | 2.0 | http://www.stjudebgem.org/web/view/largeImage/viewLargeImage.php?slideId=25939 |  |  |  |  |
|  |  |  |  |  |  |  |  |  |  |  |
|  |  |  |  |  |  |  |  |  |  |  |
| Gene symbol | Gene name | Acc. No. | Agilent probe No. | Enriched in | Factor | Allen Brain Atlas coordinates |  |  |  |  |
| rerg | RAS-like, estrogen-regulated, growth-inhibitor� | NM\_181988 | A\_51\_P305508 | CA3 | 21.5 | Rerg\_56 |  |  |  |  |
| Ras11a | RAS-like family 11 member A | XM\_485698 | A\_51\_P340699 | CA3 | 16.1 | Ras11a\_69 |  |  |  |  |
| Cd109 | CD109 antigen� | NM\_153098 | A\_51\_P423465 | CA3 | 13.49 | Cd109\_261 |  |  |  |  |
| Ncald | Neurocalcin delta | NM\_134094 | A\_51\_P291713 | CA3 | 10.1 | Ncald\_56 |  |  |  |  |
| Trhde | TRH-degrading enzyme | NM\_146241 | A\_51\_P320454 | CA3 | 8.3 | Trhde\_260 |  |  |  |  |
| Cova1 | cytosolic ovarian carcinoma antigen 1 | NM\_145951 | A\_51\_P267986 | CA3 | 7.4 | Cova1\_242 |  |  |  |  |
| Grik4 | glutamate receptor, ionotropic, kainate 4 | NM\_175481 | A\_51\_P384993 | CA3 | 6.8 | Grik4\_246 |  |  |  |  |
| Synpr | synaptoporin | NM\_028052 | A\_51\_P376656 | CA3 | 6.5 | Synpr\_221\_2378 |  |  |  |  |
| ephb1 | Eph B1 receptor | NM\_173447 | A\_51\_P358485 | CA3 | 5.6 | Ephb1\_268 |  |  |  |  |
| slit2 | Slit2 | NM\_178804 | A\_51\_P496569 | CA3 | 5.5 | Slit2\_230 |  |  |  |  |
| Ptgs2 | prostaglandin-endoperoxide synthase 2 | NM\_011198 | A\_51\_P254855 | CA3 | 5.5 | Ptgs2\_234 |  |  |  |  |
| Fmo1 | flavin containing monooxygenase 1 | NM\_010231 | A\_51\_P155190 | CA3 | 4.6 | Fmo1\_224 |  |  |  |  |
| Car4 | carbonic anhydrase 4 | NM\_007607 | A\_51\_P407025 | CA3 | 4.5 | Car4\_240 |  |  |  |  |
| Tmem46 | transmembrane protein 46 | NM\_145463 | A\_51\_P408653 | CA3 | 4.4 | Tmem46\_242 |  |  |  |  |
| Ogfrl1 | opioid growth factor receptor-like 1 | BC019747 | A\_51\_P347312 | CA3 | 4.3 | Ogfrl1\_254 |  |  |  |  |
| Clstn2 | calsyntenin 2 | NM\_022319 | A\_51\_P370423 | CA3 | 4.2 | Clstn2\_68 |  |  |  |  |
| Tspan17 | Tetraspanin 17 | NM\_028841 | A\_51\_P137079 | CA3 | 4.0 | Tspan17\_64 |  |  |  |  |
| Golph2 | golgi phosphoprotein 2 | NM\_027307 | A\_51\_P171200 | CA3 | 4.0 | Golph2\_82 |  |  |  |  |
| Pfkfb3 | 6-phosphofructo-2-kinase | NM\_133232 | A\_51\_P212491 | CA3 | 3.8 | Pfkfb3\_49 |  |  |  |  |
| Tagln3 | transgelin 3 | NM\_019754 | A\_51\_P303919 | CA3 | 3.8 | Tagln3\_60 |  |  |  |  |
| Prkcd | protein kinase C, delta | NM\_011103 | A\_51\_P460734 | CA3 | 3.8 | Prkcd\_73 |  |  |  |  |
| Ccnd2 | cyclin D2 | NM\_009829 | A\_51\_P433228 | CA3 | 3.6 | Ccnd2\_222\_1905 |  |  |  |  |
| Rph3a | rabphilin 3A | NM\_011286 | A\_51\_P436596 | CA3 | 3.6 | Rph3a\_62 |  |  |  |  |
| Unc5c | unc-5 homolog C | NM\_009472 | A\_51\_P152918 | CA3 | 3.5 | Unc5c\_57 |  |  |  |  |
| Serpinf1 | Serpinf1 | NM\_011340 | A\_51\_P517075 | CA3 | 3.4 | Serpinf1\_74 |  |  |  |  |
| Nptx1 | neuronal pentraxin 1 | NM\_008730 | A\_51\_P360615 | CA3 | 3.4 | Nptx1\_253 |  |  |  |  |
| Il16 | interleukin 16 | NM\_010551 | A\_51\_P372702 | CA3 | 3.2 | Il16\_238 |  |  |  |  |
| Apba2bp | amyloid beta (A4) precursor protein-binding, family A, member 1 binding protein� | NM\_021546 | A\_51\_P269103 | CA3 | 3.2 | Apba2bp\_256 |  |  |  |  |
| C1r | complement component 1, r subcomponent | NM\_023143 | A\_51\_P384318 | CA3 | 3.1 | C1r\_40 |  |  |  |  |
| Enc1 | ectodermal-neural cortex 1 | NM\_007930 | A\_51\_P126437 | CA3 | 3 | Enc1\_48\_2151 |  |  |  |  |
| Lhfpl2 | lipoma HMGIC fusion partner-like 2 | NM\_172589 | A\_51\_P302450 | CA3 | 2.9 | Lhfpl2\_258 |  |  |  |  |
| Tmeff2 | transmembrane protein with EGF-like and two follistatin-like domains 2 | NM\_019790 | A\_51\_P376959 | CA3 | 2.9 | Tmeff2\_230 |  |  |  |  |
| Crispld1 | cysteine-rich secretory protein LCCL domain containing 1 | NM\_031402 | A\_51\_P249749 | CA3 | 2.9 | Crispld1\_53 |  |  |  |  |
| Nrp1 | �neuropilin 1 | NM\_008737 | A\_51\_P469285 | CA3 | 2.8 | Nrp1\_41 |  |  |  |  |
| Efemp1 | EGF-containing fibulin-like extracellular matrix protein 1 | NM\_146015 | A\_51\_P337412 | CA3 | 2.8 | Efemp1\_45 |  |  |  |  |
| Syn2 | synapsin II� | NM\_013681 | A\_51\_P396331 | CA3 | 2.8 | Syn2\_252 |  |  |  |  |
| Gdf10 | growth differentiation factor 10 | NM\_145741 | A\_51\_P185248 | CA3 | 2.7 | Gdf10\_66 |  |  |  |  |
| Prss35 | protease, serine, 35 | NM\_178738 | A\_51\_P420600 | CA3 | 2.7 | Prss35\_44 |  |  |  |  |
| Kcna1 | potassium voltage-gated channel, shaker-related subfamily, member 1 | NM\_010595 | A\_51\_P222882 | CA3 | 2.7 | Kcna1\_266 |  |  |  |  |
| EphA3 | Eph receptor A3 | NM\_010140 | A\_51\_P246133 | CA3 | 2.6 | Epha3\_236 |  |  |  |  |
| Stmn2 | stathmin-like 2 | NM\_025285 | A\_51\_P164995 | CA3 | 2.6 | Stmn2\_64 |  |  |  |  |
| Mycl1 | lung carcinoma myc related oncogene 1 | NM\_008506 | A\_51\_P402818 | CA3 | 2.6 | Mycl1\_60 |  |  |  |  |
| Mbldc1 | cDNA sequence AB112350 | NM\_178728 | A\_51\_P392943 | CA3 | 2.6 | Mbldc1\_69 |  |  |  |  |
| Tle4 | transducin-like enhancer of split 4 | NM\_011600 | A\_51\_P346543 | CA3 | 2.6 | Tle4\_54 |  |  |  |  |
| Tmem9 | transmembrane protein 9 | NM\_025439 | A\_51\_P359237 | CA3 | 2.4 | Tmem9\_72 |  |  |  |  |
| Aqp11 | aquaporin 11� | NM\_175105 | A\_51\_P447248 | CA3 | 2.3 | Aqp11\_53 |  |  |  |  |
| Cib2 | calcium and integrin binding family member 2 | NM\_019686 | A\_51\_P302823 | CA3 | 2.2 | Cib2\_265 |  |  |  |  |
| Dnajc6 | DnaJ (Hsp40) homolog, subfamily C, member 6 | NM\_198412 | A\_51\_P431885 | CA3 | 2.2 | Dnajc6\_248 |  |  |  |  |
| Ostf1 | osteoclast stimulating factor 1 | NM\_017375 | A\_51\_P277345 | CA3 | 2.1 | Ostf1\_246 |  |  |  |  |
| Homer3 | Homer3 | NM\_011984 | A\_51\_P466613 | CA3 | 2 | Homer3\_214 |  |  |  |  |
| Aplp2 | amyloid beta (A4) precursor-like protein 2 | NM\_009691 | A\_51\_P237599 | CA3 | 2 | Aplp2\_65 |  |  |  |  |
| Robo1 | roundabout homolog 1 | NM\_019413 | A\_51\_P174314 | CA3 | 1.9 | Robo1\_284 |  |  |  |  |
| Bin3 | bridging integrator 3 | NM\_021328 | A\_51\_P160514 | CA3 | 1.8 | Bin3\_56 |  |  |  |  |
| Faim | Fas apoptotic inhibitory molecule | NM\_011810 | A\_51\_P267194 | CA3 | 1.8 | Faim\_64 |  |  |  |  |
| Usp48 | �ubiquitin specific protease 48 | XM\_485461 | A\_51\_P319234 | CA3 | 1.7 | Usp31\_225\_2054 |  |  |  |  |
| Zyx | Zyxin | NM\_011777 | A\_51\_P473252 | CA3 | 1.7 | Zyx\_270 |  |  |  |  |
| Pcdha3 | protocadherin alpha 3 | NM\_138662 | A\_51\_P360531 | CA3 | 1.6 | Pcdha3\_64 |  |  |  |  |
|  |  |  |  |  |  |  |  |  |  |  |
| mpped1 | metallophosphoesterase domain containing 1� | NM\_172610 | A\_51\_P296846 | CA1 | 61.1 | Mpped1\_256 |  |  |  |  |
| C630041L24 | RIKEN cDNA C630041L24 gene | NM\_183136 | A\_51\_P458638 | CA1 | 55.5 | C630041L24Rik\_280 |  |  |  |  |
| Calb1 | calbindin-28K | NM\_009788 | A\_51\_P335801 | CA1 | 15.1 | Calb1\_284 |  |  |  |  |
| Htr5b | 5-hydroxytryptamine (serotonin) receptor 5B | NM\_010483 | A\_51\_P322115 | CA1 | 12.5 | Htr5b\_72 |  |  |  |  |
| Matn2 | matrilin 2 | NM\_016762 | A\_51\_P484111 | CA1 | 10 | Matn2\_49 |  |  |  |  |
| Cadps2 | Ca2+-dependent activator protein for secretion 2 | NM\_153163 | A\_51\_P233153 | CA1 | 10 | Cadps2\_53 |  |  |  |  |
| Sorcs3 | sortilin-related VPS10 domain containing receptor 3 | NM\_025696 | A\_51\_P212543 | CA1 | 7.1 | Sorcs3\_242 |  |  |  |  |
| Itpka | inositol 1,4,5-trisphosphate 3-kinase A | NM\_146125 | A\_51\_P273609 | CA1 | 6.7 | Itpka\_78 |  |  |  |  |
| Rhbdl4 | rhomboid, veinlet-like 4 (Drosophila) | NM\_139228 | A\_51\_P267877 | CA1 | 5.6 | Rhbdl3\_64� |  |  |  |  |
| Dcamkl1 | double cortin and calcium/calmodulin-dependent protein kinase-like 1 | NM\_019978 | A\_51\_P270355 | CA1 | 5 | Dcamkl1\_84 |  |  |  |  |
| Ankrd43 | ankyrin repeat domain 43 | NM\_183173 | A\_51\_P365859 | CA1 | 4.5 | Aknrd43\_62 |  |  |  |  |
| Cacna1g | calcium channel, voltage-dependent, T type, alpha 1G subunit | NM\_009783 | A\_51\_P466910 | CA1 | 4.5 | Cacna1g\_246 |  |  |  |  |
| Hnt | neurotrimin� | NM\_172290 | A\_51\_P272993 | CA1 | 4.3 | Hnt\_253 |  |  |  |  |
| Gabra1 | GABA-A receptor subunit alpha1 | NM\_010250 | A\_51\_P247014 | CA1 | 3.9 | Gabra1\_53 |  |  |  |  |
| Doc2b | double C2, beta | NM\_007873 | A\_51\_P442704 | CA1 | 3.6 | Doc2b\_260 |  |  |  |  |
| Hunk | hormonally upregulated Neu-associated kinase | NM\_015755 | A\_51\_P103237 | CA1 | 3.3 | Hunk\_68\_2305 |  |  |  |  |
| Ccnd1 | cyclin D1 | NM\_007631 | A\_51\_P262759 | CA1 | 3.2 | Ccnd1\_50 |  |  |  |  |
| Trpc4 | transient receptor potential cation channel, subfamily C, member 4 | NM\_016984 | A\_51\_P516148 | CA1 | 3.2 | TrpC4\_226\_2357 |  |  |  |  |
| Sh3bp4 | �SH3-domain binding protein 4 | NM\_133816 | A\_51\_P322877 | CA1 | 2.8 | Sh3bp4\_265 |  |  |  |  |
| Gaa | glucosidase, alpha, acid | NM\_008064 | A\_51\_P218895 | CA1 | 2.7 | Gaa\_253 |  |  |  |  |
| Plcl2 | phospholipase C-like 2� | NM\_013880 | A\_51\_P219444 | CA1 | 2.7 | Plcl2\_80� |  |  |  |  |
| Dbp | D site albumin promoter binding protein | NM\_016974 | A\_51\_P180492 | CA1 | 2.7 | Dbp\_226\_1940 |  |  |  |  |
| Ramp1 | receptor (calcitonin) activity modifying protein 1 | NM\_016894 | A\_51\_P348665 | CA1 | 2.6 | Ramp1\_221\_2367 |  |  |  |  |
| Dapk1 | death associated protein kinase 1 | NM\_029653 | A\_51\_P444633 | CA1 | 2.5 | Dapk1\_74 |  |  |  |  |
| Sall2 | sal-like 2 | NM\_015772 | A\_51\_P137094 | CA1 | 2.5 | Sall2\_84 |  |  |  |  |
| Fabp3 | fatty acid binding protein 3 | NM\_010174 | A\_51\_P167535 | CA1 | 2.5 | Fabp3\_117 |  |  |  |  |
| Kctd15 | potassium channel tetramerisation domain containing 15� | NM\_146188 | A\_51\_P331805 | CA1 | 2.4 | Kctd15\_53� |  |  |  |  |
| Arhgef3 | Rho guanine nucleotide exchange factor (GEF) 3 | NM\_027871 | A\_51\_P380337 | CA1 | 2.3 | Arhgef\_86 |  |  |  |  |
| Dtx1 | deltex 1 homolog | NM\_008052 | A\_51\_P202050 | CA1 | 2.3 | Dtx1\_63\_2253 |  |  |  |  |
| Ngef | neuronal guanine nucleotide exchange factor | NM\_019867 | A\_51\_P170816 | CA1 | 2.2 | Ngf\_82 |  |  |  |  |
| Adcy8 | adenylate cyclase 8 | NM\_009623 | A\_51\_P303056 | CA1 | 2.1 | Adcy8\_62 |  |  |  |  |
| Drctnnb1a | down-regulated by Ctnnb1, a | NM\_053090 | A\_51\_P422124 | CA1 | 2.1 | Drctnnb1a\_85 |  |  |  |  |
| Rhoq | ras homolog gene family, member Q� | NM\_145491 | A\_51\_P338317 | CA1 | 2.0 | Rhoq\_52 |  |  |  |  |
| Pde9a | phosphodiesterase 9A | NM\_008804 | A\_51\_P383194 | CA1 | 1.9 | Pde9a\_221\_1965 |  |  |  |  |
| Tal1 | T-cell acute lymphocytic leukemia 1 | NM\_011527 | A\_51\_P255853 | CA1 | 1.9 | Tal1\_238 |  |  |  |  |
| Sbk1 | SH3-binding kinase 1 | NM\_145587 | A\_51\_P498388 | CA1 | 1.8 | Sbk1\_262 |  |  |  |  |
| Sepn1 | selenoprotein N, 1 | NM\_029100 | A\_51\_P358152 | CA1 | 1.8 | Sepn1\_84 |  |  |  |  |
| Bcl11b | B-cell leukemia/lymphoma 11B | NM\_021399 | A\_51\_P171772 | CA1 | 1.8 | Bcl11b\_100 |  |  |  |  |
| Ext1 | exostoses (multiple) 1 | NM\_010162 | A\_51\_P416689 | CA1 | 1.7 | Ext1\_142 |  |  |  |  |
| Bex4 | brain expressed, X-linked 4 | NM\_212457 | A\_51\_P494342 | CA1 | 1.6 | Bex4\_73 |  |  |  |  |
|  |  |  |  |  |  |  |  |  |  |  |
|  |  |  |  |  |  |  |  |  |  |  |
|  |  |  |  |  |  |  |  |  |  |  |
|  |  |  |  |  |  |  |  |  |  |  |
|  |  |  |  |  |  |  |  |  |  |  |
|  |  |  |  |  |  |  |  |  |  |  |
|  |  |  |  |  |  |  |  |  |  |  |
|  |  |  |  |  |  |  |  |  |  |  |
|  |  |  |  |  |  |  |  |  |  |  |
