# Supplementary material for: The functional genome of CA1 and CA3 neurons under native conditions and in response to ischemia
Source: BMC Genomics. 2007 Oct 15;8:370. doi: 10.1186/1471-2164-8-370 (PMC2194787; doi:10.1186/1471-2164-8-370)
Supplement: Additional file 4 — List of all significantly regulated genes in CA3 ischemic vs. sham. HTML file containing all genes significantly regulated in CA3 between the ischemic and the sham group. Given are Agilent probe numbers, accession numbers, gene names, enrichment factors ("M (ICA3/SCA3")), and false-discovery-rate corrected p-values. [file 1471-2164-8-370-S4.htm]

| Agilent probe# | Accession# | gene name | M (ICA3/SCA3) | P.fdr |
| A\_51\_P132530 | AK008821 | hypothetical PMP-22/EMP/MP20 and claudin family containing protein | 6.82 | 0,0003 |
| A\_51\_P144850 | NM\_178685 | PROTOCADHERIN 13 (FRAGMENT) homolog [Homo sapiens] | 6.23 | 0,0001 |
| A\_51\_P295085 | NM\_008760 | osteoglycin | 6.19 | 0,0017 |
| A\_51\_P394997 | NM\_009717 | Mus musculus neurogenic differentiation 6 (Neurod6), mRNA | 6.15 | 0,0002 |
| A\_51\_P162124 | AK012595 | unknown EST | 4.86 | 0,0001 |
| A\_51\_P338874 | NM\_027571 | Mus musculus, purinergic receptor P2Y, G-protein coupled 12, clone MGC:36953 IMAGE:4947057, mRNA, complete cds | 4.59 | 0,0018 |
| A\_51\_P152918 | NM\_009472 | Mus musculus unc5 homolog (C. elegans) 3 (Unc5h3), mRNA | 4.32 | 0,0001 |
| A\_51\_P515129 | XM\_620248 | hypothetical Sulfotransferase containing protein | 4.14 | 0,0019 |
| A\_51\_P333859 | NM\_175448 | hypothetical General substrate transporters/Cellular retinaldehyde-binding protein (CRAL)/Triple function domain (TRIO) containing protein | 4.00 | 0,0000 |
| A\_51\_P142972 | NM\_029947 | PR-DOMAIN CONTAINING PROTEIN 8 homolog [Homo sapiens] | 3.78 | 0,0209 |
| A\_51\_P397983 | AK077026 | SODIUM/HYDROGEN EXCHANGER 2 (NA(+)/H(+) EXCHANGER 2) (NHE-2) (H7) homolog [Rattus norvegicus] | 3.71 | 0,0040 |
| A\_51\_P263004 | NM\_016707 | Mus musculus B-cell CLL/lymphoma 11A (zinc finger protein) (Bcl11a), mRNA | 3.58 | 0,0003 |
| A\_51\_P372522 | NM\_001014995 | inferred: COTE1 PROTEIN. [Human] {Homo sapiens} | 3.53 | 0,0001 |
| A\_51\_P340699 | XM\_485698 | weakly similar to CDNA FLJ14361 FIS, CLONE HEMBA1000491, WEAKLY SIMILAR TO RAS-LIKE PROTEIN 2 [Homo sapiens] | 3.46 | 0,0110 |
| A\_51\_P296995 | NM\_011510 | Mus musculus ATP-binding cassette protein (Abcc8) mRNA, partial cds | 3.46 | 0,0026 |
| A\_51\_P121288 | NM\_021527 | Mus musculus McKusick-Kaufman syndrome protein (Mkks), mRNA | 3.46 | 0,0048 |
| A\_51\_P324551 | NM\_025785 | Mus musculus F-box only protein 25 (Fbxo25), mRNA | 3.41 | 0,0001 |
| A\_51\_P134045 | NM\_008792 | hypothetical protein | 3.41 | 0,0009 |
| A\_51\_P320454 | NM\_146241 | similar to THYROTROPIN-RELEASING HORMONE DEGRADING ECTOENZYME (EC 3.4.19.6) (TRH- DEGRADING ECTOENZYME) (TRH-DE) (TRH-SPECIFIC AMINOPEPTIDASE) (THYROLIBERINASE) (PYROGLUTAMYL-PEPTIDASE II) (PAP-II) [Rattus norvegicus] | 3.41 | 0,0038 |
| A\_51\_P479652 | XM\_125901 | TBP-interacting protein | 3.36 | 0,0000 |
| A\_51\_P298131 | NM\_013926 | Mus musculus chromobox homolog 8 (Drosophila Pc class) (Cbx8), mRNA | 3.36 | 0,0009 |
| A\_51\_P471498 | NM\_028049 | Mus musculus RIKEN cDNA 0610033L19 gene (0610033L19Rik), mRNA | 3.34 | 0,0001 |
| A\_51\_P209902 | NM\_177814 | hypothetical Histidine-rich region containing protein | 3.34 | 0,0010 |
| A\_51\_P340174 | NM\_172682 | hypothetical Ricin B-like lectins structure containing protein | 3.29 | 0,0076 |
| A\_51\_P341664 | NM\_133228 | Mus musculus KRAB zinc finger protein (Mzf22) (Mzf22), mRNA | 3.27 | 0,0014 |
| A\_51\_P216480 | NM\_178725 | weakly similar to BRAIN TUMOR ASSOCIATED PROTEIN NAG14 [Homo sapiens] | 3.27 | 0,0002 |
| A\_51\_P173114 | NM\_130878 | Mus musculus photoreceptor cadherin (Prcad-pending), mRNA | 3.27 | 0,0023 |
| A\_51\_P477682 | NM\_008939 | Mus musculus protease, serine, 12 neurotrypsin, (motopsin) (Prss12), mRNA | 3.25 | 0,0054 |
| A\_51\_P335981 | NM\_026272 | Mus musculus, RIKEN cDNA 4430402O11 gene, clone MGC:27610 IMAGE:4503713, mRNA, complete cds | 3.25 | 0,0000 |
| A\_51\_P134627 | NM\_178283 | Mus musculus ankyrin repeat domain-containing SOCS box protein 13 (Asb13), mRNA | 3.25 | 0,0000 |
| A\_51\_P206835 | NM\_010687 | Mus musculus like-glycosyltransferase (Large), mRNA | 3.25 | 0,0002 |
| A\_51\_P495986 | NM\_025508 | Mus musculus guanosine monophosphate reductase (Gmpr), mRNA | 3.20 | 0,0001 |
| A\_51\_P108383 | NM\_026649 | hypothetical protein | 3.20 | 0,0001 |
| A\_51\_P262196 | NM\_011069 | Mus musculus peroxisomal biogenesis factor 11b (Pex11b), mRNA | 3.16 | 0,0000 |
| A\_51\_P220262 | BC066016 | unknown EST | 3.16 | 0,0004 |
| A\_51\_P474188 | NM\_198942 | Mus musculus, Similar to RIKEN cDNA 2810407E23 gene, clone IMAGE:4489006, mRNA, partial cds | 3.16 | 0,0001 |
| A\_51\_P497395 | NM\_145100 | hypothetical Snake toxin-like structure containing protein | 3.12 | 0,0089 |
| A\_51\_P427232 | NM\_183088 | ethanol induced 6 | 3.10 | 0,0004 |
| A\_51\_P152623 | NM\_008552 | Mus musculus MAS1 oncogene (Mas1), mRNA | 3.07 | 0,0008 |
| A\_51\_P229613 | NM\_008792 | Mus musculus proprotein convertase subtilisin/kexin type 2 (Pcsk2), mRNA | 3.07 | 0,0026 |
| A\_51\_P514405 | NM\_019741 | Mus musculus solute carrier family 2 (facilitated glucose transporter), member 5 (Slc2a5), mRNA | 3.05 | 0,0000 |
| A\_51\_P496715 | NM\_029037 | hypothetical Eukaryotic protein kinase containing protein | 3.03 | 0,0000 |
| A\_51\_P392303 | NM\_010733 | Mus musculus mRNA for leucine-rich repeat protein, partial cds | 3.03 | 0,0005 |
| A\_51\_P411200 | NM\_027391 | hypothetical Nitroreductase family containing protein | 3.03 | 0,0022 |
| A\_51\_P274259 | AK053807 | ADENYLATE KINASE ISOZYME 5 homolog [Mus musculus] | 3.03 | 0,0107 |
| A\_51\_P240019 | NM\_013755 | Mus musculus glycogenin 1 (Gyg1), mRNA | 3.01 | 0,0000 |
| A\_51\_P148122 | NM\_027910 | Mus musculus RIKEN cDNA 1300011D16 gene (1300011D16Rik), mRNA | 2.99 | 0,0001 |
| A\_51\_P515108 | TC1483082 | Mus musculus cDNA, 5 end | 2.99 | 0,0001 |
| A\_51\_P467869 | NM\_007389 | Mus musculus cholinergic receptor, nicotinic, alpha polypeptide 1 (muscle) (Chrna1), mRNA | 2.99 | 0,0002 |
| A\_51\_P191779 | NM\_133859 | Mus musculus RIKEN cDNA 2810002E22 gene (2810002E22Rik), mRNA | 2.99 | 0,0003 |
| A\_51\_P383718 | NM\_030132 | hypothetical Domain of unknown function DUF94 containing protein | 2.97 | 0,0000 |
| A\_51\_P151675 | A\_51\_P151675 | Mus musculus cDNA, 5 end | 2.97 | 0,0002 |
| A\_51\_P244923 | NM\_134149 | Mus musculus expressed sequence AI837181 (AI837181), mRNA | 2.97 | 0,0006 |
| A\_51\_P236160 | NM\_172392 | Mus musculus, Similar to zinc finger protein 208, clone MGC:40747 IMAGE:5365910, mRNA, complete cds | 2.93 | 0,0003 |
| A\_51\_P471940 | XM\_357108 | MITOCHONDRIAL 39S RIBOSOMAL PROTEIN L45 (MRP-L45) homolog [Homo sapiens] | 2.93 | 0,0000 |
| A\_51\_P243623 | AK003055 | weakly similar to BREAST CANCER METASTASIS-SUPPRESSOR 1 [Homo sapiens] | 2.87 | 0,0000 |
| A\_51\_P353914 | NM\_008319 | Mus musculus intercellular adhesion molecule 5, telencephalin (Icam5), mRNA | 2.87 | 0,0119 |
| A\_51\_P197509 | NM\_153594 | hypothetical Protein-L-isoaspartate(D-aspartate) O-methyltransferase containing protein | 2.85 | 0,0003 |
| A\_51\_P490348 | NM\_025423 | Mus musculus RIKEN cDNA 1110059E24 gene (1110059E24Rik), mRNA | 2.83 | 0,0000 |
| A\_51\_P456098 | NM\_028223 | Mus musculus, RIKEN cDNA 3010001K23 gene, clone MGC:8187 IMAGE:3590497, mRNA, complete cds | 2.83 | 0,0003 |
| A\_51\_P485349 | NM\_145544 | Mus musculus, Similar to RAP1, GTP-GDP dissociation stimulator 1, clone MGC:18981 IMAGE:4008751, mRNA, complete cds | 2.81 | 0,0000 |
| A\_51\_P191354 | XM\_126946 | weakly similar to PEROXISOMAL LONG CHAIN ACYL-COA THIOESTERASE IB [Mus musculus] | 2.81 | 0,0008 |
| A\_51\_P467539 | NM\_175191 | PROBABLE G PROTEIN-COUPLED RECEPTOR GPR22 homolog [Homo sapiens] | 2.81 | 0,0117 |
| A\_51\_P517198 | NM\_009959 | Mus musculus protocadherin alpha 5 (Pcdha5), mRNA | 2.81 | 0,0001 |
| A\_51\_P302938 | NM\_145216 | RAS-LIKE PROTEIN RRP22 (RAS-RELATED PROTEIN ON CHROMOSOME 22) homolog [Homo sapiens] | 2.79 | 0,0128 |
| A\_51\_P396696 | NM\_198417 | zinc finger protein 60 | 2.79 | 0,0002 |
| A\_51\_P231630 | NM\_001001792 | Mus musculus zinc finger protein 239 (Zfp239), mRNA | 2.79 | 0,0001 |
| A\_51\_P515242 | AK122459 | hypothetical protein | 2.77 | 0,0484 |
| A\_51\_P446132 | NM\_172601 | RAS RELATED PROTEIN RAB | 2.77 | 0,0003 |
| A\_51\_P267986 | NM\_145951 | TUMOR-ASSOCIATED HYDROQUINONE (NADH) OXIDASE TNOX homolog [Homo sapiens] | 2.75 | 0,0024 |
| A\_51\_P400160 | AK013705 | hypothetical protein | 2.75 | 0,0006 |
| A\_51\_P165507 | NM\_007855 | Mus musculus dermis expressed 1 (Dermo1), mRNA | 2.75 | 0,0011 |
| A\_51\_P126437 | NM\_007930 | RIKEN full-length enriched, 10 days neonate brain Mus musculus cDNA clone K630055B09 3 | 2.75 | 0,0114 |
| A\_51\_P273609 | NM\_146125 | Mus musculus, clone MGC:28924 IMAGE:3481738, mRNA, complete cds | 2.75 | 0,0180 |
| A\_51\_P366227 | NM\_177359 | cytosolic 5 nucleotidase, type 1A | 2.73 | 0,0025 |
| A\_51\_P478003 | NM\_172555 | POLY(A) POLYMERASE GAMMA (EC 2.7.7.19) (NEO-POLY(A) POLYMERASE) homolog [Homo sapiens] | 2.73 | 0,0001 |
| A\_51\_P218216 | AK031357 | ADRENAL GLAND PROTEIN AD-005 homolog [Homo sapiens] | 2.71 | 0,0004 |
| A\_51\_P122582 | NM\_144915 | hypothetical Lipase containing protein | 2.71 | 0,0006 |
| A\_51\_P117236 | NM\_009329 | Mus musculus zinc finger protein 354A (Zfp354a), mRNA | 2.71 | 0,0003 |
| A\_51\_P492797 | NM\_177736 | Mus musculus, Similar to hypothetical protein MGC3036, clone MGC:28139 IMAGE:3981816, mRNA, complete cds | 2.71 | 0,0008 |
| A\_51\_P374900 | NM\_028808 | G protein-coupled receptor 68 homolog [Homo sapiens] | 2.69 | 0,0137 |
| A\_51\_P496845 | NM\_007604 | Mus musculus capping protein alpha 2 (Cappa2), mRNA | 2.69 | 0,0010 |
| A\_51\_P172565 | NM\_025300 | Mus musculus, mitochondrial ribosomal protein L15, clone MGC:29279 IMAGE:3498041, mRNA, complete cds | 2.69 | 0,0021 |
| A\_51\_P346488 | BC085129 | synapsin II | 2.69 | 0,0002 |
| A\_51\_P108862 | NM\_012003 | Mus musculus COP9 (constitutive photomorphogenic) homolog, subunit 7a (Arabidopsis thaliana) (Cops7a), mRNA | 2.68 | 0,0000 |
| A\_51\_P223498 | NM\_172653 | weakly similar to CDNA FLJ32338 FIS, CLONE PROST2005919, MODERATELY SIMILAR TO HUMAN BREAST CANCER, ESTROGEN REGULATED LIV-1 PROTEIN (LIV-1) MRNA [Homo sapiens] | 2.68 | 0,0006 |
| A\_51\_P282673 | NM\_175098 | weakly similar to N-ACETYLGLUCOSAMINYLTRANSFERASE (FRAGMENT) [Homo sapiens] | 2.68 | 0,0001 |
| A\_51\_P325862 | NM\_013751 | musculus 10, 11 days embryo whole body cDNA, RIKEN full-length enriched library, clone:2810012B06:unclassifiable transcript, full insert sequence | 2.66 | 0,0003 |
| A\_51\_P335750 | NM\_029716 | Mus musculus RIKEN cDNA 1700112L09 gene (1700112L09Rik), mRNA | 2.66 | 0,0015 |
| A\_51\_P263419 | NM\_013705 | Mus musculus zinc finger protein 30 (Zfp30), mRNA | 2.66 | 0,0004 |
| A\_51\_P232790 | AK030381 | hypothetical EF-hand containing protein | 2.66 | 0,0031 |
| A\_51\_P202530 | NM\_146127 | hypothetical protein | 2.64 | 0,0000 |
| A\_51\_P444379 | NM\_198294 | hypothetical Serine-rich region containing protein | 2.64 | 0,0005 |
| A\_51\_P284686 | XM\_485800 | unclassifiable | 2.64 | 0,0020 |
| A\_51\_P362089 | NM\_146052 | LEUCINE RICH REPEAT PROTEIN LRRC3 | 2.64 | 0,0003 |
| A\_51\_P330016 | NM\_015814 | dickkopf homolog 3 (Xenopus laevis) | 2.64 | 0,0020 |
| A\_51\_P267634 | NM\_178751 | hypothetical protein | 2.64 | 0,0030 |
| A\_51\_P363935 | AK021067 | unknown EST | 2.64 | 0,0014 |
| A\_51\_P202979 | NM\_033567 | Mus musculus cat eye syndrome chromosome region, candidate 6 homolog (human) (Cecr6), mRNA | 2.64 | 0,0209 |
| A\_51\_P175871 | NM\_010325 | Mus musculus glutamate oxaloacetate transaminase 2, mitochondrial (Got2), mRNA | 2.64 | 0,0001 |
| A\_51\_P359237 | NM\_025439 | Mus musculus RIKEN cDNA 1500015G18 gene (1500015G18Rik), mRNA | 2.62 | 0,0008 |
| A\_51\_P355416 | NM\_022656 | Mus musculus nischarin (Nisch), mRNA | 2.62 | 0,0003 |
| A\_51\_P298325 | NM\_025820 | Mus musculus Crn, crooked neck-like 1 (Drosophila) (Crnkl1), mRNA | 2.60 | 0,0005 |
| A\_51\_P265343 | AF114379 | Mus musculus LIM domain binding 3 (Ldb3), mRNA | 2.60 | 0,0015 |
| A\_51\_P430973 | NM\_027995 | hypothetical Uncharacterised protein family Hly-III/UPF0073 containing protein | 2.60 | 0,0045 |
| A\_51\_P279384 | NM\_016893 | Mus musculus fucosyltransferase 8 (Fut8), mRNA | 2.60 | 0,0016 |
| A\_51\_P291936 | NM\_008060 | Mus musculus alpha glucosidase 2, alpha neutral subunit (G2an), mRNA | 2.58 | 0,0001 |
| A\_51\_P224406 | NM\_026441 | Mus musculus RIKEN cDNA 2600002E23 gene (2600002E23Rik), mRNA | 2.58 | 0,0004 |
| A\_51\_P192955 | BG921888 | CD166 ANTIGEN PRECURSOR (ACTIVATED LEUKOCYTE-CELL ADHESION MOLECULE) (ALCAM) (DM-GRASP PROTEIN) | 2.58 | 0,0022 |
| A\_51\_P105877 | NM\_201600 | Mus musculus myosin Vb (Myo5b), mRNA | 2.58 | 0,0054 |
| A\_51\_P469332 | AK034607 | unknown EST | 2.58 | 0,0001 |
| A\_51\_P131358 | NM\_009151 | Mus musculus selectin, platelet (p-selectin) ligand (Selpl), mRNA | 2.58 | 0,0004 |
| A\_51\_P406129 | AK019577 | unknown EST | 2.58 | 0,0004 |
| A\_51\_P440838 | NM\_026218 | Mus musculus RIKEN cDNA 1500031J01 gene (1500031J01Rik), mRNA | 2.58 | 0,0005 |
| A\_51\_P388039 | NM\_013915 | Mus musculus zinc finger protein 238 (Zfp238), mRNA | 2.58 | 0,0056 |
| A\_51\_P402398 | NM\_178741 | hypothetical BTB/POZ domain containing protein | 2.57 | 0,0003 |
| A\_51\_P159603 | NM\_207202 | hypothetical protein | 2.57 | 0,0007 |
| A\_51\_P485740 | NM\_026086 | Mus musculus RIKEN cDNA 1600031M04 gene (1600031M04Rik), mRNA | 2.57 | 0,0004 |
| A\_51\_P275123 | NM\_030263 | Mus musculus hypothetical protein, MGC:6957 (BC003498), mRNA | 2.57 | 0,0131 |
| A\_51\_P320876 | NM\_025996 | Mus musculus RIKEN cDNA 2610100K07 gene (2610100K07Rik), mRNA | 2.57 | 0,0012 |
| A\_51\_P193490 | NM\_145564 | SIMILAR TO F-BOX ONLY PROTEIN 21 homolog [Mus musculus] | 2.57 | 0,0001 |
| A\_51\_P483438 | NM\_011348 | Mus musculus sema domain, immunoglobulin domain (Ig), short basic domain, secreted, (semaphorin) 3E (Sema3e), mRNA | 2.57 | 0,0333 |
| A\_51\_P150905 | NM\_145479 | hypothetical BTB/POZ domain and hypothetical Kelch repeat containing protein | 2.57 | 0,0008 |
| A\_51\_P224362 | AK129115 | unknown EST | 2.55 | 0,0018 |
| A\_51\_P212741 | NM\_001014761 | unknown EST | 2.55 | 0,0008 |
| A\_51\_P248403 | NM\_010871 | Mus musculus baculoviral IAP repeat-containing 1f (Birc1f), mRNA | 2.53 | 0,0097 |
| A\_51\_P222337 | NM\_172815 | hypothetical Thrombospondin type I repeat (TSP1) profile/Thrombospondin type I domain/Furin-like cysteine rich region containing protein | 2.53 | 0,0160 |
| A\_51\_P311919 | NM\_008731 | Mus musculus neuropeptide Y receptor Y2 (Npy2r), mRNA | 2.53 | 0,0159 |
| A\_51\_P426566 | NM\_134067 | Mus musculus expressed sequence AW209491 (AW209491), mRNA | 2.53 | 0,0015 |
| A\_51\_P345775 | NM\_178934 | similar to CDNA FLJ31992 FIS, CLONE NT2RP7009149, WEAKLY SIMILAR TO GLUCOSE TRANSPORTER TYPE 2, LIVER [Homo sapiens] | 2.51 | 0,0464 |
| A\_51\_P437737 | NM\_020046 | Mus musculus dihydroorotate dehydrogenase (Dhodh), mRNA | 2.51 | 0,0000 |
| A\_51\_P408653 | NM\_145463 | hypothetical protein | 2.51 | 0,0348 |
| A\_51\_P468408 | NM\_027923 | hypothetical protein | 2.51 | 0,0008 |
| A\_51\_P191439 | NM\_178927 | hypothetical protein | 2.51 | 0,0150 |
| A\_51\_P231511 | NM\_145123 | ASPIC PRECURSOR homolog [Homo sapiens] | 2.51 | 0,0069 |
| A\_51\_P496400 | NM\_138675 | Mus musculus FLJ10193 (FLJ10193), mRNA | 2.51 | 0,0000 |
| A\_51\_P493771 | AK046785 | hypothetical Prenyl group binding site (CAAX box) containing protein | 2.50 | 0,0021 |
| A\_51\_P332742 | NM\_175347 | sarcoplasmic reticulum 53K glycoprotein precursor homolog [Oryctolagus cuniculus] | 2.50 | 0,0003 |
| A\_51\_P434269 | NM\_026610 | similar to NADH DEHYDROGENASE (UBIQUINONE) 1 BETA SUBCOMPLEX, 4 (15KD, B15) [Homo sapiens] | 2.50 | 0,0011 |
| A\_51\_P118188 | NM\_207222 | TRANSCRIPTION FACTOR XLMO1 homolog [Xenopus laevis] | 2.50 | 0,0002 |
| A\_51\_P316311 | AK173053 | protein tyrosine phosphatase, receptor type, f polypeptide (PTPRF), interacting protein (liprin), alpha 4 | 2.50 | 0,0065 |
| A\_51\_P456398 | NM\_020050 | Mus musculus RIKEN cDNA 2310004K06 gene (2310004K06Rik), mRNA | 2.50 | 0,0001 |
| A\_51\_P288009 | NM\_009604 | Mus musculus cholinergic receptor, nicotinic, gamma polypeptide (Chrng), mRNA | 2.50 | 0,0168 |
| A\_51\_P514300 | NM\_009447 | Mus musculus tubulin, alpha 4 (Tuba4), mRNA | 2.48 | 0,0009 |
| A\_51\_P459479 | BC058348 | hypothetical protein | 2.48 | 0,0182 |
| A\_51\_P187284 | NM\_008351 | Mus musculus interleukin 12a (Il12a), mRNA | 2.48 | 0,0192 |
| A\_51\_P152685 | NM\_175561 | weakly similar to PECANEX 1 [Mus musculus] | 2.48 | 0,0062 |
| A\_51\_P516095 | AK013508 | unknown EST | 2.48 | 0,0010 |
| A\_51\_P507043 | NM\_021560 | Mus musculus basic helix-loop-helix domain containing, class B5 (Bhlhb5), mRNA | 2.48 | 0,0028 |
| A\_51\_P296878 | NM\_173746 | hypothetical protein | 2.48 | 0,0001 |
| A\_51\_P414243 | NM\_153540 | hypothetical Fibronectin type III structure containing protein | 2.46 | 0,0004 |
| A\_51\_P515532 | NM\_029881 | hypothetical alpha/beta-Hydrolases structure containing protein | 2.46 | 0,0179 |
| A\_51\_P352402 | NM\_026149 | CHRONIC MYELOGENOUS LEUKEMIA TUMOR ANTIGEN 66 homolog [Homo sapiens] | 2.46 | 0,0011 |
| A\_51\_P218902 | BC042507 | unknown EST | 2.46 | 0,0007 |
| A\_51\_P119039 | NM\_016708 | Mus musculus neuropeptide Y receptor Y5 (Npy5r), mRNA | 2.46 | 0,0070 |
| A\_51\_P508705 | NM\_033561 | Mus musculus Williams-Beuren syndrome chromosome region 1 homolog (human) (Wbscr1), mRNA | 2.46 | 0,0000 |
| A\_51\_P345362 | NM\_001003912 | RHOGEF GLUTAMATE TRANSPORT MODULATOR GTRAP48 homolog [Rattus norvegicus] | 2.46 | 0,0003 |
| A\_51\_P352782 | AK017901 | unknown EST | 2.45 | 0,0100 |
| A\_51\_P231920 | NM\_001013374 | HYPOTHETICAL 39.7 KDA PROTEIN homolog [Homo sapiens] | 2.45 | 0,0025 |
| A\_51\_P178011 | AK220333 | hypothetical protein | 2.45 | 0,0006 |
| A\_51\_P449507 | NM\_008445 | Mus musculus kinesin family member 3c (Kif3c), mRNA | 2.45 | 0,0010 |
| A\_51\_P354572 | NM\_198163 | inferred: guanine nucleotide-binding protein ray {Homo sapiens} | 2.45 | 0,0001 |
| A\_51\_P395948 | NM\_153579 | synaptic vesicle glycoprotein 2 b | 2.45 | 0,0125 |
| A\_51\_P179831 | NM\_175003 | SH3 DOMAIN BINDING PROTEIN 1 3BP | 2.45 | 0,0003 |
| A\_51\_P269103 | NM\_021546 | amyloid beta (A4) precursor protein-binding, family A, member 1 binding protein | 2.45 | 0,0085 |
| A\_51\_P167313 | NM\_171824 | unknown EST | 2.43 | 0,0009 |
| A\_51\_P394635 | NM\_028940 | hypothetical Cellular retinaldehyde-binding protein (CRAL)/Triple function domain (TRIO) containing protein | 2.43 | 0,0160 |
| A\_51\_P390586 | NM\_027478 | hypothetical protein | 2.43 | 0,0007 |
| A\_51\_P372839 | NM\_172599 | hypothetical protein | 2.43 | 0,0024 |
| A\_51\_P182722 | NM\_016801 | Mus musculus syntaxin 1A (brain) (Stx1a), mRNA | 2.43 | 0,0084 |
| A\_51\_P393768 | XM\_148904 | OXYSTEROL BINDING PROTEIN | 2.43 | 0,0012 |
| A\_51\_P165934 | NM\_008831 | prohibitin | 2.43 | 0,0014 |
| A\_51\_P152489 | NM\_026388 | hypothetical protein | 2.43 | 0,0001 |
| A\_51\_P261184 | NM\_172403 | similar to ZINC FINGER PROTEIN (FRAGMENT) [Homo sapiens] | 2.43 | 0,0003 |
| A\_51\_P115471 | AK046043 | inferred: Similar to apolipoprotein L {Homo sapiens} | 2.41 | 0,0000 |
| A\_51\_P332169 | NM\_173390 | Mus musculus, clone IMAGE:3591967, mRNA, partial cds | 2.41 | 0,0002 |
| A\_51\_P121159 | NM\_023326 | B-MYC TRANSFORMING PROTEIN (FRAGMENT) homolog [Rattus norvegicus] | 2.41 | 0,0008 |
| A\_51\_P138199 | AK018228 | unclassifiable, full insert sequence. | 2.41 | 0,0004 |
| A\_51\_P246744 | NM\_021435 | Mus musculus hypothetical protein, MNCb-4414 (AB041549), mRNA | 2.41 | 0,0004 |
| A\_51\_P264634 | AK082675 | hypothetical protein | 2.41 | 0,0001 |
| A\_51\_P461364 | NM\_024198 | Mus musculus RIKEN cDNA 3110050F08 gene (3110050F08Rik), mRNA | 2.41 | 0,0050 |
| A\_51\_P246133 | NM\_010140 | Mouse eph-related receptor tyrosine kinase (Mek4) mRNA, complete cds | 2.39 | 0,0054 |
| A\_51\_P368755 | NM\_008069 | Mus musculus gamma-aminobutyric acid (GABA-A) receptor, subunit beta 1 (Gabrb1), mRNA | 2.39 | 0,0006 |
| A\_51\_P423465 | NM\_153098 | Mus musculus GPI-anchored alpha 2 macroglobulin-related protein (GARP), mRNA | 2.39 | 0,0261 |
| A\_51\_P408471 | NM\_026638 | Mus musculus, Similar to CDP-diacylglycerol--inositol 3-phosphatidyltransferase (phosphatidylinositol synthase), clone MGC:36425 IMAGE:5343411, mRNA, complete cds | 2.39 | 0,0001 |
| A\_51\_P418116 | NM\_146162 | Mus musculus, clone MGC:38046 IMAGE:5250899, mRNA, complete cds | 2.39 | 0,0017 |
| A\_51\_P209996 | M19413 | Mouse testicular alpha tubulin mRNA, 3 end | 2.39 | 0,0025 |
| A\_51\_P367240 | NM\_146173 | Mus musculus, Similar to transmembrane 4 superfamily member (tetraspan NET-7), clone MGC:30714 IMAGE:3981492, mRNA, complete cds | 2.39 | 0,0060 |
| A\_51\_P246092 | NM\_027074 | hypothetical protein | 2.39 | 0,0453 |
| A\_51\_P247153 | NM\_008258 | Mus musculus hematological and neurological expressed sequence 1 (Hn1), mRNA | 2.39 | 0,0007 |
| A\_51\_P318491 | NM\_145964 | Mus musculus, Similar to hypothetical protein PRO0971, clone MGC:7434 IMAGE:3489243, mRNA, complete cds | 2.39 | 0,0010 |
| A\_51\_P481788 | AK018549 | Mus musculus cDNA clone IMAGE:6490017 5 | 2.39 | 0,0094 |
| A\_51\_P161946 | NM\_175332 | hypothetical protein | 2.38 | 0,0035 |
| A\_51\_P292490 | AK012069 | unknown EST | 2.38 | 0,0001 |
| A\_51\_P313862 | NAP108668-1 | Mus musculus olfactory receptor MOR175-6 (MOR175-6) pseudogene | 2.38 | 0,0273 |
| A\_51\_P233928 | NM\_008080 | Mus musculus UDP-N-acetyl-alpha-D-galactosamine:(N-acetylneuraminyl)- galactosylglucosylceramide-beta-1, 4-N-acetylgalactosaminyltransferase (Galgt1), mRNA | 2.38 | 0,0047 |
| A\_51\_P177118 | NM\_001013375 | weakly similar to HYPOTHETICAL WD-REPEAT PROTEIN CGI-48 [Homo sapiens] | 2.38 | 0,0123 |
| A\_51\_P137094 | NM\_015772 | Mus musculus sal-like 2 (Drosophila) (Sall2), mRNA | 2.38 | 0,0027 |
| A\_51\_P143468 | NM\_178771 | CDNA FLJ11078 FIS, CLONE PLACE1005102, WEAKLY SIMILAR TO RING CANAL PROTEIN homolog [Homo sapiens] | 2.38 | 0,0065 |
| A\_51\_P170795 | NM\_178739 | hypothetical G-protein beta WD-40 repeats containing protein | 2.36 | 0,0024 |
| A\_51\_P384993 | NM\_175481 | GLUTAMATE RECEPTOR, IONOTROPIC KAINATE 4 PRECURSOR (GLUTAMATE RECEPTOR KA-1) (KA1) homolog [Rattus norvegicus] | 2.36 | 0,0348 |
| A\_51\_P202084 | NM\_008073 | Mus musculus gamma-aminobutyric acid (GABA-A) receptor, subunit gamma 2 (Gabrg2), mRNA | 2.36 | 0,0009 |
| A\_51\_P497350 | NM\_011464 | Mus musculus hepatocyte growth factor activator inhibitor type 2 (Hai2) mRNA, complete cds | 2.36 | 0,0044 |
| A\_51\_P388298 | NM\_025962 | Mus musculus RIKEN cDNA 1810037K07 gene (1810037K07Rik), mRNA | 2.36 | 0,0003 |
| A\_51\_P319213 | NM\_175406 | weakly similar to VACUOLAR ATP SYNTHASE SUBUNIT D (EC 3.6.3.14) (V-ATPASE D SUBUNIT) (VACUOLAR PROTON PUMP D SUBUNIT) (V-ATPASE AC39 SUBUNIT) (V-ATPASE 40 KDA ACCESSORY PROTEIN) (P39) (PHYSOPHILIN) [Mus musculus] | 2.36 | 0,0313 |
| A\_51\_P290886 | NM\_007667 | Mus musculus cadherin 8 (Cdh8), mRNA | 2.36 | 0,0004 |
| A\_51\_P241262 | NM\_153820 | similar to UNCHARACTERIZED BONE MARROW PROTEIN BM046 [Homo sapiens] | 2.35 | 0,0121 |
| A\_51\_P221189 | NM\_178622 | hypothetical protein | 2.35 | 0,0019 |
| A\_51\_P303217 | NM\_026754 | hypothetical protein | 2.35 | 0,0090 |
| A\_51\_P421244 | NM\_026662 | Mus musculus RIKEN cDNA 2610101M19 gene (2610101M19Rik), mRNA | 2.35 | 0,0004 |
| A\_51\_P124951 | NM\_025296 | Mus musculus WD40 protein Ciao1 (Ciao1-pending), mRNA | 2.35 | 0,0000 |
| A\_51\_P309494 | NM\_138602 | Mus musculus DNA segment, Chr X, Immunex 39, expressed (DXImx39e), mRNA | 2.35 | 0,0001 |
| A\_51\_P224424 | NM\_172781 | KELCH-LIKE PROTEIN 4 homolog [Homo sapiens] | 2.35 | 0,0143 |
| A\_51\_P109709 | NM\_146750 | Mus musculus olfactory receptor MOR40-11 (MOR40-11) pseudogene | 2.35 | 0,0106 |
| A\_51\_P454152 | AK048416 | hypothetical Winged helix DNA-binding domain structure containing protein | 2.33 | 0,0050 |
| A\_51\_P437549 | NM\_144876 | KELCH-LIKE PROTEIN X homolog [Homo sapiens] | 2.33 | 0,0005 |
| A\_51\_P476687 | NM\_008527 | Mus musculus killer cell lectin-like receptor subfamily B member 1C (Klrb1c), mRNA | 2.33 | 0,0440 |
| A\_51\_P434497 | NM\_026981 | Mus musculus, RIKEN cDNA 1810033A06 gene, clone MGC:28242 IMAGE:3993232, mRNA, complete cds | 2.33 | 0,0017 |
| A\_51\_P237856 | NM\_008328 | Mus musculus interferon activated gene 203 (Ifi203), mRNA | 2.33 | 0,0069 |
| A\_51\_P436957 | NM\_025799 | Mus musculus RIKEN cDNA 0610025O11 gene (0610025O11Rik), mRNA | 2.33 | 0,0002 |
| A\_51\_P207570 | NM\_030064 | PHD-finger/EGF-like domain containing protein | 2.33 | 0,0008 |
| A\_51\_P262101 | NM\_026873 | hypothetical Prokaryotic membrane lipoprotein lipid attachment site/PPR repeats containing protein | 2.33 | 0,0018 |
| A\_51\_P201607 | XM\_620331 | RING FINGER PROTEIN WITH LEUCINE ZIPPER RNF26 (RESERVED) homolog [Homo sapiens] | 2.33 | 0,0006 |
| A\_51\_P403793 | NM\_021287 | Mus musculus beta III spectrin (Spnb3) mRNA, partial cds | 2.33 | 0,0086 |
| A\_51\_P371408 | NM\_029842 | similar to THYRO1000124 PROTEIN [Homo sapiens] | 2.31 | 0,0474 |
| A\_51\_P221100 | NM\_198214 | KIAA0374 (SYNTAPHILIN) (BA314N13.1.1) homolog [Homo sapiens] | 2.31 | 0,0087 |
| A\_51\_P140742 | NM\_012043 | Mus musculus immunoglobulin superfamily containing leucine-rich repeat (Islr), mRNA | 2.31 | 0,0011 |
| A\_51\_P227275 | NM\_007786 | Mus musculus casein kappa (Csnk), mRNA | 2.31 | 0,0231 |
| A\_51\_P431046 | NM\_009182 | Mus musculus sialyltransferase 8 (alpha-2, 8-sialytransferase) C (Siat8c), mRNA | 2.31 | 0,0044 |
| A\_51\_P392776 | XM\_622789 | hypothetical G-protein beta WD-40 repeats containing protein | 2.31 | 0,0014 |
| A\_51\_P386069 | NM\_176971 | RAS-RELATED PROTEIN RAB-9L (RAB9-LIKE PROTEIN) homolog [Homo sapiens] | 2.31 | 0,0020 |
| A\_51\_P388822 | NM\_174998 | HIPPOCALCIN-LIKE PROTEIN 4 (HYPOTHETICAL 22.2 KDA PROTEIN) homolog [Homo sapiens] | 2.31 | 0,0361 |
| A\_51\_P462159 | NM\_134096 | Mus musculus expressed sequence AW049604 (AW049604), mRNA | 2.31 | 0,0007 |
| A\_51\_P210082 | NM\_015769 | Mus musculus excision repair cross-complementing rodent repair deficiency, complementation group 4 (Ercc4), mRNA | 2.31 | 0,0014 |
| A\_51\_P501963 | NM\_021463 | Mus musculus phosphoribosyl pyrophosphate synthetase 1 (Prps1), mRNA | 2.31 | 0,0002 |
| A\_51\_P105515 | NM\_026053 | GEMIN6 homolog [Homo sapiens] | 2.31 | 0,0002 |
| A\_51\_P236324 | NM\_028812 | general transcription factor IIE, polypeptide 1 (alpha subunit, 56kDa) | 2.31 | 0,0061 |
| A\_51\_P480390 | NM\_008579 | Mus musculus meiosis expressed gene 1 (Meg1), mRNA | 2.30 | 0,0006 |
| A\_51\_P326994 | NM\_133746 | Mus musculus RIKEN cDNA 2810048G17 gene (2810048G17Rik), mRNA | 2.30 | 0,0019 |
| A\_51\_P192162 | BC022654 | unknown EST | 2.30 | 0,0139 |
| A\_51\_P128775 | AK008890 | unknown EST | 2.30 | 0,0011 |
| A\_51\_P376149 | NM\_026526 | Mus musculus RIKEN cDNA 2510005D08 gene (2510005D08Rik), mRNA | 2.30 | 0,0013 |
| A\_51\_P337210 | NM\_138682 | Mus musculus LIBG-like protein (MBAG1), mRNA | 2.30 | 0,0019 |
| A\_51\_P251205 | NM\_028627 | hypothetical protein | 2.30 | 0,0189 |
| A\_51\_P174512 | AK078076 | hypothetical Sushi domain / SCR repeat / CCP module containing protein | 2.28 | 0,0005 |
| A\_51\_P177242 | NM\_021468 | Mus musculus unc13 homolog (C. elegans) 1 (Unc13h1), mRNA | 2.28 | 0,0001 |
| A\_51\_P269663 | NM\_010585 | Mus musculus inositol 1,4,5-triphosphate receptor 1 (Itpr1), mRNA | 2.28 | 0,0178 |
| A\_51\_P448881 | NM\_145528 | Mus musculus, clone MGC:6816 IMAGE:2648797, mRNA, complete cds | 2.28 | 0,0005 |
| A\_51\_P120017 | BC057029 | Mus musculus, Similar to cyclic GMP stimulated phosphodiesterase, clone IMAGE:3598413, mRNA, partial cds | 2.28 | 0,0009 |
| A\_51\_P394244 | XM\_484075 | inferred: Link guanine nucleotide exchange factor II {Homo sapiens} | 2.28 | 0,0296 |
| A\_51\_P509551 | L42339 | Mus musculus sodium channel 3 mRNA, complete cds | 2.28 | 0,0016 |
| A\_51\_P240041 | NM\_011458 | Mouse mRNA for contrapsin | 2.28 | 0,0306 |
| A\_51\_P397834 | NM\_010072 | dolichol-phosphate (beta-D) mannosyltransferase 1 | 2.28 | 0,0434 |
| A\_51\_P216215 | NM\_007982 | Mus musculus PTK2 protein tyrosine kinase 2 (Ptk2), mRNA | 2.28 | 0,0001 |
| A\_51\_P289464 | AK029184 | DJ631M13.5.2 (NOVEL PROTEIN (ISOFORM 2)) homolog [Homo sapiens] | 2.28 | 0,0008 |
| A\_51\_P141610 | BC052789 | similar to CDK3-BINDING PROTEIN IK3-1 [Mus musculus] | 2.28 | 0,0003 |
| A\_51\_P380750 | NM\_009824 | "Mus musculus core-binding factor, runt domain, alpha subunit 2� translocated to, 3 homolog (human) (Cbfa2t3h), mRNA" | 2.27 | 0,0342 |
| A\_51\_P369862 | NM\_178772 | ARYLACETAMIDE DEACETYLASE EC 3.1.1.- | 2.27 | 0,0004 |
| A\_51\_P308308 | NM\_172958 | PHOSPHATIDYLINOSITOL-3 PHOSPHATE 3-PHOSPHATASE ADAPTOR SUBUNIT homolog [Homo sapiens] | 2.27 | 0,0010 |
| A\_51\_P314143 | AB045325 | Mus musculus expressed sequence AI415388 (AI415388), mRNA | 2.27 | 0,0090 |
| A\_51\_P101474 | NM\_015799 | Mus musculus transferrin receptor 2 (Trfr2), mRNA | 2.27 | 0,0152 |
| A\_51\_P386090 | D50418 | Mouse mRNA for AREC3, partial cds | 2.27 | 0,0475 |
| A\_51\_P252107 | NM\_173748 | CDNA FLJ14164 FIS, CLONE NT2RP1000460, WEAKLY SIMILAR TO NUCLEAR MOVEMENT PROTEIN NUDC homolog [Homo sapiens] | 2.25 | 0,0023 |
| A\_51\_P148216 | NM\_020025 | Mus musculus UDP-Gal:betaGlcNAc beta 1,3-galactosyltransferase, polypeptide 2 (B3galt2), mRNA | 2.25 | 0,0420 |
| A\_51\_P406478 | TC1461965 | weakly similar to TUMOR ENDOTHELIAL MARKER 4 [Homo sapiens] | 2.25 | 0,0014 |
| A\_51\_P426555 | NM\_133991 | Mus musculus expressed sequence AI931847 (AI931847), mRNA | 2.25 | 0,0001 |
| A\_51\_P148388 | NM\_017477 | Mus musculus coatomer protein complex, subunit gamma 1 (Copg1), mRNA | 2.25 | 0,0011 |
| A\_51\_P114238 | BC029786 | Mus musculus cDNA, 5 end | 2.25 | 0,0005 |
| A\_51\_P275827 | NM\_144887 | hypothetical DHHC-type Zn-finger containing protein | 2.25 | 0,0131 |
| A\_51\_P170987 | NM\_029879 | hypothetical protein | 2.25 | 0,0060 |
| A\_51\_P246895 | BC019787 | hypothetical Ribosome recycling factor containing protein | 2.25 | 0,0048 |
| A\_51\_P320843 | NM\_025921 | Mus musculus RIKEN cDNA 2610002M06 gene (2610002M06Rik), mRNA | 2.25 | 0,0002 |
| A\_51\_P474709 | AK008216 | unknown EST | 2.23 | 0,0008 |
| A\_51\_P343851 | NM\_001013025 | TGF BETA RECEPTOR ASSOCIATED PROTEIN | 2.23 | 0,0004 |
| A\_51\_P342906 | NM\_009164 | Mus musculus SH3-domain binding protein 1 (Sh3bp1), mRNA | 2.23 | 0,0014 |
| A\_51\_P300297 | NM\_026075 | Mus musculus RIKEN cDNA 3110031B13 gene (3110031B13Rik), mRNA | 2.23 | 0,0019 |
| A\_51\_P237834 | AK028322 | hypothetical protein | 2.23 | 0,0018 |
| A\_51\_P310850 | BC025841 | Mus musculus, clone IMAGE:5149318, mRNA, partial cds | 2.23 | 0,0038 |
| A\_51\_P503297 | NM\_177882 | weakly similar to finger protein (clone XlcOF6.1) (fragment) [Xenopus laevis] | 2.23 | 0,0196 |
| A\_51\_P421300 | AK002979 | D1 DOPAMINE RECEPTOR-INTERACTING PROTEIN CALCYON homolog [Mus musculus] | 2.23 | 0,0148 |
| A\_51\_P133763 | NM\_175642 | DJ91B17.1 (BRAIN-SPECIFIC ANGIOGENESIS INHIBITOR 3) (FRAGMENT) homolog [Homo sapiens] | 2.23 | 0,0229 |
| A\_51\_P144601 | NM\_017379 | Mus musculus tubulin, alpha 8 (Tuba8), mRNA | 2.23 | 0,0040 |
| A\_51\_P371241 | AK173333 | unknown EST | 2.23 | 0,0011 |
| A\_51\_P283264 | AK079330 | similar to KRUPPEL-ASSOCIATED BOX PROTEIN [Homo sapiens] | 2.23 | 0,0001 |
| A\_51\_P143951 | NM\_007495 | Mus musculus astrotactin 1 (Astn1), mRNA | 2.23 | 0,0011 |
| A\_51\_P326419 | NM\_029802 | Mus musculus, RIKEN cDNA 2310002N04 gene, clone MGC:7909 IMAGE:3583149, mRNA, complete cds | 2.23 | 0,0080 |
| A\_51\_P454913 | AK085143 | SIMILAR TO VESICLE-ASSOCIATED CALMODULIN-BINDING PROTEIN homolog [Mus musculus] | 2.23 | 0,0036 |
| A\_51\_P329811 | NM\_009042 | Mus musculus regenerating islet-derived 1 (Reg1), mRNA | 2.22 | 0,0250 |
| A\_51\_P180061 | NM\_028717 | Mus musculus amyotrophic lateral sclerosis 2 (juvenile) homolog (human) (Als2), mRNA | 2.22 | 0,0266 |
| A\_51\_P442782 | NM\_080419 | Mus musculus immunoglobulin superfamily receptor PGRL (Pgrl) mRNA, complete cds | 2.22 | 0,0040 |
| A\_51\_P235426 | NM\_178194 | H2B histone family, member S | 2.22 | 0,0126 |
| A\_51\_P375453 | NM\_139298 | WNT14 homolog [Homo sapiens] | 2.22 | 0,0038 |
| A\_51\_P318933 | NM\_007595 | Mus musculus calcium/calmodulin-dependent protein kinase II, beta (Camk2b), mRNA | 2.22 | 0,0268 |
| A\_51\_P144285 | BC058403 | ribosomal S6 protein kinase {Homo sapiens} | 2.22 | 0,0002 |
| A\_51\_P309534 | NM\_019781 | Mus musculus peroxisomal biogenesis factor 14 (Pex14), mRNA | 2.22 | 0,0000 |
| A\_51\_P214516 | NM\_008698 | Mus musculus 4-nitrophenylphosphatase domain and non-neuronal SNAP25-like protein homolog 1 (C. elegans) (Nipsnap1), mRNA | 2.22 | 0,0013 |
| A\_51\_P276598 | NM\_133886 | Mus musculus, Similar to hypothetical protein FLJ14225, clone IMAGE:5132616, mRNA, partial cds | 2.22 | 0,0004 |
| A\_51\_P132044 | NM\_013597 | Mus musculus myocyte enhancer factor 2A (Mef2a), mRNA | 2.22 | 0,0005 |
| A\_51\_P484200 | NM\_153777 | hypothetical Leucine-rich repeat containing protein | 2.22 | 0,0007 |
| A\_51\_P442894 | AK048310 | ALDEHYDE DEHYDROGENASE, DIMERIC NADP PREFERRING EC 1.2.1.5 ALDH CLASS 3 | 2.20 | 0,0066 |
| A\_51\_P275101 | AK003880 | hypothetical Histidine-rich region containing protein | 2.20 | 0,0083 |
| A\_51\_P122085 | NM\_028846 | ubiquitin specific protease 20 | 2.20 | 0,0040 |
| A\_51\_P226465 | NM\_145579 | Mus musculus Myb protein P42POP mRNA, complete cds | 2.20 | 0,0029 |
| A\_51\_P436817 | BC052075 | similar to R29144\_1 [Homo sapiens] | 2.20 | 0,0091 |
| A\_51\_P156526 | AK032322 | PLASMA MEMBRANE CALCIUM-TRANSPORTING ATPASE 3 (EC 3.6.3.8) (PMCA3) (PLASMA MEMBRANE CALCIUM PUMP ISOFORM 3) (PLASMA MEMBRANE CALCIUM ATPASE ISOFORM 3) homolog [Rattus norvegicus] | 2.20 | 0,0030 |
| A\_51\_P246124 | NM\_010113 | Mus musculus epidermal growth factor (Egf), mRNA | 2.20 | 0,0162 |
| A\_51\_P369154 | NM\_144926 | SIMILAR TO TYPE I TRANSMEMBRANE RECEPTOR (SEIZURE-RELATED PROTEIN) homolog [Mus musculus] | 2.20 | 0,0007 |
| A\_51\_P182443 | AK173016 | TRANSLATION INITIATION FACTOR IF-2 homolog [Homo sapiens] | 2.20 | 0,0002 |
| A\_51\_P260499 | NM\_183019 | similar to APC-STIMULATED GUANINE NUCLEOTIDE EXCHANGE FACTOR [Homo sapiens] | 2.20 | 0,0030 |
| A\_51\_P286946 | NM\_029609 | PHOSPHOLYSINE PHOSPHOHISTIDINE INORGANIC PYROPHOSPHATE PHOSPHATASE homolog [Homo sapiens] | 2.20 | 0,0007 |
| A\_51\_P443976 | NM\_011217 | Mus musculus protein tyrosine phosphatase, receptor type, R (Ptprr), mRNA | 2.20 | 0,0125 |
| A\_51\_P411645 | NM\_021500 | Mus musculus macrophage erythroblast attacher (Maea), mRNA | 2.20 | 0,0322 |
| A\_51\_P499254 | BC027371 | hypothetical Nucleic acid-binding proteins structure containing protein | 2.20 | 0,0135 |
| A\_51\_P244969 | NM\_025346 | Mus musculus, clone MGC:27569 IMAGE:4485143, mRNA, complete cds | 2.20 | 0,0005 |
| A\_51\_P269084 | NM\_175329 | similar to N27C7-4 PROTEIN [Homo sapiens] | 2.20 | 0,0028 |
| A\_51\_P281586 | NM\_033569 | Mus musculus cyclin M2 (Cnnm2), mRNA | 2.20 | 0,0006 |
| A\_51\_P437079 | NM\_028872 | hypothetical protein | 2.19 | 0,0495 |
| A\_51\_P331886 | AK038316 | inferred: human CLASP-5 {Homo sapiens} | 2.19 | 0,0296 |
| A\_51\_P355765 | AK019479 | hypothetical protein | 2.19 | 0,0349 |
| A\_51\_P389957 | NM\_016758 | Mus musculus regulator of G-protein signaling 14 (Rgs14), mRNA | 2.19 | 0,0403 |
| A\_51\_P501550 | NM\_026430 | UDP-GLUCURONIC ACID DECARBOXYLASE homolog [Mus musculus] | 2.19 | 0,0049 |
| A\_51\_P229499 | NM\_027452 | hypothetical Immunoglobulin and major histocompatibility complex domain containing protein | 2.19 | 0,0177 |
| A\_51\_P378978 | NM\_013496 | Mus musculus cellular retinoic acid binding protein I (Crabp1), mRNA | 2.19 | 0,0034 |
| A\_51\_P159962 | NM\_010141 | M.musculus mRNA for kinase 1 | 2.19 | 0,0319 |
| A\_51\_P479043 | NM\_022988 | Mus musculus Ngg1 interacting factor 3-like 1 (S. pombe) (Nif3l1), mRNA | 2.19 | 0,0034 |
| A\_51\_P311075 | A\_51\_P311075 | glycerol-3-phosphate acyltransferase, mitochondrial | 2.19 | 0,0458 |
| A\_51\_P431023 | BC027561 | Mus musculus, Similar to ubiquitin associated and SH3 domain containing, A, clone IMAGE:3375785, mRNA | 2.19 | 0,0411 |
| A\_51\_P455421 | NM\_013871 | Mus musculus mitogen-activated protein kinase 12 (Mapk12), mRNA | 2.19 | 0,0446 |
| A\_51\_P154596 | NM\_008721 | Mus musculus neural proliferation, differentiation and control gene 1 (Npdc1), mRNA | 2.19 | 0,0004 |
| A\_51\_P103594 | AK020485 | hypothetical protein | 2.19 | 0,0059 |
| A\_51\_P199580 | NM\_028185 | hypothetical Small nuclear ribonucleoprotein (Sm protein) containing protein | 2.19 | 0,0195 |
| A\_51\_P267447 | NM\_009616 | Mus musculus a disintegrin and metalloproteinase domain 19 (meltrin beta) (Adam19), mRNA | 2.19 | 0,0086 |
| A\_51\_P237599 | NM\_009691 | Mus musculus amyloid beta (A4) precursor-like protein 2 (Aplp2), mRNA | 2.17 | 0,0011 |
| A\_51\_P367741 | XM\_112129 | hypothetical protein | 2.17 | 0,0158 |
| A\_51\_P419116 | NM\_134050 | Mus musculus RIKEN cDNA 2310012G06 gene (2310012G06Rik), mRNA | 2.17 | 0,0202 |
| A\_51\_P221062 | AK081805 | protein kinase, cAMP dependent regulatory, type II beta | 2.17 | 0,0335 |
| A\_51\_P185332 | AK004510 | unknown EST | 2.17 | 0,0033 |
| A\_51\_P226542 | NM\_173450 | hypothetical Pseudouridine synthase containing protein | 2.17 | 0,0034 |
| A\_51\_P488768 | NM\_178696 | hypothetical Mitochondrial energy transfer proteins (carrier protein) containing protein | 2.17 | 0,0011 |
| A\_51\_P202592 | NM\_172628 | hypothetical Tetratricopeptide repeat (TPR) structure containing protein | 2.17 | 0,0354 |
| A\_51\_P195439 | NM\_025991 | Mus musculus RIKEN cDNA 2510026C23 gene (2510026C23Rik), mRNA | 2.17 | 0,0016 |
| A\_51\_P234373 | NM\_012035 | Mus musculus transient receptor protein 8 (Trrp8), mRNA | 2.17 | 0,0047 |
| A\_51\_P142989 | AK083314 | weakly similar to KRAB ZINC FINGER PROTEIN [Mus musculus] | 2.17 | 0,0266 |
| A\_51\_P486512 | NM\_134093 | Mus musculus expressed sequence AI593524 (AI593524), mRNA | 2.17 | 0,0023 |
| A\_51\_P350706 | NM\_178613 | HSPC210 homolog [Homo sapiens] | 2.17 | 0,0049 |
| A\_51\_P217115 | NM\_176834 | inferred: hypothetical protein {Homo sapiens} | 2.17 | 0,0052 |
| A\_51\_P408172 | XM\_356184 | hypothetical Integrin A (or I) domain structure containing protein | 2.17 | 0,0310 |
| A\_51\_P442016 | AK050846 | DEPENDENT PROTEIN KINASE KINASE | 2.16 | 0,0131 |
| A\_51\_P486449 | NM\_133910 | Mus musculus expressed sequence AU043625 (AU043625), mRNA | 2.16 | 0,0007 |
| A\_51\_P446085 | NM\_028908 | hypothetical Serine-rich region containing protein | 2.16 | 0,0173 |
| A\_51\_P459465 | AK083260 | KIDINS220 homolog [Rattus norvegicus] | 2.16 | 0,0084 |
| A\_51\_P502919 | NAP057073-1 | Mus musculus olfactory receptor GA\_x5J8B7W6KF8-5546505-5545716 (GA\_x5J8B7W6KF8-5546505-5545716) pseudogene | 2.16 | 0,0085 |
| A\_51\_P116421 | NM\_013790 | Mus musculus ATP-binding cassette, sub-family C (CFTR/MRP), member 5a (Abcc5a), mRNA | 2.16 | 0,0002 |
| A\_51\_P303728 | NM\_172575 | ZINC FINGER PROTEIN 277 homolog [Homo sapiens] | 2.16 | 0,0001 |
| A\_51\_P200408 | BC003939 | Mus musculus RIKEN cDNA 2810021O11 gene (2810021O11Rik), mRNA | 2.16 | 0,0006 |
| A\_51\_P188574 | XM\_146997 | weakly similar to MDC-3.13 ISOFORM 2 (TNF-INDUCED PROTEIN) [Homo sapiens] | 2.16 | 0,0015 |
| A\_51\_P112100 | AK081899 | hypothetical protein | 2.16 | 0,0003 |
| A\_51\_P141012 | AK173296 | hypothetical Cysteine-rich flanking region, C-terminal/Leucine-rich repeat/Leucine-rich repeat, typical subtype containing protein | 2.14 | 0,0155 |
| A\_51\_P491886 | NM\_009428 | Mus musculus transient receptor protein 5 (Trrp5), mRNA | 2.14 | 0,0070 |
| A\_51\_P407683 | NM\_145540 | hypothetical Glycine-rich region/Immunoglobulin and major histocompatibility complex domain containing protein | 2.14 | 0,0020 |
| A\_51\_P138671 | NM\_175244 | Mus musculus cDNA, 3 end | 2.14 | 0,0008 |
| A\_51\_P452637 | XM\_355470 | Similar to pyruvate dehydrogenase phosphatase, clone IMAGE:6492665, mRNA | 2.14 | 0,0356 |
| A\_51\_P472588 | NM\_001015876 | similar to CDNA FLJ12855 FIS, CLONE NT2RP2003506, WEAKLY SIMILAR TO NADPH-CYTOCHROME P450 REDUCTASE (EC 1.6.2.4) [Homo sapiens] | 2.14 | 0,0023 |
| A\_51\_P254864 | AK122311 | Mus musculus optic atrophy 1 homolog (human) (Opa1), mRNA | 2.14 | 0,0016 |
| A\_51\_P207153 | NM\_198001 | Mus musculus, Similar to chromosome 9 open reading frame 16, clone MGC:19388 IMAGE:2812475, mRNA, complete cds | 2.14 | 0,0099 |
| A\_51\_P421882 | NM\_029580 | Mus musculus, RIKEN cDNA 2610027O18 gene, clone MGC:6806 IMAGE:2648293, mRNA, complete cds | 2.14 | 0,0011 |
| A\_51\_P196207 | NM\_029341 | weakly similar to CALCYPHOSINE-LIKE PROTEIN [Manduca sexta] | 2.14 | 0,0117 |
| A\_51\_P520879 | NM\_012059 | Mus musculus SH3 domain protein D19 (Sh3d19), mRNA | 2.14 | 0,0119 |
| A\_51\_P201520 | BC063105 | CCR4-NOT transcription complex, subunit 2 | 2.14 | 0,0015 |
| A\_51\_P384159 | NM\_001010826 | Mus musculus, Similar to hypothetical protein MGC2376, clone MGC:37717 IMAGE:5066294, mRNA, complete cds | 2.14 | 0,0259 |
| A\_51\_P153556 | A\_51\_P153556 | hypothetical Actin-like ATPase domain structure containing protein | 2.14 | 0,0198 |
| A\_51\_P510485 | BC046305 | Mus musculus, clone IMAGE:4191452, mRNA, partial cds | 2.14 | 0,0350 |
| A\_51\_P364391 | NM\_175184 | hypothetical protein | 2.14 | 0,0027 |
| A\_51\_P228817 | NM\_025303 | Mus musculus staufen (RNA binding protein) homolog 2 (Drosophila) (Stau2), mRNA | 2.14 | 0,0015 |
| A\_51\_P389977 | NM\_015782 | Mus musculus small nuclear ribonucleoprotein polypeptide A (Snrpa), mRNA | 2.14 | 0,0001 |
| A\_51\_P379552 | XM\_132047 | unknown EST | 2.13 | 0,0079 |
| A\_51\_P370163 | NM\_009431 | Mus musculus TPR-containing, SH2-binding phosphoprotein (Tsbp), mRNA | 2.13 | 0,0014 |
| A\_51\_P265016 | NM\_023249 | Mus musculus peptidylprolyl isomerase (cyclophilin)-like 2 (Ppil2), mRNA | 2.13 | 0,0087 |
| A\_51\_P133005 | TC1516362 | AMINO ACID TRANSPORTER NAT-2 homolog [Mus musculus] | 2.13 | 0,0007 |
| A\_51\_P184806 | NM\_178736 | weakly similar to HYPOTHETICAL 23.0 KDA PROTEIN (FRAGMENT) [Homo sapiens] | 2.13 | 0,0006 |
| A\_51\_P396331 | NM\_013681 | Mus musculus synapsin II (Syn2), mRNA | 2.13 | 0,0072 |
| A\_51\_P391634 | NM\_144865 | Mus musculus, clone MGC:28827 IMAGE:4505679, mRNA, complete cds | 2.13 | 0,0013 |
| A\_51\_P192782 | AK003900 | hypothetical protein | 2.13 | 0,0028 |
| A\_51\_P466858 | AK079284 | weakly similar to HYPOTHETICAL 70.3 KDA PROTEIN (FRAGMENT) [Homo sapiens] | 2.13 | 0,0014 |
| A\_51\_P130773 | NM\_009613 | Mus musculus a disintegrin and metalloprotease domain 11 (Adam11), mRNA | 2.13 | 0,0187 |
| A\_51\_P514270 | AK013768 | unknown EST | 2.13 | 0,0146 |
| A\_51\_P386025 | AK009987 | unknown EST | 2.13 | 0,0005 |
| A\_51\_P447248 | NM\_175105 | hypothetical Eukaryotic thiol (cysteine) proteases active site containing protein | 2.13 | 0,0030 |
| A\_51\_P490747 | AK031845 | Mus musculus cDNA, 3 end | 2.13 | 0,0201 |
| A\_51\_P356962 | NM\_145219 | weakly similar to LEUCINE-RICH GLIOMA-INACTIVATED 1 PROTEIN [Mus musculus] | 2.13 | 0,0158 |
| A\_51\_P373609 | NM\_198246 | CDNA FLJ13995 FIS, CLONE Y79AA1002209, WEAKLY SIMILAR TO TYROSYL-TRNA SYNTHETASE (EC 6.1.1.1) (HYPOTHETICAL 53.2 KDA PROTEIN) homolog [Homo sapiens] | 2.13 | 0,0006 |
| A\_51\_P518666 | NM\_133704 | Mus musculus RIKEN cDNA 1810005C06 gene (1810005C06Rik), mRNA | 2.13 | 0,0047 |
| A\_51\_P446003 | NM\_013764 | Mus musculus deoxyguanosine kinase (Dguok), mRNA | 2.13 | 0,0019 |
| A\_51\_P350214 | TC1431688 | Mus musculus cDNA, 5 end | 2.13 | 0,0006 |
| A\_51\_P260789 | AK039297 | weakly similar to PUTATIVE RNA-BINDING PROTEIN 11 (RNA BINDING MOTIF PROTEIN 11) [Homo sapiens] | 2.13 | 0,0010 |
| A\_51\_P265219 | NM\_175523 | unclassifiable | 2.13 | 0,0072 |
| A\_51\_P255565 | NM\_007958 | M.musculus mRNA of enhancer-trap-locus 1 | 2.13 | 0,0010 |
| A\_51\_P278136 | NM\_028215 | hypothetical protein | 2.13 | 0,0001 |
| A\_51\_P247180 | AK030974 | hypothetical Tetratricopeptide repeat (TPR) structure containing protein | 2.11 | 0,0003 |
| A\_51\_P484653 | NM\_022022 | Mus musculus ubiquitination factor E4B, UFD2 homolog (S. cerevisiae) (Ube4b), mRNA | 2.11 | 0,0012 |
| A\_51\_P478952 | NM\_133898 | Mus musculus expressed sequence AI428195 (AI428195), mRNA | 2.11 | 0,0014 |
| A\_51\_P206268 | BC027244 | SEC13-LIKE PROTEIN homolog [Homo sapiens] | 2.11 | 0,0042 |
| A\_51\_P412966 | NM\_008516 | Mus musculus leucine rich repeat protein 1, neuronal (Lrrn1), mRNA | 2.11 | 0,0112 |
| A\_51\_P511734 | NM\_175246 | DJ423B22.2 (NOVEL PROTEIN SIMILAR TO C.ELEGANS PROTEIN CE08529) (FRAGMENT) homolog [Homo sapiens] | 2.11 | 0,0060 |
| A\_51\_P349546 | NM\_153098 | weakly similar to alpha-2-macroglobulin (fragments) [Limulus polyphemus] | 2.11 | 0,0051 |
| A\_51\_P398887 | NM\_031251 | cystinosis, nephropathic | 2.11 | 0,0008 |
| A\_51\_P460768 | AK089959 | hypothetical SET domain profile/MYND zinc finger (ZnF) domain/Cytochrome c family heme-binding site containing protein | 2.11 | 0,0037 |
| A\_51\_P394735 | NM\_133962 | Mus musculus expressed sequence AI467246 (AI467246), mRNA | 2.11 | 0,0002 |
| A\_51\_P160576 | AK014760 | unknown EST | 2.10 | 0,0026 |
| A\_51\_P256747 | NM\_173029 | SOLUBLE ADENYLYL CYCLASE homolog [Rattus norvegicus] | 2.10 | 0,0298 |
| A\_51\_P420122 | NM\_178631 | weakly similar to SIMILAR TO HETEROGENEOUS NUCLEAR RIBONUCLEOPROTEIN C [Homo sapiens] | 2.10 | 0,0092 |
| A\_51\_P323531 | AK006229 | hypothetical protein | 2.10 | 0,0137 |
| A\_51\_P437882 | NM\_016793 | Mus musculus zinc finger protein 98 (Zfp98), mRNA | 2.10 | 0,0003 |
| A\_51\_P408460 | AK086518 | unknown EST | 2.10 | 0,0178 |
| A\_51\_P395686 | AK017899 | similar to RIBOSOMAL PROTEIN S6 (FRAGMENT) [Sus scrofa] | 2.10 | 0,0104 |
| A\_51\_P431491 | BC037132 | similar to EFERIN [Homo sapiens] | 2.10 | 0,0030 |
| A\_51\_P168219 | NM\_026401 | Mus musculus mitochondrial ribosomal protein 63 (Mrp63), mRNA | 2.10 | 0,0089 |
| A\_51\_P293017 | AK045075 | MYO INOSITOL MONOPHOSPHATASE | 2.10 | 0,0005 |
| A\_51\_P357877 | NM\_175530 | hypothetical F-box domain containing protein | 2.10 | 0,0012 |
| A\_51\_P321531 | AF479672 | "Mus musculus Pur-gamma B-form (Purg) mRNA, complete cds� alternatively spliced" | 2.10 | 0,0006 |
| A\_51\_P300602 | AV124335 | ATP synthase, H+ transporting, mitochondrial F0 complex, subunit c (subunit 9), isoform 1 | 2.10 | 0,0003 |
| A\_51\_P468388 | NM\_011235 | Mus musculus RAD51-like 3 (S. cerevisiae) (Rad51l3), mRNA | 2.10 | 0,0007 |
| A\_51\_P190000 | NM\_027134 | Mus musculus, RIKEN cDNA 2310020P08 gene, clone MGC:28710 IMAGE:4456981, mRNA, complete cds | 2.10 | 0,0040 |
| A\_51\_P328300 | NM\_009787 | Mus musculus calcium binding protein, intestinal (Cai), mRNA | 2.08 | 0,0015 |
| A\_51\_P451986 | AK043702 | hypothetical protein | 2.08 | 0,0136 |
| A\_51\_P215038 | NM\_182991 | similar to BRAIN SPECIFIC MEMBRANE-ANCHORED PROTEIN PRECURSOR [Homo sapiens] | 2.08 | 0,0079 |
| A\_51\_P170463 | NM\_001025381 | Mus musculus cDNA, 3 end | 2.08 | 0,0132 |
| A\_51\_P132592 | NM\_029998 | protein phosphatase 1, regulatory (inhibitor) subunit 7 | 2.08 | 0,0045 |
| A\_51\_P322041 | NM\_177678 | hypothetical LIM domain, Villin headpiece domain containing protein | 2.08 | 0,0317 |
| A\_51\_P116039 | NM\_008256 | 3-hydroxy-3-methylglutaryl-Coenzyme A synthase 2 | 2.08 | 0,0419 |
| A\_51\_P109335 | NM\_009938 | Mus musculus, Similar to alpha-coatomer protein, clone IMAGE:3498647, mRNA, partial cds | 2.08 | 0,0014 |
| A\_51\_P468398 | NM\_175300 | ANAPHASE-PROMOTING COMPLEX SUBUNIT 2 homolog [Homo sapiens] | 2.08 | 0,0003 |
| A\_51\_P304126 | NM\_026267 | hypothetical protein | 2.08 | 0,0109 |
| A\_51\_P423431 | NM\_027838 | SENTRIN/SUMO-SPECIFIC PROTEASE homolog [Homo sapiens] | 2.08 | 0,0002 |
| A\_51\_P445912 | XM\_488951 | unknown EST | 2.08 | 0,0173 |
| A\_51\_P265676 | NM\_030113 | Mus musculus Rho GTPase activating protein 10 (Arhgap10), mRNA | 2.08 | 0,0453 |
| A\_51\_P162786 | NM\_024475 | Mus musculus hypothetical protein, MGC: 7513 (BC002236), mRNA | 2.08 | 0,0002 |
| A\_51\_P370700 | NM\_010324 | Mus musculus glutamate oxaloacetate transaminase 1, soluble (Got1), mRNA | 2.08 | 0,0008 |
| A\_51\_P199425 | NM\_133236 | Mus musculus GIG18 (Gig18) mRNA, complete cds | 2.08 | 0,0021 |
| A\_51\_P344741 | BC086456 | hypothetical Hemopexin domain/Serine/threonine specific protein phosphatase containing protein | 2.08 | 0,0006 |
| A\_51\_P118103 | BC085163 | Mus musculus cDNA, 5 end | 2.08 | 0,0003 |
| A\_51\_P256957 | NM\_146033 | hypothetical Ankyrin repeat profile/Ankyrin-repeat/MYND zinc finger (ZnF) domain/Ankyrin repeat region circular profile/Yeast DNA-binding domain containing protein | 2.08 | 0,0192 |
| A\_51\_P517075 | NM\_011340 | Mus musculus serine (or cysteine) proteinase inhibitor, clade F (alpha-2 antiplasmin, pigment epithelium derived factor), member 1 (Serpinf1), mRNA | 2.08 | 0,0386 |
| A\_51\_P348119 | NM\_027315 | unknown EST | 2.08 | 0,0002 |
| A\_51\_P268953 | AK035894 | hypothetical protein | 2.08 | 0,0068 |
| A\_51\_P247098 | AK046067 | hypothetical protein | 2.07 | 0,0003 |
| A\_51\_P460499 | NM\_001001978 | Mus musculus glyceraldehyde-3-phosphate dehydrogenase (Gapd), mRNA | 2.07 | 0,0032 |
| A\_51\_P196523 | NM\_008144 | Mus musculus G protein gamma 3 linked gene (Gng3lg), mRNA | 2.07 | 0,0028 |
| A\_51\_P167374 | NM\_026181 | Mus musculus RIKEN cDNA 1300003A17 gene (1300003A17Rik), mRNA | 2.07 | 0,0012 |
| A\_51\_P109452 | NM\_008519 | Mus musculus leukotriene B4 receptor 1 (Ltb4r1), mRNA | 2.07 | 0,0380 |
| A\_51\_P311089 | NM\_153128 | KELCH-LIKE PROTEIN C3IP1 (CDNA FLJ14750 FIS, CLONE NT2RP3002948, WEAKLY SIMILAR TO RING CANAL PROTEIN) homolog [Homo sapiens] | 2.07 | 0,0019 |
| A\_51\_P417758 | AK085009 | Mus musculus cDNA, 3 end | 2.06 | 0,0385 |
| A\_51\_P169275 | NM\_133804 | Mus musculus expressed sequence R74613 (R74613), mRNA | 2.06 | 0,0495 |
| A\_51\_P245233 | NM\_175134 | hypothetical Ankyrin repeat profile/Ankyrin-repeat/Ankyrin repeat region circular profile/Yeast DNA-binding domain containing protein | 2.06 | 0,0001 |
| A\_51\_P369510 | NM\_025839 | Mus musculus RIKEN cDNA 1110001K21 gene (1110001K21Rik), mRNA | 2.06 | 0,0030 |
| A\_51\_P502975 | NM\_146372 | Mus musculus olfactory receptor MOR267-15 (MOR267-15) pseudogene | 2.06 | 0,0163 |
| A\_51\_P304478 | NM\_173446 | hypothetical protein | 2.06 | 0,0098 |
| A\_51\_P211526 | NM\_026889 | HYPOTHETICAL PROTEIN FLJ22170 homolog [Mus musculus] | 2.06 | 0,0031 |
| A\_51\_P367772 | AK002680 | RIKEN cDNA 0610027A18 gene | 2.06 | 0,0009 |
| A\_51\_P319065 | NM\_033604 | Mus musculus arkadia (Ark-pending), mRNA | 2.06 | 0,0001 |
| A\_51\_P323281 | NM\_152825 | DJ199J3 NOVEL PROTEIN SIMILAR TO UBIQUITIN CARBOXYL TERMINAL HYDROLASE 16 EC 3.1.2.15 | 2.06 | 0,0047 |
| A\_51\_P257542 | NM\_178756 | weakly similar to DJ846F13.1 (PHOSPHATIDIC ACID PHOSPHATASE TYPE 2C) (FRAGMENT) [Homo sapiens] | 2.06 | 0,0162 |
| A\_51\_P499979 | A\_51\_P499979 | Mus musculus cDNA, 5 end | 2.06 | 0,0141 |
| A\_51\_P232868 | NM\_176995 | hypothetical protein | 2.06 | 0,0256 |
| A\_51\_P488819 | AK016591 | hypothetical Arginine-rich region containing protein | 2.04 | 0,0215 |
| A\_51\_P382928 | NM\_145529 | CLEAVAGE STIMULATION FACTOR 77KDA SUBUNIT homolog [Homo sapiens] | 2.04 | 0,0025 |
| A\_51\_P110759 | NM\_009199 | solute carrier family 1, member 1 | 2.04 | 0,0014 |
| A\_51\_P455410 | NM\_028780 | Mus musculus transmembrane 9 superfamily member 1 (Tm9sf1), mRNA | 2.04 | 0,0001 |
| A\_51\_P312885 | NM\_027898 | Mus musculus RIKEN cDNA 1300003M23 gene (1300003M23Rik), mRNA | 2.04 | 0,0061 |
| A\_51\_P499369 | NM\_145599 | SIMILAR TO HYPOTHETICAL PROTEIN FLJ10846 | 2.04 | 0,0015 |
| A\_51\_P363791 | NM\_018829 | Mus musculus adaptor-related protein complex AP-3, mu 1 subunit (Ap3m1), mRNA | 2.04 | 0,0073 |
| A\_51\_P432764 | BC062197 | Mus musculus protein tyrosine phosphatase, non-receptor type substrate 1 (Ptpns1), mRNA | 2.04 | 0,0179 |
| A\_51\_P137947 | NM\_009441 | Mus musculus tetratricopeptide repeat domain (Ttc3), mRNA | 2.04 | 0,0023 |
| A\_51\_P411996 | NM\_023483 | Mus musculus RIKEN cDNA 1110032A03 gene (1110032A03Rik), mRNA | 2.04 | 0,0006 |
| A\_51\_P137322 | NM\_031161 | Mus musculus cholecystokinin (Cck), mRNA | 2.04 | 0,0279 |
| A\_51\_P377506 | NM\_172946 | KERATIN, TYPE I | 2.04 | 0,0049 |
| A\_51\_P480178 | NM\_133191 | Mus musculus expressed sequence AI042819 (AI042819), mRNA | 2.04 | 0,0228 |
| A\_51\_P150132 | NM\_025299 | Mus musculus dim1 (S. pombe) (Dim1-pending), mRNA | 2.04 | 0,0001 |
| A\_51\_P129724 | NM\_175244 | hypothetical HECT domain (Ubiquitin-protein ligase) containing protein | 2.03 | 0,0008 |
| A\_51\_P522004 | NM\_134448 | Mus musculus dystonin (Dst), mRNA | 2.03 | 0,0396 |
| A\_51\_P217946 | AK083761 | unknown EST | 2.03 | 0,0026 |
| A\_51\_P454002 | NM\_027129 | RIKEN cDNA 0610038L13 gene | 2.03 | 0,0026 |
| A\_51\_P139069 | NM\_172372 | SIMILAR TO JM5 PROTEIN homolog [Mus musculus] | 2.03 | 0,0035 |
| A\_51\_P223085 | AK014830 | unclassifiable | 2.03 | 0,0127 |
| A\_51\_P427624 | NM\_025973 | Mus musculus RIKEN cDNA 2210410L06 gene (2210410L06Rik), mRNA | 2.03 | 0,0245 |
| A\_51\_P148592 | NM\_026045 | Mus musculus, Similar to pre-mRNA processing factor 18, clone IMAGE:3496857, mRNA | 2.03 | 0,0016 |
| A\_51\_P502107 | AK034345 | unknown EST | 2.03 | 0,0003 |
| A\_51\_P214360 | NM\_133251 | Mus musculus vestigial-related factor (LOC170828), mRNA | 2.03 | 0,0117 |
| A\_51\_P274073 | NM\_175545 | hypothetical protein | 2.03 | 0,0050 |
| A\_51\_P184236 | NM\_026173 | Mus musculus RIKEN cDNA 1200014M14 gene (1200014M14Rik), mRNA | 2.03 | 0,0122 |
| A\_51\_P386964 | XM\_485063 | hypothetical protein | 2.03 | 0,0409 |
| A\_51\_P306308 | NM\_028220 | hypothetical G-protein beta WD-40 repeats containing protein | 2.01 | 0,0106 |
| A\_51\_P401304 | NM\_029665 | hypothetical protein | 2.01 | 0,0009 |
| A\_51\_P258522 | BC032214 | CDNA FLJ12883 FIS, CLONE NT2RP2003981, WEAKLY SIMILAR TO VACUOLAR PROTEIN SORTING-ASSOCIATED PROTEIN VPS8 homolog [Homo sapiens] | 2.01 | 0,0052 |
| A\_51\_P390389 | AK086718 | unknown EST | 2.01 | 0,0014 |
| A\_51\_P407025 | NM\_007607 | Mus musculus carbonic anhydrase 4 (Car4), mRNA | 2.01 | 0,0072 |
| A\_51\_P486683 | NM\_021492 | Mus musculus mRNA, partial cds, clone:2-73 | 2.01 | 0,0006 |
| A\_51\_P164892 | TC1434225 | Mus musculus cDNA, 5 end | 2.01 | 0,0132 |
| A\_51\_P243503 | AF037454 | Mus musculus itchy (Itch), mRNA | 2.01 | 0,0014 |
| A\_51\_P501473 | AK083741 | unknown EST | 2.01 | 0,0016 |
| A\_51\_P452779 | NM\_133198 | Mus musculus liver glycogen phosphorylase (Pygl), mRNA | 2.01 | 0,0018 |
| A\_51\_P133229 | NM\_028072 | Mus musculus, RIKEN cDNA 2010004N24 gene, clone MGC:27878 IMAGE:3495588, mRNA, complete cds | 2.01 | 0,0204 |
| A\_51\_P490148 | NM\_153424 | hypothetical protein | 2.01 | 0,0025 |
| A\_51\_P471798 | AK045812 | matrin cyclophilin {Rattus norvegicus} | 2.01 | 0,0072 |
| A\_51\_P311137 | NM\_011360 | Mus musculus sarcoglycan, epsilon (Sgce), mRNA | 2.01 | 0,0004 |
| A\_51\_P488590 | NM\_008019 | Mus musculus FK506 binding protein 1a (12 kDa) (Fkbp1a), mRNA | 2.01 | 0,0370 |
| A\_51\_P514584 | NM\_023064 | Mus musculus RIKEN cDNA 1700021K02 gene (1700021K02Rik), mRNA | 2.01 | 0,0440 |
| A\_51\_P142091 | NM\_030121 | Mus musculus, ankyrin repeat and SOCS box-containing protein 8, clone MGC:36839 IMAGE:4194829, mRNA, complete cds | 2.00 | 0,0082 |
| A\_51\_P182174 | NM\_019831 | Mus musculus zinc finger protein 261 (Zfp261), mRNA | 2.00 | 0,0041 |
| A\_51\_P487360 | NM\_016677 | Mus musculus hippocalcin-like 1 (Hpcal1), mRNA | 2.00 | 0,0011 |
| A\_51\_P501793 | NM\_008237 | Mus musculus hairy and enhancer of split 3, (Drosophila) (Hes3), mRNA | 2.00 | 0,0282 |
| A\_51\_P261443 | NM\_011550 | Mus musculus transcription factor-like 4 (Tcfl4), mRNA | 2.00 | 0,0001 |
| A\_51\_P336384 | AK032843 | SALIVARY PROLINE RICH PROTEIN [CONTAINS: PEPTIDE P | 2.00 | 0,0037 |
| A\_51\_P172692 | NM\_017478 | Mus musculus coatomer protein complex, subunit gamma 2 (Copg2), mRNA | 2.00 | 0,0110 |
| A\_51\_P507899 | NM\_029553 | Mus musculus, Similar to RIKEN cDNA 0610012F22 gene, clone MGC:27659 IMAGE:4527657, mRNA, complete cds | 2.00 | 0,0013 |
| A\_51\_P312217 | BC089370 | unknown EST | 2.00 | 0,0255 |
| A\_51\_P248304 | NM\_178027 | weakly similar to VPS26 PROTEIN HOMOLOG (H<BETA58 PROTEIN) (H BETA 58) [Mus musculus] | 2.00 | 0,0004 |
| A\_51\_P169560 | XM\_126361 | PROTEIN NJMU-R1 homolog [Mus musculus] | 2.00 | 0,0006 |
| A\_51\_P311319 | NM\_029022 | Mus musculus, clone IMAGE:4222865, mRNA, partial cds | 2.00 | 0,0094 |
| A\_51\_P170405 | NM\_008356 | Mus musculus interleukin 13 receptor, alpha 2 (Il13ra2), mRNA | 2.00 | 0,0159 |
| A\_51\_P181751 | NM\_207267 | THYMOSIN BETA-LIKE PROTEIN homolog [Rattus norvegicus] | 1.99 | 0,0020 |
| A\_51\_P450123 | NM\_053163 | Mus musculus mitochondrial ribosomal protein L36 (Mrpl36), mRNA | 1.99 | 0,0090 |
| A\_51\_P115988 | NM\_024480 | Mus musculus hypothetical protein, MGC:7813 (BC003251), mRNA | 1.99 | 0,0003 |
| A\_51\_P171286 | AK037232 | unknown EST | 1.99 | 0,0373 |
| A\_51\_P154679 | NM\_173186 | hypothetical RabGAP/TBC domain containing protein | 1.99 | 0,0005 |
| A\_51\_P223033 | NM\_027968 | F-BOX DOMAIN PROTEIN homolog [Homo sapiens] | 1.99 | 0,0287 |
| A\_51\_P317211 | NM\_146256 | hypothetical Glyoxalase/Bleomycin resistance protein/Dihydroxybiphenyl dioxygenase structure containing protein | 1.99 | 0,0019 |
| A\_51\_P460067 | NM\_178709 | hypothetical RING finger containing protein | 1.99 | 0,0064 |
| A\_51\_P436717 | NM\_011431 | Mus musculus U5 small nuclear ribonucleoprotein 116 kDa (Snrp116-pending), mRNA | 1.99 | 0,0041 |
| A\_51\_P402818 | NM\_008506 | Mus musculus cDNA, 5 end | 1.99 | 0,0019 |
| A\_51\_P512744 | NM\_025388 | Mus musculus RIKEN cDNA 1110021H02 gene (1110021H02Rik), mRNA | 1.99 | 0,0002 |
| A\_51\_P118885 | NM\_008537 | Mus musculus alpha-methylacyl-CoA racemase (Amacr), mRNA | 1.99 | 0,0020 |
| A\_51\_P282179 | NM\_020009 | Mus musculus FK506 binding protein 12-rapamycin associated protein 1 (Frap1), mRNA | 1.99 | 0,0006 |
| A\_51\_P202801 | NM\_019875 | Mus musculus ATP-binding cassette, sub-family B (MDR/TAP), member 9 (Abcb9), mRNA | 1.99 | 0,0010 |
| A\_51\_P444633 | NM\_029653 | Mus musculus, Similar to death-associated protein kinase 1, clone IMAGE:5345390, mRNA, partial cds | 1.99 | 0,0346 |
| A\_51\_P317788 | NM\_176843 | unknown EST | 1.99 | 0,0003 |
| A\_51\_P147379 | NM\_133803 | Mus musculus expressed sequence C86324 (C86324), mRNA | 1.97 | 0,0037 |
| A\_51\_P464558 | NM\_199055 | similar to N-ACETYLGALACTOSAMINE 4-O-SULFOTRANSFERASE 2 GALNAC4ST-2 [Homo sapiens] | 1.97 | 0,0158 |
| A\_51\_P428742 | NM\_172437 | similar to HYPOTHETICAL 80.7 KDA PROTEIN [Homo sapiens] | 1.97 | 0,0037 |
| A\_51\_P343598 | AK012407 | unknown EST | 1.97 | 0,0010 |
| A\_51\_P122232 | NM\_025657 | hypothetical Leucine-rich repeat, typical subtype containing protein | 1.97 | 0,0027 |
| A\_51\_P417025 | NM\_013837 | Mus musculus protein-tyrosine sulfotransferase 1 (Tpst1), mRNA | 1.97 | 0,0028 |
| A\_51\_P358940 | NM\_016852 | Mus musculus WW domain binding protein 2 (Wbp2), mRNA | 1.97 | 0,0028 |
| A\_51\_P100099 | AK011292 | unknown EST | 1.97 | 0,0061 |
| A\_51\_P378858 | AK017355 | Mus musculus RIKEN cDNA 5430428K15 gene (5430428K15Rik), mRNA | 1.97 | 0,0006 |
| A\_51\_P172523 | AK029960 | similar to CTCL TUMOR ANTIGEN SE2-2 (FRAGMENT) [Homo sapiens] | 1.97 | 0,0035 |
| A\_51\_P211483 | NM\_024472 | Mus musculus hypothetical protein, MGC:7473 (BC002216), mRNA | 1.97 | 0,0001 |
| A\_51\_P389714 | BC030917 | Mus musculus, clone IMAGE:3596085, mRNA | 1.97 | 0,0046 |
| A\_51\_P108173 | NAP099398-001 | Mus musculus mitochondrial ribosomal protein L48 pseudogene (LOC269553) on chromosome 4 | 1.97 | 0,0011 |
| A\_51\_P348624 | NM\_013899 | Mus musculus translocase of inner mitochondrial membrane 13 homolog a (yeast) (Timm13a), mRNA | 1.97 | 0,0005 |
| A\_51\_P235193 | NM\_172926 | SORTING NEXIN | 1.97 | 0,0003 |
| A\_51\_P167452 | AK049330 | hypothetical Target SNARE coiled-coil domain containing protein | 1.97 | 0,0110 |
| A\_51\_P297346 | NM\_029153 | SECRETORY CARRIER-ASSOCIATED MEMBRANE PROTEIN 1 | 1.97 | 0,0005 |
| A\_51\_P355242 | AK028520 | unknown EST | 1.97 | 0,0095 |
| A\_51\_P185103 | NM\_024428 | DPY-30-LIKE PROTEIN homolog [Mus musculus] | 1.97 | 0,0004 |
| A\_51\_P381377 | NM\_023279 | Mus musculus tubulin, beta 3 (Tubb3), mRNA | 1.97 | 0,0089 |
| A\_51\_P255441 | NM\_053164 | Mus musculus mitochondrial ribosomal protein L43 (Mrpl43), mRNA | 1.97 | 0,0161 |
| A\_51\_P133245 | AK122294 | Mus musculus, Similar to KIAA0476 gene product, clone IMAGE:5365056, mRNA, partial cds | 1.97 | 0,0001 |
| A\_51\_P320668 | BC053106 | ACTIN-RELATED PROTEIN 3-BETA homolog [Homo sapiens] | 1.97 | 0,0271 |
| A\_51\_P405985 | NM\_133999 | Mus musculus expressed sequence AI326867 (AI326867), mRNA | 1.97 | 0,0003 |
| A\_51\_P459679 | NM\_007978 | Mus musculus factor 8-associated gene A (F8a), mRNA | 1.97 | 0,0186 |
| A\_51\_P293989 | NM\_146003 | similar to SUMO-1-SPECIFIC PROTEASE 1 (EC 3.4.22.-) (SENTRIN-SPECIFIC PROTEASE SENP6) (PROTEASE FKSG6) [Homo sapiens] | 1.97 | 0,0004 |
| A\_51\_P219721 | NM\_025942 | hypothetical P-loop containing nucleotide triphosphate hydrolases structure containing protein | 1.96 | 0,0057 |
| A\_51\_P186601 | M29546 | Mouse MOD-1 null malic enzyme mRNA, partial cds | 1.96 | 0,0210 |
| A\_51\_P482029 | NM\_053177 | Mus musculus mucolipin 1 (Mcoln1), mRNA | 1.96 | 0,0020 |
| A\_51\_P409408 | NM\_010921 | Mus musculus NK-3 transcription factor, locus 1 (Drosophila) (Nkx3-1), mRNA | 1.96 | 0,0126 |
| A\_51\_P266723 | NM\_172677 | weakly similar to HIGH-GLUCOSE-REGULATED PROTEIN 8 [Homo sapiens] | 1.96 | 0,0210 |
| A\_51\_P175988 | NM\_013561 | Mus musculus 5-hydroxytryptamine (serotonin) receptor 3A (Htr3a), mRNA | 1.96 | 0,0442 |
| A\_51\_P492437 | NM\_024286 | Mus musculus chromosome 10 popeye protein 3 (Pop3) mRNA, complete cds | 1.96 | 0,0034 |
| A\_51\_P114459 | NM\_027537 | hypothetical protein | 1.96 | 0,0022 |
| A\_51\_P116946 | NM\_080467 | Mus musculus ATPase, H+ transporting, lysosomal (vacuolar proton pump) noncatalytic accessory protein 1B (Atp6n1b), mRNA | 1.96 | 0,0004 |
| A\_51\_P272584 | NM\_027727 | hypothetical ARM repeat structure containing protein | 1.96 | 0,0007 |
| A\_51\_P379953 | BC038619 | unknown EST | 1.96 | 0,0156 |
| A\_51\_P142175 | NM\_021295 | Mus musculus mRNA for LanC-like protein 1 (Lancl1 gene) | 1.96 | 0,0188 |
| A\_51\_P405682 | AK034740 | hypothetical Trp-Asp repeat (WD-repeat) structure containing protein | 1.96 | 0,0006 |
| A\_51\_P194910 | TC1430357 | Mus musculus N-myristoyltransferase 1 (Nmt1), mRNA | 1.96 | 0,0008 |
| A\_51\_P492476 | NM\_031249 | Mus musculus cleavage stimulation factor, 3 pre-RNA subunit 2, 64 kDa, tau (Cstf2t-pending), mRNA | 1.96 | 0,0008 |
| A\_51\_P509747 | NM\_023638 | Mus musculus Mporc-d mRNA for porcupine-D, complete cds | 1.96 | 0,0025 |
| A\_51\_P356562 | NM\_133798 | Mus musculus expressed sequence C85658 (C85658), mRNA | 1.96 | 0,0224 |
| A\_51\_P428754 | NM\_008168 | Mus musculus glutamate receptor, ionotropic, kainate 5 (gamma 2) (Grik5), mRNA | 1.96 | 0,0361 |
| A\_51\_P487547 | NM\_025911 | Mus musculus RIKEN cDNA 1810060J02 gene (1810060J02Rik), mRNA | 1.96 | 0,0023 |
| A\_51\_P266546 | NM\_173181 | CGI-62 PROTEIN homolog [Homo sapiens] | 1.96 | 0,0103 |
| A\_51\_P367081 | NM\_011074 | Mus musculus PFTAIRE protein kinase 1 (Pftk1), mRNA | 1.96 | 0,0159 |
| A\_51\_P464021 | NM\_021528 | Mus musculus chondroitin 4-sulfotransferase 2 (C4st2-pending), mRNA | 1.96 | 0,0110 |
| A\_51\_P128096 | NM\_016878 | Mus musculus aspartyl aminopeptidase (Dnpep), mRNA | 1.96 | 0,0024 |
| A\_51\_P152203 | NM\_019565 | Mus musculus Kruppel associated box (KRAB) zinc finger 1 (Kzf1-pending), mRNA | 1.96 | 0,0028 |
| A\_51\_P194740 | NM\_030735 | Mus musculus pheromone receptor V3R9 (V3R9), mRNA | 1.95 | 0,0172 |
| A\_51\_P317542 | AK018192 | unknown EST | 1.95 | 0,0275 |
| A\_51\_P367295 | BU554808 | Mus musculus cDNA, 5 end | 1.95 | 0,0145 |
| A\_51\_P308469 | NM\_011884 | Mus musculus RNA guanylyltransferase and 5-phosphatase (Rngtt), mRNA | 1.95 | 0,0008 |
| A\_51\_P155152 | NM\_020332 | Mus musculus progressive ankylosis (ank), mRNA | 1.95 | 0,0106 |
| A\_51\_P134741 | NM\_008180 | Mus musculus glutathione synthetase (Gss), mRNA | 1.95 | 0,0266 |
| A\_51\_P211108 | XM\_283937 | hypothetical protein | 1.95 | 0,0026 |
| A\_51\_P154513 | NM\_145837 | musculus interleukin 17D (IL-17D), mRNA | 1.95 | 0,0063 |
| A\_51\_P385043 | NM\_178384 | inferred: zinc finger protein ozf {Bos taurus} | 1.95 | 0,0086 |
| A\_51\_P230904 | NM\_019877 | Mus musculus coatomer protein complex, subunit zeta 2 (Copz2), mRNA | 1.95 | 0,0365 |
| A\_51\_P307747 | BC052056 | Mus musculus T2-cadherin mRNA, partial cds | 1.95 | 0,0102 |
| A\_51\_P147056 | BF138649 | ubiquitin-like 4 | 1.95 | 0,0060 |
| A\_51\_P481238 | NM\_027293 | Mus musculus RIKEN cDNA 2610510B01 gene (2610510B01Rik), mRNA | 1.95 | 0,0040 |
| A\_51\_P308844 | NM\_153529 | neuritin | 1.95 | 0,0165 |
| A\_51\_P210470 | NM\_026310 | Mus musculus RIKEN cDNA 1010001C05 gene (1010001C05Rik), mRNA | 1.95 | 0,0012 |
| A\_51\_P431885 | NM\_198412 | DnaJ (Hsp40) homolog, subfamily B, member 6 | 1.95 | 0,0154 |
| A\_51\_P327559 | NM\_009752 | Mus musculus galactosidase, beta 1 (Glb1), mRNA | 1.95 | 0,0060 |
| A\_51\_P512630 | BC035276 | 7ACOMP PROTEIN homolog [Rattus sp] | 1.95 | 0,0074 |
| A\_51\_P125338 | NM\_172263 | similar to HIGH-AFFINITY CAMP-SPECIFIC AND IBMX-INSENSITIVE 3,5-CYCLIC PHOSPHODIESTERASE 8B (EC 3.1.4.17) (FRAGMENT) [Homo sapiens] | 1.95 | 0,0004 |
| A\_51\_P426544 | AB041544 | Mus musculus hypothetical protein, MNCb-2622 (AB041544), mRNA | 1.95 | 0,0225 |
| A\_51\_P189962 | NM\_026380 | unclassifiable | 1.95 | 0,0053 |
| A\_51\_P166695 | NM\_008385 | Mus musculus inositol polyphosphate-5-phosphatase, 75 kDa (Inpp5b), mRNA | 1.93 | 0,0197 |
| A\_51\_P274947 | NM\_026487 | Mus musculus RIKEN cDNA 4921525H23 gene (4921525H23Rik), mRNA | 1.93 | 0,0013 |
| A\_51\_P482043 | NM\_175266 | hypothetical protein | 1.93 | 0,0117 |
| A\_51\_P322542 | AK017436 | unknown EST | 1.93 | 0,0057 |
| A\_51\_P237383 | NM\_021472 | Mus musculus ribonuclease, RNase A family 4 (Rnase4), mRNA | 1.93 | 0,0027 |
| A\_51\_P196889 | XM\_620751 | IRP2, IRON-RESPONSIVE ELEMENT-BINDING PROTEIN/IRON REGULATORY PROTEIN 2 (FRAGMENT) homolog [Homo sapiens] | 1.93 | 0,0028 |
| A\_51\_P174415 | NM\_170755 | hypothetical protein | 1.93 | 0,0025 |
| A\_51\_P176783 | AK014824 | unknown EST | 1.93 | 0,0002 |
| A\_51\_P273667 | NM\_011441 | Mus musculus SRY-box containing gene 17 (Sox17), mRNA | 1.93 | 0,0182 |
| A\_51\_P293665 | AK003646 | weakly similar to LDOC1 PROTEIN (LEUCINE ZIPPER PROTEIN DOWN-REGULATED IN CANCER CELLS) [Homo sapiens] | 1.93 | 0,0009 |
| A\_51\_P318580 | NM\_028021 | weakly similar to MYOSIN HEAVY CHAIN, EMBRYONIC SMOOTH MUSCLE ISOFORM (FRAGMENT) [Oryctolagus cuniculus] | 1.93 | 0,0068 |
| A\_51\_P139364 | AK087995 | similar to B4-2 PROTEIN [Homo sapiens] | 1.93 | 0,0057 |
| A\_51\_P143470 | NM\_011592 | Mus musculus translocator of inner mitochondrial membrane 44 (Timm44), mRNA | 1.93 | 0,0003 |
| A\_51\_P370960 | NM\_027815 | ESOPHAGEAL CANCER ASSOCIATED PROTEIN homolog [Homo sapiens] | 1.93 | 0,0070 |
| A\_51\_P324172 | NM\_011245 | Mus musculus RAS protein-specific guanine nucleotide-releasing factor 1 (Rasgrf1), mRNA | 1.92 | 0,0033 |
| A\_51\_P497039 | NM\_007863 | Mus musculus membrane protein, palmitoylated 3 (MAGUK p55 subfamily member 3) (Mpp3), mRNA | 1.92 | 0,0178 |
| A\_51\_P302222 | NM\_026174 | Mus musculus lysosomal apyrase-like 1 (Lysal1), mRNA | 1.92 | 0,0024 |
| A\_51\_P489629 | AF155547 | Mus musculus erythrocyte protein band 4.9 (Epb4.9), mRNA | 1.92 | 0,0057 |
| A\_51\_P430388 | NM\_025362 | Mus musculus RIKEN cDNA 1300007M11 gene (1300007M11Rik), mRNA | 1.92 | 0,0006 |
| A\_51\_P216527 | NM\_145355 | hypothetical RING finger containing protein | 1.92 | 0,0271 |
| A\_51\_P303238 | BC006774 | Mus musculus, Similar to transmembrane trafficking protein, clone MGC:8106 IMAGE:3588721, mRNA, complete cds | 1.92 | 0,0003 |
| A\_51\_P165098 | NM\_028758 | similar to ADP-RIBOSYLATION FACTOR BINDING PROTEIN GGA2 (GOLGI-LOCALIZED, GAMMA EAR-CONTAINING, ARF-BINDING PROTEIN 2) (GAMMA-ADAPTIN RELATED PROTEIN 2) (VEAR) (VHS DOMAIN AND EAR DOMAIN OF GAMMA-ADAPTIN) [Homo sapiens] | 1.92 | 0,0006 |
| A\_51\_P375431 | AK083100 | unknown EST | 1.92 | 0,0027 |
| A\_51\_P501757 | BC096024 | hypothetical protein | 1.92 | 0,0063 |
| A\_51\_P192491 | NM\_021793 | Mus musculus transmembrane protein 8 (five membrane-spanning domains) (Tmem8), mRNA | 1.92 | 0,0032 |
| A\_51\_P422165 | NM\_023876 | Mus musculus elongation protein 4 homolog (S. cerevisiae) (Elp4), mRNA | 1.92 | 0,0225 |
| A\_51\_P313120 | AK038526 | CYTOCHROME P450 | 1.92 | 0,0008 |
| A\_51\_P132081 | NM\_001013769 | weakly similar to ZINC FINGER PROTEIN 43 (HTF6) [Homo sapiens] | 1.92 | 0,0288 |
| A\_51\_P390334 | 8430408O14 | unknown EST | 1.92 | 0,0016 |
| A\_51\_P211854 | NM\_011347 | Mus musculus selectin, platelet (Selp), mRNA | 1.92 | 0,0358 |
| A\_51\_P112662 | NM\_001018042 | Mus musculus transcription factor Sp3 mRNA, partial cds | 1.92 | 0,0007 |
| A\_51\_P449133 | NM\_013464 | Mus musculus aryl-hydrocarbon receptor (Ahr), mRNA | 1.92 | 0,0058 |
| A\_51\_P189438 | NM\_172272 | "Mus musculus, similar to glycyl-tRNA synthetase� GlyRS� glycine tRNA ligase, clone IMAGE:5360188, mRNA, partial cds" | 1.92 | 0,0002 |
| A\_51\_P107934 | NM\_172858 | SERINE/THREONINE-PROTEIN KINASE PAK 5 (EC 2.7.1.-) (P21-ACTIVATED KINASE 5) (PAK-5) homolog [Homo sapiens] | 1.92 | 0,0098 |
| A\_51\_P297961 | NM\_023042 | Mus musculus RecQ protein-like (Recql), mRNA | 1.92 | 0,0028 |
| A\_51\_P184849 | NM\_008825 | 6-phosphofructo-2-kinase fructose -2,6-biphosphatase 2, full insert sequence. | 1.92 | 0,0291 |
| A\_51\_P469950 | NM\_153070 | Mus musculus brain stress early protein (Gbi) mRNA, complete cds | 1.91 | 0,0007 |
| A\_51\_P272363 | NM\_134160 | Mus musculus mucolipin-3 (Mcoln3), mRNA | 1.91 | 0,0110 |
| A\_51\_P377789 | NM\_009582 | Mus musculus mitogen activated protein kinase kinase kinase 12 (Map3k12), mRNA | 1.91 | 0,0039 |
| A\_51\_P102225 | NM\_011543 | Mus musculus transcription elongation factor B (SIII), polypeptide 1 (15 kDa),-like (Tceb1l), mRNA | 1.91 | 0,0070 |
| A\_51\_P296512 | NM\_018818 | Mus musculus choroidermia (Chm), mRNA | 1.91 | 0,0009 |
| A\_51\_P202074 | NM\_146171 | Mus musculus, clone IMAGE:3499621, mRNA, partial cds | 1.91 | 0,0006 |
| A\_51\_P346747 | NM\_013919 | Mus musculus ubiquitin specific protease 21 (Usp21), mRNA | 1.91 | 0,0042 |
| A\_51\_P482503 | NM\_009413 | Mus musculus tumor protein D52-like 1 (Tpd52l1), mRNA | 1.91 | 0,0111 |
| A\_51\_P360918 | NM\_020578 | Mus musculus EH-domain containing 3 (Ehd3), mRNA | 1.91 | 0,0008 |
| A\_51\_P432877 | AK008158 | Mus musculus, RIKEN cDNA 2010008E23 gene, clone IMAGE:4482737, mRNA, partial cds | 1.91 | 0,0004 |
| A\_51\_P337974 | NM\_133363 | Mus musculus calsarcin-3 (LOC170947), mRNA | 1.91 | 0,0036 |
| A\_51\_P285763 | NM\_028375 | Mus musculus RIKEN cDNA 2900027G03 gene (2900027G03Rik), mRNA | 1.91 | 0,0045 |
| A\_51\_P181170 | NM\_021376 | Mus musculus peroxisomal integral membrane protein (Pmp47), mRNA | 1.91 | 0,0378 |
| A\_51\_P352264 | NM\_153056 | Mus musculus, Similar to sirtuin silent mating type information regulation 2 homolog 7 (S. cerevisiae), clone MGC:37560 IMAGE:4987746, mRNA, complete cds | 1.91 | 0,0055 |
| A\_51\_P381387 | NM\_153415 | POMT2 homolog [Homo sapiens] | 1.91 | 0,0003 |
| A\_51\_P259726 | NM\_054046 | Mus musculus differentially expressed in FDCP 8 (Def8), mRNA | 1.91 | 0,0034 |
| A\_51\_P232980 | XM\_355244 | BG120K12 3 NOVEL PROTEIN SIMILAR TO ARCHAEAL, YEAST AND WORM N2,N2 DIMETHYLGUANOSINE TRNA METHYLTRANSFERASE ISOFORM | 1.91 | 0,0001 |
| A\_51\_P221762 | NM\_007936 | Eph receptor A4 | 1.89 | 0,0038 |
| A\_51\_P299934 | NM\_173022 | Mus musculus, clone IMAGE:1246046, mRNA, partial cds | 1.89 | 0,0008 |
| A\_51\_P385370 | AK004304 | LIM ONLY PROTEIN 6 TRIPLE LIM DOMAIN PROTEIN | 1.89 | 0,0037 |
| A\_51\_P237865 | NM\_021283 | Mus musculus interleukin 4 (Il4), mRNA | 1.89 | 0,0348 |
| A\_51\_P329278 | AK078133 | unknown EST | 1.89 | 0,0006 |
| A\_51\_P106859 | NM\_138721 | Mus musculus U7 snRNP-specific Sm-like (Lsm10), mRNA | 1.89 | 0,0441 |
| A\_51\_P186483 | AK049181 | hypothetical Phosphoinositide 3-kinase family, ras-binding domain containing protein | 1.89 | 0,0073 |
| A\_51\_P465141 | NM\_026142 | Mus musculus RIKEN cDNA 3632451O06 gene (3632451O06Rik), mRNA | 1.89 | 0,0204 |
| A\_51\_P368743 | AK043880 | unknown EST | 1.89 | 0,0021 |
| A\_51\_P162624 | NM\_133678 | Mus musculus RIKEN cDNA 2410004C24 gene (2410004C24Rik), mRNA | 1.89 | 0,0059 |
| A\_51\_P334730 | NM\_020611 | Mus musculus steroid 5 alpha-reductase 2-like (Srd5a2l), mRNA | 1.89 | 0,0021 |
| A\_51\_P423361 | TC1433143 | Mus musculus cDNA, 5 end | 1.89 | 0,0047 |
| A\_51\_P255832 | NM\_019409 | Mus musculus oligodendrocyte myelin glycoprotein (Omg), mRNA | 1.89 | 0,0216 |
| A\_51\_P140797 | AK004618 | similar to PC326 PROTEIN [Homo sapiens] | 1.89 | 0,0050 |
| A\_51\_P513586 | NM\_025617 | Mus musculus RIKEN cDNA 2210012G02 gene (2210012G02Rik), mRNA | 1.89 | 0,0195 |
| A\_51\_P266763 | NM\_007998 | Mus musculus ferrochelatase (Fech), mRNA | 1.89 | 0,0087 |
| A\_51\_P333712 | NM\_013875 | Mus musculus phosphodiesterase 7B (Pde7b), mRNA | 1.89 | 0,0133 |
| A\_51\_P110699 | NM\_170756 | SPERMATOGENESIS-ASSOCIATED PROTEIN 2 homolog [Rattus norvegicus] | 1.89 | 0,0095 |
| A\_51\_P308447 | AK087556 | ubiquitin-like 1 (sentrin) activating enzyme E1A | 1.89 | 0,0015 |
| A\_51\_P327232 | NM\_177041 | Mus musculus lens epithelial protein (Lenep), mRNA | 1.89 | 0,0022 |
| A\_51\_P280200 | NM\_009462 | Mus musculus ubiquintin c-terminal hydrolase related polypeptide (Uchrp), mRNA | 1.89 | 0,0050 |
| A\_51\_P443394 | NM\_134150 | Mus musculus expressed sequence AI850305 (AI850305), mRNA | 1.89 | 0,0017 |
| A\_51\_P468876 | NM\_178118 | hypothetical DIX domain containing protein | 1.89 | 0,0015 |
| A\_51\_P417321 | AK041130 | weakly similar to ZINC FINGER PROTEIN (FRAGMENT) [Homo sapiens] | 1.89 | 0,0004 |
| A\_51\_P227785 | AK033046 | unclassifiable | 1.88 | 0,0355 |
| A\_51\_P341255 | AK078885 | unknown EST | 1.88 | 0,0118 |
| A\_51\_P138185 | NM\_134083 | Mus musculus expressed sequence AW240694 (AW240694), mRNA | 1.88 | 0,0072 |
| A\_51\_P503933 | NM\_009790 | Mus musculus, clone IMAGE:2631567, mRNA | 1.88 | 0,0048 |
| A\_51\_P204080 | NM\_013820 | Mus musculus hexokinase 2 (Hk2), mRNA | 1.88 | 0,0005 |
| A\_51\_P347965 | NM\_007427 | Mus musculus agouti related protein (Agrp), mRNA | 1.88 | 0,0161 |
| A\_51\_P492366 | NM\_007557 | Mus musculus bone morphogenetic protein 7 (Bmp7), mRNA | 1.88 | 0,0020 |
| A\_51\_P178735 | NM\_146108 | Mus musculus, Similar to 3-hydroxyisobutyryl-Coenzyme A hydrolase, clone MGC:31364 IMAGE:4238681, mRNA, complete cds | 1.88 | 0,0489 |
| A\_51\_P305352 | AB093272 | SYNAPTOJANIN 1 (EC 3.1.3.56) (SYNAPTIC INOSITOL-1,4,5-TRISPHOSPHATE 5- PHOSPHATASE 1) homolog [Rattus norvegicus] | 1.88 | 0,0226 |
| A\_51\_P458428 | NM\_138606 | Mus musculus proviral integration site 2 (Pim2), mRNA | 1.88 | 0,0146 |
| A\_51\_P438293 | BC038250 | Mus musculus cDNA, 5 end | 1.88 | 0,0059 |
| A\_51\_P483168 | AW536275 | Mus musculus cDNA, 3 end | 1.88 | 0,0135 |
| A\_51\_P363210 | XM\_485743 | hypothetical protein | 1.88 | 0,0174 |
| A\_51\_P412338 | NM\_172120 | VACUOLAR ASSEMBLY PROTEIN VPS41 HOMOLOG (S53) homolog [Homo sapiens] | 1.88 | 0,0006 |
| A\_51\_P219444 | NM\_013880 | Mus musculus PLC-L2 mRNA for phospholipase C-L2, complete cds | 1.88 | 0,0041 |
| A\_51\_P277275 | NM\_009065 | Mus musculus RAS-like protein expressed in neuron (Rin), mRNA | 1.88 | 0,0003 |
| A\_51\_P468955 | NM\_133825 | Mus musculus expressed sequence AI987691 (AI987691), mRNA | 1.88 | 0,0071 |
| A\_51\_P297896 | BC048187 | unknown EST | 1.88 | 0,0151 |
| A\_51\_P465871 | NM\_021448 | Mus musculus signal transducer and activator of transcription interacting protein 1 (Statip1), mRNA | 1.88 | 0,0011 |
| A\_51\_P245336 | NM\_009702 | AQUARIUS (FRAGMENT) | 1.88 | 0,0026 |
| A\_51\_P388984 | NM\_026446 | Mus musculus regulator of G-protein signaling 19 (Rgs19), mRNA | 1.88 | 0,0002 |
| A\_51\_P162196 | AK013739 | hypothetical protein | 1.88 | 0,0151 |
| A\_51\_P424561 | NM\_009567 | Mus musculus zinc finger protein 93 (Zfp93), mRNA | 1.88 | 0,0483 |
| A\_51\_P473259 | NM\_170778 | Mus musculus, Similar to dihydropyrimidine dehydrogenase, clone MGC:37940 IMAGE:5126155, mRNA, complete cds | 1.87 | 0,0066 |
| A\_51\_P499755 | NM\_009672 | Mus musculus acidic nuclear phosphoprotein 32 (Anp32), mRNA | 1.87 | 0,0047 |
| A\_51\_P408932 | NM\_024186 | Mus musculus single-stranded DNA binding protein 2 (Ssbp2), mRNA | 1.87 | 0,0043 |
| A\_51\_P399545 | NM\_175251 | CDNA FLJ14812 FIS, CLONE NT2RP4002081, WEAKLY SIMILAR TO TRANSCRIPTION INITIATION FACTOR IIA ALPHA AND BETA CHAINS (FRAGMENT) homolog [Homo sapiens] | 1.87 | 0,0004 |
| A\_51\_P302450 | NM\_172589 | hypothetical Aminotransferases class-II containing protein | 1.87 | 0,0286 |
| A\_51\_P406179 | NM\_025538 | inferred: putative [Mus musculus] | 1.87 | 0,0130 |
| A\_51\_P363759 | NM\_019434 | Mus musculus minichromosome maintenance deficient (S. cerevisiae) 3-associated protein (Mcm3ap), mRNA | 1.87 | 0,0112 |
| A\_51\_P195258 | NM\_019426 | Mus musculus activating transcription factor 7 interacting protein (Atf7ip), mRNA | 1.87 | 0,0099 |
| A\_51\_P143721 | NM\_134044 | Mus musculus expressed sequence AI413782 (AI413782), mRNA | 1.87 | 0,0084 |
| A\_51\_P405423 | BC094221 | unknown EST | 1.87 | 0,0078 |
| A\_51\_P421680 | BC064456 | weakly similar to DNA-BINDING PROTEIN (FRAGMENT) [Homo sapiens] | 1.87 | 0,0485 |
| A\_51\_P153812 | AK220368 | similar to UBIQUITIN SPECIFIC PROTEASE (FRAGMENT) [Mus musculus] | 1.87 | 0,0040 |
| A\_51\_P376658 | AK004090 | hypothetical protein | 1.87 | 0,0015 |
| A\_51\_P398868 | NM\_008217 | Mus musculus hyaluronan synthase 3 (Has3), mRNA | 1.87 | 0,0003 |
| A\_51\_P506748 | NM\_172739 | hypothetical protein | 1.87 | 0,0044 |
| A\_51\_P354500 | NM\_025828 | Mus musculus RIKEN cDNA 1300009F09 gene (1300009F09Rik), mRNA | 1.87 | 0,0038 |
| A\_51\_P413122 | NM\_028126 | SEROLOGICALLY DEFINED BREAST CANCER ANTIGEN NY-BR-96 homolog [Homo sapiens] | 1.87 | 0,0021 |
| A\_51\_P219055 | NM\_026936 | similar to CYTOCHROME OXIDASE BIOGENESIS PROTEIN OXA1, MITOCHONDRIAL PRECURSOR (OXA1-LIKE PROTEIN) (OXA1HS) [Homo sapiens] | 1.87 | 0,0015 |
| A\_51\_P138348 | NM\_007454 | Mus musculus adaptor protein complex AP-1, beta 1 subunit (Ap1b1), mRNA | 1.87 | 0,0011 |
| A\_51\_P351263 | NM\_001011775 | Mus musculus olfactory receptor MOR266-10 (MOR266-10) pseudogene | 1.85 | 0,0004 |
| A\_51\_P163694 | NM\_172557 | FYVE-FINGER CONTAINING PROTEIN (FRAGMENT) homolog [Mus musculus] | 1.85 | 0,0041 |
| A\_51\_P293077 | NM\_008583 | Mus musculus multiple endocrine neoplasia 1 (Men1), mRNA | 1.85 | 0,0006 |
| A\_51\_P197321 | NM\_016760 | Mus musculus clathrin, light polypeptide (Lca) (Clta), mRNA | 1.85 | 0,0001 |
| A\_51\_P148196 | NM\_009143 | Mus musculus stromal cell derived factor 2 (Sdf2), mRNA | 1.85 | 0,0027 |
| A\_51\_P274124 | NM\_026345 | Mus musculus RIKEN cDNA 9130403P13 gene (9130403P13Rik), mRNA | 1.85 | 0,0055 |
| A\_51\_P516526 | XM\_132038 | similar to SIMILAR TO STROMAL INTERACTION MOLECULE 2 (FRAGMENT) [Homo sapiens] | 1.85 | 0,0392 |
| A\_51\_P264659 | NM\_144518 | hypothetical protein | 1.85 | 0,0004 |
| A\_51\_P508770 | NM\_011846 | Mus musculus matrix metalloproteinase 17 (Mmp17), mRNA | 1.85 | 0,0309 |
| A\_51\_P485220 | NM\_173185 | unknown EST | 1.85 | 0,0036 |
| A\_51\_P300655 | BC082558 | hypothetical protein | 1.85 | 0,0170 |
| A\_51\_P283649 | NM\_172724 | hypothetical ARM repeat structure containing protein | 1.85 | 0,0004 |
| A\_51\_P401876 | NM\_028197 | unknown EST | 1.85 | 0,0108 |
| A\_51\_P465740 | NM\_175215 | hypothetical LysM motif containing protein | 1.85 | 0,0104 |
| A\_51\_P270355 | NM\_019978 | Mus musculus double cortin and calcium/calmodulin-dependent protein kinase-like 1 (Dcamkl1), mRNA | 1.85 | 0,0439 |
| A\_51\_P246816 | NM\_025898 | Mus musculus N-ethylmaleimide sensitive fusion protein attachment protein alpha (Napa), mRNA | 1.85 | 0,0180 |
| A\_51\_P215922 | NM\_009811 | Mus musculus caspase 6 (Casp6), mRNA | 1.85 | 0,0064 |
| A\_51\_P171965 | NM\_026503 | Mus musculus RIKEN cDNA 1110058L19 gene (1110058L19Rik), mRNA | 1.85 | 0,0030 |
| A\_51\_P283876 | NM\_026512 | BIPHENYL HYDROLASE-RELATED PROTEIN homolog [Homo sapiens] | 1.85 | 0,0009 |
| A\_51\_P224575 | NM\_010322 | Mus musculus glyceronephosphate O-acyltransferase (Gnpat), mRNA | 1.85 | 0,0011 |
| A\_51\_P269073 | NM\_019986 | Mus musculus hyaluronic acid binding protein 4 (Habp4), mRNA | 1.85 | 0,0062 |
| A\_51\_P371743 | NM\_134248 | Mus musculus T-cell immunoglobulin and mucin domain containing 1 (Timd1), mRNA | 1.85 | 0,0133 |
| A\_51\_P164203 | NM\_019731 | Mus musculus expressed in non-metastatic cells 4, protein (NM23-M4)(nucleoside diphosphate kinase) (Nme4), mRNA | 1.85 | 0,0009 |
| A\_51\_P234466 | XM\_132974 | Mus musculus Abcc10 mRNA, partial sequence | 1.85 | 0,0015 |
| A\_51\_P492070 | NM\_133953 | Mus musculus expressed sequence AA409318 (AA409318), mRNA | 1.85 | 0,0006 |
| A\_51\_P398235 | AK053156 | hypothetical DnaJ N-terminal domain containing protein | 1.85 | 0,0072 |
| A\_51\_P477941 | NM\_013907 | Mus musculus f-box and WD-40 domain protein 4 (Fbxw4), mRNA | 1.85 | 0,0019 |
| A\_51\_P284095 | AK076456 | DJ876B10 2 NOVEL PROTEIN ORTHOLOG OF RAT | 1.84 | 0,0053 |
| A\_51\_P343016 | NM\_145830 | G9A homolog [Mus musculus] | 1.84 | 0,0026 |
| A\_51\_P205170 | NM\_146175 | ZINC FINGER PROTEIN 282 (HTLV-I U5RE BINDING PROTEIN 1) (HUB-1) homolog [Homo sapiens] | 1.84 | 0,0002 |
| A\_51\_P330298 | NM\_031999 | Mus musculus transmembrane 7 superfamily member 1 (Tm7sf1), mRNA | 1.84 | 0,0021 |
| A\_51\_P395160 | NM\_175503 | A5D3 PROTEIN homolog [Rattus norvegicus] | 1.84 | 0,0300 |
| A\_51\_P488422 | BC050879 | hypothetical Adenosine and AMP deaminase containing protein | 1.84 | 0,0001 |
| A\_51\_P203771 | NM\_025351 | Mus musculus, Similar to RIKEN cDNA 0710001P09 gene, clone MGC:19393 IMAGE:3153325, mRNA, complete cds | 1.84 | 0,0109 |
| A\_51\_P302405 | AK006462 | hypothetical RNA-binding region RNP-1 (RNA recognition motif) containing protein | 1.84 | 0,0011 |
| A\_51\_P217430 | NM\_023633 | Mus musculus RIKEN cDNA 2410016O06 gene (2410016O06Rik), mRNA | 1.84 | 0,0003 |
| A\_51\_P193302 | NM\_025305 | mitchondrial ribosomal protein S7 | 1.84 | 0,0038 |
| A\_51\_P136848 | AK050977 | unclassifiable | 1.84 | 0,0058 |
| A\_51\_P314285 | NM\_026436 | hypothetical protein | 1.84 | 0,0122 |
| A\_51\_P464199 | NM\_178143 | inferred: 5-AMP-activated protein kinase catalytic alpha-2 subunit {Rattus norvegicus} | 1.84 | 0,0023 |
| A\_51\_P441327 | NM\_175034 | hypothetical K+-dependent Na+/Ca+ exchanger related-protein containing protein | 1.84 | 0,0009 |
| A\_51\_P397154 | NAP063254-1 | Mus musculus similar to Oxysterols receptor LXR-beta (Liver X receptor beta) | 1.84 | 0,0212 |
| A\_51\_P343208 | XM\_127466 | unknown EST | 1.84 | 0,0151 |
| A\_51\_P280244 | NM\_011751 | Mus musculus zinc finger protein 207 (Zfp207), mRNA | 1.84 | 0,0008 |
| A\_51\_P167963 | NM\_011792 | Mus musculus beta-site APP cleaving enzyme (Bace), mRNA | 1.84 | 0,0001 |
| A\_51\_P428903 | AK016770 | Mus musculus RIKEN cDNA 4933411G06 gene (4933411G06Rik), mRNA | 1.83 | 0,0099 |
| A\_51\_P356283 | NM\_133765 | Mus musculus RIKEN cDNA 2310046N15 gene (2310046N15Rik), mRNA | 1.83 | 0,0265 |
| A\_51\_P373149 | NM\_177737 | weakly similar to ZNF74=KRUPPEL-TYPE ZINC FINGER (22 KRUPPEL-RELATED ZINC FINGER PROTEIN) (FRAGMENT) [Homo sapiens] | 1.83 | 0,0002 |
| A\_51\_P224928 | AK010706 | Mus musculus ELAV (embryonic lethal, abnormal vision, Drosophila)-like 1 (Hu antigen R) (Elavl1), mRNA | 1.83 | 0,0207 |
| A\_51\_P318343 | NM\_026962 | hypothetical BTB/POZ domain/Microbodies C-terminal targeting signal/Kelch repeat containing protein | 1.83 | 0,0191 |
| A\_51\_P226417 | NM\_178376 | RAGA (RAS-RELATED GTP-BINDING PROTEIN) homolog [Homo sapiens] | 1.83 | 0,0053 |
| A\_51\_P104747 | NM\_010364 | Mus musculus general transcription factor II H, polypeptide 4 (Gtf2h4), mRNA | 1.83 | 0,0028 |
| A\_51\_P189704 | NM\_025732 | Mus musculus RIKEN cDNA 4921520G13 gene (4921520G13Rik), mRNA | 1.83 | 0,0239 |
| A\_51\_P120008 | BC068151 | similar to 3 of D-CONTAINING PROTEIN (FRAGMENT) [Rattus norvegicus] | 1.83 | 0,0007 |
| A\_51\_P227417 | AK044553 | G-PROTEIN COUPLED RECEPTOR homolog [Homo sapiens] | 1.83 | 0,0091 |
| A\_51\_P451837 | AK017353 | unknown EST | 1.83 | 0,0147 |
| A\_51\_P213459 | AK046920 | unclassifiable | 1.83 | 0,0037 |
| A\_51\_P181175 | NM\_021447 | Mus musculus ring finger protein 30 (Rnf30), mRNA | 1.83 | 0,0001 |
| A\_51\_P490155 | NM\_020271 | Mus musculus hypothetical protein, MNCb-4193 (AB041662), mRNA | 1.83 | 0,0117 |
| A\_51\_P500574 | NM\_025894 | Mus musculus proteasome (prosome, macropain) 26S subunit, non-ATPase, 12 (Psmd12), mRNA | 1.83 | 0,0001 |
| A\_51\_P408071 | XM\_132322 | hypothetical protein | 1.83 | 0,0018 |
| A\_51\_P486068 | NM\_177475 | DNA segment, Chr 10, Johns Hopkins University 82, expressed | 1.83 | 0,0079 |
| A\_51\_P423527 | NM\_175007 | Mus musculus, Similar to amphiphysin, clone IMAGE:5357091, mRNA, partial cds | 1.83 | 0,0044 |
| A\_51\_P321067 | NM\_027121 | hypothetical protein | 1.83 | 0,0392 |
| A\_51\_P215368 | NM\_172271 | SODIUM AND CHLORIDE DEPENDENT TRANSPORTER | 1.83 | 0,0228 |
| A\_51\_P309744 | NM\_026229 | hypothetical protein | 1.83 | 0,0011 |
| A\_51\_P463994 | NM\_021393 | Mus musculus hypothetical protein, MNCb-5210 (AB041607), mRNA | 1.83 | 0,0042 |
| A\_51\_P413315 | X04663 | Mus musculus tubulin, beta 5 (Tubb5), mRNA | 1.83 | 0,0009 |
| A\_51\_P160413 | NM\_178691 | hypothetical protein | 1.83 | 0,0046 |
| A\_51\_P260639 | NM\_028811 | elongation protein 3 homolog (S. cerevisiae) | 1.83 | 0,0046 |
| A\_51\_P107228 | NM\_013854 | Similar to ATP-binding cassette, sub-family F (GCN20), member 1, clone IMAGE:6485365, mRNA | 1.82 | 0,0276 |
| A\_51\_P110010 | NM\_027946 | WD REPEAT PROTEIN AN11 | 1.82 | 0,0007 |
| A\_51\_P397493 | NM\_017401 | Mus musculus polymerase (DNA directed), mu (Polm), mRNA | 1.82 | 0,0086 |
| A\_51\_P171200 | NM\_027307 | unknown EST | 1.82 | 0,0060 |
| A\_51\_P384639 | NM\_026154 | Mus musculus mitochondrial ribosomal protein L10 (Mrpl10), mRNA | 1.82 | 0,0004 |
| A\_51\_P360615 | NM\_008730 | Mus musculus neuronal pentraxin 1 (Nptx1), mRNA | 1.82 | 0,0151 |
| A\_51\_P356871 | NAP057003-1 | Mus musculus guanine nucleotide binding protein (G protein), gamma 2 subunit, pseudogene 1 (Gng2-ps1) on chromosome 4 | 1.82 | 0,0205 |
| A\_51\_P259638 | NM\_008016 | fibroblast growth factor inducible 15 | 1.82 | 0,0146 |
| A\_51\_P415475 | NM\_053246 | Mus musculus downstream of tyrosine kinase 4 (Dok4), mRNA | 1.82 | 0,0086 |
| A\_51\_P125446 | NM\_026963 | LEUCINE ZIPPER & ICAT HOMOLOGOUS PROTEIN LZIC homolog [Homo sapiens] | 1.82 | 0,0010 |
| A\_51\_P353592 | NM\_025417 | Mus musculus RIKEN cDNA 1110039H05 gene (1110039H05Rik), mRNA | 1.82 | 0,0030 |
| A\_51\_P221132 | NM\_145443 | Mus musculus, Similar to hypothetical protein FLJ12618, clone MGC:28775 IMAGE:4487011, mRNA, complete cds | 1.82 | 0,0075 |
| A\_51\_P357203 | XM\_144310 | Mus musculus, clone IMAGE:4024509, mRNA | 1.82 | 0,0053 |
| A\_51\_P491147 | BC022600 | KELCH LIKE PROTEIN | 1.80 | 0,0119 |
| A\_51\_P516016 | NM\_011473 | Mus musculus small proline-rich protein 2G (Sprr2g), mRNA | 1.80 | 0,0289 |
| A\_51\_P442091 | AK122352 | unknown EST | 1.80 | 0,0181 |
| A\_51\_P199095 | NM\_025838 | similar to DC20 [Homo sapiens] | 1.80 | 0,0063 |
| A\_51\_P133247 | AK076014 | Mus musculus fatty acid transport protein 3 mRNA, partial cds | 1.80 | 0,0001 |
| A\_51\_P491595 | NM\_145930 | Mus musculus, clone MGC:12113 IMAGE:3709876, mRNA, complete cds | 1.80 | 0,0075 |
| A\_51\_P208811 | AK030894 | weakly similar to TESMIN (METALLOTHIONEIN-LIKE 5, TESTIS-SPECIFIC) (TESTIS-SPECIFIC METALLOTHIONEIN-LIKE PROTEIN) [Homo sapiens] | 1.80 | 0,0030 |
| A\_51\_P209071 | NM\_028469 | hypothetical protein | 1.80 | 0,0058 |
| A\_51\_P129929 | NM\_177899 | ZINC FINGER PROTEIN 5 (FRAGMENT) homolog [Rattus norvegicus] | 1.80 | 0,0050 |
| A\_51\_P490415 | NM\_025566 | Mus musculus RIKEN cDNA 2600017J23 gene (2600017J23Rik), mRNA | 1.80 | 0,0215 |
| A\_51\_P490817 | NM\_145494 | NAD-DEPENDENT MALIC ENZYME, MITOCHONDRIAL PRECURSOR (EC 1.1.1.38) (NAD-ME) homolog [Homo sapiens] | 1.80 | 0,0009 |
| A\_51\_P383524 | NM\_181728 | NAD(P)(+)--ARGININE ADP-RIBOSYLTRANSFERASE (EC 2.4.2.31) homolog [Mus musculus] | 1.80 | 0,0091 |
| A\_51\_P261359 | NM\_172692 | BILE ACID BETA-GLUCOSIDASE (KIAA1605 PROTEIN) homolog [Homo sapiens] | 1.80 | 0,0005 |
| A\_51\_P336471 | NM\_013842 | Mus musculus X-box binding protein 1 (Xbp1), mRNA | 1.80 | 0,0414 |
| A\_51\_P142450 | NM\_027865 | hypothetical Immunoglobulin structure containing protein | 1.80 | 0,0181 |
| A\_51\_P170911 | NM\_019651 | Mus musculus protein tyrosine phosphatase, non-receptor type 9 (Ptpn9), mRNA | 1.80 | 0,0013 |
| A\_51\_P253732 | NM\_027265 | hypothetical protein | 1.80 | 0,0258 |
| A\_51\_P273508 | BC065083 | unknown EST | 1.80 | 0,0076 |
| A\_51\_P399071 | NM\_009672 | Mus musculus cDNA, 5 end | 1.80 | 0,0034 |
| A\_51\_P246754 | NM\_134021 | Mus musculus expressed sequence AI415282 (AI415282), mRNA | 1.80 | 0,0023 |
| A\_51\_P263373 | NM\_177588 | hypothetical Pyridoxal-5-phosphate-dependent enzymes, beta family containing protein | 1.80 | 0,0339 |
| A\_51\_P144957 | NM\_146140 | weakly similar to translocating chain-associating membrane protein [Canis lupus familiaris] | 1.80 | 0,0248 |
| A\_51\_P386415 | AK020441 | unclassifiable | 1.80 | 0,0077 |
| A\_51\_P445562 | NM\_145142 | HNK-1 SULFOTRANSFERASE homolog [Mus musculus] | 1.80 | 0,0154 |
| A\_51\_P208870 | NM\_027476 | hypothetical DHHC-type Zn-finger containing protein | 1.80 | 0,0057 |
| A\_51\_P414034 | A\_51\_P414034 | Mus musculus cDNA, 3 end | 1.80 | 0,0075 |
| A\_51\_P371194 | NM\_013917 | PITUITARY TUMOR-TRANSFORMING 1 | 1.80 | 0,0040 |
| A\_51\_P174906 | BC029621 | PHOSPHORIBOSYLPYROPHOSPHATE SYNTHETASE-ASSOCIATED PROTEIN (39 KDA) (PHOSPHORIBOSYLPYROPHOSPHATE SYNTHETASE-ASSOCIATED PROTEIN 39) homolog [Rattus norvegicus] | 1.79 | 0,0015 |
| A\_51\_P510441 | NM\_011514 | Mus musculus suppressor of variegation 3-9 homolog 1 (Drosophila) (Suv39h1), mRNA | 1.79 | 0,0028 |
| A\_51\_P269634 | NM\_178733 | zinc finger protein 14 | 1.79 | 0,0156 |
| A\_51\_P332228 | NM\_199024 | similar to NOLP PROTEIN (HRIHFB2255 PROTEIN) [Homo sapiens] | 1.79 | 0,0174 |
| A\_51\_P311659 | AK031823 | unclassifiable | 1.79 | 0,0057 |
| A\_51\_P127492 | NM\_011228 | Mus musculus RAB33A, member of RAS oncogene family (Rab33a), mRNA | 1.79 | 0,0009 |
| A\_51\_P263473 | NM\_011889 | Mus musculus septin 3 (Sept3), mRNA | 1.79 | 0,0057 |
| A\_51\_P229715 | NM\_178367 | hypothetical protein | 1.79 | 0,0162 |
| A\_51\_P356353 | AK017654 | unknown EST | 1.79 | 0,0009 |
| A\_51\_P187841 | NM\_025829 | Mus musculus RIKEN cDNA 1300018P11 gene (1300018P11Rik), mRNA | 1.79 | 0,0112 |
| A\_51\_P207988 | NM\_008965 | Mus musculus prostaglandin E receptor 4 (subtype EP4) (Ptger4), mRNA | 1.79 | 0,0003 |
| A\_51\_P194681 | AK042945 | Mus musculus, Similar to hypothetical protein FLJ11305, clone IMAGE:3590683, mRNA | 1.79 | 0,0059 |
| A\_51\_P399924 | NM\_001005506 | inferred: dJ622L5.2 (novel protein) {Homo sapiens} | 1.79 | 0,0030 |
| A\_51\_P321651 | NM\_028221 | Mus musculus expressed sequence AI596259 (AI596259), mRNA | 1.79 | 0,0007 |
| A\_51\_P395741 | XM\_132143 | weakly similar to SIGNAL RECOGNITION PARTICLE 72 KDA PROTEIN (SRP72) [Canis familiaris] | 1.79 | 0,0005 |
| A\_51\_P513785 | NM\_007794 | Mus musculus CCCTC-binding factor (Ctcf), mRNA | 1.79 | 0,0023 |
| A\_51\_P490204 | NM\_026398 | Mus musculus RIKEN cDNA 1500019J17 gene (1500019J17Rik), mRNA | 1.79 | 0,0442 |
| A\_51\_P247614 | NM\_183030 | unknown EST | 1.79 | 0,0013 |
| A\_51\_P361286 | NM\_026792 | 1-ACYL-SN-GLYCEROL-3-PHOSPHATE ACYLTRANSFERASE EPSILON (EC 2.3.1.51) (1-AGP ACYLTRANSFERASE 5) (1-AGPAT 5) (LYSOPHOSPHATIDIC ACID ACYLTRANSFERASE-EPSILON) (LPAAT-EPSILON) (1-ACYLGLYCEROL-3-PHOSPHATE O-ACYLTRANSFERASE 5) | 1.78 | 0,0284 |
| A\_51\_P182456 | AK009638 | unknown EST | 1.78 | 0,0133 |
| A\_51\_P140091 | NM\_146018 | hypothetical protein | 1.78 | 0,0003 |
| A\_51\_P468240 | XM\_134902 | c-myc promoter-binding protein irlB (fragment) homolog [Homo sapiens] | 1.78 | 0,0011 |
| A\_51\_P400555 | NM\_026906 | Mus musculus cathepsin 3 (Cts3), mRNA | 1.78 | 0,0140 |
| A\_51\_P268274 | NM\_018872 | Mus musculus RW1 protein (Rw1-pending), mRNA | 1.78 | 0,0031 |
| A\_51\_P212518 | NM\_178726 | CDNA FLJ30553 FIS, CLONE BRAWH2003689, HIGHLY SIMILAR TO MUS MUSCULUS CLONE MOUSE1-9 PUTATIVE PROTEIN PHOSPHATASE TYPE 2C MRNA homolog [Homo sapiens] | 1.78 | 0,0321 |
| A\_51\_P490700 | NM\_175430 | weakly similar to KIAA1640 PROTEIN (FRAGMENT) [Homo sapiens] | 1.78 | 0,0413 |
| A\_51\_P368074 | NM\_028132 | Mus musculus, RIKEN cDNA 2610020G18 gene, clone MGC:6642 IMAGE:3495694, mRNA, complete cds | 1.78 | 0,0077 |
| A\_51\_P206518 | NM\_019542 | Mus musculus N-acetylglucosamine kinase (Nagk), mRNA | 1.78 | 0,0291 |
| A\_51\_P433837 | AK013991 | unknown EST | 1.78 | 0,0080 |
| A\_51\_P512172 | AK010953 | hypothetical protein | 1.78 | 0,0015 |
| A\_51\_P314893 | NM\_146014 | Mus musculus, Similar to hypothetical protein MGC4607, clone MGC:37115 IMAGE:4952288, mRNA, complete cds | 1.78 | 0,0085 |
| A\_51\_P109671 | NM\_023672 | Mus musculus, hypothetical protein FLJ10355, clone MGC:36847 IMAGE:4208359, mRNA, complete cds | 1.78 | 0,0075 |
| A\_51\_P447988 | NM\_018794 | Mus musculus ATPase, H+ transporting, lysosomal (vacuolar proton pump), subunit 1 (Atp6s1), mRNA | 1.78 | 0,0013 |
| A\_51\_P483589 | NM\_198162 | hypothetical protein | 1.78 | 0,0085 |
| A\_51\_P425632 | NM\_025649 | Mus musculus RIKEN cDNA 0610009D16 gene (0610009D16Rik), mRNA | 1.78 | 0,0047 |
| A\_51\_P295904 | NM\_028934 | Mus musculus cDNA, 3 end | 1.78 | 0,0043 |
| A\_51\_P468743 | NM\_025697 | CLLL6 PROTEIN homolog [Homo sapiens] | 1.78 | 0,0022 |
| A\_51\_P451847 | NM\_025814 | Mus musculus RIKEN cDNA 1200009K13 gene (1200009K13Rik), mRNA | 1.77 | 0,0031 |
| A\_51\_P462658 | NM\_008431 | Mus musculus potassium channel, subfamily K, member 4 (Kcnk4), mRNA | 1.77 | 0,0027 |
| A\_51\_P366152 | NM\_177591 | INHIBIN BINDING PROTEIN LONG ISOFORM homolog [Rattus norvegicus] | 1.77 | 0,0220 |
| A\_51\_P408946 | NM\_007633 | Mus musculus cyclin E1 (Ccne1), mRNA | 1.77 | 0,0023 |
| A\_51\_P261107 | NM\_139144 | UDP-N-ACETYLGLUCOSAMINYLTRANSFERASE homolog [Mus musculus] | 1.77 | 0,0062 |
| A\_51\_P363800 | NM\_023217 | Mus musculus RIKEN cDNA 2810003H13 gene (2810003H13Rik), mRNA | 1.77 | 0,0199 |
| A\_51\_P519385 | NM\_030692 | Mus musculus SAC1 (supressor of actin mutations 1, homolog)-like (S. cerevisiae) (Sacm1l), mRNA | 1.77 | 0,0056 |
| A\_51\_P146440 | NM\_146122 | Mus musculus, Similar to KIAA1608 protein, clone MGC:38416 IMAGE:5346095, mRNA, complete cds | 1.77 | 0,0316 |
| A\_51\_P220163 | NM\_008716 | Mus musculus Notch gene homolog 3, (Drosophila) (Notch3), mRNA | 1.77 | 0,0290 |
| A\_51\_P295575 | NM\_025530 | Mus musculus RIKEN cDNA 2310039I18 gene (2310039I18Rik), mRNA | 1.77 | 0,0072 |
| A\_51\_P215046 | NM\_133677 | Mus musculus RIKEN cDNA 2310061J03 gene (2310061J03Rik), mRNA | 1.77 | 0,0134 |
| A\_51\_P424079 | NM\_010122 | Mus musculus eukaryotic translation initiation factor 2B (Eif2b), mRNA | 1.77 | 0,0043 |
| A\_51\_P297480 | NM\_144491 | Mus musculus diptheria toxin resistance protein required for diphthamide biosynthesis (Saccharomyces)-like 1 (Dph2l1), mRNA | 1.77 | 0,0094 |
| A\_51\_P499802 | BC030380 | RIKEN cDNA C330021I08 gene | 1.77 | 0,0019 |
| A\_51\_P329413 | NM\_029786 | O-LINKED MANNOSE BETA1,2-N-ACETYLGLUCOSAMINYLTRANSFERASE homolog [Homo sapiens] | 1.77 | 0,0033 |
| A\_51\_P319379 | BC054558 | unknown EST | 1.77 | 0,0124 |
| A\_51\_P433556 | NM\_197993 | Mus musculus, Similar to hypothetical protein FLJ22347, clone MGC:38113 IMAGE:5320398, mRNA, complete cds | 1.77 | 0,0050 |
| A\_51\_P236412 | NM\_016912 | Mus musculus cyclin-dependent kinase-like 2 (CDC2-related kinase) (Cdkl2), mRNA | 1.77 | 0,0106 |
| A\_51\_P368262 | NM\_011630 | mTR2R1=type II zinc finger DNA binding transcription factor [mice, neonatal brain, mRNA, 2339 nt] | 1.77 | 0,0027 |
| A\_51\_P492446 | NM\_172401 | hypothetical Esterase/lipase/thioesterase family active site containing protein | 1.77 | 0,0118 |
| A\_51\_P222283 | NM\_019966 | malonyl-CoA decarboxylase | 1.77 | 0,0064 |
| A\_51\_P438679 | NM\_144806 | Mus musculus, phosphoribosyl pyrophosphate synthetase-associated protein 2, clone MGC:36957 IMAGE:4947226, mRNA, complete cds | 1.77 | 0,0027 |
| A\_51\_P228254 | NM\_026647 | Mus musculus RIKEN cDNA 9130404H11 gene (9130404H11Rik), mRNA | 1.77 | 0,0014 |
| A\_51\_P364578 | NM\_172836 | hypothetical protein | 1.77 | 0,0403 |
| A\_51\_P141502 | NM\_026623 | inferred: RIKEN cDNA 5730530J16 gene | 1.77 | 0,0030 |
| A\_51\_P396375 | NM\_011586 | Mus musculus MysPDZ mRNA for myosin containing PDZ domain, complete cds | 1.77 | 0,0089 |
| A\_51\_P470568 | NM\_023230 | Mus musculus, ubiquitin-conjugating enzyme E2 variant 1, clone MGC:6536 IMAGE:2654197, mRNA, complete cds | 1.75 | 0,0008 |
| A\_51\_P358171 | NM\_019488 | Mus musculus solute carrier family 2, (facilitated glucose transporter), member 8 (Slc2a8), mRNA | 1.75 | 0,0212 |
| A\_51\_P302921 | NM\_021449 | Mus musculus cDNA sequence AF229032 (AF229032), mRNA | 1.75 | 0,0176 |
| A\_51\_P148355 | NM\_175433 | hypothetical Zinc finger, C2H2 type containing protein | 1.75 | 0,0152 |
| A\_51\_P406414 | NM\_010470 | Mus musculus, heterochromatin protein 2, binding protein 3, clone MGC:28927 IMAGE:3597151, mRNA, complete cds | 1.75 | 0,0030 |
| A\_51\_P472630 | AK021267 | HISTONE H2A.F homolog [Homo sapiens] | 1.75 | 0,0481 |
| A\_51\_P351217 | NM\_175562 | RAS-RELATED PROTEIN RAB-39 homolog [Homo sapiens] | 1.75 | 0,0069 |
| A\_51\_P125745 | NM\_012055 | Mus musculus, Similar to asparagine synthetase, clone MGC:5995 IMAGE:3585775, mRNA, complete cds | 1.75 | 0,0037 |
| A\_51\_P188614 | NM\_011853 | Mouse mRNA for 2-5A synthetase | 1.75 | 0,0022 |
| A\_51\_P153977 | NM\_198322 | ZFP71P (FRAGMENT) homolog [Mus musculus] | 1.75 | 0,0043 |
| A\_51\_P227703 | XM\_485005 | hypothetical Cysteine-rich region containing protein | 1.75 | 0,0163 |
| A\_51\_P184654 | AK122507 | inferred: RIKEN cDNA 5930418K15 gene | 1.75 | 0,0166 |
| A\_51\_P483922 | NM\_133847 | Mus musculus expressed sequence AU045326 (AU045326), mRNA | 1.75 | 0,0020 |
| A\_51\_P491835 | AK013507 | inferred: RIKEN cDNA 2900009J20 gene | 1.75 | 0,0261 |
| A\_51\_P125866 | XM\_207079 | Mus musculus spectrin alpha 2, mRNA (cDNA clone IMAGE:5353935), partial cds | 1.75 | 0,0124 |
| A\_51\_P293781 | NM\_016863 | Mus musculus FK506 binding protein 1b (12.6 kDa) (Fkbp1b), mRNA | 1.75 | 0,0051 |
| A\_51\_P299432 | NM\_026670 | Mus musculus RIKEN cDNA 5830412B09 gene (5830412B09Rik), mRNA | 1.75 | 0,0129 |
| A\_51\_P265145 | NM\_172751 | similar to KIAA0294 PROTEIN [Homo sapiens] | 1.75 | 0,0433 |
| A\_51\_P142465 | NM\_011239 | Mus musculus RAN binding protein 1 (Ranbp1), mRNA | 1.75 | 0,0014 |
| A\_51\_P502443 | BC085617 | similar to ADP-RIBOSYLATION FACTOR BINDING PROTEIN GGA3 (GOLGI-LOCALIZED, GAMMA EAR-CONTAINING, ARF-BINDING PROTEIN 3) [Homo sapiens] | 1.75 | 0,0291 |
| A\_51\_P307470 | NM\_028753 | Mus musculus RIKEN cDNA 0610037N12 gene (0610037N12Rik), mRNA | 1.75 | 0,0083 |
| A\_51\_P496448 | NM\_029402 | Mus musculus cullin 2 (Cul2), mRNA | 1.75 | 0,0219 |
| A\_51\_P289429 | AK048961 | KPL2 homolog [Rattus norvegicus] | 1.74 | 0,0063 |
| A\_51\_P494406 | NM\_145484 | Mus musculus, clone MGC:29357 IMAGE:5038660, mRNA, complete cds | 1.74 | 0,0140 |
| A\_51\_P168756 | NM\_178589 | Mus musculus cDNA, 5 end | 1.74 | 0,0066 |
| A\_51\_P196113 | NM\_019945 | Mus musculus syntrophin associated serine/threonine kinase (Sast-pending), mRNA | 1.74 | 0,0164 |
| A\_51\_P439452 | NM\_133748 | Mus musculus RIKEN cDNA 2900053I11 gene (2900053I11Rik), mRNA | 1.74 | 0,0013 |
| A\_51\_P301848 | NM\_133345 | Mus musculus DNA segment, Chr 6, Wayne State University 147, expressed (D6Wsu147e), mRNA | 1.74 | 0,0101 |
| A\_51\_P179953 | NM\_027922 | hypothetical Ankyrin-repeat containing protein | 1.74 | 0,0058 |
| A\_51\_P513661 | AK082224 | unknown EST | 1.74 | 0,0194 |
| A\_51\_P161413 | XM\_149655 | hypothetical Ubiquitin carboxyl-terminal hydrolase family 2 containing protein | 1.74 | 0,0021 |
| A\_51\_P282890 | NM\_009003 | Mus musculus RAB4A, member RAS oncogene family (Rab4a), mRNA | 1.74 | 0,0019 |
| A\_51\_P268824 | NM\_172659 | similar to SOLUTE CARRIER FAMILY 2, FACILITATED GLUCOSE TRANSPORTER, MEMBER 6 (GLUCOSE TRANSPORTER TYPE 6) (GLUCOSE TRANSPORTER TYPE 9) [Homo sapiens] | 1.74 | 0,0028 |
| A\_51\_P487668 | NM\_026230 | Mus musculus RIKEN cDNA 4933432H23 gene (4933432H23Rik), mRNA | 1.74 | 0,0012 |
| A\_51\_P197866 | AK035905 | hypothetical protein | 1.74 | 0,0264 |
| A\_51\_P189905 | NM\_010015 | Mus musculus defender against cell death 1 (Dad1), mRNA | 1.74 | 0,0040 |
| A\_51\_P208472 | NM\_029901 | Mus musculus RIKEN cDNA 9430025F20 gene (9430025F20Rik), mRNA | 1.74 | 0,0018 |
| A\_51\_P269173 | NM\_133981 | Mus musculus RIKEN cDNA 8230402H15 gene (8230402H15Rik), mRNA | 1.74 | 0,0027 |
| A\_51\_P344249 | NM\_009685 | Mus musculus Fe65 mRNA, complete cds | 1.74 | 0,0325 |
| A\_51\_P443618 | NM\_026686 | hypothetical S-adenosyl-L-methionine-dependent methyltransferases structure containing protein | 1.74 | 0,0275 |
| A\_51\_P174362 | NM\_028789 | hypothetical RING finger containing protein | 1.74 | 0,0060 |
| A\_51\_P174221 | BC057001 | Mus musculus cDNA, 5 end | 1.74 | 0,0041 |
| A\_51\_P110395 | XM\_486265 | HUNTINGTIN INTERACTING PROTEIN (FRAGMENT) homolog [Homo sapiens] | 1.74 | 0,0066 |
| A\_51\_P430620 | NM\_172436 | solute carrier family 25 (mitochondrial carrier, Aralar), member 12 | 1.74 | 0,0004 |
| A\_51\_P346722 | NM\_013865 | Mus musculus N-myc downstream regulated 3 (Ndr3), mRNA | 1.74 | 0,0311 |
| A\_51\_P195557 | NM\_026249 | Mus musculus RIKEN cDNA 4930429B21 gene (4930429B21Rik), mRNA | 1.74 | 0,0170 |
| A\_51\_P219532 | AK003388 | hypothetical protein | 1.74 | 0,0180 |
| A\_51\_P488554 | NM\_026543 | Mus musculus RIKEN cDNA 3010026O09 gene (3010026O09Rik), mRNA | 1.74 | 0,0225 |
| A\_51\_P461219 | NM\_178693 | similar to CGI-92 PROTEIN [Homo sapiens] | 1.74 | 0,0015 |
| A\_51\_P364031 | NM\_026213 | similar to OSMOSIS RESPONSIVE FACTOR [Homo sapiens] | 1.74 | 0,0048 |
| A\_51\_P222467 | NM\_009593 | Mus musculus ATP-binding cassette, sub-family G (WHITE), member 1 (Abcg1), mRNA | 1.74 | 0,0063 |
| A\_51\_P420859 | NM\_011773 | Mus musculus solute carrier family 30 (zinc transporter), member 3 (Slc30a3), mRNA | 1.73 | 0,0001 |
| A\_51\_P168439 | AK004187 | unknown EST | 1.73 | 0,0028 |
| A\_51\_P329975 | NM\_016718 | Mus musculus ninjurin 2 (Ninj2), mRNA | 1.73 | 0,0005 |
| A\_51\_P517145 | NM\_019972 | Mus musculus sortilin 1 (Sort1), mRNA | 1.73 | 0,0291 |
| A\_51\_P312576 | AK054222 | hypothetical protein | 1.73 | 0,0012 |
| A\_51\_P450829 | NM\_133987 | Mus musculus expressed sequence AA589632 (AA589632), mRNA | 1.73 | 0,0285 |
| A\_51\_P359018 | NM\_011841 | Mus musculus mitogen-activated protein kinase 7 (Mapk7), mRNA | 1.73 | 0,0275 |
| A\_51\_P509760 | NM\_016860 | Mus musculus actin-related protein 1 homolog A (yeast) (Actr1a), mRNA | 1.73 | 0,0028 |
| A\_51\_P467770 | NM\_028874 | similar to SORTING NEXIN 19 [Homo sapiens] | 1.73 | 0,0146 |
| A\_51\_P267690 | NM\_153397 | METALLOPROTEASE/DISINTEGRIN (FRAGMENT) homolog [Rattus norvegicus] | 1.73 | 0,0354 |
| A\_51\_P130203 | AK009939 | unknown EST | 1.73 | 0,0316 |
| A\_51\_P123731 | AK122438 | hypothetical 2-5-oligoadenylate synthetase containing protein | 1.73 | 0,0094 |
| A\_51\_P394946 | NM\_024438 | Mus musculus dual specificity phosphatase 19 (Dusp19), mRNA | 1.73 | 0,0034 |
| A\_51\_P240594 | NM\_145916 | Mus musculus, Similar to zinc finger protein 7 (KOX 4, clone HF.16), clone MGC:6397 IMAGE:3584546, mRNA, complete cds | 1.73 | 0,0130 |
| A\_51\_P473576 | NM\_009687 | Mus musculus apurinic/apyrimidinic endonuclease (Apex), mRNA | 1.73 | 0,0208 |
| A\_51\_P204730 | NM\_025892 | Mus musculus RIKEN cDNA 1500031L02 gene (1500031L02Rik), mRNA | 1.73 | 0,0003 |
| A\_51\_P392134 | NM\_080560 | Mus musculus ubiquitin-conjugating enzyme E2N (Ube2n), mRNA | 1.73 | 0,0078 |
| A\_51\_P186118 | NM\_024201 | Mus musculus RIKEN cDNA 0610011N22 gene (0610011N22Rik), mRNA | 1.73 | 0,0159 |
| A\_51\_P354913 | NM\_027402 | hypothetical Fibronectin type III domain containing protein | 1.73 | 0,0168 |
| A\_51\_P144770 | XM\_619964 | Mus musculus, clone MGC:27669 IMAGE:4910895, mRNA, complete cds | 1.73 | 0,0008 |
| A\_51\_P103745 | NM\_010075 | Mus musculus strain C57Bl/6 dipeptidyl aminopeptidase-like protein 6 embryonic isoform (Dpp6) mRNA, complete cds | 1.73 | 0,0233 |
| A\_51\_P105755 | AK011730 | weakly similar to BA162G10.3 (ZINC FINGER PROTEIN) (FRAGMENT) [Homo sapiens] | 1.73 | 0,0079 |
| A\_51\_P389908 | NM\_013881 | Mus musculus Unc-51 like kinase 2 (C. elegans) (Ulk2), mRNA | 1.72 | 0,0157 |
| A\_51\_P232474 | NM\_028028 | similar to DJ337O18.5.2 (NOVEL PROTEIN, ISOFORM 2) [Homo sapiens] | 1.72 | 0,0100 |
| A\_51\_P458852 | TC1429894 | Mus musculus internexin neuronal intermediate filament protein, alpha (Ina), mRNA | 1.72 | 0,0420 |
| A\_51\_P118671 | NM\_033566 | Mus musculus SWI/SNF related, matrix associated, actin dependent regulator of chromatin, subfamily f, member 1 (Smarcf1), mRNA | 1.72 | 0,0033 |
| A\_51\_P119597 | NM\_009680 | Mus musculus adaptor-related protein complex AP-3, beta 1 subunit (Ap3b1), mRNA | 1.72 | 0,0059 |
| A\_51\_P517834 | AK012806 | hypothetical S-adenosyl-L-methionine-dependent methyltransferases structure containing protein | 1.72 | 0,0075 |
| A\_51\_P468780 | NM\_025812 | Mus musculus high mobility group 20A (Hmg20a), mRNA | 1.72 | 0,0158 |
| A\_51\_P260721 | NM\_016902 | Mus musculus nephronophthisis 1 (juvenile) homolog (human) (Nphp1), mRNA | 1.72 | 0,0124 |
| A\_51\_P495767 | NM\_011948 | Mus musculus mitogen activated protein kinase kinase kinase 4 (Map3k4), mRNA | 1.72 | 0,0083 |
| A\_51\_P182752 | NM\_028331 | weakly similar to COMPLEMENT-C1Q TUMOR NECROSIS FACTOR-RELATED PROTEIN [Homo sapiens] | 1.72 | 0,0384 |
| A\_51\_P111757 | AK122456 | unknown EST | 1.72 | 0,0040 |
| A\_51\_P399477 | AK079183 | unknown EST | 1.72 | 0,0194 |
| A\_51\_P385598 | NM\_008063 | Mus musculus glucose-6-phosphatase, transport protein 1 (G6pt1), mRNA | 1.72 | 0,0134 |
| A\_51\_P397876 | NM\_010134 | Mus musculus engrailed 2 (En2), mRNA | 1.72 | 0,0035 |
| A\_51\_P285042 | XM\_283757 | unknown EST | 1.72 | 0,0019 |
| A\_51\_P252154 | NM\_201226 | hypothetical B3/B4 domain of PheRS, PheT structure containing protein | 1.72 | 0,0158 |
| A\_51\_P255931 | NM\_146116 | Mus musculus, tubulin, beta, 2, clone MGC:28623 IMAGE:4221189, mRNA, complete cds | 1.72 | 0,0142 |
| A\_51\_P164995 | NM\_025285 | Mus musculus superiorcervical ganglia, neural specific 10 (Scgn10), mRNA | 1.72 | 0,0420 |
| A\_51\_P352824 | NM\_026369 | Mus musculus actin related protein 2/3 complex, subunit 5 (165 kDa) (Arpc5), mRNA | 1.72 | 0,0094 |
| A\_51\_P185248 | NM\_145741 | growth-differentiation factor-10=bone morphogenetic protein-3 homolog [mice, CD-1, uterus, mRNA, 2322 nt] | 1.72 | 0,0046 |
| A\_51\_P450887 | NM\_022982 | Mus musculus reticulon 4 receptor (Rtn4r), mRNA | 1.72 | 0,0119 |
| A\_51\_P295355 | NM\_011957 | Mus musculus old astrocyte specifically induced substance (Oasis-pending), mRNA | 1.72 | 0,0234 |
| A\_51\_P173723 | NM\_177303 | H1 1 PUTATIVE LEUCINE RICH | 1.72 | 0,0038 |
| A\_51\_P117449 | AK019191 | Wolf-Hirschhorn syndrome candidate 2 homolog (human) | 1.72 | 0,0114 |
| A\_51\_P150608 | NM\_026365 | Mus musculus RIKEN cDNA 5830427H10 gene (5830427H10Rik), mRNA | 1.72 | 0,0122 |
| A\_51\_P144868 | NM\_029934 | Mus musculus, RIKEN cDNA 5730589L02 gene, clone MGC:32334 IMAGE:5028759, mRNA, complete cds | 1.72 | 0,0207 |
| A\_51\_P328516 | NM\_145493 | Mus musculus, Similar to hypothetical protein FLJ13993, clone MGC:27587 IMAGE:4501240, mRNA, complete cds | 1.72 | 0,0008 |
| A\_51\_P467682 | NM\_027115 | hypothetical protein | 1.72 | 0,0080 |
| A\_51\_P213051 | NM\_019752 | Mus musculus protease, serine, 25 (Prss25), mRNA | 1.72 | 0,0021 |
| A\_51\_P493709 | AK013405 | hypothetical protein | 1.72 | 0,0320 |
| A\_51\_P151775 | NM\_178719 | hypothetical protein | 1.72 | 0,0178 |
| A\_51\_P190845 | NM\_007509 | Mus musculus ATPase, H+ transporting, lysosomal (vacuolar proton pump), beta 56/58 kDa, isoform 2 (Atp6b2), mRNA | 1.72 | 0,0298 |
| A\_51\_P461416 | XM\_129704 | Mus musculus voltage-gated potassium channel KCNQ5 (Kcnq5) mRNA, partial cds | 1.72 | 0,0138 |
| A\_51\_P513611 | NM\_146078 | unknown EST | 1.72 | 0,0117 |
| A\_51\_P342897 | BC019508 | Mus musculus RIKEN cDNA 2010013B10 gene (2010013B10Rik), mRNA | 1.71 | 0,0348 |
| A\_51\_P128147 | BC036138 | CHARGED MULTIVESICULAR BODY PROTEIN 1/CHROMATIN MODIFYING PROTEIN 1 (UNKNOWN) (PROTEIN FOR MGC:15100) homolog [Homo sapiens] | 1.71 | 0,0284 |
| A\_51\_P207403 | NM\_133993 | Mus musculus RIKEN cDNA 2310058A11 gene (2310058A11Rik), mRNA | 1.71 | 0,0005 |
| A\_51\_P242076 | NM\_025666 | hypothetical PHD-finger/Putative zinc finger in N-recognin containing protein | 1.71 | 0,0115 |
| A\_51\_P168768 | A\_51\_P168768 | Mus musculus cDNA, 3 end | 1.71 | 0,0160 |
| A\_51\_P483690 | X61450 | Mus musculus brain protein 14 (Brp14), mRNA | 1.71 | 0,0030 |
| A\_51\_P426994 | NM\_153591 | hypothetical Aminoacyl-transfer RNA synthetases class-II containing protein | 1.71 | 0,0021 |
| A\_51\_P476129 | NM\_029546 | PERIODIC TRYPTOPHAN PROTEIN 2 HOMOLOG homolog [Homo sapiens] | 1.71 | 0,0071 |
| A\_51\_P330481 | NM\_010714 | Mus musculus LIM homeobox protein 9 (Lhx9), mRNA | 1.71 | 0,0045 |
| A\_51\_P260912 | 2810416G20 | unknown EST | 1.71 | 0,0007 |
| A\_51\_P222453 | NM\_025311 | RIKEN cDNA 0610008K04 gene | 1.71 | 0,0032 |
| A\_51\_P154469 | NM\_010761 | Mus musculus maternal inhibition of differentiation (Maid), mRNA | 1.71 | 0,0036 |
| A\_51\_P455572 | NM\_025631 | Mus musculus RIKEN cDNA 2310034L21 gene (2310034L21Rik), mRNA | 1.71 | 0,0033 |
| A\_51\_P373379 | NM\_009975 | casein kinase II, beta subunit | 1.71 | 0,0010 |
| A\_51\_P280914 | TC1481637 | Mus musculus cDNA | 1.71 | 0,0015 |
| A\_51\_P199187 | NM\_026062 | weakly similar to PANCREATITIS-INDUCED PROTEIN 49 [Mus musculus] | 1.71 | 0,0052 |
| A\_51\_P461191 | NM\_029674 | ASPARTATE AMINOTRANSFERASE, CYTOPLASMIC EC 2.6.1.1 TRANSAMINASE A GLUTAMATE OXALOACETATE TRANSAMINASE | 1.71 | 0,0011 |
| A\_51\_P323878 | NM\_030205 | 70 KDA WD-REPEAT TUMOR REJECTION ANTIGEN HOMOLOG homolog [Mus musculus] | 1.71 | 0,0041 |
| A\_51\_P384618 | NM\_026144 | Mus musculus RIKEN cDNA 3222401G21 gene (3222401G21Rik), mRNA | 1.71 | 0,0202 |
| A\_51\_P411757 | NM\_172945 | inferred: KE03 protein {Homo sapiens} | 1.71 | 0,0052 |
| A\_51\_P421724 | NM\_012028 | Mus musculus sialyltransferase 7 ((alpha-N-acetylneuraminyl 2,3-betagalactosyl-1,3)-N-acetyl galactosaminide alpha-2,6-sialyltransferase) E (Siat7e), mRNA | 1.71 | 0,0186 |
| A\_51\_P178081 | NM\_009049 | Mus musculus regulated endocrine-specific protein 18 (Resp18), mRNA | 1.71 | 0,0464 |
| A\_51\_P313483 | NM\_007890 | Mus musculus mnb protein kinase homolog mp86 (Dyrk) mRNA, complete cds | 1.71 | 0,0139 |
| A\_51\_P188605 | NM\_198424 | similar to SIMILAR TO CAP-BINDING PROTEIN COMPLEX INTERACTING PROTEIN 2 [Homo sapiens] | 1.71 | 0,0002 |
| A\_51\_P123805 | XM\_355637 | Mus musculus, Similar to strawberry notch, clone IMAGE:3597470, mRNA | 1.71 | 0,0086 |
| A\_51\_P386113 | AK050937 | unknown EST | 1.71 | 0,0199 |
| A\_51\_P341025 | NM\_028659 | MRNA OF MUSCLE SPECIFIC GENE M9, COMPLETE CDS (ARG134 PROTEIN) homolog [Homo sapiens] | 1.71 | 0,0060 |
| A\_51\_P445495 | NM\_026661 | BA234K24.2 (A NOVEL PROTEIN (CGI-23, DKFZP564N1363)) (SIMILAR TO DKFZP564N1363 PROTEIN) homolog [Homo sapiens] | 1.71 | 0,0269 |
| A\_51\_P489069 | NM\_146257 | Similar to: Mus musculus solute carrier family 29 (nucleoside transporters), member 4 (Slc29a4), mRNA | 1.71 | 0,0125 |
| A\_51\_P430678 | AK004405 | similar to KU70-BINDING PROTEIN (FRAGMENT) [Homo sapiens] | 1.69 | 0,0149 |
| A\_51\_P256617 | AK047064 | splicing factor, arginine/serine rich 9 (25 kDa) | 1.69 | 0,0090 |
| A\_51\_P202418 | NM\_053187 | Mus musculus PDZ protein interacting specifically with TC10 (Pist-pending), mRNA | 1.69 | 0,0251 |
| A\_51\_P209930 | NM\_013648 | Mus musculus reticulon 2 (Z-band associated protein) (Rtn2), mRNA | 1.69 | 0,0167 |
| A\_51\_P279851 | NM\_201408 | Mus musculus RIKEN cDNA 1500041N16 gene (1500041N16Rik), mRNA | 1.69 | 0,0035 |
| A\_51\_P509229 | AK014218 | unknown EST | 1.69 | 0,0028 |
| A\_51\_P357138 | NM\_019861 | Mus musculus cathepsin F (Ctsf), mRNA | 1.69 | 0,0108 |
| A\_51\_P138830 | NM\_198108 | Mus musculus similar to GH04877p [Drosophila melanogaster] (LOC226123), mRNA | 1.69 | 0,0110 |
| A\_51\_P127777 | NM\_018740 | Mus musculus retinoic acid induced 12 (Rai12), mRNA | 1.69 | 0,0004 |
| A\_51\_P189251 | NM\_013823 | Mus musculus klotho (Kl), mRNA | 1.69 | 0,0466 |
| A\_51\_P240864 | NM\_029012 | Mus musculus, clone MGC:29009 IMAGE:3154534, mRNA, complete cds | 1.69 | 0,0118 |
| A\_51\_P449449 | A\_51\_P449449 | "Similar to: Mus musculus similar to RP42 homolog� squamous cell carcinoma-related oncogene [Homo sapiens] (LOC234073), mRNA" | 1.69 | 0,0056 |
| A\_51\_P229602 | AK028562 | PLEXIN 2 | 1.69 | 0,0234 |
| A\_51\_P400375 | NM\_021446 | Mus musculus open reading frame 11 (ORF11), mRNA | 1.69 | 0,0019 |
| A\_51\_P319449 | NM\_144784 | ACETYL-COA ACETYLTRANSFERASE, MITOCHONDRIAL PRECURSOR (EC 2.3.1.9) (ACETOACETYL-COA THIOLASE) homolog [Rattus norvegicus] | 1.69 | 0,0071 |
| A\_51\_P120066 | AK034052 | hypothetical Myc-type, helix-loop-helix dimerization domain containing protein | 1.69 | 0,0022 |
| A\_51\_P182104 | NM\_152823 | hypothetical Death domain containing protein | 1.69 | 0,0066 |
| A\_51\_P370380 | NM\_023697 | Mus musculus RIKEN cDNA 3110030G19 gene (3110030G19Rik), mRNA | 1.69 | 0,0023 |
| A\_51\_P109364 | NM\_175322 | hypothetical protein | 1.69 | 0,0039 |
| A\_51\_P505694 | NM\_008916 | Mus musculus putative phosphatase (Pps), mRNA | 1.69 | 0,0005 |
| A\_51\_P173212 | NM\_144500 | Mus musculus oxysterol-binding protein-like 2 (Osbpl2), mRNA | 1.69 | 0,0045 |
| A\_51\_P445765 | TC1519807 | Mus musculus cDNA, 5 end | 1.69 | 0,0004 |
| A\_51\_P240064 | NM\_177462 | weakly similar to ZNF258 [Homo sapiens] | 1.69 | 0,0180 |
| A\_51\_P169783 | AK086378 | SIMILAR TO CONSERVED GENE TELOMERIC TO ALPHA GLOBIN CLUSTER (UNKNOWN) (PROTEIN FOR MGC:4491) homolog [Homo sapiens] | 1.69 | 0,0017 |
| A\_51\_P339356 | NM\_178607 | RING FINGER PROTEIN 24 homolog [Homo sapiens] | 1.69 | 0,0069 |
| A\_51\_P352738 | NM\_033564 | Mus musculus Mpv17 transgene, kidney disease mutant-like (Mpv17l), mRNA | 1.69 | 0,0409 |
| A\_51\_P239984 | NM\_012012 | Mus musculus exonuclease 1 (Exo1), mRNA | 1.69 | 0,0017 |
| A\_51\_P355773 | NM\_026921 | Mus musculus, RIKEN cDNA 1810010A06 gene, clone MGC:19368 IMAGE:2615786, mRNA, complete cds | 1.69 | 0,0075 |
| A\_51\_P111352 | NM\_175154 | N-ACETYLGALACTOSAMINE KINASE (EC 2.7.1.-) (GALNAC KINASE) (GALACTOKINASE 2) homolog [Homo sapiens] | 1.69 | 0,0002 |
| A\_51\_P218278 | AK032906 | PROBABLE G PROTEIN COUPLED RECEPTOR SUPER CONSERVED RECEPTOR EXPRESSED IN BRAIN | 1.69 | 0,0120 |
| A\_51\_P242978 | XM\_284451 | hypothetical protein | 1.69 | 0,0021 |
| A\_51\_P400216 | NM\_016982 | Mus musculus pre-B lymphocyte gene 1 (Vpreb1), mRNA | 1.69 | 0,0012 |
| A\_51\_P202623 | NM\_028832 | similar to TRANSCRIPTION TERMINATION FACTOR-LIKE PROTEIN [Homo sapiens] | 1.68 | 0,0061 |
| A\_51\_P493700 | XM\_129509 | unknown EST | 1.68 | 0,0077 |
| A\_51\_P352894 | AK085726 | BREFELDIN A-INHIBITED GUANINE NUCLEOTIDE-EXCHANGE PROTEIN 1 (BREFELDIN A-INHIBITED GEP 1) (P200 ARF-GEP1) (P200 ARF GUANINE NUCLEOTIDE EXCHANGE FACTOR) homolog [Homo sapiens] | 1.68 | 0,0091 |
| A\_51\_P361204 | NM\_013477 | Mus musculus ATPase, H+ transporting, lysosomal (vacuolar proton pump), 42 kDa (Atp6d), mRNA | 1.68 | 0,0071 |
| A\_51\_P258561 | AK046448 | musculus, clone IMAGE:5025694, mRNA | 1.68 | 0,0010 |
| A\_51\_P298033 | BC027567 | Mus musculus, clone IMAGE:3472070, mRNA | 1.68 | 0,0004 |
| A\_51\_P298538 | NM\_145404 | ARGININE N-METHYLTRANSFERASE P82 ISOFORM homolog [Cricetulus longicaudatus] | 1.68 | 0,0187 |
| A\_51\_P516950 | BC059894 | Mus musculus cDNA, 5 end | 1.68 | 0,0288 |
| A\_51\_P124647 | AK018169 | hypothetical Aspartic acid-rich region containing protein | 1.68 | 0,0396 |
| A\_51\_P326434 | NM\_025827 | Mus musculus RIKEN cDNA 1300002A08 gene (1300002A08Rik), mRNA | 1.68 | 0,0030 |
| A\_51\_P276063 | NM\_010726 | Mus musculus phytanoyl-CoA hydroxylase (Phyh), mRNA | 1.68 | 0,0107 |
| A\_51\_P499160 | NM\_028173 | TRAM PROTEIN TRANSLOCATING CHAIN ASSOCIATING MEMBRANE | 1.68 | 0,0446 |
| A\_51\_P240536 | NM\_027159 | Mus musculus, clone MGC:25689 IMAGE:3491825, mRNA, complete cds | 1.68 | 0,0054 |
| A\_51\_P156927 | XM\_354675 | RETINOBLASTOMA-BINDING PROTEIN 1 (RBBP-1) homolog [Homo sapiens] | 1.68 | 0,0113 |
| A\_51\_P341349 | NM\_024433 | 5-methylthioadenosine phosphorylase (EC 2.4.2.28) homolog [Homo sapiens] | 1.68 | 0,0118 |
| A\_51\_P256285 | NM\_024209 | Mus musculus, Similar to protein phosphatase 6, catalytic subunit, clone MGC:7488 IMAGE:3491111, mRNA, complete cds | 1.68 | 0,0015 |
| A\_51\_P364560 | AK013846 | hypothetical protein | 1.68 | 0,0133 |
| A\_51\_P447471 | NM\_172662 | Mus musculus RIKEN cDNA E330008O22 gene (E330008O22Rik), mRNA | 1.68 | 0,0090 |
| A\_51\_P360896 | NM\_029236 | hypothetical CheR-type MCP methyl-transferase containing protein | 1.68 | 0,0102 |
| A\_51\_P240329 | AK045401 | similar to SIMILAR TO RNA BINDING PROTEIN (FRAGMENT) [Mus musculus domesticus] | 1.68 | 0,0364 |
| A\_51\_P332165 | NM\_026077 | Mus musculus RIKEN cDNA 3110040N11 gene (3110040N11Rik), mRNA | 1.68 | 0,0037 |
| A\_51\_P220278 | NM\_028392 | SERINE/THREONINE PROTEIN PHOSPHATASE 2A, 55 KDA REGULATORY SUBUNIT B, BETA ISOFORM (PP2A, SUBUNIT B, B-BETA ISOFORM) (PP2A, SUBUNIT B, B55- BETA ISOFORM) (PP2A, SUBUNIT B, PR55-BETA ISOFORM) (PP2A, SUBUNIT B, R2-BETA ISOFORM) homolog [Homo sapiens] | 1.68 | 0,0148 |
| A\_51\_P518219 | AB041543 | Mus musculus expressed sequence AI448780 (AI448780), mRNA | 1.68 | 0,0122 |
| A\_51\_P325124 | NM\_177583 | CGI 78 | 1.68 | 0,0358 |
| A\_51\_P496590 | NM\_146099 | hypothetical Proline-rich region/Cytochrome c family heme-binding site containing protein | 1.68 | 0,0052 |
| A\_51\_P299155 | NM\_133937 | Mus musculus RIKEN cDNA 6720456B07 gene (6720456B07Rik), mRNA | 1.68 | 0,0062 |
| A\_51\_P236175 | NM\_009069 | RAS-LIKE PROTEIN EXPRESSED IN MANY TISSUES (RIT) (SIMILAR TO RAS-LIKE PROTEIN EXPRESSED IN MANY TISSUES) homolog [Mus musculus] | 1.67 | 0,0102 |
| A\_51\_P517787 | NM\_146047 | CISPLATIN RESISTANCE RELATED PROTEIN CRR9P homolog [Homo sapiens] | 1.67 | 0,0018 |
| A\_51\_P498863 | NM\_053168 | Mus musculus tripartite motif protein 11 (Trim11), mRNA | 1.67 | 0,0206 |
| A\_51\_P220934 | NM\_026955 | hypothetical Immunoglobulin subtype containing protein | 1.67 | 0,0018 |
| A\_51\_P432779 | XM\_134593 | unknown EST | 1.67 | 0,0076 |
| A\_51\_P259879 | NM\_173430 | CDNA FLJ12576 FIS, CLONE NT2RM4001032 (UNKNOWN) (PROTEIN FOR MGC:2991) (FUKUTIN-RELATED PROTEIN) homolog [Homo sapiens] | 1.67 | 0,0003 |
| A\_51\_P247665 | NM\_178811 | Mus musculus RIKEN cDNA D930014A20 gene (D930014A20Rik), mRNA | 1.67 | 0,0273 |
| A\_51\_P413710 | NM\_007460 | Mus musculus adaptor-related protein complex AP-3, delta subunit (Ap3d), mRNA | 1.67 | 0,0059 |
| A\_51\_P110753 | NM\_012031 | Mus musculus sperm associated antigen 1 (Spag1), mRNA | 1.67 | 0,0294 |
| A\_51\_P226134 | NM\_172422 | hypothetical protein | 1.67 | 0,0021 |
| A\_51\_P139103 | AK017728 | HYPOTHETICAL 25.7 KDA PROTEIN homolog [Homo sapiens] | 1.67 | 0,0273 |
| A\_51\_P375406 | NM\_144861 | unknown EST | 1.67 | 0,0361 |
| A\_51\_P108269 | NM\_145609 | CDNA FLJ14884 FIS, CLONE PLACE1003669, WEAKLY SIMILAR TO TRICHOHYALIN homolog [Homo sapiens] | 1.67 | 0,0140 |
| A\_51\_P152216 | NM\_025693 | Mus musculus, Similar to RIKEN cDNA 5730578N08 gene, clone IMAGE:3983912, mRNA | 1.67 | 0,0196 |
| A\_51\_P363537 | BC058094 | small optic lobes homolog (Drosophila) | 1.67 | 0,0196 |
| A\_51\_P440707 | BC049250 | Mus musculus RIKEN cDNA 2410004N11 gene (2410004N11Rik), mRNA | 1.67 | 0,0048 |
| A\_51\_P277389 | NM\_020494 | Mus musculus DEAD/H (Asp-Glu-Ala-Asp/His) box polypeptide 13 (RNA helicase A) (Ddx24), mRNA | 1.67 | 0,0130 |
| A\_51\_P388801 | NM\_178385 | SIMILAR TO TUBULIN-SPECIFIC CHAPERONE C homolog [Mus musculus] | 1.67 | 0,0399 |
| A\_51\_P274852 | NM\_028133 | Mus musculus EGL nine homolog 3 (C. elegans) (Egln3), mRNA | 1.67 | 0,0060 |
| A\_51\_P489108 | NM\_153391 | Mus musculus WD repeat membrane protein PWDMP (PWDMP), mRNA | 1.67 | 0,0008 |
| A\_51\_P298514 | NM\_177345 | Mus musculus expressed sequence AI591529 (AI591529), mRNA | 1.67 | 0,0076 |
| A\_51\_P486738 | NM\_010247 | Mus musculus thyroid autoantigen 70 kDa (G22p1), mRNA | 1.67 | 0,0375 |
| A\_51\_P153190 | NM\_133185 | Mus musculus RIKEN cDNA 0610011C19 gene (0610011C19Rik), mRNA | 1.67 | 0,0212 |
| A\_51\_P486568 | ENSMUST00000036357 | Mus musculus olfactory receptor MOR103-11 (MOR103-11), mRNA | 1.67 | 0,0034 |
| A\_51\_P315411 | NM\_013813 | Mus musculus erythrocyte protein band 4.1-like 3 (Epb4.1l3), mRNA | 1.66 | 0,0331 |
| A\_51\_P104172 | NM\_153802 | ZINC FINGER PROTEIN 4 (FRAGMENT) homolog [Rattus norvegicus] | 1.66 | 0,0193 |
| A\_51\_P198387 | NM\_026765 | hypothetical PRTase-like structure containing protein | 1.66 | 0,0157 |
| A\_51\_P114136 | NM\_177294 | hypothetical protein | 1.66 | 0,0073 |
| A\_51\_P508411 | NM\_177856 | hypothetical Staphylococcus nuclease (SNase) homologues containing protein | 1.66 | 0,0045 |
| A\_51\_P101719 | NM\_025978 | Mus musculus RIKEN cDNA 2700016E08 gene (2700016E08Rik), mRNA | 1.66 | 0,0133 |
| A\_51\_P383628 | NM\_126165 | Mus musculus vacuolar protein sorting protein 4a (Vps4a), mRNA | 1.66 | 0,0002 |
| A\_51\_P134835 | NM\_207685 | Mus musculus ELAV (embryonic lethal, abnormal vision, Drosophila)-like 2 (Hu antigen B) (Elavl2), mRNA | 1.66 | 0,0207 |
| A\_51\_P213691 | NM\_011324 | Mus musculus epithelial sodium channel alpha subunit mRNA, complete cds | 1.66 | 0,0014 |
| A\_51\_P326699 | NM\_007938 | Mus musculus Eph receptor A6 (Epha6), mRNA | 1.66 | 0,0266 |
| A\_51\_P447752 | NM\_027978 | hypothetical UbiA prenyltransferase containing protein | 1.66 | 0,0168 |
| A\_51\_P183985 | BC023061 | hypothetical protein | 1.66 | 0,0102 |
| A\_51\_P359445 | NM\_030219 | hypothetical B-box zinc finger domain/TNFR/CD27/30/40/95 cysteine-rich region/Fibronectin type III domain/RING finger containing protein | 1.66 | 0,0053 |
| A\_51\_P284318 | NM\_054082 | Mus musculus metastasis associated 3 (Mta3), mRNA | 1.66 | 0,0022 |
| A\_51\_P138923 | NM\_013788 | Mus musculus frequently rearranged in advanced T-cell lymphomas 3 (Frat3), mRNA | 1.66 | 0,0032 |
| A\_51\_P259360 | NM\_181417 | CRP2 BINDING PROTEIN (FRAGMENT) homolog [Homo sapiens] | 1.66 | 0,0041 |
| A\_51\_P513059 | NM\_175327 | hypothetical protein | 1.66 | 0,0115 |
| A\_51\_P372585 | NM\_019742 | Mus musculus fusion 1 (Fus1-pending), mRNA | 1.66 | 0,0037 |
| A\_51\_P415806 | NM\_026954 | hypothetical protein | 1.66 | 0,0265 |
| A\_51\_P327021 | NM\_028356 | weakly similar to ZINC FINGER PROTEIN 46 (ZINC FINGER PROTEIN KUP) [Homo sapiens] | 1.66 | 0,0049 |
| A\_51\_P121252 | NM\_027256 | Mus musculus, clone MGC:19455 IMAGE:3582093, mRNA, complete cds | 1.66 | 0,0051 |
| A\_51\_P105800 | XM\_146511 | hypothetical Protein phosphatase 2C domain/Leucine-rich repeat, typical subtype containing protein | 1.66 | 0,0062 |
| A\_51\_P411694 | BC023699 | Mus musculus, clone IMAGE:4009229, mRNA, partial cds | 1.66 | 0,0299 |
| A\_51\_P446645 | AK044675 | inferred: expressed sequence AI256776 | 1.66 | 0,0073 |
| A\_51\_P142421 | NM\_138683 | Mus musculus thrombospondin type 1 domain (R-spondin), mRNA | 1.66 | 0,0431 |
| A\_51\_P493558 | NM\_145417 | Mus musculus, Similar to aminopeptidase B, clone MGC:29229 IMAGE:5041005, mRNA, complete cds | 1.66 | 0,0160 |
| A\_51\_P364372 | NM\_001005916 | hypothetical BTB/POZ domain containing protein | 1.66 | 0,0079 |
| A\_51\_P277767 | NM\_024199 | CLEAVAGE STIMULATION FACTOR, 3 PRE-RNA, SUBUNIT 1, 50KD homolog [Homo sapiens] | 1.65 | 0,0210 |
| A\_51\_P372186 | NM\_020589 | Mus musculus hypothetical protein, MNCb-3350 (AB041616), mRNA | 1.65 | 0,0141 |
| A\_51\_P118539 | AK012399 | hypothetical protein | 1.65 | 0,0186 |
| A\_51\_P466221 | NM\_144547 | Mus musculus MIS type II receptor (Misrii) mRNA, complete cds | 1.65 | 0,0016 |
| A\_51\_P279623 | NM\_030240 | Mus musculus, clone MGC:7316 IMAGE:3485982, mRNA, complete cds | 1.65 | 0,0025 |
| A\_51\_P106000 | AK049629 | similar to KIAA1211 PROTEIN (FRAGMENT) [Homo sapiens] | 1.65 | 0,0460 |
| A\_51\_P290521 | NM\_153780 | unknown EST | 1.65 | 0,0463 |
| A\_51\_P164014 | AK049676 | hypothetical Lipocalin-related protein and Bos/Can/Equ allergen containing protein | 1.65 | 0,0063 |
| A\_51\_P498909 | NM\_146805 | Mus musculus olfactory receptor MOR165-5 (MOR165-5), mRNA | 1.65 | 0,0015 |
| A\_51\_P156697 | AK015837 | hypothetical Zinc finger, C2H2 type containing protein | 1.65 | 0,0015 |
| A\_51\_P444874 | NM\_008702 | Mus musculus nemo like kinase (Nlk), mRNA | 1.65 | 0,0066 |
| A\_51\_P381778 | AK011294 | Mus musculus RIKEN cDNA 2610002K22 gene (2610002K22Rik), mRNA | 1.65 | 0,0118 |
| A\_51\_P284966 | NM\_177648 | HYPOTHETICAL PROTEIN KIAA1094 homolog [Homo sapiens] | 1.65 | 0,0151 |
| A\_51\_P109119 | AK017113 | hypothetical protein | 1.65 | 0,0010 |
| A\_51\_P260548 | NM\_009149 | Mus musculus selectin, endothelial cell, ligand (Selel), mRNA | 1.65 | 0,0137 |
| A\_51\_P340807 | AI508699 | Mus musculus cDNA, 3 end | 1.65 | 0,0023 |
| A\_51\_P176459 | NM\_017479 | Mus musculus histone acetyltransferase (Morf-pending), mRNA | 1.65 | 0,0037 |
| A\_51\_P346826 | NM\_017475 | Mus musculus small GTPase, homolog (S. cerevisiae) (Gtr2), mRNA | 1.65 | 0,0106 |
| A\_51\_P460904 | BU592977 | Mus musculus cDNA, 5 end | 1.65 | 0,0026 |
| A\_51\_P285770 | NM\_010192 | feminization 1 homolog a (C. elegans) | 1.65 | 0,0021 |
| A\_51\_P195316 | NM\_018859 | Mus musculus aldo-keto reductase family 1, member E1 (Akr1e1), mRNA | 1.65 | 0,0220 |
| A\_51\_P160372 | NM\_016757 | Mus musculus WW domain binding protein 1 (Wbp1), mRNA | 1.65 | 0,0406 |
| A\_51\_P227463 | NM\_172754 | hypothetical Zinc finger, C2H2 type containing protein | 1.65 | 0,0163 |
| A\_51\_P284976 | NM\_015807 | Mus musculus 5,3-nucleotidase, cytosolic (Nt5c), mRNA | 1.65 | 0,0135 |
| A\_51\_P450018 | NM\_025933 | Mus musculus RIKEN cDNA 2010110M21 gene (2010110M21Rik), mRNA | 1.65 | 0,0018 |
| A\_51\_P333159 | NM\_026653 | Mus musculus replication protein A1 (70 kDa) (Rpa1), mRNA | 1.65 | 0,0071 |
| A\_51\_P498631 | NM\_018769 | Mus musculus deafness, autosomal dominant 5 homolog (human) (Dfna5h), mRNA | 1.65 | 0,0135 |
| A\_51\_P452109 | AK009711 | Mus musculus RIKEN cDNA 2310040A13 gene (2310040A13Rik), mRNA | 1.65 | 0,0141 |
| A\_51\_P306885 | NM\_025595 | Mus musculus mitochondrial ribosomal protein 64 (Mrp64), mRNA | 1.65 | 0,0058 |
| A\_51\_P340355 | NM\_172310 | THREONYL TRNA SYNTHETASE, CYTOPLASMIC EC 6.1.1.3 THREONINE TRNA LIGASE | 1.65 | 0,0354 |
| A\_51\_P472219 | NM\_199301 | Mus musculus, Similar to hypothetical protein BC004409, clone MGC:28365 IMAGE:4019717, mRNA, complete cds | 1.65 | 0,0031 |
| A\_51\_P338837 | NM\_030695 | Mus musculus LPS-responsive beige-like anchor (Lrba), mRNA | 1.65 | 0,0011 |
| A\_51\_P242265 | NM\_009950 | Mus musculus CASP2 and RIPK1 domain containing adaptor with death domain (Cradd), mRNA | 1.65 | 0,0101 |
| A\_51\_P424054 | BC055704 | BTB (POZ) domain containing 2 | 1.65 | 0,0137 |
| A\_51\_P338515 | NM\_134138 | Mus musculus expressed sequence AW545363 (AW545363), mRNA | 1.65 | 0,0369 |
| A\_51\_P380201 | AK042138 | Mus musculus cDNA, 5 end | 1.65 | 0,0065 |
| A\_51\_P131600 | NM\_172712 | MOP-4 homolog [Homo sapiens] | 1.65 | 0,0225 |
| A\_51\_P431734 | NM\_177869 | hypothetical protein | 1.64 | 0,0138 |
| A\_51\_P473918 | BC022619 | KIAA0374 (SYNTAPHILIN) (BA314N13.1.1) homolog [Homo sapiens] | 1.64 | 0,0356 |
| A\_51\_P460404 | BC016248 | Mus musculus, Similar to zinc finger protein 85 (HPF4, HTF1), clone MGC:28872 IMAGE:4527362, mRNA, complete cds | 1.64 | 0,0094 |
| A\_51\_P225083 | NM\_153680 | Mus musculus, Similar to sorting nexin 17, clone MGC:36375 IMAGE:4986268, mRNA, complete cds | 1.64 | 0,0079 |
| A\_51\_P203547 | AK015642 | Mus musculus RIKEN cDNA 4930488B01 gene (4930488B01Rik), mRNA | 1.64 | 0,0237 |
| A\_51\_P403413 | NM\_053131 | Mus musculus protocadherin beta 6 (Pcdhb6), mRNA | 1.64 | 0,0296 |
| A\_51\_P340854 | NM\_008436 | Mus musculus K+ voltage-gated channel, subfamily S, 2 (Kcns2), mRNA | 1.64 | 0,0015 |
| A\_51\_P292527 | NM\_028944 | hypothetical EF-hand/2OG-Fe(II) oxygenase superfamily containing protein | 1.64 | 0,0494 |
| A\_51\_P514776 | NM\_009771 | Mus musculus beta-transducin repeat containing protein (Btrc), mRNA | 1.64 | 0,0088 |
| A\_51\_P201945 | NM\_025747 | unknown EST | 1.64 | 0,0225 |
| A\_51\_P448987 | NM\_021714 | Mus musculus WW domain binding protein 11 (Wbp11), mRNA | 1.64 | 0,0416 |
| A\_51\_P490924 | NM\_177900 | CORE PROTEIN PRECURSOR PROTEOGLYCAN CORE PROTEIN | 1.64 | 0,0244 |
| A\_51\_P369623 | NM\_133949 | Mus musculus prostate tumor over expressed gene 1 (Ptov1), mRNA | 1.64 | 0,0413 |
| A\_51\_P224682 | NM\_008310 | Mus musculus 5-hydroxytryptamine (serotonin) receptor 1F (Htr1f), mRNA | 1.64 | 0,0080 |
| A\_51\_P366259 | NM\_010946 | Mus musculus N-terminal Asn amidase (Ntan1), mRNA | 1.64 | 0,0023 |
| A\_51\_P394074 | NM\_026255 | Mus musculus RIKEN cDNA 4930433D19 gene (4930433D19Rik), mRNA | 1.62 | 0,0009 |
| A\_51\_P179082 | NM\_011624 | Mus musculus topoisomerase (DNA) III beta (Top3b), mRNA | 1.62 | 0,0069 |
| A\_51\_P276960 | AK173113 | Mus musculus transcription repressor p66 (LOC229542), mRNA | 1.62 | 0,0266 |
| A\_51\_P142653 | NM\_178880 | SON PROTEIN homolog [Mus musculus] | 1.62 | 0,0056 |
| A\_51\_P229196 | NM\_026220 | Mus musculus microfibrillar-associated protein 1 (Mfap1), mRNA | 1.62 | 0,0097 |
| A\_51\_P390285 | NM\_026235 | Mus musculus RIKEN cDNA 5430431G03 gene (5430431G03Rik), mRNA | 1.62 | 0,0422 |
| A\_51\_P467751 | NM\_133970 | Mus musculus RIKEN cDNA 9430010M12 gene (9430010M12Rik), mRNA | 1.62 | 0,0008 |
| A\_51\_P106144 | NM\_009564 | Mus musculus zinc finger protein 64 (Zfp64), mRNA | 1.62 | 0,0027 |
| A\_51\_P388734 | NM\_008732 | Mus musculus solute carrier family 11 (proton-coupled divalent metal ion transporters), member 2 (Slc11a2), mRNA | 1.62 | 0,0061 |
| A\_51\_P352606 | NM\_009782 | Mus musculus calcium channel, voltage-dependent, R type, alpha 1E subunit (Cacna1e), mRNA | 1.62 | 0,0043 |
| A\_51\_P516994 | NM\_027886 | similar to LKB1-INTERACTING PROTEIN 1 [Homo sapiens] | 1.62 | 0,0011 |
| A\_51\_P440828 | NM\_026168 | PTX1 PROTEIN homolog [Homo sapiens] | 1.62 | 0,0172 |
| A\_51\_P431669 | NM\_178113 | hypothetical protein | 1.62 | 0,0282 |
| A\_51\_P230347 | NM\_177774 | SRRP35 homolog [Homo sapiens] | 1.62 | 0,0076 |
| A\_51\_P294340 | NM\_178599 | DNA segment, Chr 5, Bucan 26 expressed | 1.62 | 0,0175 |
| A\_51\_P209401 | BC062811 | weakly similar to PTD015 [Homo sapiens] | 1.62 | 0,0028 |
| A\_51\_P354766 | NM\_007944 | Mus musculus epidermal growth factor receptor pathway substrate 15, related sequence (Eps15-rs), mRNA | 1.62 | 0,0079 |
| A\_51\_P489821 | BC058274 | Mus musculus RIKEN cDNA 2610509D04 gene (2610509D04Rik), mRNA | 1.62 | 0,0093 |
| A\_51\_P459340 | NM\_025755 | hypothetical ARM repeat structure containing protein | 1.62 | 0,0071 |
| A\_51\_P281575 | NM\_029985 | Mus musculus, RIKEN cDNA A930011F22 gene, clone MGC:12118 IMAGE:3710091, mRNA, complete cds | 1.62 | 0,0043 |
| A\_51\_P307557 | XM\_131434 | similar to BRAIN PROTEIN (FRAGMENT) [Homo sapiens] | 1.62 | 0,0136 |
| A\_51\_P223111 | NM\_028661 | hypothetical protein | 1.62 | 0,0028 |
| A\_51\_P351697 | NM\_025430 | Mus musculus RIKEN cDNA 1110066C01 gene (1110066C01Rik), mRNA | 1.62 | 0,0004 |
| A\_51\_P476558 | BC027812 | Mus musculus, Similar to RIKEN cDNA 2010001C09 gene, clone IMAGE:2609442, mRNA | 1.62 | 0,0455 |
| A\_51\_P273197 | NM\_024227 | Mus musculus RIKEN cDNA 1110015G04 gene (1110015G04Rik), mRNA | 1.62 | 0,0403 |
| A\_51\_P444994 | NM\_177344 | hypothetical Prenyl group binding site (CAAX box) containing protein | 1.62 | 0,0157 |
| A\_51\_P288277 | AK003987 | unknown EST | 1.62 | 0,0467 |
| A\_51\_P242356 | NM\_026342 | hypothetical protein | 1.62 | 0,0041 |
| A\_51\_P220422 | NM\_011861 | Mus musculus protein kinase C and casein kinase substrate in neurons 1 (Pacsin1), mRNA | 1.62 | 0,0474 |
| A\_51\_P315646 | NM\_010911 | Mus musculus nitrogen fixation gene 1 (S. cerevisiae) (Nfs1), mRNA | 1.62 | 0,0011 |
| A\_51\_P410949 | BC066818 | similar to RNA POLYMERASE III SUBUNIT [Homo sapiens] | 1.61 | 0,0322 |
| A\_51\_P228193 | NM\_023429 | Mus musculus RIKEN cDNA 6030432N09 gene (6030432N09Rik), mRNA | 1.61 | 0,0060 |
| A\_51\_P425768 | NM\_026005 | similar to CDNA FLJ30600 FIS, CLONE BRAWH2009360 [Homo sapiens] | 1.61 | 0,0051 |
| A\_51\_P245564 | NM\_178621 | hypothetical Fibronectin type III domain containing protein | 1.61 | 0,0028 |
| A\_51\_P247478 | NM\_028105 | Mus musculus, RIKEN cDNA 2610005A10 gene, clone MGC:19303 IMAGE:4162365, mRNA, complete cds | 1.61 | 0,0070 |
| A\_51\_P484338 | NM\_001011846 | Mus musculus olfactory receptor MOR268-4 (MOR268-4) pseudogene | 1.61 | 0,0211 |
| A\_51\_P115772 | AK081204 | unknown EST | 1.61 | 0,0152 |
| A\_51\_P183822 | AK014401 | unknown EST | 1.61 | 0,0477 |
| A\_51\_P474158 | NM\_178605 | DNA SEGMENT, CHR 13, WAYNE STATE UNIVERSITY 177, EXPRESSED | 1.61 | 0,0282 |
| A\_51\_P367011 | NM\_207236 | OLFACTORY RECEPTOR | 1.61 | 0,0004 |
| A\_51\_P496735 | AK039103 | 39 KDA ANTIGEN homolog [Leishmania donovani] | 1.61 | 0,0025 |
| A\_51\_P466371 | NM\_008850 | Mus musculus phosphatidylinositol transfer protein (Pitpn), mRNA | 1.61 | 0,0276 |
| A\_51\_P410823 | NM\_023281 | SUCCINATE DEHYDROGENASE [UBIQUINONE] FLAVOPROTEIN SUBUNIT, MITOCHONDRIAL PRECURSOR EC 1.3.5.1 FP FLAVOPROTEIN SUBUNIT OF COMPLEX | 1.61 | 0,0061 |
| A\_51\_P114714 | NM\_023233 | Mus musculus tripartite motif protein 13 (Trim13), mRNA | 1.61 | 0,0224 |
| A\_51\_P484869 | NM\_010255 | Mus musculus guanidinoacetate methyltransferase (Gamt), mRNA | 1.61 | 0,0135 |
| A\_51\_P354744 | NM\_144874 | SIMILAR TO COX15 HOMOLOG, CYTOCHROME C OXIDASE ASSEMBLY PROTEIN (YEAST) (FRAGMENT) homolog [Mus musculus] | 1.61 | 0,0024 |
| A\_51\_P488937 | NM\_011182 | Mus musculus pleckstrin homology, Sec7 and coiled/coil domains 3 (Pscd3), mRNA | 1.61 | 0,0035 |
| A\_51\_P239673 | NM\_013556 | Mus musculus hypoxanthine guanine phosphoribosyl transferase (Hprt), mRNA | 1.61 | 0,0094 |
| A\_51\_P182216 | NM\_009822 | Mus musculus CBFA2T1 identified gene homolog (human) (Cbfa2t1h), mRNA | 1.61 | 0,0318 |
| A\_51\_P271665 | NM\_011264 | Mus musculus Sez4 mRNA for DNA polymerase, complete cds | 1.61 | 0,0175 |
| A\_51\_P160514 | NM\_021328 | Mus musculus bridging integrator 3 (Bin3), mRNA | 1.61 | 0,0004 |
| A\_51\_P330369 | NM\_138744 | Mus musculus expressed sequence AU014939 (AU014939), mRNA | 1.61 | 0,0211 |
| A\_51\_P231782 | AB093212 | Mus musculus, Similar to KIAA0171 gene product, clone IMAGE:3592699, mRNA, partial cds | 1.61 | 0,0400 |
| A\_51\_P463440 | NM\_130450 | Mus musculus long chain fatty acyl elongase (Lce-pending), mRNA | 1.61 | 0,0014 |
| A\_51\_P452875 | NM\_025457 | Mus musculus RIKEN cDNA 1810008A14 gene (1810008A14Rik), mRNA | 1.60 | 0,0350 |
| A\_51\_P398191 | AK028848 | hypothetical Histidine-rich region containing protein | 1.60 | 0,0169 |
| A\_51\_P329469 | NM\_173753 | SIMILAR TO PDZ DOMAIN CONTAINING GUANINE NUCLEOTIDE EXCHANGE FACTOR | 1.60 | 0,0259 |
| A\_51\_P420176 | NM\_177103 | hypothetical SUMO/Sentrin/Ubl1 specific protease containing protein | 1.60 | 0,0023 |
| A\_51\_P418165 | NM\_029103 | Mus musculus, Similar to arginine-rich, mutated in early stage tumors, clone IMAGE:4924742, mRNA | 1.60 | 0,0273 |
| A\_51\_P433506 | NM\_130866 | Mus musculus olfactory receptor 78 (Olfr78), mRNA | 1.60 | 0,0376 |
| A\_51\_P284728 | AK018518 | unknown EST | 1.60 | 0,0276 |
| A\_51\_P308590 | NM\_134084 | Mus musculus expressed sequence AW457192 (AW457192), mRNA | 1.60 | 0,0231 |
| A\_51\_P178646 | NM\_026308 | Mus musculus RIKEN cDNA 0610037N01 gene (0610037N01Rik), mRNA | 1.60 | 0,0051 |
| A\_51\_P375533 | NM\_027423 | CDNA FLJ10299 FIS, CLONE NT2RM2000013, MODERATELY SIMILAR TO DNA-DIRECTED RNA POLYMERASE III 128 KDA POLYPEPTIDE (EC 2.7.7.6) homolog [Homo sapiens] | 1.60 | 0,0375 |
| A\_51\_P288505 | XM\_134502 | similar to TNF RECEPTOR TYPE 1 ASSOCIATED DEATH DOMAIN PROTEIN | 1.60 | 0,0016 |
| A\_51\_P312360 | NM\_023058 | Mus musculus membrane-associated tyrosine-and threonine-specific cdc2-inhibitory kinase (Pkmyt1-pending), mRNA | 1.60 | 0,0089 |
| A\_51\_P234833 | NM\_133789 | Mus musculus, clone IMAGE:3494615, mRNA, partial cds | 1.60 | 0,0339 |
| A\_51\_P279571 | NM\_025932 | synapse associated protein 1 | 1.60 | 0,0097 |
| A\_51\_P126337 | NM\_183064 | fibroblast growth factor 12 | 1.60 | 0,0154 |
| A\_51\_P403578 | AK007907 | hypothetical protein | 1.60 | 0,0048 |
| A\_51\_P369971 | XM\_129261 | hypothetical protein | 1.60 | 0,0268 |
| A\_51\_P150087 | NM\_172511 | hypothetical protein | 1.60 | 0,0088 |
| A\_51\_P381763 | Z14986 | Mus musculus S-adenosylmethionine decarboxylase 1 (Amd1), mRNA | 1.60 | 0,0267 |
| A\_51\_P189351 | XM\_131217 | inferred: Mus musculus, clone IMAGE:3590287, mRNA, partial cds / Unknown (protein for IMAGE:3590287) [Mus musculus] | 1.60 | 0,0051 |
| A\_51\_P145993 | NM\_026899 | PNAS-120 homolog [Homo sapiens] | 1.60 | 0,0285 |
| A\_51\_P155997 | NM\_029850 | B-cell CLL/lymphoma 7A | 1.60 | 0,0362 |
| A\_51\_P195100 | NM\_009190 | Mus musculus vacuolar protein sorting 4b (yeast) (Vps4b), mRNA | 1.60 | 0,0407 |
| A\_51\_P392293 | AK088478 | SIMILAR TO PYRUVATE DEHYDROGENASE KINASE, ISOENZYME 3 homolog [Mus musculus] | 1.60 | 0,0298 |
| A\_51\_P236439 | NM\_177322 | Mus musculus angiotensin receptor 1a (Agtr1a), mRNA | 1.60 | 0,0220 |
| A\_51\_P504962 | NM\_146165 | Mus musculus, clone MGC:36522 IMAGE:5371230, mRNA, complete cds | 1.60 | 0,0261 |
| A\_51\_P229875 | AK122457 | Mus musculus expressed sequence AI841796 (AI841796), mRNA | 1.60 | 0,0070 |
| A\_51\_P438235 | NM\_027591 | hypothetical Microbodies C-terminal targeting signal containing protein | 1.60 | 0,0066 |
| A\_51\_P239156 | NM\_019776 | Mus musculus staphylococcal nuclease domain containing 1 (Snd1-pending), mRNA | 1.60 | 0,0315 |
| A\_51\_P381749 | NM\_025531 | Mus musculus RIKEN cDNA 2310042G06 gene (2310042G06Rik), mRNA | 1.60 | 0,0102 |
| A\_51\_P434447 | NM\_010443 | Mus musculus heme oxygenase (decycling) 2 (Hmox2), mRNA | 1.60 | 0,0247 |
| A\_51\_P519555 | NM\_023120 | Mus musculus guanine nucleotide binding protein (G protein), beta polypeptide 1-like (Gnb1l), mRNA | 1.59 | 0,0245 |
| A\_51\_P370283 | NM\_011869 | Mus musculus thyroid hormone receptor-associated protein 100 kDa (Trap100-pending), mRNA | 1.59 | 0,0065 |
| A\_51\_P423423 | BC089320 | Mus musculus cDNA, 5 end | 1.59 | 0,0080 |
| A\_51\_P225048 | AJ250693 | SR528 PROTEIN (FRAGMENT) homolog [Mus musculus] | 1.59 | 0,0035 |
| A\_51\_P320552 | NM\_028730 | similar to KAIA2502 PROTEIN [Homo sapiens] | 1.59 | 0,0066 |
| A\_51\_P367223 | NM\_009012 | Mus musculus RAD50 homolog (S. cerevisiae) (Rad50), mRNA | 1.59 | 0,0093 |
| A\_51\_P483258 | NM\_145145 | inferred: protein O-mannosyltransferase 1 {Rattus norvegicus} | 1.59 | 0,0157 |
| A\_51\_P454691 | AK036647 | hypothetical protein | 1.59 | 0,0018 |
| A\_51\_P362202 | NM\_175308 | similar to CDNA FLJ13204 FIS, CLONE NT2RP3004507, WEAKLY SIMILAR TO MOB1 PROTEIN [Homo sapiens] | 1.59 | 0,0285 |
| A\_51\_P345274 | NM\_194268 | RIKEN full-length enriched, 12 days embryo spinal ganglion Mus musculus cDNA clone D130071B21 5, mRNA sequence | 1.59 | 0,0406 |
| A\_51\_P436386 | BC006738 | Mus musculus, clone MGC:7055 IMAGE:3156574, mRNA, complete cds | 1.59 | 0,0076 |
| A\_51\_P465600 | NM\_028344 | hypothetical protein | 1.59 | 0,0013 |
| A\_51\_P402775 | AK090134 | Mus musculus meiotic check point regulator (Mcpr), mRNA | 1.59 | 0,0301 |
| A\_51\_P176341 | NM\_028769 | HRD1 homolog [Homo sapiens] | 1.59 | 0,0017 |
| A\_51\_P440936 | NM\_013581 | Mus musculus low density lipoprotein B (Ldlb), mRNA | 1.59 | 0,0090 |
| A\_51\_P403881 | NM\_011574 | M.musculus tex292 mRNA (3region) | 1.59 | 0,0469 |
| A\_51\_P218975 | NM\_173417 | voltage-gated potassium channel alpha chain Kv9.3 homolog [Rattus norvegicus] | 1.59 | 0,0105 |
| A\_51\_P461452 | NM\_026275 | Mus musculus RIKEN cDNA 1200003M11 gene (1200003M11Rik), mRNA | 1.59 | 0,0152 |
| A\_51\_P462918 | NM\_023737 | Mus musculus RIKEN cDNA 1300002P22 gene (1300002P22Rik), mRNA | 1.59 | 0,0048 |
| A\_51\_P307063 | NM\_001001493 | Mus musculus, PTD008 protein, clone MGC:19453 IMAGE:3501288, mRNA, complete cds | 1.59 | 0,0171 |
| A\_51\_P314852 | NM\_001030014 | B2 3 PUTATIVE NOVEL ACYL TRANSFERASE ISOFORM | 1.59 | 0,0293 |
| A\_51\_P269728 | NM\_011186 | Mus musculus proteasome (prosome, macropain) subunit, beta type 5 (Psmb5), mRNA | 1.59 | 0,0053 |
| A\_51\_P297968 | NM\_027959 | SIMILAR TO PROTEIN DISULFIDE ISOMERASE-RELATED PROTEIN homolog [Mus musculus] | 1.59 | 0,0164 |
| A\_51\_P330090 | NM\_029640 | hypothetical protein | 1.59 | 0,0164 |
| A\_51\_P309889 | 5430410O10 | HYPOTHETICAL 27.0 KDA PROTEIN homolog [Mus musculus] | 1.59 | 0,0131 |
| A\_51\_P490840 | NM\_146077 | Mus musculus, Similar to tripartite motif-containing 31, clone MGC:37625 IMAGE:4990292, mRNA, complete cds | 1.59 | 0,0445 |
| A\_51\_P469898 | AK020659 | hypothetical protein | 1.58 | 0,0033 |
| A\_51\_P392943 | NM\_178728 | hypothetical Metallo-hydrolase/oxidoreductase structure containing protein | 1.58 | 0,0014 |
| A\_51\_P345714 | NM\_011647 | Mus musculus tuberous sclerosis 2 (Tsc2), mRNA | 1.58 | 0,0212 |
| A\_51\_P152826 | NM\_025872 | unknown EST | 1.58 | 0,0212 |
| A\_51\_P389216 | NM\_029528 | hypothetical protein | 1.58 | 0,0045 |
| A\_51\_P465473 | NM\_027453 | TRANSCRIPTION FACTOR BTF3 RNA POLYMERASE B TRANSCRIPTION FACTOR | 1.58 | 0,0031 |
| A\_51\_P432659 | NM\_008822 | Mus musculus peroxisome biogenesis factor 7 (Pex7), mRNA | 1.58 | 0,0268 |
| A\_51\_P309677 | NM\_144517 | hypothetical RabGAP/TBC domain containing protein | 1.58 | 0,0139 |
| A\_51\_P108573 | NM\_175285 | hypothetical Serine/threonine specific protein phosphatase containing protein | 1.58 | 0,0349 |
| A\_51\_P253359 | NM\_025670 | Mus musculus RIKEN cDNA 5730403B10 gene (5730403B10Rik), mRNA | 1.58 | 0,0093 |
| A\_51\_P394471 | NM\_011548 | transcription factor E2a | 1.58 | 0,0015 |
| A\_51\_P100785 | AK009218 | NUCLEAR PROTEIN SKIP (SKI-INTERACTING PROTEIN) (SNW1 PROTEIN) (NUCLEAR RECEPTOR COACTIVATOR NCOA-62) homolog [Homo sapiens] | 1.58 | 0,0039 |
| A\_51\_P417469 | NM\_026616 | Mus musculus RIKEN cDNA 1500026D16 gene (1500026D16Rik), mRNA | 1.58 | 0,0370 |
| A\_51\_P518959 | 1110025P21 | Mus musculus ORM1-like 2 (S. cerevisiae) (Ormdl2), mRNA | 1.58 | 0,0279 |
| A\_51\_P320137 | NM\_030260 | Mus musculus hypothetical protein, MGC:7160 (BC003332), mRNA | 1.58 | 0,0137 |
| A\_51\_P334490 | NM\_172286 | hypothetical protein | 1.58 | 0,0415 |
| A\_51\_P438952 | NM\_026121 | Mus musculus BCL2-associated athanogene 4 (Bag4), mRNA | 1.58 | 0,0294 |
| A\_51\_P361580 | NM\_008997 | Mus musculus RAB11B, member RAS oncogene family (Rab11b), mRNA | 1.58 | 0,0050 |
| A\_51\_P186887 | NM\_080562 | Mus musculus ubiquitin conjugating enzyme 7 interacting protein 5 (Ubce7ip5-pending), mRNA | 1.58 | 0,0062 |
| A\_51\_P319425 | AK047081 | Mus musculus, Similar to down-regulated in metastasis, clone IMAGE:3494784, mRNA, partial cds | 1.58 | 0,0153 |
| A\_51\_P325501 | NM\_053250 | Mus musculus thymus LIM protein (Tlmp-pending), mRNA | 1.58 | 0,0214 |
| A\_51\_P114307 | NM\_008917 | Mus musculus palmitoyl-protein thioesterase (Ppt), mRNA | 1.58 | 0,0122 |
| A\_51\_P212023 | NM\_016926 | Mus musculus squamous cell carcinoma antigen recognized by T-cells 3 (Sart3), mRNA | 1.58 | 0,0143 |
| A\_51\_P304510 | NM\_145590 | Mus musculus, Similar to hypothetical protein FLJ13868, clone MGC:28903 IMAGE:4919869, mRNA, complete cds | 1.58 | 0,0027 |
| A\_51\_P222522 | NM\_023403 | Mus musculus mesoderm development candiate 2 (Mesdc2), mRNA | 1.58 | 0,0045 |
| A\_51\_P207292 | NM\_001005223 | similar to THYROID RECEPTOR INTERACTING PROTEIN 3 (TRIP-3) (FRAGMENT) [Homo sapiens] | 1.58 | 0,0363 |
| A\_51\_P434979 | NM\_029098 | LIPOCALIN-1 INTERACTING MEMBRANE RECEPTOR (LIPOCALIN-INTERACTING PROTEIN) homolog [Homo sapiens] | 1.58 | 0,0062 |
| A\_51\_P310539 | NM\_025381 | VACUOLAR ATP SYNTHASE SUBUNIT F (EC 3.6.3.14) (V-ATPASE F SUBUNIT) (VACUOLAR PROTON PUMP F SUBUNIT) (V-ATPASE 14 KDA SUBUNIT) homolog [Homo sapiens] | 1.58 | 0,0072 |
| A\_51\_P458998 | BC016258 | Mus musculus, Similar to RIKEN cDNA 1200011A11 gene, clone IMAGE:4913359, mRNA, partial cds | 1.58 | 0,0102 |
| A\_51\_P431772 | AK006243 | weakly similar to NADH-UBIQUINONE OXIDOREDUCTASE B9 SUBUNIT (EC 1.6.5.3) (EC 1.6.99.3) (COMPLEX I-B9) (CI-B9) [Mus musculus] | 1.58 | 0,0154 |
| A\_51\_P354683 | AK016104 | Mus musculus RIKEN cDNA 4930553F24 gene (4930553F24Rik), mRNA | 1.58 | 0,0361 |
| A\_51\_P129866 | AK082766 | unknown EST | 1.58 | 0,0050 |
| A\_51\_P179741 | NM\_172656 | AMYOTROPHIC LATERAL SCLEROSIS 2 (CDNA FLJ14731 FIS, CLONE NT2RP3001938, WEAKLY SIMILAR TO SPORULATION-SPECIFIC PROTEIN 1) (AMYOTROPHIC LATERAL SCLEROSIS 2 (JUVENILE) CHROMOSOME REGION, CANDIDATE 2) (EC 2.7.1.-) homolog [Homo sapiens] | 1.58 | 0,0024 |
| A\_51\_P442264 | NM\_172620 | ARE1 homolog [Rattus norvegicus] | 1.57 | 0,0115 |
| A\_51\_P384500 | NM\_007590 | Mus musculus calmodulin 3 (Calm3), mRNA | 1.57 | 0,0041 |
| A\_51\_P253904 | NM\_008704 | Mus musculus nucleoside diphosphate kinase A long form mRNA, complete cds | 1.57 | 0,0009 |
| A\_51\_P289881 | NM\_025334 | Mus musculus RIKEN cDNA 0610040B21 gene (0610040B21Rik), mRNA | 1.57 | 0,0151 |
| A\_51\_P483370 | NM\_145367 | Mus musculus, Similar to hypothetical protein MGC3178, clone MGC:28887 IMAGE:4911455, mRNA, complete cds | 1.57 | 0,0185 |
| A\_51\_P360374 | NM\_010665 | Mus musculus keratin complex 1, acidic, gene 2 (Krt1-2), mRNA | 1.57 | 0,0020 |
| A\_51\_P319031 | NM\_009945 | Mus musculus cytochrome c oxidase, subunit VIIa 3 (Cox7a3), mRNA | 1.57 | 0,0271 |
| A\_51\_P426677 | NM\_025403 | Mus musculus RIKEN cDNA 1110036B12 gene (1110036B12Rik), mRNA | 1.57 | 0,0435 |
| A\_51\_P498403 | NM\_028065 | Mus musculus putative retinoic acid-regulated protein mRNA, complete cds | 1.57 | 0,0090 |
| A\_51\_P127934 | NM\_025923 | Mus musculus RIKEN cDNA 2010322C19 gene (2010322C19Rik), mRNA | 1.57 | 0,0466 |
| A\_51\_P509083 | NM\_010214 | Mus musculus four and a half LIM domains 4 (Fhl4), mRNA | 1.57 | 0,0063 |
| A\_51\_P393968 | NM\_007701 | Mus musculus C. elegans ceh-10 homeo domain containing homolog (Chx10), mRNA | 1.57 | 0,0039 |
| A\_51\_P141521 | NM\_172965 | CDNA FLJ31417 FIS, CLONE NT2NE2000327, WEAKLY SIMILAR TO GLUCOAMYLASE S1/S2 PRECURSOR (EC 3.2.1.3) (FRAGMENT) homolog [Homo sapiens] | 1.57 | 0,0133 |
| A\_51\_P510849 | NM\_030018 | Mus musculus, RIKEN cDNA B230114J08 gene, clone MGC:6289 IMAGE:2649264, mRNA, complete cds | 1.57 | 0,0284 |
| A\_51\_P126525 | NM\_145604 | Mus musculus, Similar to CG7083 gene product, clone MGC:6480 IMAGE:2646515, mRNA, complete cds | 1.57 | 0,0280 |
| A\_51\_P458032 | AK085404 | hypothetical protein | 1.57 | 0,0375 |
| A\_51\_P210963 | AK036382 | unclassifiable | 1.57 | 0,0337 |
| A\_51\_P301435 | NM\_033149 | unknown EST | 1.57 | 0,0087 |
| A\_51\_P227502 | NM\_008546 | Mus musculus microfibrillar-associated protein 2 (Mfap2), mRNA | 1.57 | 0,0030 |
| A\_51\_P124039 | NM\_175294 | NUCLEAR UBIQUITOUS CASEIN AND CYCLIN-DEPENDENT KINASES SUBSTRATE homolog [Rattus norvegicus] | 1.57 | 0,0085 |
| A\_51\_P256566 | NM\_175164 | Mus musculus cDNA, 5 end | 1.57 | 0,0005 |
| A\_51\_P214306 | NM\_145462 | Similar to: Mus musculus similar to hypothetical protein FLJ20424 (LOC219072), mRNA | 1.57 | 0,0177 |
| A\_51\_P217737 | NM\_172773 | SIALIN homolog [Homo sapiens] | 1.56 | 0,0389 |
| A\_51\_P150394 | NM\_025842 | Mus musculus vacuolar protein sorting 28 (yeast) (Vps28), mRNA | 1.56 | 0,0056 |
| A\_51\_P121622 | AK004273 | unknown EST | 1.56 | 0,0066 |
| A\_51\_P189105 | AK077275 | Mus musculus (clone HIC-53) hydrogen peroxide-inducible protein mRNA, complete cds | 1.56 | 0,0114 |
| A\_51\_P512541 | AK049709 | similar to HCDI PROTEIN [Homo sapiens] | 1.56 | 0,0017 |
| A\_51\_P225056 | AK047799 | hypothetical protein | 1.56 | 0,0051 |
| A\_51\_P516446 | NM\_025344 | eukaryotic translation initiation factor 3, subunit 5 (epsilon) | 1.56 | 0,0240 |
| A\_51\_P288479 | NM\_181328 | hypothetical Mitochondrial energy transfer proteins (carrier protein) containing protein | 1.56 | 0,0076 |
| A\_51\_P368151 | BC016616 | hypothetical Myb DNA binding domain containing protein | 1.56 | 0,0354 |
| A\_51\_P402458 | NM\_009674 | Mus musculus annexin A7 (Anxa7), mRNA | 1.56 | 0,0172 |
| A\_51\_P487062 | AK029628 | Mus musculus olfactory receptor MOR124-1 pseudogene, partial sequence | 1.56 | 0,0015 |
| A\_51\_P414518 | NM\_019682 | Mus musculus dynein, cytoplasmic, light chain 1 (Dnclc1), mRNA | 1.56 | 0,0357 |
| A\_51\_P433989 | NM\_009158 | Mus musculus mitogen activated protein kinase 10 (Mapk10), mRNA | 1.56 | 0,0368 |
| A\_51\_P379660 | NM\_019914 | Mus musculus ALL1-fused gene from chromosome 1q (Af1q-pending), mRNA | 1.56 | 0,0276 |
| A\_51\_P261428 | NM\_011050 | Mus musculus programmed cell death 4 (Pdcd4), mRNA | 1.56 | 0,0460 |
| A\_51\_P294778 | NM\_172738 | similar to ZINC FINGER PROTEIN 12 (FRAGMENT) [Rattus norvegicus] | 1.56 | 0,0071 |
| A\_51\_P362879 | NM\_134136 | similar to 5-HT RECEPTOR [Homo sapiens] | 1.56 | 0,0048 |
| A\_51\_P106373 | NM\_025321 | Mus musculus RIKEN cDNA 0610010E03 gene (0610010E03Rik), mRNA | 1.56 | 0,0108 |
| A\_51\_P408059 | NM\_026532 | Mus musculus nuclear transport factor 2 (Nutf2), mRNA | 1.56 | 0,0097 |
| A\_51\_P391074 | NM\_007386 | M.musculus mRNA for iron responsive element binding protein | 1.56 | 0,0336 |
| A\_51\_P174158 | NM\_010571 | Mus musculus insulin receptor substrate-3 (IRS-3) mRNA, partial cds | 1.56 | 0,0073 |
| A\_51\_P510418 | NM\_028270 | Mus musculus, aldehyde dehydrogenase 1 family, member B1, clone MGC:28561 IMAGE:4207479, mRNA, complete cds | 1.56 | 0,0077 |
| A\_51\_P214916 | BU056272 | Mus musculus NADH dehydrogenase Fe-S protein 5 (Ndufs5), mRNA | 1.56 | 0,0142 |
| A\_51\_P413583 | NM\_183176 | hypothetical protein | 1.56 | 0,0496 |
| A\_51\_P473089 | NM\_178647 | P20-CGGBP homolog [Homo sapiens] | 1.56 | 0,0337 |
| A\_51\_P442402 | NM\_029657 | hypothetical Crystallin/RING finger containing protein | 1.55 | 0,0063 |
| A\_51\_P222033 | NM\_178070 | VACUOLAR PROTEIN SORTING HOMOLOG R-VPS33B homolog [Rattus norvegicus] | 1.55 | 0,0050 |
| A\_51\_P313703 | NM\_008639 | Mus musculus melatonin receptor 1A (Mtnr1a), mRNA | 1.55 | 0,0043 |
| A\_51\_P448784 | NM\_009513 | Mus musculus vesicular membrain protein p24 (Vmp), mRNA | 1.55 | 0,0268 |
| A\_51\_P371882 | NM\_133744 | Mus musculus RIKEN cDNA 2600016J21 gene (2600016J21Rik), mRNA | 1.55 | 0,0093 |
| A\_51\_P352114 | AK029175 | unclassifiable | 1.55 | 0,0233 |
| A\_51\_P255193 | NM\_178397 | inferred: RIKEN cDNA 2210404D11 gene / Unknown (protein for IMAGE:2961467) {Homo sapiens} | 1.55 | 0,0256 |
| A\_51\_P120398 | NM\_025758 | Mus musculus RIKEN cDNA 4933436O18 gene (4933436O18Rik), mRNA | 1.55 | 0,0039 |
| A\_51\_P344051 | NM\_134029 | Mus musculus 5,3-nucleotidase, mitochondrial (Nt5m), mRNA | 1.55 | 0,0088 |
| A\_51\_P464398 | AK046394 | SERINE/THREONINE KINASE NKIATRE ALPHA homolog [Rattus norvegicus] | 1.55 | 0,0188 |
| A\_51\_P173459 | NM\_175538 | hypothetical Eukaryotic protein kinase containing protein | 1.55 | 0,0294 |
| A\_51\_P158007 | NM\_133797 | Mus musculus expressed sequence C81457 (C81457), mRNA | 1.55 | 0,0060 |
| A\_51\_P248705 | NM\_023565 | Mus musculus chromosome segregation 1-like (S. cerevisiae) (Cse1l), mRNA | 1.55 | 0,0164 |
| A\_51\_P392636 | NM\_009420 | Mus musculus testis specific gene 1 (Tpx1), mRNA | 1.55 | 0,0082 |
| A\_51\_P143082 | NM\_026394 | Mus musculus RIKEN cDNA 1110055J05 gene (1110055J05Rik), mRNA | 1.55 | 0,0145 |
| A\_51\_P101582 | NM\_018875 | Mus musculus sorting nexin 12 (Snx12), mRNA | 1.55 | 0,0028 |
| A\_51\_P301394 | NM\_008179 | Mus musculus G1 to phase transition 2 (Gspt2), mRNA | 1.55 | 0,0314 |
| A\_51\_P251402 | NM\_029578 | similar to DTDP-D-GLUCOSE 4,6-DEHYDRATASE (EC 4.2.1.46) [Homo sapiens] | 1.55 | 0,0055 |
| A\_51\_P220893 | NM\_026879 | hypothetical Eukaryotic protein of unknown function, DUF279 containing protein | 1.55 | 0,0124 |
| A\_51\_P278103 | NM\_024239 | associated molecule with the SH3 domain of STAM | 1.55 | 0,0198 |
| A\_51\_P518081 | AK034331 | unknown EST | 1.55 | 0,0019 |
| A\_51\_P473240 | NM\_146221 | ZFP61P (FRAGMENT) homolog [Mus musculus] | 1.55 | 0,0160 |
| A\_51\_P438527 | NM\_028057 | NADH CYTOCHROME B5 REDUCTASE | 1.55 | 0,0175 |
| A\_51\_P140887 | NM\_016889 | Mus musculus insulinoma-associated 1 (Insm1), mRNA | 1.55 | 0,0434 |
| A\_51\_P385178 | AK029507 | hypothetical ARM repeat structure containing protein | 1.55 | 0,0004 |
| A\_51\_P155142 | NM\_026560 | similar to HYPOTHETICAL 31.3 KDA PROTEIN [Homo sapiens] | 1.55 | 0,0004 |
| A\_51\_P108850 | NM\_146106 | Mus musculus, clone MGC:28394 IMAGE:4023059, mRNA, complete cds | 1.55 | 0,0139 |
| A\_51\_P435339 | NM\_010149 | Mus musculus erythropoietin receptor (Epor), mRNA | 1.55 | 0,0059 |
| A\_51\_P223887 | AK011097 | 4930506L13RIK PROTEIN homolog [Mus musculus] | 1.55 | 0,0099 |
| A\_51\_P457492 | NM\_025791 | Mus musculus RIKEN cDNA 0610006I08 gene (0610006I08Rik), mRNA | 1.54 | 0,0438 |
| A\_51\_P407480 | NM\_008665 | Mus musculus myelin transcription factor 1 (Myt1), mRNA | 1.54 | 0,0168 |
| A\_51\_P160662 | NM\_025379 | Mus musculus cytochrome c oxidase subunit VIIb (Cox7b), mRNA | 1.54 | 0,0132 |
| A\_51\_P278840 | NM\_177321 | Mus musculus melanoma inhibitory activity protein 2 mRNA, complete cds | 1.54 | 0,0267 |
| A\_51\_P472329 | NM\_028883 | weakly similar to KIAA1609 PROTEIN (FRAGMENT) [Homo sapiens] | 1.54 | 0,0084 |
| A\_51\_P119633 | NM\_008043 | Mus musculus frequently rearranged in advanced T-cell lymphomas (Frat1), mRNA | 1.54 | 0,0155 |
| A\_51\_P391727 | NM\_009205 | Mus musculus solute carrier family 3, member 1 (Slc3a1), mRNA | 1.54 | 0,0035 |
| A\_51\_P325343 | NM\_011951 | Mus musculus mitogen activated protein kinase 14 (Mapk14), mRNA | 1.54 | 0,0108 |
| A\_51\_P331363 | NM\_030184 | hypothetical ARM repeat structure containing protein | 1.54 | 0,0476 |
| A\_51\_P475502 | BC012416 | Mus musculus, RIKEN cDNA 0610033L03 gene, clone MGC:7941 IMAGE:3584066, mRNA, complete cds | 1.54 | 0,0279 |
| A\_51\_P359454 | NM\_030561 | Mus musculus hypothetical protein, MGC:7550 (BC004004), mRNA | 1.54 | 0,0070 |
| A\_51\_P398848 | NM\_197987 | TRAF encompassing factor 3 | 1.54 | 0,0128 |
| A\_51\_P145360 | NM\_177879 | hypothetical Fibronectin type III domain containing protein | 1.54 | 0,0174 |
| A\_51\_P423398 | A\_51\_P423398 | Mus musculus cDNA, 5 end | 1.54 | 0,0016 |
| A\_51\_P277445 | NM\_013923 | Mus musculus ring finger protein (C3HC4 type) 19 (Rnf19), mRNA | 1.54 | 0,0114 |
| A\_51\_P413461 | NM\_011960 | Mus musculus poly (ADP-ribose) glycohydrolase (Parg), mRNA | 1.54 | 0,0463 |
| A\_51\_P409468 | XM\_619748 | Mus musculus olfactory receptor MOR128-1 (MOR128-1) pseudogene | 1.53 | 0,0141 |
| A\_51\_P228625 | A\_51\_P228625 | Similar to: Mus musculus perlecan (heparan sulfate proteoglycan 2) (Hspg2), mRNA | 1.53 | 0,0032 |
| A\_51\_P456756 | NM\_145524 | Mus musculus, Similar to hypothetical protein FLJ13984, clone MGC:7995 IMAGE:3585719, mRNA, complete cds | 1.53 | 0,0204 |
| A\_51\_P234174 | NM\_008886 | Mus musculus postmeiotic segregation increased 2 (S. cerevisiae) (Pms2), mRNA | 1.53 | 0,0261 |
| A\_51\_P247542 | NM\_172799 | hypothetical Tubulin-tyrosine ligase containing protein | 1.53 | 0,0027 |
| A\_51\_P206968 | NM\_008596 | Mus musculus mitsugumin 29 (Mg29), mRNA | 1.53 | 0,0269 |
| A\_51\_P258409 | NM\_010423 | Mus musculus hairy/enhancer-of-split related with YRPW motif 1 (Hey1), mRNA | 1.53 | 0,0398 |
| A\_51\_P255757 | NM\_153516 | mitochondria located 1 homolog (human) | 1.53 | 0,0266 |
| A\_51\_P121397 | NM\_025450 | Mus musculus mitochondrial ribosomal protein S17 (Mprs17), mRNA | 1.53 | 0,0178 |
| A\_51\_P176083 | NM\_146187 | Mus musculus, Similar to G protein-coupled receptor 43, clone MGC:28611 IMAGE:4218874, mRNA, complete cds | 1.53 | 0,0433 |
| A\_51\_P176387 | AK077784 | unknown EST | 1.53 | 0,0376 |
| A\_51\_P137375 | NM\_026033 | Mus musculus mRNA for ODAG protein, complete cds | 1.53 | 0,0189 |
| A\_51\_P212184 | NM\_025849 | Mus musculus RIKEN cDNA 3110001D03 gene (3110001D03Rik), mRNA | 1.53 | 0,0139 |
| A\_51\_P360508 | NM\_144801 | similar to HYPOTHETICAL 51.7 KDA PROTEIN [Homo sapiens] | 1.53 | 0,0085 |
| A\_51\_P380986 | NM\_007935 | Mus musculus enhancer of polycomb homolog 1, (Drosophila) (Epc1), mRNA | 1.53 | 0,0222 |
| A\_51\_P207310 | U85089 | Mus musculus thioredoxin 2 (Txn2), mRNA | 1.53 | 0,0016 |
| A\_51\_P154973 | NM\_021547 | Mus musculus steroidogenic acute regulatory protein related (Mln64-pending), mRNA | 1.53 | 0,0319 |
| A\_51\_P210340 | AK011997 | unknown EST | 1.53 | 0,0090 |
| A\_51\_P516078 | NM\_146067 | similar to CDNA FLJ11151 FIS, CLONE PLACE1006883 [Homo sapiens] | 1.53 | 0,0145 |
| A\_51\_P476900 | AK053449 | Mus musculus PR domain containing 15 (Prdm15), mRNA | 1.53 | 0,0051 |
| A\_51\_P290415 | BC046389 | unknown EST | 1.53 | 0,0110 |
| A\_51\_P243604 | NM\_175934 | Mus musculus, Similar to putative protein phosphatase 1 nuclear targeting subunit, clone IMAGE:3157989, mRNA, partial cds | 1.53 | 0,0159 |
| A\_51\_P270324 | NM\_012040 | Mus musculus pregnancy upregulated non-ubiquitously expressed CaM kinase (Pnck), mRNA | 1.53 | 0,0387 |
| A\_51\_P124784 | NM\_153420 | Mus musculus RIKEN cDNA C130099A20 gene (C130099A20Rik), mRNA | 1.53 | 0,0019 |
| A\_51\_P206824 | NM\_027126 | hypothetical protein | 1.53 | 0,0008 |
| A\_51\_P126817 | NM\_020580 | TH1-like homolog (Drosophila) | 1.53 | 0,0069 |
| A\_51\_P264388 | NM\_013931 | Mus musculus mitogen-activated protein kinase 8 interacting protein 3 (Mapk8ip3), mRNA | 1.53 | 0,0434 |
| A\_51\_P451066 | NM\_009722 | Mus musculus ATPase, Ca++ transporting, cardiac muscle, slow twitch 2 (Atp2a2), mRNA | 1.53 | 0,0477 |
| A\_51\_P230850 | NM\_018807 | Mus musculus pleiomorphic adenoma gene-like 2 (Plagl2), mRNA | 1.53 | 0,0048 |
| A\_51\_P482801 | NM\_023906 | Mus musculus ankyrin repeat and SOCS box-containing protein 3 (Asb3), mRNA | 1.53 | 0,0264 |
| A\_51\_P445392 | NM\_175127 | similar to F-BOX PROTEIN 28 (FRAGMENT) [Xenopus laevis] | 1.53 | 0,0398 |
| A\_51\_P215955 | NM\_025944 | hypothetical protein | 1.53 | 0,0020 |
| A\_51\_P453541 | NM\_013908 | Mus musculus f-box and WD-40 domain protein 5 (Fbxw5), mRNA | 1.53 | 0,0127 |
| A\_51\_P110671 | NM\_009074 | Mus musculus macrophage stimulating 1 receptor (c-met-related tyrosine kinase) (Mst1r), mRNA | 1.53 | 0,0054 |
| A\_51\_P483659 | NM\_018880 | Mus musculus, Similar to ring finger protein 22, clone IMAGE:3481755, mRNA | 1.53 | 0,0109 |
| A\_51\_P338040 | NM\_146168 | Mus musculus, Similar to hypothetical protein DKFZp564K0822, clone MGC:38780 IMAGE:5359195, mRNA, complete cds | 1.52 | 0,0348 |
| A\_51\_P506148 | NM\_178911 | hypothetical protein | 1.52 | 0,0042 |
| A\_51\_P371255 | NM\_016924 | ORF5 PROTEIN homolog [Mus musculus] | 1.52 | 0,0006 |
| A\_51\_P344346 | XM\_144699 | hypothetical Cysteine-rich region containing protein | 1.52 | 0,0053 |
| A\_51\_P193130 | NM\_011288 | Mus musculus mitochondrial ribosomal protein L23 (Mrpl23), mRNA | 1.52 | 0,0195 |
| A\_51\_P139711 | NM\_178908 | Mus musculus, similar to unnamed protein product, clone MGC:32360 IMAGE:5031155, mRNA, complete cds | 1.52 | 0,0457 |
| A\_51\_P256623 | NM\_013629 | Mus musculus putative homeodomain transcription factor (Phtf), mRNA | 1.52 | 0,0494 |
| A\_51\_P216905 | NM\_007438 | Mus musculus aldolase 1, A isoform (Aldo1), mRNA | 1.52 | 0,0149 |
| A\_51\_P316833 | NM\_178367 | PRE MRNA SPLICING FACTOR RNA HELICASE DEAH BOX PROTEIN | 1.52 | 0,0131 |
| A\_51\_P439210 | NM\_172805 | EAG K+ CHANNEL, ISOFORM 2 homolog [Rattus norvegicus] (Kcnh5) | 1.52 | 0,0376 |
| A\_51\_P176912 | NM\_139145 | Mus musculus Hlcs mRNA, mouse homolog of Human holocarboxylase synthtase gene HLCS, complete cds | 1.52 | 0,0096 |
| A\_51\_P270650 | NM\_207558 | Mus musculus olfactory receptor GA\_x5J8B7W5WBF-6267395-6266441 (GA\_x5J8B7W5WBF-6267395-6266441) pseudogene | 1.52 | 0,0081 |
| A\_51\_P260886 | NM\_025895 | Mus musculus endothelial-derived gene (Eg1-pending), mRNA | 1.52 | 0,0104 |
| A\_51\_P241213 | AK020696 | Mus musculus RIKEN cDNA A030005L19 gene (A030005L19Rik), mRNA | 1.52 | 0,0038 |
| A\_51\_P424999 | NM\_026729 | Mus musculus immature colon carcinoma transcript 1 (Ict1), mRNA | 1.52 | 0,0229 |
| A\_51\_P243168 | XM\_355201 | NIH\_BMAP\_FR0 Mus musculus cDNA clone IMAGE: 6413555 5, mRNA sequence | 1.52 | 0,0196 |
| A\_51\_P479832 | AK016708 | DNA-binding protein (fragment) homolog [Homo sapiens] | 1.52 | 0,0037 |
| A\_51\_P346884 | NM\_025665 | Mus musculus, Similar to hypothetical gene LOC134550, clone MGC:41429 IMAGE:3468252, mRNA, complete cds | 1.52 | 0,0293 |
| A\_51\_P313337 | AK077261 | unknown EST | 1.52 | 0,0093 |
| A\_51\_P367578 | AK079162 | FETAL ALZHEIMER ANTIGEN FETAL ALZ 50 REACTIVE CLONE | 1.52 | 0,0156 |
| A\_51\_P364671 | NM\_029272 | similar to NADH-UBIQUINONE OXIDOREDUCTASE 20 KDA SUBUNIT, MITOCHONDRIAL PRECURSOR (EC 1.6.5.3) (EC 1.6.99.3) (COMPLEX I-20KD) (CI-20KD) (PSST SUBUNIT) [Homo sapiens] | 1.52 | 0,0287 |
| A\_51\_P206971 | NM\_008612 | menage a trois 1 | 1.52 | 0,0345 |
| A\_51\_P289239 | NM\_007977 | Mus musculus coagulation factor VIII (F8), mRNA | 1.52 | 0,0458 |
| A\_51\_P416046 | BC020156 | Mus musculus, Similar to hypothetical gene MGC1127, clone MGC:28380 IMAGE:4021570, mRNA, complete cds | 1.52 | 0,0090 |
| A\_51\_P169128 | NM\_025879 | Mus musculus RIKEN cDNA 2410002O22 gene (2410002O22Rik), mRNA | 1.52 | 0,0229 |
| A\_51\_P364188 | BC027085 | unknown | 1.52 | 0,0099 |
| A\_51\_P360531 | NM\_138662 | Mus musculus protocadherin alpha 3 (Pcdha3), mRNA | 1.52 | 0,0284 |
| A\_51\_P214755 | NM\_175201 | HYPOTHETICAL 43.9 KDA PROTEIN homolog [Homo sapiens] | 1.52 | 0,0086 |
| A\_51\_P149502 | NM\_145217 | BC41195\_1 (RIG PROTEIN) (SMALL GTP-BINDING TUMOR SUPPRESSOR 1) homolog [Homo sapiens] | 1.52 | 0,0316 |
| A\_51\_P459787 | NM\_177784 | hypothetical BTB/POZ domain/Kelch repeat containing protein | 1.52 | 0,0254 |
| A\_51\_P228149 | NM\_009633 | Mus musculus adrenergic receptor, alpha 2b (Adra2b), mRNA | 1.52 | 0,0176 |
| A\_51\_P385390 | NM\_022418 | Mus musculus RIKEN cDNA 2010004O20 gene (2010004O20Rik), mRNA | 1.52 | 0,0406 |
| A\_51\_P184969 | NM\_021430 | Mus musculus RIKEN cDNA 2900002H16 gene (2900002H16Rik), mRNA | 1.52 | 0,0009 |
| A\_51\_P295701 | NM\_026721 | hypothetical ZIP Zinc transporter containing protein | 1.52 | 0,0297 |
| A\_51\_P482628 | NM\_019924 | Mus musculus ribosomal protein S6 kinase, 90kD, polypeptide 4 (Rps6ka4), mRNA | 1.52 | 0,0366 |
| A\_51\_P469221 | NM\_178624 | LEUCINE-RICH REPEATS CONTAINING F-BOX PROTEIN FBL3 homolog [Homo sapiens] | 1.51 | 0,0279 |
| A\_51\_P355622 | NM\_008087 | Mus musculus growth arrest specific 2 (Gas2), mRNA | 1.51 | 0,0499 |
| A\_51\_P431649 | NM\_133224 | Mus musculus cation-transporting atpase (catp), mRNA | 1.51 | 0,0080 |
| A\_51\_P488799 | NM\_173761 | DJ963E22.1 (NOVEL PROTEIN SIMILAR TO NY-REN-2 ANTIGEN) (FRAGMENT) homolog [Homo sapiens] | 1.51 | 0,0081 |
| A\_51\_P360840 | NM\_009569 | Mus musculus zinc finger protein, multitype 1 (Zfpm1), mRNA | 1.51 | 0,0377 |
| A\_51\_P225852 | NM\_008786 | Mus musculus protein-L-isoaspartate (D-aspartate) O-methyltransferase 1 (Pcmt1), mRNA | 1.51 | 0,0190 |
| A\_51\_P341379 | NM\_172678 | Y79AA1001048 PROTEIN (HYPOTHETICAL 68.8 KDA PROTEIN) (UNKNOWN) (PROTEIN FOR MGC:14452) homolog [Homo sapiens] | 1.51 | 0,0110 |
| A\_51\_P440267 | AI851444 | Mus musculus cDNA, 3 end | 1.51 | 0,0234 |
| A\_51\_P333839 | NM\_019971 | Mus musculus platelet-derived growth factor, C polypeptide (Pdgfc), mRNA | 1.51 | 0,0193 |
| A\_51\_P286410 | NM\_026872 | Mus musculus, Similar to KIAA0144 gene product, clone MGC:7679 IMAGE:3496608, mRNA, complete cds | 1.51 | 0,0248 |
| A\_51\_P191601 | NM\_029841 | hypothetical protein | 1.51 | 0,0459 |
| A\_51\_P172532 | NM\_012049 | Mus musculus nitrilase 1 (Nit1), mRNA | 1.51 | 0,0070 |
| A\_51\_P345186 | NM\_018877 | Mus musculus SET domain, bifurcated 1 (Setdb1), mRNA | 1.51 | 0,0276 |
| A\_51\_P298230 | XM\_488586 | unclassifiable | 1.51 | 0,0130 |
| A\_51\_P354642 | AK004981 | Mus musculus RIKEN cDNA 1300013B24 gene (1300013B24Rik), mRNA | 1.51 | 0,0241 |
| A\_51\_P115891 | NM\_026655 | hypothetical protein | 1.51 | 0,0033 |
| A\_51\_P496535 | XM\_132976 | Mus musculus, Similar to hypothetical protein FLJ22569, clone MGC:41153 IMAGE:1434214, mRNA, complete cds | 1.51 | 0,0279 |
| A\_51\_P160223 | NAP057140-1 | Mus musculus olfactory receptor GA\_x5J8B7W3SPQ-59383-59030 (GA\_x5J8B7W3SPQ-59383-59030) pseudogene | 1.51 | 0,0036 |
| A\_51\_P352565 | NM\_024177 | Mus musculus mitochondrial ribosomal protein L38 (Mrpl38), mRNA | 1.51 | 0,0070 |
| A\_51\_P154933 | NM\_133967 | Mus musculus zinc finger, DHHC domain containing 7 (Zdhhc7), mRNA | 1.51 | 0,0335 |
| A\_51\_P453088 | NM\_174987 | hypothetical Cytochrome c oxidase subunit h structure containing protein | 1.51 | 0,0370 |
| A\_51\_P474902 | AK049089 | similar to ACTIVIN-LIKE RECEPTOR KINASE-7 [Rattus norvegicus] | 1.51 | 0,0074 |
| A\_51\_P224023 | NM\_144846 | Mus musculus RIKEN cDNA 0910001A06 gene (0910001A06Rik), mRNA | 1.51 | 0,0386 |
| A\_51\_P413803 | AK021319 | hypothetical protein | 1.51 | 0,0004 |
| A\_51\_P408644 | NM\_013719 | Mus musculus eukaryotic translation initiation factor 2 alpha kinase 4 (Eif2ak4), mRNA | 1.51 | 0,0014 |
| A\_51\_P472488 | NM\_019836 | Mus musculus RIKEN cDNA 2610024G14 gene (2610024G14Rik), mRNA | 1.51 | 0,0041 |
| A\_51\_P354792 | NM\_008521 | Mus musculus leukotriene C4 synthase (Ltc4s), mRNA | 1.51 | 0,0051 |
| A\_51\_P147654 | AK013971 | hypothetical protein | 1.51 | 0,0415 |
| A\_51\_P228276 | NM\_008095 | Mus musculus glioblastoma amplified sequence (Gbas), mRNA | 1.51 | 0,0210 |
| A\_51\_P248243 | NM\_010903 | Mus musculus nuclear factor, erythroid derived 2, like 3 (Nfe2l3), mRNA | 1.51 | 0,0275 |
| A\_51\_P155747 | NM\_028776 | hypothetical ARM repeat structure containing protein | 1.51 | 0,0099 |
| A\_51\_P274667 | XM\_129972 | hypothetical Zn-finger CCHC type containing protein | 1.51 | 0,0132 |
| A\_51\_P385718 | NM\_026862 | hypothetical Prenyl group binding site (CAAX box) containing protein | 1.51 | 0,0129 |
| A\_51\_P249749 | NM\_031402 | Mus musculus Cocoacrisp (Cocoacrisp-pending), mRNA | 1.51 | 0,0445 |
| A\_51\_P483089 | NM\_010411 | Mus musculus histone deacetylase 3 (Hdac3), mRNA | 1.49 | 0,0065 |
| A\_51\_P395727 | NM\_019575 | Mus musculus secretory carrier membrane protein 4 (Scamp4), mRNA | 1.49 | 0,0044 |
| A\_51\_P501396 | NM\_021450 | Mus musculus transient receptor potential cation channel, subfamily M, member 7 (Trpm7), mRNA | 1.49 | 0,0381 |
| A\_51\_P181676 | NM\_008398 | Mus musculus integrin alpha 7 (Itga7), mRNA | 1.49 | 0,0471 |
| A\_51\_P486488 | AK040705 | unclassifiable | 1.49 | 0,0317 |
| A\_51\_P281123 | NM\_173738 | Mus musculus similar to Hypothetical zinc finger protein KIAA1559 (LOC233057), mRNA | 1.49 | 0,0383 |
| A\_51\_P424412 | NM\_011540 | Mus musculus titin-cap (Tcap), mRNA | 1.49 | 0,0045 |
| A\_51\_P176854 | NM\_027175 | Mus musculus, RIKEN cDNA 2410001M24 gene, clone MGC:27963 IMAGE:3593650, mRNA, complete cds | 1.49 | 0,0296 |
| A\_51\_P163378 | NM\_025635 | Mus musculus, RIKEN cDNA 2600001N01 gene, clone MGC:38778 IMAGE:5359154, mRNA, complete cds | 1.49 | 0,0170 |
| A\_51\_P195135 | NM\_011462 | Mus musculus spindlin (Spin), mRNA | 1.49 | 0,0193 |
| A\_51\_P414160 | NM\_027935 | SMALL MEMBRANE PROTEIN 1 homolog [Homo sapiens] | 1.49 | 0,0029 |
| A\_51\_P265685 | NM\_026673 | hypothetical protein | 1.49 | 0,0047 |
| A\_51\_P470443 | NM\_145522 | SIMILAR TO RAB9 EFFECTOR P40 homolog [Mus musculus] | 1.49 | 0,0131 |
| A\_51\_P425737 | NM\_025935 | hypothetical Ypt/Rab-GAP domain of gyp1p structure containing protein | 1.49 | 0,0260 |
| A\_51\_P448677 | NM\_008790 | Mus musculus Purkinje cell protein 2 (L7) (Pcp2), mRNA | 1.49 | 0,0065 |
| A\_51\_P241577 | NM\_027649 | hypothetical Serine-rich region containing protein | 1.49 | 0,0099 |
| A\_51\_P513211 | NM\_138660 | Mus musculus MLN51 protein (MLN51), mRNA | 1.49 | 0,0048 |
| A\_51\_P190124 | NM\_027347 | Mus musculus, RIKEN cDNA 3000002A17 gene, clone MGC:7510 IMAGE:3491622, mRNA, complete cds | 1.49 | 0,0108 |
| A\_51\_P393064 | NM\_021392 | adaptor-related protein complex AP-4, mu 1 | 1.49 | 0,0189 |
| A\_51\_P215066 | NM\_026519 | Mus musculus RIKEN cDNA 2610318K02 gene (2610318K02Rik), mRNA | 1.49 | 0,0014 |
| A\_51\_P204402 | NM\_011369 | Mus musculus Shc SH2-domain binding protein 1 (Shcbp1), mRNA | 1.49 | 0,0065 |
| A\_51\_P493940 | AK006268 | hypothetical protein | 1.49 | 0,0013 |
| A\_51\_P242733 | NM\_009357 | Mus musculus testis expressed gene 261 (Tex261), mRNA | 1.49 | 0,0440 |
| A\_51\_P500135 | NM\_145602 | Mus musculus, Similar to NDRG family, member 4, clone MGC:7067 IMAGE:3156802, mRNA, complete cds | 1.49 | 0,0460 |
| A\_51\_P509211 | NM\_030564 | Mus musculus ring finger protein 34 (Rnf34), mRNA | 1.49 | 0,0083 |
| A\_51\_P504053 | AK076973 | DNA segment, Chr 1, ERATO Doi 57, expressed | 1.48 | 0,0090 |
| A\_51\_P487263 | NM\_173038 | hypothetical RNI-like structure containing protein | 1.48 | 0,0291 |
| A\_51\_P309293 | AK129163 | Mus musculus, clone IMAGE:1378878, mRNA | 1.48 | 0,0309 |
| A\_51\_P275658 | NM\_016874 | Mus musculus deformed epidermal autoregulatory factor 1 (Drosophila) (Deaf1), mRNA | 1.48 | 0,0051 |
| A\_51\_P171553 | NM\_145412 | Mus musculus RIKEN cDNA 5830457O10 gene (5830457O10Rik), mRNA | 1.48 | 0,0288 |
| A\_51\_P118284 | NM\_013723 | Mus musculus podocalyxin-like (Podxl), mRNA | 1.48 | 0,0019 |
| A\_51\_P141071 | NM\_025958 | TIP120-FAMILY PROTEIN TIP120B, SHORT FORM homolog [Rattus norvegicus] | 1.48 | 0,0070 |
| A\_51\_P289862 | BC052065 | RAP1 GTPASE ACTIVATING PROTEIN 1 (RAP1GAP) homolog [Homo sapiens] | 1.48 | 0,0090 |
| A\_51\_P250207 | NAP057197-1 | Mus musculus olfactory receptor MOR239-8P (MOR239-8P) pseudogene | 1.48 | 0,0050 |
| A\_51\_P289872 | NM\_172585 | weakly similar to C-MPL BINDING PROTEIN [Homo sapiens] | 1.48 | 0,0066 |
| A\_51\_P310076 | NM\_010945 | Mus musculus neutral sphingomyelinase (N-SMase) activation associated factor (Nsmaf), mRNA | 1.48 | 0,0395 |
| A\_51\_P103650 | NM\_053100 | Mus musculus tripartite motif protein 8 (Trim8), mRNA | 1.48 | 0,0457 |
| A\_51\_P264527 | NM\_019833 | Mus musculus hypothetical protein, clone 1-82 (AB030186), mRNA | 1.48 | 0,0025 |
| A\_51\_P319592 | AK014101 | UBIQUITIN CARBOXYL TERMINAL HYDROLASE DUB 1 EC 3.1.2.15 UBIQUITIN THIOLESTERASE DUB 1 UBIQUITIN SPECIFIC PROCESSING PROTEASE DUB 1 DEUBIQUITINATING ENZYME | 1.48 | 0,0364 |
| A\_51\_P429046 | NM\_152810 | cell division cycle 5-like (S. pombe) | 1.48 | 0,0018 |
| A\_51\_P380772 | NM\_178224 | Mus musculus cystathionine beta-synthase mRNA, 5 untranslated region | 1.48 | 0,0156 |
| A\_51\_P442990 | NM\_028233 | similar to leucine-rich protein [Homo sapiens] | 1.48 | 0,0081 |
| A\_51\_P387006 | AI587890 | unknown EST | 1.48 | 0,0117 |
| A\_51\_P204366 | NM\_001001983 | Mus musculus, Similar to phosphatidylinositol 4-kinase, clone IMAGE:5363136, mRNA | 1.48 | 0,0286 |
| A\_51\_P102782 | NM\_199195 | Mus musculus branched-chain alpha-ketoacid dehydrogenase E1 beta-subunit mRNA sequence | 1.48 | 0,0039 |
| A\_51\_P263503 | NM\_011949 | Mus musculus mitogen activated protein kinase 1 (Mapk1), mRNA | 1.48 | 0,0492 |
| A\_51\_P215077 | NM\_025569 | Mus musculus microsomal glutathione S-transferase 3 (Mgst3), mRNA | 1.48 | 0,0106 |
| A\_51\_P158305 | M29242 | Mouse U5 small nuclear RNA, clone 2 | 1.48 | 0,0022 |
| A\_51\_P326932 | AK018172 | unknown EST | 1.48 | 0,0368 |
| A\_51\_P234233 | NM\_001009545 | similar to TMDC IV PROTEIN [Rattus norvegicus] | 1.48 | 0,0037 |
| A\_51\_P306867 | NM\_026529 | Mus musculus RIKEN cDNA 2700062C07 gene (2700062C07Rik), mRNA | 1.48 | 0,0330 |
| A\_51\_P487734 | NM\_027088 | Mus musculus uch-x4 mRNA for ubiquitin C-terminal hydrolase X4, complete cds | 1.48 | 0,0420 |
| A\_51\_P194999 | NM\_027931 | similar to SIMILAR TO THREONYL-TRNA SYNTHETASE (HYPOTHETICAL 81.0 KDA PROTEIN) [Homo sapiens] | 1.48 | 0,0141 |
| A\_51\_P511707 | NM\_025636 | Mus musculus RIKEN cDNA 2310079N02 gene (2310079N02Rik), mRNA | 1.48 | 0,0020 |
| A\_51\_P478279 | NM\_053170 | Mus musculus tripartite motif protein TRIM33 (Trim33) mRNA, partial cds | 1.47 | 0,0020 |
| A\_51\_P119841 | NM\_010491 | Mus musculus islet amyloid polypeptide (Iapp), mRNA | 1.47 | 0,0046 |
| A\_51\_P399816 | AK039074 | unknown EST | 1.47 | 0,0072 |
| A\_51\_P105008 | NM\_027853 | Mus musculus RIKEN cDNA 0610006F02 gene (0610006F02Rik), mRNA | 1.47 | 0,0220 |
| A\_51\_P421477 | AK173135 | hypothetical S6 modification enzyme RimK family protein containing protein | 1.47 | 0,0397 |
| A\_51\_P469252 | NM\_172817 | ZINC FINGER PROTEIN CLONE 647 (FRAGMENT) homolog [Homo sapiens] | 1.47 | 0,0271 |
| A\_51\_P277781 | NM\_013497 | Mus musculus, Similar to cAMresponsive element binding protein 3, clone MGC:6348 IMAGE:3490360, mRNA, complete cds | 1.47 | 0,0043 |
| A\_51\_P107612 | AK008214 | BITHORAXOID-LIKE PROTEIN (2010320M17RIK PROTEIN) (DYNEIN-ASSOCIATED PROTEIN RKM23) homolog [Mus musculus] | 1.47 | 0,0149 |
| A\_51\_P254553 | NM\_025947 | "similar to TR:O88567 O88567 BITHORAXOID-LIKE PROTEIN. � | 1.47 | 0,0125 |
| A\_51\_P180855 | NAP057133-1 | Mus musculus olfactory receptor GA\_x5J8B7W3Y5M-51270-50422 (GA\_x5J8B7W3Y5M-51270-50422) pseudogene | 1.47 | 0,0008 |
| A\_51\_P116581 | NM\_138603 | Mus musculus DNA segment, Chr X, Immunex 40, expressed (DXImx40e), mRNA | 1.47 | 0,0199 |
| A\_51\_P371521 | NM\_172562 | TRANSCRIPTIONAL ADAPTER 2 LIKE ADA2 LIKE PROTEIN | 1.47 | 0,0018 |
| A\_51\_P457054 | NM\_172990 | FANG1 homolog [Rattus norvegicus] | 1.47 | 0,0102 |
| A\_51\_P514131 | M35436 | Mouse T-cell receptor delta-chain mRNA, D2J1-region | 1.47 | 0,0103 |
| A\_51\_P423547 | NM\_029354 | hypothetical protein | 1.47 | 0,0450 |
| A\_51\_P117604 | NM\_025887 | Mus musculus RAB5A, member RAS oncogene family (Rab5a), mRNA | 1.47 | 0,0090 |
| A\_51\_P135939 | NM\_026047 | similar to CDNA FLJ13449 FIS, CLONE PLACE1003025 [Homo sapiens] | 1.47 | 0,0141 |
| A\_51\_P347177 | NM\_026386 | sorting nexin 2 | 1.47 | 0,0074 |
| A\_51\_P291819 | NM\_024210 | Mus musculus RIKEN cDNA 2310033P09 gene (2310033P09Rik), mRNA | 1.47 | 0,0375 |
| A\_51\_P211822 | NM\_001013414 | Mus musculus, Similar to G protein pathway suppressor 2, clone IMAGE:5358633, mRNA, partial cds | 1.47 | 0,0481 |
| A\_51\_P413787 | NM\_147778 | Bmi1 upstream gene | 1.47 | 0,0141 |
| A\_51\_P492807 | AK008233 | weakly similar to 33 KDA VAMP-ASSOCIATED PROTEIN [Homo sapiens] | 1.47 | 0,0026 |
| A\_51\_P393193 | NM\_144511 | Mus musculus esterase 31 (Es31), mRNA | 1.47 | 0,0011 |
| A\_51\_P122740 | NM\_028123 | Mus musculus, clone MGC:11925 IMAGE:3599603, mRNA, complete cds | 1.47 | 0,0430 |
| A\_51\_P340336 | NM\_021568 | Mus musculus poly(rC) binding protein 3 (Pcbp3), mRNA | 1.47 | 0,0279 |
| A\_51\_P450536 | NM\_028170 | Mus musculus, clone MGC:8256 IMAGE:3592293, mRNA, complete cds | 1.47 | 0,0330 |
| A\_51\_P145376 | NM\_001025613 | unknown EST | 1.47 | 0,0212 |
| A\_51\_P147791 | AK085257 | RU2S (FRAGMENT) homolog [Homo sapiens] | 1.47 | 0,0023 |
| A\_51\_P356580 | NM\_026280 | hypothetical protein | 1.47 | 0,0336 |
| A\_51\_P286878 | AK016577 | hypothetical Glutathione synthetase ATP-binding domain-like structure containing protein | 1.47 | 0,0139 |
| A\_51\_P255360 | NM\_026004 | Mus musculus RIKEN cDNA 2610206B05 gene (2610206B05Rik), mRNA | 1.47 | 0,0007 |
| A\_51\_P142760 | NM\_029102 | GLYCOSYLTRANSFERASE | 1.47 | 0,0115 |
| A\_51\_P354681 | NM\_026243 | Mus musculus RIKEN cDNA 9130411I17 gene (9130411I17Rik), mRNA | 1.47 | 0,0260 |
| A\_51\_P400585 | NM\_007466 | Mus musculus apoptosis inhibitory protein 5 (Api5), mRNA | 1.47 | 0,0167 |
| A\_51\_P223177 | NM\_028791 | Mus musculus, RIKEN cDNA 1300018I05 gene, clone MGC:30380 IMAGE:3668655, mRNA, complete cds | 1.47 | 0,0111 |
| A\_51\_P463816 | AK036188 | hypothetical AMP-dependent synthetase and ligase containing protein | 1.47 | 0,0274 |
| A\_51\_P354272 | BC072573 | weakly similar to PUTATIVE EUKARYOTIC TRANSLATION INITIATION FACTOR 3 SUBUNIT (EIF-3) (FRAGMENT) [Homo sapiens] | 1.47 | 0,0016 |
| A\_51\_P247249 | NM\_009662 | arachidonate 5-lipoxygenase | 1.47 | 0,0214 |
| A\_51\_P168945 | NM\_016985 | Mus musculus myotubularin related protein 1 (Mtmr1), mRNA | 1.47 | 0,0266 |
| A\_51\_P233947 | NM\_144522 | weakly similar to EPI64 [Homo sapiens] | 1.47 | 0,0101 |
| A\_51\_P178575 | AF269193 | Mus musculus bromodomain-containing 3 (Brd3), mRNA | 1.47 | 0,0271 |
| A\_51\_P456816 | NM\_172932 | NEUROLIGIN 3 ISOFORM HNL3 homolog [Homo sapiens] | 1.47 | 0,0158 |
| A\_51\_P493175 | NM\_025964 | Mus musculus RIKEN cDNA 2310038H17 gene (2310038H17Rik), mRNA | 1.46 | 0,0075 |
| A\_51\_P216605 | NM\_177993 | HMG-BOX CONTAINING PROTEIN 1 homolog [Rattus norvegicus] | 1.46 | 0,0014 |
| A\_51\_P426214 | NM\_009271 | Mus musculus Rous sarcoma oncogene (Src), mRNA | 1.46 | 0,0165 |
| A\_51\_P369690 | NM\_026257 | Mus musculus socius (Soc-pending), mRNA | 1.46 | 0,0015 |
| A\_51\_P500996 | XM\_126674 | F-BOX PROTEIN FBL2 homolog [Rattus norvegicus] | 1.46 | 0,0268 |
| A\_51\_P123047 | NM\_019710 | Mus musculus SMC (structural maintenance of chromosomes 1)-like 1 (S. cerevisiae) (Smc1l1), mRNA | 1.46 | 0,0100 |
| A\_51\_P417399 | NM\_026468 | ATP synthase, H+ transporting, mitochondrial F0 complex, subunit c (subunit 9), isoform 2 | 1.46 | 0,0082 |
| A\_51\_P448995 | NM\_026494 | Mus musculus RIKEN cDNA 6330579B17 gene (6330579B17Rik), mRNA | 1.46 | 0,0084 |
| A\_51\_P455880 | AK220438 | EXCHANGE FACTOR FOR ARF6 homolog [Rattus norvegicus] | 1.46 | 0,0072 |
| A\_51\_P496462 | NM\_145398 | Mus musculus, Similar to hypothetical protein FLJ21213, clone MGC:6840 IMAGE:2649724, mRNA, complete cds | 1.46 | 0,0394 |
| A\_51\_P354609 | NM\_145152 | similar to LEUCINE-RICH REPEAT PROTEIN LRRC3 PRECURSOR [Homo sapiens] | 1.46 | 0,0326 |
| A\_51\_P334174 | NM\_145383 | Mus musculus, Similar to rhodopsin (opsin 2, rod pigment) (retinitis pigmentosa 4, autosomal dominant), clone MGC:21585 IMAGE:4500760, mRNA, complete cds | 1.46 | 0,0029 |
| A\_51\_P137578 | NM\_175461 | hypothetical protein | 1.46 | 0,0169 |
| A\_51\_P123705 | NM\_144858 | Mus musculus putative zinc finger protein mRNA, complete cds | 1.46 | 0,0045 |
| A\_51\_P216549 | NM\_011641 | Mus musculus transformation related protein 63 (Trp63), mRNA | 1.46 | 0,0020 |
| A\_51\_P174552 | NM\_175004 | similar to HYPOTHETICAL PROTEIN CGI-147 [Homo sapiens] | 1.46 | 0,0250 |
| A\_51\_P111212 | NM\_026269 | hypothetical protein | 1.46 | 0,0134 |
| A\_51\_P378336 | NM\_172687 | HEXAPRENYLDIHYDROXYBENZOATE METHYLTRANSFERASE, MITOCHONDRIAL PRECURSOR EC 2.1.1.114 DIHYDROXYHEXAPRENYLBENZOATE METHYLTRANSFERASE 3,4 DIHYDROXY 5 HEXAPRENYLBENZOATE METHYLTRANSFERASE DHHB METHYLTRANSFERASE DHHB MT DHHB | 1.46 | 0,0355 |
| A\_51\_P413916 | XM\_130859 | MEMBRANE ASSOCIATED PROGESTERONE RECEPTOR COMPONENT 2 (PROGESTERONE MEMBRANE BINDING PROTEIN) (STEROID RECEPTOR PROTEIN DG6) homolog [Homo sapiens] | 1.46 | 0,0099 |
| A\_51\_P413445 | AK016098 | unclassifiable | 1.46 | 0,0053 |
| A\_51\_P207940 | NM\_178789 | hypothetical protein | 1.46 | 0,0189 |
| A\_51\_P175974 | BC038488 | Mus musculus cDNA, 5 end | 1.46 | 0,0381 |
| A\_51\_P106347 | NM\_183146 | similar to SIMILAR TO ZINC FINGER PROTEIN 85 (HPF4, HTF1) [Mus musculus] | 1.46 | 0,0426 |
| A\_51\_P318770 | NM\_153555 | H326 PROTEIN homolog [Homo sapiens] | 1.46 | 0,0052 |
| A\_51\_P338244 | NM\_011591 | Mus musculus similar to Mitochondrial import inner membrane translocase subunit TIM17 B (LOC272751), mRNA | 1.46 | 0,0272 |
| A\_51\_P435239 | NM\_010026 | Mus musculus ADP-ribosylation factor-directed GTPase activating protein isoform a (Shag1) mRNA, complete cds | 1.46 | 0,0170 |
| A\_51\_P316943 | AK012728 | unknown EST | 1.46 | 0,0197 |
| A\_51\_P138952 | NM\_028815 | hypothetical IQ calmodulin-binding motif/Leucine-rich repeat containing protein | 1.46 | 0,0434 |
| A\_51\_P159612 | NM\_019487 | Mus musculus heme binding protein 2 (Hebp2), mRNA | 1.46 | 0,0056 |
| A\_51\_P516456 | NM\_007387 | Mus musculus, Similar to Acid phosphatase 2, lysozymal, clone IMAGE:4218804, mRNA | 1.46 | 0,0040 |
| A\_51\_P461331 | XM\_487374 | Mus musculus T1-cadherin mRNA, partial cds | 1.46 | 0,0376 |
| A\_51\_P128320 | BC030862 | weakly similar to mucin-like peptide MLP 2677 [Rattus norvegicus] | 1.46 | 0,0040 |
| A\_51\_P248387 | NM\_172832 | hypothetical FAD/NAD(P)-binding domain structure containing protein | 1.46 | 0,0026 |
| A\_51\_P428582 | NM\_009647 | Mus musculus adenylate kinase 4 (Ak4), mRNA | 1.46 | 0,0249 |
| A\_51\_P353202 | BC087904 | Mus musculus olfactory receptor MOR261-8P (MOR261-8P) pseudogene | 1.46 | 0,0014 |
| A\_51\_P278489 | NM\_133927 | Mus musculus expressed sequence AI646725 (AI646725), mRNA | 1.46 | 0,0157 |
| A\_51\_P413853 | NM\_172282 | hypothetical Na+/H+ exchanger containing protein | 1.46 | 0,0323 |
| A\_51\_P447600 | AK078630 | Mus musculus cDNA, 5 end | 1.45 | 0,0321 |
| A\_51\_P314323 | NM\_030083 | hypothetical Small nuclear ribonucleoprotein (Sm protein) containing protein | 1.45 | 0,0203 |
| A\_51\_P447287 | NM\_153573 | weakly similar to FK506-BINDING PROTEIN [Homo sapiens] | 1.45 | 0,0447 |
| A\_51\_P457584 | AK090159 | FLAVOHEMOPROTEIN B5/B5RR [Mus musculus] | 1.45 | 0,0125 |
| A\_51\_P484918 | NM\_172551 | DNA DIRECTED RNA POLYMERASE, MITOCHONDRIAL PRECURSOR EC 2.7.7.6 | 1.45 | 0,0028 |
| A\_51\_P196774 | AK045646 | MESENCHYMAL STEM CELL PROTEIN DSC54 homolog [Homo sapiens] | 1.45 | 0,0199 |
| A\_51\_P264554 | NM\_019780 | Mus musculus vacuolar protein sorting 29 (S. pombe) (Vps29), mRNA | 1.45 | 0,0300 |
| A\_51\_P217750 | NM\_019704 | Mus musculus placental protein 6 (Pp6-pending), mRNA | 1.45 | 0,0164 |
| A\_51\_P177062 | AK014419 | hypothetical protein | 1.45 | 0,0117 |
| A\_51\_P381178 | AK038975 | similar to DJ583P15.7.2 (NOVEL ZINC FINGER PROTEIN SIMILAR TO RAT RIN ZF) (FRAGMENT) [Homo sapiens] | 1.45 | 0,0131 |
| A\_51\_P383063 | NM\_147060 | Mus musculus olfactory receptor MOR34-2 (MOR34-2), mRNA | 1.45 | 0,0152 |
| A\_51\_P328652 | NM\_133348 | Mus musculus brain acyl-CoA hydrolase (Bach-pending), mRNA | 1.45 | 0,0390 |
| A\_51\_P337065 | AK086050 | similar to PYRUVATE DEHYDROGENASE PHOSPHATASE REGULATORY SUBUNIT [Bos taurus] | 1.45 | 0,0152 |
| A\_51\_P507970 | NM\_025686 | BRF2, subunit of RNA polymerase III transcription initiation factor, BRF1-like | 1.45 | 0,0359 |
| A\_51\_P152191 | BC092371 | hypothetical protein | 1.45 | 0,0349 |
| A\_51\_P240875 | NM\_207556 | Mus musculus olfactory receptor MOR0-3P (MOR0-3P) pseudogene | 1.45 | 0,0111 |
| A\_51\_P333949 | NM\_178632 | hypothetical ARM repeat structure containing protein | 1.45 | 0,0169 |
| A\_51\_P190254 | AK129084 | unclassifiable | 1.45 | 0,0111 |
| A\_51\_P400236 | NM\_009536 | Mus musculus tyrosine 3-monooxygenase/tryptophan 5-monooxygenase activation protein, epsilon polypeptide (Ywhae), mRNA | 1.45 | 0,0147 |
| A\_51\_P165027 | AK011208 | hypothetical PHD-finger containing protein | 1.45 | 0,0040 |
| A\_51\_P494863 | NM\_178926 | hypothetical protein | 1.45 | 0,0332 |
| A\_51\_P168092 | NM\_024205 | SIMILAR TO HYPOTHETICAL PROTEIN homolog [Mus musculus] | 1.45 | 0,0055 |
| A\_51\_P179251 | NM\_177757 | KINESIN SUPERFAMILY PROTEIN 26B (FRAGMENT) | 1.45 | 0,0092 |
| A\_51\_P242316 | NM\_133818 | Mus musculus expressed sequence AI597479 (AI597479), mRNA | 1.45 | 0,0082 |
| A\_51\_P125679 | AK010725 | hypothetical protein | 1.45 | 0,0040 |
| A\_51\_P216075 | NM\_007838 | Mus musculus dolichyl-di-phosphooligosaccharide-protein glycotransferase (Ddost), mRNA | 1.45 | 0,0222 |
| A\_51\_P480881 | NM\_021513 | Mus musculus hypothetical protein, MNCb-2032 (AB041579), mRNA | 1.45 | 0,0022 |
| A\_51\_P375693 | NM\_028343 | 2810439K08RIK PROTEIN (PUTATIVE TRANSMEMBRANE PROTEIN) homolog [Mus musculus] | 1.45 | 0,0370 |
| A\_51\_P203004 | AY046504 | Mus musculus nuclear pore complex-associated intranuclear protein TPR (tpr) mRNA, partial cds, alternatively spliced | 1.45 | 0,0271 |
| A\_51\_P227392 | NM\_133955 | Mus musculus ras homolog gene family, member U (Arhu), mRNA | 1.45 | 0,0371 |
| A\_51\_P397375 | NM\_144896 | Mus musculus, Similar to PET112 (yeast homolog)-like, clone MGC:11629 IMAGE:3157973, mRNA, complete cds | 1.45 | 0,0084 |
| A\_51\_P220723 | NM\_026592 | Mus musculus RIKEN cDNA B230118H07 gene (B230118H07Rik), mRNA | 1.45 | 0,0167 |
| A\_51\_P176711 | NM\_026165 | Mus musculus RIKEN cDNA 1200007B05 gene (1200007B05Rik), mRNA | 1.45 | 0,0428 |
| A\_51\_P374453 | NM\_145370 | fusca protein homolog Gps1 homolog [Homo sapiens] | 1.45 | 0,0383 |
| A\_51\_P335242 | AK047014 | SIMILAR TO INTEGRIN, BETA-LIKE 1 (WITH EGF-LIKE REPEAT DOMAINS) homolog [Mus musculus] | 1.45 | 0,0252 |
| A\_51\_P414126 | NM\_011226 | RAB19, member RAS oncogene family | 1.45 | 0,0009 |
| A\_51\_P414448 | NM\_053089 | Mus musculus NMDA receptor-regulated gene 1 (Narg1-pending), mRNA | 1.44 | 0,0333 |
| A\_51\_P418374 | NM\_032399 | Mus musculus G protein-coupled receptor 87 (Gpr87), mRNA | 1.44 | 0,0052 |
| A\_51\_P286814 | NM\_011424 | Mus musculus nuclear receptor co-repressor 2 (Ncor2), mRNA | 1.44 | 0,0156 |
| A\_51\_P206563 | AK008751 | CDNA FLJ13589 FIS, CLONE PLACE1009308, WEAKLY SIMILAR TO GLUCOSE REPRESSION MEDIATOR PROTEIN homolog [Homo sapiens] | 1.44 | 0,0039 |
| A\_51\_P310348 | NM\_011912 | Mus musculus ventral anterior homeobox containing gene 2 (Vax2), mRNA | 1.44 | 0,0028 |
| A\_51\_P335000 | NM\_010211 | Mus musculus four and a half LIM domains 1 (Fhl1), mRNA | 1.44 | 0,0272 |
| A\_51\_P355112 | NAP057292-1 | Mus musculus olfactory receptor MOR135-17 (MOR135-17) pseudogene | 1.44 | 0,0209 |
| A\_51\_P516035 | NM\_147041 | Mus musculus olfactory receptor F12 mRNA, partial cds | 1.44 | 0,0175 |
| A\_51\_P467334 | NM\_133728 | hypothetical Protein prenyltransferases alpha subunit repeat containing protein | 1.44 | 0,0150 |
| A\_51\_P292447 | NM\_146219 | Mus musculus, Similar to chromosome 16 open reading frame 44, clone MGC:37805 IMAGE:5098064, mRNA, complete cds | 1.44 | 0,0189 |
| A\_51\_P400335 | NM\_020584 | Mus musculus telomeric repeat binding factor 2, interacting protein (Terf2ip), mRNA | 1.44 | 0,0263 |
| A\_51\_P261483 | NM\_025947 | Mus musculus dynein, cytoplasmic, light chain 2A (Dncl2a), mRNA | 1.44 | 0,0231 |
| A\_51\_P108489 | NM\_008187 | Mus musculus gene trap locus 3 (Gtl3), mRNA | 1.44 | 0,0091 |
| A\_51\_P103509 | NM\_019720 | Mus musculus tumor suppressor region 10 (Tsp10-pending), mRNA | 1.44 | 0,0134 |
| A\_51\_P317029 | NM\_019794 | Mus musculus DnaJ (Hsp40) homolog, subfamily A, member 2 (Dnaja2), mRNA | 1.44 | 0,0085 |
| A\_51\_P300888 | AK006690 | unknown EST | 1.44 | 0,0070 |
| A\_51\_P461123 | AF186107 | Mus musculus toll-like receptor 5 (Tlr5), mRNA | 1.44 | 0,0129 |
| A\_51\_P427762 | NM\_007858 | Mus musculus diaphanous homolog 1 (Drosophila) (Diap1), mRNA | 1.44 | 0,0008 |
| A\_51\_P491976 | NM\_175401 | hypothetical Ribosomal protein S2 containing protein | 1.44 | 0,0354 |
| A\_51\_P380330 | AK033743 | hypothetical Lipocalins structure containing protein | 1.44 | 0,0072 |
| A\_51\_P136025 | NM\_023585 | ubiquitin-conjugating enzyme E2 variant 2 | 1.44 | 0,0453 |
| A\_51\_P326086 | NM\_011293 | Mus musculus RNA polymerase II 4 (14 kDa subunit) (Rpo2-4), mRNA | 1.44 | 0,0170 |
| A\_51\_P493987 | NM\_021509 | Mus musculus RIKEN cDNA 3230402N08 gene (3230402N08Rik), mRNA | 1.44 | 0,0111 |
| A\_51\_P443819 | BC024774 | hypothetical PH domain profile/Microbodies C-terminal targeting signal/Pleckstrin homology (PH) domain containing protein | 1.44 | 0,0324 |
| A\_51\_P281900 | NAP108475-1 | Mus musculus baculoviral IAP repeat-containing 1c, pseudogene 3 (Birc1c-ps3) on chromosome 13 | 1.44 | 0,0276 |
| A\_51\_P151835 | XM\_137316 | unknown EST | 1.44 | 0,0222 |
| A\_51\_P164344 | AK015544 | unknown EST | 1.44 | 0,0471 |
| A\_51\_P472829 | NM\_145144 | Mus musculus, clone MGC:30545 IMAGE:5044495, mRNA, complete cds | 1.44 | 0,0499 |
| A\_51\_P258138 | NM\_025435 | Mus musculus RIKEN cDNA 1500006O09 gene (1500006O09Rik), mRNA | 1.44 | 0,0156 |
| A\_51\_P469568 | NM\_020504 | Mus musculus claudin 13 (Cldn13), mRNA | 1.44 | 0,0032 |
| A\_51\_P200447 | L02241 | Mus musculus protein kinase inhibitor beta, cAMP dependent, testis specific (Pkib), mRNA | 1.44 | 0,0496 |
| A\_51\_P467629 | AK015697 | hypothetical EF-hand structure containing protein | 1.44 | 0,0182 |
| A\_51\_P132258 | AK032636 | MACROH2A2 | 1.44 | 0,0063 |
| A\_51\_P492389 | NM\_172462 | zinc finger protein 11 | 1.43 | 0,0010 |
| A\_51\_P132233 | AK032440 | unknown EST | 1.43 | 0,0293 |
| A\_51\_P298997 | NM\_177266 | similar to ELONGATION FACTOR G2 (HYPOTHETICAL 86.6 KDA PROTEIN) [Homo sapiens] | 1.43 | 0,0034 |
| A\_51\_P206655 | AK041290 | Mus musculus, Similar to Lsm1 protein, clone MGC:29255 IMAGE:5054997, mRNA, complete cds | 1.43 | 0,0214 |
| A\_51\_P482123 | NM\_007902 | Mus musculus endothelin 2 (Edn2), mRNA | 1.43 | 0,0054 |
| A\_51\_P246146 | NM\_133714 | Mus musculus RIKEN cDNA 2310037I24 gene (2310037I24Rik), mRNA | 1.43 | 0,0071 |
| A\_51\_P452820 | NM\_053257 | Mus musculus ribosomal protein L31 (Rpl31), mRNA | 1.43 | 0,0202 |
| A\_51\_P207451 | NM\_199062 | weakly similar to DNA-BINDING PROTEIN (FRAGMENT) [Homo sapiens] | 1.43 | 0,0298 |
| A\_51\_P458194 | NM\_009528 | Mus musculus wingless-related MMTV integration site 7B (Wnt7b), mRNA | 1.43 | 0,0077 |
| A\_51\_P200535 | AK050387 | hypothetical protein | 1.43 | 0,0331 |
| A\_51\_P173555 | NM\_019920 | Mus musculus mitogen-activated protein kinase kinase 1 interacting protein 1 (Map2k1ip1), mRNA | 1.43 | 0,0182 |
| A\_51\_P216758 | NM\_021529 | Mus musculus phosphatase subunit gene g4-1 (G4-1-pending), mRNA | 1.43 | 0,0239 |
| A\_51\_P353125 | NM\_211138 | CHOLINEPHOSPHATE CYTIDYLYLTRANSFERASE B (EC 2.7.7.15) (PHOSPHORYLCHOLINE TRANSFERASE B) (CTP:PHOSPHOCHOLINE CYTIDYLYLTRANSFERASE B) (CT B) (CCT B) (CCT-BETA) homolog [Rattus norvegicus] | 1.43 | 0,0112 |
| A\_51\_P307325 | NM\_009030 | Mus musculus retinoblastoma binding protein 4 (Rbbp4), mRNA | 1.43 | 0,0069 |
| A\_51\_P188462 | NAP057293-1 | Mus musculus olfactory receptor MOR135-16P (MOR135-16P) pseudogene | 1.43 | 0,0059 |
| A\_51\_P263983 | NM\_146908 | Mus musculus olfactory receptor MOR248-1 (MOR248-1), mRNA | 1.43 | 0,0052 |
| A\_51\_P176693 | NM\_146105 | Mus musculus RIKEN cDNA 9630058J23 gene (9630058J23Rik), mRNA | 1.43 | 0,0228 |
| A\_51\_P395555 | NM\_178600 | hypothetical protein | 1.43 | 0,0341 |
| A\_51\_P363258 | NM\_133731 | Mus musculus RIKEN cDNA 4733401N09 gene (4733401N09Rik), mRNA | 1.43 | 0,0051 |
| A\_51\_P116027 | NM\_026896 | P37 TRAP/SMCC/PC2 SUBUNIT homolog [Homo sapiens] | 1.43 | 0,0376 |
| A\_51\_P209319 | NM\_016752 | Mus musculus UDP-galactose translocator 2 (Ugalt2), mRNA | 1.43 | 0,0049 |
| A\_51\_P364231 | NM\_010752 | Mus musculus mitotic arrest deficient 1-like 1 (Mad1l1), mRNA | 1.43 | 0,0184 |
| A\_51\_P377482 | NAP057069-1 | Mus musculus olfactory receptor GA\_x5J8B7W6RL5-13451244-13452122 (GA\_x5J8B7W6RL5-13451244-13452122) pseudogene | 1.43 | 0,0089 |
| A\_51\_P193116 | NM\_198642 | weakly similar to CDNA: FLJ21562 FIS, CLONE COL06420 [Homo sapiens] | 1.43 | 0,0028 |
| A\_51\_P183261 | NM\_007474 | Mus musculus aquaporin 8 (Aqp8), mRNA | 1.43 | 0,0064 |
| A\_51\_P332081 | NM\_007619 | Mus musculus Casitas B-lineage lymphoma (Cbl), mRNA | 1.43 | 0,0111 |
| A\_51\_P229816 | NM\_145445 | Mus musculus, Similar to eukaryotic translation initiation factor 2B, subunit 2 (beta, 39kD), clone MGC:7057 IMAGE:3156632, mRNA, complete cds | 1.43 | 0,0266 |
| A\_51\_P430552 | NM\_175935 | hypothetical PA-phosphatase related phosphoesterase protein | 1.43 | 0,0070 |
| A\_51\_P457706 | NM\_175667 | ANKYRIN REPEAT DOMAIN PROTEIN 5 homolog [Mus musculus] | 1.43 | 0,0113 |
| A\_51\_P448252 | NM\_026170 | Mus musculus RIKEN cDNA 1200007D18 gene (1200007D18Rik), mRNA | 1.43 | 0,0430 |
| A\_51\_P349262 | NM\_013604 | Mus musculus metaxin (Mtx), mRNA | 1.43 | 0,0199 |
| A\_51\_P375267 | NM\_013810 | Mus musculus drebrin-like (Dbnl), mRNA | 1.43 | 0,0268 |
| A\_51\_P401001 | A\_51\_P401001 | Mus musculus cDNA, 3 end | 1.43 | 0,0127 |
| A\_51\_P372472 | NM\_016709 | Mus musculus AU RNA binding protein/enoyl-coenzyme A hydratase (Auh), mRNA | 1.43 | 0,0049 |
| A\_51\_P464361 | NM\_172283 | L FUCOSE | 1.43 | 0,0458 |
| A\_51\_P513979 | NM\_027283 | hypothetical protein | 1.42 | 0,0005 |
| A\_51\_P506674 | NM\_001013376 | similar to RIBONUCLEASE P PROTEIN SUBUNIT P38 (EC 3.1.26.5) (RNASEP PROTEIN P38) [Homo sapiens] | 1.42 | 0,0438 |
| A\_51\_P187171 | NM\_030252 | Mus musculus hypothetical protein, MGC:7764 (BC003266), mRNA | 1.42 | 0,0059 |
| A\_51\_P453043 | NM\_030210 | ACETOACETYL-COA SYNTHETASE (EC 6.2.1.16) homolog [Rattus norvegicus] | 1.42 | 0,0089 |
| A\_51\_P331589 | A\_51\_P331589 | Mus musculus cDNA, 5 end | 1.42 | 0,0122 |
| A\_51\_P383584 | XM\_619530 | hypothetical jmjC domain containing protein | 1.42 | 0,0342 |
| A\_51\_P265696 | NM\_008085 | Mus musculus glyceraldehyde-3-phosphate dehydrogenase, spermatogenic (Gapds), mRNA | 1.42 | 0,0490 |
| A\_51\_P245546 | NM\_194341 | Mus musculus, Similar to AP1 gamma subunit binding protein 1, clone IMAGE:4458940, mRNA | 1.42 | 0,0167 |
| A\_51\_P474960 | NM\_145131 | Mus musculus, Similar to metalloprotease 1 (pitrilysin family), clone MGC:11969 IMAGE:3601081, mRNA, complete cds | 1.42 | 0,0280 |
| A\_51\_P384314 | AK038399 | unknown EST | 1.42 | 0,0294 |
| A\_51\_P368804 | NM\_023472 | Mus musculus ankyrin repeat, family A (RFXANK-like), 2 (Ankra2), mRNA | 1.42 | 0,0139 |
| A\_51\_P129260 | BI662359 | Mus musculus cDNA, 5 end | 1.42 | 0,0071 |
| A\_51\_P336161 | NM\_010764 | Mus musculus mannosidase 2, alpha B1 (Man2b1), mRNA | 1.42 | 0,0021 |
| A\_51\_P406429 | NM\_172665 | Mus musculus, Similar to pyruvate dehydrogenase kinase, isoenzyme 1, clone MGC:28719 IMAGE:4458562, mRNA, complete cds | 1.42 | 0,0482 |
| A\_51\_P376057 | NM\_178782 | hypothetical Ankyrin-repeat containing protein | 1.42 | 0,0111 |
| A\_51\_P447808 | NM\_172509 | CDNA FLJ32069 FIS, CLONE OCBBF1000118, WEAKLY SIMILAR TO TUMOR NECROSIS FACTOR, ALPHA-INDUCED PROTEIN 1, ENDOTHELIAL homolog [Homo sapiens] | 1.42 | 0,0060 |
| A\_51\_P324701 | NM\_025606 | Mus musculus mitochondrial ribosomal protein L16 (Mrpl16), mRNA | 1.42 | 0,0025 |
| A\_51\_P448127 | NM\_025890 | hypothetical Alanine-rich region containing protein | 1.42 | 0,0014 |
| A\_51\_P505230 | NM\_025332 | hypothetical P-loop containing nucleotide triphosphate hydrolases structure containing protein | 1.42 | 0,0047 |
| A\_51\_P104891 | AK122544 | Mus musculus cDNA, 3 end | 1.42 | 0,0226 |
| A\_51\_P121724 | AK016598 | similar to GLE1 [Homo sapiens] | 1.42 | 0,0090 |
| A\_51\_P373402 | NM\_026197 | hypothetical S-adenosyl-L-methionine-dependent methyltransferases structure containing protein | 1.42 | 0,0375 |
| A\_51\_P486091 | NM\_172150 | NGD5 PROTEIN homolog [Murinae gen. sp] | 1.42 | 0,0229 |
| A\_51\_P499876 | L20334 | Mouse EDG-like receptor mRNA, partial cds | 1.42 | 0,0034 |
| A\_51\_P403228 | NM\_025675 | Mus musculus RIKEN cDNA 5730421E18 gene (5730421E18Rik), mRNA | 1.42 | 0,0430 |
| A\_51\_P212993 | NM\_021499 | Mus musculus WD repeat domain 8 (Wdr8), mRNA | 1.42 | 0,0051 |
| A\_51\_P181205 | NM\_029418 | Mus musculus clone wz3#88 unknown mRNA | 1.42 | 0,0244 |
| A\_51\_P402165 | NM\_011880 | Mus musculus regulator of G protein signaling 7 (Rgs7), mRNA | 1.42 | 0,0355 |
| A\_51\_P244386 | AK086107 | MYOFERLIN (FER-1 LIKE PROTEIN 3) homolog [Homo sapiens] | 1.42 | 0,0014 |
| A\_51\_P175205 | AK079278 | RIBOSOMAL PROTEIN S6 KINASE ALPHA EC 2.7.1.- S6K ALPHA KDA RIBOSOMAL PROTEIN S6 KINASE P90 RSK RIBOSOMAL S6 KINASE RSK | 1.42 | 0,0222 |
| A\_51\_P518462 | AK034138 | weakly similar to ETAA16 PROTEIN [Homo sapiens] | 1.42 | 0,0270 |
| A\_51\_P182296 | NM\_026070 | PROTEIN AD-016 (PROTEIN CGI-116) (X0009) homolog [Homo sapiens] | 1.42 | 0,0314 |
| A\_51\_P250123 | NM\_028543 | similar to MSZF26 (FRAGMENT) [Mus musculus] | 1.42 | 0,0070 |
| A\_51\_P382551 | NM\_019496 | Mus musculus Alport syndrome, mental retardation, midface hypoplasia and elliptocytosis chromosomal region gene 1 homolog (human) (Ammecr1), mRNA | 1.41 | 0,0110 |
| A\_51\_P295446 | NM\_134100 | Mus musculus expressed sequence AW556797 (AW556797), mRNA | 1.41 | 0,0320 |
| A\_51\_P506054 | AK038997 | weakly similar to BCL2/ADENOVIRUS E1B 19-KDA PROTEIN-INTERACTING PROTEIN 2 [Homo sapiens] | 1.41 | 0,0094 |
| A\_51\_P441153 | NM\_144955 | Mus musculus homeodomain transcription factor (Nkx6-1) mRNA, complete cds | 1.41 | 0,0073 |
| A\_51\_P268131 | AK029230 | unknown EST | 1.41 | 0,0011 |
| A\_51\_P180654 | AK129084 | hypothetical protein | 1.41 | 0,0062 |
| A\_51\_P425352 | NM\_177049 | CDNA FLJ30783 FIS, CLONE FEBRA2000880, WEAKLY SIMILAR TO SARCALUMENIN PRECURSOR homolog [Homo sapiens] | 1.41 | 0,0177 |
| A\_51\_P388281 | NM\_009835 | Mus musculus chemokine (C-C) receptor 6 (Cmkbr6), mRNA | 1.41 | 0,0052 |
| A\_51\_P217094 | XM\_620495 | hypothetical ARM repeat structure containing protein | 1.41 | 0,0136 |
| A\_51\_P238554 | NM\_053181 | Mus musculus expressed sequence AA415817 (AA415817), mRNA | 1.41 | 0,0230 |
| A\_51\_P446112 | AK078307 | hypothetical protein | 1.41 | 0,0068 |
| A\_51\_P322109 | NM\_177242 | T CELL ACTIVATION PROTEIN PHOSPHATASE | 1.41 | 0,0083 |
| A\_51\_P158552 | NM\_016684 | Mus musculus zinc finger protein 96 (Zfp96) mRNA, complete cds | 1.41 | 0,0380 |
| A\_51\_P310576 | NM\_025471 | Mus musculus RIKEN cDNA 1810030N24 gene (1810030N24Rik), mRNA | 1.41 | 0,0336 |
| A\_51\_P285300 | NM\_021351 | Mus musculus crystallin, beta A4 (Cryba4), mRNA | 1.41 | 0,0017 |
| A\_51\_P105338 | NM\_172609 | MITOCHONDRIAL IMPORT RECEPTOR SUBUNIT TOM22 HOMOLOG | 1.41 | 0,0400 |
| A\_51\_P475545 | NM\_001003685 | Mus musculus (clone pCM7) growth hormone-releasing factor receptor mRNA sequence | 1.41 | 0,0122 |
| A\_51\_P374814 | NM\_028091 | similar to PUTATIVE SIALOGLYCOPROTEASE TYPE 2 [Homo sapiens] | 1.41 | 0,0099 |
| A\_51\_P466558 | XM\_131619 | Mus musculus similar to Early embryogenesis protein zyg-11 (LOC230590), mRNA | 1.41 | 0,0266 |
| A\_51\_P497263 | NM\_010837 | Mus musculus microtubule-associated protein 6 (Mtap6), mRNA | 1.41 | 0,0139 |
| A\_51\_P337691 | NM\_025985 | UBIQUITIN-CONJUGATING ENZYME E2 G1 (EC 6.3.2.19) (UBIQUITIN-PROTEIN LIGASE G1) (UBIQUITIN CARRIER PROTEIN G1) homolog [Homo sapiens] | 1.41 | 0,0095 |
| A\_51\_P376484 | AK078508 | Mus musculus cDNA, 3 end | 1.41 | 0,0364 |
| A\_51\_P513224 | XM\_622103 | hypothetical protein | 1.41 | 0,0066 |
| A\_51\_P127176 | NM\_175280 | hypothetical protein | 1.41 | 0,0181 |
| A\_51\_P356512 | NM\_026139 | Mus musculus RIKEN cDNA 3230401N03 gene (3230401N03Rik), mRNA | 1.41 | 0,0239 |
| A\_51\_P393399 | TC1415265 | Mus musculus aspartate-beta-hydroxylase (Asph), mRNA | 1.41 | 0,0272 |
| A\_51\_P173235 | NM\_028134 | hypothetical LysM motif containing protein | 1.41 | 0,0108 |
| A\_51\_P176522 | NM\_019574 | Mus musculus mazr mRNA for transcription factor MAZR, complete cds | 1.41 | 0,0023 |
| A\_51\_P281593 | BC062173 | weakly similar to HTPAP [Homo sapiens] | 1.41 | 0,0331 |
| A\_51\_P111586 | NM\_009319 | Mus musculus TAR (HIV) RNA binding protein 2 (Tarbp2), mRNA | 1.41 | 0,0341 |
| A\_51\_P247873 | NM\_026061 | Mus musculus RIKEN cDNA 2900010I05 gene (2900010I05Rik), mRNA | 1.41 | 0,0458 |
| A\_51\_P482434 | NAP057184-1 | Mus musculus olfactory receptor MOR137-1P (MOR137-1P) pseudogene | 1.40 | 0,0025 |
| A\_51\_P335480 | NM\_028077 | Mus musculus, RIKEN cDNA 1810055G02 gene, clone MGC:28461 IMAGE:4161022, mRNA, complete cds | 1.40 | 0,0448 |
| A\_51\_P518919 | NM\_172853 | CADHERIN-7 PRECURSOR homolog [Homo sapiens] | 1.40 | 0,0045 |
| A\_51\_P247450 | AK051714 | weakly similar to HYPOTHETICAL PROTEIN DJ1110N13.1 IN CHROMOSOME 7 (FRAGMENT) [Homo sapiens] | 1.40 | 0,0165 |
| A\_51\_P128174 | NM\_007937 | Mus musculus Eph receptor A5 (Epha5), mRNA | 1.40 | 0,0041 |
| A\_51\_P336398 | NM\_172049 | Mus musculus hypothetical protein DKFZp434C1714 (DKFZp434C1714), mRNA | 1.40 | 0,0160 |
| A\_51\_P518576 | NM\_145919 | SIMILAR TO DKFZP564O243 PROTEIN homolog [Mus musculus] | 1.40 | 0,0028 |
| A\_51\_P204504 | NM\_013925 | Mus musculus adenosine deaminase, tRNA-specific 1 (Adat1), mRNA | 1.40 | 0,0170 |
| A\_51\_P318375 | NM\_172480 | similar to METHIONINE SYNTHASE REDUCTASE [Homo sapiens] | 1.40 | 0,0282 |
| A\_51\_P370241 | AK015986 | hypothetical PDZ domain (also known as DHR or GLGF) containing protein | 1.40 | 0,0114 |
| A\_51\_P273405 | BC024833 | SPECTRIN BETA CHAIN, BRAIN 1 (SPECTRIN, NON-ERYTHROID BETA CHAIN 1) (BETA-II SPECTRIN) (FODRIN BETA CHAIN) | 1.40 | 0,0155 |
| A\_51\_P425165 | A\_51\_P425165 | Mus musculus cDNA, 5 end | 1.40 | 0,0354 |
| A\_51\_P356582 | NM\_147031 | Mus musculus olfactory receptor MOR264-1 (MOR264-1), mRNA | 1.40 | 0,0008 |
| A\_51\_P465128 | NM\_029935 | B CELL RAG-ASSOCIATED PROTEIN | 1.40 | 0,0192 |
| A\_51\_P232771 | NM\_025745 | Mus musculus, Similar to hypothetical protein, clone MGC:27955 IMAGE:3591901, mRNA, complete cds | 1.40 | 0,0050 |
| A\_51\_P112854 | NM\_152817 | Mus musculus RIKEN cDNA 2610511O17 gene (2610511O17Rik), mRNA | 1.40 | 0,0439 |
| A\_51\_P113672 | NM\_019994 | Mus musculus tuberin-like protein 1 (Tulip1-pending), mRNA | 1.40 | 0,0494 |
| A\_51\_P264064 | NM\_016692 | Mus musculus inner centromere protein (Incenp), mRNA | 1.40 | 0,0359 |
| A\_51\_P139462 | AK047901 | transcription factor ATF-a homolog [Homo sapiens] | 1.40 | 0,0398 |
| A\_51\_P357996 | NM\_013756 | Mus musculus defensin beta 3 (Defb3), mRNA | 1.40 | 0,0070 |
| A\_51\_P224592 | NM\_026823 | Mus musculus, clone MGC:28025 IMAGE:3661329, mRNA, complete cds | 1.40 | 0,0368 |
| A\_51\_P230663 | AK044887 | GAMMA-TUBULIN COMPLEX COMPONENT GCP5 homolog [Homo sapiens] | 1.40 | 0,0032 |
| A\_51\_P373770 | NAP057106-1 | Mus musculus olfactory receptor GA\_x5J8B7W5Q32-585040-584392 (GA\_x5J8B7W5Q32-585040-584392) pseudogene | 1.40 | 0,0034 |
| A\_51\_P268469 | NM\_053144 | Mus musculus protocadherin beta 19 (Pcdhb19), mRNA | 1.40 | 0,0151 |
| A\_51\_P441469 | NM\_021310 | Mus musculus junction-mediating and regulatory protein (Jmy-pending), mRNA | 1.40 | 0,0332 |
| A\_51\_P361426 | AK075941 | musculus 13 days embryo head cDNA, RIKEN full-length enriched library, clone:3100002J04:unclassifiable transcript, full insert sequence | 1.40 | 0,0392 |
| A\_51\_P460828 | AK129210 | Mus musculus pallidin (Pldn), mRNA | 1.40 | 0,0118 |
| A\_51\_P488389 | AK080053 | unclassifiable | 1.40 | 0,0070 |
| A\_51\_P397003 | XM\_133990 | CDNA FLJ14906 FIS, CLONE PLACE1005890, WEAKLY SIMILAR TO BEM46 PROTEIN homolog [Homo sapiens] | 1.40 | 0,0268 |
| A\_51\_P363668 | NM\_011276 | Mus musculus Hoxa1 regulated gene (Ha1r-pending), mRNA | 1.40 | 0,0434 |
| A\_51\_P173906 | NM\_026658 | Mus musculus mitochondrial translation optimization 1 homolog (S. cerevisiae) (Mto1-pending), mRNA | 1.40 | 0,0441 |
| A\_51\_P391934 | NM\_029706 | carboxypeptidase B1 (tissue) | 1.40 | 0,0043 |
| A\_51\_P453826 | NM\_146967 | Mus musculus olfactory receptor MOR233-2 (MOR233-2), mRNA | 1.40 | 0,0108 |
| A\_51\_P199871 | NM\_023371 | Mus musculus protein (peptidyl-prolyl cis/trans isomerase) NIMA-interacting 1 (Pin1), mRNA | 1.40 | 0,0130 |
| A\_51\_P167932 | NM\_177921 | hypothetical protein | 1.40 | 0,0010 |
| A\_51\_P419293 | NM\_030060 | JUN DIMERIZATION PROTEIN 1 JDP-1 homolog [Rattus norvegicus] | 1.40 | 0,0423 |
| A\_51\_P324070 | NM\_172606 | TEB4 PROTEIN homolog [Homo sapiens] | 1.40 | 0,0106 |
| A\_51\_P418540 | NM\_010722 | Mus musculus lamin B2 (Lmnb2), mRNA | 1.40 | 0,0246 |
| A\_51\_P212271 | NM\_031998 | Mus musculus testis specific gene A14 (Tsga14), mRNA | 1.40 | 0,0265 |
| A\_51\_P155935 | NM\_144905 | hypothetical Mitochondrial energy transfer proteins (carrier protein) containing protein | 1.39 | 0,0489 |
| A\_51\_P425674 | AK005024 | isovaleryl coenzyme A dehydrogenase | 1.39 | 0,0045 |
| A\_51\_P267353 | NM\_175478 | hypothetical Leucine-rich repeat containing protein | 1.39 | 0,0145 |
| A\_51\_P231099 | NM\_009996 | Mus musculus cytochrome P450, 24 (Cyp24), mRNA | 1.39 | 0,0033 |
| A\_51\_P243709 | AK034313 | inferred: tumor suppressing subtransferable candidate 1 {Homo sapiens} | 1.39 | 0,0348 |
| A\_51\_P370315 | NM\_011937 | Mus musculus glucosamine-6-phosphate deaminase (Gnpi), mRNA | 1.39 | 0,0009 |
| A\_51\_P248516 | NM\_173180 | MITOCHONDRIAL PROCESSING PEPTIDASE ALPHA SUBUNIT, MITOCHONDRIAL PRECURSOR (EC 3.4.24.64) (ALPHA-MPP) (P-55) homolog [Mus musculus] | 1.39 | 0,0109 |
| A\_51\_P258078 | NM\_138747 | Mus musculus, Similar to nucleolar protein 1 (120kD), clone MGC:6746 IMAGE:3592148, mRNA, complete cds | 1.39 | 0,0472 |
| A\_51\_P365068 | AK004258 | similar to KERATIN ASSOCIATED PROTEIN 4.15 (FRAGMENT) [Homo sapiens] | 1.39 | 0,0428 |
| A\_51\_P318683 | AF031816 | Mus musculus hybrid receptor gp250 precursor, mRNA, partial cds | 1.39 | 0,0391 |
| A\_51\_P293926 | NM\_030702 | Mus musculus smt3-specific isopeptidase 1 (Smt3ip1-pending), mRNA | 1.39 | 0,0212 |
| A\_51\_P115234 | NM\_134210 | Mus musculus vomeronasal 1 receptor, G9 (V1rg9), mRNA | 1.39 | 0,0182 |
| A\_51\_P311105 | NM\_009134 | Mus musculus sodium channel, voltage-gated, type X, alpha polypeptide (Scn10a), mRNA | 1.39 | 0,0028 |
| A\_51\_P435487 | AK005717 | similar to DJ63M2.2 (SIMILAR TO ACTIN) (FRAGMENT) [Homo sapiens] | 1.39 | 0,0025 |
| A\_51\_P427972 | AK048470 | hypothetical Guanylate-kinase-associated protein (GKAP) protein containing protein | 1.39 | 0,0027 |
| A\_51\_P446731 | AK010853 | similar to ZINC FINGER PROTEIN 4 (FRAGMENT) [Rattus norvegicus] | 1.39 | 0,0428 |
| A\_51\_P149997 | NM\_172456 | similar to ENDONUCLEASE G LIKE 1 (EC 3.1.30.-) (ENDO G LIKE) [Homo sapiens] | 1.39 | 0,0028 |
| A\_51\_P380950 | AK029164 | PUTATIVE ALPHA-MANNOSIDASE IN CHROMOSOME 20 PRECURSOR (EC 3.2.1.-) homolog [Homo sapiens] | 1.39 | 0,0059 |
| A\_51\_P108645 | NM\_010587 | Mus musculus intersectin (SH3 domain protein 1A) (Itsn), mRNA | 1.39 | 0,0158 |
| A\_51\_P111068 | NM\_146734 | Mus musculus olfactory receptor MOR204-13 (MOR204-13), mRNA | 1.39 | 0,0084 |
| A\_51\_P195573 | NM\_010066 | Mus musculus DNA methyltransferase (cytosine-5) 1 (Dnmt1), mRNA | 1.39 | 0,0129 |
| A\_51\_P454337 | NM\_001011833 | Mus musculus olfactory receptor MOR40-9P (MOR40-9P) pseudogene | 1.39 | 0,0086 |
| A\_51\_P441822 | NM\_144878 | Mus musculus flavin-containing monooxygenase 4 mRNA, complete cds | 1.39 | 0,0049 |
| A\_51\_P208511 | NM\_009924 | Mus musculus cannabinoid receptor 2 (macrophage) (Cnr2), mRNA | 1.39 | 0,0296 |
| A\_51\_P468544 | NM\_009237 | Mus musculus SRY-box containing gene 3 (Sox3), mRNA | 1.39 | 0,0058 |
| A\_51\_P130567 | NM\_020291 | Mus musculus odorant receptor S25 gene (Ors25), mRNA | 1.39 | 0,0025 |
| A\_51\_P167284 | NM\_025984 | Mus musculus small proline rich-like 3 (Sprrl3), mRNA | 1.39 | 0,0101 |
| A\_51\_P422308 | NM\_130454 | Mus musculus RecQ protein-like 5 (Recql5), mRNA | 1.39 | 0,0381 |
| A\_51\_P484238 | NM\_027530 | RAP2 INTERACTING PROTEIN X homolog [Homo sapiens] | 1.39 | 0,0227 |
| A\_51\_P488308 | NM\_139061 | Mus musculus VPS54-like (yeast) (Vps54l-pending), mRNA | 1.39 | 0,0096 |
| A\_51\_P485483 | NM\_020027 | Mus musculus HLA-B associated transcript 2 (Bat2), mRNA | 1.39 | 0,0053 |
| A\_51\_P309618 | NM\_025904 | Mus musculus RIKEN cDNA 1600012F09 gene (1600012F09Rik), mRNA | 1.39 | 0,0300 |
| A\_51\_P234462 | NM\_133756 | Mus musculus RIKEN cDNA 2410004J02 gene (2410004J02Rik), mRNA | 1.39 | 0,0228 |
| A\_51\_P450411 | BC058768 | Mus musculus mucin glycoprotein MUC3 mRNA, partial cds | 1.39 | 0,0086 |
| A\_51\_P248181 | NM\_177818 | hypothetical Immunoglobulin and major histocompatibility complex domain containing protein | 1.39 | 0,0192 |
| A\_51\_P191893 | AK054069 | hypothetical protein | 1.39 | 0,0135 |
| A\_51\_P315931 | NM\_033314 | Mus musculus solute carrier family 21 (prostaglandin transporter), member 2 (Slc21a2), mRNA | 1.39 | 0,0443 |
| A\_51\_P189151 | AK028396 | hypothetical BTB/POZ domain containing protein | 1.39 | 0,0050 |
| A\_51\_P249414 | NM\_022408 | Mus musculus expressed sequence 2 embryonic lethal (Es2el), mRNA | 1.39 | 0,0181 |
| A\_51\_P207921 | NM\_008898 | Mus musculus P450 (cytochrome) oxidoreductase (Por), mRNA | 1.39 | 0,0488 |
| A\_51\_P211519 | NM\_030728 | Mus musculus hypothetical protein, 12H19.01.T7 (AY007814), mRNA | 1.39 | 0,0315 |
| A\_51\_P172688 | NM\_024270 | Mus musculus, Similar to RIKEN cDNA 0610035N01 gene, clone MGC:6324 IMAGE:3256725, mRNA, complete cds | 1.39 | 0,0346 |
| A\_51\_P182876 | D86419 | Mus musculus mRNA for glycine tyrosine-rich hair keratin protein, complete cds | 1.39 | 0,0090 |
| A\_51\_P326043 | NM\_172916 | hypothetical protein | 1.39 | 0,0118 |
| A\_51\_P150242 | NM\_146744 | Mus musculus olfactory receptor MOR256-8 (MOR256-8), mRNA | 1.39 | 0,0027 |
| A\_51\_P277139 | AK017078 | Mus musculus RIKEN cDNA 4933436E20 gene (4933436E20Rik), mRNA | 1.39 | 0,0075 |
| A\_51\_P154788 | AK046926 | QUAKING (FRAGMENT) | 1.39 | 0,0131 |
| A\_51\_P462746 | BC006867 | Mus musculus, clone MGC:11792 IMAGE:3595167, mRNA, complete cds | 1.39 | 0,0450 |
| A\_51\_P322990 | NM\_175001 | CDNA FLJ20594 FIS, CLONE KAT08731 (SIMILAR TO MITOCHONDRIAL RIBOSOMAL PROTEIN L22) homolog [Homo sapiens] | 1.39 | 0,0215 |
| A\_51\_P358243 | AK011611 | hypothetical Rhodanese/cdc25 fold/Rhodanese signatures containing protein | 1.39 | 0,0046 |
| A\_51\_P515482 | NM\_019817 | Mus musculus coatomer protein complex, subunit zeta 1 (Copz1), mRNA | 1.39 | 0,0426 |
| A\_51\_P277321 | NM\_146085 | Mus musculus, Similar to amyloid beta (A4) precursor protein-binding, family B, member 3, clone MGC:38710 IMAGE:5357681, mRNA, complete cds | 1.39 | 0,0233 |
| A\_51\_P466773 | NM\_172749 | similar to HYPOTHETICAL ZINC FINGER PROTEIN KIAA0296 [Homo sapiens] | 1.39 | 0,0229 |
| A\_51\_P347634 | AK036522 | hypothetical protein | 1.39 | 0,0183 |
| A\_51\_P201840 | NM\_021608 | Mus musculus dynactin 5 (Dctn5), mRNA | 1.39 | 0,0354 |
| A\_51\_P455371 | NM\_178263 | Mus musculus, Similar to hypothetical protein DKFZp434L0718, clone MGC:25907 IMAGE:4221252, mRNA, complete cds | 1.39 | 0,0055 |
| A\_51\_P488230 | AK036733 | unknown EST | 1.39 | 0,0022 |
| A\_51\_P310734 | NM\_178239 | similar to NADPH-DEPENDENT FMN AND FAD CONTAINING OXIDOREDUCTASE [Homo sapiens] | 1.39 | 0,0196 |
| A\_51\_P503052 | BC026755 | Mus musculus, Similar to sperm specific antigen 1, clone MGC:25294 IMAGE:3154066, mRNA, complete cds | 1.39 | 0,0120 |
| A\_51\_P181538 | NM\_145125 | Mus musculus mRNA for WDR9 protein (Wdr9 gene), form A | 1.39 | 0,0379 |
| A\_51\_P375111 | NM\_007602 | Mus musculus calpain 5 (Capn5), mRNA | 1.39 | 0,0057 |
| A\_51\_P450320 | NM\_175086 | TYPE-1B ANGIOTENSIN II RECEPTOR | 1.39 | 0,0428 |
| A\_51\_P383320 | NM\_009543 | Mus musculus zinc finger protein 103 (Zfp103), mRNA | 1.39 | 0,0379 |
| A\_51\_P183300 | NM\_007506 | Mus musculus ATP synthase, H+ transporting, mitochondrial F0 complex, subunit c (subunit 9), isoform 1 (Atp5g1), mRNA | 1.39 | 0,0194 |
| A\_51\_P359806 | AK014741 | unknown EST | 1.39 | 0,0368 |
| A\_51\_P190805 | NM\_007456 | Mus musculus adaptor-related protein complex AP-1, mu subunit 1 (Ap1m1), mRNA | 1.38 | 0,0250 |
| A\_51\_P493264 | AK043682 | similar to PEROXISOME ASSEMBLY PROTEIN 10 (PEROXIN-10) [Homo sapiens] | 1.38 | 0,0038 |
| A\_51\_P286399 | NM\_181075 | HISTOCOMPATIBILITY 2, CLASS II ANTIGEN E BETA | 1.38 | 0,0142 |
| A\_51\_P164562 | NM\_012005 | Mus musculus cDNA clone IMAGE:3384363 5 similar to TR:O60244 O60244 EXML1. [1] | 1.38 | 0,0206 |
| A\_51\_P332185 | AK021136 | hypothetical protein | 1.38 | 0,0182 |
| A\_51\_P157982 | NM\_029930 | Mus musculus 0 day neonate eyeball cDNA, RIKEN full-length enriched library, clone:E130103N08 product:hypothetical protein, full insert sequence. | 1.38 | 0,0202 |
| A\_51\_P387968 | NM\_198113 | Mus musculus, Similar to slingshot 3, clone MGC:25738 IMAGE:3987714, mRNA, complete cds | 1.38 | 0,0481 |
| A\_51\_P497882 | NM\_030080 | Mus musculus Attaches to Cre (Atce1) | 1.38 | 0,0089 |
| A\_51\_P135357 | NM\_025679 | Mus musculus RIKEN cDNA 5730470L24 gene (5730470L24Rik), mRNA | 1.38 | 0,0012 |
| A\_51\_P348397 | NM\_138753 | Mus musculus cardiac lineage protein 1 (Clp1), mRNA | 1.38 | 0,0250 |
| A\_51\_P367843 | NM\_026329 | Mus musculus polymerase (RNA) II (DNA directed) polypeptide G (Polr2g), mRNA | 1.38 | 0,0308 |
| A\_51\_P211165 | AK088130 | Mus musculus, clone IMAGE:3598145, mRNA, partial cds | 1.38 | 0,0019 |
| A\_51\_P457599 | AK083090 | Mus musculus otospiralin (LOC260301), mRNA | 1.38 | 0,0108 |
| A\_51\_P471235 | NM\_001011828 | Mus musculus olfactory receptor MOR219-3P (MOR219-3P) pseudogene | 1.38 | 0,0041 |
| A\_51\_P119659 | NM\_008059 | Mus musculus G0/G1 switch gene 2 (G0s2), mRNA | 1.38 | 0,0157 |
| A\_51\_P513846 | NM\_026319 | Mus musculus RIKEN cDNA 1700029H06 gene (1700029H06Rik), mRNA | 1.38 | 0,0398 |
| A\_51\_P452153 | NM\_027222 | weakly similar to PROAPOPTOTIC CASPASE ADAPTOR PROTEIN [Homo sapiens] | 1.38 | 0,0077 |
| A\_51\_P221930 | AK083920 | CDNA FLJ30803 FIS, CLONE FEBRA2001245, WEAKLY SIMILAR TO NAG14 homolog [Homo sapiens] | 1.38 | 0,0131 |
| A\_51\_P500814 | NM\_010708 | Mus musculus lectin, galactose binding, soluble 9 (Lgals9), mRNA | 1.38 | 0,0302 |
| A\_51\_P404204 | NM\_029780 | Mus musculus v-raf-1 leukemia viral oncogene 1 (Raf1), mRNA | 1.38 | 0,0411 |
| A\_51\_P474535 | NM\_144852 | similar to CATIONIC AMINO ACID TRANSPORTER-4 (CAT-4) (CAT4) [Homo sapiens] | 1.38 | 0,0456 |
| A\_51\_P242021 | NM\_019400 | Mus musculus rabaptin 5 (Rab5ep-pending), mRNA | 1.38 | 0,0263 |
| A\_51\_P292630 | BC027189 | weakly similar to URIDINE PHOSPHORYLASE (EC 2.4.2.3) (UDRPASE) [Mus musculus] | 1.38 | 0,0204 |
| A\_51\_P161902 | XM\_355476 | hypothetical protein | 1.38 | 0,0036 |
| A\_51\_P290788 | NM\_025730 | Mus musculus RIKEN cDNA 4921513O20 gene (4921513O20Rik), mRNA | 1.38 | 0,0275 |
| A\_51\_P152423 | NM\_177640 | hypothetical Actinin-type actin-binding domain containing protein | 1.38 | 0,0203 |
| A\_51\_P363644 | NM\_138581 | Mus musculus DNA segment, Human EST J0827E04 (ESTJ0827E04), mRNA | 1.38 | 0,0143 |
| A\_51\_P229759 | AK054255 | hypothetical Cysteine-rich flanking region, C-terminal/Leucine-rich repeat/Immunoglobulin and major histocompatibility complex domain/Immunoglobulin C-2 type/Leucine-rich repeat, typical subtype/Immunoglobulin subtype containing protein | 1.38 | 0,0417 |
| A\_51\_P327585 | NM\_026764 | Mus musculus glutathione transferase GSTM7-7 mRNA, complete cds | 1.38 | 0,0142 |
| A\_51\_P393409 | XM\_284227 | weakly similar to CDNA FLJ31638 FIS, CLONE NT2RI2003556 [Homo sapiens] | 1.38 | 0,0089 |
| A\_51\_P416349 | NM\_029705 | SPINOCEREBELLAR ATAXIA TYPE 3 homolog [Rattus norvegicus] | 1.38 | 0,0366 |
| A\_51\_P436610 | BC021953 | Mus musculus, clone IMAGE:5133935, mRNA | 1.38 | 0,0161 |
| A\_51\_P353502 | NM\_025340 | Mus musculus, Similar to shank-interacting protein, clone MGC:27664 IMAGE:4527839, mRNA, complete cds | 1.38 | 0,0195 |
| A\_51\_P155514 | NM\_011164 | Mus musculus prolactin (Prl), mRNA | 1.38 | 0,0084 |
| A\_51\_P396635 | AK037839 | mitochondria located 1 homolog (human) | 1.38 | 0,0171 |
| A\_51\_P405304 | NM\_053172 | Mus musculus matrix extracellular phosphoglycoprotein with ASARM motif (bone) (Mepe), mRNA | 1.38 | 0,0265 |
| A\_51\_P229325 | NM\_027188 | hypothetical SET-domain of transcriptional regulators (TRX, EZ, ASH1 etc)/MYND zinc finger (ZnF) domain containing protein | 1.38 | 0,0280 |
| A\_51\_P185499 | NM\_008923 | Mus musculus protein kinase, cAMP dependent regulatory, type I beta (Prkar1b), mRNA | 1.38 | 0,0103 |
| A\_51\_P272172 | NM\_053068 | Mus musculus chromatin accessibility complex 1 (Chrac1), mRNA | 1.37 | 0,0383 |
| A\_51\_P446623 | NM\_138309 | Mus musculus Mic2l1 mRNA, complete cds | 1.37 | 0,0125 |
| A\_51\_P110220 | AK083290 | inferred: dM417G6.4 (novel protein) {Mus musculus} | 1.37 | 0,0277 |
| A\_51\_P278550 | NM\_134118 | SC2 homolog [Rattus sp] | 1.37 | 0,0244 |
| A\_51\_P153173 | AK021349 | unknown EST | 1.37 | 0,0234 |
| A\_51\_P129546 | NM\_009219 | Mus musculus somatostatin receptor 4 (Smstr4), mRNA | 1.37 | 0,0083 |
| A\_51\_P162144 | NM\_021879 | Mus musculus pink-eyed dilution (p), mRNA | 1.37 | 0,0282 |
| A\_51\_P380587 | NM\_028959 | hypothetical Outer arm dynein light chain 1 structure containing protein | 1.37 | 0,0080 |
| A\_51\_P455861 | NM\_027105 | hypothetical protein | 1.37 | 0,0048 |
| A\_51\_P417077 | NM\_011700 | Mus musculus partial mRNA for villin-like protein (Vill gene) | 1.37 | 0,0028 |
| A\_51\_P340819 | NM\_008389 | Mus musculus IAP promoted placental gene (Ipp), mRNA | 1.37 | 0,0394 |
| A\_51\_P211291 | NM\_025510 | Mus musculus RIKEN cDNA 2310004I24 gene (2310004I24Rik), mRNA | 1.37 | 0,0106 |
| A\_51\_P337756 | NM\_026107 | Mus musculus RIKEN cDNA 1700128E15 gene (1700128E15Rik), mRNA | 1.37 | 0,0475 |
| A\_51\_P448555 | NM\_008663 | Mus musculus myosin VIIa (Myo7a), mRNA | 1.37 | 0,0084 |
| A\_51\_P246187 | AK010168 | SEVEN TRANSMEMBRANE DOMAIN PROTEIN homolog [Homo sapiens] | 1.37 | 0,0320 |
| A\_51\_P196127 | NM\_011863 | Mus musculus 3-phosphoadenosine 5-phosphosulfate synthase 1 (Papss1), mRNA | 1.37 | 0,0056 |
| A\_51\_P157118 | NM\_008458 | Mus musculus serine (or cysteine) proteinase inhibitor, clade A, member 3C (Serpina3c), mRNA | 1.37 | 0,0076 |
| A\_51\_P276452 | NM\_178646 | hypothetical CENP-B protein containing protein | 1.37 | 0,0274 |
| A\_51\_P160241 | NM\_146370 | Mus musculus olfactory receptor B12 mRNA, partial cds | 1.37 | 0,0151 |
| A\_51\_P269709 | NM\_008462 | Mus musculus killer cell lectin-like receptor, subfamily A, member 2 (Klra2), mRNA | 1.37 | 0,0086 |
| A\_51\_P207275 | AK036825 | unclassifiable | 1.37 | 0,0106 |
| A\_51\_P340947 | NM\_027905 | weakly similar to DJ402G11.5 (NOVEL PROTEIN SIMILAR TO YEAST AND BACTERIAL PREDICTED PROTEINS) [Homo sapiens] | 1.37 | 0,0209 |
| A\_51\_P123066 | AK016133 | hypothetical protein | 1.37 | 0,0468 |
| A\_51\_P381157 | NM\_027420 | hypothetical protein | 1.37 | 0,0242 |
| A\_51\_P382859 | AK049769 | hypothetical Phenylalanine-rich region containing protein | 1.37 | 0,0318 |
| A\_51\_P274137 | AK009071 | hypothetical protein | 1.37 | 0,0030 |
| A\_51\_P349691 | NM\_133979 | Mus musculus, clone IMAGE:3590438, mRNA, partial cds | 1.37 | 0,0113 |
| A\_51\_P222153 | NM\_009419 | Mus musculus protein-tyrosine sulfotransferase 2 (Tpst2), mRNA | 1.37 | 0,0375 |
| A\_51\_P322851 | NM\_147083 | Mus musculus olfactory receptor MOR26-1 (MOR26-1), mRNA | 1.37 | 0,0046 |
| A\_51\_P515349 | AK049359 | PHOSPHATIDYLGLYCEROPHOSPHATE SYNTHASE homolog [Cricetulus griseus] | 1.37 | 0,0151 |
| A\_51\_P241159 | NM\_021536 | Mus musculus RIKEN cDNA 2210403N23 gene (2210403N23Rik), mRNA | 1.37 | 0,0341 |
| A\_51\_P373696 | AK031222 | hypothetical Signal peptidase containing protein | 1.37 | 0,0228 |
| A\_51\_P315077 | BC018498 | Mus musculus, Similar to RIKEN cDNA 5033428A16 gene, clone MGC:27916 IMAGE:3501540, mRNA, complete cds | 1.37 | 0,0229 |
| A\_51\_P162008 | NM\_175501 | ADAMTS-12 PRECURSOR (EC 3.4.24.-) (A DISINTEGRIN AND METALLOPROTEINASE WITH THROMBOSPONDIN MOTIFS 12) (ADAM-TS 12) (ADAM- TS12) homolog [Homo sapiens] | 1.37 | 0,0110 |
| A\_51\_P500981 | NM\_008635 | Mus musculus microtubule-associated protein 7 (Mtap7), mRNA | 1.37 | 0,0122 |
| A\_51\_P388310 | NM\_009873 | Mus musculus cyclin-dependent kinase 6 (Cdk6), mRNA | 1.36 | 0,0062 |
| A\_51\_P475858 | NM\_008990 | Mus musculus poliovirus sensitivity (Pvs), mRNA | 1.36 | 0,0013 |
| A\_51\_P117165 | AK018150 | hypothetical protein | 1.36 | 0,0264 |
| A\_51\_P328416 | AK020388 | unknown EST | 1.36 | 0,0104 |
| A\_51\_P436401 | NM\_010502 | Mus musculus interferon alpha family, gene 1 (Ifna1), mRNA | 1.36 | 0,0266 |
| A\_51\_P116298 | AK006122 | similar to BA305P22.2.5 (NOVEL PROTEIN, ISOFORM 5) (FRAGMENT) [Homo sapiens] | 1.36 | 0,0201 |
| A\_51\_P112833 | AK007293 | hypothetical protein | 1.36 | 0,0233 |
| A\_51\_P245102 | NM\_026596 | Mus musculus RIKEN cDNA 4930591A17 gene (4930591A17Rik), mRNA | 1.36 | 0,0220 |
| A\_51\_P272303 | NM\_144790 | hypothetical Ankyrin repeat profile/Ankyrin-repeat/Ankyrin repeat region circular profile/Yeast DNA-binding domain containing protein | 1.36 | 0,0050 |
| A\_51\_P248230 | NM\_145099 | Mus musculus transient receptor potential cation channel, subfamily V, member 3 (Trpv3), mRNA | 1.36 | 0,0221 |
| A\_51\_P318281 | NM\_031186 | Mus musculus N-deacetylase/N-sulfotransferase (heparan glucosaminyl) 3 (Ndst3), mRNA | 1.36 | 0,0021 |
| A\_51\_P254583 | NM\_172291 | Mus musculus hypothetical protein LOC235169 (LOC235169), mRNA | 1.36 | 0,0431 |
| A\_51\_P473288 | AK076599 | unknown EST | 1.36 | 0,0041 |
| A\_51\_P266191 | AK018108 | hypothetical Kelch repeat containing protein | 1.36 | 0,0413 |
| A\_51\_P381086 | NM\_178699 | BRAIN CDNA, CLONE MNCB-3966 (FRAGMENT) | 1.36 | 0,0043 |
| A\_51\_P145357 | NM\_011354 | small EDRK-rich factor 2 | 1.36 | 0,0476 |
| A\_51\_P124675 | XM\_134088 | hypothetical protein | 1.36 | 0,0233 |
| A\_51\_P182267 | NM\_026010 | Mus musculus RIKEN cDNA 2610209A20 gene (2610209A20Rik), mRNA | 1.36 | 0,0374 |
| A\_51\_P459988 | NAP108728-1 | Mus musculus olfactory receptor MOR248-12 (MOR248-12) pseudogene | 1.36 | 0,0037 |
| A\_51\_P223458 | NM\_025945 | Mus musculus, RIKEN cDNA 2810426M17 gene, clone MGC:27674 IMAGE:4911475, mRNA, complete cds | 1.36 | 0,0471 |
| A\_51\_P401337 | AK017242 | hypothetical protein | 1.36 | 0,0048 |
| A\_51\_P137800 | NM\_175319 | hypothetical Purple acid phosphatase, C-terminal/Serine/threonine specific protein phosphatase/Purple acid phosphatase, N-terminal containing protein | 1.36 | 0,0230 |
| A\_51\_P209460 | NM\_026585 | hypothetical protein | 1.36 | 0,0113 |
| A\_51\_P329958 | NM\_138594 | Mus musculus DNA segment, Chr 6, Wayne State University 163, expressed (D6Wsu163e), mRNA | 1.36 | 0,0231 |
| A\_51\_P290626 | NM\_178747 | Mus musculus, clone MGC:37880 IMAGE:5101228, mRNA, complete cds | 1.36 | 0,0030 |
| A\_51\_P353772 | NM\_008126 | Mus musculus gap junction membrane channel protein beta 3 (Gjb3), mRNA | 1.36 | 0,0082 |
| A\_51\_P112639 | NM\_009210 | Mus musculus SWI/SNF related, matrix associated, actin dependent regulator of chromatin, subfamily a, member 3 (Smarca3), mRNA | 1.36 | 0,0245 |
| A\_51\_P407904 | NM\_021789 | Mus musculus synbindin (Sbdn), mRNA | 1.36 | 0,0365 |
| A\_51\_P424211 | NM\_008557 | Mus musculus FXYD domain-containing ion transport regulator 3 (Fxyd3), mRNA | 1.36 | 0,0226 |
| A\_51\_P168392 | NM\_030188 | SIMILAR TO RIKEN CDNA 2510042P03 GENE homolog [Mus musculus] | 1.36 | 0,0060 |
| A\_51\_P290556 | AK034747 | CDNA FLJ30664 FIS, CLONE FCBBF1000604, WEAKLY SIMILAR TO MYOSIN HEAVY CHAIN MYR 8B homolog [Homo sapiens] | 1.36 | 0,0048 |
| A\_51\_P324838 | AK007353 | Mus musculus envoplakin (Evpl), mRNA | 1.36 | 0,0408 |
| A\_51\_P145171 | NM\_133729 | Mus musculus RIKEN cDNA 2610018G03 gene (2610018G03Rik), mRNA | 1.36 | 0,0019 |
| A\_51\_P227232 | NM\_147025 | Mus musculus olfactory receptor MOR135-1 (MOR135-1), mRNA | 1.35 | 0,0014 |
| A\_51\_P142343 | NM\_178912 | hypothetical ERCC4 domain containing protein | 1.35 | 0,0066 |
| A\_51\_P185693 | NM\_031197 | Mus musculus solute carrier family 2 (facilitated glucose transporter), member 2 (Slc2a2), mRNA | 1.35 | 0,0040 |
| A\_51\_P212064 | NM\_029638 | Mus musculus, Similar to amiloride binding protein, clone MGC:28601 IMAGE:4216952, mRNA, complete cds | 1.35 | 0,0089 |
| A\_51\_P458140 | NM\_177137 | GUANINE NUCLEOTIDE-BINDING PROTEIN G(OLF), ALPHA SUBUNIT (ADENYLATE CYCLASE-STIMULATING G ALPHA PROTEIN, OLFACTORY TYPE) homolog [Homo sapiens] | 1.35 | 0,0078 |
| A\_51\_P339055 | NM\_145469 | SIMILAR TO HYPOTHETICAL PROTEIN FLJ13955 homolog [Mus musculus] | 1.35 | 0,0103 |
| A\_51\_P362104 | NM\_032003 | Mus musculus ectonucleotide pyrophosphatase/phosphodiesterase 5 (Enpp5), mRNA | 1.35 | 0,0435 |
| A\_51\_P464158 | NM\_025480 | Mus musculus RIKEN cDNA 2810021O14 gene (2810021O14Rik), mRNA | 1.35 | 0,0262 |
| A\_51\_P237106 | NM\_001011804 | Mus musculus olfactory receptor MOR233-16P (MOR233-16P) pseudogene | 1.35 | 0,0046 |
| A\_51\_P483839 | NM\_016706 | Mus musculus coilin (Coil), mRNA | 1.35 | 0,0411 |
| A\_51\_P415413 | NM\_021892 | Mus musculus RFamide-related peptide (Rfrp), mRNA | 1.35 | 0,0143 |
| A\_51\_P333034 | NM\_025409 | Mus musculus RIKEN cDNA 1110057H19 gene (1110057H19Rik), mRNA | 1.35 | 0,0078 |
| A\_51\_P222973 | NM\_138949 | Mus musculus, similar to zinc finger protein 135 (clone pHZ-17), clone MGC:39058 IMAGE:5365187, mRNA, complete cds | 1.35 | 0,0056 |
| A\_51\_P297865 | NM\_027782 | hypothetical BTB/POZ domain/K+ channel tetramerisation domain containing protein | 1.35 | 0,0117 |
| A\_51\_P431785 | NM\_008664 | Mus musculus myomesin 2 (Myom2), mRNA | 1.35 | 0,0387 |
| A\_51\_P393863 | NM\_025716 | Mus musculus, RIKEN cDNA 4633402N23 gene, clone MGC:25683 IMAGE:4923277, mRNA, complete cds | 1.35 | 0,0454 |
| A\_51\_P205480 | NM\_144843 | MYOTUBULARIN RELATED PROTEIN 6 homolog [Homo sapiens] | 1.35 | 0,0273 |
| A\_51\_P156511 | NAP057146-1 | Mus musculus olfactory receptor GA\_x5J8B7W3DQU-679632-679375 (GA\_x5J8B7W3DQU-679632-679375) pseudogene | 1.35 | 0,0294 |
| A\_51\_P361788 | NM\_013933 | Mus musculus vesicle-associated membrane protein, associated protein A (33 kDa) (Vapa), mRNA | 1.35 | 0,0094 |
| A\_51\_P188073 | NM\_026400 | Mus musculus DnaJ (Hsp40) homolog, subfamily B, member 11 (Dnajb11), mRNA | 1.35 | 0,0280 |
| A\_51\_P228175 | XM\_486612 | hypothetical WW / rsp5 / WWP domain containing protein | 1.35 | 0,0052 |
| A\_51\_P304769 | XM\_287445 | Mus musculus antigen containing epitope to monoclonal antibody MMS-85/12 mRNA, partial cds | 1.35 | 0,0431 |
| A\_51\_P378341 | AK016780 | hypothetical protein | 1.35 | 0,0487 |
| A\_51\_P135004 | NM\_145576 | Similar to: Mus musculus Zinc finger protein 212 (Znf212), mRNA | 1.35 | 0,0281 |
| A\_51\_P128304 | NM\_146570 | Mus musculus olfactory receptor GA\_x5J8B7W2F7R-2521-3120 (GA\_x5J8B7W2F7R-2521-3120) pseudogene | 1.35 | 0,0119 |
| A\_51\_P458584 | NM\_008040 | Mus musculus formyl peptide receptor, related sequence 3 (Fpr-rs3), mRNA | 1.35 | 0,0065 |
| A\_51\_P205965 | NM\_026912 | SORTING NEXIN | 1.35 | 0,0131 |
| A\_51\_P370720 | NM\_024290 | Mus musculus tumor necrosis factor receptor superfamily, member 23 (Tnfrsf23), mRNA | 1.35 | 0,0033 |
| A\_51\_P185563 | NM\_145742 | PROBABLE ATP-DEPENDENT HELICASE DDX35 (DEAH-BOX PROTEIN 35) homolog [Homo sapiens] | 1.35 | 0,0042 |
| A\_51\_P469739 | NM\_146797 | Mus musculus olfactory receptor MOR211-4P (MOR211-4P) pseudogene | 1.35 | 0,0491 |
| A\_51\_P245468 | AI838397 | Mus musculus cDNA, 3 end | 1.35 | 0,0106 |
| A\_51\_P361443 | AK028741 | similar to HYPOTHETICAL 21.1 KDA PROTEIN [Homo sapiens] | 1.35 | 0,0192 |
| A\_51\_P390744 | NM\_146474 | Mus musculus olfactory receptor MOR234-3 (MOR234-3), mRNA | 1.35 | 0,0285 |
| A\_51\_P486939 | NM\_133710 | Mus musculus RIKEN cDNA 2810418J22 gene (2810418J22Rik), mRNA | 1.35 | 0,0248 |
| A\_51\_P349023 | NM\_026309 | Mus musculus RIKEN cDNA 1010001J12 gene (1010001J12Rik), mRNA | 1.34 | 0,0413 |
| A\_51\_P401924 | NAP057158-1 | Mus musculus olfactory receptor MOR136-18P (MOR136-18P) pseudogene | 1.34 | 0,0068 |
| A\_51\_P150845 | NM\_183289 | hypothetical WW/rsp5/WWP domain profile/WW / rsp5 / WWP domain containing protein | 1.34 | 0,0200 |
| A\_51\_P159213 | A\_51\_P159213 | Mus musculus cDNA, 3 end | 1.34 | 0,0085 |
| A\_51\_P317317 | NM\_172116 | hypothetical ThiJ/PfpI family containing protein | 1.34 | 0,0256 |
| A\_51\_P369508 | NM\_025784 | BCS1-like (yeast) | 1.34 | 0,0426 |
| A\_51\_P114002 | AK002213 | Mus musculus RIKEN cDNA 0610005A07 gene (0610005A07Rik), mRNA | 1.34 | 0,0458 |
| A\_51\_P210433 | NM\_146486 | Mus musculus olfactory receptor MOR182-5 (MOR182-5), mRNA | 1.34 | 0,0090 |
| A\_51\_P306160 | AK051378 | similar to LEUCINE ZIPPER BEARING KINASE [Homo sapiens] | 1.34 | 0,0089 |
| A\_51\_P102215 | NM\_001011777 | Mus musculus olfactory receptor MOR185-10 (MOR185-10) pseudogene | 1.34 | 0,0064 |
| A\_51\_P259584 | XM\_148595 | LIMKAIN B1 homolog [Rattus norvegicus] | 1.34 | 0,0070 |
| A\_51\_P366079 | NM\_146391 | Mus musculus olfactory receptor MOR190-3P (MOR190-3P) pseudogene | 1.34 | 0,0111 |
| A\_51\_P427156 | AI839636 | Mus musculus cDNA, 3 end | 1.34 | 0,0215 |
| A\_51\_P445153 | NM\_011897 | Mus musculus sprouty homolog 2 (Drosophila) (Spry2), mRNA | 1.34 | 0,0441 |
| A\_51\_P512997 | NM\_178676 | ectonucleoside triphosphate diphosphohydrolase 3 | 1.34 | 0,0254 |
| A\_51\_P135532 | AK021182 | hypothetical protein | 1.34 | 0,0085 |
| A\_51\_P457397 | AK009140 | unknown EST | 1.34 | 0,0258 |
| A\_51\_P419078 | BC006684 | Mus musculus RIKEN cDNA 4833432B22 gene (4833432B22Rik), mRNA | 1.34 | 0,0215 |
| A\_51\_P274506 | NM\_011573 | testis expressed gene 264 | 1.34 | 0,0063 |
| A\_51\_P445006 | NM\_172490 | Mus musculus hypothetical protein 9130208G10 (9130208G10), mRNA | 1.34 | 0,0198 |
| A\_51\_P272414 | NM\_146762 | Mus musculus olfactory receptor MOR213-2 (MOR213-2), mRNA | 1.34 | 0,0151 |
| A\_51\_P125395 | NM\_010435 | Mus musculus histone cell cycle regulation defective homolog A (S. cerevisiae) (Hira), mRNA | 1.34 | 0,0377 |
| A\_51\_P161812 | NM\_010100 | Mus musculus ectodysplasin-A receptor (Edar), mRNA | 1.34 | 0,0294 |
| A\_51\_P276203 | AK087873 | unclassifiable | 1.34 | 0,0062 |
| A\_51\_P518488 | NM\_007512 | Mus musculus ATPase inhibitor (Atpi), mRNA | 1.34 | 0,0266 |
| A\_51\_P376857 | NM\_029585 | hypothetical protein | 1.34 | 0,0114 |
| A\_51\_P157737 | NM\_172483 | similar to CYS2/HIS2 ZINC FINGER PROTEIN [Rattus norvegicus] | 1.34 | 0,0434 |
| A\_51\_P270213 | NM\_023289 | Mus musculus CEA-related cell adhesion molecule 11 (Ceacam11), mRNA | 1.34 | 0,0332 |
| A\_51\_P229915 | BC013546 | Mus musculus, clone MGC:19331 IMAGE:4210417, mRNA, complete cds | 1.34 | 0,0394 |
| A\_51\_P441837 | NM\_026837 | hypothetical protein | 1.34 | 0,0135 |
| A\_51\_P354857 | NM\_134253 | Mus musculus hypothetical protein MGC8103 (MGC8103), mRNA | 1.34 | 0,0107 |
| A\_51\_P204772 | AK043188 | unclassifiable | 1.33 | 0,0482 |
| A\_51\_P256305 | NM\_025500 | mitochondrial ribosomal protein L37 | 1.33 | 0,0078 |
| A\_51\_P171486 | AK049540 | unclassifiable | 1.33 | 0,0062 |
| A\_51\_P500152 | NM\_145505 | hypothetical protein | 1.33 | 0,0353 |
| A\_51\_P116687 | NM\_025851 | Mus musculus RIKEN cDNA 1700010I14 gene (1700010I14Rik), mRNA | 1.33 | 0,0108 |
| A\_51\_P289279 | NM\_147042 | Mus musculus clone OR55-36 putative olfactory receptor mRNA, partial cds | 1.33 | 0,0197 |
| A\_51\_P378777 | NM\_029090 | Mus musculus, RIKEN cDNA 1200013P24 gene, clone MGC:6893 IMAGE:2654387, mRNA, complete cds | 1.33 | 0,0245 |
| A\_51\_P336443 | AK036326 | weakly similar to reverse transcriptase-like protein (fragment) [Rattus norvegicus] | 1.33 | 0,0275 |
| A\_51\_P107520 | NM\_181818 | Mus musculus olfactory receptor MOR179-5 (MOR179-5) pseudogene | 1.33 | 0,0041 |
| A\_51\_P159641 | NM\_146486 | Mus musculus olfactory receptor MOR182-5 (MOR182-5), mRNA | 1.33 | 0,0221 |
| A\_51\_P509881 | NM\_153798 | Mus musculus, polymerase (RNA) II (DNA directed) polypeptide B (140kD), clone IMAGE:3158185, mRNA | 1.33 | 0,0402 |
| A\_51\_P425175 | AK089738 | unknown EST | 1.33 | 0,0071 |
| A\_51\_P170565 | NM\_177880 | hypothetical protein | 1.33 | 0,0101 |
| A\_51\_P178894 | NM\_183199 | hypothetical Zn-finger in ubiquitin-hydrolases and other proteins containing protein | 1.33 | 0,0099 |
| A\_51\_P334459 | NM\_001002789 | hypothetical Vacuolar sorting protein 9 (VPS9) domain containing protein | 1.33 | 0,0034 |
| A\_51\_P114105 | BC052872 | MYOPALLADIN homolog [Homo sapiens] | 1.33 | 0,0050 |
| A\_51\_P310699 | NM\_178707 | HYPOTHETICAL ZINC FINGER PROTEIN KIAA0211 homolog [Homo sapiens] | 1.33 | 0,0298 |
| A\_51\_P423813 | XM\_129951 | similar to ALKALINE PHOSPHATASE (FRAGMENT) [Homo sapiens] | 1.33 | 0,0498 |
| A\_51\_P150722 | NM\_010748 | Mus musculus lysosomal trafficking regulator (Lyst), mRNA | 1.33 | 0,0200 |
| A\_51\_P385030 | NM\_013679 | Mus musculus seminal vesicle secretion 6 (Svs6), mRNA | 1.33 | 0,0281 |
| A\_51\_P450682 | NM\_199455 | hypothetical protein | 1.33 | 0,0197 |
| A\_51\_P351351 | AK053479 | unknown EST | 1.33 | 0,0091 |
| A\_51\_P475985 | NM\_172974 | Mus musculus COP9 (constitutive photomorphogenic) homolog, subunit 7b (Arabidopsis thaliana) (Cops7b), mRNA | 1.33 | 0,0161 |
| A\_51\_P130459 | NM\_011694 | Mus musculus voltage-dependent anion channel 1 (Vdac1), mRNA | 1.33 | 0,0245 |
| A\_51\_P269020 | AK006578 | hypothetical protein | 1.33 | 0,0079 |
| A\_51\_P428832 | AK014640 | similar to HIGH-MOBILITY GROUP PROTEIN 2-LIKE 1 (HMGBCG PROTEIN) [Homo sapiens] | 1.33 | 0,0114 |
| A\_51\_P451758 | NM\_144538 | Mus musculus, clone MGC:28059 IMAGE:3708973, mRNA, complete cds | 1.33 | 0,0159 |
| A\_51\_P514647 | NM\_026420 | Mus musculus RIKEN cDNA 2310050K10 gene (2310050K10Rik), mRNA | 1.33 | 0,0361 |
| A\_51\_P304354 | A\_51\_P304354 | Mus musculus cDNA, 3 end | 1.33 | 0,0163 |
| A\_51\_P502133 | NM\_011985 | Mus musculus matrix metalloproteinase 23 (Mmp23), mRNA | 1.33 | 0,0214 |
| A\_51\_P391524 | NM\_023598 | Mus musculus developmentally and sexually retarded with transient immune abnormalities (Desrt), mRNA | 1.32 | 0,0484 |
| A\_51\_P124638 | NAP108490-1 | Mus musculus olfactory receptor GA\_x5J8B7W5KGR-202501-203873 (GA\_x5J8B7W5KGR-202501-203873) pseudogene | 1.32 | 0,0169 |
| A\_51\_P161200 | AK030281 | hypothetical Eukaryotic thiol (cysteine) proteases active site/Trp-Asp (WD) repeats circular profile/G-protein beta WD-40 repeats containing protein | 1.32 | 0,0306 |
| A\_51\_P320606 | NM\_023746 | Mus musculus RIKEN cDNA 1600013P04 gene (1600013P04Rik), mRNA | 1.32 | 0,0059 |
| A\_51\_P355981 | NM\_027844 | Mus musculus RIKEN cDNA 4833428E21 gene (4833428E21Rik), mRNA | 1.32 | 0,0089 |
| A\_51\_P288719 | NM\_146031 | Mus musculus, clone MGC:6956 IMAGE:3153901, mRNA, complete cds | 1.32 | 0,0037 |
| A\_51\_P174505 | NM\_146336 | Mus musculus olfactory receptor MOR170-11 (MOR170-11), mRNA | 1.32 | 0,0025 |
| A\_51\_P398118 | NM\_134218 | Mus musculus vomeronasal 1 receptor, H9 (V1rh9), mRNA | 1.32 | 0,0290 |
| A\_51\_P394676 | BC040217 | unknown EST | 1.32 | 0,0487 |
| A\_51\_P519811 | NM\_018751 | Mus musculus sulfotransferase family 1A, phenol-preferring, member 2 (Sult1a2), mRNA | 1.32 | 0,0067 |
| A\_51\_P356366 | NM\_177190 | SIMILAR TO TESTIS-SPECIFIC PROTEIN PBS13) homolog [Homo sapiens] | 1.32 | 0,0032 |
| A\_51\_P333594 | A\_51\_P333594 | Mus musculus cDNA, 3 end | 1.32 | 0,0190 |
| A\_51\_P286327 | NM\_175646 | hypothetical Pre-mRNA splicing protein containing protein | 1.32 | 0,0196 |
| A\_51\_P211923 | NM\_177586 | EUKARYOTIC TRANSLATION INITIATION FACTOR 5AII (EIF-5A2) homolog [Homo sapiens] | 1.32 | 0,0135 |
| A\_51\_P315993 | AK080082 | hypothetical protein | 1.32 | 0,0050 |
| A\_51\_P478748 | NM\_175647 | Mus musculus doublesex and mab-3 related transcription factor 4 (Dmrt4), mRNA | 1.32 | 0,0065 |
| A\_51\_P271160 | NM\_147068 | Mus musculus olfactory receptor MOR270-1 (MOR270-1), mRNA | 1.32 | 0,0325 |
| A\_51\_P291749 | NM\_023523 | Mus musculus peroxisomal trans-2-enoyl-CoA reductase (Pecr), mRNA | 1.32 | 0,0194 |
| A\_51\_P304743 | XM\_136692 | ATP-BINDING CASSETTE TRANSPORTER FAMILY A MEMBER 12 homolog [Homo sapiens] | 1.32 | 0,0076 |
| A\_51\_P441687 | NM\_146242 | Mus musculus putative leucine-rich repeat protein LOC237560 (LOC237560), mRNA | 1.32 | 0,0203 |
| A\_51\_P252519 | NM\_053269 | Mus musculus Rad51 homolog c (S. cerevisiae) (Rad51c), mRNA | 1.32 | 0,0039 |
| A\_51\_P321698 | NM\_021534 | Mus musculus peroxisomal membrane protein 4 (Pxmp4), mRNA | 1.32 | 0,0361 |
| A\_51\_P487788 | AK080276 | BETA-2 ADRENERGIC RECEPTOR | 1.32 | 0,0131 |
| A\_51\_P503719 | NM\_013554 | Mus musculus homeo box D10 (Hoxd10), mRNA | 1.32 | 0,0027 |
| A\_51\_P120027 | AK004863 | similar to ALCOHOL DEHYDROGENASE 2 (EC 1.1.1.1) [Peromyscus maniculatus] | 1.32 | 0,0029 |
| A\_51\_P140811 | BC069869 | hypothetical G-protein beta WD-40 repeats containing protein | 1.32 | 0,0279 |
| A\_51\_P230579 | AK007309 | 1700128E19RIK PROTEIN homolog [Mus musculus] | 1.32 | 0,0043 |
| A\_51\_P301549 | AK220293 | CDNA FLJ20302 FIS, CLONE HEP06648 homolog [Homo sapiens] | 1.32 | 0,0151 |
| A\_51\_P362399 | NM\_176953 | DNA LIGASE IV (EC 6.5.1.1) (POLYDEOXYRIBONUCLEOTIDE SYNTHASE [ATP]) homolog [Homo sapiens] | 1.32 | 0,0301 |
| A\_51\_P471921 | A\_51\_P471921 | Mus musculus cDNA, 5 end | 1.32 | 0,0082 |
| A\_51\_P386562 | NM\_010095 | Mus musculus early B-cell factor 2 (Ebf2), mRNA | 1.32 | 0,0045 |
| A\_51\_P501315 | AK004720 | Mus musculus gene trap locus F3b (Gtlf3b), mRNA | 1.32 | 0,0400 |
| A\_51\_P146837 | AK044171 | similar to IONOTROPIC GLUTAMATE RECEPTOR NMDA subunit NR3A[Rattus norvegicus] | 1.32 | 0,0061 |
| A\_51\_P252819 | NM\_001011869 | Mus musculus olfactory receptor MOR257-5P (MOR257-5P) pseudogene | 1.31 | 0,0139 |
| A\_51\_P248644 | BC065162 | TYROSYL-DNA PHOSPHODIESTERASE PROTEIN (FRAGMENT) homolog [Homo sapiens] | 1.31 | 0,0377 |
| A\_51\_P460929 | NM\_153176 | Mus musculus paraplegin (Spg7), mRNA | 1.31 | 0,0283 |
| A\_51\_P480046 | NM\_007647 | Mus musculus ectonucleoside triphosphate diphosphohydrolase 5 (Entpd5), mRNA | 1.31 | 0,0189 |
| A\_51\_P402416 | NM\_025504 | Mus musculus RIKEN cDNA 2310004L02 gene (2310004L02Rik), mRNA | 1.31 | 0,0300 |
| A\_51\_P484842 | NM\_007493 | Mus musculus asialoglycoprotein receptor 2 (Asgr2), mRNA | 1.31 | 0,0109 |
| A\_51\_P516268 | AK083404 | unknown EST | 1.31 | 0,0232 |
| A\_51\_P174244 | AI462521 | Mus musculus cDNA, 3 end | 1.31 | 0,0106 |
| A\_51\_P516955 | A\_51\_P516955 | Mus musculus cDNA, 3 end | 1.31 | 0,0251 |
| A\_51\_P308148 | NM\_134208 | Mus musculus vomeronasal 1 receptor, G7 (V1rg7), mRNA | 1.31 | 0,0040 |
| A\_51\_P346614 | BC024687 | "Mus musculus, similar to loop tail associated protein� vang (van gogh)-like 2 (Drosophila)� loop tail, clone IMAGE:3669388, mRNA, partial cds" | 1.31 | 0,0339 |
| A\_51\_P412579 | AK010233 | unknown EST | 1.31 | 0,0312 |
| A\_51\_P271644 | NM\_011246 | Mus musculus RAS guanyl releasing protein 1 (Rasgrp1), mRNA | 1.31 | 0,0314 |
| A\_51\_P360262 | NM\_053122 | Mus musculus inner mitochondrial membrane peptidase 2-like (S. cerevisiae) (Immp2l-pending), mRNA | 1.31 | 0,0217 |
| A\_51\_P438083 | NM\_144512 | Mus musculus, Similar to solute carrier family 6 (neurotransmitter transporter, GABA), member 13, clone MGC:28956 IMAGE:4240641, mRNA, complete cds | 1.31 | 0,0469 |
| A\_51\_P329795 | NM\_145362 | Mus musculus, Similar to beta-1,4 mannosyltransferase, clone MGC:18946 IMAGE:3980821, mRNA, complete cds | 1.31 | 0,0373 |
| A\_51\_P488028 | NAP057094-1 | Mus musculus olfactory receptor GA\_x5J8B7W62NC-668773-668438 (GA\_x5J8B7W62NC-668773-668438) pseudogene | 1.31 | 0,0031 |
| A\_51\_P199475 | NM\_145581 | SIALIC ACID-BINDING LECTIN SIGLEC-F homolog [Mus musculus] | 1.31 | 0,0271 |
| A\_51\_P427132 | NM\_177076 | hypothetical F-box domain containing protein | 1.31 | 0,0153 |
| A\_51\_P329508 | NM\_001013392 | Mus musculus, Similar to ras responsive element binding protein 1, clone IMAGE:5321450, mRNA | 1.31 | 0,0198 |
| A\_51\_P506843 | NM\_009504 | Mus musculus vitamin D receptor (Vdr), mRNA | 1.31 | 0,0126 |
| A\_51\_P211064 | NM\_020290 | Mus musculus odorant receptor S18 gene (Ors18), mRNA | 1.31 | 0,0036 |
| A\_51\_P187093 | NM\_010270 | Mus musculus mRNA for ganglioside-induced differentiation associated protein 3 | 1.31 | 0,0409 |
| A\_51\_P363137 | NM\_030147 | THYROID HORMONE RECEPTOR COACTIVATING PROTEIN homolog [Homo sapiens] | 1.31 | 0,0237 |
| A\_51\_P437717 | NM\_020003 | Mus musculus RIKEN cDNA 0610031J06 gene (0610031J06Rik), mRNA | 1.31 | 0,0477 |
| A\_51\_P289351 | NM\_032544 | Mus musculus RIKEN cDNA 2410009F13 gene (2410009F13Rik), mRNA | 1.31 | 0,0158 |
| A\_51\_P419750 | AK051383 | unknown EST | 1.31 | 0,0392 |
| A\_51\_P237879 | NM\_027671 | Mus musculus syntrophin, gamma 1 (Sntg1), mRNA | 1.31 | 0,0063 |
| A\_51\_P360728 | NAP057028-1 | Mus musculus ubiquitin-activating enzyme E1, Chr Y-1, pseudogene 2 (Ube1y1-ps2) on chromosome Y | 1.31 | 0,0162 |
| A\_51\_P185713 | NM\_013782 | Mus musculus phosphatidylserine synthase 2 (Ptdss2), mRNA | 1.30 | 0,0220 |
| A\_51\_P370155 | NM\_172539 | hypothetical Neutral zinc metallopeptidases, zinc-binding region/Astacin (Peptidase family M12A) family containing protein | 1.30 | 0,0285 |
| A\_51\_P346045 | NM\_053266 | Mus musculus RIKEN cDNA 1700012P16 gene (1700012P16Rik), mRNA | 1.30 | 0,0392 |
| A\_51\_P210021 | NM\_028735 | hypothetical Tetratricopeptide repeat (TPR) structure containing protein | 1.30 | 0,0114 |
| A\_51\_P303286 | NM\_153108 | Mus musculus mRNA for beta-defensin 8 | 1.30 | 0,0266 |
| A\_51\_P229230 | AK005637 | hypothetical Immunoglobulin and major histocompatibility complex domain containing protein | 1.30 | 0,0288 |
| A\_51\_P511329 | NM\_008966 | prostaglandin F receptor | 1.30 | 0,0108 |
| A\_51\_P490618 | NM\_146641 | Mus musculus olfactory receptor MOR174-11 (MOR174-11), mRNA | 1.30 | 0,0109 |
| A\_51\_P306770 | AK018199 | hypothetical protein | 1.30 | 0,0086 |
| A\_51\_P122507 | NM\_146032 | SIGNAL RECOGNITION PARTICLE 68 KDA PROTEIN (SRP68) homolog [Canis familiaris] | 1.30 | 0,0413 |
| A\_51\_P327890 | AK033757 | weakly similar to HYDROXYPROLINE-RICH GLYCOPROTEIN DZ-HRGP PRECURSOR [Volvox carteri f. nagariensis] | 1.30 | 0,0289 |
| A\_51\_P317887 | NM\_026907 | Mus musculus secreted and transmembrane 1 (Sectm1), mRNA | 1.30 | 0,0097 |
| A\_51\_P377302 | NM\_170599 | hypothetical Immunoglobulin subtype containing protein | 1.30 | 0,0069 |
| A\_51\_P145010 | AK010095 | weakly similar to COLLAGEN-LIKE PROTEIN [Herpesvirus saimiri] | 1.30 | 0,0168 |
| A\_51\_P394552 | NM\_011645 | Mus musculus trypsin 3 (Try3), mRNA | 1.30 | 0,0267 |
| A\_51\_P358825 | NM\_175532 | PAAD and NACHT containing protein | 1.30 | 0,0073 |
| A\_51\_P107140 | NM\_016880 | Mus musculus keratin complex 1, acidic, gene 24 (Krt1-24), mRNA | 1.30 | 0,0172 |
| A\_51\_P123077 | NM\_011955 | Mus musculus nucleotide binding protein 1 (Nubp1), mRNA | 1.30 | 0,0312 |
| A\_51\_P211757 | A\_51\_P211757 | Mus musculus cDNA, 3 end | 1.30 | 0,0288 |
| A\_51\_P257951 | NM\_020509 | Mus musculus resistin like alpha (Retnla), mRNA | 1.30 | 0,0333 |
| A\_51\_P204153 | NM\_010518 | Mus musculus insulin-like growth factor binding protein 5 (Igfbp5), mRNA | 1.30 | 0,0084 |
| A\_51\_P156740 | NM\_011851 | Mus musculus 5 nucleotidase, ecto (Nt5e), mRNA | 1.30 | 0,0111 |
| A\_51\_P170536 | NM\_170671 | inferred: hypothetical protein {Homo sapiens} | 1.30 | 0,0106 |
| A\_51\_P133072 | A\_51\_P133072 | Mus musculus cDNA, 5 end | 1.30 | 0,0042 |
| A\_51\_P431510 | BC025128 | Mus musculus, Similar to RIKEN cDNA 1700008D07 gene, clone MGC:36853 IMAGE:4225298, mRNA, complete cds | 1.30 | 0,0414 |
| A\_51\_P207820 | NM\_008270 | Mouse Hox-2.5 mRNA | 1.30 | 0,0348 |
| A\_51\_P222324 | NM\_177078 | BETA-ADRENERGIC RECEPTOR KINASE 2 (EC 2.7.1.126) (BETA-ARK-2) (G-PROTEIN COUPLED RECEPTOR KINASE 3) homolog [Rattus norvegicus] | 1.30 | 0,0322 |
| A\_51\_P381811 | AK018844 | unclassifiable | 1.30 | 0,0241 |
| A\_51\_P198694 | NM\_144909 | Mus musculus, Similar to Glucokinase regulatory protein, clone MGC:19300 IMAGE:4159892, mRNA, complete cds | 1.30 | 0,0284 |
| A\_51\_P183723 | XM\_283635 | weakly similar to SQUAMOUS CELL CARCINOMA ANTIGEN RECOGNIZED BY T CELL [Homo sapiens] | 1.30 | 0,0144 |
| A\_51\_P293278 | U46151 | Mus musculus male meiosis specific protein Tcte2 (Tcte2) mRNA, complete cds | 1.30 | 0,0093 |
| A\_51\_P490509 | NM\_009773 | Mus musculus budding uninhibited by benzimidazoles 1 homolog, beta (S. cerevisiae) (Bub1b), mRNA | 1.30 | 0,0216 |
| A\_51\_P358700 | NM\_146902 | Mus musculus olfactory receptor MOR233-3 (MOR233-3), mRNA | 1.30 | 0,0338 |
| A\_51\_P496551 | NM\_009176 | Mus musculus sialyltransferase 6 (N-acetyllacosaminide alpha 2,3-sialyltransferase) (Siat6), mRNA | 1.30 | 0,0152 |
| A\_51\_P161043 | AK220167 | Mus musculus heat shock 70 kDa protein 4 (Hspa4), mRNA | 1.30 | 0,0275 |
| A\_51\_P141630 | NM\_173432 | PROTEIN SERINE KINASE PSKH1 homolog [Mus musculus] | 1.30 | 0,0252 |
| A\_51\_P108800 | AK051614 | similar to PLEXIN 2 [Mus musculus] | 1.30 | 0,0450 |
| A\_51\_P200107 | AK002667 | hypothetical Microbodies C-terminal targeting signal containing protein | 1.30 | 0,0285 |
| A\_51\_P400366 | NM\_021375 | Mus musculus Rhesus blood group-associated B glycoprotein (Rhbg), mRNA | 1.30 | 0,0293 |
| A\_51\_P485542 | NM\_018737 | Mus musculus cytidine 5-triphosphate synthase 2 (Ctps2), mRNA | 1.30 | 0,0072 |
| A\_51\_P325514 | XM\_130308 | Mus musculus cDNA clone IMAGE:4972734 5 | 1.29 | 0,0126 |
| A\_51\_P291860 | ENSMUST00000071587 | Mus musculus olfactory receptor MOR266-3 (MOR266-3), mRNA | 1.29 | 0,0195 |
| A\_51\_P480598 | NM\_009363 | Mus musculus trefoil factor 2 (spasmolytic protein 1) (Tff2), mRNA | 1.29 | 0,0116 |
| A\_51\_P477522 | NM\_010420 | Mus musculus homeo box gene expressed in ES cells (Hesx1), mRNA | 1.29 | 0,0303 |
| A\_51\_P183401 | NM\_146851 | Mus musculus olfactory receptor MOR220-1 (MOR220-1), mRNA | 1.29 | 0,0117 |
| A\_51\_P488068 | ENSMUST00000087819 | Mus musculus olfactory receptor MOR202-32P (MOR202-32P) pseudogene | 1.29 | 0,0163 |
| A\_51\_P492346 | NM\_026685 | hypothetical protein | 1.29 | 0,0294 |
| A\_51\_P422666 | AK054167 | unknown EST | 1.29 | 0,0271 |
| A\_51\_P483180 | BC087942 | SORTING NEXIN 7 | 1.29 | 0,0340 |
| A\_51\_P166568 | AK046297 | GLUTAMINASE, KIDNEY ISOFORM, MITOCHONDRIAL PRECURSOR (EC 3.5.1.2) (GLS) (L-GLUTAMINE AMIDOHYDROLASE) (K-GLUTAMINASE) homolog [Homo sapiens] | 1.29 | 0,0361 |
| A\_51\_P439413 | NAP057235-1 | Mus musculus olfactory receptor MOR234-4P (MOR234-4P) pseudogene | 1.29 | 0,0127 |
| A\_51\_P258493 | NM\_011067 | Mus musculus period homolog 3 (Drosophila) (Per3), mRNA | 1.29 | 0,0266 |
| A\_51\_P519791 | NM\_198605 | hypothetical protein | 1.29 | 0,0251 |
| A\_51\_P197277 | NM\_147008 | Mus musculus olfactory receptor MOR135-7 (MOR135-7), mRNA | 1.29 | 0,0183 |
| A\_51\_P329501 | AK037051 | MYOSIN-1D | 1.29 | 0,0101 |
| A\_51\_P345643 | NM\_009336 | Mus musculus transcription factor-like 1 (Tcfl1), mRNA | 1.29 | 0,0242 |
| A\_51\_P243418 | NM\_027562 | Mus musculus RIKEN cDNA 4632413B12 gene (4632413B12Rik), mRNA | 1.29 | 0,0209 |
| A\_51\_P452702 | AK006382 | hypothetical protein | 1.29 | 0,0422 |
| A\_51\_P259201 | NM\_146350 | Mus musculus olfactory receptor MOR264-17 (MOR264-17), mRNA | 1.29 | 0,0305 |
| A\_51\_P281855 | NM\_028152 | Mus musculus MMS19 (MET18 S. cerevisiae)-like (Mms19l), mRNA | 1.29 | 0,0469 |
| A\_51\_P140501 | A\_51\_P140501 | SELENIDE,WATER DIKINASE EC 2.7.9.3 SELENOPHOSPHATE SYNTHETASE SELENIUM DONOR PROTEIN | 1.29 | 0,0154 |
| A\_51\_P392604 | NM\_001011864 | Mus musculus olfactory receptor MOR168-2P (MOR168-2P) pseudogene | 1.29 | 0,0061 |
| A\_51\_P351286 | NM\_008794 | Mus musculus proprotein convertase subtilisin/kexin type 7 (Pcsk7), mRNA | 1.29 | 0,0235 |
| A\_51\_P300745 | NM\_001005227 | Mus musculus olfactory receptor MOR225-12 (MOR225-12) pseudogene | 1.29 | 0,0230 |
| A\_51\_P175442 | NM\_173024 | weakly similar to SPI2 PROTEINASE INHIBITOR [Mus musculus] | 1.29 | 0,0410 |
| A\_51\_P137971 | NM\_011696 | Mus musculus voltage-dependent anion channel 3 (Vdac3), mRNA | 1.29 | 0,0182 |
| A\_51\_P459770 | AK048388 | REVERSE TRANSCRIPTASE homolog [Mus musculus] | 1.29 | 0,0063 |
| A\_51\_P337935 | AK008024 | LARGE NEUTRAL AMINO ACIDS TRANSPORTER SMALL SUBUNIT L TYPE AMINO ACID TRANSPORTER | 1.29 | 0,0280 |
| A\_51\_P515937 | NM\_144908 | Mus musculus UDP-GalNAc:polypeptide N-acetylgalactosaminyltransferase (Galnt11), mRNA | 1.29 | 0,0437 |
| A\_51\_P142350 | AK016986 | unknown EST | 1.29 | 0,0062 |
| A\_51\_P353569 | NM\_001011837 | OLFACTORY RECEPTOR | 1.29 | 0,0206 |
| A\_51\_P160272 | AK032445 | unclassifiable | 1.29 | 0,0219 |
| A\_51\_P446417 | NM\_026194 | hypothetical protein | 1.29 | 0,0265 |
| A\_51\_P446366 | NM\_031380 | Mus musculus follistatin-like 3 (Fstl3), mRNA | 1.29 | 0,0088 |
| A\_51\_P127949 | AK042690 | TRANSCRIPTION FACTOR NRF homolog [Homo sapiens] | 1.29 | 0,0376 |
| A\_51\_P517012 | AK005069 | hypothetical protein | 1.29 | 0,0369 |
| A\_51\_P262410 | AK014745 | hypothetical protein | 1.29 | 0,0299 |
| A\_51\_P354419 | NM\_146995 | Mus musculus olfactory receptor MOR182-1 (MOR182-1), mRNA | 1.29 | 0,0266 |
| A\_51\_P403243 | NM\_029779 | hypothetical protein | 1.28 | 0,0388 |
| A\_51\_P371573 | NM\_026287 | Mus musculus RIKEN cDNA 4930470P17 gene (4930470P17Rik), mRNA | 1.28 | 0,0344 |
| A\_51\_P387388 | NM\_146279 | Mus musculus olfactory receptor MOR224-12 (MOR224-12), mRNA | 1.28 | 0,0186 |
| A\_51\_P118230 | NM\_028078 | Mus musculus, RIKEN cDNA 2010003D20 gene, clone MGC:7960 IMAGE:3584645, mRNA, complete cds | 1.28 | 0,0092 |
| A\_51\_P429295 | NM\_028304 | hypothetical ATP/GTP-binding site motif A (P-loop) containing protein | 1.28 | 0,0409 |
| A\_51\_P437516 | NAP057202-1 | Mus musculus olfactory receptor MOR171-36P (MOR171-36P) pseudogene | 1.28 | 0,0050 |
| A\_51\_P515206 | NM\_011350 | Mus musculus sema domain, immunoglobulin domain (Ig), TM domain, and short cytoplasmic domain (Sema4f), mRNA | 1.28 | 0,0280 |
| A\_51\_P484261 | NM\_011468 | Mus musculus small proline-rich protein 2A (Sprr2a), mRNA | 1.28 | 0,0231 |
| A\_51\_P115374 | AK045286 | hypothetical Nucleic acid-binding proteins structure containing protein | 1.28 | 0,0185 |
| A\_51\_P362423 | NM\_010841 | Mus musculus metallothionein-like 5, testis-specific (tesmin) (Mtl5), mRNA | 1.28 | 0,0105 |
| A\_51\_P448447 | NM\_153092 | SIMILAR TO NUCLEOPORIN-LIKE PROTEIN 1 homolog [Homo sapiens] | 1.28 | 0,0461 |
| A\_51\_P220512 | AK014500 | Mus musculus RIKEN cDNA 4432416O06 gene (4432416O06Rik), mRNA | 1.28 | 0,0419 |
| A\_51\_P151493 | NM\_007526 | Mus musculus BarH-like homeobox 1 (Barx1), mRNA | 1.28 | 0,0238 |
| A\_51\_P329300 | AK041673 | PROTON MYO-INOSITOL TRANSPORTER homolog [Rattus norvegicus] | 1.28 | 0,0156 |
| A\_51\_P251821 | NM\_008507 | Mus musculus linker of T-cell receptor pathways (Lnk), mRNA | 1.28 | 0,0252 |
| A\_51\_P217360 | AK046418 | Mus musculus cDNA, 3 end | 1.28 | 0,0080 |
| A\_51\_P292230 | AK047481 | hypothetical Arginine-rich region containing protein | 1.28 | 0,0136 |
| A\_51\_P313729 | NM\_146334 | Mus musculus olfactory receptor MOR259-8 (MOR259-8), mRNA | 1.28 | 0,0335 |
| A\_51\_P347115 | NM\_177045 | hypothetical protein | 1.28 | 0,0167 |
| A\_51\_P350853 | NM\_009987 | Mus musculus chemokine (C-X3-C) receptor 1 (Cx3cr1), mRNA | 1.28 | 0,0411 |
| A\_51\_P235856 | NM\_145425 | HOMOLOC-13 homolog [Mus musculus] | 1.28 | 0,0459 |
| A\_51\_P411335 | NM\_146964 | Mus musculus olfactory receptor H7 mRNA, partial cds | 1.28 | 0,0043 |
| A\_51\_P258512 | AK086336 | inferred: alpha-2-macroglobulin precursor (alpha-2-M) {Rattus norvegicus} | 1.28 | 0,0439 |
| A\_51\_P345937 | NM\_134011 | Mus musculus strain BALB/c unknown mRNA | 1.28 | 0,0073 |
| A\_51\_P422495 | NM\_134216 | Mus musculus vomeronasal 1 receptor, H7 (V1rh7), mRNA | 1.28 | 0,0206 |
| A\_51\_P171347 | NM\_172937 | hypothetical SNF2 related domain/PHD-finger/DEAD/DEAH box helicase/Histone H1 and H5 family/Cytochrome c family heme-binding site containing protein | 1.28 | 0,0146 |
| A\_51\_P186374 | NM\_010815 | Mus musculus monocytic adaptor (Mona), mRNA | 1.28 | 0,0121 |
| A\_51\_P510518 | U96149 | Mus musculus fatty acid binding protein 9, testis (Fabp9), mRNA | 1.28 | 0,0206 |
| A\_51\_P515314 | NM\_172670 | GLYCOSYLTRANSFERASE LIKE PROTEIN LARGE EC 2.4.-.- ACETYLGLUCOSAMINYLTRANSFERASE LIKE | 1.28 | 0,0271 |
| A\_51\_P388640 | NM\_010816 | Mus musculus microrchidia (Morc), mRNA | 1.28 | 0,0335 |
| A\_51\_P400490 | AK041423 | hypothetical Dbl domain (dbl/cdc24 rhoGEF family) containing protein | 1.28 | 0,0271 |
| A\_51\_P402477 | AK173166 | hypothetical Esterase/lipase/thioesterase family active site containing protein | 1.28 | 0,0220 |
| A\_51\_P483130 | AK046696 | hypothetical Histidine acid phosphatase containing protein | 1.28 | 0,0104 |
| A\_51\_P233903 | NM\_178919 | similar to HYPOTHETICAL 80.1 KDA PROTEIN (FRAGMENT) [Homo sapiens] | 1.28 | 0,0380 |
| A\_51\_P176195 | AK083989 | proteasome (prosome, macropain) 26S subunit, ATPase 2 | 1.27 | 0,0442 |
| A\_51\_P310387 | NM\_020613 | Mus musculus epidymal sperm gene (X99300), mRNA | 1.27 | 0,0180 |
| A\_51\_P153581 | AK016891 | hypothetical protein | 1.27 | 0,0266 |
| A\_51\_P107782 | BC023820 | FOLLICULAR VARIANT TRANSLOCATION PROTEIN 1 PRECURSOR (FVT-1) homolog [Homo sapiens] | 1.27 | 0,0499 |
| A\_51\_P198747 | A\_51\_P198747 | Mus musculus cDNA, 3 end | 1.27 | 0,0416 |
| A\_51\_P210210 | NM\_148947 | Mus musculus lymphocyte antigen 6 complex, locus G5C (Ly6g5c), mRNA | 1.27 | 0,0101 |
| A\_51\_P171602 | AF011416 | Mus musculus putative pheromone receptor (VR6) mRNA, partial cds | 1.27 | 0,0078 |
| A\_51\_P517779 | NM\_147015 | Mus musculus olfactory receptor MOR180-1 (MOR180-1), mRNA | 1.27 | 0,0252 |
| A\_51\_P227165 | NM\_025865 | Mus musculus RIKEN cDNA 2310030G06 gene (2310030G06Rik), mRNA | 1.27 | 0,0403 |
| A\_51\_P104954 | NM\_173368 | Mus musculus, Similar to KIAA0308 protein, clone IMAGE:3482324, mRNA | 1.27 | 0,0345 |
| A\_51\_P339154 | NM\_146058 | Mus musculus, Similar to lymphocyte antigen 6 complex, locus F, clone MGC:29251 IMAGE:5053382, mRNA, complete cds | 1.27 | 0,0118 |
| A\_51\_P307944 | NM\_010102 | Mus musculus endothelial differentiation, G-protein-coupled receptor 6 (Edg6), mRNA | 1.27 | 0,0110 |
| A\_51\_P267617 | NM\_008125 | Mus musculus gap junction membrane channel protein beta 2 (Gjb2), mRNA | 1.27 | 0,0197 |
| A\_51\_P167263 | NM\_007650 | Mus musculus CD5 antigen (Cd5), mRNA | 1.27 | 0,0187 |
| A\_51\_P119525 | NM\_054069 | Mus musculus prostatic steroid binding protein C1 (Psbpc1-pending), mRNA | 1.27 | 0,0070 |
| A\_51\_P205233 | NAP057132-1 | Mus musculus olfactory receptor GA\_x5J8B7W4CQV-104617-103730 (GA\_x5J8B7W4CQV-104617-103730) pseudogene | 1.27 | 0,0073 |
| A\_51\_P321820 | AK076022 | hypothetical protein | 1.27 | 0,0226 |
| A\_51\_P501235 | AK015689 | weakly similar to CDNA FLJ20514 FIS, CLONE KAT09756 (HYPOTHETICAL 28.6 KDA PROTEIN) (SIMILAR TO HYPOTHETICAL PROTEIN FLJ20514) [Homo sapiens] | 1.27 | 0,0366 |
| A\_51\_P164530 | NM\_010210 | Mus musculus fragile histidine triad gene (Fhit), mRNA | 1.27 | 0,0291 |
| A\_51\_P211740 | NM\_172825 | CDNA FLJ14454 FIS, CLONE HEMBB1001872, WEAKLY SIMILAR TO CELL SURFACE GLYCOPROTEIN EMR1 PRECURSOR homolog [Homo sapiens] | 1.27 | 0,0146 |
| A\_51\_P520956 | AK006959 | hypothetical MAGE family containing protein | 1.27 | 0,0070 |
| A\_51\_P186294 | NM\_133203 | Mus musculus Ly-49Q mRNA for NK receptor Ly-49Q, complete cds | 1.27 | 0,0172 |
| A\_51\_P435410 | NM\_133355 | Mus musculus glutamate receptor, ionotropic, delta 2 (Grid2) interacting protein 1 (Grid2ip), mRNA | 1.27 | 0,0224 |
| A\_51\_P190281 | NM\_008765 | Mus musculus origin recognition complex, subunit 2 homolog (S. cerevisiae) (Orc2), mRNA | 1.27 | 0,0231 |
| A\_51\_P393086 | NM\_145502 | Mus musculus, similar to Caenorhabditis elegans protein C42C1.9, clone MGC:19110 IMAGE:4208238, mRNA, complete cds | 1.27 | 0,0268 |
| A\_51\_P342810 | NM\_027883 | KIAA0134 RNA HELICASE (HRH1) (FRAGMENT) homolog [Homo sapiens] | 1.27 | 0,0251 |
| A\_51\_P338655 | AF135494 | DELTA NAIP PROTEIN (FRAGMENT) | 1.27 | 0,0310 |
| A\_51\_P199249 | NM\_146839 | Mus musculus olfactory receptor MOR262-7 (MOR262-7), mRNA | 1.27 | 0,0316 |
| A\_51\_P115738 | NM\_053248 | Mus musculus solute carrier family 5 (sodium iodide symporter), member 5 (Slc5a5), mRNA | 1.27 | 0,0306 |
| A\_51\_P491545 | NM\_147108 | Mus musculus olfactory receptor MOR223-1 (MOR223-1), mRNA | 1.27 | 0,0111 |
| A\_51\_P165884 | NM\_001011755 | Mus musculus olfactory receptor MOR32-12 (MOR32-12) pseudogene | 1.27 | 0,0297 |
| A\_51\_P271930 | NM\_029597 | hypothetical protein | 1.27 | 0,0178 |
| A\_51\_P107652 | NM\_023557 | Mus musculus RIKEN cDNA 2210409B01 gene (2210409B01Rik), mRNA | 1.27 | 0,0122 |
| A\_51\_P172004 | NM\_026594 | Mus musculus RIKEN cDNA 4930517K11 gene (4930517K11Rik), mRNA | 1.27 | 0,0435 |
| A\_51\_P434557 | NM\_008415 | Mus musculus jerky (Jrk), mRNA | 1.27 | 0,0152 |
| A\_51\_P158238 | AK087696 | hypothetical CD9/CD37/CD63 antigens containing protein | 1.27 | 0,0079 |
| A\_51\_P155073 | NM\_025399 | Mus musculus, RIKEN cDNA 1110030M18 gene, clone MGC:29286 IMAGE:3986887, mRNA, complete cds | 1.27 | 0,0224 |
| A\_51\_P504613 | AK078580 | unclassifiable | 1.27 | 0,0426 |
| A\_51\_P115487 | M36516 | Mouse zinc finger protein (mkr5) mRNA, 3 end | 1.27 | 0,0481 |
| A\_51\_P116985 | NM\_001004721 | BA346K17.2 (A NOVEL PROTEIN SIMILAR TO THE CELL DIVISION CONTROL PROTEIN 91 (CDC91, YLR459W OR L9122.2) FROM YEAST) homolog [Homo sapiens] | 1.27 | 0,0159 |
| A\_51\_P373208 | AK079259 | KIAA0372 PROTEIN homolog [Homo sapiens] | 1.27 | 0,0185 |
| A\_51\_P294255 | NM\_178413 | Mus musculus, clone MGC:38040 IMAGE:5250319, mRNA, complete cds | 1.27 | 0,0388 |
| A\_51\_P262131 | NM\_146698 | Mus musculus olfactory receptor MOR202-8 (MOR202-8), mRNA | 1.27 | 0,0177 |
| A\_51\_P447946 | NM\_016859 | Mus musculus bystin-like (Bysl), mRNA | 1.27 | 0,0282 |
| A\_51\_P130439 | NM\_009464 | Mus musculus uncoupling protein 3, mitochondrial (Ucp3), mRNA | 1.27 | 0,0391 |
| A\_51\_P302324 | AA739048 | Mus musculus mRNA for HGT keratin, partial cds | 1.27 | 0,0252 |
| A\_51\_P271755 | AA867168 | Mus musculus, clone IMAGE:1380536, mRNA, partial cds | 1.27 | 0,0244 |
| A\_51\_P487004 | NM\_027732 | similar to DOUBLESEX-MAB-3 (DM) DOMAIN (FRAGMENT) [Homo sapiens] | 1.27 | 0,0198 |
| A\_51\_P233855 | NM\_172750 | ADP RIBOSYLARGININE HYDROLASE EC 3.2.2.19 ADP RIBOSE L ARGININE CLEAVING | 1.27 | 0,0154 |
| A\_51\_P183197 | NM\_026415 | Mus musculus RIKEN cDNA 2310002J15 gene (2310002J15Rik), mRNA | 1.27 | 0,0291 |
| A\_51\_P480441 | NM\_146934 | Mus musculus olfactory receptor MOR253-10P (MOR253-10P) pseudogene | 1.27 | 0,0124 |
| A\_51\_P453303 | AK029737 | NEURABIN-I (NEURAL TISSUE-SPECIFIC F-ACTIN BINDING PROTEIN I) (PROTEIN PHOSPHATASE 1 REGULATORY SUBUNIT 9A) (P180) (PP1BP175) homolog [Rattus norvegicus] | 1.26 | 0,0161 |
| A\_51\_P157171 | AK082864 | similar to GAS-2 RELATED PROTEIN ON CHROMOSOME 22 (GAR22 PROTEIN) [Homo sapiens] | 1.26 | 0,0193 |
| A\_51\_P401580 | NM\_010030 | Mus musculus defensin beta 2 (Defb2), mRNA | 1.26 | 0,0142 |
| A\_51\_P495550 | NM\_001011830 | Mus musculus olfactory receptor MOR263-7 (MOR263-7) pseudogene | 1.26 | 0,0139 |
| A\_51\_P211978 | NM\_146604 | Mus musculus olfactory receptor MOR260-2 (MOR260-2), mRNA | 1.26 | 0,0160 |
| A\_51\_P151020 | NM\_009407 | Mus musculus transition protein 1 (Tnp1), mRNA | 1.26 | 0,0220 |
| A\_51\_P410462 | AW113486 | Mus musculus cDNA, 3 end | 1.26 | 0,0234 |
| A\_51\_P191089 | NM\_010593 | Mus musculus plakoglobin mRNA, partial cds | 1.26 | 0,0340 |
| A\_51\_P367880 | NM\_008474 | Mus musculus type II 65kD keratin (Krt2-16) mRNA, complete cds | 1.26 | 0,0388 |
| A\_51\_P304431 | NAP057072-1 | Mus musculus olfactory receptor GA\_x5J8B7W6KF8-5563315-5562883 (GA\_x5J8B7W6KF8-5563315-5562883) pseudogene | 1.26 | 0,0317 |
| A\_51\_P286460 | NM\_178187 | HISTONE H2A homolog [Homo sapiens] | 1.26 | 0,0403 |
| A\_51\_P393634 | NM\_013759 | Mus musculus selenoprotein R (Sepr), mRNA | 1.26 | 0,0434 |
| A\_51\_P505852 | BC027044 | Mus musculus, non-metastatic cells 1, protein (NM23A) expressed in, clone MGC:36664 IMAGE:5367221, mRNA, complete cds | 1.26 | 0,0156 |
| A\_51\_P488108 | AF240172 | Mus musculus MRP9 mRNA, partial cds | 1.26 | 0,0195 |
| A\_51\_P493627 | NM\_194334 | hypothetical RabGAP/TBC domain/Pleckstrin homology (PH) domain containing protein | 1.26 | 0,0154 |
| A\_51\_P310590 | NM\_007436 | Mus musculus aldehyde dehydrogenase family 3, subfamily A1 (Aldh3a1), mRNA | 1.26 | 0,0308 |
| A\_51\_P120987 | AK081466 | phosphodiesterase 4D, cAMP specific | 1.26 | 0,0262 |
| A\_51\_P327713 | NM\_001012402 | HEPARAN SULFATE D GLUCOSAMINYL 3 O SULFOTRANSFERASE | 1.26 | 0,0312 |
| A\_51\_P307831 | NM\_183124 | hypothetical Defensin-like structure containing protein | 1.26 | 0,0437 |
| A\_51\_P286798 | AK086725 | Mus musculus melanoma antigen, family L, 2 (Magel2), mRNA | 1.26 | 0,0430 |
| A\_51\_P462629 | BC027245 | Mus musculus, clone MGC:28005 IMAGE:3602400, mRNA, complete cds | 1.26 | 0,0287 |
| A\_51\_P143855 | NM\_026402 | Mus musculus RIKEN cDNA 2610016C12 gene (2610016C12Rik), mRNA | 1.26 | 0,0248 |
| A\_51\_P213737 | AK029749 | protocadherin beta 12 | 1.26 | 0,0314 |
| A\_51\_P300867 | NM\_177395 | MIXED LINEAGE KINASE MLK1 (FRAGMENT) homolog [Homo sapiens] | 1.26 | 0,0431 |
| A\_51\_P439584 | NM\_145530 | RHO FAMILY GTPASE CHP homolog [Homo sapiens] | 1.26 | 0,0169 |
| A\_51\_P380056 | BC016220 | hypothetical protein | 1.26 | 0,0215 |
| A\_51\_P219527 | NM\_025308 | Mus musculus EST AA238765 (AA238765), mRNA | 1.26 | 0,0305 |
| A\_51\_P359729 | A\_51\_P359729 | Mus musculus cDNA, 3 end | 1.26 | 0,0494 |
| A\_51\_P416278 | AK038734 | hypothetical protein | 1.26 | 0,0133 |
| A\_51\_P331690 | NM\_015745 | Mus musculus, Similar to retinol-binding protein 3, interstitial, clone MGC:27658 IMAGE:4527589, mRNA, complete cds | 1.26 | 0,0473 |
| A\_51\_P155714 | AK014457 | similar to MIP-T3 [Homo sapiens] | 1.26 | 0,0454 |
| A\_51\_P227946 | XM\_484393 | similar to NEURONAL PROTEIN [Felis silvestris catus] | 1.26 | 0,0206 |
| A\_51\_P432773 | BC005782 | Mus musculus, clone MGC:12025 IMAGE:3603243, mRNA, complete cds | 1.26 | 0,0144 |
| A\_51\_P162023 | NM\_134205 | Mus musculus vomeronasal 1 receptor, G4 (V1rg4), mRNA | 1.26 | 0,0305 |
| A\_51\_P211171 | AK002608 | Mus musculus 3-hydroxyanthranilate 3,4-dioxygenase (Haao), mRNA | 1.26 | 0,0306 |
| A\_51\_P325889 | AK037440 | unknown EST | 1.26 | 0,0172 |
| A\_51\_P433388 | AK006101 | hypothetical Glutamic acid-rich region containing protein | 1.26 | 0,0106 |
| A\_51\_P406433 | AK016466 | hypothetical SEC7-like domain/SEC7 domain profile containing protein | 1.25 | 0,0293 |
| A\_51\_P126258 | NM\_033584 | Mus musculus protocadherin gamma subfamily A, 1 (Pcdhga1), mRNA | 1.25 | 0,0127 |
| A\_51\_P361242 | NM\_177394 | hypothetical protein | 1.25 | 0,0339 |
| A\_51\_P267169 | NM\_001004173 | unknown EST | 1.25 | 0,0374 |
| A\_51\_P275283 | AK047234 | similar to SORTING NEXIN 6 (TRAF4-ASSOCIATED FACTOR 2) [Homo sapiens] | 1.25 | 0,0183 |
| A\_51\_P405283 | AK020284 | hypothetical Dbl domain (dbl/cdc24 rhoGEF family) containing protein | 1.25 | 0,0447 |
| A\_51\_P138755 | NM\_177912 | similar to MLZE [Mus musculus] | 1.25 | 0,0168 |
| A\_51\_P458748 | NM\_013552 | Mus musculus hyaluronan mediated motility receptor (RHAMM) (Hmmr), mRNA | 1.25 | 0,0162 |
| A\_51\_P125665 | NM\_198110 | Mus musculus, clone IMAGE:3157942, mRNA | 1.25 | 0,0168 |
| A\_51\_P120645 | NM\_027087 | Mus musculus RIKEN cDNA 2300006N05 gene (2300006N05Rik), mRNA | 1.25 | 0,0494 |
| A\_51\_P451395 | TC1520710 | Mus musculus cDNA, 5 end | 1.25 | 0,0460 |
| A\_51\_P192824 | NM\_146274 | Mus musculus olfactory receptor MOR245-21 (MOR245-21), mRNA | 1.25 | 0,0259 |
| A\_51\_P415095 | AK083149 | synapsin I | 1.25 | 0,0165 |
| A\_51\_P342418 | NM\_177661 | ARYLACETAMIDE DEACETYLASE EC 3.1.1.- | 1.25 | 0,0345 |
| A\_51\_P107985 | AK006597 | hypothetical protein | 1.25 | 0,0238 |
| A\_51\_P472419 | NM\_018798 | Mus musculus ubiquilin 2 (Ubqln2), mRNA | 1.25 | 0,0286 |
| A\_51\_P431684 | NM\_146582 | Mus musculus olfactory receptor MOR194-1 (MOR194-1), mRNA | 1.25 | 0,0305 |
| A\_51\_P201751 | NM\_146904 | Mus musculus olfactory receptor 72 gene (Olfr72), mRNA | 1.25 | 0,0146 |
| A\_51\_P388835 | NM\_009380 | Mus musculus thyroid hormone receptor beta (Thrb), mRNA | 1.25 | 0,0294 |
| A\_51\_P515462 | NM\_146725 | Mus musculus olfactory receptor MOR268-2 (MOR268-2), mRNA | 1.25 | 0,0400 |
| A\_51\_P451226 | AK015074 | 3-phosphoglycerate dehydrogenase | 1.25 | 0,0364 |
| A\_51\_P380572 | AK016678 | hypothetical protein | 1.25 | 0,0301 |
| A\_51\_P393754 | NM\_153064 | Mus musculus, Similar to NADH dehydrogenase (ubiquinone) Fe-S protein 2 (49kD) (NADH-coenzyme Q reductase), clone MGC:27667 IMAGE:4910260, mRNA, complete cds | 1.25 | 0,0179 |
| A\_51\_P107113 | NM\_016694 | Mus musculus parkin (Park2), mRNA | 1.25 | 0,0217 |
| A\_51\_P381449 | AK015845 | unclassifiable | 1.25 | 0,0104 |
| A\_51\_P177323 | NAP101619-1 | ALKALINE PHOSPHATASE, PRECURSOR EC 3.1.3.1 | 1.25 | 0,0184 |
| A\_51\_P113773 | NM\_029420 | inferred: Unknown (protein for MGC:2532) {Homo sapiens} | 1.25 | 0,0428 |
| A\_51\_P433399 | NM\_139141 | zinc finger protein 192 | 1.25 | 0,0420 |
| A\_51\_P223504 | NM\_031261 | Mus musculus ferritin, heavy polypeptide-like 17 (Fthl17), mRNA | 1.25 | 0,0498 |
| A\_51\_P412895 | NM\_146591 | Mus musculus olfactory receptor MOR179-3 (MOR179-3), mRNA | 1.25 | 0,0142 |
| A\_51\_P504517 | NM\_027083 | similar to LYSOZYME HOMOLOG [Homo sapiens] | 1.25 | 0,0225 |
| A\_51\_P480796 | BC070476 | ANTISENSE RNA OVERLAPPING MCH PROTEIN [Rattus norvegicus] | 1.25 | 0,0323 |
| A\_51\_P406193 | AK020389 | hypothetical protein | 1.25 | 0,0182 |
| A\_51\_P268812 | NAP057061-1 | Mus musculus olfactory receptor GA\_x5J8B7W89HK-6403592-6404251 (GA\_x5J8B7W89HK-6403592-6404251) pseudogene | 1.25 | 0,0233 |
| A\_51\_P170725 | NM\_028788 | hypothetical protein | 1.25 | 0,0160 |
| A\_51\_P292221 | NM\_010608 | Mus musculus Kcnk3 channel mRNA, complete cds | 1.25 | 0,0263 |
| A\_51\_P154946 | AK082737 | WEAKLY SIMILAR TO L- RIBULOKINASE homolog [Homo sapiens] | 1.24 | 0,0162 |
| A\_51\_P407533 | NAP057090-1 | Mus musculus olfactory receptor GA\_x5J8B7W6337-1183223-1183810 (GA\_x5J8B7W6337-1183223-1183810) pseudogene | 1.24 | 0,0244 |
| A\_51\_P455082 | NM\_010546 | Mus musculus IkB kinase-beta (Ikkb) mRNA, complete cds | 1.24 | 0,0205 |
| A\_51\_P141560 | NAP057190-1 | Mus musculus olfactory receptor MOR175-8P (MOR175-8P) pseudogene | 1.24 | 0,0155 |
| A\_51\_P402868 | AK046215 | weakly similar to GROUP III SECRETED PHOSPHOLIPASE A2 | 1.24 | 0,0322 |
| A\_51\_P485985 | NM\_134163 | Mus musculus CHCR (CHCR), mRNA | 1.24 | 0,0482 |
| A\_51\_P110791 | NM\_009212 | Mus musculus immunoglobulin mu binding protein 2 (Ighmbp2), mRNA | 1.24 | 0,0152 |
| A\_51\_P319527 | NAP057288-1 | Mus musculus olfactory receptor MOR177-11P (MOR177-11P) pseudogene | 1.24 | 0,0236 |
| A\_51\_P273657 | XM\_355958 | Mus musculus, clone IMAGE:5036118, mRNA | 1.24 | 0,0455 |
| A\_51\_P308625 | NM\_146759 | Mus musculus olfactory receptor MOR119-1 (MOR119-1), mRNA | 1.24 | 0,0366 |
| A\_51\_P324484 | AK079063 | unknown EST | 1.24 | 0,0200 |
| A\_51\_P456196 | NM\_001011818 | Mus musculus olfactory receptor MOR285-3P (MOR285-3P) pseudogene | 1.24 | 0,0299 |
| A\_51\_P433360 | NM\_177406 | M.musculus Cyp4a-12 mRNA | 1.24 | 0,0268 |
| A\_51\_P301696 | A\_51\_P301696 | Mus musculus cDNA, 3 end | 1.24 | 0,0285 |
| A\_51\_P109439 | NM\_146325 | Mus musculus olfactory receptor MOR25-1 (MOR25-1), mRNA | 1.24 | 0,0246 |
| A\_51\_P467498 | NM\_013689 | Mus musculus cytoplasmic tyrosine kinase, Dscr28C related (Drosophila) (Tec), mRNA | 1.24 | 0,0282 |
| A\_51\_P355829 | NM\_010504 | Mus musculus interferon alpha family, gene 4 (Ifna4), mRNA | 1.24 | 0,0185 |
| A\_51\_P205559 | NM\_029331 | Mus musculus RIKEN cDNA 1700019G17 gene (1700019G17Rik), mRNA | 1.24 | 0,0103 |
| A\_51\_P171086 | AK083525 | unknown EST | 1.24 | 0,0140 |
| A\_51\_P190111 | NM\_008566 | Mus musculus mini chromosome maintenance deficient 5 (S. cerevisiae) (Mcmd5), mRNA | 1.24 | 0,0365 |
| A\_51\_P107839 | NAP057180-1 | Mus musculus olfactory receptor MOR248-13P (MOR248-13P) pseudogene | 1.24 | 0,0388 |
| A\_51\_P391560 | NM\_198927 | musculus, clone IMAGE:4923848, mRNA, partial cds | 1.24 | 0,0305 |
| A\_51\_P393288 | NM\_146988 | Mus musculus olfactory receptor MOR261-1 (MOR261-1), mRNA | 1.24 | 0,0152 |
| A\_51\_P388450 | NM\_181682 | M.musculus mRNA for desmoglein type 1 | 1.24 | 0,0325 |
| A\_51\_P453456 | AK006510 | unknown EST | 1.24 | 0,0305 |
| A\_51\_P240286 | NM\_007608 | Mus musculus carbonic anhydrase 5a, mitochondrial (Car5a), mRNA | 1.24 | 0,0318 |
| A\_51\_P208534 | AK021185 | hypothetical protein | 1.23 | 0,0367 |
| A\_51\_P413660 | AK041728 | unknown EST | 1.23 | 0,0258 |
| A\_51\_P275163 | NM\_146354 | Mus musculus olfactory receptor MOR12-5 (MOR12-5), mRNA | 1.23 | 0,0258 |
| A\_51\_P153036 | NM\_020502 | Mus musculus candidate taste receptor T2R8 gene (T2r8), mRNA | 1.23 | 0,0243 |
| A\_51\_P404263 | NM\_175236 | CDNA FLJ32430 FIS, CLONE SKMUS2001129, WEAKLY SIMILAR TO NAD-DEPENDENT METHANOL DEHYDROGENASE (EC 1.1.1.244) homolog [Homo sapiens] | 1.23 | 0,0267 |
| A\_51\_P301023 | NM\_133895 | Mus musculus expressed sequence AA987064 (AA987064), mRNA | 1.23 | 0,0476 |
| A\_51\_P349713 | NM\_022016 | Mus musculus interphotoreceptor matrix proteoglycan 1 (Impg1), mRNA | 1.23 | 0,0166 |
| A\_51\_P283997 | AK051490 | unknown EST | 1.23 | 0,0178 |
| A\_51\_P124968 | NM\_144548 | Mus musculus interleukin 23 receptor (Il23r), mRNA | 1.23 | 0,0383 |
| A\_51\_P486350 | NM\_001018013 | hypothetical Immunoglobulin structure containing protein | 1.23 | 0,0266 |
| A\_51\_P313602 | NM\_053222 | Mus musculus vomeronasal 1 receptor, A7 (V1ra7), mRNA | 1.23 | 0,0434 |
| A\_51\_P230055 | NM\_146504 | ODORANT RECEPTOR M37 (OLFACTORY RECEPTOR MOR224-5) | 1.23 | 0,0216 |
| A\_51\_P490879 | TC1521143 | Mus musculus cDNA, 3 end | 1.23 | 0,0281 |
| A\_51\_P406539 | AK047455 | unknown EST | 1.23 | 0,0220 |
| A\_51\_P382688 | NM\_026021 | Mus musculus RIKEN cDNA 2700064H14 gene (2700064H14Rik), mRNA | 1.23 | 0,0406 |
| A\_51\_P362531 | NM\_144835 | SIMILAR TO HYPOTHETICAL PROTEIN FLJ10359 homolog [Mus musculus] | 1.23 | 0,0381 |
| A\_51\_P415931 | NM\_172844 | weakly similar to DIMETHYLANILINE MONOOXYGENASE [N-OXIDE FORMING] 5 (EC 1.14.13.8) (HEPATIC FLAVIN-CONTAINING MONOOXYGENASE 5) (FMO 5) (DIMETHYLANILINE OXIDASE 5) (FMO 1C1) (FMO FORM 3) [Oryctolagus cuniculus] | 1.23 | 0,0416 |
| A\_51\_P155582 | NM\_009013 | Mus musculus RAD51 associated protein 1 (Rad51ap1), mRNA | 1.23 | 0,0159 |
| A\_51\_P242695 | NM\_001011771 | Mus musculus olfactory receptor MOR193-1 (MOR193-1) pseudogene | 1.23 | 0,0162 |
| A\_51\_P509679 | NM\_001024700 | IG MU CHAIN C REGION | 1.23 | 0,0134 |
| A\_51\_P328818 | NM\_207673 | Mus musculus olfactory receptor GA\_x5J8B7W5KGR-1200441-1200915 (GA\_x5J8B7W5KGR-1200441-1200915) pseudogene | 1.23 | 0,0160 |
| A\_51\_P331549 | NM\_178878 | "TRIFUNCTIONAL ENZYME ALPHA SUBUNIT, MITOCHONDRIAL PRECURSOR (TP-ALPHA) [INCLUDES: LONG-CHAIN ENOYL-COA HYDRATASE (EC 4.2.1.17)� LONG CHAIN 3-HYDROXYACYL-COA DEHYDROGENASE (EC 1.1.1.35)] homolog [Rattus norvegicus]" | 1.23 | 0,0454 |
| A\_51\_P452367 | NM\_013675 | Mus musculus beta-spectrin 1 (Spnb1), mRNA | 1.23 | 0,0311 |
| A\_51\_P233801 | NM\_009626 | alcohol dehydrogenase 3 complex | 1.23 | 0,0212 |
| A\_51\_P254302 | XM\_126776 | hypothetical protein | 1.22 | 0,0197 |
| A\_51\_P250319 | NM\_009470 | Mus musculus uromodulin mRNA, complete cds | 1.22 | 0,0407 |
| A\_51\_P151732 | NM\_019645 | Mus musculus plakophilin 1 (Pkp1), mRNA | 1.22 | 0,0314 |
| A\_51\_P214275 | XM\_618928 | M.musculus mRNA for type I keratin (MHR a-1) | 1.22 | 0,0242 |
| A\_51\_P123581 | A\_51\_P123581 | Mus musculus cDNA, 3 end | 1.22 | 0,0310 |
| A\_51\_P151243 | NM\_207560 | OLFACTORY RECEPTOR | 1.22 | 0,0485 |
| A\_51\_P514730 | NM\_026615 | Mus musculus, RIKEN cDNA 2900073H19 gene, clone MGC:36675 IMAGE:5368627, mRNA, complete cds | 1.22 | 0,0272 |
| A\_51\_P387072 | NM\_177571 | hypothetical RNI-like structure containing protein | 1.22 | 0,0292 |
| A\_51\_P497931 | NM\_175452 | Mus musculus gap junction membrane channel protein alpha 12 (Gja12-pending), mRNA | 1.22 | 0,0419 |
| A\_51\_P334763 | AK006255 | hypothetical protein | 1.22 | 0,0338 |
| A\_51\_P410219 | NM\_027633 | HYPOTHETICAL 20.2 KDA PROTEIN homolog [Homo sapiens] | 1.22 | 0,0314 |
| A\_51\_P404244 | NM\_033478 | Mus musculus lymphocyte antigen 6 complex, locus G6D (Ly6g6d), mRNA | 1.22 | 0,0198 |
| A\_51\_P427812 | NM\_178638 | hypothetical protein | 1.22 | 0,0376 |
| A\_51\_P520751 | A\_51\_P520751 | Mus musculus cDNA, 3 end | 1.22 | 0,0459 |
| A\_51\_P398633 | NM\_007418 | Mus musculus adrenergic receptor, alpha 2c (Adra2c), mRNA | 1.22 | 0,0320 |
| A\_51\_P415051 | NAP057097-1 | Mus musculus olfactory receptor GA\_x5J8B7W62NC-1313389-1313225 (GA\_x5J8B7W62NC-1313389-1313225) pseudogene | 1.22 | 0,0397 |
| A\_51\_P459091 | NM\_172550 | hypothetical Uncharacterized protein family UPF0054 containing protein | 1.22 | 0,0403 |
| A\_51\_P187184 | XM\_355941 | inferred: serine/threonine protein kinase TAO2 {Rattus norvegicus} | 1.21 | 0,0240 |
| A\_51\_P353232 | NM\_009394 | Mus musculus troponin C, fast skeletal (Tncs), mRNA | 1.21 | 0,0431 |
| A\_51\_P341688 | NM\_153061 | Mus musculus transforming growth factor beta 1-induced factor 2 mRNA, complete cds | 1.21 | 0,0426 |
| A\_51\_P486144 | NM\_147155 | Mus musculus FKSG15 (FKSG15) mRNA, complete cds | 1.21 | 0,0482 |
| A\_51\_P280697 | NM\_008118 | Mus musculus gastric intrinsic factor (Gif), mRNA | 1.21 | 0,0302 |
| A\_51\_P477972 | NM\_001011756 | Mus musculus olfactory receptor MOR171-45 (MOR171-45) pseudogene | 1.21 | 0,0366 |
| A\_51\_P285056 | NM\_023764 | Mus musculus toll interacting protein (Tollip-pending), mRNA | 1.21 | 0,0449 |
| A\_51\_P239376 | NM\_177356 | Mus musculus lysosomal-associated membrane protein 3 (Lamp3), mRNA | 1.21 | 0,0321 |
| A\_51\_P198453 | NM\_145710 | Mus musculus oocyte specific homeobox 6 (Obox6), mRNA | 1.21 | 0,0198 |
| A\_51\_P386688 | NM\_133359 | Mus musculus high-glycine/tyrosine protein type I E5 (LOC170939), mRNA | 1.21 | 0,0464 |
| A\_51\_P209357 | NM\_053258 | Mus musculus ectopic ossification 1 (Etos1), mRNA | 1.21 | 0,0305 |
| A\_51\_P312916 | BF464414 | Mus musculus cDNA, 3 end | 1.21 | 0,0422 |
| A\_51\_P130676 | NM\_020522 | Mus musculus vomeronasal 1 receptor, B6 (V1rb6), mRNA | 1.21 | 0,0426 |
| A\_51\_P429715 | X90829 | Mus musculus lady bird-like homeobox 1 homolog, (Drosophila) (Lbx1h), mRNA | 1.21 | 0,0257 |
| A\_51\_P506835 | AK033449 | POZ 56 PROTEIN homolog [Mus musculus] | 1.21 | 0,0382 |
| A\_51\_P268186 | NM\_028982 | Mus musculus RIKEN cDNA 8430419L09 gene (8430419L09Rik), mRNA | 1.21 | 0,0442 |
| A\_51\_P472638 | NM\_009952 | Mus musculus cAMP responsive element binding protein 1 (Creb1), mRNA | 1.21 | 0,0288 |
| A\_51\_P200544 | NM\_001001495 | weakly similar to ABIN-3 PROTEIN [Homo sapiens] | 1.21 | 0,0225 |
| A\_51\_P371647 | NM\_147037 | Mus musculus olfactory receptor MOR208-1 (MOR208-1), mRNA | 1.21 | 0,0429 |
| A\_51\_P233338 | NM\_146934 | Mus musculus olfactory receptor F5 mRNA, partial cds | 1.21 | 0,0241 |
| A\_51\_P312856 | AI840671 | Mus musculus cDNA, 3 end | 1.21 | 0,0469 |
| A\_51\_P168989 | NM\_018792 | Mus musculus histone H1-like protein in spermatids 1 (Hils1), mRNA | 1.21 | 0,0443 |
| A\_51\_P371695 | XM\_620559 | PHOSPHATIDYLINOSITOL 3-KINASE-RELATED PROTEIN KINASE homolog [Homo sapiens] | 1.21 | 0,0427 |
| A\_51\_P213651 | BC018431 | Mus musculus, Similar to HCR (a-helix coiled-coil rod homologue), clone IMAGE:3602525, mRNA | 1.21 | 0,0436 |
| A\_51\_P106169 | NM\_011829 | Mus musculus inosine 5-phosphate dehydrogenase 1 (Impdh1), mRNA | 1.21 | 0,0204 |
| A\_51\_P429770 | NM\_010184 | Mus musculus Fc receptor, IgE, high affinity I, alpha polypeptide (Fcer1a), mRNA | 1.21 | 0,0461 |
| A\_51\_P196019 | NM\_011643 | Mus musculus transient receptor protein 1 (Trrp1), mRNA | 1.21 | 0,0327 |
| A\_51\_P276364 | NAP108673-1 | Mus musculus olfactory receptor MOR159-2P (MOR159-2P) pseudogene | 1.21 | 0,0443 |
| A\_51\_P407494 | NM\_177099 | LEFT-RIGHT DETERMINATION FACTOR B PRECURSOR (LEFTY-2 PROTEIN) | 1.21 | 0,0369 |
| A\_51\_P271573 | A\_51\_P271573 | Mus musculus cDNA, 5 end | 1.21 | 0,0374 |
| A\_51\_P443032 | NM\_013616 | Mus musculus olfactory receptor 64 (Olfr64), mRNA | 1.21 | 0,0425 |
| A\_51\_P449644 | TC1427190 | Mus musculus cDNA, 5 end | 1.20 | 0,0317 |
| A\_51\_P474038 | TC1525739 | Mus musculus cDNA, 5 end | 1.20 | 0,0430 |
| A\_51\_P330379 | XM\_145466 | CYTOCHROME P450 EC 1.14.14.1 | 1.20 | 0,0472 |
| A\_51\_P342117 | NM\_007675 | Mus musculus CEA-related cell adhesion molecule 10 (Ceacam10), mRNA | 1.20 | 0,0340 |
| A\_51\_P483401 | A\_51\_P483401 | Mus musculus cDNA, 3 end | 1.20 | 0,0431 |
| A\_51\_P479138 | NM\_146547 | Mus musculus olfactory receptor MOR114-8 (MOR114-8), mRNA | 1.20 | 0,0446 |
| A\_51\_P483454 | NM\_177724 | hypothetical Glycine-rich region/Serine-rich region containing protein | 1.20 | 0,0434 |
| A\_51\_P179051 | NM\_146809 | Mus musculus olfactory receptor MOR239-7 (MOR239-7) pseudogene | 1.20 | 0,0350 |
| A\_51\_P109643 | NAP057031-1 | Mus musculus ribosomal protein S12, pseudogene 1 (Rps12-ps1) on chromosome X | 1.20 | 0,0351 |
| A\_51\_P504737 | NM\_146791 | Mus musculus olfactory receptor MOR231-10 (MOR231-10), mRNA | 1.20 | 0,0420 |
| A\_51\_P320953 | NM\_153416 | Mus musculus, clone MGC:38135 IMAGE:5321013, mRNA, complete cds | 1.19 | 0,0336 |
| A\_51\_P371867 | NM\_027512 | hypothetical MAGE family containing protein | 1.19 | 0,0342 |
| A\_51\_P208240 | NM\_019418 | Mus musculus tumor necrosis factor (ligand) superfamily, member 14 (Tnfsf14), mRNA | 1.19 | 0,0387 |
| A\_51\_P344177 | NM\_007401 | a disintegrin and metalloprotease domain 5 | 1.19 | 0,0492 |
| A\_51\_P191100 | AK017008 | hypothetical ARM repeat structure containing protein | 1.19 | 0,0497 |
| A\_51\_P269236 | NM\_022888 | Mus musculus folate receptor 4 (delta) (Folr4), mRNA | 1.19 | 0,0444 |
| A\_51\_P100396 | NM\_175475 | Mus musculus cytochrome P450, 26, retinoic acid B1 (Cyp26b1), mRNA | 1.18 | 0,0499 |
| A\_51\_P464279 | NM\_009757 | Mus musculus bone morphogenetic protein 15 (Bmp15), mRNA | 1.18 | 0,0410 |
| A\_51\_P395370 | AK033531 | hypothetical Membrane all-alpha structure containing protein | 1.18 | 0,0431 |
| A\_51\_P447299 | NM\_173439 | Mus musculus hypothetical protein MGC19444 (MGC19444), mRNA | 1.17 | 0,0383 |
| A\_51\_P510929 | NM\_207145 | OLFACTORY RECEPTOR MOR150-2 | 1.17 | 0,0466 |
| A\_51\_P441286 | NM\_177625 | hypothetical TPR repeat containing protein | 0.84 | 0,0462 |
| A\_51\_P413376 | NM\_009556 | Mus musculus zinc finger protein 42 (Zfp42), mRNA | 0.84 | 0,0434 |
| A\_51\_P105424 | NM\_029831 | similar to tissue kallikrein (EC 3.4.21.35), submandibular mGK-2 (fragment) [Mus musculus] | 0.84 | 0,0345 |
| A\_51\_P234483 | AF177170 | Mus musculus tropomodulin 2 (Tmod2), mRNA | 0.84 | 0,0250 |
| A\_51\_P136269 | NM\_027874 | Mus musculus casein kinase 1, delta (Csnk1d), transcript variant 1, mRNA | 0.84 | 0,0462 |
| A\_51\_P417933 | NM\_028623 | CYSTATIN N homolog [Rattus norvegicus] | 0.84 | 0,0388 |
| A\_51\_P414606 | NM\_007698 | Mus musculus cholinergic receptor, muscarinic 1, CNS (Chrm1), mRNA | 0.83 | 0,0481 |
| A\_51\_P253834 | NM\_013603 | Mus musculus metallothionein 3 (Mt3), mRNA | 0.83 | 0,0369 |
| A\_51\_P483739 | NM\_025824 | Mus musculus basic leucine zipper and W2 domains 1 (Bzw1), mRNA | 0.82 | 0,0288 |
| A\_51\_P353703 | NM\_010237 | Mus musculus B-cell src-homology tyrosine kinase (Frk), mRNA | 0.82 | 0,0442 |
| A\_51\_P261718 | NM\_134037 | Mus musculus strain ILS ATP citrate lyase mRNA, complete cds | 0.82 | 0,0434 |
| A\_51\_P391716 | NM\_013848 | Mus musculus erythroblast membrane-associated protein (Ermap), mRNA | 0.82 | 0,0319 |
| A\_51\_P140042 | XM\_486166 | hypothetical Double-stranded RNA binding (DsRBD) domain/Adenosine-deaminase (editase) domain containing protein | 0.82 | 0,0400 |
| A\_51\_P338714 | NM\_007494 | Mus musculus argininosuccinate synthetase 1 (Ass1), mRNA | 0.82 | 0,0327 |
| A\_51\_P368660 | NM\_026483 | Mus musculus, Similar to M-phase phosphoprotein 10 (U3 small nucleolar ribonucleoprotein), clone IMAGE:3979017, mRNA | 0.82 | 0,0384 |
| A\_51\_P467889 | NM\_025400 | Mus musculus RIKEN cDNA 1110028N05 gene (1110028N05Rik), mRNA | 0.82 | 0,0454 |
| A\_51\_P101426 | TC1522453 | Mus musculus DNA segment, Chr 8, Wayne State University 151, expressed (D8Wsu151e), mRNA | 0.82 | 0,0306 |
| A\_51\_P278034 | NM\_027356 | Mus musculus RIKEN cDNA 2700038N03 gene (2700038N03Rik), mRNA | 0.82 | 0,0370 |
| A\_51\_P214786 | NM\_053079 | Mus musculus solute carrier family 15 (oligopeptide transporter), member 1 (Slc15a1), mRNA | 0.81 | 0,0451 |
| A\_51\_P159792 | NM\_009928 | Mus musculus procollagen, type XV (Col15a1), mRNA | 0.81 | 0,0373 |
| A\_51\_P464600 | NM\_182840 | Mus musculus, clone IMAGE:3485728, mRNA | 0.81 | 0,0229 |
| A\_51\_P365790 | AK043052 | unknown EST | 0.81 | 0,0170 |
| A\_51\_P412050 | NM\_178192 | Mus musculus histone 4 protein (Hist4), mRNA | 0.81 | 0,0366 |
| A\_51\_P203148 | AB017433 | Mus musculus mRNA for anti-IL-18 IgG heavy chain, clone 125-2H, partial cds | 0.81 | 0,0296 |
| A\_51\_P444283 | NM\_029084 | similar to BCM-LIKE MEMBRANE PROTEIN [Homo sapiens] | 0.81 | 0,0369 |
| A\_51\_P207853 | AK008732 | musculus adult male stomach cDNA, RIKEN full-length enriched library, clone:2210015I05:hypothetical protein, full insert sequence | 0.81 | 0,0442 |
| A\_51\_P149078 | NAP057194-1 | Mus musculus olfactory receptor MOR264-15P (MOR264-15P) pseudogene | 0.81 | 0,0443 |
| A\_51\_P219098 | NM\_027019 | Mus musculus RIKEN cDNA 1700011O04 gene (1700011O04Rik), mRNA | 0.81 | 0,0239 |
| A\_51\_P465449 | NM\_008653 | Mus musculus myosin binding protein C, cardiac (Mybpc3), mRNA | 0.81 | 0,0257 |
| A\_51\_P453312 | AU067806 | Mus musculus cDNA, 3 end | 0.81 | 0,0306 |
| A\_51\_P288828 | NM\_029643 | Mus musculus, RIKEN cDNA 2010003P03 gene, clone MGC:28910 IMAGE:4921900, mRNA, complete cds | 0.81 | 0,0344 |
| A\_51\_P496741 | NM\_019459 | Mus musculus nephrosis 1 homolog, nephrin (human) (Nphs1), mRNA | 0.81 | 0,0441 |
| A\_51\_P386798 | AK006064 | hypothetical protein | 0.81 | 0,0238 |
| A\_51\_P326417 | NM\_019712 | Mus musculus ring-box 1 (Rbx1), mRNA | 0.80 | 0,0458 |
| A\_51\_P386983 | NM\_009406 | Mus musculus troponin I, cardiac (Tnni3), mRNA | 0.80 | 0,0497 |
| A\_51\_P224275 | NAP100068-001 | Mus musculus olfactory receptor MOR0-10P (MOR0-10P) pseudogene | 0.80 | 0,0255 |
| A\_51\_P387868 | AK005842 | weakly similar to TEKTIN A1 [Strongylocentrotus purpuratus] | 0.80 | 0,0310 |
| A\_51\_P201338 | NM\_144800 | Mus musculus, similar to metastasis suppressor protein, clone MGC:37896 IMAGE:5101888, mRNA, complete cds | 0.80 | 0,0186 |
| A\_51\_P385874 | NM\_011143 | Mouse Brn-3 gene POU-box region | 0.80 | 0,0125 |
| A\_51\_P206445 | AK013056 | weakly similar to CDNA FLJ31384 FIS, CLONE NHNPC2000212, MODERATELY SIMILAR TO ZINC FINGER PROTEIN 83 [Homo sapiens] | 0.80 | 0,0289 |
| A\_51\_P411072 | AK084283 | unclassifiable | 0.80 | 0,0425 |
| A\_51\_P377948 | NM\_174960 | weakly similar to HUMAN IMMUNITY ASSOCIATED PROTEIN 1 [Homo sapiens] | 0.80 | 0,0439 |
| A\_51\_P358256 | NM\_009803 | Mus musculus nuclear receptor subfamily 1, group I, member 3 (Nr1i3), mRNA | 0.80 | 0,0430 |
| A\_51\_P203183 | NM\_138310 | Mus musculus apolipoprotein B48 receptor (Apob48r-pending), mRNA | 0.80 | 0,0310 |
| A\_51\_P392560 | NAP057167-1 | Mus musculus olfactory receptor MOR110-9 (MOR110-9) pseudogene | 0.80 | 0,0313 |
| A\_51\_P256902 | NM\_031884 | Mus musculus ATP-binding cassette, sub-family G (WHITE), member 5 (Abcg5), mRNA | 0.80 | 0,0452 |
| A\_51\_P140956 | NM\_027164 | weakly similar to KIAA1674 PROTEIN (FRAGMENT) [Homo sapiens] | 0.80 | 0,0272 |
| A\_51\_P222733 | NM\_010381 | Mus musculus histocompatibility 2, class II antigen E alpha (H2-Ea), mRNA | 0.80 | 0,0266 |
| A\_51\_P149872 | NM\_010596 | Mus musculus potassium voltage-gated channel, shaker-related subfamily, member 7 (Kcna7), mRNA | 0.80 | 0,0436 |
| A\_51\_P410853 | NM\_025949 | RIBOSOMAL PROTEIN S6 KINASE ALPHA 6 (EC 2.7.1.-) (S6K-ALPHA 6) (90 KDA RIBOSOMAL PROTEIN S6 KINASE 6) (P90-RSK 6) (RIBOSOMAL S6 KINASE 4) (RSK-4) (PP90RSK4) homolog [Homo sapiens] | 0.80 | 0,0470 |
| A\_51\_P343041 | NM\_016968 | Mus musculus oligodendrocyte transcription factor 1 (Olig1), mRNA | 0.80 | 0,0229 |
| A\_51\_P316497 | NM\_001025067 | hypothetical Cysteine-rich flanking region, C-terminal/Immunoglobulin and major histocompatibility complex domain/Immunoglobulin C-2 type/Immunoglobulin subtype containing protein | 0.80 | 0,0387 |
| A\_51\_P471057 | AF357403 | Mus musculus clone MBI-100 H/ACA box snoRNA, partial sequence | 0.80 | 0,0499 |
| A\_51\_P376501 | AK029170 | hypothetical Serine-rich region containing protein | 0.80 | 0,0466 |
| A\_51\_P279062 | NM\_027763 | hypothetical Immunoglobulin subtype containing protein | 0.79 | 0,0251 |
| A\_51\_P244856 | NM\_007504 | SARCOPLASMIC/ENDOPLASMIC RETICULUM CALCIUM ATPASE 2 EC 3.6.3.8 CALCIUM PUMP 2 SERCA2 SR CA 2+ ATPASE 2 CALCIUM TRANSPORTING ATPASE SARCOPLASMIC RETICULUM TYPE, SLOW TWITCH SKELETAL MUSCLE ISOFORM ENDOPLASMIC RETICULUM CLASS 1/2 CA 2+ | 0.79 | 0,0200 |
| A\_51\_P136516 | NM\_009732 | Mus musculus arginine vasopressin (Avp), mRNA | 0.79 | 0,0362 |
| A\_51\_P501299 | NM\_029203 | Mus musculus RIKEN cDNA 4930539I12 gene (4930539I12Rik), mRNA | 0.79 | 0,0438 |
| A\_51\_P443508 | NM\_026438 | Mus musculus RIKEN cDNA 2010317E03 gene (2010317E03Rik), mRNA | 0.79 | 0,0387 |
| A\_51\_P289889 | NM\_033525 | Mus musculus nephronectin (Npnt), mRNA | 0.79 | 0,0320 |
| A\_51\_P314669 | NM\_011128 | Mus musculus pancreatic lipase-related protein 2 (Pnliprp2), mRNA | 0.79 | 0,0181 |
| A\_51\_P240191 | NM\_198927 | hypothetical Serine-rich region containing protein | 0.79 | 0,0471 |
| A\_51\_P483499 | NM\_011453 | Mus musculus serine protease inhibitor 11 (Spi11), mRNA | 0.79 | 0,0498 |
| A\_51\_P328659 | NM\_146585 | Mus musculus olfactory receptor MOR185-5 (MOR185-5), mRNA | 0.79 | 0,0394 |
| A\_51\_P200930 | NM\_025737 | Mus musculus RIKEN cDNA 4931417E11 gene (4931417E11Rik), mRNA | 0.79 | 0,0296 |
| A\_51\_P356467 | NM\_009903 | Mus musculus claudin 4 (Cldn4), mRNA | 0.79 | 0,0308 |
| A\_51\_P359891 | NM\_011426 | Mus musculus sialoadhesin (Sn), mRNA | 0.79 | 0,0394 |
| A\_51\_P119358 | AK005633 | hypothetical Apoptosis regulator protein, Bcl-2 family BH domain containing protein | 0.79 | 0,0234 |
| A\_51\_P101065 | NM\_008315 | Mus musculus 5-hydroxytryptamine (serotonin) receptor 7 (Htr7), mRNA | 0.79 | 0,0436 |
| A\_51\_P414442 | XM\_132099 | hypothetical SAM domain (Sterile alpha motif)/Pleckstrin homology (PH) domain containing protein | 0.79 | 0,0317 |
| A\_51\_P314830 | NM\_011306 | Mus musculus retinoid X receptor beta (Rxrb), mRNA | 0.79 | 0,0399 |
| A\_51\_P269045 | A\_51\_P269045 | Mus musculus RIKEN cDNA 4930517G15 gene (4930517G15Rik), mRNA | 0.78 | 0,0362 |
| A\_51\_P231820 | AK047983 | hypothetical Sp100 domain containing protein | 0.78 | 0,0336 |
| A\_51\_P375285 | AK083796 | weakly similar to ENVELOPE PROTEIN (FRAGMENT) [Friend spleen focus-forming virus] | 0.78 | 0,0283 |
| A\_51\_P381060 | NM\_133209 | Mus musculus immunoglobulin-like cell surface receptor FDFACT, activating counterpart (FDFACT), mRNA | 0.78 | 0,0458 |
| A\_51\_P385639 | NM\_010291 | Mus musculus gap junction membrane channel protein beta 5 (Gjb5), mRNA | 0.78 | 0,0360 |
| A\_51\_P222071 | NM\_153088 | Mus musculus golli-interacting protein mRNA, complete cds | 0.78 | 0,0434 |
| A\_51\_P311785 | NM\_008550 | Mus musculus mannosidase 2, alpha B2 (Man2b2), mRNA | 0.78 | 0,0127 |
| A\_51\_P385478 | AK019589 | hypothetical protein | 0.78 | 0,0150 |
| A\_51\_P389004 | NM\_011891 | Mus musculus sarcoglycan, delta (35kD dystrophin-associated glycoprotein) (Sgcd), mRNA | 0.78 | 0,0197 |
| A\_51\_P386189 | NM\_016788 | Mus musculus tyrosine kinase, non-receptor, 2 (Tnk2), mRNA | 0.78 | 0,0258 |
| A\_51\_P190604 | NM\_134010 | Mus musculus, clone IMAGE:3497824, mRNA, partial cds | 0.78 | 0,0332 |
| A\_51\_P139751 | U26472 | Mus musculus nucleosome-reactive monoclonal antibody MGC23, Ig light chain variable region mRNA, partial cds | 0.78 | 0,0289 |
| A\_51\_P512899 | NM\_007551 | Mus musculus Burkitt lymphoma receptor 1 (Blr1), mRNA | 0.78 | 0,0235 |
| A\_51\_P366770 | NM\_053270 | Mus musculus partial mRNA for Rim, splice variant 1 | 0.78 | 0,0339 |
| A\_51\_P274907 | AK029583 | Rab6-interacting protein 2 | 0.78 | 0,0333 |
| A\_51\_P328060 | NM\_019929 | Mus musculus SMT3 (supressor of mif two, 3) homolog 1 (S. cerevisiae) (Smt3h1), mRNA | 0.78 | 0,0348 |
| A\_51\_P173741 | NM\_009595 | Mus musculus tyrosine kinase (arg) mRNA, partial cds | 0.78 | 0,0198 |
| A\_51\_P412508 | NM\_018885 | Mus musculus iroquois related homeobox 4 (Drosophila) (Irx4), mRNA | 0.78 | 0,0179 |
| A\_51\_P380239 | BC014761 | Mus musculus, Similar to leucine rich repeat (in FLII) interacting protein 2, clone MGC:25637 IMAGE:4217995, mRNA, complete cds | 0.78 | 0,0147 |
| A\_51\_P293753 | NM\_025611 | Mus musculus RIKEN cDNA 2510004L20 gene (2510004L20Rik), mRNA | 0.78 | 0,0206 |
| A\_51\_P440743 | NM\_009886 | Mus musculus cadherin EGF LAG seven-pass G-type receptor 1 (Celsr1), mRNA | 0.78 | 0,0182 |
| A\_51\_P499673 | NM\_025401 | Mus musculus ubiquitin-like 5 (Ubl5), mRNA | 0.78 | 0,0265 |
| A\_51\_P115346 | NM\_011028 | Mus musculus purinergic receptor P2X-like 1, orphan receptor (P2rxl1), mRNA | 0.78 | 0,0257 |
| A\_51\_P358908 | NM\_133671 | Mus musculus U2 small nuclear ribonucleoprotein auxiliary factor (U2AF), 65 kDa (U2af2), mRNA | 0.78 | 0,0479 |
| A\_51\_P118255 | NM\_012045 | Mus musculus phospholipase A2, group IIF (Pla2g2f), mRNA | 0.78 | 0,0305 |
| A\_51\_P492830 | NM\_021886 | Mus musculus centromere autoantigen H (Cenph), mRNA | 0.78 | 0,0175 |
| A\_51\_P346608 | A\_51\_P346608 | Mus musculus meiosis-specific nuclear structural protein 1 (Mns1), mRNA | 0.78 | 0,0287 |
| A\_51\_P408269 | NM\_013547 | Mus musculus homogentisate 1, 2-dioxygenase (Hgd), mRNA | 0.78 | 0,0098 |
| A\_51\_P272066 | NM\_025929 | Mus musculus RIKEN cDNA 2010109I03 gene (2010109I03Rik), mRNA | 0.78 | 0,0463 |
| A\_51\_P127695 | NM\_015764 | Mus musculus RIKEN cDNA 5730583K22 gene (5730583K22Rik), mRNA | 0.78 | 0,0244 |
| A\_51\_P237418 | AK014752 | hypothetical protein | 0.77 | 0,0274 |
| A\_51\_P412391 | NM\_011998 | Mus musculus carbohydrate (chondroitin 6/keratan) sulfotransferase 4 (Chst4), mRNA | 0.77 | 0,0200 |
| A\_51\_P390531 | NM\_172663 | ENHANCER OF POLYCOMB 1 homolog [Homo sapiens] | 0.77 | 0,0270 |
| A\_51\_P175146 | NM\_027769 | copine III | 0.77 | 0,0348 |
| A\_51\_P435497 | NM\_008637 | Mus musculus nudix (nucleoside diphosphate linked moiety X)-type motif 1 (Nudt1), mRNA | 0.77 | 0,0386 |
| A\_51\_P433157 | XM\_284439 | similar to DJ620E11.1.1 (NOVEL HELICASE C-TERMINAL DOMAIN AND SNF2 N-TERMINAL DOMAINS CONTAINING PROTEIN, SIMILAR TO KIAA0308 (ISOFORM 1)) (FRAGMENT) [Homo sapiens] | 0.77 | 0,0464 |
| A\_51\_P476008 | NM\_013854 | Mus musculus ATP-binding cassette protein (Abcf1) mRNA, partial cds | 0.77 | 0,0228 |
| A\_51\_P151182 | NM\_011940 | Mus musculus interferon activated gene 202A (Ifi202a), mRNA | 0.77 | 0,0243 |
| A\_51\_P454286 | XM\_198225 | MYOSIN VC (MYOSIN 5C) homolog [Homo sapiens] | 0.77 | 0,0287 |
| A\_51\_P175842 | NM\_013528 | Mus musculus glutamine fructose-6-phosphate transaminase 1 (Gfpt1), mRNA | 0.77 | 0,0332 |
| A\_51\_P185941 | NM\_019658 | Mus musculus soc-2 (suppressor of clear) homolog (C. elegans) (Shoc2), mRNA | 0.77 | 0,0300 |
| A\_51\_P138648 | NM\_010562 | Mus musculus integrin linked kinase (Ilk), mRNA | 0.77 | 0,0277 |
| A\_51\_P150698 | NM\_010177 | Mus musculus tumor necrosis factor (ligand) superfamily, member 6 (Tnfsf6), mRNA | 0.77 | 0,0271 |
| A\_51\_P441080 | AK087712 | hypothetical Phosphoglycerate mutase-like structure containing protein | 0.77 | 0,0382 |
| A\_51\_P227261 | NM\_033041 | Mus musculus hairy and enhancer of split 7 (Drosophila) (Hes7), mRNA | 0.77 | 0,0346 |
| A\_51\_P258938 | NM\_172270 | Mus musculus hypothetical protein MGC25852 (MGC25852), mRNA | 0.77 | 0,0118 |
| A\_51\_P205035 | AK042573 | hypothetical protein | 0.77 | 0,0361 |
| A\_51\_P377528 | NM\_011011 | Mouse kappa opioid receptor mRNA, complete cds | 0.77 | 0,0261 |
| A\_51\_P372156 | AK044443 | hypothetical protein | 0.77 | 0,0120 |
| A\_51\_P103645 | NM\_031384 | Mus musculus testis expressed gene 11 (Tex11), mRNA | 0.77 | 0,0320 |
| A\_51\_P291741 | NM\_021337 | Mus musculus superkiller viralicidic activity 2-like (S. cerevisiae ) (Skiv2l), mRNA | 0.77 | 0,0236 |
| A\_51\_P119749 | BB096820 | CDNA FLJ12921 FIS, CLONE NT2RP2004600 homolog [Homo sapiens] | 0.77 | 0,0225 |
| A\_51\_P316042 | NM\_175362 | CARD-CONTAINING MAGUK PROTEIN CARMA1 homolog [Homo sapiens] | 0.77 | 0,0064 |
| A\_51\_P502608 | NM\_133784 | Mus musculus RIKEN cDNA 2310058J06 gene (2310058J06Rik), mRNA | 0.77 | 0,0161 |
| A\_51\_P507290 | NM\_009769 | Mus musculus Kruppel-like factor 5 (Klf5), mRNA | 0.77 | 0,0241 |
| A\_51\_P125265 | NM\_008091 | Mus musculus GATA binding protein 3 (Gata3), mRNA | 0.77 | 0,0305 |
| A\_51\_P210990 | AK015714 | brain protein 44-like | 0.77 | 0,0369 |
| A\_51\_P473051 | AK089344 | arachidonate 5-lipoxygenase | 0.77 | 0,0206 |
| A\_51\_P268797 | NM\_134224 | Mus musculus vomeronasal 1 receptor, I7 (V1ri7), mRNA | 0.77 | 0,0133 |
| A\_51\_P135622 | NM\_010089 | Mus musculus deubiquitinating enzyme 2 (Dub2), mRNA | 0.77 | 0,0356 |
| A\_51\_P468071 | NM\_008116 | Mus musculus gamma-glutamyl transpeptidase (Ggtp), mRNA | 0.77 | 0,0333 |
| A\_51\_P108309 | NM\_146990 | Mus musculus olfactory receptor MOR266-1 (MOR266-1), mRNA | 0.77 | 0,0308 |
| A\_51\_P438399 | NM\_010049 | unknown EST | 0.77 | 0,0089 |
| A\_51\_P384187 | NM\_011339 | Mus musculus small inducible cytokine subfamily B, member 15 (Scyb15), mRNA | 0.77 | 0,0187 |
| A\_51\_P408249 | AK013187 | unknown EST | 0.77 | 0,0119 |
| A\_51\_P469531 | NM\_019652 | Mus musculus arsA (bacterial) arsenite transporter, ATP-binding, homolog 1 (Asna1), mRNA | 0.77 | 0,0100 |
| A\_51\_P307220 | NM\_144849 | DJ466N1.4 (NOVEL PROTEIN SIMILAR TO ANK3 (ANKYRIN 3, NODE OF RANVIER (ANKYRIN G))) (HYPOTHETICAL 32.5 KDA PROTEIN) homolog [Homo sapiens] | 0.77 | 0,0436 |
| A\_51\_P308948 | XM\_132806 | similar to HYPOTHETICAL 35.9 KDA PROTEIN [Homo sapiens] | 0.77 | 0,0431 |
| A\_51\_P203827 | NM\_007419 | Mus musculus adrenergic receptor, beta 1 (Adrb1), mRNA | 0.77 | 0,0485 |
| A\_51\_P221014 | NM\_010581 | Mus musculus integrin-associated protein (Itgp), mRNA | 0.77 | 0,0455 |
| A\_51\_P463379 | NM\_007925 | Mus musculus elastin (Eln), mRNA | 0.77 | 0,0268 |
| A\_51\_P222252 | AF001293 | Mus musculus transcription factor aiolos mRNA, partial cds | 0.77 | 0,0496 |
| A\_51\_P515412 | NAP002111-001 | ZINC FINGER | 0.77 | 0,0458 |
| A\_51\_P217697 | AK006127 | hypothetical protein | 0.77 | 0,0270 |
| A\_51\_P169567 | NM\_010909 | Mus musculus nuclear factor of kappa light polypeptide gene enhancer in B-cells inhibitor-like 1 (Nfkbil1), mRNA | 0.77 | 0,0359 |
| A\_51\_P213765 | AF177144 | Mus musculus inositol hexaphosphate kinase 1 (Ihpk1), mRNA | 0.77 | 0,0348 |
| A\_51\_P461902 | L22886 | Mus musculus rearranged IgH mRNA, V-region, cell line Cyd-1 | 0.77 | 0,0127 |
| A\_51\_P170285 | NM\_030255 | Mus musculus hypothetical protein, MGC:7002 (BC003314), mRNA | 0.77 | 0,0255 |
| A\_51\_P386970 | NAP108738-1 | Mus musculus olfactory receptor MOR31-14 (MOR31-14) pseudogene | 0.76 | 0,0293 |
| A\_51\_P169415 | NM\_133167 | Mus musculus parvin, beta (Parvb), mRNA | 0.76 | 0,0122 |
| A\_51\_P483329 | NM\_172713 | weakly similar to MYSTERY 45A [Drosophila melanogaster] | 0.76 | 0,0229 |
| A\_51\_P358683 | NM\_183031 | EBV-INDUCED G PROTEIN-COUPLED RECEPTOR 2 (EBI2) homolog [Homo sapiens] | 0.76 | 0,0380 |
| A\_51\_P219025 | NM\_026898 | hypothetical Trp-Asp (WD) repeats profile/Trp-Asp (WD) repeats circular profile/G-protein beta WD-40 repeats containing protein | 0.76 | 0,0164 |
| A\_51\_P462385 | NM\_008061 | Mus musculus glucose-6-phosphatase, catalytic (G6pc), mRNA | 0.76 | 0,0240 |
| A\_51\_P205779 | NM\_009690 | Mus musculus apoptosis inhibitory 6 (Api6), mRNA | 0.76 | 0,0061 |
| A\_51\_P334344 | AK047044 | Mus musculus mRNA similar to CGI-72 protein (cDNA clone MGC:28361 IMAGE:4019308), complete cds | 0.76 | 0,0065 |
| A\_51\_P486207 | AK032580 | weakly similar to REVERSE TRANSCRIPTASE (FRAGMENT) [Sheep pulmonary adenomatosis virus] | 0.76 | 0,0425 |
| A\_51\_P507709 | NM\_173772 | SIALIDASE 3 EC 3.2.1.18 MEMBRANE SIALIDASE GANGLIOSIDE SIALIDASE N ACETYL ALPHA NEURAMINIDASE | 0.76 | 0,0106 |
| A\_51\_P406306 | NM\_172655 | hypothetical C2 domain/C2-domain profile containing protein | 0.76 | 0,0138 |
| A\_51\_P421780 | AK077135 | hypothetical Serine-rich region containing protein | 0.76 | 0,0384 |
| A\_51\_P121485 | NM\_146625 | OLFACTORY RECEPTOR | 0.76 | 0,0065 |
| A\_51\_P475883 | XM\_622891 | hypothetical protein | 0.76 | 0,0172 |
| A\_51\_P309661 | AK082667 | hypothetical Serine-rich region/Treacher Collins syndrome protein Treacle containing protein | 0.76 | 0,0309 |
| A\_51\_P419389 | NM\_007561 | Mus musculus bone morphogenic protein receptor, type II (serine/threonine kinase) (Bmpr2), mRNA | 0.76 | 0,0417 |
| A\_51\_P299860 | AK007281 | hypothetical protein | 0.76 | 0,0351 |
| A\_51\_P453286 | NM\_177150 | hypothetical Histone-fold structure containing protein | 0.76 | 0,0336 |
| A\_51\_P318856 | NM\_145935 | Mus musculus, clone MGC:18871 IMAGE:4234793, mRNA, complete cds | 0.76 | 0,0392 |
| A\_51\_P378387 | XM\_355790 | weakly similar to RSEC15 [Rattus norvegicus] | 0.76 | 0,0246 |
| A\_51\_P315941 | AK016167 | Mus musculus RIKEN cDNA 4930557O20 gene (4930557O20Rik), mRNA | 0.76 | 0,0162 |
| A\_51\_P425372 | NM\_028047 | CDNA FLJ12886 FIS, CLONE NT2RP2004041, WEAKLY SIMILAR TO SYNAPSINS IA AND IB homolog [Homo sapiens] | 0.76 | 0,0321 |
| A\_51\_P473509 | NM\_026423 | Mus musculus RIKEN cDNA 2410018C20 gene (2410018C20Rik), mRNA | 0.76 | 0,0306 |
| A\_51\_P320304 | NM\_011122 | Mus musculus procollagen-lysine, 2-oxoglutarate 5-dioxygenase 1 (Plod1), mRNA | 0.76 | 0,0312 |
| A\_51\_P391432 | NM\_145449 | Mus musculus, Similar to TLH29 protein precursor, clone MGC:25891 IMAGE:4217067, mRNA, complete cds | 0.76 | 0,0127 |
| A\_51\_P489003 | AK129355 | calcium-activated potassium channel protein homolog [Rattus norvegicus] | 0.76 | 0,0261 |
| A\_51\_P310949 | NM\_008911 | Mus musculus protoporphyrinogen oxidase (Ppox), mRNA | 0.76 | 0,0043 |
| A\_51\_P296535 | NM\_007734 | Mus musculus procollagen, type IV, alpha 3 (Col4a3), mRNA | 0.76 | 0,0154 |
| A\_51\_P407915 | AK040806 | similar to COMPLEMENT COMPONENT C7 PRECURSOR [Homo sapiens] | 0.76 | 0,0350 |
| A\_51\_P480858 | NM\_021453 | Mus musculus pepsinogen F (Pepf-pending), mRNA | 0.76 | 0,0146 |
| A\_51\_P311576 | NM\_052994 | Mus musculus sparc/osteonectin, cwcv and kazal-like domains proteoglycan 2 (Spock2), mRNA | 0.76 | 0,0187 |
| A\_51\_P131277 | NM\_172857 | hypothetical 3-5 exonuclease containing protein | 0.76 | 0,0172 |
| A\_51\_P352216 | NM\_178679 | similar to KIAA0844 PROTEIN [Homo sapiens] | 0.76 | 0,0326 |
| A\_51\_P302488 | NM\_027426 | hypothetical protein | 0.76 | 0,0412 |
| A\_51\_P234245 | AK081677 | Mus musculus cDNA, 3 end | 0.76 | 0,0137 |
| A\_51\_P187716 | AK003350 | unclassifiable | 0.76 | 0,0188 |
| A\_51\_P204121 | AK086019 | weakly similar to NUCLEOSIDE DIPHOSPHATE-LINKED MOIETY X MOTIF 6 (PROTEIN GFG) [Rattus norvegicus] | 0.76 | 0,0277 |
| A\_51\_P370470 | AK053388 | similar to CYTOCHROME P450 MONOOXYGENASE [Homo sapiens] | 0.76 | 0,0466 |
| A\_51\_P102106 | NM\_146364 | Mus musculus olfactory receptor MOR204-37 (MOR204-37), mRNA | 0.76 | 0,0103 |
| A\_51\_P346153 | NM\_009720 | Mus musculus ATX1 (antioxidant protein 1) homolog 1 (yeast) (Atox1), mRNA | 0.76 | 0,0308 |
| A\_51\_P448608 | NM\_008738 | Mus musculus neurturin (Nrtn), mRNA | 0.76 | 0,0202 |
| A\_51\_P364714 | NM\_172203 | Mus musculus NADPH oxidase 1 (Nox1), mRNA | 0.76 | 0,0157 |
| A\_51\_P184680 | NM\_011560 | Mus musculus t-complex-associated testis expressed 3 (Tcte3), mRNA | 0.76 | 0,0403 |
| A\_51\_P329094 | BC087920 | Mus musculus RIKEN cDNA 4930550L24 gene (4930550L24Rik), mRNA | 0.76 | 0,0081 |
| A\_51\_P454736 | NM\_009612 | Mus musculus activin A receptor, type II-like 1 (Acvrl1), mRNA | 0.76 | 0,0317 |
| A\_51\_P352532 | NM\_023835 | Mus musculus tripartite motif protein 12 (Trim12), mRNA | 0.76 | 0,0277 |
| A\_51\_P359272 | NM\_009655 | Mus musculus activated leukocyte cell adhesion molecule CD166 (ALCAM) mRNA, complete cds | 0.76 | 0,0143 |
| A\_51\_P295210 | NM\_178309 | similar to BRCA1-BINDING HELICASE-LIKE PROTEIN BACH1 [Homo sapiens] | 0.76 | 0,0112 |
| A\_51\_P160083 | AK005363 | hypothetical protein | 0.76 | 0,0290 |
| A\_51\_P205326 | NM\_177743 | hypothetical protein | 0.76 | 0,0429 |
| A\_51\_P398647 | NM\_175558 | weakly similar to SIMILAR TO HYPOTHETICAL PROTEIN FLJ20626 [Homo sapiens] and weakly similar to ZINC FINGER 202 M3 SPLICE VARIANT [Mus musculus] | 0.76 | 0,0435 |
| A\_51\_P357696 | AK013342 | musculus 10, 11 days embryo whole body cDNA, RIKEN full-length enriched library, clone:2810454F19:hypothetical protein, full insert sequence | 0.76 | 0,0391 |
| A\_51\_P443279 | AK076351 | unknown EST | 0.76 | 0,0352 |
| A\_51\_P235345 | XM\_283804 | hypothetical protein | 0.76 | 0,0323 |
| A\_51\_P355617 | AK002346 | CASPASE RECRUITMENT DOMAIN PROTEIN 11 (CARD-CONTAINING MAGUK PROTEIN 3) (CARMA 1) homolog [Homo sapiens] | 0.76 | 0,0408 |
| A\_51\_P230634 | AK015141 | unknown EST | 0.76 | 0,0071 |
| A\_51\_P127976 | NM\_026025 | MADP-1 PROTEIN homolog [Homo sapiens] | 0.76 | 0,0170 |
| A\_51\_P477716 | NM\_153112 | Mus musculus membrane glycoprotein (Tsll2) mRNA, complete cds | 0.76 | 0,0122 |
| A\_51\_P191463 | AY302216 | MHC NONCLASSICAL CLASS I THY19.4 GENE (H-2 D HAPLOTYPE) (FRAGMENT) | 0.76 | 0,0481 |
| A\_51\_P348154 | NM\_146448 | OLFACTORY RECEPTOR | 0.75 | 0,0201 |
| A\_51\_P103450 | XM\_130219 | weakly similar to N-ACETYLLACTOSAMINIDE ALPHA-1,3-GALACTOSYLTRANSFERASE (EC 2.4.1.151) (GALACTOSYLTRANSFERASE) (UDP-GALACTOSE:BETA-D-GALACTOSYL-1,4-N-ACETYL- D-GLUCOSAMINIDE ALPHA-1,3-GALACTOSYLTRANSFERASE) [Bos taurus] | 0.75 | 0,0295 |
| A\_51\_P252157 | NM\_011623 | Mus musculus topoisomerase (DNA) II alpha (Top2a), mRNA | 0.75 | 0,0100 |
| A\_51\_P484880 | NM\_207680 | Mus musculus BCL2-like 11 (apoptosis facilitator) (Bcl2l11), mRNA | 0.75 | 0,0062 |
| A\_51\_P304490 | NM\_206896 | Mus musculus olfactory receptor MOR208-5 (MOR208-5) pseudogene | 0.75 | 0,0435 |
| A\_51\_P256224 | NM\_146202 | similar to ZINC FINGER PROTEIN 11 (FRAGMENT) [Rattus norvegicus] | 0.75 | 0,0177 |
| A\_51\_P267861 | NM\_021542 | Mus musculus potassium channel, subfamily K, member 5 (Kcnk5), mRNA | 0.75 | 0,0348 |
| A\_51\_P445479 | NM\_013524 | Mus musculus fucosyltransferase 7 (Fut7), mRNA | 0.75 | 0,0471 |
| A\_51\_P362348 | NM\_020280 | Mus musculus melanoma antigen, family A, 4 (Magea4), mRNA | 0.75 | 0,0060 |
| A\_51\_P376883 | NM\_029601 | hypothetical protein | 0.75 | 0,0282 |
| A\_51\_P445726 | NM\_011095 | Mus musculus immunoglobulin-like receptor PIRB5 (6M1) mRNA, complete cds | 0.75 | 0,0484 |
| A\_51\_P394207 | NM\_007986 | Mus musculus fibroblast activation protein (Fap), mRNA | 0.75 | 0,0192 |
| A\_51\_P326573 | AK043719 | hypothetical KRAB box containing protein | 0.75 | 0,0191 |
| A\_51\_P284716 | AK029593 | weakly similar to CDNA: FLJ23584 FIS, CLONE LNG14307 (HYPOTHETICAL 25.7 KDA PROTEIN) [Homo sapiens] | 0.75 | 0,0177 |
| A\_51\_P148421 | NM\_029082 | Mus musculus RIKEN cDNA 5830411J07 gene (5830411J07Rik), mRNA | 0.75 | 0,0229 |
| A\_51\_P324535 | NM\_019835 | Mus musculus UDP-Gal:betaGlcNAc beta 1,4-galactosyltransferase, polypeptide 5 (B4galt5), mRNA | 0.75 | 0,0210 |
| A\_51\_P183746 | NM\_009116 | Mus musculus paired related homeobox 2 (Prrx2), mRNA | 0.75 | 0,0101 |
| A\_51\_P506915 | NM\_019762 | Mus musculus plakophilin 3 (Pkp3), mRNA | 0.75 | 0,0072 |
| A\_51\_P324871 | NM\_201351 | unknown | 0.75 | 0,0163 |
| A\_51\_P444162 | NM\_144939 | Mus musculus, Similar to suc1-associated neurotrophic factor target 2 (FGFR signalling adaptor), clone MGC:25496 IMAGE:4506982, mRNA, complete cds | 0.75 | 0,0112 |
| A\_51\_P305262 | XM\_485782 | Mouse mRNA for Ig C.C58 M75 kappa light chain (VK Ser-group) | 0.75 | 0,0294 |
| A\_51\_P195935 | BC067054 | hypothetical Serine-rich region containing protein | 0.75 | 0,0417 |
| A\_51\_P333349 | NM\_172541 | TRANSMEMBRANE PROTEIN INDUCED BY TUMOR NECROSIS FACTOR ALPHA homolog [Homo sapiens] | 0.75 | 0,0357 |
| A\_51\_P418725 | NM\_024413 | Mus musculus, clone MGC:6627 IMAGE:3491671, mRNA, complete cds | 0.75 | 0,0320 |
| A\_51\_P191290 | AK015098 | inferred: RIKEN cDNA 4930405H06 gene / putative [Mus musculus] | 0.75 | 0,0479 |
| A\_51\_P462271 | NM\_007424 | Mus musculus aggrecan 1 (Agc1), mRNA | 0.75 | 0,0224 |
| A\_51\_P188155 | NM\_080644 | Mus musculus voltage-dependent calcium channel gamma-5 subunit (Cacng5) mRNA, complete cds | 0.75 | 0,0131 |
| A\_51\_P144180 | NM\_010510 | Mus musculus interferon beta, fibroblast (Ifnb), mRNA | 0.75 | 0,0081 |
| A\_51\_P259445 | AK007933 | hypothetical protein | 0.75 | 0,0312 |
| A\_51\_P463878 | NM\_011795 | Mus musculus C1q related factor (C1qrf-pending), mRNA | 0.75 | 0,0403 |
| A\_51\_P346243 | NM\_010326 | Mus musculus glycoprotein 1b, alpha polypeptide (Gp1ba), mRNA | 0.75 | 0,0472 |
| A\_51\_P321930 | AK002366 | hypothetical protein | 0.75 | 0,0182 |
| A\_51\_P499530 | NM\_133971 | Mus musculus expressed sequence AW549277 (AW549277), mRNA | 0.75 | 0,0364 |
| A\_51\_P321391 | NM\_008810 | Mus musculus pyruvate dehydrogenase E1 alpha 1 (Pdha1), mRNA | 0.75 | 0,0082 |
| A\_51\_P152670 | AF357512 | Mus musculus clone MBII-223 miscellaneous RNA, partial sequence | 0.75 | 0,0084 |
| A\_51\_P227242 | NM\_031381 | Mus musculus testis expressed gene 13 (Tex13), mRNA | 0.75 | 0,0214 |
| A\_51\_P344531 | NM\_027660 | similar to CDNA FLJ32828 FIS, CLONE TESTI2003117, WEAKLY SIMILAR TO TEKTIN A1 [Homo sapiens] | 0.75 | 0,0469 |
| A\_51\_P390117 | NM\_023256 | keratin 21, type I, cytoskeletal homolog [Rattus norvegicus] | 0.75 | 0,0260 |
| A\_51\_P172054 | NM\_019521 | Mus musculus growth arrest specific 6 (Gas6), mRNA | 0.75 | 0,0193 |
| A\_51\_P223489 | AK040351 | unclassifiable | 0.75 | 0,0030 |
| A\_51\_P239166 | NM\_020330 | Mus musculus a disintegrin and metalloprotease domain 21 (Adam21), mRNA | 0.75 | 0,0171 |
| A\_51\_P269553 | NM\_010439 | Mus musculus high mobility group box 1 (Hmgb1), mRNA | 0.75 | 0,0435 |
| A\_51\_P446978 | NM\_011801 | Mus musculus craniofacial development protein 1 (Cfdp), mRNA | 0.75 | 0,0181 |
| A\_51\_P364841 | NM\_029529 | hypothetical protein | 0.75 | 0,0208 |
| A\_51\_P297011 | NM\_028243 | similar to LYSOSOMAL PRO-X CARBOXYPEPTIDASE PRECURSOR (EC 3.4.16.2) (PROLYLCARBOXYPEPTIDASE) (PRCP) (PROLINE CARBOXYPEPTIDASE) (ANGIOTENSINASE C) (LYSOSOMAL CARBOXYPEPTIDASE C) [Homo sapiens] | 0.75 | 0,0204 |
| A\_51\_P272681 | AK044390 | unknown EST | 0.75 | 0,0157 |
| A\_51\_P193711 | NM\_181423 | ATP-DEPENDENT MITOCHONDRIAL RNA HELICASE homolog [Homo sapiens] | 0.75 | 0,0270 |
| A\_51\_P187444 | NM\_011242 | Mus musculus RAS, guanyl releasing protein 2 (Rasgrp2), mRNA | 0.75 | 0,0059 |
| A\_51\_P186510 | NM\_008759 | Mus musculus OG9 homeobox gene (Og9x), mRNA | 0.75 | 0,0216 |
| A\_51\_P496549 | NM\_012009 | Mus musculus EWS/FLI1 activated transcript 2 (Eat2), mRNA | 0.75 | 0,0222 |
| A\_51\_P436530 | NM\_008971 | Mus musculus protein tyrosine kinase 9 (Ptk9), mRNA | 0.75 | 0,0427 |
| A\_51\_P463452 | NM\_007981 | Mus musculus fatty acid Coenzyme A ligase, long chain 2 (Facl2), mRNA | 0.74 | 0,0119 |
| A\_51\_P464420 | AK014845 | hypothetical protein | 0.74 | 0,0077 |
| A\_51\_P499923 | NM\_213733 | Mus musculus cDNA sequence BC023239, mRNA (cDNA clone IMAGE:4207154), partial cds | 0.74 | 0,0405 |
| A\_51\_P204004 | NM\_177112 | POLYHOMEOTIC | 0.74 | 0,0303 |
| A\_51\_P367934 | NM\_130877 | Mus musculus mRNA for Edr protein | 0.74 | 0,0269 |
| A\_51\_P294328 | NM\_172727 | hypothetical protein | 0.74 | 0,0389 |
| A\_51\_P230537 | XM\_133435 | Mus musculus, clone IMAGE:4216925, mRNA, partial cds | 0.74 | 0,0344 |
| A\_51\_P381015 | NM\_007966 | Mus musculus even skipped homeotic gene 1 homolog (Evx1), mRNA | 0.74 | 0,0201 |
| A\_51\_P477093 | NM\_021322 | Mus musculus WD repeat domain 4 (Wdr4), mRNA | 0.74 | 0,0414 |
| A\_51\_P300038 | NM\_027570 | weakly similar to PUTATIVE D-LACTATE DEHYDROGENASE (CYTOCHROME) OXIDOREDUCTASE PROTEIN (EC 1.1.2.4) [Ralstonia solanacearum] | 0.74 | 0,0163 |
| A\_51\_P108901 | NM\_023731 | Mus musculus brain cDNA, clone MNCb-4327 | 0.74 | 0,0152 |
| A\_51\_P330353 | NM\_026226 | POLY A BINDING PROTEIN, CYTOPLASMIC 1 homolog [Mus musculus] | 0.74 | 0,0154 |
| A\_51\_P154692 | NM\_207157 | Mus musculus olfactory receptor MOR259-3P (MOR259-3P) pseudogene | 0.74 | 0,0441 |
| A\_51\_P490442 | NM\_007478 | Mus musculus ADP-ribosylation factor 3 (Arf3), mRNA | 0.74 | 0,0116 |
| A\_51\_P406835 | AY270177 | hypothetical ARM repeat structure containing protein | 0.74 | 0,0257 |
| A\_51\_P140240 | NM\_009712 | M.musculus mRNA for arylsulfatase B protein | 0.74 | 0,0329 |
| A\_51\_P357313 | NM\_023055 | Mus musculus solute carrier family 9 (sodium/hydrogen exchanger), isoform 3 regulator 2 (Slc9a3r2), mRNA | 0.74 | 0,0296 |
| A\_51\_P508469 | NM\_146833 | Mus musculus olfactory receptor MOR250-3 (MOR250-3), mRNA | 0.74 | 0,0229 |
| A\_51\_P124388 | NM\_145977 | PROSTEIN homolog [Homo sapiens] | 0.74 | 0,0221 |
| A\_51\_P201354 | AK048370 | L1 repeat, Tf subfamily, member 30 | 0.74 | 0,0346 |
| A\_51\_P187121 | NM\_008127 | Mus musculus gap junction membrane channel protein beta 4 (Gjb4), mRNA | 0.74 | 0,0231 |
| A\_51\_P297579 | NM\_008102 | Mus musculus GTP cyclohydrolase 1 (Gch), mRNA | 0.74 | 0,0177 |
| A\_51\_P114037 | AK041084 | unknown EST | 0.74 | 0,0096 |
| A\_51\_P275930 | NM\_194355 | Mus musculus RIKEN cDNA 6030430B19 gene, mRNA (cDNA clone MGC:51665 IMAGE:5318015), complete cds | 0.74 | 0,0094 |
| A\_51\_P432117 | NM\_020513 | Mus musculus gene for odorant receptor MOR10 (Or10), mRNA | 0.74 | 0,0332 |
| A\_51\_P123604 | NM\_172807 | weakly similar to LD39850P [Drosophila melanogaster] | 0.74 | 0,0094 |
| A\_51\_P421094 | BC059917 | similar to ASC-1 COMPLEX SUBUNIT P200 (FRAGMENT) [Homo sapiens] | 0.74 | 0,0267 |
| A\_51\_P330870 | AK016162 | homeodomain interacting protein kinase 1 | 0.74 | 0,0245 |
| A\_51\_P362538 | XM\_143616 | weakly similar to THYRO1000381 PROTEIN (FRAGMENT) [Homo sapiens] | 0.74 | 0,0126 |
| A\_51\_P293789 | NM\_007498 | Mus musculus activating transcription factor 3 (Atf3), mRNA | 0.74 | 0,0225 |
| A\_51\_P294705 | NM\_009981 | Mus musculus phosphate cytidylyltransferase 1, choline, alpha isoform (Pcyt1a), mRNA | 0.74 | 0,0310 |
| A\_51\_P255395 | NM\_007738 | Mus musculus procollagen, type VII, alpha 1 (Col7a1), mRNA | 0.74 | 0,0098 |
| A\_51\_P520066 | NM\_020273 | Mus musculus glucocorticoid modulatory element binding protein 1 (Gmeb1), mRNA | 0.74 | 0,0069 |
| A\_51\_P279050 | NM\_008444 | Mus musculus kinesin family member 3b (Kif3b), mRNA | 0.74 | 0,0126 |
| A\_51\_P194976 | AK004984 | weakly similar to CYTOCHROME P450 MONOOXYGENASE (FRAGMENT) [Brachydanio rerio] | 0.74 | 0,0068 |
| A\_51\_P100519 | AK016018 | hypothetical protein | 0.74 | 0,0393 |
| A\_51\_P279683 | AK005991 | RIKEN cDNA 1700015F03 gene | 0.74 | 0,0392 |
| A\_51\_P384908 | AK011977 | hypothetical protein | 0.74 | 0,0250 |
| A\_51\_P172935 | NM\_026092 | Mus musculus RIKEN cDNA 1700038F02 gene (1700038F02Rik), mRNA | 0.74 | 0,0425 |
| A\_51\_P506813 | NM\_009442 | Mus musculus transcription termination factor 1 (Ttf1), mRNA | 0.74 | 0,0089 |
| A\_51\_P335916 | NM\_023331 | Mus musculus RIKEN cDNA 3110052F15 gene (3110052F15Rik), mRNA | 0.74 | 0,0129 |
| A\_51\_P448545 | AK013012 | hypothetical BTB/POZ domain/Kelch repeat containing protein | 0.74 | 0,0331 |
| A\_51\_P148785 | AK075648 | hypothetical protein | 0.74 | 0,0249 |
| A\_51\_P387845 | AK172909 | Mus musculus, clone IMAGE:3597827, mRNA, partial cds | 0.74 | 0,0270 |
| A\_51\_P199104 | NM\_009813 | Mus musculus calsequestrin 1 (Casq1), mRNA | 0.74 | 0,0139 |
| A\_51\_P161890 | NM\_010189 | Mus musculus Fc receptor, IgG, alpha chain transporter (Fcgrt), mRNA | 0.74 | 0,0367 |
| A\_51\_P106745 | NM\_008363 | Mus musculus interleukin 1 receptor-associated kinase (Il1rak), mRNA | 0.74 | 0,0274 |
| A\_51\_P124895 | AK075665 | weakly similar to DUAL SPECIFICITY PROTEIN PHOSPHATASE 13 (EC 3.1.3.48) (EC 3.1.3.16) (TESTIS- AND SKELETAL-MUSCLE-SPECIFIC DSP) [Homo sapiens] | 0.74 | 0,0242 |
| A\_51\_P275350 | NM\_008960 | Mus musculus phosphatase and tensin homolog (Pten), mRNA | 0.74 | 0,0471 |
| A\_51\_P240136 | NM\_010320 | Mus musculus guanine nucleotide binding protein (G protein), gamma 8 subunit (Gng8), mRNA | 0.74 | 0,0175 |
| A\_51\_P106011 | NM\_133947 | similar to NUCLEAR MITOTIC APPARATUS PROTEIN (FRAGMENT) [Homo sapiens] | 0.74 | 0,0158 |
| A\_51\_P134030 | NM\_145210 | Mus musculus 2-5 oligoadenylate synthetase 1E (Oas1e), mRNA | 0.74 | 0,0055 |
| A\_51\_P104920 | NM\_008895 | pro-opiomelanocortin-alpha | 0.74 | 0,0268 |
| A\_51\_P167713 | BC068151 | WD-CONTAINING PROTEIN (FRAGMENT) homolog [Rattus norvegicus] | 0.74 | 0,0192 |
| A\_51\_P249544 | NM\_029621 | Mus musculus RIKEN cDNA 2410004L22 gene (2410004L22Rik), mRNA | 0.74 | 0,0250 |
| A\_51\_P513071 | NM\_175372 | weakly similar to SD07613P [Drosophila melanogaster] | 0.74 | 0,0289 |
| A\_51\_P291027 | XM\_355528 | hypothetical ARM repeat structure containing protein | 0.74 | 0,0073 |
| A\_51\_P230987 | NM\_025951 | Mus musculus phosphatidylinositol 4-kinase type 2 beta (Pi4k2b-pending), mRNA | 0.74 | 0,0443 |
| A\_51\_P249335 | NM\_145565 | Mus musculus, Similar to serine dehydratase, clone MGC:37901 IMAGE:5102037, mRNA, complete cds | 0.74 | 0,0119 |
| A\_51\_P236074 | NM\_011168 | Mus musculus prolactin-like protein F (Prlpf), mRNA | 0.74 | 0,0375 |
| A\_51\_P245989 | NM\_009915 | Mus musculus chemokine (C-C) receptor 2 (Cmkbr2), mRNA | 0.74 | 0,0329 |
| A\_51\_P354062 | NM\_011197 | Mus musculus prostaglandin F2 receptor negative regulator (Ptgfrn), mRNA | 0.74 | 0,0216 |
| A\_51\_P225232 | NM\_029727 | similar to GASDERMIN [Mus musculus] | 0.74 | 0,0137 |
| A\_51\_P263033 | NM\_010739 | Mus musculus lymphocyte antigen 64 (Ly64), mRNA | 0.74 | 0,0069 |
| A\_51\_P466478 | NM\_011213 | Mus musculus protein tyrosine phosphatase, receptor-type, F (Ptprf), mRNA | 0.74 | 0,0133 |
| A\_51\_P117903 | BE306420 | Mus musculus cDNA clone NIA:K0746F08 IMAGE:30078019 5 | 0.74 | 0,0336 |
| A\_51\_P432678 | NM\_008405 | Mus musculus integrin beta 2-like (Itgb2l), mRNA | 0.74 | 0,0314 |
| A\_51\_P302553 | NAP057030-1 | Mus musculus ubiquitin-conjugating enzyme E2L 3, pseudogene 1 (Ube2l3-ps1) | 0.74 | 0,0376 |
| A\_51\_P103196 | A\_51\_P103196 | Mus musculus cDNA, 3 end | 0.74 | 0,0453 |
| A\_51\_P346679 | XM\_138377 | DNA sequence of a peptide which has a inhibition activity of binding ICAM-1 and LFA-1 | 0.74 | 0,0076 |
| A\_51\_P259773 | NM\_020258 | Mus musculus solute carrier family 37 (glycerol-3-phosphate transporter), member 1 (Slc37a1), mRNA | 0.74 | 0,0110 |
| A\_51\_P415546 | NM\_054074 | Mus musculus defensin beta 6 (Defb6), mRNA | 0.74 | 0,0122 |
| A\_51\_P301499 | AK007620 | weakly similar to NADH-UBIQUINONE OXIDOREDUCTASE 13 KDA-A SUBUNIT, MITOCHONDRIAL PRECURSOR (EC 1.6.5.3) (EC 1.6.99.3) (COMPLEX I-13KD-A) (CI-13KD-A) [Homo sapiens] | 0.74 | 0,0074 |
| A\_51\_P233532 | AK015842 | hypothetical Serine proteases, trypsin family/Chymotrypsin serine protease family (S1) containing protein | 0.74 | 0,0172 |
| A\_51\_P110576 | NM\_011175 | Mus musculus legumain (Lgmn), mRNA | 0.74 | 0,0451 |
| A\_51\_P149699 | NM\_013534 | Mus musculus gene rich cluster, B gene (Grcb), mRNA | 0.74 | 0,0220 |
| A\_51\_P169693 | NM\_198095 | Mus musculus, Similar to bone marrow stromal cell antigen 2, clone MGC:28276 IMAGE:4009434, mRNA, complete cds | 0.74 | 0,0458 |
| A\_51\_P312497 | NM\_009726 | Mus musculus ATPase, Cu++ transporting, alpha polypeptide (Atp7a), mRNA | 0.74 | 0,0105 |
| A\_51\_P444015 | A\_51\_P444015 | PROBABLE UBIQUITIN CARBOXYL-TERMINAL HYDROLASE FAF-X (EC 3.1.2.15) (UBIQUITIN THIOLESTERASE FAF-X) (UBIQUITIN-SPECIFIC PROCESSING PROTEASE FAF-X) (DEUBIQUITINATING ENZYME FAF-X) (FAT FACETS PROTEIN RELATED, X-LINKED) (UBIQUIT | 0.74 | 0,0066 |
| A\_51\_P428977 | BC023444 | musculus, clone MGC:32491 IMAGE:5053834, mRNA, complete cds | 0.74 | 0,0040 |
| A\_51\_P184300 | NM\_010087 | Mus musculus dystrobrevin alpha (Dtna), mRNA | 0.74 | 0,0460 |
| A\_51\_P178705 | NM\_146366 | Mus musculus olfactory receptor MOR179-6 (MOR179-6), mRNA | 0.74 | 0,0407 |
| A\_51\_P445985 | NM\_019415 | Mus musculus solute carrier family 12, member 3 (Slc12a3), mRNA | 0.74 | 0,0183 |
| A\_51\_P377171 | NM\_183264 | hypothetical protein | 0.74 | 0,0321 |
| A\_51\_P131119 | NM\_178697 | similar to CALCIUM-ACTIVATED CHLORIDE CHANNEL-2 [Homo sapiens] | 0.74 | 0,0392 |
| A\_51\_P411345 | NM\_177448 | weakly similar to DIACYLGLYCEROL ACYLTRANSFERASE 2-LIKE PROTEIN [Mus musculus] | 0.74 | 0,0289 |
| A\_51\_P493270 | NM\_029963 | mitochondrial ribosomal protein S5 | 0.74 | 0,0175 |
| A\_51\_P169576 | A\_51\_P169576 | Mus musculus high mobility group box 1, related sequence 16 (Hmgb1-rs16) pseudogene | 0.74 | 0,0069 |
| A\_51\_P170675 | NM\_011386 | Mus musculus ski/sno related (Skir), mRNA | 0.74 | 0,0093 |
| A\_51\_P298455 | NM\_175427 | weakly similar to UNKNOWN (PROTEIN FOR MGC:16664) [Homo sapiens] | 0.74 | 0,0106 |
| A\_51\_P314472 | NM\_139234 | ACTIN-BINDING PROTEIN FRABIN-GAMMA homolog [Mus musculus] | 0.73 | 0,0485 |
| A\_51\_P405074 | AK015402 | hypothetical protein | 0.73 | 0,0347 |
| A\_51\_P143418 | NAP108607-1 | Mus musculus olfactory receptor MOR189-5P (MOR189-5P) pseudogene | 0.73 | 0,0328 |
| A\_51\_P351125 | NM\_013793 | Mus musculus killer cell lectin-like receptor, subfamily A, member 15 (Klra15), mRNA | 0.73 | 0,0239 |
| A\_51\_P201450 | AK014425 | hypothetical Histidine-rich region containing protein | 0.73 | 0,0033 |
| A\_51\_P501622 | NM\_026628 | Mus musculus RIKEN cDNA 1700120K04 gene (1700120K04Rik), mRNA | 0.73 | 0,0147 |
| A\_51\_P375360 | D86421 | Mus musculus keratin associated protein 6-1 (Krtap6-1), mRNA | 0.73 | 0,0236 |
| A\_51\_P477830 | XM\_144142 | hypothetical Galactose oxidase, central domain structure containing protein | 0.73 | 0,0148 |
| A\_51\_P250001 | NM\_027304 | hypothetical protein | 0.73 | 0,0354 |
| A\_51\_P375870 | NM\_198214 | Mus musculus mRNA for mKIAA0374 protein | 0.73 | 0,0371 |
| A\_51\_P193291 | CN839756 | hypothetical IQ calmodulin-binding motif containing protein | 0.73 | 0,0375 |
| A\_51\_P284608 | NM\_010545 | H-2 CLASS II HISTOCOMPATIBILITY ANTIGEN, GAMMA CHAIN (MHC CLASS II(IA) ASSOCIATED INVARIANT CHAIN) | 0.73 | 0,0332 |
| A\_51\_P172947 | AK029607 | hypothetical protein | 0.73 | 0,0383 |
| A\_51\_P283698 | XM\_136682 | erythroblastic leukemia viral oncogene homolog 4 (avian) | 0.73 | 0,0213 |
| A\_51\_P384629 | NM\_009983 | Mus musculus cathepsin D (Ctsd), mRNA | 0.73 | 0,0172 |
| A\_51\_P464691 | NM\_028648 | hypothetical protein | 0.73 | 0,0357 |
| A\_51\_P513741 | AK173242 | Mus musculus cDNA clone IMAGE:6335382 5 | 0.73 | 0,0056 |
| A\_51\_P392967 | NM\_053253 | Mus musculus Blu protein (Blu), mRNA | 0.73 | 0,0260 |
| A\_51\_P476767 | NM\_010924 | Mus musculus nicotinamide N-methyltransferase (Nnmt), mRNA | 0.73 | 0,0161 |
| A\_51\_P436469 | NM\_144821 | similar to DJ1112D6.1 (PUTATIVE NOVEL PROTEIN SIMILAR TO BACTERIAL NARK (NITRITE EXTRUSION PROTEIN, NITRITE FACILITATOR)) (FRAGMENT) [Homo sapiens] | 0.73 | 0,0266 |
| A\_51\_P356883 | NM\_008728 | Mus musculus natriuretic peptide receptor 3 (Npr3), mRNA | 0.73 | 0,0266 |
| A\_51\_P300684 | ENSMUST00000087769 | Mus musculus olfactory receptor GA\_x5J8B7W6KF8-5957071-5957489 (GA\_x5J8B7W6KF8-5957071-5957489) pseudogene | 0.73 | 0,0386 |
| A\_51\_P378754 | NM\_017467 | Mus musculus zinc finger protein 316 (Zfp316), mRNA | 0.73 | 0,0141 |
| A\_51\_P354925 | NM\_008638 | Mus musculus methylenetetrahydrofolate dehydrogenase (NAD+ dependent), methenyltetrahydrofolate cyclohydrolase (Mthfd2), mRNA | 0.73 | 0,0142 |
| A\_51\_P266717 | XM\_125542 | CGI 130 | 0.73 | 0,0258 |
| A\_51\_P506284 | NM\_026131 | Mus musculus RIKEN cDNA 1110003B01 gene (1110003B01Rik), mRNA | 0.73 | 0,0135 |
| A\_51\_P150598 | AK044190 | unclassifiable | 0.73 | 0,0392 |
| A\_51\_P163639 | NM\_027055 | hypothetical protein | 0.73 | 0,0157 |
| A\_51\_P369185 | NM\_028625 | Mus musculus small proline rich-like 2 (Sprrl2), mRNA | 0.73 | 0,0157 |
| A\_51\_P233583 | NM\_019985 | Mus musculus C-type lectin-like receptor 2 (Clec2-pending), mRNA | 0.73 | 0,0277 |
| A\_51\_P268983 | XM\_485520 | hypothetical Immunoglobulin and major histocompatibility complex domain/Immunoglobulin C-2 type/Immunoglobulin subtype containing protein | 0.73 | 0,0321 |
| A\_51\_P343497 | XM\_129647 | "BIFUNCTIONAL AMINOACYL-TRNA SYNTHETASE [INCLUDES: GLUTAMYL-TRNA SYNTHETASE (EC 6.1.1.17) (GLUTAMATE--TRNA LIGASE)� PROLYL-TRNA SYNTHETASE (EC 6.1.1.15) (PROLINE--TRNA LIGASE)], homolog [Homo sapiens]" | 0.73 | 0,0048 |
| A\_51\_P310156 | AK013367 | hypothetical protein | 0.73 | 0,0308 |
| A\_51\_P425205 | AK080908 | hypothetical Cu,Zn superoxide dismutase-like structure containing protein | 0.73 | 0,0364 |
| A\_51\_P437096 | AK083440 | hypothetical protein | 0.73 | 0,0117 |
| A\_51\_P437707 | NM\_019815 | Mus musculus claudin 18 (Cldn18), mRNA | 0.73 | 0,0061 |
| A\_51\_P115601 | X67198 | IG KAPPA CHAIN V-V REGION L7 PRECURSOR (FRAGMENT) | 0.73 | 0,0147 |
| A\_51\_P512017 | NM\_007922 | Mus musculus ELK1, member of ETS oncogene family (Elk1), mRNA | 0.73 | 0,0471 |
| A\_51\_P497484 | NM\_172923 | hypothetical Cation channels (non-ligand gated) containing protein | 0.73 | 0,0034 |
| A\_51\_P467483 | NM\_173443 | Mus musculus, clone IMAGE:3591498, mRNA | 0.73 | 0,0190 |
| A\_51\_P312956 | AK034271 | similar to ORF4 PROTEIN [Rattus norvegicus] | 0.73 | 0,0330 |
| A\_51\_P207324 | NM\_011881 | Mus musculus rhodopsin kinase (Rhok), mRNA | 0.73 | 0,0335 |
| A\_51\_P228892 | NM\_016927 | Mus musculus polycystic kidney disease 2-like 2 (Pkd2l2), mRNA | 0.73 | 0,0235 |
| A\_51\_P291037 | AK129175 | PLEXIN A3 PRECURSOR PLEXIN 4 TRANSMEMBRANE PROTEIN | 0.73 | 0,0403 |
| A\_51\_P245103 | NM\_008021 | Mus musculus forkhead box M1 (Foxm1), mRNA | 0.73 | 0,0101 |
| A\_51\_P240760 | NM\_028189 | Mus musculus UDP-GlcNAc:betaGal beta-1,3-N-acetylglucosaminyltransferase 3 (B3gnt3), mRNA | 0.73 | 0,0361 |
| A\_51\_P349783 | NM\_007388 | Mus musculus acid phosphatase 5, tartrate resistant (Acp5), mRNA | 0.73 | 0,0198 |
| A\_51\_P521374 | NM\_026341 | Mus musculus RIKEN cDNA 4933433B15 gene (4933433B15Rik), mRNA | 0.73 | 0,0274 |
| A\_51\_P308397 | AK006714 | inferred: Mus musculus, Similar to zinc finger protein 135 (clone pHZ-17), clone MGC:7841 IMAGE:3500812, mRNA, complete cds | 0.73 | 0,0072 |
| A\_51\_P477046 | NM\_011903 | Mus musculus tousled-like kinase 2 (Arabidopsis) (Tlk2), mRNA | 0.73 | 0,0204 |
| A\_51\_P152841 | NM\_153557 | hypothetical protein | 0.73 | 0,0016 |
| A\_51\_P410312 | XM\_619801 | Mus musculus mRNA for motor domain of KIF16A, partial cds | 0.73 | 0,0167 |
| A\_51\_P107547 | NM\_147085 | Mus musculus olfactory receptor MOR21-1 (MOR21-1), mRNA | 0.73 | 0,0262 |
| A\_51\_P246345 | NM\_022879 | Mus musculus myosin light chain, regulatory A (Mylc2a), mRNA | 0.73 | 0,0204 |
| A\_51\_P106322 | NM\_134116 | Mus musculus expressed sequence AA960287 (AA960287), mRNA | 0.73 | 0,0426 |
| A\_51\_P334903 | NM\_181665 | SEROLOGICALLY DEFINED COLON CANCER ANTIGEN | 0.73 | 0,0314 |
| A\_51\_P371923 | NM\_011519 | Mus musculus syndecan 1 (Sdc1), mRNA | 0.73 | 0,0238 |
| A\_51\_P272113 | NM\_183123 | hypothetical Kazal-type serine protease inhibitor domain containing protein | 0.73 | 0,0243 |
| A\_51\_P275915 | AY550908 | PROGESTIN INDUCED PROTEIN homolog [Homo sapiens] | 0.73 | 0,0306 |
| A\_51\_P271603 | NM\_019424 | Mus musculus Hermansky-Pudlak syndrome 1 homolog (human) (Hps1), mRNA | 0.73 | 0,0364 |
| A\_51\_P196434 | NM\_026593 | Mus musculus RIKEN cDNA D730048I06 gene (D730048I06Rik), mRNA | 0.73 | 0,0258 |
| A\_51\_P243641 | BC032281 | Mus musculus, clone IMAGE:3989034, mRNA | 0.73 | 0,0090 |
| A\_51\_P135379 | NM\_025703 | Mus musculus RIKEN cDNA 3930402F23 gene (3930402F23Rik), mRNA | 0.73 | 0,0202 |
| A\_51\_P480290 | AK010559 | hypothetical Proline-rich region/Zinc finger, C2H2 type containing protein | 0.73 | 0,0059 |
| A\_51\_P101663 | NM\_025818 | Mus musculus RIKEN cDNA 1200014J11 gene (1200014J11Rik), mRNA | 0.73 | 0,0377 |
| A\_51\_P352743 | NM\_001013616 | Mus musculus tripartite motif protein TRIM6 (Trim6) mRNA, partial cds | 0.73 | 0,0499 |
| A\_51\_P113322 | NM\_172896 | Similar to PAN2 protein | 0.73 | 0,0181 |
| A\_51\_P390937 | BF579422 | IG KAPPA CHAIN V-V REGION L6 PRECURSOR (FRAGMENT) | 0.73 | 0,0034 |
| A\_51\_P213981 | NM\_177296 | NUCLEAR TRANSPORT RECEPTOR homolog [Homo sapiens] | 0.73 | 0,0368 |
| A\_51\_P278675 | NM\_009643 | M.musculus mRNA for desmoyokin, partial | 0.73 | 0,0043 |
| A\_51\_P473734 | NM\_010386 | Mus musculus histocompatibility 2, class II, locus DMa (H2-DMa), mRNA | 0.73 | 0,0083 |
| A\_51\_P479181 | NM\_009704 | amphiregulin | 0.73 | 0,0309 |
| A\_51\_P285959 | NM\_018758 | Mus musculus amyloid beta (A4) precursor protein-binding, family A, member 3 (Apba3), mRNA | 0.73 | 0,0173 |
| A\_51\_P394921 | NM\_025492 | RIKEN cDNA 1700020L24 gene | 0.73 | 0,0219 |
| A\_51\_P215496 | NM\_011225 | Mus musculus RAB18, member RAS oncogene family (Rab18), mRNA | 0.73 | 0,0024 |
| A\_51\_P497061 | NM\_026391 | Mus musculus protein phosphatase 2A B regulatory subunit delta isoform mRNA, complete cds | 0.73 | 0,0455 |
| A\_51\_P451316 | NM\_027687 | Mus musculus calcium binding protein CBP86-1 mRNA, complete cds | 0.73 | 0,0279 |
| A\_51\_P333923 | NM\_133681 | Mus musculus RIKEN cDNA 9030418M05 gene (9030418M05Rik), mRNA | 0.73 | 0,0021 |
| A\_51\_P360274 | NM\_052977 | Mus musculus adenosine deaminase 3, RNA dependent (Adar3-pending), mRNA | 0.73 | 0,0073 |
| A\_51\_P277336 | NM\_138741 | Mus musculus serum deprivation response (Sdpr), mRNA | 0.73 | 0,0095 |
| A\_51\_P302167 | NM\_172496 | Mus musculus cordon-bleu mRNA, partial cds | 0.73 | 0,0255 |
| A\_51\_P135117 | NM\_183189 | Mus musculus, similar to RIKEN cDNA 2310057N15, clone MGC:41371 IMAGE:1363815, mRNA, complete cds | 0.73 | 0,0183 |
| A\_51\_P503696 | NM\_174996 | hypothetical protein | 0.72 | 0,0126 |
| A\_51\_P251588 | NM\_026000 | Mus musculus proteasome (prosome, macropain) 26S subunit, non-ATPase, 9 (Psmd9), mRNA | 0.72 | 0,0239 |
| A\_51\_P335151 | XM\_484161 | unknown | 0.72 | 0,0150 |
| A\_51\_P257309 | AK122224 | unknown EST | 0.72 | 0,0087 |
| A\_51\_P345975 | NM\_022883 | Mus musculus lipin 3 (Lpin3), mRNA | 0.72 | 0,0033 |
| A\_51\_P367734 | XM\_129785 | weakly similar to KIAA1092 PROTEIN (FRAGMENT) [Homo sapiens] | 0.72 | 0,0060 |
| A\_51\_P370050 | NM\_013796 | N-ACETYLGLUCOSAMINE-1-PHOSPHODIESTER ALPHA-N-ACETYLGLUCOSAMINIDASE (EC 3.1.4.45) (FRAGMENT) homolog [Mus musculus] | 0.72 | 0,0070 |
| A\_51\_P343147 | AK006280 | weakly similar to PROTEIN PHOSPHATASE INHIBITOR 2 (IPP-2) [Rattus norvegicus] | 0.72 | 0,0398 |
| A\_51\_P321643 | NM\_173371 | "similar to GDH/6PGL ENDOPLASMIC BIFUNCTIONAL PROTEIN PRECURSOR [INCLUDES: GLUCOSE 1-DEHYDROGENASE (EC 1.1.1.47) (HEXOSE-6-PHOSPHATE DEHYDROGENASE)� 6- PHOSPHOGLUCONOLACTONASE (EC 3.1.1.31) (6PGL)] [Homo sapiens]" | 0.72 | 0,0045 |
| A\_51\_P307277 | AI834762 | Mus musculus cDNA, 3 end | 0.72 | 0,0416 |
| A\_51\_P318430 | AF071562 | Mus musculus G substrate (Gsbs-pending), mRNA | 0.72 | 0,0046 |
| A\_51\_P500051 | NM\_019923 | M.musculus mRNA for inositol 1,4,5-trisphosphate receptor (type 2) | 0.72 | 0,0117 |
| A\_51\_P114797 | NM\_172633 | Mus sp. cerebellin 2 (Cbln2) mRNA, partial cds | 0.72 | 0,0085 |
| A\_51\_P391566 | BC002189 | Mus musculus, clone MGC:7386 IMAGE:3487872, mRNA, complete cds | 0.72 | 0,0419 |
| A\_51\_P438537 | NM\_011352 | Mus musculus sema domain, immunoglobulin domain (Ig), and GPI membrane anchor, (semaphorin) 7A (Sema7a), mRNA | 0.72 | 0,0100 |
| A\_51\_P273130 | NM\_030116 | Mus musculus MRPL9 mRNA for mitochondrial ribosomal protein L9 (L9mt), partial cds | 0.72 | 0,0061 |
| A\_51\_P420397 | D16503 | Mus musculus lymphoid enhancer binding factor 1 (Lef1), mRNA | 0.72 | 0,0342 |
| A\_51\_P403443 | NM\_053008 | Mus musculus oligodendrocyte transcription factor 3 (Olig3), mRNA | 0.72 | 0,0485 |
| A\_51\_P419016 | NM\_011866 | Mus musculus phosphodiesterase 10A (Pde10a), mRNA | 0.72 | 0,0354 |
| A\_51\_P274086 | BC058078 | Mus musculus, clone IMAGE:3599662, mRNA, partial cds | 0.72 | 0,0261 |
| A\_51\_P477419 | NM\_026756 | NUCLEAR FACTOR 1 C-TYPE (NUCLEAR FACTOR 1/C) (NF1-C) (NFI-C) (NF-I/C) (CCAAT-BOX BINDING TRANSCRIPTION FACTOR) (CTF) (TGGCA-BINDING PROTEIN) | 0.72 | 0,0046 |
| A\_51\_P177583 | NM\_133691 | Mus musculus RIKEN cDNA 2410104I19 gene (2410104I19Rik), mRNA | 0.72 | 0,0294 |
| A\_51\_P327778 | NM\_011070 | Mus musculus prefoldin 2 (Pfdn2), mRNA | 0.72 | 0,0102 |
| A\_51\_P120201 | NM\_181582 | Mus musculus, eukaryotic translation initiation factor 5A, clone MGC:25474 IMAGE:4482804, mRNA, complete cds | 0.72 | 0,0331 |
| A\_51\_P171533 | AI595224 | hypothetical Lysine-rich region containing protein | 0.72 | 0,0351 |
| A\_51\_P338615 | NM\_007414 | Mus musculus ADP-ribosylarginine hydrolase (Adprh), mRNA | 0.72 | 0,0025 |
| A\_51\_P105709 | AK010336 | thyroid hormone receptor interactor 13 | 0.72 | 0,0217 |
| A\_51\_P107807 | NM\_147111 | Mus musculus olfactory receptor GA\_x5J8B7W5P47-202028-201789 (GA\_x5J8B7W5P47-202028-201789) pseudogene | 0.72 | 0,0050 |
| A\_51\_P158529 | NM\_030689 | Mus musculus neuronal pentraxin receptor (Nptxr), mRNA | 0.72 | 0,0407 |
| A\_51\_P192991 | A\_51\_P192991 | Mus musculus cDNA, 5 end | 0.72 | 0,0032 |
| A\_51\_P312517 | NM\_008058 | Mus musculus frizzled homolog 8 (Drosophila) (Fzd8), mRNA | 0.72 | 0,0027 |
| A\_51\_P161612 | NM\_009831 | Mus musculus cyclin G (Ccng), mRNA | 0.72 | 0,0332 |
| A\_51\_P107738 | NM\_028175 | hypothetical protein | 0.72 | 0,0067 |
| A\_51\_P290974 | XM\_125706 | BCR PROTEIN (FRAGMENT) | 0.72 | 0,0242 |
| A\_51\_P234092 | A\_51\_P234092 | Mus musculus cDNA, 3 end | 0.72 | 0,0311 |
| A\_51\_P223776 | NM\_145434 | SIMILAR TO NUCLEAR RECEPTOR SUBFAMILY 1, GROUP D, MEMBER 1 homolog [Mus musculus] | 0.72 | 0,0309 |
| A\_51\_P292168 | NM\_027698 | Mus musculus RIKEN cDNA 4933424N09 gene (4933424N09Rik), mRNA | 0.72 | 0,0107 |
| A\_51\_P345416 | M64608 | Yamaguchi sarcoma viral (v-yes-1) oncogene homolog | 0.72 | 0,0044 |
| A\_51\_P359983 | NM\_144529 | Mus musculus homolog of rat nadrin (Rich1-pending), mRNA | 0.72 | 0,0055 |
| A\_51\_P482348 | NAP056999-1 | Mus musculus ras homolog A translocated to the Y, pseudogene 3 (Arhay-ps3) on chromosome Y | 0.72 | 0,0139 |
| A\_51\_P498684 | NM\_029756 | similar to ANTIGEN NY-CO-8 (FRAGMENT) [Homo sapiens] | 0.72 | 0,0254 |
| A\_51\_P174591 | NM\_012021 | Mus musculus peroxiredoxin 6 (Prdx6), mRNA | 0.72 | 0,0427 |
| A\_51\_P309530 | NM\_025684 | Mus musculus RIKEN cDNA 5730521E12 gene (5730521E12Rik), mRNA | 0.72 | 0,0127 |
| A\_51\_P465918 | NM\_172721 | weakly similar to F-BOX PROTEIN FBX29 (FRAGMENT) [Homo sapiens] | 0.72 | 0,0009 |
| A\_51\_P390628 | NM\_146247 | Mus musculus, clone MGC:38788 IMAGE:5359405, mRNA, complete cds | 0.72 | 0,0100 |
| A\_51\_P189121 | XM\_486167 | SODIUM/POTASSIUM TRANSPORTING ATPASE ALPHA CHAIN EC 3.6.3.9 SODIUM PUMP NA+/K+ ATPASE | 0.72 | 0,0431 |
| A\_51\_P276404 | NM\_009408 | Mus musculus topoisomerase (DNA) I (Top1), mRNA | 0.72 | 0,0303 |
| A\_51\_P357043 | NM\_177615 | SULFATE TRANSPORTER | 0.72 | 0,0093 |
| A\_51\_P378587 | AJ242954 | Mus musculus partial mRNA for dysferlin (dysf gene) | 0.72 | 0,0431 |
| A\_51\_P133576 | BC036332 | hypothetical Adenine nucleotide alpha hydrolases structure containing protein | 0.72 | 0,0150 |
| A\_51\_P199674 | Y00746 | Mus musculus phosphodiesterase 6G, cGMP-specific, rod, gamma (Pde6g), mRNA | 0.72 | 0,0239 |
| A\_51\_P176487 | NM\_018814 | Mus musculus pecanex homolog (Drosophila) (Pcnx), mRNA | 0.72 | 0,0477 |
| A\_51\_P194785 | U50631 | Mus musculus heat-responsive protein 12 (Hrsp12), mRNA | 0.72 | 0,0166 |
| A\_51\_P377596 | AK078481 | unclassifiable | 0.72 | 0,0173 |
| A\_51\_P334072 | NM\_009989 | Mus musculus cytochrome c, testis (Cyct), mRNA | 0.72 | 0,0332 |
| A\_51\_P246488 | NAP057252-1 | Mus musculus olfactory receptor MOR202-31P (MOR202-31P) pseudogene | 0.72 | 0,0205 |
| A\_51\_P461005 | NM\_013724 | Mus musculus Nik related kinase (Nrk), mRNA | 0.72 | 0,0346 |
| A\_51\_P175367 | AK015669 | hypothetical HMG-I and HMG-Y DNA-binding domain (A+T-hook) containing protein | 0.72 | 0,0279 |
| A\_51\_P288643 | BC006028 | Mus musculus, clone MGC:7391 IMAGE:3487966, mRNA, complete cds | 0.72 | 0,0239 |
| A\_51\_P515908 | NM\_175451 | similar to P63 PROTEIN [Homo sapiens] | 0.72 | 0,0426 |
| A\_51\_P469401 | NM\_009400 | Mus musculus tumor necrosis factor receptor superfamily, member 18 (Tnfrsf18), mRNA | 0.72 | 0,0028 |
| A\_51\_P321331 | NM\_011479 | Mus musculus serine palmitoyltransferase, long chain base subunit 2 (Sptlc2), mRNA | 0.71 | 0,0168 |
| A\_51\_P189361 | NM\_027950 | similar to PREGNANCY-INDUCED GROWTH INHIBITOR [Homo sapiens] | 0.71 | 0,0258 |
| A\_51\_P381397 | XM\_131770 | hypothetical protein | 0.71 | 0,0020 |
| A\_51\_P278353 | AK006745 | hypothetical protein | 0.71 | 0,0157 |
| A\_51\_P329269 | AK041510 | ribonucleotide reductase M2 B (TP53 inducible) | 0.71 | 0,0268 |
| A\_51\_P162553 | AK018093 | hypothetical Pleckstrin putative G-protein interacting domain containing protein | 0.71 | 0,0049 |
| A\_51\_P455620 | NM\_177628 | hypothetical protein | 0.71 | 0,0341 |
| A\_51\_P485203 | NM\_147201 | Mus musculus HLS7-interacting protein kinase mRNA, complete cds | 0.71 | 0,0187 |
| A\_51\_P473409 | NM\_134117 | Mus musculus, clone IMAGE:5134400, mRNA, partial cds | 0.71 | 0,0049 |
| A\_51\_P407074 | NM\_025982 | Mus musculus RIKEN cDNA 2700085A14 gene (2700085A14Rik), mRNA | 0.71 | 0,0129 |
| A\_51\_P343189 | XM\_145298 | weakly similar to PLATELET GLYCOPROTEIN VI PRECURSOR (PLATELET GLYCOPROTEIN VI-1) [Homo sapiens] | 0.71 | 0,0396 |
| A\_51\_P280013 | NM\_028194 | hypothetical protein | 0.71 | 0,0451 |
| A\_51\_P203062 | NM\_010135 | Mus musculus enabled homolog (Drosophila) (Enah), mRNA | 0.71 | 0,0429 |
| A\_51\_P490767 | NM\_008439 | Mus musculus ketohexokinase (Khk), mRNA | 0.71 | 0,0052 |
| A\_51\_P177992 | XM\_289712 | CG1/XAP80 PROTEIN homolog [Mus musculus] | 0.71 | 0,0236 |
| A\_51\_P400096 | NM\_011541 | Mus musculus transcription elongation factor A (SII) 1 (Tcea1), mRNA | 0.71 | 0,0419 |
| A\_51\_P110938 | NM\_172803 | Mus musculus RIKEN cDNA 6330411N01 gene (6330411N01Rik), mRNA | 0.71 | 0,0366 |
| A\_51\_P476706 | NM\_175526 | weakly similar to C-TYPE LECTIN-LIKE RECEPTOR-1 [Homo sapiens] | 0.71 | 0,0310 |
| A\_51\_P132491 | NM\_175008 | "hypothetical EF-hand/Guanine nucleotide exchange factor for Ras-like GTPases� N-terminal motif containing protein" | 0.71 | 0,0060 |
| A\_51\_P448757 | NM\_013698 | Mus musculus TXK tyrosine kinase (Txk), mRNA | 0.71 | 0,0266 |
| A\_51\_P213597 | NM\_178189 | HISTONE H2A homolog [Homo sapiens] | 0.71 | 0,0451 |
| A\_51\_P391096 | NAP027677-1 | GAMMA AMINOBUTYRIC ACID RECEPTOR SUBUNIT PRECURSOR GABA A RECEPTOR | 0.71 | 0,0230 |
| A\_51\_P163901 | NM\_146899 | Mus musculus olfactory receptor MOR233-6 (MOR233-6), mRNA | 0.71 | 0,0099 |
| A\_51\_P147527 | NM\_022030 | Mus musculus synaptic vesicle glycoprotein 2 a (Sv2a), mRNA | 0.71 | 0,0067 |
| A\_51\_P458242 | NM\_019880 | Mus musculus mitochondrial carrier homolog 1 (Mtch1-pending), mRNA | 0.71 | 0,0172 |
| A\_51\_P179894 | AK090370 | hypothetical RFX DNA-binding domain containing protein | 0.71 | 0,0066 |
| A\_51\_P218091 | NM\_010195 | Mus musculus G protein-coupled receptor 49 (Gpr49), mRNA | 0.71 | 0,0077 |
| A\_51\_P120295 | NM\_029562 | Mus musculus RIKEN cDNA 1300006E06 gene (1300006E06Rik), mRNA | 0.71 | 0,0108 |
| A\_51\_P131442 | NM\_013874 | Mus musculus neuronal d4 domain family member (Neud4), mRNA | 0.71 | 0,0403 |
| A\_51\_P320249 | AK122579 | musculus adult male colon cDNA, RIKEN full-length enriched library, clone:9030612M13:unclassifiable transcript, full insert sequence | 0.71 | 0,0110 |
| A\_51\_P234893 | NM\_021306 | Mus musculus endothelin converting enzyme-like 1 (Ecel1), mRNA | 0.71 | 0,0117 |
| A\_51\_P484537 | NM\_008017 | Mus musculus SMC2 structural maintenance of chromosomes 2-like 1 (yeast) (Smc2l1), mRNA | 0.71 | 0,0100 |
| A\_51\_P369683 | NM\_007842 | Mus musculus RNA helicase A (Ddx9) mRNA, complete cds | 0.71 | 0,0419 |
| A\_51\_P397454 | AK038921 | GAMMA TUBULIN RING COMPLEX PROTEIN homolog [Homo sapiens] | 0.71 | 0,0239 |
| A\_51\_P427312 | NM\_013855 | Mus musculus ATP-binding cassette protein (Abca3) mRNA, partial cds | 0.71 | 0,0465 |
| A\_51\_P267925 | NM\_172864 | similar to TESTIS DEVELOPMENT PROTEIN NYD-SP29 [Homo sapiens] | 0.71 | 0,0288 |
| A\_51\_P520262 | AK018056 | hypothetical protein | 0.71 | 0,0345 |
| A\_51\_P495876 | NM\_022328 | Mus musculus myeloid/lymphoid or mixed lineage-leukemia translocation to 4 homolog (Drosophila) (Mllt1), mRNA | 0.71 | 0,0264 |
| A\_51\_P332547 | AK017723 | Mus musculus RIKEN cDNA 5730493B19 gene (5730493B19Rik), mRNA | 0.71 | 0,0108 |
| A\_51\_P430259 | BC061017 | hypothetical protein | 0.71 | 0,0451 |
| A\_51\_P450394 | AV080718 | Mus musculus cDNA | 0.71 | 0,0027 |
| A\_51\_P358812 | AK081685 | DNA segment, Chr 5, Brigham & Womens Genetics 0676 expressed | 0.71 | 0,0031 |
| A\_51\_P409729 | NM\_021307 | Mus musculus zinc finger protein 112 (Zfp112), mRNA | 0.71 | 0,0368 |
| A\_51\_P411079 | AK045374 | enhancer of zeste homolog 1 (Drosophila) | 0.71 | 0,0146 |
| A\_51\_P146593 | NM\_146773 | Mus musculus olfactory receptor MOR204-6 (MOR204-6), mRNA | 0.71 | 0,0164 |
| A\_51\_P282663 | NM\_010421 | Mus musculus hexosaminidase A (Hexa), mRNA | 0.71 | 0,0028 |
| A\_51\_P439686 | NM\_172507 | Mus musculus RIKEN cDNA A930014C21 gene (A930014C21Rik), mRNA | 0.71 | 0,0139 |
| A\_51\_P352027 | TC1436661 | Mus musculus cDNA, 5 end | 0.71 | 0,0070 |
| A\_51\_P342786 | AK029786 | weakly similar to DNA MISMATCH REPAIR PROTEIN MLH3 (MUTL PROTEIN HOMOLOG 3) [Homo sapiens] | 0.71 | 0,0091 |
| A\_51\_P118712 | NM\_010047 | DiGeorge syndrome chromosome region 6 | 0.71 | 0,0491 |
| A\_51\_P106059 | NM\_009423 | Mus musculus Tnf receptor associated factor 4 (Traf4), mRNA | 0.71 | 0,0130 |
| A\_51\_P219109 | NM\_008353 | Mus musculus interleukin 12 receptor, beta 1 (Il12rb1), mRNA | 0.71 | 0,0060 |
| A\_51\_P233825 | NM\_009648 | Mus musculus A kinase (PRKA) anchor protein 1 (Akap1), mRNA | 0.71 | 0,0033 |
| A\_51\_P183041 | NM\_021418 | Mus musculus hypothetical protein, MNCb-4779 (AB041550), mRNA | 0.71 | 0,0428 |
| A\_51\_P189292 | NM\_138657 | Mus musculus SH2 domain containing SOCS box protein SOCS7 (SOCS7), mRNA | 0.71 | 0,0123 |
| A\_51\_P472671 | NM\_026219 | Mus musculus RIKEN cDNA 2210415M14 gene (2210415M14Rik), mRNA | 0.71 | 0,0044 |
| A\_51\_P318182 | XM\_159553 | hypothetical Leucine-rich region containing protein | 0.71 | 0,0487 |
| A\_51\_P121058 | NM\_011639 | Mus musculus thyroid hormone receptor interactor 6 (Trip6), mRNA | 0.71 | 0,0435 |
| A\_51\_P316616 | NM\_174992 | Mus musculus, Similar to hypothetical protein from clone 643, clone MGC:7903 IMAGE:3582955, mRNA, complete cds | 0.71 | 0,0258 |
| A\_51\_P459320 | NM\_019832 | Mus musculus 42 kD cGMP-dependent protein kinase anchoring protein (Gkap42-pending), mRNA | 0.71 | 0,0202 |
| A\_51\_P230324 | BC050801 | hypothetical EF-hand containing protein | 0.71 | 0,0445 |
| A\_51\_P488411 | NM\_022028 | Mus musculus WW domain-containing protein 3 (Wwp3-pending), mRNA | 0.71 | 0,0489 |
| A\_51\_P314941 | NM\_013862 | Mus musculus hematopoietic, heart, liver (Hhl-pending), mRNA | 0.71 | 0,0327 |
| A\_51\_P408310 | XM\_138431 | hypothetical Vitamin B12 dependent methionine synthase activation domain containing protein | 0.71 | 0,0111 |
| A\_51\_P424990 | AK045732 | hypothetical Kelch repeat containing protein | 0.71 | 0,0147 |
| A\_51\_P390310 | NM\_026248 | Mus musculus RIKEN cDNA 4930430A15 gene (4930430A15Rik), mRNA | 0.71 | 0,0224 |
| A\_51\_P294402 | NM\_153790 | Mus musculus scavenger receptor class F, member 2 (Scarf2), mRNA | 0.71 | 0,0015 |
| A\_51\_P162671 | NM\_008035 | Mus musculus folate receptor 2 (fetal) (Folr2), mRNA | 0.71 | 0,0028 |
| A\_51\_P481563 | AK035983 | RIKEN cDNA 4631403P03 gene | 0.71 | 0,0163 |
| A\_51\_P273750 | NM\_008145 | ENSMUSG00000015812 | 0.71 | 0,0379 |
| A\_51\_P290299 | AK028860 | unknown EST | 0.71 | 0,0126 |
| A\_51\_P409170 | NM\_008492 | Mus musculus lactate dehydrogenase 2, B chain (Ldh2), mRNA | 0.71 | 0,0451 |
| A\_51\_P254181 | NM\_029629 | unknown | 0.71 | 0,0099 |
| A\_51\_P380136 | AK018651 | syntrophin associated serine/threonine kinase | 0.71 | 0,0083 |
| A\_51\_P360655 | NM\_008766 | Mus musculus solute carrier family 22 (organic anion transporter), member 6 (Slc22a6), mRNA | 0.71 | 0,0031 |
| A\_51\_P363194 | AK019693 | similar to EIF4GII [Homo sapiens] | 0.71 | 0,0109 |
| A\_51\_P471830 | NM\_153075 | Mus musculus putative ion channel protein CATSPER2 mRNA, complete cds | 0.71 | 0,0086 |
| A\_51\_P472469 | NM\_175111 | PROTEIN ASSOCIATING WITH SMALL STRESS PROTEIN PASS1 homolog [Rattus norvegicus] | 0.71 | 0,0175 |
| A\_51\_P482925 | NM\_175363 | hypothetical Serine-rich region containing protein | 0.71 | 0,0043 |
| A\_51\_P514085 | NM\_013606 | Mus musculus myxovirus (influenza virus) resistance 2 (Mx2), mRNA | 0.71 | 0,0394 |
| A\_51\_P131261 | A\_51\_P131261 | Mus musculus cDNA, 3 end | 0.71 | 0,0245 |
| A\_51\_P445882 | AK018014 | musculus adult male thymus cDNA, RIKEN full-length enriched library, clone:5830455I16:T-cell receptor beta, joining region, full insert sequence | 0.71 | 0,0077 |
| A\_51\_P401673 | NM\_053072 | Mus musculus ethanol decreased 4 (Etohd4), mRNA | 0.71 | 0,0167 |
| A\_51\_P140415 | NM\_008265 | Murine mRNA for Hox-1.4 protein | 0.71 | 0,0018 |
| A\_51\_P260265 | NM\_010469 | Mus musculus homeo box D4 (Hoxd4), mRNA | 0.71 | 0,0374 |
| A\_51\_P231549 | NM\_153760 | Mus musculus MHC I - like leukocyte 2 (Mill2), mRNA | 0.71 | 0,0135 |
| A\_51\_P118742 | NM\_021354 | Mus musculus developmentally regulated GTP binding protein 2 (Drg2), mRNA | 0.71 | 0,0396 |
| A\_51\_P162886 | AK186053 | Mus musculus cDNA, 5 end | 0.71 | 0,0132 |
| A\_51\_P134333 | BC006665 | Mus musculus similar to Mitogen-activated protein kinase kinase kinase 7 (Transforming growth factor-beta-activated kinase 1) (TGF-beta-activated kinase 1) (LOC216178), mRNA | 0.71 | 0,0231 |
| A\_51\_P178251 | NM\_028833 | hypothetical IQ calmodulin-binding motif containing protein | 0.71 | 0,0181 |
| A\_51\_P292160 | NM\_177772 | hypothetical Lipid-binding serum glycoprotein containing protein | 0.70 | 0,0249 |
| A\_51\_P119266 | NM\_011768 | Mus musculus zinc finger protein X-linked (Zfx), mRNA | 0.70 | 0,0155 |
| A\_51\_P379478 | NM\_172484 | hypothetical protein | 0.70 | 0,0174 |
| A\_51\_P301930 | BC030317 | 37 kDa leucine-rich repeat (LRR) protein | 0.70 | 0,0493 |
| A\_51\_P348022 | NM\_026623 | Mus musculus cleavage and polyadenylation specific factor 5, 25 kD subunit (Cpsf5), mRNA | 0.70 | 0,0022 |
| A\_51\_P441970 | NM\_175162 | hypothetical protein | 0.70 | 0,0064 |
| A\_51\_P215489 | NM\_153062 | GLYCEROL-3-PHOSPHATE TRANSPORTER (G-3-P TRANSPORTER) (G-3-P PERMEASE) homolog [Homo sapiens] | 0.70 | 0,0103 |
| A\_51\_P351975 | NM\_010449 | Mus musculus homeo box A1 (Hoxa1), mRNA | 0.70 | 0,0234 |
| A\_51\_P337350 | NM\_012016 | Mus musculus endoplasmic reticulum (ER) to nucleus signalling 2 (Ern2), mRNA | 0.70 | 0,0082 |
| A\_51\_P247928 | NM\_053155 | Mus musculus calmin (Clmn), mRNA | 0.70 | 0,0282 |
| A\_51\_P484510 | NM\_011848 | Mus musculus NIMA (never in mitosis gene a)-related expressed kinase 3 (Nek3), mRNA | 0.70 | 0,0233 |
| A\_51\_P137640 | AK011315 | RIKEN cDNA 2610005D18 gene | 0.70 | 0,0146 |
| A\_51\_P352141 | NM\_001013380 | DYNEIN LIGHT INTERMEDIATE CHAIN 2, CYTOSOLIC (LIC53/55) (LIC-2) homolog [Rattus norvegicus] | 0.70 | 0,0066 |
| A\_51\_P446678 | NM\_018736 | Mus musculus meiotic recombination 11 homolog A (S. cerevisiae) (Mre11a), mRNA | 0.70 | 0,0231 |
| A\_51\_P355301 | NM\_007818 | Mus musculus cytochrome P450, family 3, subfamily a, polypeptide 11 (Cyp3a11), mRNA | 0.70 | 0,0271 |
| A\_51\_P510782 | NM\_130859 | Mus musculus caspase recruitment domain family, member 10 (Card10), mRNA | 0.70 | 0,0381 |
| A\_51\_P323291 | NM\_011588 | Mus musculus tripartite motif protein 28 (Trim28), mRNA | 0.70 | 0,0263 |
| A\_51\_P166948 | BC027735 | Mus musculus, Similar to KIAA1623 protein, clone IMAGE:3711771, mRNA | 0.70 | 0,0020 |
| A\_51\_P339331 | AK018683 | HOMEOBOX PROTEIN NKX 6 | 0.70 | 0,0104 |
| A\_51\_P287656 | NM\_001011536 | Mus musculus olfactory receptor MOR14-7P (MOR14-7P) pseudogene | 0.70 | 0,0499 |
| A\_51\_P364014 | NM\_183144 | NK6 transcription factor related, locus 2 (Drosophila) | 0.70 | 0,0459 |
| A\_51\_P195374 | NM\_025833 | INSULIN RECEPTOR TYROSINE KINASE SUBSTRATE homolog [Homo sapiens] | 0.70 | 0,0113 |
| A\_51\_P391996 | BC011329 | Mus musculus, RIKEN cDNA 0610042A05 gene, clone MGC:18955 IMAGE:3984654, mRNA, complete cds | 0.70 | 0,0188 |
| A\_51\_P288386 | NM\_008837 | Mus musculus protein kinase C, alpha binding protein (Prkcabp), mRNA | 0.70 | 0,0282 |
| A\_51\_P413075 | NM\_175524 | hypothetical Rhodopsin-like GPCR superfamily containing protein | 0.70 | 0,0167 |
| A\_51\_P450547 | NM\_011060 | Mus musculus peptidyl arginine deiminase, type III (Pdi3), mRNA | 0.70 | 0,0212 |
| A\_51\_P334404 | A\_51\_P334404 | Mus musculus pyridoxal (pyridoxine, vitamin B6) kinase, pseudogene (Pdxk-ps) on chromosome 17 | 0.70 | 0,0020 |
| A\_51\_P207622 | NM\_021355 | Mus musculus fibromodulin (Fmod), mRNA | 0.70 | 0,0263 |
| A\_51\_P184162 | NM\_178755 | hypothetical Zn-dependent exopeptidases structure containing protein | 0.70 | 0,0125 |
| A\_51\_P167505 | AK079340 | inferred: heat shock factor 2 {Mus musculus} | 0.70 | 0,0110 |
| A\_51\_P281670 | NM\_172660 | endonuclease G | 0.70 | 0,0051 |
| A\_51\_P166339 | NM\_009635 | Mus musculus advillin (Advil-pending), mRNA | 0.70 | 0,0325 |
| A\_51\_P364609 | NM\_019699 | Mus musculus fatty acid desaturase 2 (Fads2), mRNA | 0.70 | 0,0110 |
| A\_51\_P461844 | NM\_008775 | Mus musculus platelet-activating factor acetylhydrolase, isoform 1b, alpha2 subunit (Pafah1b2), mRNA | 0.70 | 0,0219 |
| A\_51\_P421763 | XM\_485619 | hypothetical protein | 0.70 | 0,0067 |
| A\_51\_P285462 | NM\_146276 | Mus musculus olfactory receptor MOR280-1 (MOR280-1), mRNA | 0.70 | 0,0046 |
| A\_51\_P278929 | AK014905 | hypothetical Tubulin-tyrosine ligase containing protein | 0.70 | 0,0436 |
| A\_51\_P238073 | BU962389 | Mus musculus cDNA, 5 end | 0.70 | 0,0137 |
| A\_51\_P483934 | NM\_023505 | Mus musculus glutaredoxin 2 (thioltransferase) (Glrx2), mRNA | 0.70 | 0,0104 |
| A\_51\_P487950 | XM\_483962 | Mus musculus brush border myosin-I (BBM-I) mRNA, partial cds | 0.70 | 0,0091 |
| A\_51\_P409101 | AK006216 | hypothetical Cadherin structure containing protein | 0.70 | 0,0012 |
| A\_51\_P295286 | AK006906 | 1700066M21RIK PROTEIN homolog [Mus musculus] | 0.70 | 0,0232 |
| A\_51\_P343218 | NM\_009792 | Mus musculus calcium/calmodulin-dependent protein kinase II alpha (Camk2a), mRNA | 0.70 | 0,0348 |
| A\_51\_P155085 | NM\_172477 | weakly similar to tumor cell suppression protein HTS1 (fragment) [Homo sapiens] | 0.70 | 0,0364 |
| A\_51\_P206734 | NM\_133657 | Mus musculus cytochrome P450, 2a12 (Cyp2a12), mRNA | 0.70 | 0,0186 |
| A\_51\_P390857 | AK054376 | unclassifiable | 0.70 | 0,0138 |
| A\_51\_P479052 | NM\_033074 | Mus musculus DNA segment, Chr 15, Wayne State University 59, expressed (D15Wsu59e), mRNA | 0.70 | 0,0305 |
| A\_51\_P260167 | NM\_008158 | Mus musculus G protein-coupled receptor 27 (Gpr27), mRNA | 0.70 | 0,0058 |
| A\_51\_P228208 | NM\_172711 | CDNA FLJ13220 FIS, CLONE NT2RP4002047, MODERATELY SIMILAR TO GTP-BINDING PROTEIN LEPA homolog [Homo sapiens] | 0.70 | 0,0147 |
| A\_51\_P417096 | NM\_011728 | Mus musculus xeroderma pigmentosum, complementation group A (Xpa), mRNA | 0.70 | 0,0102 |
| A\_51\_P134844 | NM\_024225 | Mus musculus sorting nexin 5 (Snx5), mRNA | 0.70 | 0,0411 |
| A\_51\_P184484 | NM\_008607 | Mus musculus matrix metalloproteinase 13 (Mmp13), mRNA | 0.70 | 0,0153 |
| A\_51\_P148374 | AK018327 | SUBSTANCE K RECEPTOR SKR NEUROKININ A RECEPTOR NK 2 RECEPTOR NK | 0.70 | 0,0100 |
| A\_51\_P150145 | AK077046 | unknown EST | 0.70 | 0,0317 |
| A\_51\_P464490 | A\_51\_P464490 | Mus musculus cDNA, 5 end | 0.70 | 0,0137 |
| A\_51\_P269002 | NM\_080793 | Mus musculus SET domain-containing protein 7 (Set7), mRNA | 0.70 | 0,0124 |
| A\_51\_P482289 | NM\_023525 | "CAD PROTEIN [INCLUDES: GLUTAMINE DEPENDENT CARBAMOYL PHOSPHATE SYNTHASE EC 6.3.5.5� ASPARTATE CARBAMOYLTRANSFERASE EC 2.1.3.- 2� DIHYDROOROTASE EC 3.5.-.- 2 3" | 0.70 | 0,0174 |
| A\_51\_P231697 | XM\_112192 | INTEGRIN ALPHA-10 PRECURSOR homolog [Homo sapiens] | 0.70 | 0,0370 |
| A\_51\_P205209 | NM\_018729 | Mus musculus non MHC restricted killing associated (Nmrk), mRNA | 0.70 | 0,0065 |
| A\_51\_P482473 | NM\_009092 | ribosomal protein S17 | 0.70 | 0,0393 |
| A\_51\_P203277 | NM\_134230 | Mus musculus vomeronasal 1 receptor, E11 (V1re11), mRNA | 0.70 | 0,0317 |
| A\_51\_P347764 | NM\_021315 | Mus musculus unknown mRNA | 0.69 | 0,0035 |
| A\_51\_P328769 | NM\_182999 | KAIA2769 PROTEIN (FRAGMENT) homolog [Homo sapiens] | 0.69 | 0,0258 |
| A\_51\_P114126 | AK012034 | hypothetical protein | 0.69 | 0,0143 |
| A\_51\_P140607 | NM\_138757 | Mus musculus hypothetical protein MGC28965 (MGC28965), mRNA | 0.69 | 0,0093 |
| A\_51\_P495459 | NM\_153394 | Mus musculus GABAB-related G-protein coupled receptor (GABABL), mRNA | 0.69 | 0,0245 |
| A\_51\_P447495 | NM\_027580 | hypothetical protein | 0.69 | 0,0321 |
| A\_51\_P245208 | NM\_010317 | Mus musculus guanine nucleotide binding protein (G protein), gamma 4 subunit (Gng4), mRNA | 0.69 | 0,0157 |
| A\_51\_P267663 | NM\_030251 | Mus musculus ankyrin repeat and BTB (POZ) domain containing 1 (Abtb1), mRNA | 0.69 | 0,0252 |
| A\_51\_P476859 | NAP057262-1 | Mus musculus olfactory receptor MOR125-3P (MOR125-3P) pseudogene | 0.69 | 0,0397 |
| A\_51\_P219896 | XM\_620244 | BA436C9.2 (PUTATIVE NOVEL PROTEIN SIMILAR TO PART OF HSP70/HSP90 ORGANIZING PROTEIN AND TRANSFORMATION SENSITIVE PROTEIN) (FRAGMENT) homolog [Homo sapiens] | 0.69 | 0,0346 |
| A\_51\_P376789 | NM\_145387 | hypothetical Six-hairpin glycosyltransferases structure containing protein | 0.69 | 0,0498 |
| A\_51\_P175303 | NM\_019725 | Mus musculus transducin-like enhancer of split 2, homolog of Drosophila E(spl) (Tle2), mRNA | 0.69 | 0,0281 |
| A\_51\_P500090 | NM\_014194 | Mus musculus killer cell lectin-like receptor, subfamily A, member 7 (Klra7), mRNA | 0.69 | 0,0453 |
| A\_51\_P486289 | AK122224 | hypothetical Serine-rich region containing protein | 0.69 | 0,0131 |
| A\_51\_P125842 | NM\_145402 | similar to HYPOTHETICAL 27.8 KDA PROTEIN [Homo sapiens] | 0.69 | 0,0370 |
| A\_51\_P297470 | NM\_025511 | hypothetical protein | 0.69 | 0,0172 |
| A\_51\_P242024 | NM\_018753 | Mus musculus tyrosine 3-monooxygenase/tryptophan 5-monooxygenase activation protein, beta polypeptide (Ywhab), mRNA | 0.69 | 0,0027 |
| A\_51\_P516615 | NM\_026684 | NADH dehydrogenase (ubiquinone) (EC 1.6.5.3) chain CI-PDSW homolog [Bos primigenius taurus] | 0.69 | 0,0363 |
| A\_51\_P460302 | NM\_130863 | Mus musculus, Similar to adrenergic, beta, receptor kinase 1, clone IMAGE:3586085, mRNA, partial cds | 0.69 | 0,0100 |
| A\_51\_P279888 | NM\_001029933 | Mus musculus zinc finger protein ZFP110 mRNA, partial cds | 0.69 | 0,0030 |
| A\_51\_P258381 | BC010717 | Mus musculus, clone IMAGE:4009470, mRNA | 0.69 | 0,0021 |
| A\_51\_P372302 | XM\_355182 | Mus musculus, clone IMAGE:2811941, mRNA | 0.69 | 0,0041 |
| A\_51\_P453330 | NM\_173433 | weakly similar to HYPOTHETICAL 37.5 KDA PROTEIN [Homo sapiens] | 0.69 | 0,0206 |
| A\_51\_P314339 | NM\_023215 | Mus musculus RIKEN cDNA 2500003M10 gene (2500003M10Rik), mRNA | 0.69 | 0,0195 |
| A\_51\_P441455 | AK032831 | hypothetical PPR repeats containing protein | 0.69 | 0,0204 |
| A\_51\_P409570 | NM\_146156 | Mus musculus, similar to Cell division control protein 2 homolog (P34 protein kinase), clone MGC:36635 IMAGE:5358000, mRNA, complete cds | 0.69 | 0,0376 |
| A\_51\_P484010 | NM\_007941 | Mus musculus epimorphin (Epim), mRNA | 0.69 | 0,0053 |
| A\_51\_P119235 | XM\_622090 | Mus musculus cardiac morphogenesis (Xin), mRNA | 0.69 | 0,0276 |
| A\_51\_P146533 | AK039142 | similar to PRESENILINS ASSOCIATED RHOMBOID-LIKE PROTEIN [Homo sapiens] | 0.69 | 0,0322 |
| A\_51\_P304527 | NM\_029738 | Mus musculus, clone MGC:28015 IMAGE:3603454, mRNA, complete cds | 0.69 | 0,0358 |
| A\_51\_P180773 | NM\_146846 | Mus musculus olfactory receptor MOR188-5 (MOR188-5), mRNA | 0.69 | 0,0071 |
| A\_51\_P216965 | NM\_010221 | Mus musculus FK506 binding protein 10 (65 kDa) (Fkbp10), mRNA | 0.69 | 0,0112 |
| A\_51\_P511646 | NM\_029561 | NEDD4 WW DOMAIN-BINDING PROTEIN 5A homolog [Mus musculus] | 0.69 | 0,0430 |
| A\_51\_P379373 | NM\_145381 | Similar to: Mus musculus CGI-83 protein (Cgi-83-pending), mRNA | 0.69 | 0,0031 |
| A\_51\_P197763 | NM\_199007 | hypothetical protein | 0.69 | 0,0101 |
| A\_51\_P497594 | AK037109 | hypothetical protein | 0.69 | 0,0047 |
| A\_51\_P387632 | NM\_022885 | Mus musculus zinc transporter like 1 (Zntl1), mRNA | 0.69 | 0,0377 |
| A\_51\_P273863 | NM\_172919 | similar to MSZF23-1 (FRAGMENT) [Mus musculus] | 0.69 | 0,0037 |
| A\_51\_P410040 | AK045603 | unknown EST | 0.69 | 0,0277 |
| A\_51\_P412595 | NM\_018796 | Mus musculus eukaryotic translation elongation factor 1 beta 2 (Eef1b2), mRNA | 0.69 | 0,0274 |
| A\_51\_P488619 | NM\_030131 | PROTEIN HSPC163 homolog [Homo sapiens] | 0.69 | 0,0322 |
| A\_51\_P253527 | NM\_026097 | Mus musculus RIKEN cDNA 1700051E09 gene (1700051E09Rik), mRNA | 0.69 | 0,0288 |
| A\_51\_P507982 | NM\_019635 | Mus musculus serine/threonine kinase 3 (Ste20, yeast homolog) (Stk3), mRNA | 0.69 | 0,0375 |
| A\_51\_P472730 | BC048726 | hypothetical KRAB box containing protein | 0.69 | 0,0120 |
| A\_51\_P496668 | NM\_028938 | hypothetical IQ calmodulin-binding motif/Protein splicing (intein)/Leucine-rich repeat containing protein | 0.69 | 0,0038 |
| A\_51\_P325173 | AK077713 | Mus musculus tropomyosin 1, alpha (Tpm1), mRNA | 0.69 | 0,0190 |
| A\_51\_P162593 | NM\_025513 | APOPTOSIS-RELATED PROTEIN PNAS-3 (FRAGMENT) homolog [Homo sapiens] | 0.69 | 0,0264 |
| A\_51\_P427425 | NM\_144793 | hypothetical Mitochondrial energy transfer proteins (carrier protein) containing protein | 0.69 | 0,0068 |
| A\_51\_P110301 | NM\_009778 | Mouse complement component C3 mRNA, alpha and beta subunits, complete cds | 0.69 | 0,0232 |
| A\_51\_P151038 | BC087943 | MITOCHONDRIAL PROCESSING PEPTIDASE BETA SUBUNIT, MITOCHONDRIAL PRECURSOR (EC 3.4.24.64) (BETA-MPP) (P-52) | 0.69 | 0,0173 |
| A\_51\_P446315 | NM\_025950 | Mus musculus cell division cycle 37 homolog (S. cerevisiae)-like (Cdc37l), mRNA | 0.69 | 0,0010 |
| A\_51\_P468418 | NM\_145618 | Mus musculus NMDA receptor-regulated gene 2 (Narg2), mRNA | 0.69 | 0,0252 |
| A\_51\_P169374 | NM\_027172 | hypothetical protein | 0.69 | 0,0210 |
| A\_51\_P188271 | NM\_054042 | Mus musculus tumor endothelial marker 1 precursor (Tem1-pending), mRNA | 0.69 | 0,0040 |
| A\_51\_P344636 | NM\_146924 | Mus musculus olfactory bulb punch clone MORPC9 olfactory receptor mRNA, partial cds | 0.69 | 0,0066 |
| A\_51\_P365139 | AK014911 | hypothetical leucine zipper-like motif containing protein | 0.69 | 0,0470 |
| A\_51\_P257457 | NM\_013589 | Mus musculus latent transforming growth factor beta binding protein 2 (Ltbp2), mRNA | 0.69 | 0,0417 |
| A\_51\_P357195 | XM\_132740 | Mus musculus cDNA clone K0420E06 3, mRNA sequence | 0.69 | 0,0036 |
| A\_51\_P253195 | AK035031 | hypothetical 2Fe-2S Ferredoxin/Zinc finger, C2H2 type containing protein | 0.69 | 0,0136 |
| A\_51\_P339417 | NM\_013905 | Mus musculus hairy/enhancer-of-split related with YRPW motif-like (Heyl), mRNA | 0.69 | 0,0085 |
| A\_51\_P355427 | NM\_080639 | Mus musculus tissue inhibitor of metalloproteinase 4 (Timp4), mRNA | 0.69 | 0,0012 |
| A\_51\_P295022 | NM\_010890 | Mus musculus neural precursor cell expressed, developmentally down-regulated gene 4a (Nedd4a), mRNA | 0.69 | 0,0174 |
| A\_51\_P294550 | NM\_019829 | Mus musculus syntaxin 5A (Stx5a), mRNA | 0.69 | 0,0326 |
| A\_51\_P505738 | NM\_145635 | Mus musculus SMAF1 mRNA, complete cds | 0.69 | 0,0183 |
| A\_51\_P386182 | NM\_015823 | Mus musculus activin receptor interacting protein 1 (Acvrip1-pending), mRNA | 0.69 | 0,0301 |
| A\_51\_P104710 | NM\_173428 | Mus musculus SCO-spondin (LOC243369), mRNA | 0.69 | 0,0097 |
| A\_51\_P214466 | NM\_173744 | hypothetical protein | 0.69 | 0,0415 |
| A\_51\_P422465 | NM\_134187 | Mus musculus vomeronasal 1 receptor, C32 (V1rc32), mRNA | 0.69 | 0,0215 |
| A\_51\_P393958 | NM\_025995 | f-box only protein 31 | 0.69 | 0,0229 |
| A\_51\_P260428 | NM\_008976 | Mus musculus protein tyrosine phosphatase, non-receptor type 14 (Ptpn14), mRNA | 0.69 | 0,0364 |
| A\_51\_P148512 | AK079640 | unknown EST | 0.69 | 0,0239 |
| A\_51\_P272184 | NM\_026201 | weakly similar to CDNA FLJ10590 FIS, CLONE NT2RP2004392, WEAKLY SIMILAR TO MNN4 PROTEIN [Homo sapiens] | 0.69 | 0,0317 |
| A\_51\_P280297 | NM\_019934 | Mus musculus fucosyltransferase 10 (Fut10-pending), mRNA | 0.69 | 0,0285 |
| A\_51\_P386870 | NM\_011472 | Mus musculus small proline-rich protein 2F (Sprr2f), mRNA | 0.69 | 0,0324 |
| A\_51\_P171125 | NM\_026251 | hypothetical protein | 0.69 | 0,0320 |
| A\_51\_P409260 | NM\_008539 | Mus musculus MAD homolog 1 (Drosophila) (Madh1), mRNA | 0.69 | 0,0059 |
| A\_51\_P183732 | NM\_016980 | ribosomal protein L5 | 0.69 | 0,0333 |
| A\_51\_P226882 | NM\_021309 | Mus musculus SH2 domain protein 2A (Sh2d2a), mRNA | 0.69 | 0,0039 |
| A\_51\_P161503 | XM\_622761 | similar to VACUOLAR ATP SYNTHASE SUBUNIT F (EC 3.6.3.14) (V-ATPASE F SUBUNIT) (VACUOLAR PROTON PUMP F SUBUNIT) (V-ATPASE 14 KDA SUBUNIT) [Rattus norvegicus] | 0.68 | 0,0305 |
| A\_51\_P215780 | NM\_029572 | Mus musculus endoplasmic reticulum resident protein 44kDa (Erp44-pending), mRNA | 0.68 | 0,0060 |
| A\_51\_P338594 | NM\_025387 | Mus musculus RIKEN cDNA 1110021D01 gene (1110021D01Rik), mRNA | 0.68 | 0,0016 |
| A\_51\_P252127 | NAP057276-1 | Mus musculus olfactory receptor MOR111-8P (MOR111-8P) pseudogene | 0.68 | 0,0490 |
| A\_51\_P255899 | NM\_011578 | Mus musculus betaglycan mRNA, complete cds | 0.68 | 0,0355 |
| A\_51\_P332957 | NM\_033398 | Mus musculus phosphatidylserine receptor (Ptdsr), mRNA | 0.68 | 0,0437 |
| A\_51\_P205129 | AB030188 | Mus musculus hypothetical protein, clone 1-53 (AB030188), mRNA | 0.68 | 0,0052 |
| A\_51\_P178377 | AK019700 | Mus musculus mitochondrial solute carrier protein (Mscp-pending), mRNA | 0.68 | 0,0110 |
| A\_51\_P140207 | NM\_025601 | Mus musculus RIKEN cDNA 1700029H14 gene (1700029H14Rik), mRNA | 0.68 | 0,0265 |
| A\_51\_P453657 | NM\_018802 | Mus musculus synaptotagmin 8 (Syt8), mRNA | 0.68 | 0,0458 |
| A\_51\_P209258 | AK080354 | unclassifiable | 0.68 | 0,0092 |
| A\_51\_P323583 | NM\_134111 | Mus musculus expressed sequence AW048865 (AW048865), mRNA | 0.68 | 0,0014 |
| A\_51\_P317695 | NM\_019510 | Mus musculus transient receptor protein 3 (Trrp3), mRNA | 0.68 | 0,0295 |
| A\_51\_P497317 | NM\_146008 | Mus musculus, Similar to hypothetical protein FLJ11336, clone IMAGE:3600297, mRNA | 0.68 | 0,0050 |
| A\_51\_P441494 | NM\_021440 | Mus musculus testis specific protein, Ddc8 (Ddc8-pending), mRNA | 0.68 | 0,0056 |
| A\_51\_P473086 | NM\_009325 | Mus musculus thromboxane A2 receptor (Tbxa2r), mRNA | 0.68 | 0,0416 |
| A\_51\_P461444 | L26316 | Mus musculus dihydrofolate reductase (Dhfr), mRNA | 0.68 | 0,0253 |
| A\_51\_P128229 | AK013201 | inferred: HN1 like {Homo sapiens} | 0.68 | 0,0126 |
| A\_51\_P317443 | NM\_145822 | "Mus musculus, Similar to CD3-epsilon-associated protein� antisense to ERCC-1, clone IMAGE:4485007, mRNA" | 0.68 | 0,0407 |
| A\_51\_P519002 | BC028549 | Mus musculus, clone IMAGE:1380624, mRNA | 0.68 | 0,0146 |
| A\_51\_P150530 | NM\_053157 | Mus musculus beta-amyloid binding protein (Bbp) mRNA, complete cds | 0.68 | 0,0458 |
| A\_51\_P292846 | NM\_023348 | Mus musculus RIKEN cDNA 1300018G05 gene (1300018G05Rik), mRNA | 0.68 | 0,0141 |
| A\_51\_P339822 | NM\_138630 | Mus musculus Rho GTPase activating protein 4 (Arhgap4), mRNA | 0.68 | 0,0221 |
| A\_51\_P469522 | NM\_011828 | Mus musculus heparan sulfate 2-O-sulfotransferase 1 (Hs2st1), mRNA | 0.68 | 0,0493 |
| A\_51\_P214663 | NM\_016785 | Mus musculus thiopurine methyltransferase (Tpmt), mRNA | 0.68 | 0,0182 |
| A\_51\_P459741 | NM\_026081 | PER1 INTERACTING PROTEIN OF THE SUPRACHIAMATIC NUCLEUS homolog [Rattus norvegicus] | 0.68 | 0,0257 |
| A\_51\_P321136 | NM\_146222 | Mus musculus, Similar to hypothetical protein FLJ23342, clone MGC:37388 IMAGE:4977181, mRNA, complete cds | 0.68 | 0,0017 |
| A\_51\_P146505 | NM\_029570 | Mus musculus, clone IMAGE:5363357, mRNA, partial cds | 0.68 | 0,0312 |
| A\_51\_P462149 | AF317223 | Mus musculus tropomyosin 3, gamma (Tpm3), mRNA | 0.68 | 0,0183 |
| A\_51\_P389751 | NM\_009046 | Mus musculus avian reticuloendotheliosis viral (v-rel) oncogene related B (Relb), mRNA | 0.68 | 0,0213 |
| A\_51\_P412297 | NM\_023625 | Mus musculus RIKEN cDNA 1300012G16 gene (1300012G16Rik), mRNA | 0.68 | 0,0285 |
| A\_51\_P294346 | AK035117 | hypothetical Myb DNA binding domain containing protein | 0.68 | 0,0226 |
| A\_51\_P495311 | NM\_130905 | Mus musculus Cd209e antigen (Cd209e-pending), mRNA | 0.68 | 0,0028 |
| A\_51\_P149189 | NM\_028459 | Mus musculus mRNA for N-WASP protein | 0.68 | 0,0083 |
| A\_51\_P259694 | AK009778 | X-ray repair complementing defective repair in Chinese hamster cells 1 | 0.68 | 0,0040 |
| A\_51\_P135423 | NM\_009798 | Mus musculus capping protein beta 1 (Cappb1), mRNA | 0.68 | 0,0190 |
| A\_51\_P170647 | S79463 | M-Sema F=a factor in neural network development [mice, neonatal brain, mRNA, 3503 nt] | 0.68 | 0,0214 |
| A\_51\_P107938 | NM\_009461 | Mus musculus ubiquitin protein ligase E3 component n-recognin 1 (Ubr1), mRNA | 0.68 | 0,0098 |
| A\_51\_P453909 | NM\_007817 | Mus musculus cytochrome P450, 2f2 (Cyp2f2), mRNA | 0.68 | 0,0152 |
| A\_51\_P185902 | NM\_025659 | similar to NESH (NESH PROTEIN) [Homo sapiens] | 0.68 | 0,0137 |
| A\_51\_P320281 | NM\_010574 | Mus musculus iroquois-class homeobox protein Irx6 (Irx6) mRNA, partial cds | 0.68 | 0,0148 |
| A\_51\_P507344 | NM\_001003915 | SODIUM/IODIDE COTRANSPORTER NA + /I COTRANSPORTER SODIUM IODIDE SYMPORTER NA+/I | 0.68 | 0,0411 |
| A\_51\_P233474 | NM\_009480 | Mus musculus upstream transcription factor 1 (Usf1), mRNA | 0.68 | 0,0011 |
| A\_51\_P133078 | NM\_008942 | Mus musculus puromycin-sensitive aminopeptidase (Psa), mRNA | 0.68 | 0,0117 |
| A\_51\_P477641 | NM\_008883 | Mus musculus plexin 3 (Plxn3), mRNA | 0.68 | 0,0147 |
| A\_51\_P339932 | XM\_130232 | Mus musculus nebulin mRNA, partial cds | 0.68 | 0,0030 |
| A\_51\_P481742 | XM\_139909 | SOMATOSTATIN RECEPTOR TYPE 5 (SS5R) | 0.68 | 0,0123 |
| A\_51\_P452280 | NM\_010839 | Mus musculus mature T-cell proliferation 1 (Mtcp1), mRNA | 0.68 | 0,0442 |
| A\_51\_P377641 | NM\_028434 | hypothetical protein | 0.68 | 0,0143 |
| A\_51\_P230298 | NM\_008232 | Mus musculus hepatoma-derived growth factor, related protein 1 (Hdgfrp1), mRNA | 0.68 | 0,0273 |
| A\_51\_P301215 | NM\_023197 | Mus musculus RIKEN cDNA 2310008H09 gene (2310008H09Rik), mRNA | 0.68 | 0,0279 |
| A\_51\_P282958 | AK031036 | BA307L3.1 (SIMILAR TO NUCLEAR PROTEIN NP95) (FRAGMENT) homolog [Homo sapiens] | 0.68 | 0,0267 |
| A\_51\_P442890 | AF289178 | Mus musculus anti-DNA monoclonal autoantibody G4-20 heavy chain variable region mRNA, partial cds | 0.68 | 0,0028 |
| A\_51\_P221831 | NM\_130875 | Mus musculus keratin associated protein 16-7 (Krtap16-7), mRNA | 0.68 | 0,0079 |
| A\_51\_P468658 | NM\_153543 | Mus musculus RIKEN cDNA D330038I09 gene (D330038I09Rik), mRNA | 0.68 | 0,0257 |
| A\_51\_P206783 | NM\_145619 | similar to POLY [ADP-RIBOSE] POLYMERASE-3 (EC 2.4.2.30) (PARP-3) (NAD(+) ADP- RIBOSYLTRANSFERASE-3) (POLY[ADP-RIBOSE] SYNTHETASE-3) (PADPRT-3) (HPARP-3) [Homo sapiens] | 0.68 | 0,0362 |
| A\_51\_P315344 | NM\_172486 | similar to DNA-BINDING PROTEIN (FRAGMENT) [Homo sapiens] | 0.68 | 0,0416 |
| A\_51\_P509264 | NM\_010455 | Mus musculus homeo box A7 (Hoxa7), mRNA | 0.68 | 0,0020 |
| A\_51\_P136870 | AK030022 | hypothetical Ankyrin repeat profile/Serine-rich region/Ankyrin-repeat/Ankyrin repeat region circular profile/Yeast DNA-binding domain containing protein | 0.68 | 0,0258 |
| A\_51\_P487010 | NM\_008899 | Mus musculus POU domain, class 3, transcription factor 2 (Pou3f2), mRNA | 0.68 | 0,0054 |
| A\_51\_P139437 | NM\_080464 | Mus musculus protein phosphatase 1, regulatory (inhibitor) subunit 3A (Ppp1r3a), mRNA | 0.68 | 0,0388 |
| A\_51\_P366542 | NM\_011925 | Mus musculus CD97 antigen (Cd97), mRNA | 0.68 | 0,0038 |
| A\_51\_P374651 | AK047427 | CHROMATIN | 0.68 | 0,0417 |
| A\_51\_P439311 | AK129415 | unknown EST | 0.68 | 0,0039 |
| A\_51\_P134158 | NM\_011604 | Mus musculus toll-like receptor 6 (Tlr6), mRNA | 0.68 | 0,0097 |
| A\_51\_P291062 | NM\_028266 | COLLAGEN ALPHA 1(XVI) CHAIN PRECURSOR homolog [Homo sapiens] | 0.68 | 0,0224 |
| A\_51\_P319386 | NM\_010862 | Mus musculus myosin XV (Myo15), mRNA | 0.68 | 0,0150 |
| A\_51\_P319490 | NM\_144866 | EUKARYOTIC PEPTIDE CHAIN RELEASE FACTOR SUBUNIT 1 (ERF1) (EUKARYOTIC RELEASE FACTOR 1) (TB3-1) (C11 PROTEIN) homolog [Homo sapiens] | 0.68 | 0,0022 |
| A\_51\_P100394 | NAP057315-1 | Mus musculus c222389 (c222389) pseudogene | 0.68 | 0,0317 |
| A\_51\_P165237 | NM\_029068 | SORTING NEXIN 16 homolog [Rattus norvegicus] | 0.68 | 0,0329 |
| A\_51\_P386612 | NM\_027153 | PIRIN [Mus musculus] | 0.68 | 0,0434 |
| A\_51\_P421294 | NM\_008132 | Mus musculus glutamine repeat protein 1 (Glrp1), mRNA | 0.68 | 0,0165 |
| A\_51\_P271897 | ENSMUST00000020074 | unknown | 0.68 | 0,0345 |
| A\_51\_P511029 | NM\_026720 | weakly similar to KE03 PROTEIN (FRAGMENT) [Homo sapiens] | 0.68 | 0,0035 |
| A\_51\_P284918 | BC028776 | Mus musculus, similar to teashirt 2, clone MGC:41494 IMAGE:1067302, mRNA, complete cds | 0.68 | 0,0368 |
| A\_51\_P266419 | AK014915 | inferred: IDN3 {Homo sapiens} | 0.68 | 0,0425 |
| A\_51\_P416647 | NM\_010115 | Mus musculus kallikrein 13 (Klk13), mRNA | 0.68 | 0,0022 |
| A\_51\_P520639 | NM\_177822 | weakly similar to SIMILAR TO PRE-PRO-MEGAKARYCYTE POTENTIATING FACTOR [Homo sapiens] | 0.68 | 0,0161 |
| A\_51\_P418935 | AK004524 | hypothetical SOCS domain, C-terminus of STAT-inhibitors containing protein | 0.68 | 0,0023 |
| A\_51\_P287939 | NM\_134090 | Mus musculus expressed sequence AI173274 (AI173274), mRNA | 0.68 | 0,0279 |
| A\_51\_P170156 | NM\_026614 | Mus musculus RIKEN cDNA 2900002J19 gene (2900002J19Rik), mRNA | 0.68 | 0,0434 |
| A\_51\_P295961 | NM\_001001182 | BROMODOMAIN ADJACENT TO ZINC FINGER DOMAIN 2B [Homo sapiens] | 0.68 | 0,0067 |
| A\_51\_P262858 | NM\_033620 | Mus musculus par-3 (partitioning defective 3) homolog (C. elegans) (Pard3), mRNA | 0.68 | 0,0079 |
| A\_51\_P441843 | NM\_145946 | Mus musculus, clone MGC:38195 IMAGE:5322726, mRNA, complete cds | 0.68 | 0,0142 |
| A\_51\_P352572 | NM\_019701 | Mus musculus chloride channel K1-like (Clcnk1l-pending), mRNA | 0.68 | 0,0189 |
| A\_51\_P420100 | NM\_025736 | Mus musculus RIKEN cDNA 4921531G14 gene (4921531G14Rik), mRNA | 0.68 | 0,0124 |
| A\_51\_P184314 | NM\_026390 | Mus musculus, Similar to RIKEN cDNA 1300013G12 gene, clone IMAGE:3582146, mRNA | 0.68 | 0,0249 |
| A\_51\_P482990 | NM\_145996 | similar to MODULATOR RECOGNITION FACTOR I (FRAGMENT) [Homo sapiens] | 0.68 | 0,0063 |
| A\_51\_P423732 | NM\_029035 | Mus musculus RIKEN cDNA 4930422J18 gene (4930422J18Rik), mRNA | 0.68 | 0,0049 |
| A\_51\_P292110 | AK085946 | AUTISM-RELATED PROTEIN 1 homolog [Homo sapiens] | 0.68 | 0,0017 |
| A\_51\_P461040 | NM\_028798 | similar to NICE-1 PROTEIN [Homo sapiens] | 0.67 | 0,0076 |
| A\_51\_P117439 | NM\_011847 | Mus musculus DnaJ (Hsp40) homolog, subfamily B, member 6 (Dnajb6), mRNA | 0.67 | 0,0177 |
| A\_51\_P317993 | Y17344 | Mus musculus acid phosphatase 1, soluble (Acp1), mRNA | 0.67 | 0,0077 |
| A\_51\_P181615 | NM\_178712 | similar to G-PROTEIN-COUPLED RECEPTOR HE6 PRECURSOR homolog [Homo sapiens] | 0.67 | 0,0302 |
| A\_51\_P335889 | NM\_026080 | Mus musculus RIKEN cDNA 3110030K20 gene (3110030K20Rik), mRNA | 0.67 | 0,0372 |
| A\_51\_P236769 | NM\_027109 | DEOXYRIBONUCLEASE I EC 3.1.21.1 DNASE | 0.67 | 0,0360 |
| A\_51\_P304437 | NM\_173019 | "6-PHOSPHOFRUCTO-2-KINASE/FRUCTOSE-2,6-BIPHOSPHATASE 4 (6PF-2-K/FRU- 2,6-P2ASE TESTIS-TYPE ISOZYME) [INCLUDES: 6-PHOSPHOFRUCTO-2-KINASE (EC 2.7.1.105)� FRUCTOSE-2,6-BISPHOSPHATASE (EC 3.1.3.46)] homolog [Rattus norvegicus]" | 0.67 | 0,0032 |
| A\_51\_P465582 | NM\_024257 | hypothetical Haloacid dehalogenase/epoxide hydrolase family containing protein | 0.67 | 0,0015 |
| A\_51\_P329198 | NM\_020521 | Mus musculus vomeronasal 1 receptor, B5 (V1rb5), mRNA | 0.67 | 0,0229 |
| A\_51\_P291210 | NM\_027373 | ACTIN FILAMENT ASSOCIATED | 0.67 | 0,0160 |
| A\_51\_P498558 | NM\_146028 | Mus musculus, similar to src homology three (SH3) and cysteine rich domain, clone MGC:38869 IMAGE:5361431, mRNA, complete cds | 0.67 | 0,0255 |
| A\_51\_P490788 | A\_51\_P490788 | Mus musculus cDNA, 5 end | 0.67 | 0,0134 |
| A\_51\_P275983 | BC027414 | Mus musculus RNA component of mitochondrial RNAase P, 1 (Rmrp1), misc RNA | 0.67 | 0,0489 |
| A\_51\_P132944 | BE980346 | Mus musculus cDNA, 3 end | 0.67 | 0,0376 |
| A\_51\_P131195 | X73372 | Mus musculus prolactin receptor (Prlr), mRNA | 0.67 | 0,0289 |
| A\_51\_P255271 | NM\_019821 | Mus musculus glycolipid transfer protein (Gltp-pending), mRNA | 0.67 | 0,0392 |
| A\_51\_P439426 | NM\_133360 | Mus musculus clone 677 acetyl-CoA carboxylase 265 mRNA, partial cds | 0.67 | 0,0381 |
| A\_51\_P278286 | NM\_009424 | Mus musculus Tnf receptor-associated factor 6 (Traf6), mRNA | 0.67 | 0,0481 |
| A\_51\_P301274 | NM\_026606 | hypothetical protein | 0.67 | 0,0150 |
| A\_51\_P118443 | AK051656 | similar to HYPOTHETICAL 18.6 KDA PROTEIN (FRAGMENT) [Homo sapiens] | 0.67 | 0,0150 |
| A\_51\_P367040 | NM\_026958 | RIKEN cDNA 1810035L17 gene | 0.67 | 0,0037 |
| A\_51\_P273096 | AK042259 | inferred: putative {Mus musculus} | 0.67 | 0,0034 |
| A\_51\_P233596 | AF323080 | Mus musculus resistin (Retn), mRNA | 0.67 | 0,0080 |
| A\_51\_P235968 | AK016462 | unclassifiable | 0.67 | 0,0491 |
| A\_51\_P458722 | XM\_484079 | ULTRA-HIGH SULPHUR KERATIN homolog [Mus musculus] | 0.67 | 0,0351 |
| A\_51\_P368394 | NM\_019964 | Mus musculus DnaJ (Hsp40) homolog, subfamily B, member 8 (Dnajb8), mRNA | 0.67 | 0,0028 |
| A\_51\_P277295 | NM\_027977 | INTEGRAL PLASMA MEMBRANE PROTEIN | 0.67 | 0,0083 |
| A\_51\_P163958 | NM\_011467 | Mus musculus sepiapterin reductase (Spr), mRNA | 0.67 | 0,0425 |
| A\_51\_P427794 | NM\_198649 | ACTIN BINDING LIM PROTEIN | 0.67 | 0,0266 |
| A\_51\_P229946 | AK038045 | unclassifiable | 0.67 | 0,0410 |
| A\_51\_P405733 | NM\_001011834 | Mus musculus olfactory receptor MOR177-12 (MOR177-12) pseudogene | 0.67 | 0,0167 |
| A\_51\_P460279 | XM\_131470 | hypothetical protein | 0.67 | 0,0041 |
| A\_51\_P161335 | NM\_009220 | Mus musculus spermiogenesis gene (Smy), mRNA | 0.67 | 0,0358 |
| A\_51\_P515492 | NM\_152824 | 45KDA SPLICING FACTOR homolog [Homo sapiens] | 0.67 | 0,0079 |
| A\_51\_P110381 | NM\_144943 | Mus musculus mRNA for C type lectin (langerin gene) | 0.67 | 0,0045 |
| A\_51\_P246627 | AK030188 | Kinesin superfamily protein 2B | 0.67 | 0,0011 |
| A\_51\_P453079 | NM\_148943 | Mus musculus mRNA for putative ubiquitin-specific protease (Usp9y gene) | 0.67 | 0,0195 |
| A\_51\_P388002 | NM\_021335 | U2 small nuclear ribonucleoprotein B | 0.67 | 0,0201 |
| A\_51\_P243921 | NM\_018773 | Mus musculus src family associated phosphoprotein 2 (Scap2), mRNA | 0.67 | 0,0157 |
| A\_51\_P160625 | NM\_001004436 | hypothetical protein | 0.67 | 0,0122 |
| A\_51\_P468939 | NM\_007831 | Mus musculus deleted in colorectal carcinoma (Dcc), mRNA | 0.67 | 0,0280 |
| A\_51\_P320479 | A\_51\_P320479 | Mus musculus cDNA, 3 end | 0.67 | 0,0254 |
| A\_51\_P212053 | NM\_173051 | SERINE PROTEASE INHIBITOR EIC | 0.67 | 0,0064 |
| A\_51\_P129160 | AK085720 | unknown EST | 0.67 | 0,0442 |
| A\_51\_P264477 | AK008163 | weakly similar to SPF31 [Homo sapiens] | 0.67 | 0,0067 |
| A\_51\_P313503 | NM\_147109 | Mus musculus olfactory receptor MOR7-2 (MOR7-2), mRNA | 0.67 | 0,0410 |
| A\_51\_P386448 | AK040480 | serine/threonine kinase 33 | 0.67 | 0,0269 |
| A\_51\_P399914 | BC039937 | proteasome (prosome, macropain) 26S subunit, non-ATPase, 1 | 0.67 | 0,0044 |
| A\_51\_P393747 | NM\_172689 | Mus musculus, Similar to RNA helicase, clone IMAGE:4950144, mRNA | 0.67 | 0,0019 |
| A\_51\_P277075 | NM\_027650 | Mus musculus RIKEN cDNA 4933405P08 gene (4933405P08Rik), mRNA | 0.67 | 0,0092 |
| A\_51\_P274992 | NM\_026578 | Mus musculus nucleolar protein family A, member 1 (H/ACA small nucleolar RNPs) (Nola1), mRNA | 0.67 | 0,0104 |
| A\_51\_P399538 | NM\_009795 | Mus musculus calpain, small subunit 1 (Capns1), mRNA | 0.67 | 0,0106 |
| A\_51\_P335306 | NM\_008910 | Mus musculus protein phosphatase 1A, magnesium dependent, alpha isoform (Ppm1a), mRNA | 0.67 | 0,0160 |
| A\_51\_P233145 | NM\_023546 | Mus musculus RIKEN cDNA 1700029H17 gene (1700029H17Rik), mRNA | 0.67 | 0,0070 |
| A\_51\_P311096 | AK031878 | Mus musculus, Similar to RIKEN cDNA 2410003K15 gene, clone IMAGE:4167150, mRNA | 0.67 | 0,0229 |
| A\_51\_P389878 | NM\_013795 | ATP synthase, H+ transporting, mitochondrial F0 complex, subunit g | 0.67 | 0,0199 |
| A\_51\_P248986 | NM\_133698 | Mus musculus hornerin mRNA, complete cds | 0.67 | 0,0112 |
| A\_51\_P395646 | NM\_144961 | Mus musculus myosin, heavy polypeptide 2, skeletal muscle, adult (Myh2), mRNA | 0.67 | 0,0188 |
| A\_51\_P235644 | NM\_054057 | Mus musculus proline synthetase co-transcribed (Prosc), mRNA | 0.67 | 0,0271 |
| A\_51\_P281134 | AB019028 | Mus musculus cofactor required for Sp1 transcriptional activation subunit 2 (150 kDa) (Crsp2), mRNA | 0.67 | 0,0411 |
| A\_51\_P303000 | NM\_172765 | hypothetical BTB/POZ domain containing protein | 0.67 | 0,0313 |
| A\_51\_P365694 | NM\_019491 | Mus musculus v-ral simian leukemia viral oncogene homolog A (ras related) (Rala), mRNA | 0.67 | 0,0128 |
| A\_51\_P473506 | NM\_173398 | PROBABLE G PROTEIN-COUPLED RECEPTOR H963 homolog [Homo sapiens] | 0.67 | 0,0052 |
| A\_51\_P461108 | NM\_148958 | similar to OXYSTEROL-BINDING PROTEIN-LIKE PROTEIN OSBPL10 [Homo sapiens] | 0.67 | 0,0019 |
| A\_51\_P476757 | NM\_198640 | Mouse mRNA sequence, partial cds | 0.67 | 0,0364 |
| A\_51\_P467398 | NAP057063-1 | Mus musculus olfactory receptor GA\_x5J8B7W72BC-33522-34417 (GA\_x5J8B7W72BC-33522-34417) pseudogene | 0.67 | 0,0329 |
| A\_51\_P334785 | NM\_175026 | Similar to interferon activated gene 203 [Mus musculus] | 0.67 | 0,0310 |
| A\_51\_P331028 | BC025009 | Mus musculus, clone IMAGE:3500618, mRNA, partial cds | 0.67 | 0,0383 |
| A\_51\_P507571 | NM\_013638 | Mus musculus protamine 3 (Prm3), mRNA | 0.67 | 0,0009 |
| A\_51\_P146208 | AI841821 | Mus musculus cDNA, 3 end | 0.67 | 0,0070 |
| A\_51\_P150879 | NM\_133743 | Mus musculus GPI-anchored metastasis-associated protein homolog (C4.4a-pending), mRNA | 0.67 | 0,0147 |
| A\_51\_P293729 | NM\_054102 | Mus musculus Nd1 (Nd1-pending), mRNA | 0.67 | 0,0342 |
| A\_51\_P245393 | NM\_145993 | H-L(3)MBT-LIKE PROTEIN (HYPOTHETICAL 79.1 KDA PROTEIN) homolog [Homo sapiens] | 0.67 | 0,0021 |
| A\_51\_P235139 | AK078669 | KELCH LIKE PROTEIN | 0.67 | 0,0044 |
| A\_51\_P494675 | NM\_028071 | Mus musculus coactosin-like protein (Clp-pending), mRNA | 0.66 | 0,0091 |
| A\_51\_P503042 | NM\_152229 | mtll=tailless homolog [mice, embryos, mRNA, 2026 nt] | 0.66 | 0,0482 |
| A\_51\_P129435 | AK007269 | hypothetical AT-rich interaction domain (ARID) containing protein | 0.66 | 0,0068 |
| A\_51\_P475748 | NM\_008412 | Mus musculus involucrin (Ivl), mRNA | 0.66 | 0,0169 |
| A\_51\_P293369 | NM\_009488 | Mus musculus putative pheromone receptor (VR1) mRNA, complete cds | 0.66 | 0,0198 |
| A\_51\_P384894 | NM\_001001332 | "Mus musculus cDNA clone IMAGE:2088069 5 similar to gb:X05978 CYSTATIN A (HUMAN) | 142.02 | 0,0102 |
| A\_51\_P272243 | NM\_033568 | ELL COMPLEX EAP30 SUBUNIT homolog [Homo sapiens] | 0.66 | 0,0190 |
| A\_51\_P504490 | NM\_172762 | weakly similar to DJ835G14.2 (KIAA0117 (HAL845) PROTEIN) (FRAGMENT) [Homo sapiens] | 0.66 | 0,0030 |
| A\_51\_P432180 | NM\_134038 | Mus musculus expressed sequence AW743111 (AW743111), mRNA | 0.66 | 0,0488 |
| A\_51\_P464900 | BC054735 | Mus musculus gamma-aminobutyric acid (GABA-B) receptor, 1 (Gabbr1), mRNA | 0.66 | 0,0058 |
| A\_51\_P408044 | NM\_007434 | Mus musculus thymoma viral proto-oncogene 2 (Akt2), mRNA | 0.66 | 0,0157 |
| A\_51\_P194099 | NM\_009381 | Mus musculus thyroid hormone responsive SPOT14 homolog (Rattus) (Thrsp), mRNA | 0.66 | 0,0048 |
| A\_51\_P488340 | NM\_016715 | Mus musculus thymic stromal-derived lymphopoietin, receptor (Tslpr), mRNA | 0.66 | 0,0340 |
| A\_51\_P415607 | NM\_010222 | Mus musculus FK506 binding protein 7 (23 kDa) (Fkbp7), mRNA | 0.66 | 0,0252 |
| A\_51\_P355801 | M77174 | Mus musculus perlecan (heparan sulfate proteoglycan 2) (Hspg2), mRNA | 0.66 | 0,0075 |
| A\_51\_P145230 | ENSMUST00000072181 | Mus musculus high mobility group box 1, related sequence 18 (Hmgb1-rs18) pseudogene | 0.66 | 0,0154 |
| A\_51\_P346304 | NM\_198169 | Mus musculus hypothetical protein LOC229004, mRNA (cDNA clone IMAGE:4953646), partial cds | 0.66 | 0,0006 |
| A\_51\_P317417 | AK087383 | unknown EST | 0.66 | 0,0235 |
| A\_51\_P447727 | NM\_027955 | weakly similar to GERM CELL-LESS 1 PROTEIN [Mus musculus] | 0.66 | 0,0329 |
| A\_51\_P345896 | NM\_133880 | Mus musculus expressed sequence AI507170 (AI507170), mRNA | 0.66 | 0,0236 |
| A\_51\_P448203 | NM\_013493 | Mus musculus cellular nucleic acid binding protein (Cnbp), mRNA | 0.66 | 0,0289 |
| A\_51\_P131093 | BF783780 | Mus musculus cDNA, 5 end | 0.66 | 0,0168 |
| A\_51\_P201308 | NM\_139296 | Mus musculus mRNA for dopamine beta-hydroxylase-like, complete cds | 0.66 | 0,0051 |
| A\_51\_P223656 | NM\_008484 | Mus musculus laminin, beta 3 (Lamb3), mRNA | 0.66 | 0,0340 |
| A\_51\_P248047 | NM\_008483 | Mus musculus laminin, beta 2 (Lamb2), mRNA | 0.66 | 0,0171 |
| A\_51\_P480309 | NM\_010168 | Mus musculus coagulation factor II (F2), mRNA | 0.66 | 0,0154 |
| A\_51\_P453736 | XM\_283314 | hypothetical protein | 0.66 | 0,0152 |
| A\_51\_P398053 | NM\_008627 | Mus musculus myeloid ecotropic viral integration site-related gene 2 (Mrg2), mRNA | 0.66 | 0,0122 |
| A\_51\_P323174 | NM\_178017 | hypothetical HMG1/2 (high mobility group) box containing protein | 0.66 | 0,0147 |
| A\_51\_P216313 | NM\_153794 | hypothetical protein | 0.66 | 0,0499 |
| A\_51\_P364984 | NM\_008065 | Mus musculus GA repeat binding protein, alpha (Gabpa), mRNA | 0.66 | 0,0118 |
| A\_51\_P183025 | NM\_133893 | Mus musculus 2-5 oligoadenylate synthetase 1D (Oas1d), mRNA | 0.66 | 0,0023 |
| A\_51\_P452119 | NM\_008498 | Mus musculus LIM homeobox protein 1 (Lhx1), mRNA | 0.66 | 0,0129 |
| A\_51\_P386058 | NM\_198168 | Mus musculus, Similar to protein phosphatase 2, regulatory subunit B (B56), beta isoform, clone IMAGE:5007577, mRNA, partial cds | 0.66 | 0,0282 |
| A\_51\_P230600 | NM\_201376 | Mus musculus squamous cell carcinoma antigen 2 (Scca2), mRNA | 0.66 | 0,0051 |
| A\_51\_P139280 | NM\_007773 | Mus musculus crystallin, beta B2 (Crybb2), mRNA | 0.66 | 0,0013 |
| A\_51\_P272227 | CN838888 | hypothetical protein | 0.66 | 0,0104 |
| A\_51\_P347206 | NM\_147088 | Mus musculus olfactory receptor MOR30-1 (MOR30-1), mRNA | 0.66 | 0,0092 |
| A\_51\_P182896 | NM\_028502 | Mus musculus RIKEN cDNA 1700013N18 gene (1700013N18Rik), mRNA | 0.66 | 0,0069 |
| A\_51\_P499683 | NM\_033523 | Mus musculus sprouty protein with EVH-1 domain 2, related sequence (Spred2-pending), mRNA | 0.66 | 0,0460 |
| A\_51\_P436689 | XM\_137955 | Similar to: Mus musculus, Similar to apolipoprotein B (including Ag(x) antigen), clone IMAGE:5052989, mRNA, partial cds | 0.66 | 0,0091 |
| A\_51\_P227342 | NM\_007876 | Mus musculus dipeptidase 1 (renal) (Dpep1), mRNA | 0.66 | 0,0026 |
| A\_51\_P474538 | AK087668 | unclassifiable | 0.66 | 0,0419 |
| A\_51\_P321250 | AK031195 | neoplastic progression 2 | 0.66 | 0,0142 |
| A\_51\_P370073 | AK052881 | unclassifiable | 0.66 | 0,0475 |
| A\_51\_P262079 | NM\_010394 | Mus musculus histocompatibility 2, Q region locus 7 (H2-Q7), mRNA | 0.66 | 0,0028 |
| A\_51\_P363714 | NM\_016786 | Mus musculus huntingtin interacting protein 2 (Hip2), mRNA | 0.66 | 0,0266 |
| A\_51\_P327570 | AK018881 | 1700066C05RIK PROTEIN homolog [Mus musculus] | 0.66 | 0,0255 |
| A\_51\_P424448 | NM\_019929 | SMT3 (supressor of mif two, 3) homolog 1 (S. cerevisiae) | 0.66 | 0,0430 |
| A\_51\_P351015 | NM\_010735 | Mus musculus lymphotoxin A (Lta), mRNA | 0.66 | 0,0259 |
| A\_51\_P208580 | NM\_133831 | Mus musculus, Similar to glioma tumor suppressor candidate region gene 2, clone MGC:28894 IMAGE:4912415, mRNA, complete cds | 0.66 | 0,0098 |
| A\_51\_P494447 | NM\_001025106 | Mus musculus cDNA, 5 end | 0.66 | 0,0110 |
| A\_51\_P251977 | NM\_134184 | Mus musculus vomeronasal 1 receptor, C29 (V1rc29), mRNA | 0.66 | 0,0126 |
| A\_51\_P224046 | AK013818 | PROTEIN TYROSINE PHOSPHATASE BETA PRECURSOR EC 3.1.3.48 R PTP | 0.66 | 0,0377 |
| A\_51\_P373043 | NM\_013898 | Mus musculus translocase of inner mitochondrial membrane 8 homolog a (yeast) (Timm8a), mRNA | 0.66 | 0,0063 |
| A\_51\_P226066 | AK016187 | hypothetical protein | 0.66 | 0,0300 |
| A\_51\_P368591 | NM\_053254 | Mus musculus transducin-like enhancer of split 6, homolog of Drosophila E(spl) (Tle6), mRNA | 0.66 | 0,0006 |
| A\_51\_P324120 | NM\_194342 | SAD1 UNC-84 DOMAIN PROTEIN 2 (FRAGMENT) homolog [Homo sapiens] | 0.66 | 0,0428 |
| A\_51\_P384754 | NM\_010697 | Mus musculus LIM domain binding 1 (Ldb1), mRNA | 0.66 | 0,0062 |
| A\_51\_P483118 | NM\_016660 | Mus musculus high mobility group AT-hook 1 (Hmga1), mRNA | 0.66 | 0,0286 |
| A\_51\_P379970 | NM\_007487 | Mus musculus ADP-ribosylation-like 4 (Arl4), mRNA | 0.66 | 0,0448 |
| A\_51\_P378348 | NM\_172695 | phospholipase A2, activating protein | 0.66 | 0,0242 |
| A\_51\_P248234 | NM\_008676 | Mus musculus next to the Brca1 (Nbr1), mRNA | 0.66 | 0,0025 |
| A\_51\_P113108 | NM\_007888 | Mus musculus dishevelled 2, dsh homolog (Drosophila) (Dvl2), mRNA | 0.66 | 0,0021 |
| A\_51\_P515585 | NM\_026017 | Mus musculus, clone MGC:25664 IMAGE:4486464, mRNA, complete cds | 0.66 | 0,0080 |
| A\_51\_P281930 | NM\_024255 | Mus musculus RIKEN cDNA 2610207I16 gene (2610207I16Rik), mRNA | 0.66 | 0,0368 |
| A\_51\_P495379 | NM\_177699 | Mus musculus, Similar to FH1/FH2 domain-containing protein, clone IMAGE:4503930, mRNA | 0.66 | 0,0015 |
| A\_51\_P520191 | NM\_022320 | Mus musculus G protein-coupled receptor 35 (Gpr35), mRNA | 0.66 | 0,0036 |
| A\_51\_P268529 | AF045741 | Mus musculus deoxyribonuclease II alpha (Dnase2a), mRNA | 0.66 | 0,0054 |
| A\_51\_P203200 | NM\_145129 | NEURONAL NICOTINIC ACETYLCHOLINE RECEPTOR ALPHA 3 SUBUNIT homolog [Mus musculus] | 0.66 | 0,0175 |
| A\_51\_P319154 | BC025170 | Mus musculus, clone IMAGE:4013674, mRNA | 0.66 | 0,0089 |
| A\_51\_P353042 | NM\_181589 | weakly similar to ORF FOR OVERLAPPING PROTEIN [Kennedya yellow mosaic virus] | 0.66 | 0,0184 |
| A\_51\_P211088 | AK037397 | unclassifiable | 0.66 | 0,0066 |
| A\_51\_P472937 | TC1536721 | Mus musculus cDNA clone IMAGE:5681032 3 | 0.66 | 0,0137 |
| A\_51\_P209118 | AK015736 | unknown EST | 0.66 | 0,0094 |
| A\_51\_P381527 | NM\_011958 | Mus musculus origin recognition complex, subunit 4 (Orc4), mRNA | 0.66 | 0,0228 |
| A\_51\_P400752 | NM\_010393 | Mus musculus histocompatibility 2, Q region locus 5 (H2-Q5), mRNA | 0.66 | 0,0110 |
| A\_51\_P312121 | NM\_011723 | Mus musculus xanthine dehydrogenase (Xdh), mRNA | 0.66 | 0,0028 |
| A\_51\_P199199 | NM\_031376 | Mus musculus B cell phosphoinositide 3-kinase adaptor (Bcap), mRNA | 0.66 | 0,0030 |
| A\_51\_P511985 | NM\_010092 | Mus musculus dual-specificity tyrosine-(Y)-phosphorylation regulated kinase 1b (Dyrk1b), mRNA | 0.66 | 0,0163 |
| A\_51\_P382781 | X67083 | Mus musculus DNA-damage inducible transcript 3 (Ddit3), mRNA | 0.66 | 0,0221 |
| A\_51\_P115905 | AK036480 | inferred: KIAA1742 protein {Homo sapiens} | 0.66 | 0,0055 |
| A\_51\_P499155 | NM\_177381 | unknown EST | 0.66 | 0,0174 |
| A\_51\_P488888 | NM\_013632 | Mus musculus purine-nucleoside phosphorylase (Pnp), mRNA | 0.66 | 0,0031 |
| A\_51\_P199593 | XM\_131409 | unknown EST | 0.66 | 0,0043 |
| A\_51\_P495212 | NM\_172442 | similar to DELTEX 2 (FRAGMENT) [Gallus gallus] | 0.66 | 0,0083 |
| A\_51\_P319470 | NM\_146331 | Mus musculus olfactory receptor MOR171-43 (MOR171-43) pseudogene | 0.66 | 0,0325 |
| A\_51\_P124103 | NM\_019633 | Mus musculus recombinant antineuraminidase single chain Ig VH and VL domains (LOC56304), mRNA | 0.66 | 0,0015 |
| A\_51\_P164270 | NM\_021336 | Mus musculus U2 small nuclear ribonucleoprotein polypeptide A (Snrpa1), mRNA | 0.66 | 0,0118 |
| A\_51\_P364778 | NM\_024187 | Mus musculus U2 small nuclear ribonucleoprotein auxiliary factor (U2AF),35 kDa (U2af1), mRNA | 0.66 | 0,0267 |
| A\_51\_P308351 | AK041604 | weakly similar to HYPOTHETICAL 39.6 KDA PROTEIN [Homo sapiens] | 0.66 | 0,0350 |
| A\_51\_P141930 | NM\_181413 | weakly similar to E2A-PBX1-ASSOCIATED PROTEIN (FRAGMENT) [Homo sapiens] | 0.66 | 0,0324 |
| A\_51\_P222380 | NM\_021436 | Mus musculus partial mRNA for tomoregulin-1 (M7365) (TR-1 gene) | 0.66 | 0,0136 |
| A\_51\_P185775 | NM\_028873 | Mus musculus, RIKEN cDNA 5730551F12 gene, clone MGC:19282 IMAGE:4016209, mRNA, complete cds | 0.66 | 0,0449 |
| A\_51\_P189959 | BC060645 | OXIDATIVE-STRESS RESPONSIVE 1 homolog [Homo sapiens] | 0.66 | 0,0384 |
| A\_51\_P300726 | NM\_011014 | Mus musculus opioid receptor, sigma 1 (Oprs1), mRNA | 0.66 | 0,0058 |
| A\_51\_P439612 | NM\_020266 | Mus musculus DnaJ (Hsp40) homolog, subfamily B, member 10 (Dnajb10), mRNA | 0.66 | 0,0084 |
| A\_51\_P460851 | XM\_130919 | weakly similar to ADP-RIBOSYLATION FACTOR (FRAGMENT) [Ajellomyces capsulata] | 0.66 | 0,0087 |
| A\_51\_P172752 | NM\_025663 | Mus musculus RIKEN cDNA 2610029K21 gene (2610029K21Rik), mRNA | 0.66 | 0,0096 |
| A\_51\_P195023 | NM\_144886 | Mus musculus, clone MGC:36427 IMAGE:5344620, mRNA, complete cds | 0.66 | 0,0010 |
| A\_51\_P381506 | NM\_172145 | hypothetical protein | 0.66 | 0,0247 |
| A\_51\_P287823 | NM\_011887 | Mus musculus sodium channel, voltage-gated, type XI, alpha polypeptide (Scn11a), mRNA | 0.66 | 0,0212 |
| A\_51\_P175567 | AK077691 | Mus musculus thymus expressed gene 3 (Thyex3-pending), mRNA | 0.66 | 0,0325 |
| A\_51\_P224534 | NM\_009643 | Mus musculus cDNA clone NIA:C0644C09 IMAGE:30024416 5 | 0.66 | 0,0088 |
| A\_51\_P491366 | NM\_026112 | ZINC FINGER PROTEIN 328 homolog [Homo sapiens] | 0.66 | 0,0062 |
| A\_51\_P316199 | NM\_054090 | Mus musculus olfactory receptor 73 (Olfr73), mRNA | 0.66 | 0,0070 |
| A\_51\_P295635 | BY730955 | hypothetical ICE-like protease (caspase) p20 domain containing protein | 0.66 | 0,0062 |
| A\_51\_P486971 | AK016804 | hypothetical protein | 0.66 | 0,0279 |
| A\_51\_P365578 | NM\_016891 | Mus musculus protein phosphatase 2 (formerly 2A), regulatory subunit A (PR 65), alpha isoform (Ppp2r1a), mRNA | 0.66 | 0,0355 |
| A\_51\_P346862 | NM\_018854 | Mus musculus RIKEN cDNA 0610009H04 gene (0610009H04Rik), mRNA | 0.66 | 0,0073 |
| A\_51\_P152013 | NM\_009234 | Mus musculus SRY-box containing gene 11 (Sox11), mRNA | 0.66 | 0,0118 |
| A\_51\_P426633 | NM\_153806 | Mus musculus, Similar to acidic 82 kDa protein mRNA, clone MGC:27716 IMAGE:2609541, mRNA, complete cds | 0.66 | 0,0270 |
| A\_51\_P339503 | NM\_009837 | Mus musculus chaperonin subunit 4 (delta) (Cct4), mRNA | 0.66 | 0,0468 |
| A\_51\_P449580 | AK033703 | high mobility group AT-hook 2, pseudogene 1 | 0.66 | 0,0279 |
| A\_51\_P228777 | AK172927 | hypothetical Aminotransferases class-II/Cell division control protein 15 (CDC15) containing protein | 0.66 | 0,0273 |
| A\_51\_P365516 | X06342 | Mus musculus serine protease inhibitor, Kazal type 3 (Spink3), mRNA | 0.66 | 0,0306 |
| A\_51\_P483231 | NM\_008413 | Mus musculus Janus kinase 2 (Jak2), mRNA | 0.66 | 0,0033 |
| A\_51\_P320509 | BC026716 | Mus musculus, clone IMAGE:4952780, mRNA | 0.66 | 0,0394 |
| A\_51\_P135296 | XM\_127913 | similar to HYPOTHETICAL 66.1 KDA PROTEIN [Macaca fascicularis] | 0.66 | 0,0009 |
| A\_51\_P295442 | NM\_021605 | Mus musculus NIMA (never in mitosis gene a)-related expressed kinase 7 (Nek7), mRNA | 0.66 | 0,0425 |
| A\_51\_P136028 | AK046838 | unclassifiable | 0.66 | 0,0014 |
| A\_51\_P407406 | NM\_023126 | Mus musculus cell line NK14 derived transforming oncogene (Mel), mRNA | 0.66 | 0,0067 |
| A\_51\_P384436 | XM\_355529 | similar to CALDECRIN PRECURSOR (EC 3.4.21.2) (CHYMOTRYPSIN C) (SERUM CALCIUM- DECREASING FACTOR) [Rattus norvegicus] | 0.66 | 0,0068 |
| A\_51\_P339074 | NM\_144813 | Mus musculus, Similar to sodium/calcium/potassium exchanger, clone MGC:27617 IMAGE:4504496, mRNA, complete cds | 0.66 | 0,0019 |
| A\_51\_P278018 | NM\_027338 | Mus musculus, clone MGC:19122 IMAGE:4210911, mRNA, complete cds | 0.66 | 0,0215 |
| A\_51\_P432724 | NM\_023597 | Mus musculus RIKEN cDNA 5430402I10 gene (5430402I10Rik), mRNA | 0.66 | 0,0274 |
| A\_51\_P210877 | AK003768 | PEST CONTAINING NUCLEAR | 0.65 | 0,0051 |
| A\_51\_P326709 | NM\_001025613 | ZINC FINGER PROTEIN CEZANNE homolog [Homo sapiens] | 0.65 | 0,0070 |
| A\_51\_P454696 | BC028441 | hypothetical protein | 0.65 | 0,0029 |
| A\_51\_P498242 | NM\_024242 | Mus musculus RIKEN cDNA 5430416A05 gene (5430416A05Rik), mRNA | 0.65 | 0,0213 |
| A\_51\_P410346 | NM\_008958 | Mus musculus patched homolog 2 (Ptch2), mRNA | 0.65 | 0,0495 |
| A\_51\_P146126 | BB697153 | Mus musculus cDNA, 3 end | 0.65 | 0,0466 |
| A\_51\_P125368 | NM\_008214 | Mus musculus histidyl tRNA synthetase (Hars), mRNA | 0.65 | 0,0232 |
| A\_51\_P275293 | NM\_008879 | Mus musculus plastin 2, L (Pls2), mRNA | 0.65 | 0,0033 |
| A\_51\_P290202 | NM\_008267 | Mus musculus homeo box B13 (Hoxb13), mRNA | 0.65 | 0,0029 |
| A\_51\_P326762 | NM\_133225 | Mus musculus peripherial benzodiazepine receptor associated protein (Pap7) mRNA, complete cds | 0.65 | 0,0329 |
| A\_51\_P210634 | NM\_023547 | Mus musculus RIKEN cDNA 2510009I23 gene (2510009I23Rik), mRNA | 0.65 | 0,0262 |
| A\_51\_P430952 | NM\_009068 | Mus musculus receptor (TNFRSF)-interacting serine-threonine kinase 1 (Ripk1), mRNA | 0.65 | 0,0132 |
| A\_51\_P127718 | NM\_015825 | Mus musculus SH3-binding domain glutamic acid-rich protein (Sh3bgr), mRNA | 0.65 | 0,0293 |
| A\_51\_P514561 | NM\_008972 | prothymosin alpha homolog [Mus musculus] | 0.65 | 0,0335 |
| A\_51\_P450628 | NM\_013700 | Mus musculus ubiquitin specific protease 5 (isopeptidase T) (Usp5), mRNA | 0.65 | 0,0096 |
| A\_51\_P264315 | NM\_009186 | Mus musculus silica-induced gene 41 (Silg41), mRNA | 0.65 | 0,0076 |
| A\_51\_P192979 | NM\_173408 | hypothetical protein | 0.65 | 0,0082 |
| A\_51\_P443322 | NM\_146200 | Mus musculus RIKEN cDNA 3230401O13 gene (3230401O13Rik), mRNA | 0.65 | 0,0205 |
| A\_51\_P188381 | NM\_177093 | hypothetical Leucine-rich repeat, outliers/Leucine-rich repeat/Leucine-rich repeat, typical subtype containing protein | 0.65 | 0,0297 |
| A\_51\_P183252 | NM\_080442 | Mus musculus testis-specific serine/threonine kinase 3b (Tssk3b) mRNA, complete cds | 0.65 | 0,0044 |
| A\_51\_P372073 | NM\_011730 | Mus musculus X transporter protein 2 (Xtrp2), mRNA | 0.65 | 0,0194 |
| A\_51\_P346516 | NM\_008974 | Mus musculus protein tyrosine phosphatase 4a2 (Ptp4a2), mRNA | 0.65 | 0,0011 |
| A\_51\_P457013 | NM\_145605 | Mus musculus, Similar to hypothetical protein DKFZp434G0522, clone MGC:19372 IMAGE:2631632, mRNA, complete cds | 0.65 | 0,0062 |
| A\_51\_P357858 | NM\_016676 | Mus musculus RAB10, member RAS oncogene family (Rab10), mRNA | 0.65 | 0,0027 |
| A\_51\_P245718 | XM\_145254 | Mus musculus, clone IMAGE:3488682, mRNA, partial cds | 0.65 | 0,0473 |
| A\_51\_P367763 | NM\_033602 | Mus musculus pellino 2 (Peli2), mRNA | 0.65 | 0,0044 |
| A\_51\_P101765 | NM\_031842 | Mus musculus SWI/SNF related, matrix associated, actin dependent regulator of chromatin, subfamily d, member 1 (Smarcd1), mRNA | 0.65 | 0,0237 |
| A\_51\_P180032 | NM\_009169 | Mus musculus split hand/foot deleted gene 1 (Shfdg1), mRNA | 0.65 | 0,0197 |
| A\_51\_P266237 | NM\_177566 | weakly similar to GUANINE NUCLEOTIDE REGULATORY PROTEIN (FRAGMENT) [Rattus norvegicus] | 0.65 | 0,0168 |
| A\_51\_P377154 | NM\_009997 | Mus musculus cytochrome P450, 2a4 (Cyp2a4), mRNA | 0.65 | 0,0032 |
| A\_51\_P103779 | NM\_145512 | hypothetical protein | 0.65 | 0,0077 |
| A\_51\_P335350 | NM\_027799 | musculus RIKEN cDNA 5530600A18 gene (5530600A18Rik), mRNA | 0.65 | 0,0204 |
| A\_51\_P249286 | NM\_011267 | Mus musculus, Similar to regulator of G-protein signaling 16, clone MGC:13876 IMAGE:4020813, mRNA, complete cds | 0.65 | 0,0387 |
| A\_51\_P269494 | NM\_146153 | THYROID HORMONE RECEPTOR-ASSOCIATED PROTEIN COMPLEX COMPONENT TRAP150 homolog [Homo sapiens] | 0.65 | 0,0152 |
| A\_51\_P440294 | BG173369 | hypothetical Zinc finger, C2H2 type containing protein | 0.65 | 0,0015 |
| A\_51\_P317391 | AK038197 | T-cell receptor delta chain precursor V region (2B4.Exp) (fragment) homolog [Mus musculus] | 0.65 | 0,0232 |
| A\_51\_P210864 | NAP108679-1 | Mus musculus olfactory receptor MOR264-11 (MOR264-11) pseudogene | 0.65 | 0,0012 |
| A\_51\_P292146 | NM\_177387 | DERMATAN/CHONDROITIN SULFATE 2-SULFOTRANSFERASE homolog [Homo sapiens] | 0.65 | 0,0109 |
| A\_51\_P143135 | NM\_175682 | hypothetical Haem peroxidase superfamily containing protein | 0.65 | 0,0017 |
| A\_51\_P484274 | NAP057143-1 | Mus musculus olfactory receptor GA\_x5J8B7W3KVV-170014-170572 (GA\_x5J8B7W3KVV-170014-170572) pseudogene | 0.65 | 0,0317 |
| A\_51\_P241725 | TC1449962 | Mus musculus cDNA, 3 end | 0.65 | 0,0091 |
| A\_51\_P208922 | NM\_011491 | Mus musculus stanniocalcin 2 (Stc2), mRNA | 0.65 | 0,0006 |
| A\_51\_P382524 | XM\_283873 | Mus musculus putative E1-E2 ATPase mRNA, partial cds | 0.65 | 0,0065 |
| A\_51\_P129100 | NM\_153055 | Mus musculus, Similar to SEC63 protein, clone MGC:30552 IMAGE:5066908, mRNA, complete cds | 0.65 | 0,0111 |
| A\_51\_P362029 | NM\_007649 | M.musculus Bcm-1 mRNA for BCM1 antigen | 0.65 | 0,0029 |
| A\_51\_P152765 | NM\_011488 | Mus musculus signal transducer and activator of transcription 5A (Stat5a), mRNA | 0.65 | 0,0091 |
| A\_51\_P224705 | BG244175 | Mus musculus cDNA, 5 end | 0.65 | 0,0442 |
| A\_51\_P338799 | NM\_172938 | hypothetical SAM domain (Sterile alpha motif) containing protein | 0.65 | 0,0468 |
| A\_51\_P211732 | NM\_008977 | Mus musculus protein tyrosine phosphatase, non-receptor type 2 (Ptpn2), mRNA | 0.65 | 0,0167 |
| A\_51\_P279197 | NM\_009124 | Mus musculus spinocerebellar ataxia 1 homolog (human) (Sca1), mRNA | 0.65 | 0,0036 |
| A\_51\_P154684 | NM\_011568 | Mus musculus RNA and export factor binding protein 1 (Refbp1), mRNA | 0.65 | 0,0031 |
| A\_51\_P279704 | BC024679 | Mus musculus, Similar to hypothetical gene LOC133157, clone IMAGE:3991705, mRNA, partial cds | 0.65 | 0,0021 |
| A\_51\_P107315 | NM\_145423 | Mus musculus similar to Sodium/iodide cotransporter (Na(+)/I(-) cotransporter) (Sodium-iodide symporter) (Na+/I-symporter) (LOC216225), mRNA | 0.65 | 0,0008 |
| A\_51\_P212135 | NM\_146763 | Mus musculus olfactory receptor MOR267-5 (MOR267-5), mRNA | 0.65 | 0,0059 |
| A\_51\_P297088 | AK033080 | cytosolic 5 nucleotidase, type 1A | 0.65 | 0,0358 |
| A\_51\_P319244 | D89080 | Mus musculus fibroblast growth factor 10 (Fgf10), mRNA | 0.65 | 0,0065 |
| A\_51\_P478419 | NM\_178680 | similar to UNC45-RELATED PROTEIN [Brachydanio rerio] | 0.65 | 0,0144 |
| A\_51\_P421984 | NM\_019818 | Mus musculus translocase of inner mitochondrial membrane 22 homolog (yeast) (Timm22), mRNA | 0.65 | 0,0025 |
| A\_51\_P339987 | NM\_175344 | hypothetical protein | 0.65 | 0,0031 |
| A\_51\_P494491 | NM\_008888 | Mus musculus paired mesoderm homeobox 2b (Pmx2b), mRNA | 0.65 | 0,0058 |
| A\_51\_P399664 | NM\_007803 | Mus musculus cortactin (Cttn), mRNA | 0.65 | 0,0161 |
| A\_51\_P407004 | NM\_025774 | Mus musculus RIKEN cDNA 8430424D23 gene (8430424D23Rik), mRNA | 0.65 | 0,0194 |
| A\_51\_P218556 | AK016089 | hypothetical protein | 0.65 | 0,0029 |
| A\_51\_P249867 | NM\_026382 | Mus musculus RIKEN cDNA 6530403A03 gene (6530403A03Rik), mRNA | 0.65 | 0,0100 |
| A\_51\_P372762 | NM\_010127 | Mus musculus POU domain, class 6, transcription factor 1 (Pou6f1), mRNA | 0.64 | 0,0110 |
| A\_51\_P504534 | NM\_007919 | Mus musculus elastase 2 (Ela2), mRNA | 0.64 | 0,0396 |
| A\_51\_P390255 | NM\_026444 | Mus musculus citrate synthase (Cs), mRNA | 0.64 | 0,0186 |
| A\_51\_P484832 | NM\_008014 | Mus musculus protein phosphatase 1G (formerly 2C), magnesium-dependent, gamma isoform (Ppm1g), mRNA | 0.64 | 0,0461 |
| A\_51\_P282706 | NM\_010477 | heat shock protein, 60 kDa | 0.64 | 0,0419 |
| A\_51\_P226374 | NM\_007516 | Mus musculus heterogeneous nuclear ribonucleoprotein D (Hnrpd), mRNA | 0.64 | 0,0008 |
| A\_51\_P140434 | NM\_027600 | hypothetical protein | 0.64 | 0,0028 |
| A\_51\_P436928 | NM\_029767 | SIMILAR TO RIBOSOMAL PROTEIN S9 (UNKNOWN) (PROTEIN FOR MGC:14341) (PROTEIN FOR MGC:2458) (PROTEIN FOR MGC:4138) homolog [Homo sapiens] | 0.64 | 0,0448 |
| A\_51\_P511480 | NM\_028680 | Mus musculus RIKEN cDNA 4833420A15 gene (4833420A15Rik), mRNA | 0.64 | 0,0098 |
| A\_51\_P249232 | NM\_008972 | Mus musculus prothymosin alpha (Ptma), mRNA | 0.64 | 0,0276 |
| A\_51\_P144382 | NM\_172086 | ribosomal protein L32 | 0.64 | 0,0272 |
[truncated: 177,338 more chars]
