# Supplementary material for: The functional genome of CA1 and CA3 neurons under native conditions and in response to ischemia
Source: BMC Genomics. 2007 Oct 15;8:370. doi: 10.1186/1471-2164-8-370 (PMC2194787; doi:10.1186/1471-2164-8-370)
Supplement: Additional file 5 — List of all significantly regulated genes in CA1 ischemic vs. sham. HTML file containing all genes significantly regulated in CA1 between the ischemic and the sham group. Given are Agilent probe numbers, accession numbers, gene names, enrichment factors ("M (ICA1/SCA1")), and false-discovery-rate corrected p-values. [file 1471-2164-8-370-S5.htm]

| Agilent probe# | Accession# | gene name | M (ICA1/SCA1) | P.fdr |
| A\_51\_P142972 | NM\_029947 | PR-DOMAIN CONTAINING PROTEIN 8 homolog [Homo sapiens] | 7.36 | 0,0019 |
| A\_51\_P144850 | NM\_178685 | PROTOCADHERIN 13 (FRAGMENT) homolog [Homo sapiens] | 6.45 | 0,0001 |
| A\_51\_P338874 | NM\_027571 | Mus musculus, purinergic receptor P2Y, G-protein coupled 12, clone MGC:36953 IMAGE:4947057, mRNA, complete cds | 5.70 | 0,0007 |
| A\_51\_P394997 | NM\_009717 | Mus musculus neurogenic differentiation 6 (Neurod6), mRNA | 5.54 | 0,0003 |
| A\_51\_P162124 | AK012595 | unknown EST | 5.24 | 0,0001 |
| A\_51\_P206835 | NM\_010687 | Mus musculus like-glycosyltransferase (Large), mRNA | 4.72 | 0,0000 |
| A\_51\_P495986 | NM\_025508 | Mus musculus guanosine monophosphate reductase (Gmpr), mRNA | 4.08 | 0,0000 |
| A\_51\_P427232 | NM\_183088 | ethanol induced 6 | 4.06 | 0,0001 |
| A\_51\_P335981 | NM\_026272 | Mus musculus, RIKEN cDNA 4430402O11 gene, clone MGC:27610 IMAGE:4503713, mRNA, complete cds | 4.00 | 0,0000 |
| A\_51\_P298131 | NM\_013926 | Mus musculus chromobox homolog 8 (Drosophila Pc class) (Cbx8), mRNA | 3.94 | 0,0004 |
| A\_51\_P341664 | NM\_133228 | Mus musculus KRAB zinc finger protein (Mzf22) (Mzf22), mRNA | 3.92 | 0,0005 |
| A\_51\_P400160 | AK013705 | hypothetical protein | 3.84 | 0,0001 |
| A\_51\_P389957 | NM\_016758 | Mus musculus regulator of G-protein signaling 14 (Rgs14), mRNA | 3.81 | 0,0021 |
| A\_51\_P515129 | XM\_620248 | hypothetical Sulfotransferase containing protein | 3.68 | 0,0029 |
| A\_51\_P220262 | BC066016 | unknown EST | 3.68 | 0,0002 |
| A\_51\_P274259 | AK053807 | ADENYLATE KINASE ISOZYME 5 homolog [Mus musculus] | 3.68 | 0,0039 |
| A\_51\_P372522 | NM\_001014995 | inferred: COTE1 PROTEIN. [Human] {Homo sapiens} | 3.63 | 0,0001 |
| A\_51\_P170987 | NM\_029879 | hypothetical protein | 3.58 | 0,0003 |
| A\_51\_P353914 | NM\_008319 | Mus musculus intercellular adhesion molecule 5, telencephalin (Icam5), mRNA | 3.53 | 0,0039 |
| A\_51\_P263004 | NM\_016707 | Mus musculus B-cell CLL/lymphoma 11A (zinc finger protein) (Bcl11a), mRNA | 3.51 | 0,0003 |
| A\_51\_P515242 | AK122459 | hypothetical protein | 3.51 | 0,0181 |
| A\_51\_P161946 | NM\_175332 | hypothetical protein | 3.46 | 0,0003 |
| A\_51\_P515532 | NM\_029881 | hypothetical alpha/beta-Hydrolases structure containing protein | 3.43 | 0,0029 |
| A\_51\_P302938 | NM\_145216 | RAS-LIKE PROTEIN RRP22 (RAS-RELATED PROTEIN ON CHROMOSOME 22) homolog [Homo sapiens] | 3.41 | 0,0045 |
| A\_51\_P221100 | NM\_198214 | KIAA0374 (SYNTAPHILIN) (BA314N13.1.1) homolog [Homo sapiens] | 3.39 | 0,0008 |
| A\_51\_P333859 | NM\_175448 | hypothetical General substrate transporters/Cellular retinaldehyde-binding protein (CRAL)/Triple function domain (TRIO) containing protein | 3.39 | 0,0000 |
| A\_51\_P216480 | NM\_178725 | weakly similar to BRAIN TUMOR ASSOCIATED PROTEIN NAG14 [Homo sapiens] | 3.36 | 0,0002 |
| A\_51\_P264676 | AK083198 | unknown EST | 3.36 | 0,0040 |
| A\_51\_P397983 | AK077026 | SODIUM/HYDROGEN EXCHANGER 2 (NA(+)/H(+) EXCHANGER 2) (NHE-2) (H7) homolog [Rattus norvegicus] | 3.29 | 0,0066 |
| A\_51\_P456098 | NM\_028223 | Mus musculus, RIKEN cDNA 3010001K23 gene, clone MGC:8187 IMAGE:3590497, mRNA, complete cds | 3.29 | 0,0001 |
| A\_51\_P231630 | NM\_001001792 | Mus musculus zinc finger protein 239 (Zfp239), mRNA | 3.29 | 0,0000 |
| A\_51\_P108383 | NM\_026649 | hypothetical protein | 3.27 | 0,0001 |
| A\_51\_P191779 | NM\_133859 | Mus musculus RIKEN cDNA 2810002E22 gene (2810002E22Rik), mRNA | 3.27 | 0,0002 |
| A\_51\_P507043 | NM\_021560 | Mus musculus basic helix-loop-helix domain containing, class B5 (Bhlhb5), mRNA | 3.27 | 0,0005 |
| A\_51\_P324551 | NM\_025785 | Mus musculus F-box only protein 25 (Fbxo25), mRNA | 3.25 | 0,0001 |
| A\_51\_P134045 | NM\_008792 | hypothetical protein | 3.25 | 0,0010 |
| A\_51\_P514405 | NM\_019741 | Mus musculus solute carrier family 2 (facilitated glucose transporter), member 5 (Slc2a5), mRNA | 3.25 | 0,0000 |
| A\_51\_P269663 | NM\_010585 | Mus musculus inositol 1,4,5-triphosphate receptor 1 (Itpr1), mRNA | 3.23 | 0,0023 |
| A\_51\_P374900 | NM\_028808 | G protein-coupled receptor 68 homolog [Homo sapiens] | 3.20 | 0,0052 |
| A\_51\_P152918 | NM\_009472 | Mus musculus unc5 homolog (C. elegans) 3 (Unc5h3), mRNA | 3.20 | 0,0003 |
| A\_51\_P229613 | NM\_008792 | Mus musculus proprotein convertase subtilisin/kexin type 2 (Pcsk2), mRNA | 3.18 | 0,0020 |
| A\_51\_P167313 | NM\_171824 | unknown EST | 3.18 | 0,0002 |
| A\_51\_P267634 | NM\_178751 | hypothetical protein | 3.16 | 0,0009 |
| A\_51\_P218902 | BC042507 | unknown EST | 3.14 | 0,0001 |
| A\_51\_P191439 | NM\_178927 | hypothetical protein | 3.14 | 0,0042 |
| A\_51\_P292490 | AK012069 | unknown EST | 3.14 | 0,0000 |
| A\_51\_P326994 | NM\_133746 | Mus musculus RIKEN cDNA 2810048G17 gene (2810048G17Rik), mRNA | 3.14 | 0,0002 |
| A\_51\_P209902 | NM\_177814 | hypothetical Histidine-rich region containing protein | 3.14 | 0,0013 |
| A\_51\_P226465 | NM\_145579 | Mus musculus Myb protein P42POP mRNA, complete cds | 3.12 | 0,0003 |
| A\_51\_P295085 | NM\_008760 | osteoglycin | 3.10 | 0,0248 |
| A\_51\_P414243 | NM\_153540 | hypothetical Fibronectin type III structure containing protein | 3.10 | 0,0001 |
| A\_51\_P345775 | NM\_178934 | similar to CDNA FLJ31992 FIS, CLONE NT2RP7009149, WEAKLY SIMILAR TO GLUCOSE TRANSPORTER TYPE 2, LIVER [Homo sapiens] | 3.10 | 0,0178 |
| A\_51\_P479652 | XM\_125901 | TBP-interacting protein | 3.05 | 0,0000 |
| A\_51\_P316951 | XM\_620310 | unknown EST | 3.05 | 0,0176 |
| A\_51\_P483438 | NM\_011348 | Mus musculus sema domain, immunoglobulin domain (Ig), short basic domain, secreted, (semaphorin) 3E (Sema3e), mRNA | 3.05 | 0,0139 |
| A\_51\_P403793 | NM\_021287 | Mus musculus beta III spectrin (Spnb3) mRNA, partial cds | 3.03 | 0,0015 |
| A\_51\_P430973 | NM\_027995 | hypothetical Uncharacterised protein family Hly-III/UPF0073 containing protein | 3.03 | 0,0017 |
| A\_51\_P303217 | NM\_026754 | hypothetical protein | 3.03 | 0,0018 |
| A\_51\_P383718 | NM\_030132 | hypothetical Domain of unknown function DUF94 containing protein | 3.01 | 0,0000 |
| A\_51\_P105877 | NM\_201600 | Mus musculus myosin Vb (Myo5b), mRNA | 3.01 | 0,0020 |
| A\_51\_P152685 | NM\_175561 | weakly similar to PECANEX 1 [Mus musculus] | 3.01 | 0,0018 |
| A\_51\_P485349 | NM\_145544 | Mus musculus, Similar to RAP1, GTP-GDP dissociation stimulator 1, clone MGC:18981 IMAGE:4008751, mRNA, complete cds | 3.01 | 0,0000 |
| A\_51\_P478003 | NM\_172555 | POLY(A) POLYMERASE GAMMA (EC 2.7.7.19) (NEO-POLY(A) POLYMERASE) homolog [Homo sapiens] | 2.99 | 0,0000 |
| A\_51\_P296995 | NM\_011510 | Mus musculus ATP-binding cassette protein (Abcc8) mRNA, partial cds | 2.97 | 0,0054 |
| A\_51\_P131008 | NM\_010408 | Mus musculus hyperpolarization-activated, cyclic nucleotide-gated K+ 1 (Hcn1), mRNA | 2.97 | 0,0054 |
| A\_51\_P442894 | AK048310 | ALDEHYDE DEHYDROGENASE, DIMERIC NADP PREFERRING EC 1.2.1.5 ALDH CLASS 3 | 2.97 | 0,0008 |
| A\_51\_P212741 | NM\_001014761 | unknown EST | 2.95 | 0,0003 |
| A\_51\_P121288 | NM\_021527 | Mus musculus McKusick-Kaufman syndrome protein (Mkks), mRNA | 2.95 | 0,0100 |
| A\_51\_P436817 | BC052075 | similar to R29144\_1 [Homo sapiens] | 2.93 | 0,0013 |
| A\_51\_P388822 | NM\_174998 | HIPPOCALCIN-LIKE PROTEIN 4 (HYPOTHETICAL 22.2 KDA PROTEIN) homolog [Homo sapiens] | 2.93 | 0,0102 |
| A\_51\_P474188 | NM\_198942 | Mus musculus, Similar to RIKEN cDNA 2810407E23 gene, clone IMAGE:4489006, mRNA, partial cds | 2.91 | 0,0001 |
| A\_51\_P196718 | NM\_009622 | Mus musculus cDNA, 5 end | 2.89 | 0,0076 |
| A\_51\_P218216 | AK031357 | ADRENAL GLAND PROTEIN AD-005 homolog [Homo sapiens] | 2.89 | 0,0002 |
| A\_51\_P352782 | AK017901 | unknown EST | 2.89 | 0,0036 |
| A\_51\_P233928 | NM\_008080 | Mus musculus UDP-N-acetyl-alpha-D-galactosamine:(N-acetylneuraminyl)- galactosylglucosylceramide-beta-1, 4-N-acetylgalactosaminyltransferase (Galgt1), mRNA | 2.89 | 0,0012 |
| A\_51\_P104798 | NM\_181074 | hypothetical RNI-like structure containing protein | 2.87 | 0,0320 |
| A\_51\_P138199 | AK018228 | unclassifiable, full insert sequence. | 2.87 | 0,0001 |
| A\_51\_P449507 | NM\_008445 | Mus musculus kinesin family member 3c (Kif3c), mRNA | 2.87 | 0,0003 |
| A\_51\_P459479 | BC058348 | hypothetical protein | 2.87 | 0,0080 |
| A\_51\_P141012 | AK173296 | hypothetical Cysteine-rich flanking region, C-terminal/Leucine-rich repeat/Leucine-rich repeat, typical subtype containing protein | 2.87 | 0,0023 |
| A\_51\_P442016 | AK050846 | DEPENDENT PROTEIN KINASE KINASE | 2.85 | 0,0020 |
| A\_51\_P128775 | AK008890 | unknown EST | 2.85 | 0,0002 |
| A\_51\_P394244 | XM\_484075 | inferred: Link guanine nucleotide exchange factor II {Homo sapiens} | 2.85 | 0,0082 |
| A\_51\_P496715 | NM\_029037 | hypothetical Eukaryotic protein kinase containing protein | 2.85 | 0,0000 |
| A\_51\_P273609 | NM\_146125 | Mus musculus, clone MGC:28924 IMAGE:3481738, mRNA, complete cds | 2.85 | 0,0143 |
| A\_51\_P410361 | AK030326 | Mus musculus cDNA, 5 end | 2.85 | 0,0044 |
| A\_51\_P320454 | NM\_146241 | similar to THYROTROPIN-RELEASING HORMONE DEGRADING ECTOENZYME (EC 3.4.19.6) (TRH- DEGRADING ECTOENZYME) (TRH-DE) (TRH-SPECIFIC AMINOPEPTIDASE) (THYROLIBERINASE) (PYROGLUTAMYL-PEPTIDASE II) (PAP-II) [Rattus norvegicus] | 2.85 | 0,0091 |
| A\_51\_P102987 | NM\_001002927 | Mouse spermatogenic-specific proenkephalin mRNA, complete cds | 2.85 | 0,0139 |
| A\_51\_P346747 | NM\_013919 | Mus musculus ubiquitin specific protease 21 (Usp21), mRNA | 2.85 | 0,0002 |
| A\_51\_P332742 | NM\_175347 | sarcoplasmic reticulum 53K glycoprotein precursor homolog [Oryctolagus cuniculus] | 2.83 | 0,0001 |
| A\_51\_P513311 | NM\_009107 | Mus musculus retinoid X receptor gamma (Rxrg), mRNA | 2.83 | 0,0085 |
| A\_51\_P252324 | NM\_020278 | Mus musculus leucine-rich, glioma inactivated 1 (Lgi1), mRNA | 2.83 | 0,0071 |
| A\_51\_P396696 | NM\_198417 | zinc finger protein 60 | 2.83 | 0,0002 |
| A\_51\_P462159 | NM\_134096 | Mus musculus expressed sequence AW049604 (AW049604), mRNA | 2.81 | 0,0002 |
| A\_51\_P497395 | NM\_145100 | hypothetical Snake toxin-like structure containing protein | 2.81 | 0,0143 |
| A\_51\_P509551 | L42339 | Mus musculus sodium channel 3 mRNA, complete cds | 2.81 | 0,0003 |
| A\_51\_P514270 | AK013768 | unknown EST | 2.81 | 0,0022 |
| A\_51\_P131358 | NM\_009151 | Mus musculus selectin, platelet (p-selectin) ligand (Selpl), mRNA | 2.81 | 0,0002 |
| A\_51\_P244923 | NM\_134149 | Mus musculus expressed sequence AI837181 (AI837181), mRNA | 2.79 | 0,0008 |
| A\_51\_P451957 | NM\_009947 | Mus musculus copine 6 (Cpne6), mRNA | 2.79 | 0,0401 |
| A\_51\_P318933 | NM\_007595 | Mus musculus calcium/calmodulin-dependent protein kinase II, beta (Camk2b), mRNA | 2.79 | 0,0067 |
| A\_51\_P320876 | NM\_025996 | Mus musculus RIKEN cDNA 2610100K07 gene (2610100K07Rik), mRNA | 2.79 | 0,0006 |
| A\_51\_P224530 | NM\_020610 | Mus musculus RIKEN cDNA A330103B05 gene (A330103B05Rik), mRNA | 2.79 | 0,0412 |
| A\_51\_P248403 | NM\_010871 | Mus musculus baculoviral IAP repeat-containing 1f (Birc1f), mRNA | 2.79 | 0,0054 |
| A\_51\_P148122 | NM\_027910 | Mus musculus RIKEN cDNA 1300011D16 gene (1300011D16Rik), mRNA | 2.77 | 0,0002 |
| A\_51\_P446132 | NM\_172601 | RAS RELATED PROTEIN RAB | 2.75 | 0,0003 |
| A\_51\_P108862 | NM\_012003 | Mus musculus COP9 (constitutive photomorphogenic) homolog, subunit 7a (Arabidopsis thaliana) (Cops7a), mRNA | 2.75 | 0,0000 |
| A\_51\_P275123 | NM\_030263 | Mus musculus hypothetical protein, MGC:6957 (BC003498), mRNA | 2.75 | 0,0085 |
| A\_51\_P216215 | NM\_007982 | Mus musculus PTK2 protein tyrosine kinase 2 (Ptk2), mRNA | 2.73 | 0,0000 |
| A\_51\_P337210 | NM\_138682 | Mus musculus LIBG-like protein (MBAG1), mRNA | 2.73 | 0,0005 |
| A\_51\_P159603 | NM\_207202 | hypothetical protein | 2.73 | 0,0004 |
| A\_51\_P388039 | NM\_013915 | Mus musculus zinc finger protein 238 (Zfp238), mRNA | 2.71 | 0,0037 |
| A\_51\_P182722 | NM\_016801 | Mus musculus syntaxin 1A (brain) (Stx1a), mRNA | 2.69 | 0,0040 |
| A\_51\_P120017 | BC057029 | Mus musculus, Similar to cyclic GMP stimulated phosphodiesterase, clone IMAGE:3598413, mRNA, partial cds | 2.69 | 0,0002 |
| A\_51\_P458638 | NM\_183136 | hypothetical Ovomucoid/PCI-1 like inhibitors structure containing protein | 2.69 | 0,0361 |
| A\_51\_P165934 | NM\_008831 | prohibitin | 2.69 | 0,0006 |
| A\_51\_P516095 | AK013508 | unknown EST | 2.68 | 0,0005 |
| A\_51\_P355416 | NM\_022656 | Mus musculus nischarin (Nisch), mRNA | 2.68 | 0,0002 |
| A\_51\_P243900 | NM\_016743 | Mus musculus nel-like 2 homolog (chicken) (Nell2), mRNA | 2.68 | 0,0109 |
| A\_51\_P393768 | XM\_148904 | OXYSTEROL BINDING PROTEIN | 2.68 | 0,0006 |
| A\_51\_P431491 | BC037132 | similar to EFERIN [Homo sapiens] | 2.68 | 0,0005 |
| A\_51\_P151675 | A\_51\_P151675 | Mus musculus cDNA, 5 end | 2.66 | 0,0003 |
| A\_51\_P406129 | AK019577 | unknown EST | 2.66 | 0,0003 |
| A\_51\_P179831 | NM\_175003 | SH3 DOMAIN BINDING PROTEIN 1 3BP | 2.66 | 0,0002 |
| A\_51\_P471498 | NM\_028049 | Mus musculus RIKEN cDNA 0610033L19 gene (0610033L19Rik), mRNA | 2.66 | 0,0002 |
| A\_51\_P306308 | NM\_028220 | hypothetical G-protein beta WD-40 repeats containing protein | 2.66 | 0,0014 |
| A\_51\_P243623 | AK003055 | weakly similar to BREAST CANCER METASTASIS-SUPPRESSOR 1 [Homo sapiens] | 2.66 | 0,0001 |
| A\_51\_P134627 | NM\_178283 | Mus musculus ankyrin repeat domain-containing SOCS box protein 13 (Asb13), mRNA | 2.64 | 0,0000 |
| A\_51\_P488768 | NM\_178696 | hypothetical Mitochondrial energy transfer proteins (carrier protein) containing protein | 2.64 | 0,0002 |
| A\_51\_P437737 | NM\_020046 | Mus musculus dihydroorotate dehydrogenase (Dhodh), mRNA | 2.64 | 0,0000 |
| A\_51\_P363537 | BC058094 | small optic lobes homolog (Drosophila) | 2.64 | 0,0004 |
| A\_51\_P468231 | AK003303 | musculus 18 days embryo whole body cDNA, RIKEN full-length enriched library, clone:1110002J03:unclassifiable transcript, full insert sequence | 2.64 | 0,0025 |
| A\_51\_P431046 | NM\_009182 | Mus musculus sialyltransferase 8 (alpha-2, 8-sialytransferase) C (Siat8c), mRNA | 2.62 | 0,0017 |
| A\_51\_P380750 | NM\_009824 | "Mus musculus core-binding factor, runt domain, alpha subunit 2� translocated to, 3 homolog (human) (Cbfa2t3h), mRNA" | 2.62 | 0,0150 |
| A\_51\_P224424 | NM\_172781 | KELCH-LIKE PROTEIN 4 homolog [Homo sapiens] | 2.62 | 0,0070 |
| A\_51\_P101474 | NM\_015799 | Mus musculus transferrin receptor 2 (Trfr2), mRNA | 2.60 | 0,0061 |
| A\_51\_P201607 | XM\_620331 | RING FINGER PROTEIN WITH LEUCINE ZIPPER RNF26 (RESERVED) homolog [Homo sapiens] | 2.60 | 0,0002 |
| A\_51\_P270355 | NM\_019978 | Mus musculus double cortin and calcium/calmodulin-dependent protein kinase-like 1 (Dcamkl1), mRNA | 2.58 | 0,0044 |
| A\_51\_P395948 | NM\_153579 | synaptic vesicle glycoprotein 2 b | 2.58 | 0,0082 |
| A\_51\_P246092 | NM\_027074 | hypothetical protein | 2.58 | 0,0300 |
| A\_51\_P265016 | NM\_023249 | Mus musculus peptidylprolyl isomerase (cyclophilin)-like 2 (Ppil2), mRNA | 2.58 | 0,0021 |
| A\_51\_P379341 | NM\_010895 | Mus musculus neurogenic differentiation 2 (Neurod2), mRNA | 2.58 | 0,0245 |
| A\_51\_P170463 | NM\_001025381 | Mus musculus cDNA, 3 end | 2.58 | 0,0028 |
| A\_51\_P262196 | NM\_011069 | Mus musculus peroxisomal biogenesis factor 11b (Pex11b), mRNA | 2.58 | 0,0000 |
| A\_51\_P232868 | NM\_176995 | hypothetical protein | 2.58 | 0,0055 |
| A\_51\_P180061 | NM\_028717 | Mus musculus amyotrophic lateral sclerosis 2 (juvenile) homolog (human) (Als2), mRNA | 2.58 | 0,0106 |
| A\_51\_P342906 | NM\_009164 | Mus musculus SH3-domain binding protein 1 (Sh3bp1), mRNA | 2.58 | 0,0005 |
| A\_51\_P499254 | BC027371 | hypothetical Nucleic acid-binding proteins structure containing protein | 2.57 | 0,0045 |
| A\_51\_P336282 | NM\_010934 | Mus musculus neuropeptide Y receptor Y1 (Npy1r), mRNA | 2.57 | 0,0172 |
| A\_51\_P133005 | TC1516362 | AMINO ACID TRANSPORTER NAT-2 homolog [Mus musculus] | 2.57 | 0,0002 |
| A\_51\_P515108 | TC1483082 | Mus musculus cDNA, 5 end | 2.57 | 0,0002 |
| A\_51\_P197509 | NM\_153594 | hypothetical Protein-L-isoaspartate(D-aspartate) O-methyltransferase containing protein | 2.57 | 0,0006 |
| A\_51\_P396331 | NM\_013681 | Mus musculus synapsin II (Syn2), mRNA | 2.57 | 0,0018 |
| A\_51\_P476687 | NM\_008527 | Mus musculus killer cell lectin-like receptor subfamily B member 1C (Klrb1c), mRNA | 2.55 | 0,0270 |
| A\_51\_P477682 | NM\_008939 | Mus musculus protease, serine, 12 neurotrypsin, (motopsin) (Prss12), mRNA | 2.55 | 0,0183 |
| A\_51\_P122085 | NM\_028846 | ubiquitin specific protease 20 | 2.55 | 0,0013 |
| A\_51\_P357980 | NM\_012014 | Mus musculus GRIN1 (Z16) mRNA, complete cds | 2.55 | 0,0354 |
| A\_51\_P290886 | NM\_007667 | Mus musculus cadherin 8 (Cdh8), mRNA | 2.55 | 0,0002 |
| A\_51\_P138671 | NM\_175244 | Mus musculus cDNA, 3 end | 2.55 | 0,0002 |
| A\_51\_P335750 | NM\_029716 | Mus musculus RIKEN cDNA 1700112L09 gene (1700112L09Rik), mRNA | 2.55 | 0,0019 |
| A\_51\_P508705 | NM\_033561 | Mus musculus Williams-Beuren syndrome chromosome region 1 homolog (human) (Wbscr1), mRNA | 2.53 | 0,0000 |
| A\_51\_P480390 | NM\_008579 | Mus musculus meiosis expressed gene 1 (Meg1), mRNA | 2.53 | 0,0002 |
| A\_51\_P193490 | NM\_145564 | SIMILAR TO F-BOX ONLY PROTEIN 21 homolog [Mus musculus] | 2.53 | 0,0001 |
| A\_51\_P418116 | NM\_146162 | Mus musculus, clone MGC:38046 IMAGE:5250899, mRNA, complete cds | 2.53 | 0,0011 |
| A\_51\_P144868 | NM\_029934 | Mus musculus, RIKEN cDNA 5730589L02 gene, clone MGC:32334 IMAGE:5028759, mRNA, complete cds | 2.53 | 0,0008 |
| A\_51\_P314143 | AB045325 | Mus musculus expressed sequence AI415388 (AI415388), mRNA | 2.53 | 0,0042 |
| A\_51\_P488180 | NM\_178678 | hypothetical Leucine-rich repeat, typical subtype containing protein | 2.51 | 0,0398 |
| A\_51\_P115471 | AK046043 | inferred: Similar to apolipoprotein L {Homo sapiens} | 2.51 | 0,0000 |
| A\_51\_P251205 | NM\_028627 | hypothetical protein | 2.51 | 0,0104 |
| A\_51\_P288009 | NM\_009604 | Mus musculus cholinergic receptor, nicotinic, gamma polypeptide (Chrng), mRNA | 2.51 | 0,0154 |
| A\_51\_P229499 | NM\_027452 | hypothetical Immunoglobulin and major histocompatibility complex domain containing protein | 2.51 | 0,0072 |
| A\_51\_P448881 | NM\_145528 | Mus musculus, clone MGC:6816 IMAGE:2648797, mRNA, complete cds | 2.51 | 0,0002 |
| A\_51\_P392303 | NM\_010733 | Mus musculus mRNA for leucine-rich repeat protein, partial cds | 2.51 | 0,0016 |
| A\_51\_P231511 | NM\_145123 | ASPIC PRECURSOR homolog [Homo sapiens] | 2.50 | 0,0067 |
| A\_51\_P202084 | NM\_008073 | Mus musculus gamma-aminobutyric acid (GABA-A) receptor, subunit gamma 2 (Gabrg2), mRNA | 2.50 | 0,0005 |
| A\_51\_P408471 | NM\_026638 | Mus musculus, Similar to CDP-diacylglycerol--inositol 3-phosphatidyltransferase (phosphatidylinositol synthase), clone MGC:36425 IMAGE:5343411, mRNA, complete cds | 2.50 | 0,0001 |
| A\_51\_P265778 | AK019528 | CHONDROITIN | 2.50 | 0,0206 |
| A\_51\_P182174 | NM\_019831 | Mus musculus zinc finger protein 261 (Zfp261), mRNA | 2.50 | 0,0007 |
| A\_51\_P384993 | NM\_175481 | GLUTAMATE RECEPTOR, IONOTROPIC KAINATE 4 PRECURSOR (GLUTAMATE RECEPTOR KA-1) (KA1) homolog [Rattus norvegicus] | 2.50 | 0,0256 |
| A\_51\_P221189 | NM\_178622 | hypothetical protein | 2.50 | 0,0011 |
| A\_51\_P223033 | NM\_027968 | F-BOX DOMAIN PROTEIN homolog [Homo sapiens] | 2.48 | 0,0064 |
| A\_51\_P346488 | BC085129 | synapsin II | 2.48 | 0,0004 |
| A\_51\_P428754 | NM\_008168 | Mus musculus glutamate receptor, ionotropic, kainate 5 (gamma 2) (Grik5), mRNA | 2.48 | 0,0074 |
| A\_51\_P189962 | NM\_026380 | unclassifiable | 2.48 | 0,0007 |
| A\_51\_P133245 | AK122294 | Mus musculus, Similar to KIAA0476 gene product, clone IMAGE:5365056, mRNA, partial cds | 2.48 | 0,0000 |
| A\_51\_P345362 | NM\_001003912 | RHOGEF GLUTAMATE TRANSPORT MODULATOR GTRAP48 homolog [Rattus norvegicus] | 2.48 | 0,0003 |
| A\_51\_P310850 | BC025841 | Mus musculus, clone IMAGE:5149318, mRNA, partial cds | 2.46 | 0,0017 |
| A\_51\_P263473 | NM\_011889 | Mus musculus septin 3 (Sept3), mRNA | 2.46 | 0,0003 |
| A\_51\_P363935 | AK021067 | unknown EST | 2.46 | 0,0020 |
| A\_51\_P389977 | NM\_015782 | Mus musculus small nuclear ribonucleoprotein polypeptide A (Snrpa), mRNA | 2.46 | 0,0000 |
| A\_51\_P493771 | AK046785 | hypothetical Prenyl group binding site (CAAX box) containing protein | 2.46 | 0,0022 |
| A\_51\_P268953 | AK035894 | hypothetical protein | 2.46 | 0,0018 |
| A\_51\_P219444 | NM\_013880 | Mus musculus PLC-L2 mRNA for phospholipase C-L2, complete cds | 2.46 | 0,0004 |
| A\_51\_P364391 | NM\_175184 | hypothetical protein | 2.46 | 0,0008 |
| A\_51\_P142744 | NM\_172294 | SULFATASE FP homolog [Rattus norvegicus] | 2.45 | 0,0214 |
| A\_51\_P357877 | NM\_175530 | hypothetical F-box domain containing protein | 2.45 | 0,0003 |
| A\_51\_P367240 | NM\_146173 | Mus musculus, Similar to transmembrane 4 superfamily member (tetraspan NET-7), clone MGC:30714 IMAGE:3981492, mRNA, complete cds | 2.45 | 0,0048 |
| A\_51\_P343851 | NM\_001013025 | TGF BETA RECEPTOR ASSOCIATED PROTEIN | 2.45 | 0,0002 |
| A\_51\_P303728 | NM\_172575 | ZINC FINGER PROTEIN 277 homolog [Homo sapiens] | 2.45 | 0,0000 |
| A\_51\_P496569 | NM\_178804 | Mus musculus SLIT2 (Slit2) mRNA, complete cds | 2.45 | 0,0369 |
| A\_51\_P490348 | NM\_025423 | Mus musculus RIKEN cDNA 1110059E24 gene (1110059E24Rik), mRNA | 2.45 | 0,0000 |
| A\_51\_P175988 | NM\_013561 | Mus musculus 5-hydroxytryptamine (serotonin) receptor 3A (Htr3a), mRNA | 2.45 | 0,0109 |
| A\_51\_P263419 | NM\_013705 | Mus musculus zinc finger protein 30 (Zfp30), mRNA | 2.45 | 0,0007 |
| A\_51\_P469950 | NM\_153070 | Mus musculus brain stress early protein (Gbi) mRNA, complete cds | 2.45 | 0,0001 |
| A\_51\_P304478 | NM\_173446 | hypothetical protein | 2.43 | 0,0027 |
| A\_51\_P284686 | XM\_485800 | unclassifiable | 2.43 | 0,0031 |
| A\_51\_P354500 | NM\_025828 | Mus musculus RIKEN cDNA 1300009F09 gene (1300009F09Rik), mRNA | 2.43 | 0,0003 |
| A\_51\_P313862 | NAP108668-1 | Mus musculus olfactory receptor MOR175-6 (MOR175-6) pseudogene | 2.43 | 0,0239 |
| A\_51\_P431885 | NM\_198412 | DnaJ (Hsp40) homolog, subfamily B, member 6 | 2.43 | 0,0027 |
| A\_51\_P410260 | NM\_008846 | Mus musculus phosphatidylinositol-4-phosphate 5-kinase, type 1 alpha (Pip5k1a), mRNA | 2.43 | 0,0058 |
| A\_51\_P437882 | NM\_016793 | Mus musculus zinc finger protein 98 (Zfp98), mRNA | 2.43 | 0,0001 |
| A\_51\_P110759 | NM\_009199 | solute carrier family 1, member 1 | 2.41 | 0,0003 |
| A\_51\_P147379 | NM\_133803 | Mus musculus expressed sequence C86324 (C86324), mRNA | 2.41 | 0,0006 |
| A\_51\_P513661 | AK082224 | unknown EST | 2.41 | 0,0012 |
| A\_51\_P291936 | NM\_008060 | Mus musculus alpha glucosidase 2, alpha neutral subunit (G2an), mRNA | 2.41 | 0,0002 |
| A\_51\_P417758 | AK085009 | Mus musculus cDNA, 3 end | 2.41 | 0,0145 |
| A\_51\_P202979 | NM\_033567 | Mus musculus cat eye syndrome chromosome region, candidate 6 homolog (human) (Cecr6), mRNA | 2.41 | 0,0316 |
| A\_51\_P442782 | NM\_080419 | Mus musculus immunoglobulin superfamily receptor PGRL (Pgrl) mRNA, complete cds | 2.41 | 0,0020 |
| A\_51\_P185332 | AK004510 | unknown EST | 2.41 | 0,0014 |
| A\_51\_P309534 | NM\_019781 | Mus musculus peroxisomal biogenesis factor 14 (Pex14), mRNA | 2.41 | 0,0000 |
| A\_51\_P196925 | NM\_009142 | Mus musculus small inducible cytokine subfamily D, 1 (Scyd1), mRNA | 2.41 | 0,0127 |
| A\_51\_P122582 | NM\_144915 | hypothetical Lipase containing protein | 2.41 | 0,0013 |
| A\_51\_P343208 | XM\_127466 | unknown EST | 2.41 | 0,0016 |
| A\_51\_P170795 | NM\_178739 | hypothetical G-protein beta WD-40 repeats containing protein | 2.41 | 0,0020 |
| A\_51\_P386090 | D50418 | Mouse mRNA for AREC3, partial cds | 2.41 | 0,0336 |
| A\_51\_P459465 | AK083260 | KIDINS220 homolog [Rattus norvegicus] | 2.41 | 0,0037 |
| A\_51\_P246124 | NM\_010113 | Mus musculus epidermal growth factor (Egf), mRNA | 2.39 | 0,0089 |
| A\_51\_P175871 | NM\_010325 | Mus musculus glutamate oxaloacetate transaminase 2, mitochondrial (Got2), mRNA | 2.39 | 0,0001 |
| A\_51\_P276598 | NM\_133886 | Mus musculus, Similar to hypothetical protein FLJ14225, clone IMAGE:5132616, mRNA, partial cds | 2.39 | 0,0002 |
| A\_51\_P371408 | NM\_029842 | similar to THYRO1000124 PROTEIN [Homo sapiens] | 2.39 | 0,0390 |
| A\_51\_P465591 | BC096533 | METABOTROPIC GLUTAMATE RECEPTOR MGLUR5 PRECURSOR homolog [Rattus norvegicus] | 2.39 | 0,0297 |
| A\_51\_P110699 | NM\_170756 | SPERMATOGENESIS-ASSOCIATED PROTEIN 2 homolog [Rattus norvegicus] | 2.39 | 0,0013 |
| A\_51\_P449133 | NM\_013464 | Mus musculus aryl-hydrocarbon receptor (Ahr), mRNA | 2.39 | 0,0008 |
| A\_51\_P217115 | NM\_176834 | inferred: hypothetical protein {Homo sapiens} | 2.39 | 0,0023 |
| A\_51\_P240019 | NM\_013755 | Mus musculus glycogenin 1 (Gyg1), mRNA | 2.39 | 0,0002 |
| A\_51\_P322115 | NM\_010483 | Mus musculus 5-hydroxytryptamine (serotonin) receptor 5B (Htr5b), mRNA | 2.39 | 0,0069 |
| A\_51\_P421300 | AK002979 | D1 DOPAMINE RECEPTOR-INTERACTING PROTEIN CALCYON homolog [Mus musculus] | 2.38 | 0,0092 |
| A\_51\_P126437 | NM\_007930 | RIKEN full-length enriched, 10 days neonate brain Mus musculus cDNA clone K630055B09 3 | 2.38 | 0,0241 |
| A\_51\_P464021 | NM\_021528 | Mus musculus chondroitin 4-sulfotransferase 2 (C4st2-pending), mRNA | 2.38 | 0,0022 |
| A\_51\_P247153 | NM\_008258 | Mus musculus hematological and neurological expressed sequence 1 (Hn1), mRNA | 2.38 | 0,0007 |
| A\_51\_P202592 | NM\_172628 | hypothetical Tetratricopeptide repeat (TPR) structure containing protein | 2.38 | 0,0208 |
| A\_51\_P408653 | NM\_145463 | hypothetical protein | 2.38 | 0,0453 |
| A\_51\_P437079 | NM\_028872 | hypothetical protein | 2.36 | 0,0319 |
| A\_51\_P359237 | NM\_025439 | Mus musculus RIKEN cDNA 1500015G18 gene (1500015G18Rik), mRNA | 2.36 | 0,0016 |
| A\_51\_P139364 | AK087995 | similar to B4-2 PROTEIN [Homo sapiens] | 2.36 | 0,0009 |
| A\_51\_P260499 | NM\_183019 | similar to APC-STIMULATED GUANINE NUCLEOTIDE EXCHANGE FACTOR [Homo sapiens] | 2.36 | 0,0016 |
| A\_51\_P431023 | BC027561 | Mus musculus, Similar to ubiquitin associated and SH3 domain containing, A, clone IMAGE:3375785, mRNA | 2.36 | 0,0260 |
| A\_51\_P325862 | NM\_013751 | musculus 10, 11 days embryo whole body cDNA, RIKEN full-length enriched library, clone:2810012B06:unclassifiable transcript, full insert sequence | 2.36 | 0,0005 |
| A\_51\_P265676 | NM\_030113 | Mus musculus Rho GTPase activating protein 10 (Arhgap10), mRNA | 2.36 | 0,0217 |
| A\_51\_P231920 | NM\_001013374 | HYPOTHETICAL 39.7 KDA PROTEIN homolog [Homo sapiens] | 2.36 | 0,0031 |
| A\_51\_P282673 | NM\_175098 | weakly similar to N-ACETYLGLUCOSAMINYLTRANSFERASE (FRAGMENT) [Homo sapiens] | 2.35 | 0,0002 |
| A\_51\_P256747 | NM\_173029 | SOLUBLE ADENYLYL CYCLASE homolog [Rattus norvegicus] | 2.35 | 0,0146 |
| A\_51\_P331886 | AK038316 | inferred: human CLASP-5 {Homo sapiens} | 2.35 | 0,0193 |
| A\_51\_P406031 | NM\_013468 | ankyrin-like repeat protein | 2.35 | 0,0453 |
| A\_51\_P245895 | NM\_172632 | MITOGEN-ACTIVATED PROTEIN KINASE 4 (EC 2.7.1.-) (EXTRACELLULAR SIGNAL- REGULATED KINASE 4) (ERK-4) (MAP KINASE ISOFORM P63) (P63-MAPK) homolog [Homo sapiens] | 2.35 | 0,0013 |
| A\_51\_P255441 | NM\_053164 | Mus musculus mitochondrial ribosomal protein L43 (Mrpl43), mRNA | 2.35 | 0,0042 |
| A\_51\_P221062 | AK081805 | protein kinase, cAMP dependent regulatory, type II beta | 2.35 | 0,0211 |
| A\_51\_P369154 | NM\_144926 | SIMILAR TO TYPE I TRANSMEMBRANE RECEPTOR (SEIZURE-RELATED PROTEIN) homolog [Mus musculus] | 2.35 | 0,0004 |
| A\_51\_P160576 | AK014760 | unknown EST | 2.35 | 0,0010 |
| A\_51\_P444379 | NM\_198294 | hypothetical Serine-rich region containing protein | 2.35 | 0,0011 |
| A\_51\_P202530 | NM\_146127 | hypothetical protein | 2.33 | 0,0000 |
| A\_51\_P224406 | NM\_026441 | Mus musculus RIKEN cDNA 2600002E23 gene (2600002E23Rik), mRNA | 2.33 | 0,0008 |
| A\_51\_P358940 | NM\_016852 | Mus musculus WW domain binding protein 2 (Wbp2), mRNA | 2.33 | 0,0006 |
| A\_51\_P329811 | NM\_009042 | Mus musculus regenerating islet-derived 1 (Reg1), mRNA | 2.33 | 0,0181 |
| A\_51\_P415475 | NM\_053246 | Mus musculus downstream of tyrosine kinase 4 (Dok4), mRNA | 2.33 | 0,0009 |
| A\_51\_P269084 | NM\_175329 | similar to N27C7-4 PROTEIN [Homo sapiens] | 2.33 | 0,0017 |
| A\_51\_P137094 | NM\_015772 | Mus musculus sal-like 2 (Drosophila) (Sall2), mRNA | 2.33 | 0,0028 |
| A\_51\_P386069 | NM\_176971 | RAS-RELATED PROTEIN RAB-9L (RAB9-LIKE PROTEIN) homolog [Homo sapiens] | 2.33 | 0,0018 |
| A\_51\_P210082 | NM\_015769 | Mus musculus excision repair cross-complementing rodent repair deficiency, complementation group 4 (Ercc4), mRNA | 2.33 | 0,0012 |
| A\_51\_P150905 | NM\_145479 | hypothetical BTB/POZ domain and hypothetical Kelch repeat containing protein | 2.33 | 0,0015 |
| A\_51\_P106859 | NM\_138721 | Mus musculus U7 snRNP-specific Sm-like (Lsm10), mRNA | 2.33 | 0,0109 |
| A\_51\_P296878 | NM\_173746 | hypothetical protein | 2.33 | 0,0001 |
| A\_51\_P454913 | AK085143 | SIMILAR TO VESICLE-ASSOCIATED CALMODULIN-BINDING PROTEIN homolog [Mus musculus] | 2.33 | 0,0024 |
| A\_51\_P422335 | NM\_172740 | hypothetical Zinc finger, C2H2 type containing protein | 2.31 | 0,0333 |
| A\_51\_P211526 | NM\_026889 | HYPOTHETICAL PROTEIN FLJ22170 homolog [Mus musculus] | 2.31 | 0,0011 |
| A\_51\_P502975 | NM\_146372 | Mus musculus olfactory receptor MOR267-15 (MOR267-15) pseudogene | 2.31 | 0,0070 |
| A\_51\_P227275 | NM\_007786 | Mus musculus casein kappa (Csnk), mRNA | 2.31 | 0,0225 |
| A\_51\_P127738 | AK032187 | sodium channel protein II homolog [Rattus norvegicus] | 2.31 | 0,0179 |
| A\_51\_P355906 | NM\_021099 | Mus musculus kit oncogene (Kit), mRNA | 2.31 | 0,0414 |
| A\_51\_P177242 | NM\_021468 | Mus musculus unc13 homolog (C. elegans) 1 (Unc13h1), mRNA | 2.31 | 0,0001 |
| A\_51\_P486449 | NM\_133910 | Mus musculus expressed sequence AU043625 (AU043625), mRNA | 2.31 | 0,0004 |
| A\_51\_P232474 | NM\_028028 | similar to DJ337O18.5.2 (NOVEL PROTEIN, ISOFORM 2) [Homo sapiens] | 2.31 | 0,0007 |
| A\_51\_P137947 | NM\_009441 | Mus musculus tetratricopeptide repeat domain (Ttc3), mRNA | 2.31 | 0,0008 |
| A\_51\_P178011 | AK220333 | hypothetical protein | 2.31 | 0,0008 |
| A\_51\_P438293 | BC038250 | Mus musculus cDNA, 5 end | 2.30 | 0,0009 |
| A\_51\_P142989 | AK083314 | weakly similar to KRAB ZINC FINGER PROTEIN [Mus musculus] | 2.30 | 0,0180 |
| A\_51\_P232790 | AK030381 | hypothetical EF-hand containing protein | 2.30 | 0,0074 |
| A\_51\_P437549 | NM\_144876 | KELCH-LIKE PROTEIN X homolog [Homo sapiens] | 2.30 | 0,0005 |
| A\_51\_P460768 | AK089959 | hypothetical SET domain profile/MYND zinc finger (ZnF) domain/Cytochrome c family heme-binding site containing protein | 2.30 | 0,0017 |
| A\_51\_P444633 | NM\_029653 | Mus musculus, Similar to death-associated protein kinase 1, clone IMAGE:5345390, mRNA, partial cds | 2.30 | 0,0127 |
| A\_51\_P152623 | NM\_008552 | Mus musculus MAS1 oncogene (Mas1), mRNA | 2.30 | 0,0051 |
| A\_51\_P167374 | NM\_026181 | Mus musculus RIKEN cDNA 1300003A17 gene (1300003A17Rik), mRNA | 2.30 | 0,0004 |
| A\_51\_P240041 | NM\_011458 | Mouse mRNA for contrapsin | 2.30 | 0,0285 |
| A\_51\_P509747 | NM\_023638 | Mus musculus Mporc-d mRNA for porcupine-D, complete cds | 2.30 | 0,0006 |
| A\_51\_P228817 | NM\_025303 | Mus musculus staufen (RNA binding protein) homolog 2 (Drosophila) (Stau2), mRNA | 2.30 | 0,0008 |
| A\_51\_P267877 | NM\_139228 | Mus musculus rhomboid like gene 4 (Drosophila) (Rhbdl4), mRNA | 2.30 | 0,0008 |
| A\_51\_P454152 | AK048416 | hypothetical Winged helix DNA-binding domain structure containing protein | 2.30 | 0,0053 |
| A\_51\_P322877 | NM\_133816 | Mus musculus expressed sequence AI594717 (AI594717), mRNA | 2.30 | 0,0033 |
| A\_51\_P322542 | AK017436 | unknown EST | 2.28 | 0,0013 |
| A\_51\_P384159 | NM\_001010826 | Mus musculus, Similar to hypothetical protein MGC2376, clone MGC:37717 IMAGE:5066294, mRNA, complete cds | 2.28 | 0,0166 |
| A\_51\_P236160 | NM\_172392 | Mus musculus, Similar to zinc finger protein 208, clone MGC:40747 IMAGE:5365910, mRNA, complete cds | 2.28 | 0,0015 |
| A\_51\_P252107 | NM\_173748 | CDNA FLJ14164 FIS, CLONE NT2RP1000460, WEAKLY SIMILAR TO NUCLEAR MOVEMENT PROTEIN NUDC homolog [Homo sapiens] | 2.28 | 0,0019 |
| A\_51\_P453817 | AK082735 | unknown EST | 2.28 | 0,0025 |
| A\_51\_P497039 | NM\_007863 | Mus musculus membrane protein, palmitoylated 3 (MAGUK p55 subfamily member 3) (Mpp3), mRNA | 2.28 | 0,0048 |
| A\_51\_P355765 | AK019479 | hypothetical protein | 2.28 | 0,0275 |
| A\_51\_P109335 | NM\_009938 | Mus musculus, Similar to alpha-coatomer protein, clone IMAGE:3498647, mRNA, partial cds | 2.28 | 0,0006 |
| A\_51\_P494037 | NM\_138650 | Mus musculus diacylglycerol kinase, gamma 3 (Dagk3), mRNA | 2.27 | 0,0248 |
| A\_51\_P172565 | NM\_025300 | Mus musculus, mitochondrial ribosomal protein L15, clone MGC:29279 IMAGE:3498041, mRNA, complete cds | 2.27 | 0,0062 |
| A\_51\_P466858 | AK079284 | weakly similar to HYPOTHETICAL 70.3 KDA PROTEIN (FRAGMENT) [Homo sapiens] | 2.27 | 0,0007 |
| A\_51\_P308308 | NM\_172958 | PHOSPHATIDYLINOSITOL-3 PHOSPHATE 3-PHOSPHATASE ADAPTOR SUBUNIT homolog [Homo sapiens] | 2.27 | 0,0009 |
| A\_51\_P156526 | AK032322 | PLASMA MEMBRANE CALCIUM-TRANSPORTING ATPASE 3 (EC 3.6.3.8) (PMCA3) (PLASMA MEMBRANE CALCIUM PUMP ISOFORM 3) (PLASMA MEMBRANE CALCIUM ATPASE ISOFORM 3) homolog [Rattus norvegicus] | 2.27 | 0,0023 |
| A\_51\_P192955 | BG921888 | CD166 ANTIGEN PRECURSOR (ACTIVATED LEUKOCYTE-CELL ADHESION MOLECULE) (ALCAM) (DM-GRASP PROTEIN) | 2.27 | 0,0051 |
| A\_51\_P451196 | AK002502 | Mus musculus RIKEN cDNA 0610010M13 gene (0610010M13Rik), mRNA | 2.27 | 0,0466 |
| A\_51\_P420122 | NM\_178631 | weakly similar to SIMILAR TO HETEROGENEOUS NUCLEAR RIBONUCLEOPROTEIN C [Homo sapiens] | 2.27 | 0,0051 |
| A\_51\_P211854 | NM\_011347 | Mus musculus selectin, platelet (Selp), mRNA | 2.27 | 0,0111 |
| A\_51\_P366227 | NM\_177359 | cytosolic 5 nucleotidase, type 1A | 2.27 | 0,0082 |
| A\_51\_P109709 | NM\_146750 | Mus musculus olfactory receptor MOR40-11 (MOR40-11) pseudogene | 2.27 | 0,0126 |
| A\_51\_P146440 | NM\_146122 | Mus musculus, Similar to KIAA1608 protein, clone MGC:38416 IMAGE:5346095, mRNA, complete cds | 2.27 | 0,0044 |
| A\_51\_P265343 | AF114379 | Mus musculus LIM domain binding 3 (Ldb3), mRNA | 2.27 | 0,0038 |
| A\_51\_P324303 | NM\_153789 | Mus musculus, Similar to myosin regulatory light chain interacting protein, clone MGC:11702 IMAGE:3964381, mRNA, complete cds | 2.25 | 0,0025 |
| A\_51\_P489629 | AF155547 | Mus musculus erythrocyte protein band 4.9 (Epb4.9), mRNA | 2.25 | 0,0014 |
| A\_51\_P200408 | BC003939 | Mus musculus RIKEN cDNA 2810021O11 gene (2810021O11Rik), mRNA | 2.25 | 0,0004 |
| A\_51\_P330016 | NM\_015814 | dickkopf homolog 3 (Xenopus laevis) | 2.25 | 0,0057 |
| A\_51\_P456280 | NM\_177708 | hypothetical Leucine-rich repeat containing protein | 2.25 | 0,0219 |
| A\_51\_P352894 | AK085726 | BREFELDIN A-INHIBITED GUANINE NUCLEOTIDE-EXCHANGE PROTEIN 1 (BREFELDIN A-INHIBITED GEP 1) (P200 ARF-GEP1) (P200 ARF GUANINE NUCLEOTIDE EXCHANGE FACTOR) homolog [Homo sapiens] | 2.25 | 0,0005 |
| A\_51\_P171286 | AK037232 | unknown EST | 2.25 | 0,0166 |
| A\_51\_P496400 | NM\_138675 | Mus musculus FLJ10193 (FLJ10193), mRNA | 2.25 | 0,0001 |
| A\_51\_P512630 | BC035276 | 7ACOMP PROTEIN homolog [Rattus sp] | 2.25 | 0,0021 |
| A\_51\_P264634 | AK082675 | hypothetical protein | 2.25 | 0,0001 |
| A\_51\_P237834 | AK028322 | hypothetical protein | 2.25 | 0,0017 |
| A\_51\_P181341 | NM\_054095 | Mus musculus neuronal calcium binding 2 (Necab2-pending), mRNA | 2.23 | 0,0044 |
| A\_51\_P427624 | NM\_025973 | Mus musculus RIKEN cDNA 2210410L06 gene (2210410L06Rik), mRNA | 2.23 | 0,0121 |
| A\_51\_P397834 | NM\_010072 | dolichol-phosphate (beta-D) mannosyltransferase 1 | 2.23 | 0,0459 |
| A\_51\_P455421 | NM\_013871 | Mus musculus mitogen-activated protein kinase 12 (Mapk12), mRNA | 2.23 | 0,0382 |
| A\_51\_P408172 | XM\_356184 | hypothetical Integrin A (or I) domain structure containing protein | 2.23 | 0,0248 |
| A\_51\_P490148 | NM\_153424 | hypothetical protein | 2.23 | 0,0009 |
| A\_51\_P354913 | NM\_027402 | hypothetical Fibronectin type III domain containing protein | 2.23 | 0,0017 |
| A\_51\_P135877 | BC005577 | hypothetical P-loop containing nucleotide triphosphate hydrolases structure containing protein | 2.23 | 0,0141 |
| A\_51\_P148355 | NM\_175433 | hypothetical Zinc finger, C2H2 type containing protein | 2.23 | 0,0018 |
| A\_51\_P501793 | NM\_008237 | Mus musculus hairy and enhancer of split 3, (Drosophila) (Hes3), mRNA | 2.23 | 0,0133 |
| A\_51\_P148388 | NM\_017477 | Mus musculus coatomer protein complex, subunit gamma 1 (Copg1), mRNA | 2.23 | 0,0011 |
| A\_51\_P517198 | NM\_009959 | Mus musculus protocadherin alpha 5 (Pcdha5), mRNA | 2.23 | 0,0003 |
| A\_51\_P503297 | NM\_177882 | weakly similar to finger protein (clone XlcOF6.1) (fragment) [Xenopus laevis] | 2.23 | 0,0193 |
| A\_51\_P124951 | NM\_025296 | Mus musculus WD40 protein Ciao1 (Ciao1-pending), mRNA | 2.22 | 0,0000 |
| A\_51\_P141610 | BC052789 | similar to CDK3-BINDING PROTEIN IK3-1 [Mus musculus] | 2.22 | 0,0003 |
| A\_51\_P164892 | TC1434225 | Mus musculus cDNA, 5 end | 2.22 | 0,0060 |
| A\_51\_P237383 | NM\_021472 | Mus musculus ribonuclease, RNase A family 4 (Rnase4), mRNA | 2.22 | 0,0007 |
| A\_51\_P480178 | NM\_133191 | Mus musculus expressed sequence AI042819 (AI042819), mRNA | 2.22 | 0,0119 |
| A\_51\_P340653 | NM\_146136 | MONOCARBOXYLATE TRANSPORTER 5 MCT 5 MCT | 2.22 | 0,0201 |
| A\_51\_P390334 | 8430408O14 | unknown EST | 2.22 | 0,0004 |
| A\_51\_P118188 | NM\_207222 | TRANSCRIPTION FACTOR XLMO1 homolog [Xenopus laevis] | 2.22 | 0,0005 |
| A\_51\_P490747 | AK031845 | Mus musculus cDNA, 3 end | 2.22 | 0,0147 |
| A\_51\_P312885 | NM\_027898 | Mus musculus RIKEN cDNA 1300003M23 gene (1300003M23Rik), mRNA | 2.22 | 0,0029 |
| A\_51\_P218895 | NM\_008064 | Mus musculus glucosidase, alpha, acid (Gaa), mRNA | 2.22 | 0,0041 |
| A\_51\_P154596 | NM\_008721 | Mus musculus neural proliferation, differentiation and control gene 1 (Npdc1), mRNA | 2.22 | 0,0003 |
| A\_51\_P327559 | NM\_009752 | Mus musculus galactosidase, beta 1 (Glb1), mRNA | 2.22 | 0,0018 |
| A\_51\_P116039 | NM\_008256 | 3-hydroxy-3-methylglutaryl-Coenzyme A synthase 2 | 2.22 | 0,0286 |
| A\_51\_P125338 | NM\_172263 | similar to HIGH-AFFINITY CAMP-SPECIFIC AND IBMX-INSENSITIVE 3,5-CYCLIC PHOSPHODIESTERASE 8B (EC 3.1.4.17) (FRAGMENT) [Homo sapiens] | 2.22 | 0,0001 |
| A\_51\_P305352 | AB093272 | SYNAPTOJANIN 1 (EC 3.1.3.56) (SYNAPTIC INOSITOL-1,4,5-TRISPHOSPHATE 5- PHOSPHATASE 1) homolog [Rattus norvegicus] | 2.20 | 0,0063 |
| A\_51\_P247614 | NM\_183030 | unknown EST | 2.20 | 0,0002 |
| A\_51\_P402398 | NM\_178741 | hypothetical BTB/POZ domain containing protein | 2.20 | 0,0009 |
| A\_51\_P303258 | NM\_013540 | Mus musculus glutamate receptor, ionotropic, AMPA2 (alpha 2) (Gria2), mRNA | 2.20 | 0,0184 |
| A\_51\_P275827 | NM\_144887 | hypothetical DHHC-type Zn-finger containing protein | 2.20 | 0,0141 |
| A\_51\_P152532 | XM\_620516 | weakly similar to BM-013 [Homo sapiens] | 2.20 | 0,0080 |
| A\_51\_P502919 | NAP057073-1 | Mus musculus olfactory receptor GA\_x5J8B7W6KF8-5546505-5545716 (GA\_x5J8B7W6KF8-5546505-5545716) pseudogene | 2.20 | 0,0070 |
| A\_51\_P274124 | NM\_026345 | Mus musculus RIKEN cDNA 9130403P13 gene (9130403P13Rik), mRNA | 2.20 | 0,0011 |
| A\_51\_P311919 | NM\_008731 | Mus musculus neuropeptide Y receptor Y2 (Npy2r), mRNA | 2.20 | 0,0336 |
| A\_51\_P406527 | NM\_019697 | Mus musculus potassium voltage-gated channel, Shal-related family, member 2 (Kcnd2), mRNA | 2.20 | 0,0037 |
| A\_51\_P503933 | NM\_009790 | Mus musculus, clone IMAGE:2631567, mRNA | 2.20 | 0,0011 |
| A\_51\_P399545 | NM\_175251 | CDNA FLJ14812 FIS, CLONE NT2RP4002081, WEAKLY SIMILAR TO TRANSCRIPTION INITIATION FACTOR IIA ALPHA AND BETA CHAINS (FRAGMENT) homolog [Homo sapiens] | 2.20 | 0,0001 |
| A\_51\_P327232 | NM\_177041 | Mus musculus lens epithelial protein (Lenep), mRNA | 2.19 | 0,0005 |
| A\_51\_P283264 | AK079330 | similar to KRUPPEL-ASSOCIATED BOX PROTEIN [Homo sapiens] | 2.19 | 0,0001 |
| A\_51\_P456398 | NM\_020050 | Mus musculus RIKEN cDNA 2310004K06 gene (2310004K06Rik), mRNA | 2.19 | 0,0003 |
| A\_51\_P195439 | NM\_025991 | Mus musculus RIKEN cDNA 2510026C23 gene (2510026C23Rik), mRNA | 2.19 | 0,0014 |
| A\_51\_P490155 | NM\_020271 | Mus musculus hypothetical protein, MNCb-4193 (AB041662), mRNA | 2.19 | 0,0023 |
| A\_51\_P467770 | NM\_028874 | similar to SORTING NEXIN 19 [Homo sapiens] | 2.19 | 0,0017 |
| A\_51\_P223498 | NM\_172653 | weakly similar to CDNA FLJ32338 FIS, CLONE PROST2005919, MODERATELY SIMILAR TO HUMAN BREAST CANCER, ESTROGEN REGULATED LIV-1 PROTEIN (LIV-1) MRNA [Homo sapiens] | 2.19 | 0,0022 |
| A\_51\_P476538 | AK077391 | unknown EST | 2.19 | 0,0053 |
| A\_51\_P378858 | AK017355 | Mus musculus RIKEN cDNA 5430428K15 gene (5430428K15Rik), mRNA | 2.19 | 0,0002 |
| A\_51\_P222337 | NM\_172815 | hypothetical Thrombospondin type I repeat (TSP1) profile/Thrombospondin type I domain/Furin-like cysteine rich region containing protein | 2.19 | 0,0357 |
| A\_51\_P481238 | NM\_027293 | Mus musculus RIKEN cDNA 2610510B01 gene (2610510B01Rik), mRNA | 2.19 | 0,0013 |
| A\_51\_P501473 | AK083741 | unknown EST | 2.19 | 0,0007 |
| A\_51\_P165304 | AK079356 | unclassifiable | 2.19 | 0,0248 |
| A\_51\_P137322 | NM\_031161 | Mus musculus cholecystokinin (Cck), mRNA | 2.19 | 0,0173 |
| A\_51\_P116421 | NM\_013790 | Mus musculus ATP-binding cassette, sub-family C (CFTR/MRP), member 5a (Abcc5a), mRNA | 2.17 | 0,0002 |
| A\_51\_P196113 | NM\_019945 | Mus musculus syntrophin associated serine/threonine kinase (Sast-pending), mRNA | 2.17 | 0,0022 |
| A\_51\_P468876 | NM\_178118 | hypothetical DIX domain containing protein | 2.17 | 0,0004 |
| A\_51\_P273667 | NM\_011441 | Mus musculus SRY-box containing gene 17 (Sox17), mRNA | 2.17 | 0,0070 |
| A\_51\_P215368 | NM\_172271 | SODIUM AND CHLORIDE DEPENDENT TRANSPORTER | 2.17 | 0,0054 |
| A\_51\_P391634 | NM\_144865 | Mus musculus, clone MGC:28827 IMAGE:4505679, mRNA, complete cds | 2.17 | 0,0010 |
| A\_51\_P317542 | AK018192 | unknown EST | 2.17 | 0,0124 |
| A\_51\_P333159 | NM\_026653 | Mus musculus replication protein A1 (70 kDa) (Rpa1), mRNA | 2.17 | 0,0004 |
| A\_51\_P258522 | BC032214 | CDNA FLJ12883 FIS, CLONE NT2RP2003981, WEAKLY SIMILAR TO VACUOLAR PROTEIN SORTING-ASSOCIATED PROTEIN VPS8 homolog [Homo sapiens] | 2.17 | 0,0026 |
| A\_51\_P298325 | NM\_025820 | Mus musculus Crn, crooked neck-like 1 (Drosophila) (Crnkl1), mRNA | 2.17 | 0,0020 |
| A\_51\_P486683 | NM\_021492 | Mus musculus mRNA, partial cds, clone:2-73 | 2.17 | 0,0003 |
| A\_51\_P369862 | NM\_178772 | ARYLACETAMIDE DEACETYLASE EC 3.1.1.- | 2.17 | 0,0005 |
| A\_51\_P297896 | BC048187 | unknown EST | 2.16 | 0,0045 |
| A\_51\_P449055 | NM\_053234 | Mus musculus vomeronasal 1 receptor, C4 (V1rc4), mRNA | 2.16 | 0,0245 |
| A\_51\_P207570 | NM\_030064 | PHD-finger/EGF-like domain containing protein | 2.16 | 0,0013 |
| A\_51\_P425824 | NM\_026125 | Mus musculus RIKEN cDNA 1110035L05 gene (1110035L05Rik), mRNA | 2.16 | 0,0227 |
| A\_51\_P343016 | NM\_145830 | G9A homolog [Mus musculus] | 2.16 | 0,0005 |
| A\_51\_P166695 | NM\_008385 | Mus musculus inositol polyphosphate-5-phosphatase, 75 kDa (Inpp5b), mRNA | 2.16 | 0,0084 |
| A\_51\_P513785 | NM\_007794 | Mus musculus CCCTC-binding factor (Ctcf), mRNA | 2.16 | 0,0003 |
| A\_51\_P328300 | NM\_009787 | Mus musculus calcium binding protein, intestinal (Cai), mRNA | 2.16 | 0,0011 |
| A\_51\_P426566 | NM\_134067 | Mus musculus expressed sequence AW209491 (AW209491), mRNA | 2.16 | 0,0047 |
| A\_51\_P176783 | AK014824 | unknown EST | 2.16 | 0,0001 |
| A\_51\_P163694 | NM\_172557 | FYVE-FINGER CONTAINING PROTEIN (FRAGMENT) homolog [Mus musculus] | 2.16 | 0,0009 |
| A\_51\_P309494 | NM\_138602 | Mus musculus DNA segment, Chr X, Immunex 39, expressed (DXImx39e), mRNA | 2.16 | 0,0002 |
| A\_51\_P368755 | NM\_008069 | Mus musculus gamma-aminobutyric acid (GABA-A) receptor, subunit beta 1 (Gabrb1), mRNA | 2.16 | 0,0012 |
| A\_51\_P367741 | XM\_112129 | hypothetical protein | 2.16 | 0,0166 |
| A\_51\_P260548 | NM\_009149 | Mus musculus selectin, endothelial cell, ligand (Selel), mRNA | 2.16 | 0,0010 |
| A\_51\_P430388 | NM\_025362 | Mus musculus RIKEN cDNA 1300007M11 gene (1300007M11Rik), mRNA | 2.14 | 0,0002 |
| A\_51\_P131744 | AK045478 | unknown EST | 2.14 | 0,0076 |
| A\_51\_P224362 | AK129115 | unknown EST | 2.14 | 0,0059 |
| A\_51\_P214360 | NM\_133251 | Mus musculus vestigial-related factor (LOC170828), mRNA | 2.14 | 0,0071 |
| A\_51\_P343739 | NM\_146128 | Mus musculus, clone MGC:37636 IMAGE:5002845, mRNA, complete cds | 2.14 | 0,0080 |
| A\_51\_P311075 | A\_51\_P311075 | glycerol-3-phosphate acyltransferase, mitochondrial | 2.14 | 0,0491 |
| A\_51\_P309854 | NM\_080465 | Mus musculus potassium intermediate/small conductance calcium-activated channel, subfamily N, member 2 (Kcnn2), mRNA | 2.14 | 0,0132 |
| A\_51\_P361478 | NM\_011116 | Mus musculus phospholipase D3 (Pld3), mRNA | 2.14 | 0,0309 |
| A\_51\_P508770 | NM\_011846 | Mus musculus matrix metalloproteinase 17 (Mmp17), mRNA | 2.14 | 0,0103 |
| A\_51\_P300602 | AV124335 | ATP synthase, H+ transporting, mitochondrial F0 complex, subunit c (subunit 9), isoform 1 | 2.14 | 0,0002 |
| A\_51\_P336384 | AK032843 | SALIVARY PROLINE RICH PROTEIN [CONTAINS: PEPTIDE P | 2.14 | 0,0019 |
| A\_51\_P469252 | NM\_172817 | ZINC FINGER PROTEIN CLONE 647 (FRAGMENT) homolog [Homo sapiens] | 2.14 | 0,0005 |
| A\_51\_P140237 | NM\_010212 | Mus musculus four and a half LIM domains 2 (Fhl2), mRNA | 2.14 | 0,0163 |
| A\_51\_P195258 | NM\_019426 | Mus musculus activating transcription factor 7 interacting protein (Atf7ip), mRNA | 2.14 | 0,0029 |
| A\_51\_P118103 | BC085163 | Mus musculus cDNA, 5 end | 2.14 | 0,0002 |
| A\_51\_P184849 | NM\_008825 | 6-phosphofructo-2-kinase fructose -2,6-biphosphatase 2, full insert sequence. | 2.14 | 0,0127 |
| A\_51\_P446003 | NM\_013764 | Mus musculus deoxyguanosine kinase (Dguok), mRNA | 2.14 | 0,0017 |
| A\_51\_P132044 | NM\_013597 | Mus musculus myocyte enhancer factor 2A (Mef2a), mRNA | 2.14 | 0,0006 |
| A\_51\_P467539 | NM\_175191 | PROBABLE G PROTEIN-COUPLED RECEPTOR GPR22 homolog [Homo sapiens] | 2.13 | 0,0491 |
| A\_51\_P143468 | NM\_178771 | CDNA FLJ11078 FIS, CLONE PLACE1005102, WEAKLY SIMILAR TO RING CANAL PROTEIN homolog [Homo sapiens] | 2.13 | 0,0127 |
| A\_51\_P268496 | NM\_053195 | Mus musculus, Similar to solute carrier family 24 (sodium/potassium/calcium exchanger), member 3, clone MGC:27686 IMAGE:4914291, mRNA, complete cds | 2.13 | 0,0381 |
| A\_51\_P246895 | BC019787 | hypothetical Ribosome recycling factor containing protein | 2.13 | 0,0066 |
| A\_51\_P208811 | AK030894 | weakly similar to TESMIN (METALLOTHIONEIN-LIKE 5, TESTIS-SPECIFIC) (TESTIS-SPECIFIC METALLOTHIONEIN-LIKE PROTEIN) [Homo sapiens] | 2.13 | 0,0006 |
| A\_51\_P394735 | NM\_133962 | Mus musculus expressed sequence AI467246 (AI467246), mRNA | 2.13 | 0,0002 |
| A\_51\_P293017 | AK045075 | MYO INOSITOL MONOPHOSPHATASE | 2.13 | 0,0004 |
| A\_51\_P261359 | NM\_172692 | BILE ACID BETA-GLUCOSIDASE (KIAA1605 PROTEIN) homolog [Homo sapiens] | 2.13 | 0,0001 |
| A\_51\_P439842 | NM\_054077 | Mus musculus proline arginine-rich end leucine-rich repeat (Prelp), mRNA | 2.13 | 0,0092 |
| A\_51\_P117236 | NM\_009329 | Mus musculus zinc finger protein 354A (Zfp354a), mRNA | 2.11 | 0,0016 |
| A\_51\_P479043 | NM\_022988 | Mus musculus Ngg1 interacting factor 3-like 1 (S. pombe) (Nif3l1), mRNA | 2.11 | 0,0041 |
| A\_51\_P374572 | NM\_008344 | Mus musculus insulin-like growth factor binding protein 6 (Igfbp6), mRNA | 2.11 | 0,0341 |
| A\_51\_P501963 | NM\_021463 | Mus musculus phosphoribosyl pyrophosphate synthetase 1 (Prps1), mRNA | 2.11 | 0,0003 |
| A\_51\_P225056 | AK047799 | hypothetical protein | 2.11 | 0,0002 |
| A\_51\_P367081 | NM\_011074 | Mus musculus PFTAIRE protein kinase 1 (Pftk1), mRNA | 2.11 | 0,0082 |
| A\_51\_P269073 | NM\_019986 | Mus musculus hyaluronic acid binding protein 4 (Habp4), mRNA | 2.11 | 0,0017 |
| A\_51\_P459679 | NM\_007978 | Mus musculus factor 8-associated gene A (F8a), mRNA | 2.11 | 0,0103 |
| A\_51\_P333279 | NM\_008227 | Mus musculus hyperpolarization-activated, cyclic nucleotide-gated K+ 3 (Hcn3), mRNA | 2.11 | 0,0051 |
| A\_51\_P126337 | NM\_183064 | fibroblast growth factor 12 | 2.11 | 0,0009 |
| A\_51\_P399924 | NM\_001005506 | inferred: dJ622L5.2 (novel protein) {Homo sapiens} | 2.11 | 0,0006 |
| A\_51\_P398235 | AK053156 | hypothetical DnaJ N-terminal domain containing protein | 2.11 | 0,0028 |
| A\_51\_P471940 | XM\_357108 | MITOCHONDRIAL 39S RIBOSOMAL PROTEIN L45 (MRP-L45) homolog [Homo sapiens] | 2.11 | 0,0003 |
| A\_51\_P143951 | NM\_007495 | Mus musculus astrotactin 1 (Astn1), mRNA | 2.10 | 0,0017 |
| A\_51\_P501757 | BC096024 | hypothetical protein | 2.10 | 0,0026 |
| A\_51\_P144319 | NM\_207654 | hypothetical protein | 2.10 | 0,0104 |
| A\_51\_P510485 | BC046305 | Mus musculus, clone IMAGE:4191452, mRNA, partial cds | 2.10 | 0,0376 |
| A\_51\_P293077 | NM\_008583 | Mus musculus multiple endocrine neoplasia 1 (Men1), mRNA | 2.10 | 0,0002 |
| A\_51\_P298107 | NM\_028813 | VITRIN homolog [Mus musculus] | 2.10 | 0,0269 |
| A\_51\_P299934 | NM\_173022 | Mus musculus, clone IMAGE:1246046, mRNA, partial cds | 2.10 | 0,0003 |
| A\_51\_P481788 | AK018549 | Mus musculus cDNA clone IMAGE:6490017 5 | 2.10 | 0,0213 |
| A\_51\_P114238 | BC029786 | Mus musculus cDNA, 5 end | 2.10 | 0,0008 |
| A\_51\_P131962 | NM\_198247 | dJ667H12.2.1 (novel protein (isoform 1)) {Homo sapiens} | 2.10 | 0,0320 |
| A\_51\_P182443 | AK173016 | TRANSLATION INITIATION FACTOR IF-2 homolog [Homo sapiens] | 2.10 | 0,0002 |
| A\_51\_P376658 | AK004090 | hypothetical protein | 2.10 | 0,0005 |
| A\_51\_P167452 | AK049330 | hypothetical Target SNARE coiled-coil domain containing protein | 2.10 | 0,0065 |
| A\_51\_P266403 | AK009163 | unknown EST | 2.08 | 0,0495 |
| A\_51\_P484653 | NM\_022022 | Mus musculus ubiquitination factor E4B, UFD2 homolog (S. cerevisiae) (Ube4b), mRNA | 2.08 | 0,0012 |
| A\_51\_P112662 | NM\_001018042 | Mus musculus transcription factor Sp3 mRNA, partial cds | 2.08 | 0,0003 |
| A\_51\_P351263 | NM\_001011775 | Mus musculus olfactory receptor MOR266-10 (MOR266-10) pseudogene | 2.08 | 0,0001 |
| A\_51\_P408460 | AK086518 | unknown EST | 2.08 | 0,0178 |
| A\_51\_P129724 | NM\_175244 | hypothetical HECT domain (Ubiquitin-protein ligase) containing protein | 2.08 | 0,0006 |
| A\_51\_P149932 | A\_51\_P149932 | Mus musculus cDNA, 5 end | 2.08 | 0,0234 |
| A\_51\_P419116 | NM\_134050 | Mus musculus RIKEN cDNA 2310012G06 gene (2310012G06Rik), mRNA | 2.08 | 0,0256 |
| A\_51\_P468408 | NM\_027923 | hypothetical protein | 2.08 | 0,0033 |
| A\_51\_P219055 | NM\_026936 | similar to CYTOCHROME OXIDASE BIOGENESIS PROTEIN OXA1, MITOCHONDRIAL PRECURSOR (OXA1-LIKE PROTEIN) (OXA1HS) [Homo sapiens] | 2.08 | 0,0005 |
| A\_51\_P253732 | NM\_027265 | hypothetical protein | 2.08 | 0,0077 |
| A\_51\_P433837 | AK013991 | unknown EST | 2.08 | 0,0017 |
| A\_51\_P112500 | A\_51\_P112500 | Mus musculus cDNA, 3 end | 2.08 | 0,0030 |
| A\_51\_P496845 | NM\_007604 | Mus musculus capping protein alpha 2 (Cappa2), mRNA | 2.08 | 0,0063 |
| A\_51\_P423709 | NM\_029007 | Mus musculus, RIKEN cDNA 4731402F03 gene, clone MGC:7324 IMAGE:3486254, mRNA, complete cds | 2.08 | 0,0416 |
| A\_51\_P278136 | NM\_028215 | hypothetical protein | 2.08 | 0,0001 |
| A\_51\_P477941 | NM\_013907 | Mus musculus f-box and WD-40 domain protein 4 (Fbxw4), mRNA | 2.07 | 0,0006 |
| A\_51\_P440099 | NM\_175240 | hypothetical Immunoglobulin structure containing protein | 2.07 | 0,0423 |
| A\_51\_P452637 | XM\_355470 | Similar to pyruvate dehydrogenase phosphatase, clone IMAGE:6492665, mRNA | 2.07 | 0,0420 |
| A\_51\_P246133 | NM\_010140 | Mouse eph-related receptor tyrosine kinase (Mek4) mRNA, complete cds | 2.07 | 0,0143 |
| A\_51\_P506748 | NM\_172739 | hypothetical protein | 2.07 | 0,0015 |
| A\_51\_P293665 | AK003646 | weakly similar to LDOC1 PROTEIN (LEUCINE ZIPPER PROTEIN DOWN-REGULATED IN CANCER CELLS) [Homo sapiens] | 2.07 | 0,0004 |
| A\_51\_P100099 | AK011292 | unknown EST | 2.07 | 0,0038 |
| A\_51\_P411645 | NM\_021500 | Mus musculus macrophage erythroblast attacher (Maea), mRNA | 2.07 | 0,0446 |
| A\_51\_P227703 | XM\_485005 | hypothetical Cysteine-rich region containing protein | 2.07 | 0,0036 |
| A\_51\_P316311 | AK173053 | protein tyrosine phosphatase, receptor type, f polypeptide (PTPRF), interacting protein (liprin), alpha 4 | 2.07 | 0,0211 |
| A\_51\_P388298 | NM\_025962 | Mus musculus RIKEN cDNA 1810037K07 gene (1810037K07Rik), mRNA | 2.07 | 0,0009 |
| A\_51\_P370163 | NM\_009431 | Mus musculus TPR-containing, SH2-binding phosphoprotein (Tsbp), mRNA | 2.07 | 0,0018 |
| A\_51\_P376149 | NM\_026526 | Mus musculus RIKEN cDNA 2510005D08 gene (2510005D08Rik), mRNA | 2.06 | 0,0029 |
| A\_51\_P204801 | NM\_009920 | Mus musculus cornichon-like (Drosophila) (Cnil), mRNA | 2.06 | 0,0181 |
| A\_51\_P388984 | NM\_026446 | Mus musculus regulator of G-protein signaling 19 (Rgs19), mRNA | 2.06 | 0,0001 |
| A\_51\_P379953 | BC038619 | unknown EST | 2.06 | 0,0101 |
| A\_51\_P321531 | AF479672 | "Mus musculus Pur-gamma B-form (Purg) mRNA, complete cds� alternatively spliced" | 2.06 | 0,0006 |
| A\_51\_P497350 | NM\_011464 | Mus musculus hepatocyte growth factor activator inhibitor type 2 (Hai2) mRNA, complete cds | 2.06 | 0,0111 |
| A\_51\_P307557 | XM\_131434 | similar to BRAIN PROTEIN (FRAGMENT) [Homo sapiens] | 2.06 | 0,0012 |
| A\_51\_P219532 | AK003388 | hypothetical protein | 2.06 | 0,0038 |
| A\_51\_P486068 | NM\_177475 | DNA segment, Chr 10, Johns Hopkins University 82, expressed | 2.06 | 0,0025 |
| A\_51\_P244969 | NM\_025346 | Mus musculus, clone MGC:27569 IMAGE:4485143, mRNA, complete cds | 2.06 | 0,0008 |
| A\_51\_P499369 | NM\_145599 | SIMILAR TO HYPOTHETICAL PROTEIN FLJ10846 | 2.06 | 0,0014 |
| A\_51\_P260721 | NM\_016902 | Mus musculus nephronophthisis 1 (juvenile) homolog (human) (Nphp1), mRNA | 2.06 | 0,0022 |
| A\_51\_P492797 | NM\_177736 | Mus musculus, Similar to hypothetical protein MGC3036, clone MGC:28139 IMAGE:3981816, mRNA, complete cds | 2.06 | 0,0057 |
| A\_51\_P461364 | NM\_024198 | Mus musculus RIKEN cDNA 3110050F08 gene (3110050F08Rik), mRNA | 2.06 | 0,0146 |
| A\_51\_P421882 | NM\_029580 | Mus musculus, RIKEN cDNA 2610027O18 gene, clone MGC:6806 IMAGE:2648293, mRNA, complete cds | 2.04 | 0,0016 |
| A\_51\_P468398 | NM\_175300 | ANAPHASE-PROMOTING COMPLEX SUBUNIT 2 homolog [Homo sapiens] | 2.04 | 0,0004 |
| A\_51\_P220422 | NM\_011861 | Mus musculus protein kinase C and casein kinase substrate in neurons 1 (Pacsin1), mRNA | 2.04 | 0,0062 |
| A\_51\_P171965 | NM\_026503 | Mus musculus RIKEN cDNA 1110058L19 gene (1110058L19Rik), mRNA | 2.04 | 0,0011 |
| A\_51\_P489069 | NM\_146257 | Similar to: Mus musculus solute carrier family 29 (nucleoside transporters), member 4 (Slc29a4), mRNA | 2.04 | 0,0020 |
| A\_51\_P274073 | NM\_175545 | hypothetical protein | 2.04 | 0,0042 |
| A\_51\_P398887 | NM\_031251 | cystinosis, nephropathic | 2.04 | 0,0009 |
| A\_51\_P358316 | NM\_007693 | Mus musculus chromogranin A (Chga), mRNA | 2.04 | 0,0006 |
| A\_51\_P103745 | NM\_010075 | Mus musculus strain C57Bl/6 dipeptidyl aminopeptidase-like protein 6 embryonic isoform (Dpp6) mRNA, complete cds | 2.04 | 0,0051 |
| A\_51\_P485740 | NM\_026086 | Mus musculus RIKEN cDNA 1600031M04 gene (1600031M04Rik), mRNA | 2.04 | 0,0022 |
| A\_51\_P428742 | NM\_172437 | similar to HYPOTHETICAL 80.7 KDA PROTEIN [Homo sapiens] | 2.04 | 0,0026 |
| A\_51\_P326419 | NM\_029802 | Mus musculus, RIKEN cDNA 2310002N04 gene, clone MGC:7909 IMAGE:3583149, mRNA, complete cds | 2.04 | 0,0147 |
| A\_51\_P485220 | NM\_173185 | unknown EST | 2.04 | 0,0013 |
| A\_51\_P262101 | NM\_026873 | hypothetical Prokaryotic membrane lipoprotein lipid attachment site/PPR repeats containing protein | 2.04 | 0,0048 |
| A\_51\_P104985 | AI391295 | Mus musculus cDNA, 3 end | 2.03 | 0,0169 |
| A\_51\_P326191 | AY862185 | Mouse spi2 proteinase inhibitor (spi2/eb1) mRNA, 3 end | 2.03 | 0,0186 |
| A\_51\_P169886 | NM\_020276 | Mus musculus nasal embryonic LHRH factor (Nelf), mRNA | 2.03 | 0,0192 |
| A\_51\_P483168 | AW536275 | Mus musculus cDNA, 3 end | 2.03 | 0,0068 |
| A\_51\_P227785 | AK033046 | unclassifiable | 2.03 | 0,0206 |
| A\_51\_P111049 | NM\_178711 | PHOSPHOLIPID SCRAMBLASE 4 (PL SCRAMBLASE 4) (CA2+ DEPENDENT PHOSPHOLIPID SCRAMBLASE 4) (FRAGMENT) homolog [Mus musculus] | 2.03 | 0,0077 |
| A\_51\_P434497 | NM\_026981 | Mus musculus, RIKEN cDNA 1810033A06 gene, clone MGC:28242 IMAGE:3993232, mRNA, complete cds | 2.03 | 0,0051 |
| A\_51\_P386964 | XM\_485063 | hypothetical protein | 2.03 | 0,0391 |
| A\_51\_P344741 | BC086456 | hypothetical Hemopexin domain/Serine/threonine specific protein phosphatase containing protein | 2.03 | 0,0007 |
| A\_51\_P337974 | NM\_133363 | Mus musculus calsarcin-3 (LOC170947), mRNA | 2.03 | 0,0019 |
| A\_51\_P267447 | NM\_009616 | Mus musculus a disintegrin and metalloproteinase domain 19 (meltrin beta) (Adam19), mRNA | 2.03 | 0,0145 |
| A\_51\_P354526 | NM\_025912 | Mus musculus, RIKEN cDNA 2010011I20 gene, clone MGC:27703 IMAGE:4924329, mRNA, complete cds | 2.01 | 0,0219 |
| A\_51\_P463440 | NM\_130450 | Mus musculus long chain fatty acyl elongase (Lce-pending), mRNA | 2.01 | 0,0001 |
| A\_51\_P416046 | BC020156 | Mus musculus, Similar to hypothetical gene MGC1127, clone MGC:28380 IMAGE:4021570, mRNA, complete cds | 2.01 | 0,0003 |
| A\_51\_P202801 | NM\_019875 | Mus musculus ATP-binding cassette, sub-family B (MDR/TAP), member 9 (Abcb9), mRNA | 2.01 | 0,0008 |
| A\_51\_P357203 | XM\_144310 | Mus musculus, clone IMAGE:4024509, mRNA | 2.01 | 0,0017 |
| A\_51\_P386025 | AK009987 | unknown EST | 2.01 | 0,0007 |
| A\_51\_P169560 | XM\_126361 | PROTEIN NJMU-R1 homolog [Mus musculus] | 2.01 | 0,0005 |
| A\_51\_P125866 | XM\_207079 | Mus musculus spectrin alpha 2, mRNA (cDNA clone IMAGE:5353935), partial cds | 2.01 | 0,0031 |
| A\_51\_P425402 | NM\_021286 | Mus musculus seizure related gene 6 (Sez6), mRNA | 2.01 | 0,0142 |
| A\_51\_P311137 | NM\_011360 | Mus musculus sarcoglycan, epsilon (Sgce), mRNA | 2.01 | 0,0004 |
| A\_51\_P118671 | NM\_033566 | Mus musculus SWI/SNF related, matrix associated, actin dependent regulator of chromatin, subfamily f, member 1 (Smarcf1), mRNA | 2.01 | 0,0006 |
| A\_51\_P107228 | NM\_013854 | Similar to ATP-binding cassette, sub-family F (GCN20), member 1, clone IMAGE:6485365, mRNA | 2.01 | 0,0121 |
| A\_51\_P334876 | NM\_007384 | Mus musculus amiloride-sensitive cation channel 1, neuronal (degenerin) (Accn1), mRNA | 2.01 | 0,0049 |
| A\_51\_P397493 | NM\_017401 | Mus musculus polymerase (DNA directed), mu (Polm), mRNA | 2.01 | 0,0032 |
| A\_51\_P169061 | NM\_173014 | hypothetical EF-hand/Phospholipid and glycerol acyltransferase (from motifs\_6.msf) containing protein | 2.01 | 0,0106 |
| A\_51\_P103650 | NM\_053100 | Mus musculus tripartite motif protein 8 (Trim8), mRNA | 2.01 | 0,0020 |
| A\_51\_P412914 | NM\_010112 | Mus musculus embryonal Fyn-associated substrate (Efs), mRNA | 2.01 | 0,0296 |
| A\_51\_P328870 | D86599 | Mus sp. mRNA for oxytocin receptor, complete cds | 2.00 | 0,0255 |
| A\_51\_P421244 | NM\_026662 | Mus musculus RIKEN cDNA 2610101M19 gene (2610101M19Rik), mRNA | 2.00 | 0,0015 |
| A\_51\_P396375 | NM\_011586 | Mus musculus MysPDZ mRNA for myosin containing PDZ domain, complete cds | 2.00 | 0,0024 |
| A\_51\_P278893 | NM\_010412 | Mus musculus histone deacetylase 5 (Hdac5), mRNA | 2.00 | 0,0069 |
| A\_51\_P255565 | NM\_007958 | M.musculus mRNA of enhancer-trap-locus 1 | 2.00 | 0,0015 |
| A\_51\_P196889 | XM\_620751 | IRP2, IRON-RESPONSIVE ELEMENT-BINDING PROTEIN/IRON REGULATORY PROTEIN 2 (FRAGMENT) homolog [Homo sapiens] | 2.00 | 0,0019 |
| A\_51\_P224575 | NM\_010322 | Mus musculus glyceronephosphate O-acyltransferase (Gnpat), mRNA | 2.00 | 0,0005 |
| A\_51\_P443976 | NM\_011217 | Mus musculus protein tyrosine phosphatase, receptor type, R (Ptprr), mRNA | 2.00 | 0,0234 |
| A\_51\_P434269 | NM\_026610 | similar to NADH DEHYDROGENASE (UBIQUINONE) 1 BETA SUBCOMPLEX, 4 (15KD, B15) [Homo sapiens] | 2.00 | 0,0057 |
| A\_51\_P389714 | BC030917 | Mus musculus, clone IMAGE:3596085, mRNA | 2.00 | 0,0038 |
| A\_51\_P502107 | AK034345 | unknown EST | 2.00 | 0,0002 |
| A\_51\_P342829 | NM\_027909 | hypothetical C2 domain (Calcium/lipid-binding domain, CaLB) structure containing protein | 2.00 | 0,0106 |
| A\_51\_P146970 | NM\_145831 | similar to DMRT2/TERRA-LIKE PROTEIN [Homo sapiens] | 2.00 | 0,0120 |
| A\_51\_P317788 | NM\_176843 | unknown EST | 2.00 | 0,0002 |
| A\_51\_P199187 | NM\_026062 | weakly similar to PANCREATITIS-INDUCED PROTEIN 49 [Mus musculus] | 2.00 | 0,0009 |
| A\_51\_P113195 | NM\_178924 | TGFbeta-regulated protein TI-1 homolog [Mustela vison] | 2.00 | 0,0083 |
| A\_51\_P133763 | NM\_175642 | DJ91B17.1 (BRAIN-SPECIFIC ANGIOGENESIS INHIBITOR 3) (FRAGMENT) homolog [Homo sapiens] | 2.00 | 0,0434 |
| A\_51\_P377506 | NM\_172946 | KERATIN, TYPE I | 2.00 | 0,0054 |
| A\_51\_P372839 | NM\_172599 | hypothetical protein | 1.99 | 0,0101 |
| A\_51\_P366152 | NM\_177591 | INHIBIN BINDING PROTEIN LONG ISOFORM homolog [Rattus norvegicus] | 1.99 | 0,0077 |
| A\_51\_P426555 | NM\_133991 | Mus musculus expressed sequence AI931847 (AI931847), mRNA | 1.99 | 0,0002 |
| A\_51\_P350706 | NM\_178613 | HSPC210 homolog [Homo sapiens] | 1.99 | 0,0089 |
| A\_51\_P190000 | NM\_027134 | Mus musculus, RIKEN cDNA 2310020P08 gene, clone MGC:28710 IMAGE:4456981, mRNA, complete cds | 1.99 | 0,0057 |
| A\_51\_P275101 | AK003880 | hypothetical Histidine-rich region containing protein | 1.99 | 0,0170 |
| A\_51\_P154513 | NM\_145837 | musculus interleukin 17D (IL-17D), mRNA | 1.99 | 0,0049 |
| A\_51\_P422685 | NM\_177086 | THYRO1000793 PROTEIN homolog [Homo sapiens] | 1.99 | 0,0466 |
| A\_51\_P267986 | NM\_145951 | TUMOR-ASSOCIATED HYDROQUINONE (NADH) OXIDASE TNOX homolog [Homo sapiens] | 1.99 | 0,0209 |
| A\_51\_P223946 | AK122414 | 49J10.1.2 (ANDROGEN-INDUCED PROSTATE PROLIFERATIVE SHUTOFF ASSOCIATED PROTEIN, ISOFORM 2) (FRAGMENT) homolog [Homo sapiens] | 1.99 | 0,0142 |
| A\_51\_P454002 | NM\_027129 | RIKEN cDNA 0610038L13 gene | 1.99 | 0,0029 |
| A\_51\_P207153 | NM\_198001 | Mus musculus, Similar to chromosome 9 open reading frame 16, clone MGC:19388 IMAGE:2812475, mRNA, complete cds | 1.99 | 0,0168 |
| A\_51\_P214516 | NM\_008698 | Mus musculus 4-nitrophenylphosphatase domain and non-neuronal SNAP25-like protein homolog 1 (C. elegans) (Nipsnap1), mRNA | 1.99 | 0,0030 |
| A\_51\_P474709 | AK008216 | unknown EST | 1.99 | 0,0020 |
| A\_51\_P436957 | NM\_025799 | Mus musculus RIKEN cDNA 0610025O11 gene (0610025O11Rik), mRNA | 1.99 | 0,0006 |
| A\_51\_P361286 | NM\_026792 | 1-ACYL-SN-GLYCEROL-3-PHOSPHATE ACYLTRANSFERASE EPSILON (EC 2.3.1.51) (1-AGP ACYLTRANSFERASE 5) (1-AGPAT 5) (LYSOPHOSPHATIDIC ACID ACYLTRANSFERASE-EPSILON) (LPAAT-EPSILON) (1-ACYLGLYCEROL-3-PHOSPHATE O-ACYLTRANSFERASE 5) | 1.99 | 0,0117 |
| A\_51\_P323281 | NM\_152825 | DJ199J3 NOVEL PROTEIN SIMILAR TO UBIQUITIN CARBOXYL TERMINAL HYDROLASE 16 EC 3.1.2.15 | 1.99 | 0,0059 |
| A\_51\_P138348 | NM\_007454 | Mus musculus adaptor protein complex AP-1, beta 1 subunit (Ap1b1), mRNA | 1.99 | 0,0005 |
| A\_51\_P492446 | NM\_172401 | hypothetical Esterase/lipase/thioesterase family active site containing protein | 1.99 | 0,0037 |
| A\_51\_P245275 | NM\_010436 | Mus musculus H2A histone family, member X (H2afx), mRNA | 1.99 | 0,0377 |
| A\_51\_P277275 | NM\_009065 | Mus musculus RAS-like protein expressed in neuron (Rin), mRNA | 1.99 | 0,0001 |
| A\_51\_P109452 | NM\_008519 | Mus musculus leukotriene B4 receptor 1 (Ltb4r1), mRNA | 1.99 | 0,0472 |
| A\_51\_P378978 | NM\_013496 | Mus musculus cellular retinoic acid binding protein I (Crabp1), mRNA | 1.99 | 0,0072 |
| A\_51\_P281586 | NM\_033569 | Mus musculus cyclin M2 (Cnnm2), mRNA | 1.99 | 0,0014 |
| A\_51\_P128096 | NM\_016878 | Mus musculus aspartyl aminopeptidase (Dnpep), mRNA | 1.99 | 0,0020 |
| A\_51\_P478952 | NM\_133898 | Mus musculus expressed sequence AI428195 (AI428195), mRNA | 1.99 | 0,0024 |
| A\_51\_P363791 | NM\_018829 | Mus musculus adaptor-related protein complex AP-3, mu 1 subunit (Ap3m1), mRNA | 1.99 | 0,0088 |
| A\_51\_P425165 | A\_51\_P425165 | Mus musculus cDNA, 5 end | 1.97 | 0,0006 |
| A\_51\_P413710 | NM\_007460 | Mus musculus adaptor-related protein complex AP-3, delta subunit (Ap3d), mRNA | 1.97 | 0,0009 |
| A\_51\_P152489 | NM\_026388 | hypothetical protein | 1.97 | 0,0004 |
| A\_51\_P256617 | AK047064 | splicing factor, arginine/serine rich 9 (25 kDa) | 1.97 | 0,0019 |
| A\_51\_P241262 | NM\_153820 | similar to UNCHARACTERIZED BONE MARROW PROTEIN BM046 [Homo sapiens] | 1.97 | 0,0356 |
| A\_51\_P322041 | NM\_177678 | hypothetical LIM domain, Villin headpiece domain containing protein | 1.97 | 0,0430 |
| A\_51\_P170405 | NM\_008356 | Mus musculus interleukin 13 receptor, alpha 2 (Il13ra2), mRNA | 1.97 | 0,0164 |
| A\_51\_P204845 | AK196672 | CUG triplet repeat, RNA binding protein 1 | 1.97 | 0,0293 |
| A\_51\_P174221 | BC057001 | Mus musculus cDNA, 5 end | 1.97 | 0,0010 |
| A\_51\_P483922 | NM\_133847 | Mus musculus expressed sequence AU045326 (AU045326), mRNA | 1.97 | 0,0005 |
| A\_51\_P375406 | NM\_144861 | unknown EST | 1.97 | 0,0082 |
| A\_51\_P204329 | AK049846 | similar to CDNA FLJ30794 FIS, CLONE FEBRA2001093, WEAKLY SIMILAR TO MONOCARBOXYLATE TRANSPORTER 4 [Homo sapiens] | 1.97 | 0,0049 |
| A\_51\_P323878 | NM\_030205 | 70 KDA WD-REPEAT TUMOR REJECTION ANTIGEN HOMOLOG homolog [Mus musculus] | 1.97 | 0,0008 |
| A\_51\_P489488 | NM\_031401 | Mus musculus upregulated during skeletal muscle growth 4 (Usmg4), mRNA | 1.97 | 0,0238 |
| A\_51\_P311476 | NM\_177740 | hypothetical protein | 1.97 | 0,0239 |
| A\_51\_P370700 | NM\_010324 | Mus musculus glutamate oxaloacetate transaminase 1, soluble (Got1), mRNA | 1.97 | 0,0012 |
| A\_51\_P394149 | AK009373 | cell division cycle 2-like 5 (cholinesterase-related cell division controller) | 1.97 | 0,0017 |
| A\_51\_P362089 | NM\_146052 | LEUCINE RICH REPEAT PROTEIN LRRC3 | 1.97 | 0,0028 |
| A\_51\_P371241 | AK173333 | unknown EST | 1.97 | 0,0029 |
| A\_51\_P195557 | NM\_026249 | Mus musculus RIKEN cDNA 4930429B21 gene (4930429B21Rik), mRNA | 1.97 | 0,0053 |
| A\_51\_P513611 | NM\_146078 | unknown EST | 1.97 | 0,0028 |
| A\_51\_P381377 | NM\_023279 | Mus musculus tubulin, beta 3 (Tubb3), mRNA | 1.97 | 0,0085 |
| A\_51\_P319065 | NM\_033604 | Mus musculus arkadia (Ark-pending), mRNA | 1.97 | 0,0002 |
| A\_51\_P363759 | NM\_019434 | Mus musculus minichromosome maintenance deficient (S. cerevisiae) 3-associated protein (Mcm3ap), mRNA | 1.97 | 0,0070 |
| A\_51\_P103594 | AK020485 | hypothetical protein | 1.96 | 0,0126 |
| A\_51\_P500200 | NM\_172971 | inositol polyphosphate-4-phosphatase, type I, 107kD | 1.96 | 0,0261 |
| A\_51\_P261184 | NM\_172403 | similar to ZINC FINGER PROTEIN (FRAGMENT) [Homo sapiens] | 1.96 | 0,0012 |
| A\_51\_P499802 | BC030380 | RIKEN cDNA C330021I08 gene | 1.96 | 0,0006 |
| A\_51\_P482365 | NM\_183087 | weakly similar to HYPOTHETICAL PROTEIN KIAA0574 (FRAGMENT) [Homo sapiens] | 1.96 | 0,0219 |
| A\_51\_P516526 | XM\_132038 | similar to SIMILAR TO STROMAL INTERACTION MOLECULE 2 (FRAGMENT) [Homo sapiens] | 1.96 | 0,0255 |
| A\_51\_P197866 | AK035905 | hypothetical protein | 1.96 | 0,0092 |
| A\_51\_P450469 | NM\_010932 | Mouse mRNA for nociceptin/orphanin FQ, complete cds | 1.96 | 0,0346 |
| A\_51\_P345714 | NM\_011647 | Mus musculus tuberous sclerosis 2 (Tsc2), mRNA | 1.96 | 0,0023 |
| A\_51\_P356962 | NM\_145219 | weakly similar to LEUCINE-RICH GLIOMA-INACTIVATED 1 PROTEIN [Mus musculus] | 1.96 | 0,0269 |
| A\_51\_P192491 | NM\_021793 | Mus musculus transmembrane protein 8 (five membrane-spanning domains) (Tmem8), mRNA | 1.96 | 0,0024 |
| A\_51\_P202418 | NM\_053187 | Mus musculus PDZ protein interacting specifically with TC10 (Pist-pending), mRNA | 1.96 | 0,0069 |
| A\_51\_P280851 | TC1448939 | ZINC FINGER PROTEIN 46 ZINC FINGER PROTEIN | 1.96 | 0,0280 |
| A\_51\_P361580 | NM\_008997 | Mus musculus RAB11B, member RAS oncogene family (Rab11b), mRNA | 1.96 | 0,0004 |
| A\_51\_P138923 | NM\_013788 | Mus musculus frequently rearranged in advanced T-cell lymphomas 3 (Frat3), mRNA | 1.96 | 0,0005 |
| A\_51\_P300297 | NM\_026075 | Mus musculus RIKEN cDNA 3110031B13 gene (3110031B13Rik), mRNA | 1.96 | 0,0057 |
| A\_51\_P352283 | BC043107 | weakly similar to 101F6 PROTEIN [Mus musculus] | 1.96 | 0,0080 |
| A\_51\_P302222 | NM\_026174 | Mus musculus lysosomal apyrase-like 1 (Lysal1), mRNA | 1.96 | 0,0019 |
| A\_51\_P107991 | NM\_009550 | Mus musculus zinc finger protein 2 (Zfp2), mRNA | 1.95 | 0,0024 |
| A\_51\_P283649 | NM\_172724 | hypothetical ARM repeat structure containing protein | 1.95 | 0,0002 |
| A\_51\_P312217 | BC089370 | unknown EST | 1.95 | 0,0285 |
| A\_51\_P279851 | NM\_201408 | Mus musculus RIKEN cDNA 1500041N16 gene (1500041N16Rik), mRNA | 1.95 | 0,0007 |
| A\_51\_P354572 | NM\_198163 | inferred: guanine nucleotide-binding protein ray {Homo sapiens} | 1.95 | 0,0004 |
| A\_51\_P319449 | NM\_144784 | ACETYL-COA ACETYLTRANSFERASE, MITOCHONDRIAL PRECURSOR (EC 2.3.1.9) (ACETOACETYL-COA THIOLASE) homolog [Rattus norvegicus] | 1.95 | 0,0016 |
| A\_51\_P140091 | NM\_146018 | hypothetical protein | 1.95 | 0,0001 |
| A\_51\_P405985 | NM\_133999 | Mus musculus expressed sequence AI326867 (AI326867), mRNA | 1.95 | 0,0003 |
| A\_51\_P431976 | AK036079 | unknown EST | 1.95 | 0,0312 |
| A\_51\_P469332 | AK034607 | unknown EST | 1.95 | 0,0012 |
| A\_51\_P406478 | TC1461965 | weakly similar to TUMOR ENDOTHELIAL MARKER 4 [Homo sapiens] | 1.95 | 0,0045 |
| A\_51\_P369510 | NM\_025839 | Mus musculus RIKEN cDNA 1110001K21 gene (1110001K21Rik), mRNA | 1.95 | 0,0047 |
| A\_51\_P301809 | XM\_203363 | Mus musculus SLIT3 (Slit3) mRNA, partial cds | 1.95 | 0,0320 |
| A\_51\_P514922 | BC021475 | Mus musculus, clone IMAGE:5343456, mRNA, partial cds | 1.95 | 0,0236 |
| A\_51\_P499979 | A\_51\_P499979 | Mus musculus cDNA, 5 end | 1.95 | 0,0204 |
| A\_51\_P144285 | BC058403 | ribosomal S6 protein kinase {Homo sapiens} | 1.95 | 0,0005 |
| A\_51\_P352264 | NM\_153056 | Mus musculus, Similar to sirtuin silent mating type information regulation 2 homolog 7 (S. cerevisiae), clone MGC:37560 IMAGE:4987746, mRNA, complete cds | 1.95 | 0,0042 |
| A\_51\_P377789 | NM\_009582 | Mus musculus mitogen activated protein kinase kinase kinase 12 (Map3k12), mRNA | 1.95 | 0,0031 |
| A\_51\_P168905 | NM\_015777 | Mus musculus immunoglobulin (CD79A) binding protein 1b (Igbp1b-pending), mRNA | 1.93 | 0,0043 |
| A\_51\_P477692 | NM\_178086 | similar to FATTY ACID HYDROXYLASE (UNKNOWN) (PROTEIN FOR MGC:4282) [Homo sapiens] | 1.93 | 0,0387 |
| A\_51\_P488953 | NM\_007767 | Mus musculus protocadherin alpha 9 mRNA, complete cds | 1.93 | 0,0312 |
| A\_51\_P464199 | NM\_178143 | inferred: 5-AMP-activated protein kinase catalytic alpha-2 subunit {Rattus norvegicus} | 1.93 | 0,0012 |
| A\_51\_P205740 | NM\_054093 | Mus musculus ubiquitin-protein ligase UBE3B (Ube3b) mRNA, partial cds | 1.93 | 0,0104 |
| A\_51\_P116755 | NM\_026056 | Mus musculus RIKEN cDNA 2810452G09 gene (2810452G09Rik), mRNA | 1.93 | 0,0287 |
| A\_51\_P494589 | NM\_021494 | Mus musculus partial mRNA for hypothetical protein (ORF37 DNA) | 1.93 | 0,0234 |
| A\_51\_P221677 | NM\_178110 | FINGER PROTEIN FINGER PROTEIN TRIPARTITE MOTIF PROTEIN | 1.93 | 0,0101 |
| A\_51\_P453810 | NM\_207215 | PROTEIN ASSOCIATED WITH | 1.93 | 0,0059 |
| A\_51\_P230722 | NM\_001025572 | Mus musculus, Similar to KIAA0874 protein, clone IMAGE:5363060, mRNA | 1.93 | 0,0099 |
| A\_51\_P245564 | NM\_178621 | hypothetical Fibronectin type III domain containing protein | 1.93 | 0,0003 |
| A\_51\_P153556 | A\_51\_P153556 | hypothetical Actin-like ATPase domain structure containing protein | 1.93 | 0,0382 |
| A\_51\_P119039 | NM\_016708 | Mus musculus neuropeptide Y receptor Y5 (Npy5r), mRNA | 1.93 | 0,0341 |
| A\_51\_P269634 | NM\_178733 | zinc finger protein 14 | 1.93 | 0,0079 |
| A\_51\_P160372 | NM\_016757 | Mus musculus WW domain binding protein 1 (Wbp1), mRNA | 1.93 | 0,0102 |
| A\_51\_P186483 | AK049181 | hypothetical Phosphoinositide 3-kinase family, ras-binding domain containing protein | 1.92 | 0,0060 |
| A\_51\_P467682 | NM\_027115 | hypothetical protein | 1.92 | 0,0023 |
| A\_51\_P286946 | NM\_029609 | PHOSPHOLYSINE PHOSPHOHISTIDINE INORGANIC PYROPHOSPHATE PHOSPHATASE homolog [Homo sapiens] | 1.92 | 0,0021 |
| A\_51\_P395686 | AK017899 | similar to RIBOSOMAL PROTEIN S6 (FRAGMENT) [Sus scrofa] | 1.92 | 0,0194 |
| A\_51\_P314285 | NM\_026436 | hypothetical protein | 1.92 | 0,0079 |
| A\_51\_P148592 | NM\_026045 | Mus musculus, Similar to pre-mRNA processing factor 18, clone IMAGE:3496857, mRNA | 1.92 | 0,0025 |
| A\_51\_P164203 | NM\_019731 | Mus musculus expressed in non-metastatic cells 4, protein (NM23-M4)(nucleoside diphosphate kinase) (Nme4), mRNA | 1.92 | 0,0005 |
| A\_51\_P154679 | NM\_173186 | hypothetical RabGAP/TBC domain containing protein | 1.92 | 0,0007 |
| A\_51\_P224928 | AK010706 | Mus musculus ELAV (embryonic lethal, abnormal vision, Drosophila)-like 1 (Hu antigen R) (Elavl1), mRNA | 1.92 | 0,0136 |
| A\_51\_P446085 | NM\_028908 | hypothetical Serine-rich region containing protein | 1.92 | 0,0371 |
| A\_51\_P482029 | NM\_053177 | Mus musculus mucolipin 1 (Mcoln1), mRNA | 1.92 | 0,0023 |
| A\_51\_P360840 | NM\_009569 | Mus musculus zinc finger protein, multitype 1 (Zfpm1), mRNA | 1.92 | 0,0030 |
| A\_51\_P385370 | AK004304 | LIM ONLY PROTEIN 6 TRIPLE LIM DOMAIN PROTEIN | 1.92 | 0,0030 |
| A\_51\_P483589 | NM\_198162 | hypothetical protein | 1.92 | 0,0038 |
| A\_51\_P440694 | NM\_029846 | CDNA FLJ14948 FIS, CLONE PLACE2000164, WEAKLY SIMILAR TO TIPD PROTEIN homolog [Homo sapiens] | 1.92 | 0,0122 |
| A\_51\_P381657 | NM\_146144 | Mus musculus, Similar to ubiquitin specific protease 1, clone MGC:25528 IMAGE:3585191, mRNA, complete cds | 1.92 | 0,0145 |
| A\_51\_P375431 | AK083100 | unknown EST | 1.92 | 0,0025 |
| A\_51\_P211483 | NM\_024472 | Mus musculus hypothetical protein, MGC:7473 (BC002216), mRNA | 1.92 | 0,0002 |
| A\_51\_P401304 | NM\_029665 | hypothetical protein | 1.92 | 0,0014 |
| A\_51\_P352402 | NM\_026149 | CHRONIC MYELOGENOUS LEUKEMIA TUMOR ANTIGEN 66 homolog [Homo sapiens] | 1.92 | 0,0082 |
| A\_51\_P484200 | NM\_153777 | hypothetical Leucine-rich repeat containing protein | 1.92 | 0,0022 |
| A\_51\_P234373 | NM\_012035 | Mus musculus transient receptor protein 8 (Trrp8), mRNA | 1.91 | 0,0123 |
| A\_51\_P442402 | NM\_029657 | hypothetical Crystallin/RING finger containing protein | 1.91 | 0,0005 |
| A\_51\_P195066 | NM\_013658 | Mus musculus sema domain, immunoglobulin domain (Ig), transmembrane domain (TM) and short cytoplasmic domain, (semaphorin) 4A (Sema4a), mRNA | 1.91 | 0,0083 |
| A\_51\_P254864 | AK122311 | Mus musculus optic atrophy 1 homolog (human) (Opa1), mRNA | 1.91 | 0,0044 |
| A\_51\_P348624 | NM\_013899 | Mus musculus translocase of inner mitochondrial membrane 13 homolog a (yeast) (Timm13a), mRNA | 1.91 | 0,0006 |
| A\_51\_P348433 | NM\_013832 | Mus musculus RAS protein activator like 1 (GAP1 like) (Rasal1), mRNA | 1.91 | 0,0122 |
| A\_51\_P382928 | NM\_145529 | CLEAVAGE STIMULATION FACTOR 77KDA SUBUNIT homolog [Homo sapiens] | 1.91 | 0,0045 |
| A\_51\_P209930 | NM\_013648 | Mus musculus reticulon 2 (Z-band associated protein) (Rtn2), mRNA | 1.91 | 0,0053 |
| A\_51\_P117226 | AK046533 | SMALL REC homolog [Rattus norvegicus] | 1.91 | 0,0276 |
| A\_51\_P432779 | XM\_134593 | unknown EST | 1.91 | 0,0018 |
| A\_51\_P320668 | BC053106 | ACTIN-RELATED PROTEIN 3-BETA homolog [Homo sapiens] | 1.91 | 0,0332 |
| A\_51\_P217946 | AK083761 | unknown EST | 1.91 | 0,0045 |
| A\_51\_P506204 | NM\_009866 | Mus musculus cadherin 11 (Cdh11), mRNA | 1.91 | 0,0365 |
| A\_51\_P464530 | NM\_213614 | Mus musculus CDCREL-1 homolog (Cdcrel-1) mRNA, partial cds | 1.91 | 0,0119 |
| A\_51\_P132081 | NM\_001013769 | weakly similar to ZINC FINGER PROTEIN 43 (HTF6) [Homo sapiens] | 1.89 | 0,0300 |
| A\_51\_P438235 | NM\_027591 | hypothetical Microbodies C-terminal targeting signal containing protein | 1.89 | 0,0008 |
| A\_51\_P246744 | NM\_021435 | Mus musculus hypothetical protein, MNCb-4414 (AB041549), mRNA | 1.89 | 0,0030 |
| A\_51\_P173212 | NM\_144500 | Mus musculus oxysterol-binding protein-like 2 (Osbpl2), mRNA | 1.89 | 0,0012 |
| A\_51\_P483690 | X61450 | Mus musculus brain protein 14 (Brp14), mRNA | 1.89 | 0,0009 |
| A\_51\_P191354 | XM\_126946 | weakly similar to PEROXISOMAL LONG CHAIN ACYL-COA THIOESTERASE IB [Mus musculus] | 1.89 | 0,0134 |
| A\_51\_P259550 | NM\_172876 | DJ50O24.1 (NOVEL PROTEIN SIMILAR TO C. ELEGANS K07B1.7 (TR:O01886)) (FRAGMENT) homolog [Homo sapiens] | 1.89 | 0,0028 |
| A\_51\_P174415 | NM\_170755 | hypothetical protein | 1.89 | 0,0029 |
| A\_51\_P149502 | NM\_145217 | BC41195\_1 (RIG PROTEIN) (SMALL GTP-BINDING TUMOR SUPPRESSOR 1) homolog [Homo sapiens] | 1.89 | 0,0029 |
| A\_51\_P413122 | NM\_028126 | SEROLOGICALLY DEFINED BREAST CANCER ANTIGEN NY-BR-96 homolog [Homo sapiens] | 1.89 | 0,0017 |
| A\_51\_P356353 | AK017654 | unknown EST | 1.89 | 0,0005 |
| A\_51\_P350214 | TC1431688 | Mus musculus cDNA, 5 end | 1.89 | 0,0017 |
| A\_51\_P298514 | NM\_177345 | Mus musculus expressed sequence AI591529 (AI591529), mRNA | 1.88 | 0,0019 |
| A\_51\_P332169 | NM\_173390 | Mus musculus, clone IMAGE:3591967, mRNA, partial cds | 1.88 | 0,0011 |
| A\_51\_P334490 | NM\_172286 | hypothetical protein | 1.88 | 0,0079 |
| A\_51\_P455410 | NM\_028780 | Mus musculus transmembrane 9 superfamily member 1 (Tm9sf1), mRNA | 1.88 | 0,0001 |
| A\_51\_P424054 | BC055704 | BTB (POZ) domain containing 2 | 1.88 | 0,0032 |
| A\_51\_P498291 | NM\_029007 | Mus musculus mRNA, one isoform of PTP-RL9 | 1.88 | 0,0397 |
| A\_51\_P304126 | NM\_026267 | hypothetical protein | 1.88 | 0,0230 |
| A\_51\_P345396 | NM\_173399 | unknown EST | 1.88 | 0,0310 |
| A\_51\_P440707 | BC049250 | Mus musculus RIKEN cDNA 2410004N11 gene (2410004N11Rik), mRNA | 1.88 | 0,0012 |
| A\_51\_P392776 | XM\_622789 | hypothetical G-protein beta WD-40 repeats containing protein | 1.88 | 0,0075 |
| A\_51\_P325065 | NAP057120-1 | Mus musculus olfactory receptor GA\_x5J8B7W531T-1577060-1577325 (GA\_x5J8B7W531T-1577060-1577325) pseudogene | 1.88 | 0,0450 |
| A\_51\_P399071 | NM\_009672 | Mus musculus cDNA, 5 end | 1.88 | 0,0020 |
| A\_51\_P507053 | NM\_134086 | Mus musculus solute carrier family 38, member 1 (Slc38a1), mRNA | 1.88 | 0,0068 |
| A\_51\_P172523 | AK029960 | similar to CTCL TUMOR ANTIGEN SE2-2 (FRAGMENT) [Homo sapiens] | 1.88 | 0,0051 |
| A\_51\_P460067 | NM\_178709 | hypothetical RING finger containing protein | 1.88 | 0,0099 |
| A\_51\_P468388 | NM\_011235 | Mus musculus RAD51-like 3 (S. cerevisiae) (Rad51l3), mRNA | 1.88 | 0,0018 |
| A\_51\_P308469 | NM\_011884 | Mus musculus RNA guanylyltransferase and 5-phosphatase (Rngtt), mRNA | 1.88 | 0,0010 |
| A\_51\_P138185 | NM\_134083 | Mus musculus expressed sequence AW240694 (AW240694), mRNA | 1.88 | 0,0070 |
| A\_51\_P271665 | NM\_011264 | Mus musculus Sez4 mRNA for DNA polymerase, complete cds | 1.88 | 0,0034 |
| A\_51\_P295701 | NM\_026721 | hypothetical ZIP Zinc transporter containing protein | 1.88 | 0,0028 |
| A\_51\_P169783 | AK086378 | SIMILAR TO CONSERVED GENE TELOMERIC TO ALPHA GLOBIN CLUSTER (UNKNOWN) (PROTEIN FOR MGC:4491) homolog [Homo sapiens] | 1.88 | 0,0005 |
| A\_51\_P143470 | NM\_011592 | Mus musculus translocator of inner mitochondrial membrane 44 (Timm44), mRNA | 1.88 | 0,0003 |
| A\_51\_P386638 | NM\_008502 | Mus musculus lethal giant larvae homolog (Llglh), mRNA | 1.88 | 0,0029 |
| A\_51\_P348119 | NM\_027315 | unknown EST | 1.88 | 0,0005 |
| A\_51\_P367295 | BU554808 | Mus musculus cDNA, 5 end | 1.88 | 0,0189 |
| A\_51\_P111962 | XM\_486154 | Mus musculus Nedd4-binding brain specific protein BEAN mRNA, partial cds | 1.88 | 0,0217 |
| A\_51\_P433428 | NM\_007905 | Mus musculus early development regulator 1 (homolog of polyhomeotic 1) (Edr1), mRNA | 1.88 | 0,0043 |
| A\_51\_P407683 | NM\_145540 | hypothetical Glycine-rich region/Immunoglobulin and major histocompatibility complex domain containing protein | 1.88 | 0,0063 |
| A\_51\_P504863 | NM\_008795 | Mus musculus PCTAIRE-motif protein kinase 3 (Pctk3), mRNA | 1.88 | 0,0255 |
| A\_51\_P210470 | NM\_026310 | Mus musculus RIKEN cDNA 1010001C05 gene (1010001C05Rik), mRNA | 1.87 | 0,0017 |
| A\_51\_P222467 | NM\_009593 | Mus musculus ATP-binding cassette, sub-family G (WHITE), member 1 (Abcg1), mRNA | 1.87 | 0,0027 |
| A\_51\_P424561 | NM\_009567 | Mus musculus zinc finger protein 93 (Zfp93), mRNA | 1.87 | 0,0473 |
| A\_51\_P468240 | XM\_134902 | c-myc promoter-binding protein irlB (fragment) homolog [Homo sapiens] | 1.87 | 0,0006 |
| A\_51\_P261443 | NM\_011550 | Mus musculus transcription factor-like 4 (Tcfl4), mRNA | 1.87 | 0,0002 |
| A\_51\_P384609 | XM\_284494 | DEAD/H (Asp-Glu-Ala-Asp/His) box polypeptide 10 (RNA helicase) | 1.87 | 0,0356 |
| A\_51\_P494406 | NM\_145484 | Mus musculus, clone MGC:29357 IMAGE:5038660, mRNA, complete cds | 1.87 | 0,0072 |
| A\_51\_P412338 | NM\_172120 | VACUOLAR ASSEMBLY PROTEIN VPS41 HOMOLOG (S53) homolog [Homo sapiens] | 1.87 | 0,0005 |
| A\_51\_P354744 | NM\_144874 | SIMILAR TO COX15 HOMOLOG, CYTOCHROME C OXIDASE ASSEMBLY PROTEIN (YEAST) (FRAGMENT) homolog [Mus musculus] | 1.87 | 0,0004 |
| A\_51\_P372186 | NM\_020589 | Mus musculus hypothetical protein, MNCb-3350 (AB041616), mRNA | 1.87 | 0,0040 |
| A\_51\_P145360 | NM\_177879 | hypothetical Fibronectin type III domain containing protein | 1.87 | 0,0019 |
| A\_51\_P341255 | AK078885 | unknown EST | 1.87 | 0,0124 |
| A\_51\_P441327 | NM\_175034 | hypothetical K+-dependent Na+/Ca+ exchanger related-protein containing protein | 1.87 | 0,0007 |
| A\_51\_P107934 | NM\_172858 | SERINE/THREONINE-PROTEIN KINASE PAK 5 (EC 2.7.1.-) (P21-ACTIVATED KINASE 5) (PAK-5) homolog [Homo sapiens] | 1.87 | 0,0119 |
| A\_51\_P313483 | NM\_007890 | Mus musculus mnb protein kinase homolog mp86 (Dyrk) mRNA, complete cds | 1.87 | 0,0055 |
| A\_51\_P130773 | NM\_009613 | Mus musculus a disintegrin and metalloprotease domain 11 (Adam11), mRNA | 1.87 | 0,0445 |
| A\_51\_P120008 | BC068151 | similar to 3 of D-CONTAINING PROTEIN (FRAGMENT) [Rattus norvegicus] | 1.87 | 0,0005 |
| A\_51\_P340987 | NM\_178888 | hypothetical protein | 1.85 | 0,0008 |
| A\_51\_P356283 | NM\_133765 | Mus musculus RIKEN cDNA 2310046N15 gene (2310046N15Rik), mRNA | 1.85 | 0,0229 |
| A\_51\_P347965 | NM\_007427 | Mus musculus agouti related protein (Agrp), mRNA | 1.85 | 0,0171 |
| A\_51\_P215046 | NM\_133677 | Mus musculus RIKEN cDNA 2310061J03 gene (2310061J03Rik), mRNA | 1.85 | 0,0080 |
| A\_51\_P217430 | NM\_023633 | Mus musculus RIKEN cDNA 2410016O06 gene (2410016O06Rik), mRNA | 1.85 | 0,0002 |
| A\_51\_P406179 | NM\_025538 | inferred: putative [Mus musculus] | 1.85 | 0,0132 |
| A\_51\_P450756 | BC051044 | hypothetical Ubiquitin-protein ligase E3a, Hect catalytic domain (E6ap) structure containing protein | 1.85 | 0,0374 |
| A\_51\_P144770 | XM\_619964 | Mus musculus, clone MGC:27669 IMAGE:4910895, mRNA, complete cds | 1.85 | 0,0003 |
| A\_51\_P273508 | BC065083 | unknown EST | 1.85 | 0,0054 |
| A\_51\_P354766 | NM\_007944 | Mus musculus epidermal growth factor receptor pathway substrate 15, related sequence (Eps15-rs), mRNA | 1.85 | 0,0017 |
| A\_51\_P293989 | NM\_146003 | similar to SUMO-1-SPECIFIC PROTEASE 1 (EC 3.4.22.-) (SENTRIN-SPECIFIC PROTEASE SENP6) (PROTEASE FKSG6) [Homo sapiens] | 1.85 | 0,0007 |
| A\_51\_P142653 | NM\_178880 | SON PROTEIN homolog [Mus musculus] | 1.85 | 0,0012 |
| A\_51\_P373609 | NM\_198246 | CDNA FLJ13995 FIS, CLONE Y79AA1002209, WEAKLY SIMILAR TO TYROSYL-TRNA SYNTHETASE (EC 6.1.1.1) (HYPOTHETICAL 53.2 KDA PROTEIN) homolog [Homo sapiens] | 1.85 | 0,0021 |
| A\_51\_P402775 | AK090134 | Mus musculus meiotic check point regulator (Mcpr), mRNA | 1.85 | 0,0066 |
| A\_51\_P296512 | NM\_018818 | Mus musculus choroidermia (Chm), mRNA | 1.85 | 0,0011 |
| A\_51\_P188574 | XM\_146997 | weakly similar to MDC-3.13 ISOFORM 2 (TNF-INDUCED PROTEIN) [Homo sapiens] | 1.85 | 0,0055 |
| A\_51\_P346722 | NM\_013865 | Mus musculus N-myc downstream regulated 3 (Ndr3), mRNA | 1.85 | 0,0178 |
| A\_51\_P174512 | AK078076 | hypothetical Sushi domain / SCR repeat / CCP module containing protein | 1.85 | 0,0033 |
| A\_51\_P329842 | NM\_027998 | Mus musculus, RIKEN cDNA 2310014B08 gene, clone MGC:28564 IMAGE:4207924, mRNA, complete cds | 1.85 | 0,0271 |
| A\_51\_P104172 | NM\_153802 | ZINC FINGER PROTEIN 4 (FRAGMENT) homolog [Rattus norvegicus] | 1.85 | 0,0066 |
| A\_51\_P354706 | NM\_010094 | Mus musculus endometrial bleeding associated factor (Ebaf), mRNA | 1.85 | 0,0304 |
| A\_51\_P443394 | NM\_134150 | Mus musculus expressed sequence AI850305 (AI850305), mRNA | 1.85 | 0,0019 |
| A\_51\_P171200 | NM\_027307 | unknown EST | 1.85 | 0,0047 |
| A\_51\_P128667 | NM\_011838 | Mus musculus Ly6/neurotoxin 1 (Lynx1), mRNA | 1.85 | 0,0137 |
| A\_51\_P280200 | NM\_009462 | Mus musculus ubiquintin c-terminal hydrolase related polypeptide (Uchrp), mRNA | 1.85 | 0,0057 |
| A\_51\_P507899 | NM\_029553 | Mus musculus, Similar to RIKEN cDNA 0610012F22 gene, clone MGC:27659 IMAGE:4527657, mRNA, complete cds | 1.85 | 0,0025 |
| A\_51\_P473918 | BC022619 | KIAA0374 (SYNTAPHILIN) (BA314N13.1.1) homolog [Homo sapiens] | 1.85 | 0,0118 |
| A\_51\_P345274 | NM\_194268 | RIKEN full-length enriched, 12 days embryo spinal ganglion Mus musculus cDNA clone D130071B21 5, mRNA sequence | 1.85 | 0,0099 |
| A\_51\_P440838 | NM\_026218 | Mus musculus RIKEN cDNA 1500031J01 gene (1500031J01Rik), mRNA | 1.85 | 0,0077 |
| A\_51\_P263373 | NM\_177588 | hypothetical Pyridoxal-5-phosphate-dependent enzymes, beta family containing protein | 1.85 | 0,0267 |
| A\_51\_P371194 | NM\_013917 | PITUITARY TUMOR-TRANSFORMING 1 | 1.84 | 0,0027 |
| A\_51\_P498322 | NM\_145452 | Mus musculus, Similar to RAS p21 protein activator, clone MGC:7759 IMAGE:3498774, mRNA, complete cds | 1.84 | 0,0224 |
| A\_51\_P491886 | NM\_009428 | Mus musculus transient receptor protein 5 (Trrp5), mRNA | 1.84 | 0,0221 |
| A\_51\_P162624 | NM\_133678 | Mus musculus RIKEN cDNA 2410004C24 gene (2410004C24Rik), mRNA | 1.84 | 0,0070 |
| A\_51\_P449361 | NM\_010413 | Mus musculus histone deacetylase 6 (Hdac6), mRNA | 1.84 | 0,0087 |
| A\_51\_P451847 | NM\_025814 | Mus musculus RIKEN cDNA 1200009K13 gene (1200009K13Rik), mRNA | 1.84 | 0,0019 |
| A\_51\_P144957 | NM\_146140 | weakly similar to translocating chain-associating membrane protein [Canis lupus familiaris] | 1.84 | 0,0193 |
| A\_51\_P371882 | NM\_133744 | Mus musculus RIKEN cDNA 2600016J21 gene (2600016J21Rik), mRNA | 1.84 | 0,0011 |
| A\_51\_P113806 | NM\_145066 | Mus musculus orphan G protein-coupled receptor 85 (Gpr85) mRNA, complete cds | 1.84 | 0,0263 |
| A\_51\_P375453 | NM\_139298 | WNT14 homolog [Homo sapiens] | 1.84 | 0,0162 |
| A\_51\_P215038 | NM\_182991 | similar to BRAIN SPECIFIC MEMBRANE-ANCHORED PROTEIN PRECURSOR [Homo sapiens] | 1.84 | 0,0207 |
| A\_51\_P194910 | TC1430357 | Mus musculus N-myristoyltransferase 1 (Nmt1), mRNA | 1.84 | 0,0013 |
| A\_51\_P425632 | NM\_025649 | Mus musculus RIKEN cDNA 0610009D16 gene (0610009D16Rik), mRNA | 1.84 | 0,0029 |
| A\_51\_P218975 | NM\_173417 | voltage-gated potassium channel alpha chain Kv9.3 homolog [Rattus norvegicus] | 1.84 | 0,0019 |
| A\_51\_P134835 | NM\_207685 | Mus musculus ELAV (embryonic lethal, abnormal vision, Drosophila)-like 2 (Hu antigen B) (Elavl2), mRNA | 1.84 | 0,0075 |
| A\_51\_P427825 | AK009836 | hypothetical protein | 1.84 | 0,0123 |
| A\_51\_P412966 | NM\_008516 | Mus musculus leucine rich repeat protein 1, neuronal (Lrrn1), mRNA | 1.84 | 0,0305 |
| A\_51\_P204402 | NM\_011369 | Mus musculus Shc SH2-domain binding protein 1 (Shcbp1), mRNA | 1.84 | 0,0004 |
| A\_51\_P246754 | NM\_134021 | Mus musculus expressed sequence AI415282 (AI415282), mRNA | 1.84 | 0,0017 |
| A\_51\_P339356 | NM\_178607 | RING FINGER PROTEIN 24 homolog [Homo sapiens] | 1.84 | 0,0026 |
| A\_51\_P334263 | NM\_133227 | Mus musculus nucleoporin 155 (Nup155), mRNA | 1.84 | 0,0023 |
| A\_51\_P411757 | NM\_172945 | inferred: KE03 protein {Homo sapiens} | 1.84 | 0,0021 |
| A\_51\_P207988 | NM\_008965 | Mus musculus prostaglandin E receptor 4 (subtype EP4) (Ptger4), mRNA | 1.84 | 0,0002 |
| A\_51\_P329413 | NM\_029786 | O-LINKED MANNOSE BETA1,2-N-ACETYLGLUCOSAMINYLTRANSFERASE homolog [Homo sapiens] | 1.84 | 0,0020 |
| A\_51\_P121622 | AK004273 | unknown EST | 1.84 | 0,0009 |
| A\_51\_P392134 | NM\_080560 | Mus musculus ubiquitin-conjugating enzyme E2N (Ube2n), mRNA | 1.83 | 0,0040 |
| A\_51\_P365153 | NM\_010474 | Mus musculus heparan sulfate (glucosamine) 3-O-sulfotransferase 1 (Hs3st1), mRNA | 1.83 | 0,0391 |
| A\_51\_P456816 | NM\_172932 | NEUROLIGIN 3 ISOFORM HNL3 homolog [Homo sapiens] | 1.83 | 0,0010 |
| A\_51\_P321984 | AK045803 | unclassifiable | 1.83 | 0,0080 |
| A\_51\_P415806 | NM\_026954 | hypothetical protein | 1.83 | 0,0102 |
| A\_51\_P255832 | NM\_019409 | Mus musculus oligodendrocyte myelin glycoprotein (Omg), mRNA | 1.83 | 0,0269 |
| A\_51\_P333839 | NM\_019971 | Mus musculus platelet-derived growth factor, C polypeptide (Pdgfc), mRNA | 1.83 | 0,0020 |
| A\_51\_P482628 | NM\_019924 | Mus musculus ribosomal protein S6 kinase, 90kD, polypeptide 4 (Rps6ka4), mRNA | 1.83 | 0,0048 |
| A\_51\_P229602 | AK028562 | PLEXIN 2 | 1.83 | 0,0112 |
| A\_51\_P142091 | NM\_030121 | Mus musculus, ankyrin repeat and SOCS box-containing protein 8, clone MGC:36839 IMAGE:4194829, mRNA, complete cds | 1.83 | 0,0170 |
| A\_51\_P223085 | AK014830 | unclassifiable | 1.83 | 0,0275 |
| A\_51\_P140797 | AK004618 | similar to PC326 PROTEIN [Homo sapiens] | 1.83 | 0,0066 |
| A\_51\_P224564 | NM\_176833 | CA 2+ /CALMODULIN DEPENDENT PROTEIN KINASE PHOSPHATASE EC 3.1.3.16 | 1.83 | 0,0161 |
| A\_51\_P337110 | NM\_027694 | similar to SIMILAR TO HYPOTHETICAL PROTEIN, MNCB-1213 [Homo sapiens] | 1.83 | 0,0138 |
| A\_51\_P142450 | NM\_027865 | hypothetical Immunoglobulin structure containing protein | 1.83 | 0,0156 |
| A\_51\_P236013 | NM\_172281 | KIAA0661 PROTEIN (95 KDA RETINOBLASTOMA PROTEIN BINDING PROTEIN, KIAA0661 GENE PRODUCT) (SIMILAR TO 95 KDA RETINOBLASTOMA PROTEIN BINDING PROTEIN) homolog [Homo sapiens] | 1.83 | 0,0038 |
| A\_51\_P169087 | XM\_125928 | GLUTAMINASE, LIVER ISOFORM, MITOCHONDRIAL PRECURSOR (EC 3.5.1.2) (GLS) (L-GLUTAMINE AMIDOHYDROLASE) (L-GLUTAMINASE) homolog [Rattus norvegicus] | 1.83 | 0,0193 |
| A\_51\_P432764 | BC062197 | Mus musculus protein tyrosine phosphatase, non-receptor type substrate 1 (Ptpns1), mRNA | 1.83 | 0,0385 |
| A\_51\_P423290 | XM\_284198 | weakly similar to multimerin, endothelial cell, precursor [Homo sapiens] | 1.83 | 0,0172 |
| A\_51\_P419726 | NM\_011218 | Mouse mRNA for protein tyrosine phosphatase PTPT9 | 1.83 | 0,0420 |
| A\_51\_P199095 | NM\_025838 | similar to DC20 [Homo sapiens] | 1.83 | 0,0054 |
| A\_51\_P268193 | NM\_017394 | Mus musculus solute carrier family 7 (cationic amino acid transporter, y+ system), member 10 (Slc7a10), mRNA | 1.83 | 0,0357 |
| A\_51\_P464300 | NM\_008107 | Mus musculus growth differentiation factor 1 (Gdf1), mRNA | 1.82 | 0,0366 |
| A\_51\_P368262 | NM\_011630 | mTR2R1=type II zinc finger DNA binding transcription factor [mice, neonatal brain, mRNA, 2339 nt] | 1.82 | 0,0018 |
| A\_51\_P179082 | NM\_011624 | Mus musculus topoisomerase (DNA) III beta (Top3b), mRNA | 1.82 | 0,0018 |
| A\_51\_P147284 | NM\_198014 | unclassifiable | 1.82 | 0,0176 |
| A\_51\_P488819 | AK016591 | hypothetical Arginine-rich region containing protein | 1.82 | 0,0469 |
| A\_51\_P168647 | AK039580 | Mus musculus cDNA, 5 end | 1.82 | 0,0299 |
| A\_51\_P206268 | BC027244 | SEC13-LIKE PROTEIN homolog [Homo sapiens] | 1.82 | 0,0141 |
| A\_51\_P498403 | NM\_028065 | Mus musculus putative retinoic acid-regulated protein mRNA, complete cds | 1.82 | 0,0016 |
| A\_51\_P232980 | XM\_355244 | BG120K12 3 NOVEL PROTEIN SIMILAR TO ARCHAEAL, YEAST AND WORM N2,N2 DIMETHYLGUANOSINE TRNA METHYLTRANSFERASE ISOFORM | 1.82 | 0,0001 |
| A\_51\_P314852 | NM\_001030014 | B2 3 PUTATIVE NOVEL ACYL TRANSFERASE ISOFORM | 1.82 | 0,0075 |
| A\_51\_P442091 | AK122352 | unknown EST | 1.82 | 0,0168 |
| A\_51\_P489108 | NM\_153391 | Mus musculus WD repeat membrane protein PWDMP (PWDMP), mRNA | 1.82 | 0,0002 |
| A\_51\_P294165 | AK004569 | PRE-MRNA SPLICING FACTOR PRP17 homolog [Mus musculus] | 1.82 | 0,0382 |
| A\_51\_P512541 | AK049709 | similar to HCDI PROTEIN [Homo sapiens] | 1.82 | 0,0002 |
| A\_51\_P460404 | BC016248 | Mus musculus, Similar to zinc finger protein 85 (HPF4, HTF1), clone MGC:28872 IMAGE:4527362, mRNA, complete cds | 1.82 | 0,0030 |
| A\_51\_P385390 | NM\_022418 | Mus musculus RIKEN cDNA 2010004O20 gene (2010004O20Rik), mRNA | 1.82 | 0,0060 |
| A\_51\_P142175 | NM\_021295 | Mus musculus mRNA for LanC-like protein 1 (Lancl1 gene) | 1.82 | 0,0328 |
| A\_51\_P451301 | NM\_178764 | hypothetical protein | 1.82 | 0,0132 |
| A\_51\_P139069 | NM\_172372 | SIMILAR TO JM5 PROTEIN homolog [Mus musculus] | 1.82 | 0,0094 |
| A\_51\_P450123 | NM\_053163 | Mus musculus mitochondrial ribosomal protein L36 (Mrpl36), mRNA | 1.82 | 0,0193 |
| A\_51\_P275469 | NM\_177581 | Mus musculus, clone IMAGE:5054193, mRNA | 1.82 | 0,0076 |
| A\_51\_P332361 | NM\_027213 | RNA POLYMERASE II TRANSCRIPTIONAL REGULATION MEDIATOR (MED6, S. CEREVISIAE, HOMOLOG OF) homolog [Homo sapiens] | 1.82 | 0,0112 |
| A\_51\_P465600 | NM\_028344 | hypothetical protein | 1.80 | 0,0002 |
| A\_51\_P288277 | AK003987 | unknown EST | 1.80 | 0,0174 |
| A\_51\_P107433 | NM\_053162 | Mus musculus mitochondrial ribosomal protein L34 (Mrpl34), mRNA | 1.80 | 0,0455 |
| A\_51\_P148196 | NM\_009143 | Mus musculus stromal cell derived factor 2 (Sdf2), mRNA | 1.80 | 0,0032 |
| A\_51\_P324134 | NM\_138646 | Mus musculus Hermansky-Pudlak syndrome 4 homolog (human) (Hps4), mRNA | 1.80 | 0,0102 |
| A\_51\_P127492 | NM\_011228 | Mus musculus RAB33A, member of RAS oncogene family (Rab33a), mRNA | 1.80 | 0,0007 |
| A\_51\_P406414 | NM\_010470 | Mus musculus, heterochromatin protein 2, binding protein 3, clone MGC:28927 IMAGE:3597151, mRNA, complete cds | 1.80 | 0,0021 |
| A\_51\_P119633 | NM\_008043 | Mus musculus frequently rearranged in advanced T-cell lymphomas (Frat1), mRNA | 1.80 | 0,0023 |
| A\_51\_P299432 | NM\_026670 | Mus musculus RIKEN cDNA 5830412B09 gene (5830412B09Rik), mRNA | 1.80 | 0,0091 |
| A\_51\_P203547 | AK015642 | Mus musculus RIKEN cDNA 4930488B01 gene (4930488B01Rik), mRNA | 1.80 | 0,0089 |
| A\_51\_P207310 | U85089 | Mus musculus thioredoxin 2 (Txn2), mRNA | 1.80 | 0,0002 |
| A\_51\_P107599 | NM\_008541 | unknown EST | 1.80 | 0,0253 |
| A\_51\_P247180 | AK030974 | hypothetical Tetratricopeptide repeat (TPR) structure containing protein | 1.80 | 0,0012 |
| A\_51\_P143721 | NM\_134044 | Mus musculus expressed sequence AI413782 (AI413782), mRNA | 1.80 | 0,0109 |
| A\_51\_P151775 | NM\_178719 | hypothetical protein | 1.80 | 0,0104 |
| A\_51\_P129929 | NM\_177899 | ZINC FINGER PROTEIN 5 (FRAGMENT) homolog [Rattus norvegicus] | 1.80 | 0,0047 |
| A\_51\_P414034 | A\_51\_P414034 | Mus musculus cDNA, 3 end | 1.80 | 0,0070 |
| A\_51\_P142760 | NM\_029102 | GLYCOSYLTRANSFERASE | 1.80 | 0,0008 |
| A\_51\_P492070 | NM\_133953 | Mus musculus expressed sequence AA409318 (AA409318), mRNA | 1.80 | 0,0007 |
| A\_51\_P168219 | NM\_026401 | Mus musculus mitochondrial ribosomal protein 63 (Mrp63), mRNA | 1.80 | 0,0279 |
| A\_51\_P452528 | BC035277 | unknown EST | 1.80 | 0,0285 |
| A\_51\_P379552 | XM\_132047 | unknown EST | 1.80 | 0,0284 |
| A\_51\_P424079 | NM\_010122 | Mus musculus eukaryotic translation initiation factor 2B (Eif2b), mRNA | 1.80 | 0,0032 |
| A\_51\_P117664 | XM\_129477 | hypothetical protein | 1.80 | 0,0189 |
| A\_51\_P264495 | NM\_018870 | Mus musculus phosphoglycerate mutase 2 (Pgam2), mRNA | 1.80 | 0,0175 |
| A\_51\_P318491 | NM\_145964 | Mus musculus, Similar to hypothetical protein PRO0971, clone MGC:7434 IMAGE:3489243, mRNA, complete cds | 1.79 | 0,0105 |
| A\_51\_P467869 | NM\_007389 | Mus musculus cholinergic receptor, nicotinic, alpha polypeptide 1 (muscle) (Chrna1), mRNA | 1.79 | 0,0083 |
| A\_51\_P259726 | NM\_054046 | Mus musculus differentially expressed in FDCP 8 (Def8), mRNA | 1.79 | 0,0057 |
| A\_51\_P450829 | NM\_133987 | Mus musculus expressed sequence AA589632 (AA589632), mRNA | 1.79 | 0,0205 |
| A\_51\_P133229 | NM\_028072 | Mus musculus, RIKEN cDNA 2010004N24 gene, clone MGC:27878 IMAGE:3495588, mRNA, complete cds | 1.79 | 0,0448 |
| A\_51\_P181175 | NM\_021447 | Mus musculus ring finger protein 30 (Rnf30), mRNA | 1.79 | 0,0001 |
| A\_51\_P251289 | NM\_145534 | Mus musculus similar to BTB/POZ domain containing protein 3 (LOC235826), mRNA | 1.79 | 0,0010 |
| A\_51\_P494863 | NM\_178926 | hypothetical protein | 1.79 | 0,0028 |
| A\_51\_P451986 | AK043702 | hypothetical protein | 1.79 | 0,0406 |
| A\_51\_P458428 | NM\_138606 | Mus musculus proviral integration site 2 (Pim2), mRNA | 1.79 | 0,0209 |
| A\_51\_P259360 | NM\_181417 | CRP2 BINDING PROTEIN (FRAGMENT) homolog [Homo sapiens] | 1.79 | 0,0016 |
| A\_51\_P304045 | XM\_618798 | E2A-PBX1-ASSOCIATED PROTEIN (FRAGMENT) homolog [Homo sapiens] | 1.79 | 0,0293 |
| A\_51\_P461398 | NM\_053123 | Mus musculus SWI/SNF related, matrix associated, actin dependent regulator of chromatin, subfamily a, member 1 (Smarca1), mRNA | 1.79 | 0,0263 |
| A\_51\_P279100 | NM\_008969 | Mus musculus prostaglandin-endoperoxide synthase 1 (Ptgs1), mRNA | 1.79 | 0,0069 |
| A\_51\_P390389 | AK086718 | unknown EST | 1.79 | 0,0042 |
| A\_51\_P482503 | NM\_009413 | Mus musculus tumor protein D52-like 1 (Tpd52l1), mRNA | 1.79 | 0,0185 |
| A\_51\_P213051 | NM\_019752 | Mus musculus protease, serine, 25 (Prss25), mRNA | 1.79 | 0,0012 |
| A\_51\_P447471 | NM\_172662 | Mus musculus RIKEN cDNA E330008O22 gene (E330008O22Rik), mRNA | 1.79 | 0,0044 |
| A\_51\_P175974 | BC038488 | Mus musculus cDNA, 5 end | 1.79 | 0,0040 |
| A\_51\_P380986 | NM\_007935 | Mus musculus enhancer of polycomb homolog 1, (Drosophila) (Epc1), mRNA | 1.79 | 0,0037 |
| A\_51\_P167544 | NM\_153584 | hypothetical protein | 1.79 | 0,0113 |
| A\_51\_P397876 | NM\_010134 | Mus musculus engrailed 2 (En2), mRNA | 1.78 | 0,0022 |
| A\_51\_P269173 | NM\_133981 | Mus musculus RIKEN cDNA 8230402H15 gene (8230402H15Rik), mRNA | 1.78 | 0,0019 |
| A\_51\_P430620 | NM\_172436 | solute carrier family 25 (mitochondrial carrier, Aralar), member 12 | 1.78 | 0,0003 |
| A\_51\_P433026 | BC052412 | hypothetical PA-phosphatase related phosphoesterase containing protein | 1.78 | 0,0335 |
| A\_51\_P513059 | NM\_175327 | hypothetical protein | 1.78 | 0,0052 |
| A\_51\_P505172 | NM\_025674 | Mus musculus, Similar to transcription factor 19 (SC1), clone MGC:6711 IMAGE:3585301, mRNA, complete cds | 1.78 | 0,0420 |
| A\_51\_P118885 | NM\_008537 | Mus musculus alpha-methylacyl-CoA racemase (Amacr), mRNA | 1.78 | 0,0054 |
| A\_51\_P311089 | NM\_153128 | KELCH-LIKE PROTEIN C3IP1 (CDNA FLJ14750 FIS, CLONE NT2RP3002948, WEAKLY SIMILAR TO RING CANAL PROTEIN) homolog [Homo sapiens] | 1.78 | 0,0074 |
| A\_51\_P201520 | BC063105 | CCR4-NOT transcription complex, subunit 2 | 1.78 | 0,0079 |
| A\_51\_P162786 | NM\_024475 | Mus musculus hypothetical protein, MGC: 7513 (BC002236), mRNA | 1.78 | 0,0008 |
| A\_51\_P279384 | NM\_016893 | Mus musculus fucosyltransferase 8 (Fut8), mRNA | 1.78 | 0,0269 |
| A\_51\_P142465 | NM\_011239 | Mus musculus RAN binding protein 1 (Ranbp1), mRNA | 1.78 | 0,0011 |
| A\_51\_P257542 | NM\_178756 | weakly similar to DJ846F13.1 (PHOSPHATIDIC ACID PHOSPHATASE TYPE 2C) (FRAGMENT) [Homo sapiens] | 1.78 | 0,0450 |
| A\_51\_P509229 | AK014218 | unknown EST | 1.78 | 0,0016 |
| A\_51\_P167963 | NM\_011792 | Mus musculus beta-site APP cleaving enzyme (Bace), mRNA | 1.78 | 0,0002 |
| A\_51\_P167535 | NM\_010174 | Mus musculus fatty acid binding protein 3, muscle and heart (Fabp3), mRNA | 1.78 | 0,0051 |
| A\_51\_P469898 | AK020659 | hypothetical protein | 1.78 | 0,0008 |
| A\_51\_P265219 | NM\_175523 | unclassifiable | 1.78 | 0,0283 |
| A\_51\_P165914 | NM\_175466 | weakly similar to MSZF47 (FRAGMENT) [Mus musculus] | 1.78 | 0,0128 |
| A\_51\_P256623 | NM\_013629 | Mus musculus putative homeodomain transcription factor (Phtf), mRNA | 1.78 | 0,0104 |
| A\_51\_P433556 | NM\_197993 | Mus musculus, Similar to hypothetical protein FLJ22347, clone MGC:38113 IMAGE:5320398, mRNA, complete cds | 1.78 | 0,0044 |
| A\_51\_P274947 | NM\_026487 | Mus musculus RIKEN cDNA 4921525H23 gene (4921525H23Rik), mRNA | 1.78 | 0,0029 |
| A\_51\_P449449 | A\_51\_P449449 | "Similar to: Mus musculus similar to RP42 homolog� squamous cell carcinoma-related oncogene [Homo sapiens] (LOC234073), mRNA" | 1.78 | 0,0031 |
| A\_51\_P189704 | NM\_025732 | Mus musculus RIKEN cDNA 4921520G13 gene (4921520G13Rik), mRNA | 1.78 | 0,0299 |
| A\_51\_P493700 | XM\_129509 | unknown EST | 1.78 | 0,0043 |
| A\_51\_P461452 | NM\_026275 | Mus musculus RIKEN cDNA 1200003M11 gene (1200003M11Rik), mRNA | 1.77 | 0,0044 |
| A\_51\_P425768 | NM\_026005 | similar to CDNA FLJ30600 FIS, CLONE BRAWH2009360 [Homo sapiens] | 1.77 | 0,0016 |
| A\_51\_P292527 | NM\_028944 | hypothetical EF-hand/2OG-Fe(II) oxygenase superfamily containing protein | 1.77 | 0,0246 |
| A\_51\_P245336 | NM\_009702 | AQUARIUS (FRAGMENT) | 1.77 | 0,0043 |
| A\_51\_P328516 | NM\_145493 | Mus musculus, Similar to hypothetical protein FLJ13993, clone MGC:27587 IMAGE:4501240, mRNA, complete cds | 1.77 | 0,0005 |
| A\_51\_P353592 | NM\_025417 | Mus musculus RIKEN cDNA 1110039H05 gene (1110039H05Rik), mRNA | 1.77 | 0,0036 |
| A\_51\_P162196 | AK013739 | hypothetical protein | 1.77 | 0,0236 |
| A\_51\_P329469 | NM\_173753 | SIMILAR TO PDZ DOMAIN CONTAINING GUANINE NUCLEOTIDE EXCHANGE FACTOR | 1.77 | 0,0095 |
| A\_51\_P205170 | NM\_146175 | ZINC FINGER PROTEIN 282 (HTLV-I U5RE BINDING PROTEIN 1) (HUB-1) homolog [Homo sapiens] | 1.77 | 0,0002 |
| A\_51\_P313120 | AK038526 | CYTOCHROME P450 | 1.77 | 0,0019 |
| A\_51\_P189351 | XM\_131217 | inferred: Mus musculus, clone IMAGE:3590287, mRNA, partial cds / Unknown (protein for IMAGE:3590287) [Mus musculus] | 1.77 | 0,0014 |
| A\_51\_P259638 | NM\_008016 | fibroblast growth factor inducible 15 | 1.77 | 0,0179 |
| A\_51\_P500574 | NM\_025894 | Mus musculus proteasome (prosome, macropain) 26S subunit, non-ATPase, 12 (Psmd12), mRNA | 1.77 | 0,0002 |
| A\_51\_P378079 | NM\_008031 | Mus musculus fragile X mental retardation syndrome 1 homolog (Fmr1), mRNA | 1.77 | 0,0494 |
| A\_51\_P471798 | AK045812 | matrin cyclophilin {Rattus norvegicus} | 1.77 | 0,0207 |
| A\_51\_P264659 | NM\_144518 | hypothetical protein | 1.77 | 0,0005 |
| A\_51\_P280914 | TC1481637 | Mus musculus cDNA | 1.77 | 0,0009 |
| A\_51\_P490817 | NM\_145494 | NAD-DEPENDENT MALIC ENZYME, MITOCHONDRIAL PRECURSOR (EC 1.1.1.38) (NAD-ME) homolog [Homo sapiens] | 1.77 | 0,0010 |
| A\_51\_P323531 | AK006229 | hypothetical protein | 1.77 | 0,0470 |
| A\_51\_P227392 | NM\_133955 | Mus musculus ras homolog gene family, member U (Arhu), mRNA | 1.77 | 0,0037 |
| A\_51\_P509760 | NM\_016860 | Mus musculus actin-related protein 1 homolog A (yeast) (Actr1a), mRNA | 1.77 | 0,0021 |
| A\_51\_P395160 | NM\_175503 | A5D3 PROTEIN homolog [Rattus norvegicus] | 1.77 | 0,0406 |
| A\_51\_P492476 | NM\_031249 | Mus musculus cleavage stimulation factor, 3 pre-RNA subunit 2, 64 kDa, tau (Cstf2t-pending), mRNA | 1.77 | 0,0022 |
| A\_51\_P495767 | NM\_011948 | Mus musculus mitogen activated protein kinase kinase kinase 4 (Map3k4), mRNA | 1.77 | 0,0061 |
| A\_51\_P234174 | NM\_008886 | Mus musculus postmeiotic segregation increased 2 (S. cerevisiae) (Pms2), mRNA | 1.77 | 0,0053 |
| A\_51\_P514898 | NM\_175160 | DHHC-DOMAIN-CONTAINING CYSTEINE-RICH PROTEIN (FRAGMENT) homolog [Homo sapiens] | 1.77 | 0,0102 |
| A\_51\_P297586 | NM\_007566 | Mus musculus baculoviral IAP repeat-containing 6 (Birc6), mRNA | 1.77 | 0,0219 |
| A\_51\_P283876 | NM\_026512 | BIPHENYL HYDROLASE-RELATED PROTEIN homolog [Homo sapiens] | 1.77 | 0,0014 |
| A\_51\_P432380 | NM\_007467 | Mus musculus amyloid beta (A4) precursor-like protein 1 (Aplp1), mRNA | 1.77 | 0,0146 |
| A\_51\_P264487 | NM\_138605 | Mus musculus DNA segment, Chr X, Immunex 48, expressed (DXImx48e), mRNA | 1.77 | 0,0097 |
| A\_51\_P121432 | NM\_175104 | PUTATIVE NUCLEAR PROTEIN homolog [Homo sapiens] | 1.77 | 0,0254 |
| A\_51\_P408946 | NM\_007633 | Mus musculus cyclin E1 (Ccne1), mRNA | 1.77 | 0,0023 |
| A\_51\_P518666 | NM\_133704 | Mus musculus RIKEN cDNA 1810005C06 gene (1810005C06Rik), mRNA | 1.77 | 0,0214 |
| A\_51\_P455572 | NM\_025631 | Mus musculus RIKEN cDNA 2310034L21 gene (2310034L21Rik), mRNA | 1.77 | 0,0022 |
| A\_51\_P260789 | AK039297 | weakly similar to PUTATIVE RNA-BINDING PROTEIN 11 (RNA BINDING MOTIF PROTEIN 11) [Homo sapiens] | 1.77 | 0,0055 |
| A\_51\_P199425 | NM\_133236 | Mus musculus GIG18 (Gig18) mRNA, complete cds | 1.77 | 0,0096 |
| A\_51\_P330090 | NM\_029640 | hypothetical protein | 1.77 | 0,0051 |
| A\_51\_P209996 | M19413 | Mouse testicular alpha tubulin mRNA, 3 end | 1.75 | 0,0264 |
| A\_51\_P336471 | NM\_013842 | Mus musculus X-box binding protein 1 (Xbp1), mRNA | 1.75 | 0,0483 |
| A\_51\_P420859 | NM\_011773 | Mus musculus solute carrier family 30 (zinc transporter), member 3 (Slc30a3), mRNA | 1.75 | 0,0001 |
| A\_51\_P192782 | AK003900 | hypothetical protein | 1.75 | 0,0147 |
| A\_51\_P360918 | NM\_020578 | Mus musculus EH-domain containing 3 (Ehd3), mRNA | 1.75 | 0,0017 |
| A\_51\_P120066 | AK034052 | hypothetical Myc-type, helix-loop-helix dimerization domain containing protein | 1.75 | 0,0013 |
| A\_51\_P436386 | BC006738 | Mus musculus, clone MGC:7055 IMAGE:3156574, mRNA, complete cds | 1.75 | 0,0022 |
| A\_51\_P479914 | NM\_029094 | PHOSPHATIDYLINOSITOL 3-KINASE CATALYTIC SUBUNIT, BETA ISOFORM (EC 2.7.1.137) (PI3-KINASE P110 SUBUNIT BETA) (PTDINS-3-KINASE P110) (PI3K) (PI3KBETA) homolog [Rattus norvegicus] | 1.75 | 0,0128 |
| A\_51\_P176341 | NM\_028769 | HRD1 homolog [Homo sapiens] | 1.75 | 0,0005 |
| A\_51\_P309889 | 5430410O10 | HYPOTHETICAL 27.0 KDA PROTEIN homolog [Mus musculus] | 1.75 | 0,0040 |
| A\_51\_P473089 | NM\_178647 | P20-CGGBP homolog [Homo sapiens] | 1.75 | 0,0095 |
| A\_51\_P110010 | NM\_027946 | WD REPEAT PROTEIN AN11 | 1.75 | 0,0010 |
| A\_51\_P122290 | AK008125 | Mus musculus RIKEN cDNA 2010005I16 gene (2010005I16Rik), mRNA | 1.75 | 0,0077 |
| A\_51\_P289429 | AK048961 | KPL2 homolog [Rattus norvegicus] | 1.75 | 0,0057 |
| A\_51\_P246816 | NM\_025898 | Mus musculus N-ethylmaleimide sensitive fusion protein attachment protein alpha (Napa), mRNA | 1.75 | 0,0276 |
| A\_51\_P111757 | AK122456 | unknown EST | 1.75 | 0,0030 |
| A\_51\_P112100 | AK081899 | hypothetical protein | 1.75 | 0,0021 |
| A\_51\_P513586 | NM\_025617 | Mus musculus RIKEN cDNA 2210012G02 gene (2210012G02Rik), mRNA | 1.75 | 0,0342 |
| A\_51\_P398868 | NM\_008217 | Mus musculus hyaluronan synthase 3 (Has3), mRNA | 1.75 | 0,0006 |
| A\_51\_P463994 | NM\_021393 | Mus musculus hypothetical protein, MNCb-5210 (AB041607), mRNA | 1.75 | 0,0061 |
| A\_51\_P266546 | NM\_173181 | CGI-62 PROTEIN homolog [Homo sapiens] | 1.75 | 0,0251 |
| A\_51\_P307470 | NM\_028753 | Mus musculus RIKEN cDNA 0610037N12 gene (0610037N12Rik), mRNA | 1.75 | 0,0078 |
| A\_51\_P154469 | NM\_010761 | Mus musculus maternal inhibition of differentiation (Maid), mRNA | 1.75 | 0,0025 |
| A\_51\_P234185 | BC040462 | Mus musculus sprouty protein with EVH-1 domain 2, related sequence (Spred2-pending), mRNA | 1.75 | 0,0072 |
| A\_51\_P290415 | BC046389 | unknown EST | 1.74 | 0,0020 |
| A\_51\_P334619 | NM\_011742 | M.musculus mRNA for Zpf-1 zinc finger protein | 1.74 | 0,0412 |
| A\_51\_P181751 | NM\_207267 | THYMOSIN BETA-LIKE PROTEIN homolog [Rattus norvegicus] | 1.74 | 0,0069 |
| A\_51\_P109671 | NM\_023672 | Mus musculus, hypothetical protein FLJ10355, clone MGC:36847 IMAGE:4208359, mRNA, complete cds | 1.74 | 0,0084 |
| A\_51\_P266723 | NM\_172677 | weakly similar to HIGH-GLUCOSE-REGULATED PROTEIN 8 [Homo sapiens] | 1.74 | 0,0483 |
| A\_51\_P152826 | NM\_025872 | unknown EST | 1.74 | 0,0074 |
| A\_51\_P477030 | NM\_019988 | G protein beta subunit-like | 1.74 | 0,0229 |
| A\_51\_P263302 | NM\_178607 | unknown EST | 1.74 | 0,0149 |
| A\_51\_P405423 | BC094221 | unknown EST | 1.74 | 0,0142 |
| A\_51\_P411694 | BC023699 | Mus musculus, clone IMAGE:4009229, mRNA, partial cds | 1.74 | 0,0182 |
| A\_51\_P226417 | NM\_178376 | RAGA (RAS-RELATED GTP-BINDING PROTEIN) homolog [Homo sapiens] | 1.74 | 0,0083 |
| A\_51\_P482043 | NM\_175266 | hypothetical protein | 1.74 | 0,0273 |
| A\_51\_P236324 | NM\_028812 | general transcription factor IIE, polypeptide 1 (alpha subunit, 56kDa) | 1.74 | 0,0458 |
| A\_51\_P164495 | BC034701 | hypothetical protein | 1.74 | 0,0037 |
| A\_51\_P371743 | NM\_134248 | Mus musculus T-cell immunoglobulin and mucin domain containing 1 (Timd1), mRNA | 1.74 | 0,0220 |
| A\_51\_P108269 | NM\_145609 | CDNA FLJ14884 FIS, CLONE PLACE1003669, WEAKLY SIMILAR TO TRICHOHYALIN homolog [Homo sapiens] | 1.74 | 0,0086 |
| A\_51\_P359018 | NM\_011841 | Mus musculus mitogen-activated protein kinase 7 (Mapk7), mRNA | 1.74 | 0,0255 |
| A\_51\_P472588 | NM\_001015876 | similar to CDNA FLJ12855 FIS, CLONE NT2RP2003506, WEAKLY SIMILAR TO NADPH-CYTOCHROME P450 REDUCTASE (EC 1.6.2.4) [Homo sapiens] | 1.74 | 0,0144 |
| A\_51\_P331525 | NM\_015771 | Mus musculus large tumor suppressor 2 (Lats2), mRNA | 1.74 | 0,0423 |
| A\_51\_P211822 | NM\_001013414 | Mus musculus, Similar to G protein pathway suppressor 2, clone IMAGE:5358633, mRNA, partial cds | 1.74 | 0,0081 |
| A\_51\_P184236 | NM\_026173 | Mus musculus RIKEN cDNA 1200014M14 gene (1200014M14Rik), mRNA | 1.74 | 0,0382 |
| A\_51\_P124647 | AK018169 | hypothetical Aspartic acid-rich region containing protein | 1.74 | 0,0294 |
| A\_51\_P282179 | NM\_020009 | Mus musculus FK506 binding protein 12-rapamycin associated protein 1 (Frap1), mRNA | 1.74 | 0,0021 |
| A\_51\_P286410 | NM\_026872 | Mus musculus, Similar to KIAA0144 gene product, clone MGC:7679 IMAGE:3496608, mRNA, complete cds | 1.74 | 0,0047 |
| A\_51\_P423431 | NM\_027838 | SENTRIN/SUMO-SPECIFIC PROTEASE homolog [Homo sapiens] | 1.74 | 0,0010 |
| A\_51\_P121159 | NM\_023326 | B-MYC TRANSFORMING PROTEIN (FRAGMENT) homolog [Rattus norvegicus] | 1.73 | 0,0128 |
| A\_51\_P284095 | AK076456 | DJ876B10 2 NOVEL PROTEIN ORTHOLOG OF RAT | 1.73 | 0,0092 |
| A\_51\_P219721 | NM\_025942 | hypothetical P-loop containing nucleotide triphosphate hydrolases structure containing protein | 1.73 | 0,0167 |
| A\_51\_P253824 | AK008211 | Mus musculus RIKEN cDNA 2010012L10 gene (2010012L10Rik), mRNA | 1.73 | 0,0037 |
| A\_51\_P486512 | NM\_134093 | Mus musculus expressed sequence AI593524 (AI593524), mRNA | 1.73 | 0,0159 |
| A\_51\_P220278 | NM\_028392 | SERINE/THREONINE PROTEIN PHOSPHATASE 2A, 55 KDA REGULATORY SUBUNIT B, BETA ISOFORM (PP2A, SUBUNIT B, B-BETA ISOFORM) (PP2A, SUBUNIT B, B55- BETA ISOFORM) (PP2A, SUBUNIT B, PR55-BETA ISOFORM) (PP2A, SUBUNIT B, R2-BETA ISOFORM) homolog [Homo sapiens] | 1.73 | 0,0101 |
| A\_51\_P387302 | NM\_029498 | ZINC FINGER PROTEIN 198 (FRAGMENT) | 1.73 | 0,0201 |
| A\_51\_P376641 | A\_51\_P376641 | Mus musculus cDNA, 3 end | 1.73 | 0,0286 |
| A\_51\_P153812 | AK220368 | similar to UBIQUITIN SPECIFIC PROTEASE (FRAGMENT) [Mus musculus] | 1.73 | 0,0079 |
| A\_51\_P497821 | NM\_146185 | ZINC FINGER PROTEIN 30 | 1.73 | 0,0227 |
| A\_51\_P304769 | XM\_287445 | Mus musculus antigen containing epitope to monoclonal antibody MMS-85/12 mRNA, partial cds | 1.73 | 0,0014 |
| A\_51\_P244531 | AK018107 | unknown EST | 1.73 | 0,0248 |
| A\_51\_P213459 | AK046920 | unclassifiable | 1.73 | 0,0062 |
| A\_51\_P291529 | AK077428 | unknown EST | 1.73 | 0,0030 |
| A\_51\_P234466 | XM\_132974 | Mus musculus Abcc10 mRNA, partial sequence | 1.73 | 0,0030 |
| A\_51\_P473576 | NM\_009687 | Mus musculus apurinic/apyrimidinic endonuclease (Apex), mRNA | 1.73 | 0,0200 |
| A\_51\_P370283 | NM\_011869 | Mus musculus thyroid hormone receptor-associated protein 100 kDa (Trap100-pending), mRNA | 1.73 | 0,0023 |
| A\_51\_P324172 | NM\_011245 | Mus musculus RAS protein-specific guanine nucleotide-releasing factor 1 (Rasgrf1), mRNA | 1.73 | 0,0091 |
| A\_51\_P470568 | NM\_023230 | Mus musculus, ubiquitin-conjugating enzyme E2 variant 1, clone MGC:6536 IMAGE:2654197, mRNA, complete cds | 1.73 | 0,0009 |
| A\_51\_P153190 | NM\_133185 | Mus musculus RIKEN cDNA 0610011C19 gene (0610011C19Rik), mRNA | 1.73 | 0,0144 |
| A\_51\_P221008 | AK122465 | Mus musculus cDNA, 5 end | 1.73 | 0,0297 |
| A\_51\_P451837 | AK017353 | unknown EST | 1.72 | 0,0241 |
| A\_51\_P177984 | NM\_207217 | hypothetical Quinoprotein alcohol dehydrogenase structure containing protein | 1.72 | 0,0358 |
| A\_51\_P410650 | NM\_172624 | DIPEPTIDYL PEPTIDASE 9 homolog [Homo sapiens] | 1.72 | 0,0169 |
| A\_51\_P194681 | AK042945 | Mus musculus, Similar to hypothetical protein FLJ11305, clone IMAGE:3590683, mRNA | 1.72 | 0,0080 |
| A\_51\_P243503 | AF037454 | Mus musculus itchy (Itch), mRNA | 1.72 | 0,0065 |
| A\_51\_P520849 | NM\_009144 | Mus musculus secreted frizzled-related sequence protein 2 (Sfrp2), mRNA | 1.72 | 0,0260 |
| A\_51\_P234140 | NM\_183294 | cyclin-dependent kinase-like 1 (CDC2-related kinase) | 1.72 | 0,0466 |
| A\_51\_P319572 | AK053542 | hypothetical protein | 1.72 | 0,0150 |
| A\_51\_P325247 | NM\_011721 | Mus musculus Werner syndrome homolog (human) (Wrn), mRNA | 1.72 | 0,0197 |
| A\_51\_P413315 | X04663 | Mus musculus tubulin, beta 5 (Tubb5), mRNA | 1.72 | 0,0017 |
| A\_51\_P337756 | NM\_026107 | Mus musculus RIKEN cDNA 1700128E15 gene (1700128E15Rik), mRNA | 1.72 | 0,0024 |
| A\_51\_P302405 | AK006462 | hypothetical RNA-binding region RNP-1 (RNA recognition motif) containing protein | 1.72 | 0,0022 |
| A\_51\_P421724 | NM\_012028 | Mus musculus sialyltransferase 7 ((alpha-N-acetylneuraminyl 2,3-betagalactosyl-1,3)-N-acetyl galactosaminide alpha-2,6-sialyltransferase) E (Siat7e), mRNA | 1.72 | 0,0162 |
| A\_51\_P211108 | XM\_283937 | hypothetical protein | 1.72 | 0,0083 |
| A\_51\_P319379 | BC054558 | unknown EST | 1.72 | 0,0152 |
| A\_51\_P398191 | AK028848 | hypothetical Histidine-rich region containing protein | 1.72 | 0,0079 |
| A\_51\_P420415 | BC079863 | unknown | 1.72 | 0,0487 |
| A\_51\_P197321 | NM\_016760 | Mus musculus clathrin, light polypeptide (Lca) (Clta), mRNA | 1.72 | 0,0002 |
| A\_51\_P373149 | NM\_177737 | weakly similar to ZNF74=KRUPPEL-TYPE ZINC FINGER (22 KRUPPEL-RELATED ZINC FINGER PROTEIN) (FRAGMENT) [Homo sapiens] | 1.72 | 0,0003 |
| A\_51\_P242733 | NM\_009357 | Mus musculus testis expressed gene 261 (Tex261), mRNA | 1.72 | 0,0095 |
| A\_51\_P141293 | NM\_175556 | hypothetical Phosphatidylinositol-specific phospholipase C, X domain containing protein | 1.72 | 0,0416 |
| A\_51\_P409468 | XM\_619748 | Mus musculus olfactory receptor MOR128-1 (MOR128-1) pseudogene | 1.72 | 0,0034 |
| A\_51\_P109364 | NM\_175322 | hypothetical protein | 1.72 | 0,0030 |
| A\_51\_P487734 | NM\_027088 | Mus musculus uch-x4 mRNA for ubiquitin C-terminal hydrolase X4, complete cds | 1.72 | 0,0081 |
| A\_51\_P320461 | NM\_011299 | Mus musculus ribosomal protein S6 kinase, 90kD, polypeptide 2 (Rps6ka2), mRNA | 1.72 | 0,0457 |
| A\_51\_P109119 | AK017113 | hypothetical protein | 1.72 | 0,0005 |
| A\_51\_P239156 | NM\_019776 | Mus musculus staphylococcal nuclease domain containing 1 (Snd1-pending), mRNA | 1.72 | 0,0152 |
| A\_51\_P518081 | AK034331 | unknown EST | 1.72 | 0,0005 |
| A\_51\_P384618 | NM\_026144 | Mus musculus RIKEN cDNA 3222401G21 gene (3222401G21Rik), mRNA | 1.72 | 0,0182 |
| A\_51\_P276960 | AK173113 | Mus musculus transcription repressor p66 (LOC229542), mRNA | 1.72 | 0,0154 |
| A\_51\_P340336 | NM\_021568 | Mus musculus poly(rC) binding protein 3 (Pcbp3), mRNA | 1.72 | 0,0045 |
| A\_51\_P300655 | BC082558 | hypothetical protein | 1.72 | 0,0316 |
| A\_51\_P327796 | NM\_010580 | Mus musculus integrin beta 5 (Itgb5), mRNA | 1.72 | 0,0294 |
| A\_51\_P226542 | NM\_173450 | hypothetical Pseudouridine synthase containing protein | 1.72 | 0,0247 |
| A\_51\_P143232 | NM\_176923 | hypothetical Laminin-type EGF-like (LE) domain/Cytochrome c family heme-binding site/G-protein beta WD-40 repeats containing protein | 1.72 | 0,0178 |
| A\_51\_P512172 | AK010953 | hypothetical protein | 1.72 | 0,0021 |
| A\_51\_P191601 | NM\_029841 | hypothetical protein | 1.72 | 0,0121 |
| A\_51\_P411996 | NM\_023483 | Mus musculus RIKEN cDNA 1110032A03 gene (1110032A03Rik), mRNA | 1.72 | 0,0034 |
| A\_51\_P301508 | AK009316 | musculus adult male tongue cDNA, RIKEN full-length enriched library, clone:2310012I02:unclassifiable transcript, full insert sequence | 1.71 | 0,0043 |
| A\_51\_P203771 | NM\_025351 | Mus musculus, Similar to RIKEN cDNA 0710001P09 gene, clone MGC:19393 IMAGE:3153325, mRNA, complete cds | 1.71 | 0,0206 |
| A\_51\_P182752 | NM\_028331 | weakly similar to COMPLEMENT-C1Q TUMOR NECROSIS FACTOR-RELATED PROTEIN [Homo sapiens] | 1.71 | 0,0387 |
| A\_51\_P197773 | BC062096 | hypothetical ADP-ribosylation structure containing protein | 1.71 | 0,0248 |
| A\_51\_P184806 | NM\_178736 | weakly similar to HYPOTHETICAL 23.0 KDA PROTEIN (FRAGMENT) [Homo sapiens] | 1.71 | 0,0048 |
| A\_51\_P228254 | NM\_026647 | Mus musculus RIKEN cDNA 9130404H11 gene (9130404H11Rik), mRNA | 1.71 | 0,0018 |
| A\_51\_P417016 | NM\_013663 | splicing factor, arginine/serine-rich 3 (SRp20) | 1.71 | 0,0084 |
| A\_51\_P487668 | NM\_026230 | Mus musculus RIKEN cDNA 4933432H23 gene (4933432H23Rik), mRNA | 1.71 | 0,0014 |
| A\_51\_P182456 | AK009638 | unknown EST | 1.71 | 0,0189 |
| A\_51\_P298538 | NM\_145404 | ARGININE N-METHYLTRANSFERASE P82 ISOFORM homolog [Cricetulus longicaudatus] | 1.71 | 0,0157 |
| A\_51\_P285763 | NM\_028375 | Mus musculus RIKEN cDNA 2900027G03 gene (2900027G03Rik), mRNA | 1.71 | 0,0119 |
| A\_51\_P383524 | NM\_181728 | NAD(P)(+)--ARGININE ADP-RIBOSYLTRANSFERASE (EC 2.4.2.31) homolog [Mus musculus] | 1.71 | 0,0147 |
| A\_51\_P466221 | NM\_144547 | Mus musculus MIS type II receptor (Misrii) mRNA, complete cds | 1.71 | 0,0010 |
| A\_51\_P129866 | AK082766 | unknown EST | 1.71 | 0,0017 |
| A\_51\_P390586 | NM\_027478 | hypothetical protein | 1.71 | 0,0142 |
| A\_51\_P196523 | NM\_008144 | Mus musculus G protein gamma 3 linked gene (Gng3lg), mRNA | 1.71 | 0,0156 |
| A\_51\_P140742 | NM\_012043 | Mus musculus immunoglobulin superfamily containing leucine-rich repeat (Islr), mRNA | 1.71 | 0,0154 |
| A\_51\_P168756 | NM\_178589 | Mus musculus cDNA, 5 end | 1.71 | 0,0080 |
| A\_51\_P237599 | NM\_009691 | Mus musculus amyloid beta (A4) precursor-like protein 2 (Aplp2), mRNA | 1.71 | 0,0104 |
| A\_51\_P426283 | NM\_133347 | Mus musculus RIKEN cDNA 2810477H02 gene (2810477H02Rik), mRNA | 1.71 | 0,0103 |
| A\_51\_P405682 | AK034740 | hypothetical Trp-Asp repeat (WD-repeat) structure containing protein | 1.71 | 0,0024 |
| A\_51\_P351697 | NM\_025430 | Mus musculus RIKEN cDNA 1110066C01 gene (1110066C01Rik), mRNA | 1.71 | 0,0002 |
| A\_51\_P172692 | NM\_017478 | Mus musculus coatomer protein complex, subunit gamma 2 (Copg2), mRNA | 1.71 | 0,0387 |
| A\_51\_P464398 | AK046394 | SERINE/THREONINE KINASE NKIATRE ALPHA homolog [Rattus norvegicus] | 1.71 | 0,0063 |
| A\_51\_P401876 | NM\_028197 | unknown EST | 1.71 | 0,0226 |
| A\_51\_P490867 | AK173048 | unclassifiable | 1.71 | 0,0313 |
| A\_51\_P435239 | NM\_010026 | Mus musculus ADP-ribosylation factor-directed GTPase activating protein isoform a (Shag1) mRNA, complete cds | 1.71 | 0,0023 |
| A\_51\_P225048 | AJ250693 | SR528 PROTEIN (FRAGMENT) homolog [Mus musculus] | 1.71 | 0,0015 |
| A\_51\_P165098 | NM\_028758 | similar to ADP-RIBOSYLATION FACTOR BINDING PROTEIN GGA2 (GOLGI-LOCALIZED, GAMMA EAR-CONTAINING, ARF-BINDING PROTEIN 2) (GAMMA-ADAPTIN RELATED PROTEIN 2) (VEAR) (VHS DOMAIN AND EAR DOMAIN OF GAMMA-ADAPTIN) [Homo sapiens] | 1.71 | 0,0022 |
| A\_51\_P247478 | NM\_028105 | Mus musculus, RIKEN cDNA 2610005A10 gene, clone MGC:19303 IMAGE:4162365, mRNA, complete cds | 1.69 | 0,0037 |
| A\_51\_P245233 | NM\_175134 | hypothetical Ankyrin repeat profile/Ankyrin-repeat/Ankyrin repeat region circular profile/Yeast DNA-binding domain containing protein | 1.69 | 0,0010 |
| A\_51\_P204366 | NM\_001001983 | Mus musculus, Similar to phosphatidylinositol 4-kinase, clone IMAGE:5363136, mRNA | 1.69 | 0,0057 |
| A\_51\_P240864 | NM\_029012 | Mus musculus, clone MGC:29009 IMAGE:3154534, mRNA, complete cds | 1.69 | 0,0111 |
| A\_51\_P514300 | NM\_009447 | Mus musculus tubulin, alpha 4 (Tuba4), mRNA | 1.69 | 0,0207 |
| A\_51\_P454234 | BC057621 | hypothetical Vacuolar sorting protein 9 (VPS9) domain containing protein | 1.69 | 0,0351 |
| A\_51\_P363556 | NM\_198127 | unknown EST | 1.69 | 0,0311 |
| A\_51\_P284966 | NM\_177648 | HYPOTHETICAL PROTEIN KIAA1094 homolog [Homo sapiens] | 1.69 | 0,0109 |
| A\_51\_P437068 | NM\_031396 | Mus musculus cyclin M1 (Cnnm1), mRNA | 1.69 | 0,0223 |
| A\_51\_P433506 | NM\_130866 | Mus musculus olfactory receptor 78 (Olfr78), mRNA | 1.69 | 0,0222 |
| A\_51\_P370960 | NM\_027815 | ESOPHAGEAL CANCER ASSOCIATED PROTEIN homolog [Homo sapiens] | 1.69 | 0,0217 |
| A\_51\_P109050 | NM\_023824 | Mus musculus RIKEN cDNA 1500004C10 gene (1500004C10Rik), mRNA | 1.69 | 0,0137 |
| A\_51\_P338461 | NM\_133947 | Mus musculus, clone IMAGE:3498604, mRNA, partial cds | 1.69 | 0,0052 |
| A\_51\_P393310 | NM\_198034 | weakly similar to CGI-40 PROTEIN [Homo sapiens] | 1.69 | 0,0360 |
| A\_51\_P221535 | NM\_175287 | hypothetical protein | 1.69 | 0,0472 |
| A\_51\_P114136 | NM\_177294 | hypothetical protein | 1.69 | 0,0058 |
| A\_51\_P396852 | NM\_026619 | similar to BA127L20.1 (NOVEL GLUTATHIONE-S-TRANSFERASE) [Homo sapiens] | 1.69 | 0,0435 |
| A\_51\_P329278 | AK078133 | unknown EST | 1.69 | 0,0021 |
| A\_51\_P189251 | NM\_013823 | Mus musculus klotho (Kl), mRNA | 1.69 | 0,0461 |
| A\_51\_P340038 | AK032598 | hypothetical Phosphotyrosine interaction (PID or PI) containing protein | 1.69 | 0,0254 |
| A\_51\_P271425 | AK220449 | unknown EST | 1.69 | 0,0188 |
| A\_51\_P517834 | AK012806 | hypothetical S-adenosyl-L-methionine-dependent methyltransferases structure containing protein | 1.69 | 0,0087 |
| A\_51\_P217899 | NM\_146163 | Mus musculus myosin IH (Myo1h), mRNA | 1.69 | 0,0277 |
| A\_51\_P301848 | NM\_133345 | Mus musculus DNA segment, Chr 6, Wayne State University 147, expressed (D6Wsu147e), mRNA | 1.69 | 0,0135 |
| A\_51\_P234833 | NM\_133789 | Mus musculus, clone IMAGE:3494615, mRNA, partial cds | 1.68 | 0,0200 |
| A\_51\_P366344 | NM\_009365 | Mus musculus transforming growth factor beta 1 induced transcript 1 (Tgfb1i1), mRNA | 1.68 | 0,0377 |
| A\_51\_P417025 | NM\_013837 | Mus musculus protein-tyrosine sulfotransferase 1 (Tpst1), mRNA | 1.68 | 0,0125 |
| A\_51\_P275658 | NM\_016874 | Mus musculus deformed epidermal autoregulatory factor 1 (Drosophila) (Deaf1), mRNA | 1.68 | 0,0008 |
| A\_51\_P155152 | NM\_020332 | Mus musculus progressive ankylosis (ank), mRNA | 1.68 | 0,0343 |
| A\_51\_P518219 | AB041543 | Mus musculus expressed sequence AI448780 (AI448780), mRNA | 1.68 | 0,0110 |
| A\_51\_P220163 | NM\_008716 | Mus musculus Notch gene homolog 3, (Drosophila) (Notch3), mRNA | 1.68 | 0,0411 |
| A\_51\_P430585 | NM\_026740 | CDNA FLJ30107 FIS, CLONE BNGH41000198, WEAKLY SIMILAR TO TETRACYCLINE RESISTANCE PROTEIN, CLASS E homolog [Homo sapiens] | 1.68 | 0,0017 |
| A\_51\_P516016 | NM\_011473 | Mus musculus small proline-rich protein 2G (Sprr2g), mRNA | 1.68 | 0,0487 |
| A\_51\_P328652 | NM\_133348 | Mus musculus brain acyl-CoA hydrolase (Bach-pending), mRNA | 1.68 | 0,0071 |
| A\_51\_P381150 | AK129097 | Mus musculus cDNA clone IMAGE:891063 3. | 1.68 | 0,0138 |
| A\_51\_P119776 | AK004418 | unknown EST | 1.68 | 0,0087 |
| A\_51\_P417053 | NM\_011656 | Mus musculus tuftelin 1 (Tuft1), mRNA | 1.68 | 0,0426 |
| A\_51\_P352850 | NM\_007909 | Mus musculus ephrin A2 (Efna2), mRNA | 1.68 | 0,0171 |
| A\_51\_P176083 | NM\_146187 | Mus musculus, Similar to G protein-coupled receptor 43, clone MGC:28611 IMAGE:4218874, mRNA, complete cds | 1.68 | 0,0161 |
| A\_51\_P367578 | AK079162 | FETAL ALZHEIMER ANTIGEN FETAL ALZ 50 REACTIVE CLONE | 1.68 | 0,0041 |
| A\_51\_P491835 | AK013507 | inferred: RIKEN cDNA 2900009J20 gene | 1.68 | 0,0350 |
| A\_51\_P239984 | NM\_012012 | Mus musculus exonuclease 1 (Exo1), mRNA | 1.68 | 0,0017 |
| A\_51\_P138830 | NM\_198108 | Mus musculus similar to GH04877p [Drosophila melanogaster] (LOC226123), mRNA | 1.68 | 0,0115 |
| A\_51\_P118539 | AK012399 | hypothetical protein | 1.68 | 0,0151 |
| A\_51\_P370614 | NM\_009718 | Mus musculus neurogenin 2 (Neurog2), mRNA | 1.68 | 0,0201 |
| A\_51\_P483089 | NM\_010411 | Mus musculus histone deacetylase 3 (Hdac3), mRNA | 1.68 | 0,0013 |
| A\_51\_P220934 | NM\_026955 | hypothetical Immunoglobulin subtype containing protein | 1.68 | 0,0016 |
| A\_51\_P359800 | NM\_009131 | Mus musculus stem cell growth factor (Scgf), mRNA | 1.68 | 0,0060 |
| A\_51\_P268033 | BC026638 | Mus musculus, Similar to mannosyl (alpha-1,3-)-glycoprotein beta-1,4-N-acetylglucosaminyltransferase, isoenzyme B, clone MGC:37502 IMAGE:4984907, mRNA, complete cds | 1.68 | 0,0349 |
| A\_51\_P336952 | NM\_030004 | Mus musculus crystallin, lamda 1 (Cryl1), mRNA | 1.68 | 0,0014 |
| A\_51\_P115988 | NM\_024480 | Mus musculus hypothetical protein, MGC:7813 (BC003251), mRNA | 1.68 | 0,0020 |
| A\_51\_P363210 | XM\_485743 | hypothetical protein | 1.68 | 0,0419 |
| A\_51\_P172811 | AK045397 | NULP1 homolog [Mus musculus] | 1.68 | 0,0337 |
| A\_51\_P268274 | NM\_018872 | Mus musculus RW1 protein (Rw1-pending), mRNA | 1.68 | 0,0056 |
| A\_51\_P212518 | NM\_178726 | CDNA FLJ30553 FIS, CLONE BRAWH2003689, HIGHLY SIMILAR TO MUS MUSCULUS CLONE MOUSE1-9 PUTATIVE PROTEIN PHOSPHATASE TYPE 2C MRNA homolog [Homo sapiens] | 1.68 | 0,0497 |
| A\_51\_P314893 | NM\_146014 | Mus musculus, Similar to hypothetical protein MGC4607, clone MGC:37115 IMAGE:4952288, mRNA, complete cds | 1.68 | 0,0146 |
| A\_51\_P389908 | NM\_013881 | Mus musculus Unc-51 like kinase 2 (C. elegans) (Ulk2), mRNA | 1.68 | 0,0197 |
| A\_51\_P174854 | NM\_019791 | Mus musculus melanoma antigen, family D, 1 (Maged1), mRNA | 1.67 | 0,0331 |
| A\_51\_P227502 | NM\_008546 | Mus musculus microfibrillar-associated protein 2 (Mfap2), mRNA | 1.67 | 0,0011 |
| A\_51\_P383369 | NM\_011857 | Mus musculus odd Oz/ten-m homolog 3 (Drosophila) (Odz3), mRNA | 1.67 | 0,0443 |
| A\_51\_P161413 | XM\_149655 | hypothetical Ubiquitin carboxyl-terminal hydrolase family 2 containing protein | 1.67 | 0,0031 |
| A\_51\_P368074 | NM\_028132 | Mus musculus, RIKEN cDNA 2610020G18 gene, clone MGC:6642 IMAGE:3495694, mRNA, complete cds | 1.67 | 0,0136 |
| A\_51\_P188605 | NM\_198424 | similar to SIMILAR TO CAP-BINDING PROTEIN COMPLEX INTERACTING PROTEIN 2 [Homo sapiens] | 1.67 | 0,0002 |
| A\_51\_P281575 | NM\_029985 | Mus musculus, RIKEN cDNA A930011F22 gene, clone MGC:12118 IMAGE:3710091, mRNA, complete cds | 1.67 | 0,0027 |
| A\_51\_P363800 | NM\_023217 | Mus musculus RIKEN cDNA 2810003H13 gene (2810003H13Rik), mRNA | 1.67 | 0,0314 |
| A\_51\_P222283 | NM\_019966 | malonyl-CoA decarboxylase | 1.67 | 0,0104 |
| A\_51\_P310447 | BC079880 | Mus musculus, RIKEN cDNA 2310066E14 gene, clone MGC:11838 IMAGE:3596826, mRNA, complete cds | 1.67 | 0,0081 |
| A\_51\_P366306 | NM\_028325 | hypothetical Zn-finger CCHC type containing protein | 1.67 | 0,0283 |
| A\_51\_P102225 | NM\_011543 | Mus musculus transcription elongation factor B (SIII), polypeptide 1 (15 kDa),-like (Tceb1l), mRNA | 1.67 | 0,0226 |
| A\_51\_P150132 | NM\_025299 | Mus musculus dim1 (S. pombe) (Dim1-pending), mRNA | 1.67 | 0,0004 |
| A\_51\_P466371 | NM\_008850 | Mus musculus phosphatidylinositol transfer protein (Pitpn), mRNA | 1.67 | 0,0191 |
| A\_51\_P450536 | NM\_028170 | Mus musculus, clone MGC:8256 IMAGE:3592293, mRNA, complete cds | 1.67 | 0,0075 |
| A\_51\_P115891 | NM\_026655 | hypothetical protein | 1.67 | 0,0007 |
| A\_51\_P449911 | BC037217 | unclassifiable | 1.67 | 0,0188 |
| A\_51\_P391074 | NM\_007386 | M.musculus mRNA for iron responsive element binding protein | 1.67 | 0,0162 |
| A\_51\_P144348 | A\_51\_P144348 | Mus musculus cDNA, 5 end | 1.67 | 0,0411 |
| A\_51\_P219505 | NM\_177388 | hypothetical Divalent cation transporter containing protein | 1.67 | 0,0192 |
| A\_51\_P187841 | NM\_025829 | Mus musculus RIKEN cDNA 1300018P11 gene (1300018P11Rik), mRNA | 1.67 | 0,0212 |
| A\_51\_P303238 | BC006774 | Mus musculus, Similar to transmembrane trafficking protein, clone MGC:8106 IMAGE:3588721, mRNA, complete cds | 1.67 | 0,0011 |
| A\_51\_P502443 | BC085617 | similar to ADP-RIBOSYLATION FACTOR BINDING PROTEIN GGA3 (GOLGI-LOCALIZED, GAMMA EAR-CONTAINING, ARF-BINDING PROTEIN 3) [Homo sapiens] | 1.66 | 0,0423 |
| A\_51\_P490924 | NM\_177900 | CORE PROTEIN PRECURSOR PROTEOGLYCAN CORE PROTEIN | 1.66 | 0,0195 |
| A\_51\_P476129 | NM\_029546 | PERIODIC TRYPTOPHAN PROTEIN 2 HOMOLOG homolog [Homo sapiens] | 1.66 | 0,0090 |
| A\_51\_P458998 | BC016258 | Mus musculus, Similar to RIKEN cDNA 1200011A11 gene, clone IMAGE:4913359, mRNA, partial cds | 1.66 | 0,0051 |
| A\_51\_P167132 | NM\_019461 | Mus musculus ubiquitin specific protease (Uspx) mRNA, partial cds | 1.66 | 0,0260 |
| A\_51\_P173785 | NM\_009620 | Mus musculus ADAM 4 protein precursor (ADAM 4) mRNA, partial cds | 1.66 | 0,0106 |
| A\_51\_P499755 | NM\_009672 | Mus musculus acidic nuclear phosphoprotein 32 (Anp32), mRNA | 1.66 | 0,0146 |
| A\_51\_P223111 | NM\_028661 | hypothetical protein | 1.66 | 0,0020 |
| A\_51\_P253310 | NM\_019445 | Mus musculus formin 2 (Fmn2), mRNA | 1.66 | 0,0009 |
| A\_51\_P194306 | NM\_172528 | Mus musculus, Similar to LAP (leucine-rich repeats and PDZ) and no PDZ protein, clone IMAGE:4167448, mRNA, partial cds | 1.66 | 0,0066 |
| A\_51\_P482233 | XM\_484158 | unknown EST | 1.66 | 0,0362 |
| A\_51\_P384639 | NM\_026154 | Mus musculus mitochondrial ribosomal protein L10 (Mrpl10), mRNA | 1.66 | 0,0009 |
| A\_51\_P511734 | NM\_175246 | DJ423B22.2 (NOVEL PROTEIN SIMILAR TO C.ELEGANS PROTEIN CE08529) (FRAGMENT) homolog [Homo sapiens] | 1.66 | 0,0424 |
| A\_51\_P277321 | NM\_146085 | Mus musculus, Similar to amyloid beta (A4) precursor protein-binding, family B, member 3, clone MGC:38710 IMAGE:5357681, mRNA, complete cds | 1.66 | 0,0018 |
| A\_51\_P397154 | NAP063254-1 | Mus musculus similar to Oxysterols receptor LXR-beta (Liver X receptor beta) | 1.66 | 0,0474 |
| A\_51\_P168768 | A\_51\_P168768 | Mus musculus cDNA, 3 end | 1.66 | 0,0211 |
| A\_51\_P109171 | NM\_177614 | Mus musculus, clone IMAGE:3598453, mRNA, partial cds | 1.66 | 0,0066 |
| A\_51\_P332228 | NM\_199024 | similar to NOLP PROTEIN (HRIHFB2255 PROTEIN) [Homo sapiens] | 1.66 | 0,0338 |
| A\_51\_P185103 | NM\_024428 | DPY-30-LIKE PROTEIN homolog [Mus musculus] | 1.66 | 0,0025 |
| A\_51\_P111259 | NM\_026373 | Mus musculus RIKEN cDNA 5830466O21 gene (5830466O21Rik), mRNA | 1.66 | 0,0264 |
| A\_51\_P294778 | NM\_172738 | similar to ZINC FINGER PROTEIN 12 (FRAGMENT) [Rattus norvegicus] | 1.66 | 0,0030 |
| A\_51\_P105755 | AK011730 | weakly similar to BA162G10.3 (ZINC FINGER PROTEIN) (FRAGMENT) [Homo sapiens] | 1.66 | 0,0115 |
| A\_51\_P223887 | AK011097 | 4930506L13RIK PROTEIN homolog [Mus musculus] | 1.66 | 0,0039 |
| A\_51\_P385598 | NM\_008063 | Mus musculus glucose-6-phosphatase, transport protein 1 (G6pt1), mRNA | 1.66 | 0,0189 |
| A\_51\_P307747 | BC052056 | Mus musculus T2-cadherin mRNA, partial cds | 1.66 | 0,0392 |
| A\_51\_P268824 | NM\_172659 | similar to SOLUTE CARRIER FAMILY 2, FACILITATED GLUCOSE TRANSPORTER, MEMBER 6 (GLUCOSE TRANSPORTER TYPE 6) (GLUCOSE TRANSPORTER TYPE 9) [Homo sapiens] | 1.65 | 0,0050 |
| A\_51\_P501396 | NM\_021450 | Mus musculus transient receptor potential cation channel, subfamily M, member 7 (Trpm7), mRNA | 1.65 | 0,0128 |
| A\_51\_P431669 | NM\_178113 | hypothetical protein | 1.65 | 0,0234 |
| A\_51\_P354792 | NM\_008521 | Mus musculus leukotriene C4 synthase (Ltc4s), mRNA | 1.65 | 0,0013 |
| A\_51\_P469545 | NM\_020035 | Mus musculus phosphatidylinositol glycan, class O (Pigo), mRNA | 1.65 | 0,0115 |
| A\_51\_P323930 | BC025640 | Mus musculus, similar to Unknown (protein for IMAGE:3627860), clone IMAGE:5250918, mRNA | 1.65 | 0,0199 |
| A\_51\_P182055 | NM\_016877 | Mus musculus CCR4-NOT transcription complex, subunit 4 (Cnot4), mRNA | 1.65 | 0,0472 |
| A\_51\_P104747 | NM\_010364 | Mus musculus general transcription factor II H, polypeptide 4 (Gtf2h4), mRNA | 1.65 | 0,0083 |
| A\_51\_P277445 | NM\_013923 | Mus musculus ring finger protein (C3HC4 type) 19 (Rnf19), mRNA | 1.65 | 0,0044 |
| A\_51\_P368151 | BC016616 | hypothetical Myb DNA binding domain containing protein | 1.65 | 0,0197 |
| A\_51\_P242265 | NM\_009950 | Mus musculus CASP2 and RIPK1 domain containing adaptor with death domain (Cradd), mRNA | 1.65 | 0,0092 |
| A\_51\_P492707 | NM\_011216 | Mus musculus protein tyrosine phosphatase, receptor type, O (Ptpro), mRNA | 1.65 | 0,0181 |
| A\_51\_P340704 | NM\_027328 | Mus musculus RIKEN cDNA 1500019O16 gene (1500019O16Rik), mRNA | 1.65 | 0,0317 |
| A\_51\_P297480 | NM\_144491 | Mus musculus diptheria toxin resistance protein required for diphthamide biosynthesis (Saccharomyces)-like 1 (Dph2l1), mRNA | 1.65 | 0,0181 |
| A\_51\_P393064 | NM\_021392 | adaptor-related protein complex AP-4, mu 1 | 1.65 | 0,0054 |
| A\_51\_P253359 | NM\_025670 | Mus musculus RIKEN cDNA 5730403B10 gene (5730403B10Rik), mRNA | 1.65 | 0,0055 |
| A\_51\_P336161 | NM\_010764 | Mus musculus mannosidase 2, alpha B1 (Man2b1), mRNA | 1.65 | 0,0002 |
| A\_51\_P433989 | NM\_009158 | Mus musculus mitogen activated protein kinase 10 (Mapk10), mRNA | 1.65 | 0,0206 |
| A\_51\_P488937 | NM\_011182 | Mus musculus pleckstrin homology, Sec7 and coiled/coil domains 3 (Pscd3), mRNA | 1.65 | 0,0024 |
| A\_51\_P381778 | AK011294 | Mus musculus RIKEN cDNA 2610002K22 gene (2610002K22Rik), mRNA | 1.65 | 0,0117 |
| A\_51\_P188614 | NM\_011853 | Mouse mRNA for 2-5A synthetase | 1.65 | 0,0044 |
| A\_51\_P136848 | AK050977 | unclassifiable | 1.65 | 0,0169 |
| A\_51\_P376057 | NM\_178782 | hypothetical Ankyrin-repeat containing protein | 1.65 | 0,0013 |
| A\_51\_P183822 | AK014401 | unknown EST | 1.65 | 0,0395 |
| A\_51\_P399477 | AK079183 | unknown EST | 1.64 | 0,0288 |
| A\_51\_P207403 | NM\_133993 | Mus musculus RIKEN cDNA 2310058A11 gene (2310058A11Rik), mRNA | 1.64 | 0,0008 |
| A\_51\_P489821 | BC058274 | Mus musculus RIKEN cDNA 2610509D04 gene (2610509D04Rik), mRNA | 1.64 | 0,0078 |
| A\_51\_P160413 | NM\_178691 | hypothetical protein | 1.64 | 0,0128 |
| A\_51\_P106000 | AK049629 | similar to KIAA1211 PROTEIN (FRAGMENT) [Homo sapiens] | 1.64 | 0,0470 |
| A\_51\_P340854 | NM\_008436 | Mus musculus K+ voltage-gated channel, subfamily S, 2 (Kcns2), mRNA | 1.64 | 0,0013 |
| A\_51\_P184223 | NM\_053132 | Mus musculus protocadherin beta 7 (Pcdhb7), mRNA | 1.64 | 0,0022 |
| A\_51\_P266043 | NM\_153459 | Mus musculus dual specificity phosphatase 7 (Dusp7), mRNA | 1.64 | 0,0292 |
| A\_51\_P198387 | NM\_026765 | hypothetical PRTase-like structure containing protein | 1.64 | 0,0176 |
| A\_51\_P272363 | NM\_134160 | Mus musculus mucolipin-3 (Mcoln3), mRNA | 1.64 | 0,0397 |
| A\_51\_P452109 | AK009711 | Mus musculus RIKEN cDNA 2310040A13 gene (2310040A13Rik), mRNA | 1.64 | 0,0141 |
| A\_51\_P155574 | NM\_008989 | Mus musculus purine rich element binding protein A (Pura), mRNA | 1.64 | 0,0107 |
| A\_51\_P174906 | BC029621 | PHOSPHORIBOSYLPYROPHOSPHATE SYNTHETASE-ASSOCIATED PROTEIN (39 KDA) (PHOSPHORIBOSYLPYROPHOSPHATE SYNTHETASE-ASSOCIATED PROTEIN 39) homolog [Rattus norvegicus] | 1.64 | 0,0042 |
| A\_51\_P256285 | NM\_024209 | Mus musculus, Similar to protein phosphatase 6, catalytic subunit, clone MGC:7488 IMAGE:3491111, mRNA, complete cds | 1.64 | 0,0020 |
| A\_51\_P498631 | NM\_018769 | Mus musculus deafness, autosomal dominant 5 homolog (human) (Dfna5h), mRNA | 1.64 | 0,0136 |
| A\_51\_P495049 | NM\_009897 | Mus musculus creatine kinase, mitochondrial 1, ubiquitous (Ckmt1), mRNA | 1.64 | 0,0486 |
| A\_51\_P515901 | AK009928 | unknown EST | 1.64 | 0,0427 |
| A\_51\_P351217 | NM\_175562 | RAS-RELATED PROTEIN RAB-39 homolog [Homo sapiens] | 1.64 | 0,0137 |
| A\_51\_P236412 | NM\_016912 | Mus musculus cyclin-dependent kinase-like 2 (CDC2-related kinase) (Cdkl2), mRNA | 1.64 | 0,0216 |
| A\_51\_P141521 | NM\_172965 | CDNA FLJ31417 FIS, CLONE NT2NE2000327, WEAKLY SIMILAR TO GLUCOAMYLASE S1/S2 PRECURSOR (EC 3.2.1.3) (FRAGMENT) homolog [Homo sapiens] | 1.64 | 0,0079 |
| A\_51\_P447248 | NM\_175105 | hypothetical Eukaryotic thiol (cysteine) proteases active site containing protein | 1.64 | 0,0298 |
| A\_51\_P275273 | NM\_010575 | Mus musculus integrin alpha 2b (Itga2b), mRNA | 1.64 | 0,0280 |
| A\_51\_P153977 | NM\_198322 | ZFP71P (FRAGMENT) homolog [Mus musculus] | 1.64 | 0,0087 |
| A\_51\_P275516 | NM\_012027 | Mus musculus p116Rip mRNA, complete cds | 1.64 | 0,0106 |
| A\_51\_P327021 | NM\_028356 | weakly similar to ZINC FINGER PROTEIN 46 (ZINC FINGER PROTEIN KUP) [Homo sapiens] | 1.64 | 0,0055 |
| A\_51\_P221132 | NM\_145443 | Mus musculus, Similar to hypothetical protein FLJ12618, clone MGC:28775 IMAGE:4487011, mRNA, complete cds | 1.64 | 0,0201 |
| A\_51\_P393193 | NM\_144511 | Mus musculus esterase 31 (Es31), mRNA | 1.64 | 0,0002 |
| A\_51\_P172532 | NM\_012049 | Mus musculus nitrilase 1 (Nit1), mRNA | 1.64 | 0,0022 |
| A\_51\_P245102 | NM\_026596 | Mus musculus RIKEN cDNA 4930591A17 gene (4930591A17Rik), mRNA | 1.64 | 0,0014 |
| A\_51\_P137578 | NM\_175461 | hypothetical protein | 1.64 | 0,0041 |
| A\_51\_P101719 | NM\_025978 | Mus musculus RIKEN cDNA 2700016E08 gene (2700016E08Rik), mRNA | 1.64 | 0,0155 |
| A\_51\_P105869 | NM\_028141 | similar to ZINC FINGER 2.2 (FRAGMENT) [Homo sapiens] | 1.64 | 0,0074 |
| A\_51\_P461219 | NM\_178693 | similar to CGI-92 PROTEIN [Homo sapiens] | 1.64 | 0,0030 |
| A\_51\_P123805 | XM\_355637 | Mus musculus, Similar to strawberry notch, clone IMAGE:3597470, mRNA | 1.64 | 0,0128 |
| A\_51\_P503194 | NM\_175402 | PUTATIVE RNA BINDING PROTEIN 15 RNA BINDING MOTIF PROTEIN 15 ONE TWENTY TWO | 1.64 | 0,0357 |
| A\_51\_P193302 | NM\_025305 | mitchondrial ribosomal protein S7 | 1.64 | 0,0127 |
| A\_51\_P252154 | NM\_201226 | hypothetical B3/B4 domain of PheRS, PheT structure containing protein | 1.64 | 0,0254 |
| A\_51\_P147056 | BF138649 | ubiquitin-like 4 | 1.64 | 0,0290 |
| A\_51\_P128786 | NM\_029425 | DJ846F13.1 (PHOSPHATIDIC ACID PHOSPHATASE TYPE 2C) (FRAGMENT) homolog [Homo sapiens] | 1.64 | 0,0093 |
| A\_51\_P442264 | NM\_172620 | ARE1 homolog [Rattus norvegicus] | 1.62 | 0,0073 |
| A\_51\_P483258 | NM\_145145 | inferred: protein O-mannosyltransferase 1 {Rattus norvegicus} | 1.62 | 0,0118 |
| A\_51\_P354683 | AK016104 | Mus musculus RIKEN cDNA 4930553F24 gene (4930553F24Rik), mRNA | 1.62 | 0,0253 |
| A\_51\_P496590 | NM\_146099 | hypothetical Proline-rich region/Cytochrome c family heme-binding site containing protein | 1.62 | 0,0069 |
| A\_51\_P124039 | NM\_175294 | NUCLEAR UBIQUITOUS CASEIN AND CYCLIN-DEPENDENT KINASES SUBSTRATE homolog [Rattus norvegicus] | 1.62 | 0,0050 |
| A\_51\_P346803 | NM\_017380 | Mus musculus septin 9 (Sept9), mRNA | 1.62 | 0,0081 |
| A\_51\_P285770 | NM\_010192 | feminization 1 homolog a (C. elegans) | 1.62 | 0,0023 |
| A\_51\_P157255 | NM\_008304 | Mus musculus syndecan 2 (Sdc2), mRNA | 1.62 | 0,0223 |
| A\_51\_P487062 | AK029628 | Mus musculus olfactory receptor MOR124-1 pseudogene, partial sequence | 1.62 | 0,0007 |
| A\_51\_P491595 | NM\_145930 | Mus musculus, clone MGC:12113 IMAGE:3709876, mRNA, complete cds | 1.62 | 0,0203 |
| A\_51\_P348397 | NM\_138753 | Mus musculus cardiac lineage protein 1 (Clp1), mRNA | 1.62 | 0,0022 |
| A\_51\_P272584 | NM\_027727 | hypothetical ARM repeat structure containing protein | 1.62 | 0,0052 |
| A\_51\_P207921 | NM\_008898 | Mus musculus P450 (cytochrome) oxidoreductase (Por), mRNA | 1.62 | 0,0066 |
| A\_51\_P394471 | NM\_011548 | transcription factor E2a | 1.62 | 0,0010 |
| A\_51\_P150430 | NM\_023290 | Mus musculus makorin, ring finger protein, 2 (Mkrn2), mRNA | 1.62 | 0,0092 |
| A\_51\_P517787 | NM\_146047 | CISPLATIN RESISTANCE RELATED PROTEIN CRR9P homolog [Homo sapiens] | 1.62 | 0,0024 |
| A\_51\_P358171 | NM\_019488 | Mus musculus solute carrier family 2, (facilitated glucose transporter), member 8 (Slc2a8), mRNA | 1.62 | 0,0412 |
| A\_51\_P222033 | NM\_178070 | VACUOLAR PROTEIN SORTING HOMOLOG R-VPS33B homolog [Rattus norvegicus] | 1.62 | 0,0025 |
| A\_51\_P355622 | NM\_008087 | Mus musculus growth arrest specific 2 (Gas2), mRNA | 1.62 | 0,0233 |
| A\_51\_P367772 | AK002680 | RIKEN cDNA 0610027A18 gene | 1.62 | 0,0099 |
| A\_51\_P383628 | NM\_126165 | Mus musculus vacuolar protein sorting protein 4a (Vps4a), mRNA | 1.62 | 0,0002 |
| A\_51\_P242356 | NM\_026342 | hypothetical protein | 1.62 | 0,0037 |
| A\_51\_P518576 | NM\_145919 | SIMILAR TO DKFZP564O243 PROTEIN homolog [Mus musculus] | 1.62 | 0,0003 |
| A\_51\_P141502 | NM\_026623 | inferred: RIKEN cDNA 5730530J16 gene | 1.62 | 0,0072 |
| A\_51\_P189905 | NM\_010015 | Mus musculus defender against cell death 1 (Dad1), mRNA | 1.62 | 0,0085 |
| A\_51\_P451292 | NM\_028806 | musculus adult male cerebellum cDNA, RIKEN full-length enriched library, clone:1500003N10:homolog to DJ899C14.1 (A NOVEL PROTEIN) (FRAGMENT), full insert sequence | 1.62 | 0,0466 |
| A\_51\_P312360 | NM\_023058 | Mus musculus membrane-associated tyrosine-and threonine-specific cdc2-inhibitory kinase (Pkmyt1-pending), mRNA | 1.62 | 0,0075 |
| A\_51\_P436717 | NM\_011431 | Mus musculus U5 small nuclear ribonucleoprotein 116 kDa (Snrp116-pending), mRNA | 1.62 | 0,0263 |
| A\_51\_P444874 | NM\_008702 | Mus musculus nemo like kinase (Nlk), mRNA | 1.62 | 0,0077 |
| A\_51\_P106373 | NM\_025321 | Mus musculus RIKEN cDNA 0610010E03 gene (0610010E03Rik), mRNA | 1.61 | 0,0064 |
| A\_51\_P516078 | NM\_146067 | similar to CDNA FLJ11151 FIS, CLONE PLACE1006883 [Homo sapiens] | 1.61 | 0,0068 |
| A\_51\_P472329 | NM\_028883 | weakly similar to KIAA1609 PROTEIN (FRAGMENT) [Homo sapiens] | 1.61 | 0,0041 |
| A\_51\_P484918 | NM\_172551 | DNA DIRECTED RNA POLYMERASE, MITOCHONDRIAL PRECURSOR EC 2.7.7.6 | 1.61 | 0,0005 |
| A\_51\_P499482 | BC058277 | Mus musculus aldehyde dehydrogenase family 1, subfamily A3 (Aldh1a3), mRNA | 1.61 | 0,0453 |
| A\_51\_P403881 | NM\_011574 | M.musculus tex292 mRNA (3region) | 1.61 | 0,0386 |
| A\_51\_P105008 | NM\_027853 | Mus musculus RIKEN cDNA 0610006F02 gene (0610006F02Rik), mRNA | 1.61 | 0,0070 |
| A\_51\_P392636 | NM\_009420 | Mus musculus testis specific gene 1 (Tpx1), mRNA | 1.61 | 0,0045 |
| A\_51\_P488554 | NM\_026543 | Mus musculus RIKEN cDNA 3010026O09 gene (3010026O09Rik), mRNA | 1.61 | 0,0407 |
| A\_51\_P467971 | NM\_170728 | Mus musculus ankyrin 3, epithelial (Ank3), mRNA | 1.61 | 0,0094 |
| A\_51\_P132592 | NM\_029998 | protein phosphatase 1, regulatory (inhibitor) subunit 7 | 1.61 | 0,0397 |
| A\_51\_P369623 | NM\_133949 | Mus musculus prostate tumor over expressed gene 1 (Ptov1), mRNA | 1.61 | 0,0440 |
| A\_51\_P209460 | NM\_026585 | hypothetical protein | 1.61 | 0,0006 |
| A\_51\_P214882 | NM\_021434 | Mus musculus hypothetical protein, MNCb-3029 (AB041545), mRNA | 1.61 | 0,0181 |
| A\_51\_P492366 | NM\_007557 | Mus musculus bone morphogenetic protein 7 (Bmp7), mRNA | 1.61 | 0,0100 |
| A\_51\_P344661 | BC059060 | hypothetical protein | 1.61 | 0,0313 |
| A\_51\_P361204 | NM\_013477 | Mus musculus ATPase, H+ transporting, lysosomal (vacuolar proton pump), 42 kDa (Atp6d), mRNA | 1.61 | 0,0113 |
| A\_51\_P352114 | AK029175 | unclassifiable | 1.61 | 0,0148 |
| A\_51\_P435990 | NM\_022565 | Mus musculus N-deacetylase/N-sulfotransferase (heparin glucosaminyl) 4 (Ndst4), mRNA | 1.61 | 0,0003 |
| A\_51\_P261107 | NM\_139144 | UDP-N-ACETYLGLUCOSAMINYLTRANSFERASE homolog [Mus musculus] | 1.61 | 0,0159 |
| A\_51\_P266763 | NM\_007998 | Mus musculus ferrochelatase (Fech), mRNA | 1.61 | 0,0360 |
| A\_51\_P513224 | XM\_622103 | hypothetical protein | 1.61 | 0,0008 |
| A\_51\_P316943 | AK012728 | unknown EST | 1.61 | 0,0056 |
| A\_51\_P110671 | NM\_009074 | Mus musculus macrophage stimulating 1 receptor (c-met-related tyrosine kinase) (Mst1r), mRNA | 1.61 | 0,0023 |
| A\_51\_P438679 | NM\_144806 | Mus musculus, phosphoribosyl pyrophosphate synthetase-associated protein 2, clone MGC:36957 IMAGE:4947226, mRNA, complete cds | 1.60 | 0,0074 |
| A\_51\_P298033 | BC027567 | Mus musculus, clone IMAGE:3472070, mRNA | 1.60 | 0,0008 |
| A\_51\_P232107 | NM\_011822 | Mus musculus phosphatidylinositol glycan, class Q (Pigq), mRNA | 1.60 | 0,0263 |
| A\_51\_P395727 | NM\_019575 | Mus musculus secretory carrier membrane protein 4 (Scamp4), mRNA | 1.60 | 0,0016 |
| A\_51\_P210963 | AK036382 | unclassifiable | 1.60 | 0,0260 |
| A\_51\_P105800 | XM\_146511 | hypothetical Protein phosphatase 2C domain/Leucine-rich repeat, typical subtype containing protein | 1.60 | 0,0085 |
| A\_51\_P460904 | BU592977 | Mus musculus cDNA, 5 end | 1.60 | 0,0034 |
| A\_51\_P398406 | NM\_172747 | TNFAIP1-LIKE PROTEIN homolog [Homo sapiens] | 1.60 | 0,0306 |
| A\_51\_P318770 | NM\_153555 | H326 PROTEIN homolog [Homo sapiens] | 1.60 | 0,0013 |
| A\_51\_P513000 | NM\_030699 | Mus musculus netrin G1 (Ntng1), mRNA | 1.60 | 0,0403 |
| A\_51\_P364671 | NM\_029272 | similar to NADH-UBIQUINONE OXIDOREDUCTASE 20 KDA SUBUNIT, MITOCHONDRIAL PRECURSOR (EC 1.6.5.3) (EC 1.6.99.3) (COMPLEX I-20KD) (CI-20KD) (PSST SUBUNIT) [Homo sapiens] | 1.60 | 0,0148 |
| A\_51\_P225083 | NM\_153680 | Mus musculus, Similar to sorting nexin 17, clone MGC:36375 IMAGE:4986268, mRNA, complete cds | 1.60 | 0,0099 |
| A\_51\_P498863 | NM\_053168 | Mus musculus tripartite motif protein 11 (Trim11), mRNA | 1.60 | 0,0304 |
| A\_51\_P487547 | NM\_025911 | Mus musculus RIKEN cDNA 1810060J02 gene (1810060J02Rik), mRNA | 1.60 | 0,0167 |
| A\_51\_P201945 | NM\_025747 | unknown EST | 1.60 | 0,0264 |
| A\_51\_P451377 | NM\_023125 | Mus musculus kininogen (Kng), mRNA | 1.60 | 0,0065 |
| A\_51\_P312576 | AK054222 | hypothetical protein | 1.60 | 0,0031 |
| A\_51\_P246146 | NM\_133714 | Mus musculus RIKEN cDNA 2310037I24 gene (2310037I24Rik), mRNA | 1.60 | 0,0013 |
| A\_51\_P445765 | TC1519807 | Mus musculus cDNA, 5 end | 1.60 | 0,0007 |
| A\_51\_P508411 | NM\_177856 | hypothetical Staphylococcus nuclease (SNase) homologues containing protein | 1.60 | 0,0067 |
| A\_51\_P115018 | NM\_001003815 | Mus musculus erythrocyte protein band 4.1-like 1 (Epb4.1l1), mRNA | 1.60 | 0,0058 |
| A\_51\_P486568 | ENSMUST00000036357 | Mus musculus olfactory receptor MOR103-11 (MOR103-11), mRNA | 1.60 | 0,0053 |
| A\_51\_P174158 | NM\_010571 | Mus musculus insulin receptor substrate-3 (IRS-3) mRNA, partial cds | 1.60 | 0,0048 |
| A\_51\_P490999 | BC031785 | M.musculus mRNA polyA site sequence | 1.60 | 0,0164 |
| A\_51\_P202074 | NM\_146171 | Mus musculus, clone IMAGE:3499621, mRNA, partial cds | 1.60 | 0,0043 |
| A\_51\_P311659 | AK031823 | unclassifiable | 1.60 | 0,0179 |
| A\_51\_P379660 | NM\_019914 | Mus musculus ALL1-fused gene from chromosome 1q (Af1q-pending), mRNA | 1.60 | 0,0205 |
| A\_51\_P510441 | NM\_011514 | Mus musculus suppressor of variegation 3-9 homolog 1 (Drosophila) (Suv39h1), mRNA | 1.59 | 0,0101 |
| A\_51\_P289881 | NM\_025334 | Mus musculus RIKEN cDNA 0610040B21 gene (0610040B21Rik), mRNA | 1.59 | 0,0121 |
| A\_51\_P123091 | NM\_029274 | ZINC FINGER PROTEIN HRX ALL 1 | 1.59 | 0,0128 |
| A\_51\_P343598 | AK012407 | unknown EST | 1.59 | 0,0098 |
| A\_51\_P156927 | XM\_354675 | RETINOBLASTOMA-BINDING PROTEIN 1 (RBBP-1) homolog [Homo sapiens] | 1.59 | 0,0190 |
| A\_51\_P166107 | AK075904 | unknown EST | 1.59 | 0,0032 |
| A\_51\_P175205 | AK079278 | RIBOSOMAL PROTEIN S6 KINASE ALPHA EC 2.7.1.- S6K ALPHA KDA RIBOSOMAL PROTEIN S6 KINASE P90 RSK RIBOSOMAL S6 KINASE RSK | 1.59 | 0,0044 |
| A\_51\_P505694 | NM\_008916 | Mus musculus putative phosphatase (Pps), mRNA | 1.59 | 0,0011 |
| A\_51\_P229759 | AK054255 | hypothetical Cysteine-rich flanking region, C-terminal/Leucine-rich repeat/Immunoglobulin and major histocompatibility complex domain/Immunoglobulin C-2 type/Leucine-rich repeat, typical subtype/Immunoglobulin subtype containing protein | 1.59 | 0,0055 |
| A\_51\_P125745 | NM\_012055 | Mus musculus, Similar to asparagine synthetase, clone MGC:5995 IMAGE:3585775, mRNA, complete cds | 1.59 | 0,0104 |
| A\_51\_P264388 | NM\_013931 | Mus musculus mitogen-activated protein kinase 8 interacting protein 3 (Mapk8ip3), mRNA | 1.59 | 0,0267 |
| A\_51\_P337065 | AK086050 | similar to PYRUVATE DEHYDROGENASE PHOSPHATASE REGULATORY SUBUNIT [Bos taurus] | 1.59 | 0,0043 |
| A\_51\_P331363 | NM\_030184 | hypothetical ARM repeat structure containing protein | 1.59 | 0,0325 |
| A\_51\_P221762 | NM\_007936 | Eph receptor A4 | 1.59 | 0,0214 |
| A\_51\_P248304 | NM\_178027 | weakly similar to VPS26 PROTEIN HOMOLOG (H<BETA58 PROTEIN) (H BETA 58) [Mus musculus] | 1.59 | 0,0042 |
| A\_51\_P315646 | NM\_010911 | Mus musculus nitrogen fixation gene 1 (S. cerevisiae) (Nfs1), mRNA | 1.59 | 0,0013 |
| A\_51\_P145993 | NM\_026899 | PNAS-120 homolog [Homo sapiens] | 1.59 | 0,0293 |
| A\_51\_P286814 | NM\_011424 | Mus musculus nuclear receptor co-repressor 2 (Ncor2), mRNA | 1.59 | 0,0042 |
| A\_51\_P428157 | NM\_028351 | THROMBOSPONDIN homolog [Homo sapiens] | 1.59 | 0,0014 |
| A\_51\_P423527 | NM\_175007 | Mus musculus, Similar to amphiphysin, clone IMAGE:5357091, mRNA, partial cds | 1.59 | 0,0182 |
| A\_51\_P457989 | NM\_027491 | BA11D8.2.2 (NOVEL PROTEIN (TRANSLATION OF CDNA DKFZP761H171 (EM:AL137502))) (POSSIBLE GTP BINDING PROTEIN (ISOFORM 2)) (HYPOTHETICAL GTP-BINDING PROTEIN DKFZP761H171) homolog [Homo sapiens] | 1.59 | 0,0221 |
| A\_51\_P183985 | BC023061 | hypothetical protein | 1.59 | 0,0162 |
| A\_51\_P117449 | AK019191 | Wolf-Hirschhorn syndrome candidate 2 homolog (human) | 1.59 | 0,0242 |
| A\_51\_P247665 | NM\_178811 | Mus musculus RIKEN cDNA D930014A20 gene (D930014A20Rik), mRNA | 1.59 | 0,0424 |
| A\_51\_P209071 | NM\_028469 | hypothetical protein | 1.59 | 0,0207 |
| A\_51\_P301435 | NM\_033149 | unknown EST | 1.59 | 0,0071 |
| A\_51\_P293926 | NM\_030702 | Mus musculus smt3-specific isopeptidase 1 (Smt3ip1-pending), mRNA | 1.59 | 0,0033 |
| A\_51\_P218196 | NM\_010835 | Mus musculus homeo box, msh-like 1 (Msx1), mRNA | 1.58 | 0,0074 |
| A\_51\_P174362 | NM\_028789 | hypothetical RING finger containing protein | 1.58 | 0,0156 |
| A\_51\_P248999 | NM\_054040 | Mus musculus tubby like protein 4 (Tulp4), mRNA | 1.58 | 0,0456 |
| A\_51\_P434447 | NM\_010443 | Mus musculus heme oxygenase (decycling) 2 (Hmox2), mRNA | 1.58 | 0,0257 |
| A\_51\_P227463 | NM\_172754 | hypothetical Zinc finger, C2H2 type containing protein | 1.58 | 0,0238 |
| A\_51\_P432877 | AK008158 | Mus musculus, RIKEN cDNA 2010008E23 gene, clone IMAGE:4482737, mRNA, partial cds | 1.58 | 0,0031 |
| A\_51\_P457054 | NM\_172990 | FANG1 homolog [Rattus norvegicus] | 1.58 | 0,0037 |
| A\_51\_P463570 | NM\_008641 | Mus musculus microtubule associated testis specific serine/threonine protein kinase (Mtssk), mRNA | 1.58 | 0,0191 |
| A\_51\_P493987 | NM\_021509 | Mus musculus RIKEN cDNA 3230402N08 gene (3230402N08Rik), mRNA | 1.58 | 0,0027 |
| A\_51\_P486715 | NM\_008044 | Mus musculus Friedreich ataxia (Frda), mRNA | 1.58 | 0,0046 |
| A\_51\_P423398 | A\_51\_P423398 | Mus musculus cDNA, 5 end | 1.58 | 0,0009 |
| A\_51\_P143082 | NM\_026394 | Mus musculus RIKEN cDNA 1110055J05 gene (1110055J05Rik), mRNA | 1.58 | 0,0107 |
| A\_51\_P345186 | NM\_018877 | Mus musculus SET domain, bifurcated 1 (Setdb1), mRNA | 1.58 | 0,0153 |
| A\_51\_P196127 | NM\_011863 | Mus musculus 3-phosphoadenosine 5-phosphosulfate synthase 1 (Papss1), mRNA | 1.58 | 0,0004 |
| A\_51\_P178575 | AF269193 | Mus musculus bromodomain-containing 3 (Brd3), mRNA | 1.58 | 0,0107 |
| A\_51\_P235193 | NM\_172926 | SORTING NEXIN | 1.58 | 0,0032 |
| A\_51\_P173459 | NM\_175538 | hypothetical Eukaryotic protein kinase containing protein | 1.58 | 0,0232 |
| A\_51\_P388734 | NM\_008732 | Mus musculus solute carrier family 11 (proton-coupled divalent metal ion transporters), member 2 (Slc11a2), mRNA | 1.58 | 0,0082 |
| A\_51\_P104891 | AK122544 | Mus musculus cDNA, 3 end | 1.58 | 0,0053 |
| A\_51\_P286878 | AK016577 | hypothetical Glutathione synthetase ATP-binding domain-like structure containing protein | 1.58 | 0,0052 |
| A\_51\_P111352 | NM\_175154 | N-ACETYLGALACTOSAMINE KINASE (EC 2.7.1.-) (GALNAC KINASE) (GALACTOKINASE 2) homolog [Homo sapiens] | 1.58 | 0,0004 |
| A\_51\_P232717 | NM\_018878 | Mus musculus PAX interacting (with transcription-activation domain) protein 1 (Paxip1), mRNA | 1.58 | 0,0055 |
| A\_51\_P158305 | M29242 | Mouse U5 small nuclear RNA, clone 2 | 1.58 | 0,0008 |
| A\_51\_P268884 | NM\_024249 | Mus musculus, clone IMAGE:3708675, mRNA, partial cds | 1.58 | 0,0104 |
| A\_51\_P389099 | AK040491 | unknown EST | 1.58 | 0,0220 |
| A\_51\_P127777 | NM\_018740 | Mus musculus retinoic acid induced 12 (Rai12), mRNA | 1.58 | 0,0010 |
| A\_51\_P140498 | AK016808 | weakly similar to WUGSC:H\_RG013N12.GW.1335199.A PROTEIN (FRAGMENT) [Homo sapiens] | 1.58 | 0,0111 |
| A\_51\_P244453 | NM\_172650 | BA275K8.1 (NY-REN-45 ANTIGEN) homolog [Homo sapiens] | 1.58 | 0,0278 |
| A\_51\_P186118 | NM\_024201 | Mus musculus RIKEN cDNA 0610011N22 gene (0610011N22Rik), mRNA | 1.58 | 0,0366 |
| A\_51\_P242446 | NM\_177758 | ZINC FINGER PROTEIN 31 (ZINC FINGER PROTEIN KOX29) (FRAGMENT) homolog [Homo sapiens] | 1.58 | 0,0285 |
| A\_51\_P189438 | NM\_172272 | "Mus musculus, similar to glycyl-tRNA synthetase� GlyRS� glycine tRNA ligase, clone IMAGE:5360188, mRNA, partial cds" | 1.58 | 0,0021 |
| A\_51\_P291078 | NM\_172710 | hypothetical Concanavalin A-like lectins/glucanases structure containing protein | 1.58 | 0,0425 |
| A\_51\_P450018 | NM\_025933 | Mus musculus RIKEN cDNA 2010110M21 gene (2010110M21Rik), mRNA | 1.58 | 0,0029 |
| A\_51\_P258570 | NM\_011177 | Mus musculus protease, serine, 18 (Prss18), mRNA | 1.58 | 0,0148 |
| A\_51\_P123047 | NM\_019710 | Mus musculus SMC (structural maintenance of chromosomes 1)-like 1 (S. cerevisiae) (Smc1l1), mRNA | 1.57 | 0,0035 |
| A\_51\_P465785 | NM\_019940 | Mus musculus zinc finger protein 111 (Zfp111), mRNA | 1.57 | 0,0349 |
| A\_51\_P486488 | AK040705 | unclassifiable | 1.57 | 0,0177 |
| A\_51\_P280244 | NM\_011751 | Mus musculus zinc finger protein 207 (Zfp207), mRNA | 1.57 | 0,0046 |
| A\_51\_P147654 | AK013971 | hypothetical protein | 1.57 | 0,0246 |
| A\_51\_P465740 | NM\_175215 | hypothetical LysM motif containing protein | 1.57 | 0,0448 |
| A\_51\_P246684 | NM\_020329 | Mus musculus RIKEN cDNA 0610011H20 gene (0610011H20Rik), mRNA | 1.57 | 0,0456 |
| A\_51\_P414790 | NM\_178005 | hypothetical RNI-like structure containing protein | 1.57 | 0,0126 |
| A\_51\_P190254 | AK129084 | unclassifiable | 1.57 | 0,0035 |
| A\_51\_P156697 | AK015837 | hypothetical Zinc finger, C2H2 type containing protein | 1.57 | 0,0026 |
| A\_51\_P195573 | NM\_010066 | Mus musculus DNA methyltransferase (cytosine-5) 1 (Dnmt1), mRNA | 1.57 | 0,0019 |
| A\_51\_P353502 | NM\_025340 | Mus musculus, Similar to shank-interacting protein, clone MGC:27664 IMAGE:4527839, mRNA, complete cds | 1.57 | 0,0024 |
| A\_51\_P326030 | NM\_145490 | Mus musculus, Similar to zinc finger protein 97, clone MGC:18740 IMAGE:3986622, mRNA, complete cds | 1.57 | 0,0168 |
| A\_51\_P428903 | AK016770 | Mus musculus RIKEN cDNA 4933411G06 gene (4933411G06Rik), mRNA | 1.57 | 0,0405 |
| A\_51\_P416660 | NM\_172587 | TYROSINE PHOSPHATASE homolog [Homo sapiens] | 1.57 | 0,0013 |
| A\_51\_P331661 | NM\_027873 | TRANSITIONAL EPITHELIA RESPONSE PROTEIN homolog [Homo sapiens] | 1.57 | 0,0122 |
| A\_51\_P314631 | NM\_008860 | Mus musculus protein kinase C, zeta (Prkcz), mRNA | 1.57 | 0,0310 |
| A\_51\_P501529 | NM\_175235 | RNA-BINDING PROTEIN BRUNOL5 (FRAGMENT) homolog [Homo sapiens] | 1.57 | 0,0127 |
| A\_51\_P367223 | NM\_009012 | Mus musculus RAD50 homolog (S. cerevisiae) (Rad50), mRNA | 1.57 | 0,0107 |
| A\_51\_P319592 | AK014101 | UBIQUITIN CARBOXYL TERMINAL HYDROLASE DUB 1 EC 3.1.2.15 UBIQUITIN THIOLESTERASE DUB 1 UBIQUITIN SPECIFIC PROCESSING PROTEASE DUB 1 DEUBIQUITINATING ENZYME | 1.57 | 0,0187 |
| A\_51\_P187082 | NM\_008062 | Mus musculus glucose-6-phosphate dehydrogenase X-linked (G6pdx), mRNA | 1.57 | 0,0440 |
| A\_51\_P171553 | NM\_145412 | Mus musculus RIKEN cDNA 5830457O10 gene (5830457O10Rik), mRNA | 1.57 | 0,0151 |
| A\_51\_P431649 | NM\_133224 | Mus musculus cation-transporting atpase (catp), mRNA | 1.57 | 0,0045 |
| A\_51\_P126817 | NM\_020580 | TH1-like homolog (Drosophila) | 1.57 | 0,0044 |
| A\_51\_P320843 | NM\_025921 | Mus musculus RIKEN cDNA 2610002M06 gene (2610002M06Rik), mRNA | 1.57 | 0,0061 |
| A\_51\_P248243 | NM\_010903 | Mus musculus nuclear factor, erythroid derived 2, like 3 (Nfe2l3), mRNA | 1.57 | 0,0163 |
| A\_51\_P249297 | NM\_011994 | Mus musculus ATP-binding cassette, sub-family D (ALD), member 2 (Abcd2), mRNA | 1.57 | 0,0222 |
| A\_51\_P496074 | NM\_178162 | weakly similar to SIMILAR TO REV/REX ACTIVATION DOMAIN BINDING PROTEIN-RELATED [Homo sapiens] | 1.57 | 0,0297 |
| A\_51\_P378816 | AK017613 | inferred: RIKEN cDNA 5730434B08 gene | 1.57 | 0,0381 |
| A\_51\_P488799 | NM\_173761 | DJ963E22.1 (NOVEL PROTEIN SIMILAR TO NY-REN-2 ANTIGEN) (FRAGMENT) homolog [Homo sapiens] | 1.57 | 0,0046 |
| A\_51\_P111612 | BC025091 | hypothetical Immunoglobulin structure containing protein | 1.57 | 0,0464 |
| A\_51\_P250358 | NM\_177806 | CDNA FLJ11128 FIS, CLONE PLACE1006236 homolog [Homo sapiens] | 1.57 | 0,0153 |
| A\_51\_P417321 | AK041130 | weakly similar to ZINC FINGER PROTEIN (FRAGMENT) [Homo sapiens] | 1.57 | 0,0033 |
| A\_51\_P465473 | NM\_027453 | TRANSCRIPTION FACTOR BTF3 RNA POLYMERASE B TRANSCRIPTION FACTOR | 1.56 | 0,0034 |
| A\_51\_P498909 | NM\_146805 | Mus musculus olfactory receptor MOR165-5 (MOR165-5), mRNA | 1.56 | 0,0029 |
| A\_51\_P400909 | NM\_011665 | UBIQUITIN CONJUGATING ENZYME EC 6.3.2.19 UBIQUITIN PROTEIN LIGASE UBIQUITIN CARRIER PROTEIN | 1.56 | 0,0161 |
| A\_51\_P497263 | NM\_010837 | Mus musculus microtubule-associated protein 6 (Mtap6), mRNA | 1.56 | 0,0030 |
| A\_51\_P152423 | NM\_177640 | hypothetical Actinin-type actin-binding domain containing protein | 1.56 | 0,0028 |
| A\_51\_P467751 | NM\_133970 | Mus musculus RIKEN cDNA 9430010M12 gene (9430010M12Rik), mRNA | 1.56 | 0,0013 |
| A\_51\_P325343 | NM\_011951 | Mus musculus mitogen activated protein kinase 14 (Mapk14), mRNA | 1.56 | 0,0084 |
| A\_51\_P398848 | NM\_197987 | TRAF encompassing factor 3 | 1.56 | 0,0099 |
| A\_51\_P356580 | NM\_026280 | hypothetical protein | 1.56 | 0,0165 |
| A\_51\_P513211 | NM\_138660 | Mus musculus MLN51 protein (MLN51), mRNA | 1.56 | 0,0024 |
| A\_51\_P364099 | NM\_175146 | hypothetical protein | 1.56 | 0,0359 |
| A\_51\_P119306 | AK053070 | unknown EST | 1.56 | 0,0080 |
| A\_51\_P500226 | NM\_028024 | KAPPA B-RAS 2 (I-KAPPA-B-INTERACTING RAS-LIKE PROTEIN 2) homolog [Homo sapiens] | 1.56 | 0,0166 |
| A\_51\_P457492 | NM\_025791 | Mus musculus RIKEN cDNA 0610006I08 gene (0610006I08Rik), mRNA | 1.56 | 0,0378 |
| A\_51\_P332185 | AK021136 | hypothetical protein | 1.56 | 0,0027 |
| A\_51\_P504962 | NM\_146165 | Mus musculus, clone MGC:36522 IMAGE:5371230, mRNA, complete cds | 1.56 | 0,0322 |
| A\_51\_P496735 | AK039103 | 39 KDA ANTIGEN homolog [Leishmania donovani] | 1.56 | 0,0037 |
| A\_51\_P160344 | AK014046 | hypothetical protein | 1.56 | 0,0237 |
| A\_51\_P423361 | TC1433143 | Mus musculus cDNA, 5 end | 1.56 | 0,0306 |
| A\_51\_P479832 | AK016708 | DNA-binding protein (fragment) homolog [Homo sapiens] | 1.56 | 0,0023 |
| A\_51\_P237553 | NM\_009652 | Mus musculus thymoma viral proto-oncogene 1 (Akt1), mRNA | 1.56 | 0,0100 |
| A\_51\_P167097 | AK038845 | hypothetical protein | 1.56 | 0,0392 |
| A\_51\_P215922 | NM\_009811 | Mus musculus caspase 6 (Casp6), mRNA | 1.56 | 0,0346 |
| A\_51\_P478279 | NM\_053170 | Mus musculus tripartite motif protein TRIM33 (Trim33) mRNA, partial cds | 1.56 | 0,0008 |
| A\_51\_P364031 | NM\_026213 | similar to OSMOSIS RESPONSIVE FACTOR [Homo sapiens] | 1.56 | 0,0160 |
| A\_51\_P278489 | NM\_133927 | Mus musculus expressed sequence AI646725 (AI646725), mRNA | 1.55 | 0,0065 |
| A\_51\_P392244 | NM\_029878 | similar to TUBULIN-SPECIFIC CHAPERONE D [Homo sapiens] | 1.55 | 0,0024 |
| A\_51\_P110395 | XM\_486265 | HUNTINGTIN INTERACTING PROTEIN (FRAGMENT) homolog [Homo sapiens] | 1.55 | 0,0218 |
| A\_51\_P380330 | AK033743 | hypothetical Lipocalins structure containing protein | 1.55 | 0,0023 |
| A\_51\_P124467 | BC063268 | unknown EST | 1.55 | 0,0156 |
| A\_51\_P338244 | NM\_011591 | Mus musculus similar to Mitochondrial import inner membrane translocase subunit TIM17 B (LOC272751), mRNA | 1.55 | 0,0128 |
| A\_51\_P226791 | A\_51\_P226791 | Mus musculus cytochrome P450, 11a, cholesterol side chain cleavage (Cyp11a), mRNA | 1.55 | 0,0014 |
| A\_51\_P335480 | NM\_028077 | Mus musculus, RIKEN cDNA 1810055G02 gene, clone MGC:28461 IMAGE:4161022, mRNA, complete cds | 1.55 | 0,0132 |
| A\_51\_P335089 | AK019621 | hypothetical DHHC-type Zn-finger/Zn-finger CCHC type/N-6 Adenine-specific DNA methylase/ATP/GTP-binding site motif A (P-loop) containing protein | 1.55 | 0,0135 |
| A\_51\_P505464 | AK011828 | huntingtin-interacting protein 1, full insert sequence. | 1.55 | 0,0197 |
| A\_51\_P459340 | NM\_025755 | hypothetical ARM repeat structure containing protein | 1.55 | 0,0119 |
| A\_51\_P185563 | NM\_145742 | PROBABLE ATP-DEPENDENT HELICASE DDX35 (DEAH-BOX PROTEIN 35) homolog [Homo sapiens] | 1.55 | 0,0003 |
| A\_51\_P460499 | NM\_001001978 | Mus musculus glyceraldehyde-3-phosphate dehydrogenase (Gapd), mRNA | 1.55 | 0,0449 |
| A\_51\_P407690 | NM\_175195 | SIMILAR TO HYPOTHETICAL PROTEIN FROM EUROIMAGE 42353 homolog [Homo sapiens] | 1.55 | 0,0188 |
| A\_51\_P418374 | NM\_032399 | Mus musculus G protein-coupled receptor 87 (Gpr87), mRNA | 1.55 | 0,0017 |
| A\_51\_P454691 | AK036647 | hypothetical protein | 1.55 | 0,0025 |
| A\_51\_P207940 | NM\_178789 | hypothetical protein | 1.55 | 0,0089 |
| A\_51\_P135888 | NM\_010897 | Mouse neurofibromin (NF1) mRNA, 3 end | 1.55 | 0,0132 |
| A\_51\_P180891 | NM\_028341 | hypothetical Tetratricopeptide repeat (TPR) structure containing protein | 1.55 | 0,0372 |
| A\_51\_P331090 | BC009019 | Mus musculus expressed sequence AI661311 (AI661311), mRNA | 1.55 | 0,0423 |
| A\_51\_P408932 | NM\_024186 | Mus musculus single-stranded DNA binding protein 2 (Ssbp2), mRNA | 1.55 | 0,0288 |
| A\_51\_P136838 | NM\_027804 | Mus musculus, clone MGC:11980 IMAGE:3601364, mRNA, complete cds | 1.55 | 0,0465 |
| A\_51\_P260639 | NM\_028811 | elongation protein 3 homolog (S. cerevisiae) | 1.55 | 0,0247 |
| A\_51\_P318683 | AF031816 | Mus musculus hybrid receptor gp250 precursor, mRNA, partial cds | 1.55 | 0,0102 |
| A\_51\_P501720 | AK042850 | unclassifiable | 1.55 | 0,0112 |
| A\_51\_P300657 | NM\_010904 | Mouse neurofilament component (NF-H) mRNA, complete cds | 1.55 | 0,0332 |
| A\_51\_P293781 | NM\_016863 | Mus musculus FK506 binding protein 1b (12.6 kDa) (Fkbp1b), mRNA | 1.55 | 0,0191 |
| A\_51\_P440892 | NM\_026344 | similar to DIPHTHAMIDE BIOSYNTHESIS PROTEIN-2 [Homo sapiens] | 1.55 | 0,0195 |
| A\_51\_P341379 | NM\_172678 | Y79AA1001048 PROTEIN (HYPOTHETICAL 68.8 KDA PROTEIN) (UNKNOWN) (PROTEIN FOR MGC:14452) homolog [Homo sapiens] | 1.55 | 0,0077 |
| A\_51\_P352824 | NM\_026369 | Mus musculus actin related protein 2/3 complex, subunit 5 (165 kDa) (Arpc5), mRNA | 1.55 | 0,0280 |
| A\_51\_P279571 | NM\_025932 | synapse associated protein 1 | 1.55 | 0,0145 |
| A\_51\_P478739 | NM\_021901 | Mus musculus T-cell leukemia, homeobox 1 (Tlx1), mRNA | 1.55 | 0,0426 |
| A\_51\_P152216 | NM\_025693 | Mus musculus, Similar to RIKEN cDNA 5730578N08 gene, clone IMAGE:3983912, mRNA | 1.54 | 0,0409 |
| A\_51\_P264527 | NM\_019833 | Mus musculus hypothetical protein, clone 1-82 (AB030186), mRNA | 1.54 | 0,0012 |
| A\_51\_P123724 | AI842957 | Mus musculus cDNA, 3 end | 1.54 | 0,0291 |
| A\_51\_P136699 | XM\_127105 | weakly similar to HYPOTHETICAL PROTEIN KIAA1140 (FRAGMENT) [Homo sapiens] | 1.54 | 0,0282 |
| A\_51\_P453541 | NM\_013908 | Mus musculus f-box and WD-40 domain protein 5 (Fbxw5), mRNA | 1.54 | 0,0104 |
| A\_51\_P355112 | NAP057292-1 | Mus musculus olfactory receptor MOR135-17 (MOR135-17) pseudogene | 1.54 | 0,0087 |
| A\_51\_P300666 | NM\_153288 | Mus musculus neuropeptide B (Npb-pending), mRNA | 1.54 | 0,0232 |
| A\_51\_P129642 | NM\_028963 | hypothetical protein | 1.54 | 0,0418 |
| A\_51\_P224023 | NM\_144846 | Mus musculus RIKEN cDNA 0910001A06 gene (0910001A06Rik), mRNA | 1.54 | 0,0290 |
| A\_51\_P138952 | NM\_028815 | hypothetical IQ calmodulin-binding motif/Leucine-rich repeat containing protein | 1.54 | 0,0237 |
| A\_51\_P381387 | NM\_153415 | POMT2 homolog [Homo sapiens] | 1.54 | 0,0034 |
| A\_51\_P453043 | NM\_030210 | ACETOACETYL-COA SYNTHETASE (EC 6.2.1.16) homolog [Rattus norvegicus] | 1.54 | 0,0026 |
| A\_51\_P190198 | NM\_027438 | PARANEOPLASTIC ONCONEURONAL PROTEIN MA1 homolog [Rattus norvegicus] | 1.54 | 0,0283 |
| A\_51\_P258561 | AK046448 | musculus, clone IMAGE:5025694, mRNA | 1.54 | 0,0032 |
| A\_51\_P284728 | AK018518 | unknown EST | 1.54 | 0,0409 |
| A\_51\_P466773 | NM\_172749 | similar to HYPOTHETICAL ZINC FINGER PROTEIN KIAA0296 [Homo sapiens] | 1.54 | 0,0046 |
| A\_51\_P162773 | NM\_178653 | hypothetical NAD(P)-binding Rossmann-fold domains structure containing protein | 1.54 | 0,0097 |
| A\_51\_P484238 | NM\_027530 | RAP2 INTERACTING PROTEIN X homolog [Homo sapiens] | 1.54 | 0,0051 |
| A\_51\_P366259 | NM\_010946 | Mus musculus N-terminal Asn amidase (Ntan1), mRNA | 1.54 | 0,0048 |
| A\_51\_P357138 | NM\_019861 | Mus musculus cathepsin F (Ctsf), mRNA | 1.54 | 0,0297 |
| A\_51\_P516994 | NM\_027886 | similar to LKB1-INTERACTING PROTEIN 1 [Homo sapiens] | 1.54 | 0,0023 |
| A\_51\_P297346 | NM\_029153 | SECRETORY CARRIER-ASSOCIATED MEMBRANE PROTEIN 1 | 1.54 | 0,0084 |
| A\_51\_P249514 | AK087211 | unknown EST | 1.54 | 0,0240 |
| A\_51\_P253904 | NM\_008704 | Mus musculus nucleoside diphosphate kinase A long form mRNA, complete cds | 1.54 | 0,0012 |
| A\_51\_P503993 | NM\_172449 | RIM BINDING PROTEIN 1B (FRAGMENT) homolog [Rattus norvegicus] | 1.54 | 0,0060 |
| A\_51\_P369508 | NM\_025784 | BCS1-like (yeast) | 1.54 | 0,0060 |
| A\_51\_P285042 | XM\_283757 | unknown EST | 1.54 | 0,0075 |
| A\_51\_P332165 | NM\_026077 | Mus musculus RIKEN cDNA 3110040N11 gene (3110040N11Rik), mRNA | 1.54 | 0,0104 |
| A\_51\_P514776 | NM\_009771 | Mus musculus beta-transducin repeat containing protein (Btrc), mRNA | 1.54 | 0,0177 |
| A\_51\_P283754 | NM\_172302 | weakly similar to SIMILAR TO CLEAVAGE AND POLYADENYLATION SPECIFIC FACTOR 6, 68KD SUBUNIT [Homo sapiens] | 1.54 | 0,0121 |
| A\_51\_P483220 | NM\_008378 | Mus musculus imprinted and ancient (Impact), mRNA | 1.54 | 0,0196 |
| A\_51\_P128320 | BC030862 | weakly similar to mucin-like peptide MLP 2677 [Rattus norvegicus] | 1.54 | 0,0017 |
| A\_51\_P199899 | NM\_011936 | Mus musculus fatso (Fto), mRNA | 1.54 | 0,0377 |
| A\_51\_P463816 | AK036188 | hypothetical AMP-dependent synthetase and ligase containing protein | 1.53 | 0,0162 |
| A\_51\_P487263 | NM\_173038 | hypothetical RNI-like structure containing protein | 1.53 | 0,0204 |
| A\_51\_P422639 | AK013707 | hypothetical protein | 1.53 | 0,0163 |
| A\_51\_P296905 | XM\_620209 | inferred: amidophosphoribosyltransferase precursor {Rattus norvegicus} | 1.53 | 0,0226 |
| A\_51\_P298190 | XM\_488538 | MYOTUBULARIN HOMOLOGOUS PROTEIN 1 (FRAGMENT) | 1.53 | 0,0478 |
| A\_51\_P450887 | NM\_022982 | Mus musculus reticulon 4 receptor (Rtn4r), mRNA | 1.53 | 0,0372 |
| A\_51\_P401864 | XM\_484947 | unknown EST | 1.53 | 0,0252 |
| A\_51\_P502432 | NM\_009785 | Mus musculus calcium channel, voltage dependent, alpha2/delta subunit 3 (Cacna2d3), mRNA | 1.53 | 0,0104 |
| A\_51\_P404687 | A\_51\_P404687 | Mus musculus cDNA, 5 end | 1.53 | 0,0411 |
| A\_51\_P181571 | NM\_177301 | Mus musculus mRNA for protein L, partial cds | 1.53 | 0,0103 |
| A\_51\_P518503 | BC068305 | SKI PROTO-ONCOGENE homolog [Mus musculus] | 1.53 | 0,0044 |
| A\_51\_P438952 | NM\_026121 | Mus musculus BCL2-associated athanogene 4 (Bag4), mRNA | 1.53 | 0,0405 |
| A\_51\_P334730 | NM\_020611 | Mus musculus steroid 5 alpha-reductase 2-like (Srd5a2l), mRNA | 1.53 | 0,0207 |
| A\_51\_P490795 | AK031169 | Max dimerization protein | 1.53 | 0,0364 |
| A\_51\_P176522 | NM\_019574 | Mus musculus mazr mRNA for transcription factor MAZR, complete cds | 1.53 | 0,0006 |
| A\_51\_P152203 | NM\_019565 | Mus musculus Kruppel associated box (KRAB) zinc finger 1 (Kzf1-pending), mRNA | 1.53 | 0,0324 |
| A\_51\_P202623 | NM\_028832 | similar to TRANSCRIPTION TERMINATION FACTOR-LIKE PROTEIN [Homo sapiens] | 1.53 | 0,0187 |
| A\_51\_P204730 | NM\_025892 | Mus musculus RIKEN cDNA 1500031L02 gene (1500031L02Rik), mRNA | 1.53 | 0,0014 |
| A\_51\_P220195 | NM\_028292 | Mus musculus, Similar to protein phosphatase methylesterase-1, clone MGC:11835 IMAGE:3596696, mRNA, complete cds | 1.53 | 0,0313 |
| A\_51\_P413853 | NM\_172282 | hypothetical Na+/H+ exchanger containing protein | 1.53 | 0,0185 |
| A\_51\_P273405 | BC024833 | SPECTRIN BETA CHAIN, BRAIN 1 (SPECTRIN, NON-ERYTHROID BETA CHAIN 1) (BETA-II SPECTRIN) (FODRIN BETA CHAIN) | 1.53 | 0,0045 |
| A\_51\_P170911 | NM\_019651 | Mus musculus protein tyrosine phosphatase, non-receptor type 9 (Ptpn9), mRNA | 1.53 | 0,0093 |
| A\_51\_P105515 | NM\_026053 | GEMIN6 homolog [Homo sapiens] | 1.53 | 0,0141 |
| A\_51\_P359173 | AK080792 | unknown EST | 1.53 | 0,0125 |
| A\_51\_P364231 | NM\_010752 | Mus musculus mitotic arrest deficient 1-like 1 (Mad1l1), mRNA | 1.53 | 0,0077 |
| A\_51\_P518851 | AK051071 | unknown EST | 1.52 | 0,0430 |
| A\_51\_P440936 | NM\_013581 | Mus musculus low density lipoprotein B (Ldlb), mRNA | 1.52 | 0,0150 |
| A\_51\_P494535 | AK050879 | unknown EST | 1.52 | 0,0034 |
| A\_51\_P181538 | NM\_145125 | Mus musculus mRNA for WDR9 protein (Wdr9 gene), form A | 1.52 | 0,0102 |
| A\_51\_P266191 | AK018108 | hypothetical Kelch repeat containing protein | 1.52 | 0,0081 |
| A\_51\_P453519 | NM\_023740 | Mus musculus RIKEN cDNA 1500015N03 gene (1500015N03Rik), mRNA | 1.52 | 0,0298 |
| A\_51\_P393863 | NM\_025716 | Mus musculus, RIKEN cDNA 4633402N23 gene, clone MGC:25683 IMAGE:4923277, mRNA, complete cds | 1.52 | 0,0082 |
| A\_51\_P225506 | NM\_026383 | Mus musculus RIKEN cDNA 0610011E17 gene (0610011E17Rik), mRNA | 1.52 | 0,0448 |
| A\_51\_P236439 | NM\_177322 | Mus musculus angiotensin receptor 1a (Agtr1a), mRNA | 1.52 | 0,0364 |
| A\_51\_P425149 | BG294923 | Mus musculus cDNA, 5 end | 1.52 | 0,0167 |
| A\_51\_P180091 | NM\_028775 | similar to CYTOCHROME P450 2S1 [Homo sapiens] | 1.52 | 0,0028 |
| A\_51\_P220062 | NM\_008609 | Mus musculus matrix metalloproteinase 15 (Mmp15), mRNA | 1.52 | 0,0329 |
| A\_51\_P419750 | AK051383 | unknown EST | 1.52 | 0,0035 |
| A\_51\_P258078 | NM\_138747 | Mus musculus, Similar to nucleolar protein 1 (120kD), clone MGC:6746 IMAGE:3592148, mRNA, complete cds | 1.52 | 0,0164 |
| A\_51\_P267353 | NM\_175478 | hypothetical Leucine-rich repeat containing protein | 1.52 | 0,0040 |
| A\_51\_P100785 | AK009218 | NUCLEAR PROTEIN SKIP (SKI-INTERACTING PROTEIN) (SNW1 PROTEIN) (NUCLEAR RECEPTOR COACTIVATOR NCOA-62) homolog [Homo sapiens] | 1.52 | 0,0065 |
| A\_51\_P424232 | NM\_010788 | Mus musculus methyl CpG binding protein 2 (Mecp2), mRNA | 1.52 | 0,0041 |
| A\_51\_P331805 | NM\_146188 | hypothetical BTB/POZ domain containing protein | 1.52 | 0,0283 |
| A\_51\_P351872 | NM\_008135 | Mus musculus glycine transporter 1 (Glyt1), mRNA | 1.52 | 0,0338 |
| A\_51\_P420726 | AK046032 | hypothetical Metallo-beta-lactamase superfamily containing protein | 1.52 | 0,0160 |
| A\_51\_P382666 | XM\_489366 | unknown EST | 1.52 | 0,0401 |
| A\_51\_P447808 | NM\_172509 | CDNA FLJ32069 FIS, CLONE OCBBF1000118, WEAKLY SIMILAR TO TUMOR NECROSIS FACTOR, ALPHA-INDUCED PROTEIN 1, ENDOTHELIAL homolog [Homo sapiens] | 1.52 | 0,0021 |
| A\_51\_P280125 | NM\_176860 | SIMILAR TO UBIQUITIN ASSOCIATED AND SH3 DOMAIN CONTAINING, A homolog [Homo sapiens] | 1.52 | 0,0211 |
| A\_51\_P387968 | NM\_198113 | Mus musculus, Similar to slingshot 3, clone MGC:25738 IMAGE:3987714, mRNA, complete cds | 1.52 | 0,0138 |
| A\_51\_P472998 | NAP057029-1 | Mus musculus zinc finger protein 148, pseudogene 1 (Zfp148-ps1) on chromosome 8 | 1.52 | 0,0071 |
| A\_51\_P277767 | NM\_024199 | CLEAVAGE STIMULATION FACTOR, 3 PRE-RNA, SUBUNIT 1, 50KD homolog [Homo sapiens] | 1.52 | 0,0489 |
| A\_51\_P519385 | NM\_030692 | Mus musculus SAC1 (supressor of actin mutations 1, homolog)-like (S. cerevisiae) (Sacm1l), mRNA | 1.52 | 0,0291 |
| A\_51\_P141288 | NM\_010693 | Mouse mRNA for tyrosine protein kinase p56-tck | 1.52 | 0,0098 |
| A\_51\_P452651 | NM\_020508 | Mus musculus bromodomain-containing 4 (Brd4), mRNA | 1.52 | 0,0351 |
| A\_51\_P121252 | NM\_027256 | Mus musculus, clone MGC:19455 IMAGE:3582093, mRNA, complete cds | 1.52 | 0,0148 |
| A\_51\_P492807 | AK008233 | weakly similar to 33 KDA VAMP-ASSOCIATED PROTEIN [Homo sapiens] | 1.52 | 0,0016 |
| A\_51\_P399853 | NM\_133218 | Mus musculus glucocorticoid-induced gene 1 (Gig1), mRNA | 1.52 | 0,0263 |
| A\_51\_P475465 | NM\_183270 | E2IG2 homolog [Homo sapiens] | 1.52 | 0,0134 |
| A\_51\_P456756 | NM\_145524 | Mus musculus, Similar to hypothetical protein FLJ13984, clone MGC:7995 IMAGE:3585719, mRNA, complete cds | 1.52 | 0,0231 |
| A\_51\_P430678 | AK004405 | similar to KU70-BINDING PROTEIN (FRAGMENT) [Homo sapiens] | 1.51 | 0,0466 |
| A\_51\_P408071 | XM\_132322 | hypothetical protein | 1.51 | 0,0161 |
| A\_51\_P437016 | BC058971 | Mus musculus, Similar to KIAA1042 protein, clone IMAGE:4217060, mRNA | 1.51 | 0,0365 |
| A\_51\_P281855 | NM\_028152 | Mus musculus MMS19 (MET18 S. cerevisiae)-like (Mms19l), mRNA | 1.51 | 0,0038 |
| A\_51\_P420176 | NM\_177103 | hypothetical SUMO/Sentrin/Ubl1 specific protease containing protein | 1.51 | 0,0052 |
| A\_51\_P234233 | NM\_001009545 | similar to TMDC IV PROTEIN [Rattus norvegicus] | 1.51 | 0,0025 |
| A\_51\_P154973 | NM\_021547 | Mus musculus steroidogenic acute regulatory protein related (Mln64-pending), mRNA | 1.51 | 0,0354 |
| A\_51\_P176459 | NM\_017479 | Mus musculus histone acetyltransferase (Morf-pending), mRNA | 1.51 | 0,0106 |
| A\_51\_P153982 | AK220465 | unknown EST | 1.51 | 0,0186 |
| A\_51\_P190124 | NM\_027347 | Mus musculus, RIKEN cDNA 3000002A17 gene, clone MGC:7510 IMAGE:3491622, mRNA, complete cds | 1.51 | 0,0091 |
| A\_51\_P108489 | NM\_008187 | Mus musculus gene trap locus 3 (Gtl3), mRNA | 1.51 | 0,0046 |
| A\_51\_P103865 | NM\_027242 | hypothetical protein | 1.51 | 0,0155 |
| A\_51\_P346453 | NM\_153570 | Mus musculus, Similar to hypothetical protein MGC3162, clone MGC:28606 IMAGE:4218310, mRNA, complete cds | 1.51 | 0,0136 |
| A\_51\_P259879 | NM\_173430 | CDNA FLJ12576 FIS, CLONE NT2RM4001032 (UNKNOWN) (PROTEIN FOR MGC:2991) (FUKUTIN-RELATED PROTEIN) homolog [Homo sapiens] | 1.51 | 0,0011 |
| A\_51\_P354272 | BC072573 | weakly similar to PUTATIVE EUKARYOTIC TRANSLATION INITIATION FACTOR 3 SUBUNIT (EIF-3) (FRAGMENT) [Homo sapiens] | 1.51 | 0,0010 |
| A\_51\_P400375 | NM\_021446 | Mus musculus open reading frame 11 (ORF11), mRNA | 1.51 | 0,0083 |
| A\_51\_P413803 | AK021319 | hypothetical protein | 1.51 | 0,0004 |
| A\_51\_P102782 | NM\_199195 | Mus musculus branched-chain alpha-ketoacid dehydrogenase E1 beta-subunit mRNA sequence | 1.51 | 0,0028 |
| A\_51\_P230347 | NM\_177774 | SRRP35 homolog [Homo sapiens] | 1.51 | 0,0183 |
| A\_51\_P265906 | BC066035 | hypothetical SNF2 related domain , Helicase c-terminal domain, DEAD/DEAH box helicase containing protein | 1.51 | 0,0244 |
| A\_51\_P433333 | NM\_053118 | Mus musculus G protein-coupled receptor, family C, group 5, member D (Gprc5d), mRNA | 1.51 | 0,0472 |
| A\_51\_P492437 | NM\_024286 | Mus musculus chromosome 10 popeye protein 3 (Pop3) mRNA, complete cds | 1.51 | 0,0444 |
| A\_51\_P399816 | AK039074 | unknown EST | 1.51 | 0,0054 |
| A\_51\_P353882 | BC066001 | LDL INDUCED ENDOTHELIAL CELL PROTEIN (FRAGMENT) homolog [Homo sapiens] | 1.51 | 0,0187 |
| A\_51\_P278840 | NM\_177321 | Mus musculus melanoma inhibitory activity protein 2 mRNA, complete cds | 1.51 | 0,0333 |
| A\_51\_P242555 | TC1500979 | Soares\_NMEBA\_branchial\_arch Mus musculus cDNA clone IMAGE:3970641 5. | 1.51 | 0,0220 |
| A\_51\_P283300 | NM\_023116 | calcium channel, voltage-dependent, beta 2 subunit | 1.51 | 0,0478 |
| A\_51\_P469188 | XM\_620089 | Mus musculus, clone IMAGE:3662238, mRNA | 1.51 | 0,0430 |
| A\_51\_P415059 | NM\_011496 | Mus musculus, serine/threonine kinase 5, clone MGC:5803 IMAGE:3501444, mRNA, complete cds | 1.51 | 0,0159 |
| A\_51\_P366120 | XM\_485738 | weakly similar to TRANSCRIPTION FACTOR BTEB4 (BASIC TRANSCRIPTION ELEMENT BINDING- PROTEIN 4) (BTE-BINDING PROTEIN 4) (NOVEL SP1-LIKE ZINC FINGER TRANSCRIPTION FACTOR 2) (TRANSCRIPTION FACTOR NSLP2) [Homo sapiens] | 1.51 | 0,0264 |
| A\_51\_P186887 | NM\_080562 | Mus musculus ubiquitin conjugating enzyme 7 interacting protein 5 (Ubce7ip5-pending), mRNA | 1.51 | 0,0113 |
| A\_51\_P352565 | NM\_024177 | Mus musculus mitochondrial ribosomal protein L38 (Mrpl38), mRNA | 1.51 | 0,0069 |
| A\_51\_P281380 | NM\_019571 | Mus musculus transmembrane 4 superfamily member 9 (Tm4sf9-pending), mRNA | 1.51 | 0,0485 |
| A\_51\_P489903 | NM\_010957 | Mus musculus 8-oxoguanine DNA-glycosylase 1 (Ogg1), mRNA | 1.51 | 0,0420 |
| A\_51\_P365932 | NM\_026250 | gross passage A viral integration region 1 | 1.51 | 0,0398 |
| A\_51\_P149562 | NM\_009686 | amyloid beta (A4) precursor protein-binding, family B, member 2 | 1.49 | 0,0442 |
| A\_51\_P501132 | NAP057261-1 | Mus musculus olfactory receptor MOR125-4P (MOR125-4P) pseudogene | 1.49 | 0,0163 |
| A\_51\_P115772 | AK081204 | unknown EST | 1.49 | 0,0334 |
| A\_51\_P395741 | XM\_132143 | weakly similar to SIGNAL RECOGNITION PARTICLE 72 KDA PROTEIN (SRP72) [Canis familiaris] | 1.49 | 0,0046 |
| A\_51\_P223177 | NM\_028791 | Mus musculus, RIKEN cDNA 1300018I05 gene, clone MGC:30380 IMAGE:3668655, mRNA, complete cds | 1.49 | 0,0081 |
| A\_51\_P493558 | NM\_145417 | Mus musculus, Similar to aminopeptidase B, clone MGC:29229 IMAGE:5041005, mRNA, complete cds | 1.49 | 0,0429 |
| A\_51\_P216516 | AK013596 | hypothetical protein | 1.49 | 0,0494 |
| A\_51\_P106144 | NM\_009564 | Mus musculus zinc finger protein 64 (Zfp64), mRNA | 1.49 | 0,0078 |
| A\_51\_P504053 | AK076973 | DNA segment, Chr 1, ERATO Doi 57, expressed | 1.49 | 0,0080 |
| A\_51\_P114459 | NM\_027537 | hypothetical protein | 1.49 | 0,0346 |
| A\_51\_P372895 | AK007242 | similar to ZINC FINGER PROTEIN (FRAGMENT) [Homo sapiens] | 1.49 | 0,0437 |
| A\_51\_P299155 | NM\_133937 | Mus musculus RIKEN cDNA 6720456B07 gene (6720456B07Rik), mRNA | 1.49 | 0,0226 |
| A\_51\_P349262 | NM\_013604 | Mus musculus metaxin (Mtx), mRNA | 1.49 | 0,0103 |
| A\_51\_P289862 | BC052065 | RAP1 GTPASE ACTIVATING PROTEIN 1 (RAP1GAP) homolog [Homo sapiens] | 1.49 | 0,0079 |
| A\_51\_P443084 | CK787647 | ATP BINDING CASSETTE, SUB FAMILY A, MEMBER ATP BINDING CASSETTE TRANSPORTER ATP BINDING CASSETTE | 1.49 | 0,0170 |
| A\_51\_P330369 | NM\_138744 | Mus musculus expressed sequence AU014939 (AU014939), mRNA | 1.49 | 0,0438 |
| A\_51\_P150087 | NM\_172511 | hypothetical protein | 1.49 | 0,0195 |
| A\_51\_P511707 | NM\_025636 | Mus musculus RIKEN cDNA 2310079N02 gene (2310079N02Rik), mRNA | 1.49 | 0,0016 |
| A\_51\_P421223 | NM\_010244 | Mus musculus Friend virus susceptibility 1 (Fv1), mRNA | 1.49 | 0,0276 |
| A\_51\_P487788 | AK080276 | BETA-2 ADRENERGIC RECEPTOR | 1.49 | 0,0013 |
| A\_51\_P512744 | NM\_025388 | Mus musculus RIKEN cDNA 1110021H02 gene (1110021H02Rik), mRNA | 1.49 | 0,0044 |
| A\_51\_P483659 | NM\_018880 | Mus musculus, Similar to ring finger protein 22, clone IMAGE:3481755, mRNA | 1.49 | 0,0133 |
| A\_51\_P295904 | NM\_028934 | Mus musculus cDNA, 3 end | 1.49 | 0,0278 |
| A\_51\_P414755 | NM\_026361 | CATENIN DELTA 2 NEURAL PLAKOPHILIN RELATED ARM REPEAT PROTEIN NPRAP | 1.49 | 0,0085 |
| A\_51\_P284318 | NM\_054082 | Mus musculus metastasis associated 3 (Mta3), mRNA | 1.49 | 0,0087 |
| A\_51\_P368545 | NM\_022419 | Mus musculus RIKEN cDNA 0910001L24 gene (0910001L24Rik), mRNA | 1.49 | 0,0406 |
| A\_51\_P247098 | AK046067 | hypothetical protein | 1.49 | 0,0111 |
| A\_51\_P472829 | NM\_145144 | Mus musculus, clone MGC:30545 IMAGE:5044495, mRNA, complete cds | 1.49 | 0,0323 |
| A\_51\_P360896 | NM\_029236 | hypothetical CheR-type MCP methyl-transferase containing protein | 1.49 | 0,0358 |
| A\_51\_P236197 | NM\_009088 | Mus musculus RNA polymerase 1-4 (194 kDa subunit) (Rpo1-4), mRNA | 1.49 | 0,0208 |
| A\_51\_P423000 | NM\_007417 | Mus musculus adrenergic receptor, alpha 2a (Adra2a), mRNA | 1.49 | 0,0332 |
| A\_51\_P217750 | NM\_019704 | Mus musculus placental protein 6 (Pp6-pending), mRNA | 1.49 | 0,0117 |
| A\_51\_P111586 | NM\_009319 | Mus musculus TAR (HIV) RNA binding protein 2 (Tarbp2), mRNA | 1.48 | 0,0161 |
| A\_51\_P383584 | XM\_619530 | hypothetical jmjC domain containing protein | 1.48 | 0,0192 |
| A\_51\_P245546 | NM\_194341 | Mus musculus, Similar to AP1 gamma subunit binding protein 1, clone IMAGE:4458940, mRNA | 1.48 | 0,0085 |
| A\_51\_P499876 | L20334 | Mouse EDG-like receptor mRNA, partial cds | 1.48 | 0,0014 |
| A\_51\_P311995 | AK049850 | inferred: APXL {Homo sapiens} | 1.48 | 0,0357 |
| A\_51\_P149025 | AK032094 | finger protein kox14 (fragment) homolog [Homo sapiens] | 1.48 | 0,0129 |
| A\_51\_P215066 | NM\_026519 | Mus musculus RIKEN cDNA 2610318K02 gene (2610318K02Rik), mRNA | 1.48 | 0,0013 |
| A\_51\_P434979 | NM\_029098 | LIPOCALIN-1 INTERACTING MEMBRANE RECEPTOR (LIPOCALIN-INTERACTING PROTEIN) homolog [Homo sapiens] | 1.48 | 0,0127 |
| A\_51\_P385178 | AK029507 | hypothetical ARM repeat structure containing protein | 1.48 | 0,0007 |
| A\_51\_P286460 | NM\_178187 | HISTONE H2A homolog [Homo sapiens] | 1.48 | 0,0019 |
| A\_51\_P440828 | NM\_026168 | PTX1 PROTEIN homolog [Homo sapiens] | 1.48 | 0,0429 |
| A\_51\_P304510 | NM\_145590 | Mus musculus, Similar to hypothetical protein FLJ13868, clone MGC:28903 IMAGE:4919869, mRNA, complete cds | 1.48 | 0,0061 |
| A\_51\_P376857 | NM\_029585 | hypothetical protein | 1.48 | 0,0016 |
| A\_51\_P382789 | NM\_027025 | similar to AD026 [Homo sapiens] | 1.48 | 0,0069 |
| A\_51\_P125446 | NM\_026963 | LEUCINE ZIPPER & ICAT HOMOLOGOUS PROTEIN LZIC homolog [Homo sapiens] | 1.48 | 0,0114 |
| A\_51\_P371521 | NM\_172562 | TRANSCRIPTIONAL ADAPTER 2 LIKE ADA2 LIKE PROTEIN | 1.48 | 0,0016 |
| A\_51\_P341349 | NM\_024433 | 5-methylthioadenosine phosphorylase (EC 2.4.2.28) homolog [Homo sapiens] | 1.48 | 0,0429 |
| A\_51\_P465871 | NM\_021448 | Mus musculus signal transducer and activator of transcription interacting protein 1 (Statip1), mRNA | 1.48 | 0,0164 |
| A\_51\_P469568 | NM\_020504 | Mus musculus claudin 13 (Cldn13), mRNA | 1.48 | 0,0018 |
| A\_51\_P378777 | NM\_029090 | Mus musculus, RIKEN cDNA 1200013P24 gene, clone MGC:6893 IMAGE:2654387, mRNA, complete cds | 1.48 | 0,0041 |
| A\_51\_P173235 | NM\_028134 | hypothetical LysM motif containing protein | 1.48 | 0,0047 |
| A\_51\_P190805 | NM\_007456 | Mus musculus adaptor-related protein complex AP-1, mu subunit 1 (Ap1m1), mRNA | 1.48 | 0,0083 |
| A\_51\_P335242 | AK047014 | SIMILAR TO INTEGRIN, BETA-LIKE 1 (WITH EGF-LIKE REPEAT DOMAINS) homolog [Mus musculus] | 1.48 | 0,0183 |
| A\_51\_P318375 | NM\_172480 | similar to METHIONINE SYNTHASE REDUCTASE [Homo sapiens] | 1.48 | 0,0136 |
| A\_51\_P212023 | NM\_016926 | Mus musculus squamous cell carcinoma antigen recognized by T-cells 3 (Sart3), mRNA | 1.48 | 0,0290 |
| A\_51\_P419078 | BC006684 | Mus musculus RIKEN cDNA 4833432B22 gene (4833432B22Rik), mRNA | 1.48 | 0,0039 |
| A\_51\_P423423 | BC089320 | Mus musculus cDNA, 5 end | 1.48 | 0,0197 |
| A\_51\_P517012 | AK005069 | hypothetical protein | 1.48 | 0,0034 |
| A\_51\_P518919 | NM\_172853 | CADHERIN-7 PRECURSOR homolog [Homo sapiens] | 1.48 | 0,0018 |
| A\_51\_P364372 | NM\_001005916 | hypothetical BTB/POZ domain containing protein | 1.48 | 0,0276 |
| A\_51\_P488422 | BC050879 | hypothetical Adenosine and AMP deaminase containing protein | 1.48 | 0,0011 |
| A\_51\_P369003 | NM\_021275 | Mus musculus potassium voltage-gated channel, shaker-related subfamily, member 4 (Kcna4), mRNA | 1.48 | 0,0375 |
| A\_51\_P274356 | NM\_147024 | Mus musculus olfactory receptor MOR135-2 (MOR135-2), mRNA | 1.47 | 0,0179 |
| A\_51\_P506148 | NM\_178911 | hypothetical protein | 1.47 | 0,0061 |
| A\_51\_P368381 | NM\_019778 | Mus musculus zinc finger protein 288 (Zfp288), mRNA | 1.47 | 0,0113 |
| A\_51\_P165834 | NM\_027539 | hypothetical Eukaryotic protein kinase/Doublecortin containing protein | 1.47 | 0,0231 |
| A\_51\_P435339 | NM\_010149 | Mus musculus erythropoietin receptor (Epor), mRNA | 1.47 | 0,0103 |
| A\_51\_P285300 | NM\_021351 | Mus musculus crystallin, beta A4 (Cryba4), mRNA | 1.47 | 0,0007 |
| A\_51\_P240536 | NM\_027159 | Mus musculus, clone MGC:25689 IMAGE:3491825, mRNA, complete cds | 1.47 | 0,0245 |
| A\_51\_P195135 | NM\_011462 | Mus musculus spindlin (Spin), mRNA | 1.47 | 0,0224 |
| A\_51\_P408059 | NM\_026532 | Mus musculus nuclear transport factor 2 (Nutf2), mRNA | 1.47 | 0,0185 |
| A\_51\_P321651 | NM\_028221 | Mus musculus expressed sequence AI596259 (AI596259), mRNA | 1.47 | 0,0076 |
| A\_51\_P123705 | NM\_144858 | Mus musculus putative zinc finger protein mRNA, complete cds | 1.47 | 0,0039 |
| A\_51\_P355773 | NM\_026921 | Mus musculus, RIKEN cDNA 1810010A06 gene, clone MGC:19368 IMAGE:2615786, mRNA, complete cds | 1.47 | 0,0335 |
| A\_51\_P270650 | NM\_207558 | Mus musculus olfactory receptor GA\_x5J8B7W5WBF-6267395-6266441 (GA\_x5J8B7W5WBF-6267395-6266441) pseudogene | 1.47 | 0,0115 |
| A\_51\_P390744 | NM\_146474 | Mus musculus olfactory receptor MOR234-3 (MOR234-3), mRNA | 1.47 | 0,0066 |
| A\_51\_P357996 | NM\_013756 | Mus musculus defensin beta 3 (Defb3), mRNA | 1.47 | 0,0030 |
| A\_51\_P139462 | AK047901 | transcription factor ATF-a homolog [Homo sapiens] | 1.47 | 0,0213 |
| A\_51\_P284891 | NM\_011373 | Mus musculus sialyltransferase 7 ((alpha-N-acetylneuraminyl 2,3-betagalactosyl-1,3)-N-acetyl galactosaminide alpha-2,6-sialyltransferase) D (Siat7d), mRNA | 1.47 | 0,0197 |
| A\_51\_P394074 | NM\_026255 | Mus musculus RIKEN cDNA 4930433D19 gene (4930433D19Rik), mRNA | 1.47 | 0,0039 |
| A\_51\_P426994 | NM\_153591 | hypothetical Aminoacyl-transfer RNA synthetases class-II containing protein | 1.47 | 0,0138 |
| A\_51\_P291819 | NM\_024210 | Mus musculus RIKEN cDNA 2310033P09 gene (2310033P09Rik), mRNA | 1.47 | 0,0375 |
| A\_51\_P283774 | NM\_028199 | Mus musculus tumor endothelial marker 7 precursor (Tem7) mRNA, complete cds | 1.47 | 0,0318 |
| A\_51\_P372585 | NM\_019742 | Mus musculus fusion 1 (Fus1-pending), mRNA | 1.47 | 0,0163 |
| A\_51\_P448127 | NM\_025890 | hypothetical Alanine-rich region containing protein | 1.47 | 0,0008 |
| A\_51\_P518758 | NM\_010598 | Mus musculus potassium voltage-gated channel, shaker-related subfamily, beta member 2 (Kcnab2), mRNA | 1.46 | 0,0260 |
| A\_51\_P508191 | NM\_007834 | Mus musculus Down syndrome critical region gene a (Dcra), mRNA | 1.46 | 0,0364 |
| A\_51\_P123066 | AK016133 | hypothetical protein | 1.46 | 0,0170 |
| A\_51\_P197209 | NM\_133770 | Mus musculus RIKEN cDNA 0610012P18 gene (0610012P18Rik), mRNA | 1.46 | 0,0253 |
| A\_51\_P486939 | NM\_133710 | Mus musculus RIKEN cDNA 2810418J22 gene (2810418J22Rik), mRNA | 1.46 | 0,0058 |
| A\_51\_P501107 | NM\_027807 | VASOPRESSIN-ACTIVATED CALCIUM-MOBILIZING RECEPTOR (VACM-1) (CULLIN HOMOLOG 5) (CUL-5) homolog [Rattus norvegicus] | 1.46 | 0,0497 |
| A\_51\_P278103 | NM\_024239 | associated molecule with the SH3 domain of STAM | 1.46 | 0,0353 |
| A\_51\_P410286 | NM\_172132 | Mus musculus, Similar to KIAA0677 gene product, clone MGC:6349 IMAGE:3490671, mRNA, complete cds | 1.46 | 0,0202 |
| A\_51\_P255360 | NM\_026004 | Mus musculus RIKEN cDNA 2610206B05 gene (2610206B05Rik), mRNA | 1.46 | 0,0006 |
| A\_51\_P469221 | NM\_178624 | LEUCINE-RICH REPEATS CONTAINING F-BOX PROTEIN FBL3 homolog [Homo sapiens] | 1.46 | 0,0377 |
| A\_51\_P180855 | NAP057133-1 | Mus musculus olfactory receptor GA\_x5J8B7W3Y5M-51270-50422 (GA\_x5J8B7W3Y5M-51270-50422) pseudogene | 1.46 | 0,0008 |
| A\_51\_P320552 | NM\_028730 | similar to KAIA2502 PROTEIN [Homo sapiens] | 1.46 | 0,0187 |
| A\_51\_P483370 | NM\_145367 | Mus musculus, Similar to hypothetical protein MGC3178, clone MGC:28887 IMAGE:4911455, mRNA, complete cds | 1.46 | 0,0393 |
| A\_51\_P158772 | NM\_170777 | hypothetical protein | 1.46 | 0,0213 |
| A\_51\_P257978 | NM\_021323 | Mus musculus ubiquitin specific protease 29 (Usp29), mRNA | 1.46 | 0,0223 |
| A\_51\_P400216 | NM\_016982 | Mus musculus pre-B lymphocyte gene 1 (Vpreb1), mRNA | 1.46 | 0,0081 |
| A\_51\_P104670 | NM\_013481 | block of proliferation 1 | 1.46 | 0,0102 |
| A\_51\_P222522 | NM\_023403 | Mus musculus mesoderm development candiate 2 (Mesdc2), mRNA | 1.46 | 0,0116 |
| A\_51\_P287232 | NM\_133794 | GLUTAMINYL-TRNA SYNTHETASE (EC 6.1.1.18) (GLUTAMINE--TRNA LIGASE) (GLNRS) homolog [Homo sapiens] | 1.46 | 0,0210 |
| A\_51\_P518298 | AK075944 | Mus musculus mRNA, complete cds, clone:2-24 | 1.46 | 0,0260 |
| A\_51\_P161724 | NM\_052976 | Mus musculus oligophrenin 1 (Ophn1), mRNA | 1.46 | 0,0495 |
| A\_51\_P442990 | NM\_028233 | similar to leucine-rich protein [Homo sapiens] | 1.46 | 0,0094 |
| A\_51\_P402974 | BC026389 | Mus musculus, clone IMAGE:4190185, mRNA, partial cds | 1.46 | 0,0296 |
| A\_51\_P494825 | XM\_356068 | MASL1 {Homo sapiens} | 1.46 | 0,0051 |
| A\_51\_P247249 | NM\_009662 | arachidonate 5-lipoxygenase | 1.46 | 0,0223 |
| A\_51\_P338837 | NM\_030695 | Mus musculus LPS-responsive beige-like anchor (Lrba), mRNA | 1.46 | 0,0055 |
| A\_51\_P487162 | NM\_028002 | PP35 homolog [Homo sapiens] | 1.46 | 0,0404 |
| A\_51\_P507970 | NM\_025686 | BRF2, subunit of RNA polymerase III transcription initiation factor, BRF1-like | 1.46 | 0,0332 |
| A\_51\_P206153 | D13903 | Mouse mRNA for MPTPdelta (type A) | 1.46 | 0,0181 |
| A\_51\_P491976 | NM\_175401 | hypothetical Ribosomal protein S2 containing protein | 1.46 | 0,0297 |
| A\_51\_P313703 | NM\_008639 | Mus musculus melatonin receptor 1A (Mtnr1a), mRNA | 1.46 | 0,0095 |
| A\_51\_P154933 | NM\_133967 | Mus musculus zinc finger, DHHC domain containing 7 (Zdhhc7), mRNA | 1.46 | 0,0453 |
| A\_51\_P439403 | NM\_011059 | Mus musculus peptidyl arginine deiminase, type I (Pdi1), mRNA | 1.46 | 0,0231 |
| A\_51\_P248387 | NM\_172832 | hypothetical FAD/NAD(P)-binding domain structure containing protein | 1.46 | 0,0024 |
| A\_51\_P167932 | NM\_177921 | hypothetical protein | 1.46 | 0,0004 |
| A\_51\_P111068 | NM\_146734 | Mus musculus olfactory receptor MOR204-13 (MOR204-13), mRNA | 1.45 | 0,0036 |
| A\_51\_P309293 | AK129163 | Mus musculus, clone IMAGE:1378878, mRNA | 1.45 | 0,0376 |
| A\_51\_P154946 | AK082737 | WEAKLY SIMILAR TO L- RIBULOKINASE homolog [Homo sapiens] | 1.45 | 0,0005 |
| A\_51\_P300888 | AK006690 | unknown EST | 1.45 | 0,0058 |
| A\_51\_P509211 | NM\_030564 | Mus musculus ring finger protein 34 (Rnf34), mRNA | 1.45 | 0,0107 |
| A\_51\_P344051 | NM\_134029 | Mus musculus 5,3-nucleotidase, mitochondrial (Nt5m), mRNA | 1.45 | 0,0189 |
| A\_51\_P431888 | NM\_178702 | hypothetical Gram-positive cocci surface protein anchoring hexapeptide/RA domain/PDZ domain (also known as DHR or GLGF) containing protein | 1.45 | 0,0110 |
| A\_51\_P179953 | NM\_027922 | hypothetical Ankyrin-repeat containing protein | 1.45 | 0,0405 |
| A\_51\_P462746 | BC006867 | Mus musculus, clone MGC:11792 IMAGE:3595167, mRNA, complete cds | 1.45 | 0,0226 |
| A\_51\_P279623 | NM\_030240 | Mus musculus, clone MGC:7316 IMAGE:3485982, mRNA, complete cds | 1.45 | 0,0126 |
| A\_51\_P385958 | NM\_172257 | Mus musculus, Similar to hypothetical protein FLJ20174, clone IMAGE:3595651, mRNA, partial cds | 1.45 | 0,0398 |
| A\_51\_P274667 | XM\_129972 | hypothetical Zn-finger CCHC type containing protein | 1.45 | 0,0191 |
| A\_51\_P117165 | AK018150 | hypothetical protein | 1.45 | 0,0091 |
| A\_51\_P228625 | A\_51\_P228625 | Similar to: Mus musculus perlecan (heparan sulfate proteoglycan 2) (Hspg2), mRNA | 1.45 | 0,0066 |
| A\_51\_P452702 | AK006382 | hypothetical protein | 1.45 | 0,0058 |
| A\_51\_P438527 | NM\_028057 | NADH CYTOCHROME B5 REDUCTASE | 1.45 | 0,0349 |
| A\_51\_P501315 | AK004720 | Mus musculus gene trap locus F3b (Gtlf3b), mRNA | 1.45 | 0,0080 |
| A\_51\_P448677 | NM\_008790 | Mus musculus Purkinje cell protein 2 (L7) (Pcp2), mRNA | 1.45 | 0,0092 |
| A\_51\_P250207 | NAP057197-1 | Mus musculus olfactory receptor MOR239-8P (MOR239-8P) pseudogene | 1.45 | 0,0066 |
| A\_51\_P472488 | NM\_019836 | Mus musculus RIKEN cDNA 2610024G14 gene (2610024G14Rik), mRNA | 1.45 | 0,0065 |
| A\_51\_P492389 | NM\_172462 | zinc finger protein 11 | 1.45 | 0,0008 |
| A\_51\_P437717 | NM\_020003 | Mus musculus RIKEN cDNA 0610031J06 gene (0610031J06Rik), mRNA | 1.45 | 0,0089 |
| A\_51\_P185508 | AK087318 | unknown EST | 1.45 | 0,0240 |
| A\_51\_P207451 | NM\_199062 | weakly similar to DNA-BINDING PROTEIN (FRAGMENT) [Homo sapiens] | 1.45 | 0,0251 |
| A\_51\_P414126 | NM\_011226 | RAB19, member RAS oncogene family | 1.45 | 0,0008 |
| A\_51\_P513846 | NM\_026319 | Mus musculus RIKEN cDNA 1700029H06 gene (1700029H06Rik), mRNA | 1.45 | 0,0186 |
| A\_51\_P174552 | NM\_175004 | similar to HYPOTHETICAL PROTEIN CGI-147 [Homo sapiens] | 1.45 | 0,0271 |
| A\_51\_P344346 | XM\_144699 | hypothetical Cysteine-rich region containing protein | 1.45 | 0,0097 |
| A\_51\_P328950 | AK043018 | hypothetical Second domain of Mu2 adaptin subunit (ap50) of ap2 adaptor structure containing protein | 1.45 | 0,0376 |
| A\_51\_P169128 | NM\_025879 | Mus musculus RIKEN cDNA 2410002O22 gene (2410002O22Rik), mRNA | 1.45 | 0,0363 |
| A\_51\_P440865 | NM\_173426 | hypothetical Microbodies C-terminal targeting signal containing protein | 1.45 | 0,0144 |
| A\_51\_P205965 | NM\_026912 | SORTING NEXIN | 1.45 | 0,0034 |
| A\_51\_P112639 | NM\_009210 | Mus musculus SWI/SNF related, matrix associated, actin dependent regulator of chromatin, subfamily a, member 3 (Smarca3), mRNA | 1.45 | 0,0080 |
| A\_51\_P415931 | NM\_172844 | weakly similar to DIMETHYLANILINE MONOOXYGENASE [N-OXIDE FORMING] 5 (EC 1.14.13.8) (HEPATIC FLAVIN-CONTAINING MONOOXYGENASE 5) (FMO 5) (DIMETHYLANILINE OXIDASE 5) (FMO 1C1) (FMO FORM 3) [Oryctolagus cuniculus] | 1.45 | 0,0016 |
| A\_51\_P201635 | NM\_011676 | Mus musculus unc119 homolog (C. elegans) (Unc119h), mRNA | 1.44 | 0,0083 |
| A\_51\_P383320 | NM\_009543 | Mus musculus zinc finger protein 103 (Zfp103), mRNA | 1.44 | 0,0193 |
| A\_51\_P116581 | NM\_138603 | Mus musculus DNA segment, Chr X, Immunex 40, expressed (DXImx40e), mRNA | 1.44 | 0,0251 |
| A\_51\_P256642 | NM\_146145 | Mus sp. JAK1 protein tyrosine kinase mRNA, complete cds | 1.44 | 0,0198 |
| A\_51\_P325318 | NM\_172702 | FKSG84 homolog [Homo sapiens] | 1.44 | 0,0080 |
| A\_51\_P518462 | AK034138 | weakly similar to ETAA16 PROTEIN [Homo sapiens] | 1.44 | 0,0202 |
| A\_51\_P178646 | NM\_026308 | Mus musculus RIKEN cDNA 0610037N01 gene (0610037N01Rik), mRNA | 1.44 | 0,0187 |
| A\_51\_P431517 | XM\_355743 | Mus musculus Dlx-6 mRNA, partial cds | 1.44 | 0,0148 |
| A\_51\_P462918 | NM\_023737 | Mus musculus RIKEN cDNA 1300002P22 gene (1300002P22Rik), mRNA | 1.44 | 0,0163 |
| A\_51\_P385849 | NM\_177307 | weakly similar to CYTOCHROME P450 4F2 (EC 1.14.13.30) (CYPIVF2) (LEUKOTRIENE-B4 OMEGA- HYDROXYLASE) (LEUKOTRIENE-B4 20-MONOOXYGENASE) (CYTOCHROME P450- LTB-OMEGA) [Homo sapiens] | 1.44 | 0,0264 |
| A\_51\_P177364 | NM\_020501 | Mus musculus candidate taste receptor T2R5 gene (T2r5), mRNA | 1.44 | 0,0333 |
| A\_51\_P306885 | NM\_025595 | Mus musculus mitochondrial ribosomal protein 64 (Mrp64), mRNA | 1.44 | 0,0275 |
| A\_51\_P331589 | A\_51\_P331589 | Mus musculus cDNA, 5 end | 1.44 | 0,0097 |
| A\_51\_P323701 | NM\_146597 | Mus musculus olfactory receptor MOR260-4 (MOR260-4), mRNA | 1.44 | 0,0055 |
| A\_51\_P157737 | NM\_172483 | similar to CYS2/HIS2 ZINC FINGER PROTEIN [Rattus norvegicus] | 1.44 | 0,0132 |
| A\_51\_P440807 | NM\_007760 | Mus musculus carnitine acetyltransferase (Crat), mRNA | 1.44 | 0,0409 |
| A\_51\_P182876 | D86419 | Mus musculus mRNA for glycine tyrosine-rich hair keratin protein, complete cds | 1.44 | 0,0044 |
| A\_51\_P221930 | AK083920 | CDNA FLJ30803 FIS, CLONE FEBRA2001245, WEAKLY SIMILAR TO NAG14 homolog [Homo sapiens] | 1.44 | 0,0057 |
| A\_51\_P394676 | BC040217 | unknown EST | 1.44 | 0,0128 |
| A\_51\_P407480 | NM\_008665 | Mus musculus myelin transcription factor 1 (Myt1), mRNA | 1.44 | 0,0361 |
| A\_51\_P216549 | NM\_011641 | Mus musculus transformation related protein 63 (Trp63), mRNA | 1.44 | 0,0025 |
| A\_51\_P425352 | NM\_177049 | CDNA FLJ30783 FIS, CLONE FEBRA2000880, WEAKLY SIMILAR TO SARCALUMENIN PRECURSOR homolog [Homo sapiens] | 1.44 | 0,0133 |
| A\_51\_P313635 | NM\_134420 | Mus musculus solute carrier family 26, member 6 (Slc26a6), mRNA | 1.44 | 0,0366 |
| A\_51\_P468743 | NM\_025697 | CLLL6 PROTEIN homolog [Homo sapiens] | 1.44 | 0,0263 |
| A\_51\_P132233 | AK032440 | unknown EST | 1.44 | 0,0277 |
| A\_51\_P457397 | AK009140 | unknown EST | 1.44 | 0,0075 |
| A\_51\_P220407 | NM\_181405 | similar to RNPEP-LIKE PROTEIN [Homo sapiens] | 1.44 | 0,0491 |
| A\_51\_P395864 | AK021106 | unknown EST | 1.44 | 0,0304 |
| A\_51\_P155142 | NM\_026560 | similar to HYPOTHETICAL 31.3 KDA PROTEIN [Homo sapiens] | 1.44 | 0,0011 |
| A\_51\_P106347 | NM\_183146 | similar to SIMILAR TO ZINC FINGER PROTEIN 85 (HPF4, HTF1) [Mus musculus] | 1.44 | 0,0494 |
| A\_51\_P514131 | M35436 | Mouse T-cell receptor delta-chain mRNA, D2J1-region | 1.44 | 0,0141 |
| A\_51\_P367011 | NM\_207236 | OLFACTORY RECEPTOR | 1.44 | 0,0021 |
| A\_51\_P116946 | NM\_080467 | Mus musculus ATPase, H+ transporting, lysosomal (vacuolar proton pump) noncatalytic accessory protein 1B (Atp6n1b), mRNA | 1.44 | 0,0151 |
| A\_51\_P119841 | NM\_010491 | Mus musculus islet amyloid polypeptide (Iapp), mRNA | 1.43 | 0,0067 |
| A\_51\_P230850 | NM\_018807 | Mus musculus pleiomorphic adenoma gene-like 2 (Plagl2), mRNA | 1.43 | 0,0103 |
| A\_51\_P241577 | NM\_027649 | hypothetical Serine-rich region containing protein | 1.43 | 0,0016 |
| A\_51\_P228149 | NM\_009633 | Mus musculus adrenergic receptor, alpha 2b (Adra2b), mRNA | 1.43 | 0,0320 |
| A\_51\_P509389 | NM\_031868 | Mus musculus protein phosphatase 1, catalytic subunit, alpha isoform (Ppp1ca), mRNA | 1.43 | 0,0239 |
| A\_51\_P199871 | NM\_023371 | Mus musculus protein (peptidyl-prolyl cis/trans isomerase) NIMA-interacting 1 (Pin1), mRNA | 1.43 | 0,0084 |
| A\_51\_P322989 | CB247354 | weakly similar to CG14903 PROTEIN [Drosophila melanogaster] | 1.43 | 0,0306 |
| A\_51\_P205480 | NM\_144843 | MYOTUBULARIN RELATED PROTEIN 6 homolog [Homo sapiens] | 1.43 | 0,0099 |
| A\_51\_P404204 | NM\_029780 | Mus musculus v-raf-1 leukemia viral oncogene 1 (Raf1), mRNA | 1.43 | 0,0223 |
| A\_51\_P353202 | BC087904 | Mus musculus olfactory receptor MOR261-8P (MOR261-8P) pseudogene | 1.43 | 0,0018 |
| A\_51\_P117035 | NM\_010769 | Mus musculus matrilin 1, cartilage matrix protein 1 (Matn1), mRNA | 1.43 | 0,0248 |
| A\_51\_P363668 | NM\_011276 | Mus musculus Hoxa1 regulated gene (Ha1r-pending), mRNA | 1.43 | 0,0319 |
| A\_51\_P452820 | NM\_053257 | Mus musculus ribosomal protein L31 (Rpl31), mRNA | 1.43 | 0,0206 |
| A\_51\_P168439 | AK004187 | unknown EST | 1.43 | 0,0285 |
| A\_51\_P127949 | AK042690 | TRANSCRIPTION FACTOR NRF homolog [Homo sapiens] | 1.43 | 0,0061 |
| A\_51\_P268469 | NM\_053144 | Mus musculus protocadherin beta 19 (Pcdhb19), mRNA | 1.43 | 0,0106 |
| A\_51\_P510418 | NM\_028270 | Mus musculus, aldehyde dehydrogenase 1 family, member B1, clone MGC:28561 IMAGE:4207479, mRNA, complete cds | 1.43 | 0,0221 |
| A\_51\_P239673 | NM\_013556 | Mus musculus hypoxanthine guanine phosphoribosyl transferase (Hprt), mRNA | 1.43 | 0,0375 |
| A\_51\_P118284 | NM\_013723 | Mus musculus podocalyxin-like (Podxl), mRNA | 1.43 | 0,0033 |
| A\_51\_P503822 | NM\_175499 | hypothetical Cysteine-rich flanking region, C-terminal/Leucine-rich repeat/Leucine-rich repeat, typical subtype containing protein | 1.43 | 0,0177 |
| A\_51\_P133072 | A\_51\_P133072 | Mus musculus cDNA, 5 end | 1.43 | 0,0005 |
| A\_51\_P364188 | BC027085 | unknown | 1.43 | 0,0207 |
| A\_51\_P251209 | BC058783 | VOLTAGE-GATED CALCIUM CHANNEL PORE FORMING SUBUNIT CAV1.3 ALPHA1D (FRAGMENT) homolog [Rattus norvegicus] | 1.43 | 0,0420 |
| A\_51\_P150394 | NM\_025842 | Mus musculus vacuolar protein sorting 28 (yeast) (Vps28), mRNA | 1.42 | 0,0181 |
| A\_51\_P204504 | NM\_013925 | Mus musculus adenosine deaminase, tRNA-specific 1 (Adat1), mRNA | 1.42 | 0,0131 |
| A\_51\_P160241 | NM\_146370 | Mus musculus olfactory receptor B12 mRNA, partial cds | 1.42 | 0,0069 |
| A\_51\_P119597 | NM\_009680 | Mus musculus adaptor-related protein complex AP-3, beta 1 subunit (Ap3b1), mRNA | 1.42 | 0,0474 |
| A\_51\_P313337 | AK077261 | unknown EST | 1.42 | 0,0203 |
| A\_51\_P417399 | NM\_026468 | ATP synthase, H+ transporting, mitochondrial F0 complex, subunit c (subunit 9), isoform 2 | 1.42 | 0,0119 |
| A\_51\_P320190 | XM\_128010 | Mus musculus, clone IMAGE:4210825, mRNA, partial cds | 1.42 | 0,0275 |
| A\_51\_P352720 | XM\_132983 | Mus musculus cardiac leiomodin mRNA, partial cds | 1.42 | 0,0247 |
| A\_51\_P280434 | NM\_133649 | Mus musculus solute carrier family 12, member 6 (Slc12a6), mRNA | 1.42 | 0,0346 |
| A\_51\_P414448 | NM\_053089 | Mus musculus NMDA receptor-regulated gene 1 (Narg1-pending), mRNA | 1.42 | 0,0390 |
| A\_51\_P341844 | NAP057100-1 | Mus musculus olfactory receptor GA\_x5J8B7W60AJ-703922-704828 (GA\_x5J8B7W60AJ-703922-704828) pseudogene | 1.42 | 0,0312 |
| A\_51\_P219064 | NM\_133182 | Mus musculus heterogeneous nuclear ribonucleoprotein methyltransferase-like 1 (S. cerevisiae) (Hrmt1l1), mRNA | 1.42 | 0,0103 |
| A\_51\_P471458 | NM\_020564 | Mus musculus sulfotransferase-related protein SULT-X1 (Sult-x1), mRNA | 1.42 | 0,0164 |
| A\_51\_P426214 | NM\_009271 | Mus musculus Rous sarcoma oncogene (Src), mRNA | 1.42 | 0,0240 |
| A\_51\_P246543 | AK220542 | unknown EST | 1.42 | 0,0260 |
| A\_51\_P415413 | NM\_021892 | Mus musculus RFamide-related peptide (Rfrp), mRNA | 1.42 | 0,0054 |
| A\_51\_P286373 | NM\_183017 | DJ526I14.2 (KIAA0153 (SIMILAR TO WORM D2013.9)) (KIAA0153 PROTEIN) homolog [Homo sapiens] | 1.42 | 0,0090 |
| A\_51\_P162207 | NM\_011627 | Mus musculus trophoblast glycoprotein (Tpbg), mRNA | 1.42 | 0,0362 |
| A\_51\_P359445 | NM\_030219 | hypothetical B-box zinc finger domain/TNFR/CD27/30/40/95 cysteine-rich region/Fibronectin type III domain/RING finger containing protein | 1.42 | 0,0337 |
| A\_51\_P450088 | XM\_484897 | Mus musculus collagen alpha 3 chain type VI | 1.42 | 0,0234 |
| A\_51\_P237076 | NM\_146012 | NUCLEAR LIM INTERACTOR-INTERACTING FACTOR 2 (NLI-INTERACTING FACTOR 2) (PROTEIN OS-4) homolog [Homo sapiens] | 1.42 | 0,0489 |
| A\_51\_P179741 | NM\_172656 | AMYOTROPHIC LATERAL SCLEROSIS 2 (CDNA FLJ14731 FIS, CLONE NT2RP3001938, WEAKLY SIMILAR TO SPORULATION-SPECIFIC PROTEIN 1) (AMYOTROPHIC LATERAL SCLEROSIS 2 (JUVENILE) CHROMOSOME REGION, CANDIDATE 2) (EC 2.7.1.-) homolog [Homo sapiens] | 1.42 | 0,0102 |
| A\_51\_P215077 | NM\_025569 | Mus musculus microsomal glutathione S-transferase 3 (Mgst3), mRNA | 1.42 | 0,0183 |
| A\_51\_P326086 | NM\_011293 | Mus musculus RNA polymerase II 4 (14 kDa subunit) (Rpo2-4), mRNA | 1.42 | 0,0202 |
| A\_51\_P502133 | NM\_011985 | Mus musculus matrix metalloproteinase 23 (Mmp23), mRNA | 1.42 | 0,0061 |
| A\_51\_P292630 | BC027189 | weakly similar to URIDINE PHOSPHORYLASE (EC 2.4.2.3) (UDRPASE) [Mus musculus] | 1.42 | 0,0115 |
| A\_51\_P288505 | XM\_134502 | similar to TNF RECEPTOR TYPE 1 ASSOCIATED DEATH DOMAIN PROTEIN | 1.42 | 0,0091 |
| A\_51\_P108645 | NM\_010587 | Mus musculus intersectin (SH3 domain protein 1A) (Itsn), mRNA | 1.42 | 0,0112 |
| A\_51\_P242978 | XM\_284451 | hypothetical protein | 1.42 | 0,0201 |
| A\_51\_P513979 | NM\_027283 | hypothetical protein | 1.41 | 0,0005 |
| A\_51\_P318281 | NM\_031186 | Mus musculus N-deacetylase/N-sulfotransferase (heparan glucosaminyl) 3 (Ndst3), mRNA | 1.41 | 0,0008 |
| A\_51\_P360622 | NM\_144917 | similar to PP4068 [Homo sapiens] | 1.41 | 0,0306 |
| A\_51\_P224592 | NM\_026823 | Mus musculus, clone MGC:28025 IMAGE:3661329, mRNA, complete cds | 1.41 | 0,0320 |
| A\_51\_P454884 | L10908 | Mus musculus Gcap1 mRNA, partial cds | 1.41 | 0,0351 |
| A\_51\_P281593 | BC062173 | weakly similar to HTPAP [Homo sapiens] | 1.41 | 0,0305 |
| A\_51\_P180654 | AK129084 | hypothetical protein | 1.41 | 0,0059 |
| A\_51\_P133156 | A\_51\_P133156 | Mus musculus cDNA, 3 end | 1.41 | 0,0341 |
| A\_51\_P362879 | NM\_134136 | similar to 5-HT RECEPTOR [Homo sapiens] | 1.41 | 0,0175 |
| A\_51\_P116298 | AK006122 | similar to BA305P22.2.5 (NOVEL PROTEIN, ISOFORM 5) (FRAGMENT) [Homo sapiens] | 1.41 | 0,0101 |
| A\_51\_P191893 | AK054069 | hypothetical protein | 1.41 | 0,0096 |
| A\_51\_P402416 | NM\_025504 | Mus musculus RIKEN cDNA 2310004L02 gene (2310004L02Rik), mRNA | 1.41 | 0,0084 |
| A\_51\_P299287 | NM\_053261 | Mus musculus inositol (myo)-1(or 4)-monophosphatase 2 (Impa2), mRNA | 1.41 | 0,0268 |
| A\_51\_P103509 | NM\_019720 | Mus musculus tumor suppressor region 10 (Tsp10-pending), mRNA | 1.41 | 0,0178 |
| A\_51\_P376484 | AK078508 | Mus musculus cDNA, 3 end | 1.41 | 0,0347 |
| A\_51\_P439452 | NM\_133748 | Mus musculus RIKEN cDNA 2900053I11 gene (2900053I11Rik), mRNA | 1.41 | 0,0194 |
| A\_51\_P386562 | NM\_010095 | Mus musculus early B-cell factor 2 (Ebf2), mRNA | 1.41 | 0,0009 |
| A\_51\_P438039 | NM\_183272 | unknown EST | 1.41 | 0,0062 |
| A\_51\_P356512 | NM\_026139 | Mus musculus RIKEN cDNA 3230401N03 gene (3230401N03Rik), mRNA | 1.41 | 0,0228 |
| A\_51\_P384314 | AK038399 | unknown EST | 1.41 | 0,0319 |
| A\_51\_P121797 | A\_51\_P121797 | Mus musculus cDNA, 5 end | 1.41 | 0,0137 |
| A\_51\_P291749 | NM\_023523 | Mus musculus peroxisomal trans-2-enoyl-CoA reductase (Pecr), mRNA | 1.41 | 0,0056 |
| A\_51\_P448114 | AK014599 | similar to HYALURONIDASE 4 [Homo sapiens] | 1.41 | 0,0365 |
| A\_51\_P350996 | NM\_080461 | Mus musculus zinc finger protein 358 (Zfp358), mRNA | 1.41 | 0,0338 |
| A\_51\_P112529 | NM\_011256 | Mus musculus retinal degeneration B2 homolog (Drosophila) (Rdgb2), mRNA | 1.41 | 0,0030 |
| A\_51\_P509083 | NM\_010214 | Mus musculus four and a half LIM domains 4 (Fhl4), mRNA | 1.41 | 0,0249 |
| A\_51\_P101582 | NM\_018875 | Mus musculus sorting nexin 12 (Snx12), mRNA | 1.41 | 0,0108 |
| A\_51\_P288479 | NM\_181328 | hypothetical Mitochondrial energy transfer proteins (carrier protein) containing protein | 1.41 | 0,0278 |
| A\_51\_P385718 | NM\_026862 | hypothetical Prenyl group binding site (CAAX box) containing protein | 1.41 | 0,0282 |
| A\_51\_P185499 | NM\_008923 | Mus musculus protein kinase, cAMP dependent regulatory, type I beta (Prkar1b), mRNA | 1.41 | 0,0060 |
| A\_51\_P284946 | NM\_028810 | Mus musculus ras homolog gene family, member E (Arhe), mRNA | 1.41 | 0,0318 |
| A\_51\_P158552 | NM\_016684 | Mus musculus zinc finger protein 96 (Zfp96) mRNA, complete cds | 1.41 | 0,0382 |
| A\_51\_P396635 | AK037839 | mitochondria located 1 homolog (human) | 1.41 | 0,0105 |
| A\_51\_P352606 | NM\_009782 | Mus musculus calcium channel, voltage-dependent, R type, alpha 1E subunit (Cacna1e), mRNA | 1.41 | 0,0266 |
| A\_51\_P505230 | NM\_025332 | hypothetical P-loop containing nucleotide triphosphate hydrolases structure containing protein | 1.40 | 0,0052 |
| A\_51\_P168945 | NM\_016985 | Mus musculus myotubularin related protein 1 (Mtmr1), mRNA | 1.40 | 0,0434 |
| A\_51\_P393968 | NM\_007701 | Mus musculus C. elegans ceh-10 homeo domain containing homolog (Chx10), mRNA | 1.40 | 0,0176 |
| A\_51\_P295446 | NM\_134100 | Mus musculus expressed sequence AW556797 (AW556797), mRNA | 1.40 | 0,0342 |
| A\_51\_P242316 | NM\_133818 | Mus musculus expressed sequence AI597479 (AI597479), mRNA | 1.40 | 0,0127 |
| A\_51\_P355981 | NM\_027844 | Mus musculus RIKEN cDNA 4833428E21 gene (4833428E21Rik), mRNA | 1.40 | 0,0025 |
| A\_51\_P381749 | NM\_025531 | Mus musculus RIKEN cDNA 2310042G06 gene (2310042G06Rik), mRNA | 1.40 | 0,0457 |
| A\_51\_P136143 | NM\_011786 | Mus musculus arachidonate lipoxygenase 3 (Aloxe3), mRNA | 1.40 | 0,0104 |
| A\_51\_P380069 | NM\_198962 | OREXIN RECEPTOR TYPE 2 | 1.40 | 0,0323 |
| A\_51\_P471235 | NM\_001011828 | Mus musculus olfactory receptor MOR219-3P (MOR219-3P) pseudogene | 1.40 | 0,0025 |
| A\_51\_P427762 | NM\_007858 | Mus musculus diaphanous homolog 1 (Drosophila) (Diap1), mRNA | 1.40 | 0,0012 |
| A\_51\_P162144 | NM\_021879 | Mus musculus pink-eyed dilution (p), mRNA | 1.40 | 0,0183 |
| A\_51\_P153173 | AK021349 | unknown EST | 1.40 | 0,0150 |
| A\_51\_P161200 | AK030281 | hypothetical Eukaryotic thiol (cysteine) proteases active site/Trp-Asp (WD) repeats circular profile/G-protein beta WD-40 repeats containing protein | 1.40 | 0,0110 |
| A\_51\_P107520 | NM\_181818 | Mus musculus olfactory receptor MOR179-5 (MOR179-5) pseudogene | 1.40 | 0,0012 |
| A\_51\_P215955 | NM\_025944 | hypothetical protein | 1.40 | 0,0069 |
| A\_51\_P308447 | AK087556 | ubiquitin-like 1 (sentrin) activating enzyme E1A | 1.40 | 0,0434 |
| A\_51\_P426886 | NM\_008155 | Mus musculus glucose phosphate isomerase 1 complex (Gpi1), mRNA | 1.40 | 0,0488 |
| A\_51\_P467076 | NM\_010000 | Mus musculus cytochrome P450, 2b9, phenobarbitol inducible, type a (Cyp2b9), mRNA | 1.40 | 0,0058 |
| A\_51\_P105338 | NM\_172609 | MITOCHONDRIAL IMPORT RECEPTOR SUBUNIT TOM22 HOMOLOG | 1.40 | 0,0424 |
| A\_51\_P227165 | NM\_025865 | Mus musculus RIKEN cDNA 2310030G06 gene (2310030G06Rik), mRNA | 1.40 | 0,0073 |
| A\_51\_P488389 | AK080053 | unclassifiable | 1.40 | 0,0067 |
| A\_51\_P210970 | NM\_009522 | Mus musculus wingless-related MMTV integration site 3A (Wnt3a), mRNA | 1.40 | 0,0394 |
| A\_51\_P289279 | NM\_147042 | Mus musculus clone OR55-36 putative olfactory receptor mRNA, partial cds | 1.40 | 0,0076 |
| A\_51\_P212064 | NM\_029638 | Mus musculus, Similar to amiloride binding protein, clone MGC:28601 IMAGE:4216952, mRNA, complete cds | 1.40 | 0,0042 |
| A\_51\_P227232 | NM\_147025 | Mus musculus olfactory receptor MOR135-1 (MOR135-1), mRNA | 1.40 | 0,0006 |
| A\_51\_P151835 | XM\_137316 | unknown EST | 1.40 | 0,0304 |
| A\_51\_P139490 | NM\_146034 | Mus musculus, Similar to meningioma expressed antigen 6 (coiled-coil proline-rich), clone MGC:30562 IMAGE:2647214, mRNA, complete cds | 1.40 | 0,0282 |
| A\_51\_P450373 | AK010113 | hypothetical protein | 1.40 | 0,0077 |
| A\_51\_P435410 | NM\_133355 | Mus musculus glutamate receptor, ionotropic, delta 2 (Grid2) interacting protein 1 (Grid2ip), mRNA | 1.40 | 0,0030 |
| A\_51\_P500996 | XM\_126674 | F-BOX PROTEIN FBL2 homolog [Rattus norvegicus] | 1.40 | 0,0454 |
| A\_51\_P174068 | BC050868 | inferred: Traf2 and NCK interacting kinase, splice variant 8 {Homo sapiens} | 1.40 | 0,0293 |
| A\_51\_P143309 | NM\_146309 | Mus musculus olfactory receptor MOR259-10 (MOR259-10), mRNA | 1.40 | 0,0047 |
| A\_51\_P303675 | NM\_021712 | Mus musculus solute carrier family 18 (vesicular monoamine), member 3 (Slc18a3), mRNA | 1.40 | 0,0237 |
| A\_51\_P215729 | NM\_176924 | hypothetical von Willebrand factor type D domain/EGF-like domain/Trypsin Inhibitor-like cysteine rich domain containing protein | 1.40 | 0,0438 |
| A\_51\_P233101 | NM\_008520 | Mus musculus latent transforming growth factor beta binding protein 3 (Ltbp3), mRNA | 1.40 | 0,0352 |
| A\_51\_P326043 | NM\_172916 | hypothetical protein | 1.39 | 0,0096 |
| A\_51\_P290556 | AK034747 | CDNA FLJ30664 FIS, CLONE FCBBF1000604, WEAKLY SIMILAR TO MYOSIN HEAVY CHAIN MYR 8B homolog [Homo sapiens] | 1.39 | 0,0023 |
| A\_51\_P125679 | AK010725 | hypothetical protein | 1.39 | 0,0070 |
| A\_51\_P470443 | NM\_145522 | SIMILAR TO RAB9 EFFECTOR P40 homolog [Mus musculus] | 1.39 | 0,0310 |
| A\_51\_P158007 | NM\_133797 | Mus musculus expressed sequence C81457 (C81457), mRNA | 1.39 | 0,0239 |
| A\_51\_P461123 | AF186107 | Mus musculus toll-like receptor 5 (Tlr5), mRNA | 1.39 | 0,0198 |
| A\_51\_P400236 | NM\_009536 | Mus musculus tyrosine 3-monooxygenase/tryptophan 5-monooxygenase activation protein, epsilon polypeptide (Ywhae), mRNA | 1.39 | 0,0246 |
| A\_51\_P344376 | NM\_013533 | Mus musculus gene rich cluster, A gene (Grca), mRNA | 1.39 | 0,0275 |
| A\_51\_P370241 | AK015986 | hypothetical PDZ domain (also known as DHR or GLGF) containing protein | 1.39 | 0,0122 |
| A\_51\_P391934 | NM\_029706 | carboxypeptidase B1 (tissue) | 1.39 | 0,0042 |
| A\_51\_P277781 | NM\_013497 | Mus musculus, Similar to cAMresponsive element binding protein 3, clone MGC:6348 IMAGE:3490360, mRNA, complete cds | 1.39 | 0,0099 |
| A\_51\_P132258 | AK032636 | MACROH2A2 | 1.39 | 0,0096 |
| A\_51\_P165027 | AK011208 | hypothetical PHD-finger containing protein | 1.39 | 0,0075 |
| A\_51\_P340807 | AI508699 | Mus musculus cDNA, 3 end | 1.39 | 0,0219 |
| A\_51\_P211165 | AK088130 | Mus musculus, clone IMAGE:3598145, mRNA, partial cds | 1.39 | 0,0014 |
| A\_51\_P206824 | NM\_027126 | hypothetical protein | 1.39 | 0,0033 |
| A\_51\_P434859 | BC026648 | inferred: l-Afadin {Rattus norvegicus} | 1.39 | 0,0398 |
| A\_51\_P203004 | AY046504 | Mus musculus nuclear pore complex-associated intranuclear protein TPR (tpr) mRNA, partial cds, alternatively spliced | 1.39 | 0,0429 |
| A\_51\_P234025 | NM\_148930 | Mus musculus RNA binding motif protein 5 (Rbm5), mRNA | 1.39 | 0,0215 |
| A\_51\_P229875 | AK122457 | Mus musculus expressed sequence AI841796 (AI841796), mRNA | 1.39 | 0,0382 |
| A\_51\_P149997 | NM\_172456 | similar to ENDONUCLEASE G LIKE 1 (EC 3.1.30.-) (ENDO G LIKE) [Homo sapiens] | 1.39 | 0,0026 |
| A\_51\_P499128 | BE368005 | Mus musculus cDNA, 5 end | 1.39 | 0,0363 |
| A\_51\_P282890 | NM\_009003 | Mus musculus RAB4A, member RAS oncogene family (Rab4a), mRNA | 1.39 | 0,0318 |
| A\_51\_P176693 | NM\_146105 | Mus musculus RIKEN cDNA 9630058J23 gene (9630058J23Rik), mRNA | 1.39 | 0,0324 |
| A\_51\_P204772 | AK043188 | unclassifiable | 1.39 | 0,0245 |
| A\_51\_P362812 | NM\_022313 | Mus musculus Era (G-protein)-like 1 (E. coli) (Eral1), mRNA | 1.39 | 0,0122 |
| A\_51\_P238554 | NM\_053181 | Mus musculus expressed sequence AA415817 (AA415817), mRNA | 1.39 | 0,0281 |
| A\_51\_P243604 | NM\_175934 | Mus musculus, Similar to putative protein phosphatase 1 nuclear targeting subunit, clone IMAGE:3157989, mRNA, partial cds | 1.39 | 0,0481 |
| A\_51\_P430314 | NM\_028481 | similar to DJ717I23.1 (NOVEL PROTEIN SIMILAR TO XENOPUS LAEVIS SOJO PROTEIN) (FRAGMENT) [Homo sapiens] | 1.39 | 0,0125 |
| A\_51\_P232771 | NM\_025745 | Mus musculus, Similar to hypothetical protein, clone MGC:27955 IMAGE:3591901, mRNA, complete cds | 1.39 | 0,0057 |
| A\_51\_P482434 | NAP057184-1 | Mus musculus olfactory receptor MOR137-1P (MOR137-1P) pseudogene | 1.39 | 0,0030 |
| A\_51\_P317029 | NM\_019794 | Mus musculus DnaJ (Hsp40) homolog, subfamily A, member 2 (Dnaja2), mRNA | 1.39 | 0,0150 |
| A\_51\_P400585 | NM\_007466 | Mus musculus apoptosis inhibitory protein 5 (Api5), mRNA | 1.39 | 0,0346 |
| A\_51\_P477978 | AK078141 | unclassifiable | 1.39 | 0,0269 |
| A\_51\_P230287 | NM\_030889 | Mus musculus VPS10 domain receptor protein SORCS 2 (Sorcs2-pending), mRNA | 1.39 | 0,0401 |
| A\_51\_P147791 | AK085257 | RU2S (FRAGMENT) homolog [Homo sapiens] | 1.39 | 0,0059 |
| A\_51\_P160514 | NM\_021328 | Mus musculus bridging integrator 3 (Bin3), mRNA | 1.39 | 0,0038 |
| A\_51\_P340947 | NM\_027905 | weakly similar to DJ402G11.5 (NOVEL PROTEIN SIMILAR TO YEAST AND BACTERIAL PREDICTED PROTEINS) [Homo sapiens] | 1.39 | 0,0153 |
| A\_51\_P353125 | NM\_211138 | CHOLINEPHOSPHATE CYTIDYLYLTRANSFERASE B (EC 2.7.7.15) (PHOSPHORYLCHOLINE TRANSFERASE B) (CTP:PHOSPHOCHOLINE CYTIDYLYLTRANSFERASE B) (CT B) (CCT B) (CCT-BETA) homolog [Rattus norvegicus] | 1.39 | 0,0181 |
| A\_51\_P356582 | NM\_147031 | Mus musculus olfactory receptor MOR264-1 (MOR264-1), mRNA | 1.39 | 0,0009 |
| A\_51\_P178894 | NM\_183199 | hypothetical Zn-finger in ubiquitin-hydrolases and other proteins containing protein | 1.39 | 0,0041 |
| A\_51\_P373379 | NM\_009975 | casein kinase II, beta subunit | 1.39 | 0,0168 |
| A\_51\_P424641 | XM\_485674 | SIRTUIN TYPE 4 homolog [Homo sapiens] | 1.39 | 0,0374 |
| A\_51\_P472799 | NM\_027184 | INOSITOL POLYPHOSPHATE MULTIKINASE homolog [Rattus norvegicus] | 1.39 | 0,0421 |
| A\_51\_P233947 | NM\_144522 | weakly similar to EPI64 [Homo sapiens] | 1.39 | 0,0233 |
| A\_51\_P101316 | NM\_133740 | PROTEIN ARGININE N-METHYLTRANSFERASE 3 (EC 2.1.1.-) homolog [Rattus norvegicus] | 1.39 | 0,0093 |
| A\_51\_P362514 | NM\_030244 | Mus musculus RIKEN cDNA 2610524G09 gene (2610524G09Rik), mRNA | 1.39 | 0,0238 |
| A\_51\_P241213 | AK020696 | Mus musculus RIKEN cDNA A030005L19 gene (A030005L19Rik), mRNA | 1.39 | 0,0146 |
| A\_51\_P144068 | NM\_026817 | weakly similar to RAB, MEMBER OF RAS ONCOGENE FAMILY-LIKE 2B [Homo sapiens] | 1.39 | 0,0237 |
| A\_51\_P301891 | NM\_015801 | Mus musculus neuropathy target esterase (Nte-pending), mRNA | 1.39 | 0,0268 |
| A\_51\_P362531 | NM\_144835 | SIMILAR TO HYPOTHETICAL PROTEIN FLJ10359 homolog [Mus musculus] | 1.39 | 0,0033 |
| A\_51\_P457388 | NM\_018774 | Mus musculus early development regulator 2 (homolog of polyhomeotic 2) (Edr2), mRNA | 1.39 | 0,0483 |
| A\_51\_P474034 | XM\_356401 | hypothetical Homeobox domain containing protein | 1.39 | 0,0188 |
| A\_51\_P365482 | NM\_146152 | KIAA0724 PROTEIN (RAN BINDING PROTEIN 13) homolog [Homo sapiens] | 1.39 | 0,0240 |
| A\_51\_P187171 | NM\_030252 | Mus musculus hypothetical protein, MGC:7764 (BC003266), mRNA | 1.39 | 0,0092 |
| A\_51\_P182267 | NM\_026010 | Mus musculus RIKEN cDNA 2610209A20 gene (2610209A20Rik), mRNA | 1.39 | 0,0270 |
| A\_51\_P369690 | NM\_026257 | Mus musculus socius (Soc-pending), mRNA | 1.39 | 0,0041 |
| A\_51\_P457706 | NM\_175667 | ANKYRIN REPEAT DOMAIN PROTEIN 5 homolog [Mus musculus] | 1.39 | 0,0187 |
| A\_51\_P360508 | NM\_144801 | similar to HYPOTHETICAL 51.7 KDA PROTEIN [Homo sapiens] | 1.39 | 0,0325 |
| A\_51\_P268131 | AK029230 | unknown EST | 1.39 | 0,0017 |
| A\_51\_P448555 | NM\_008663 | Mus musculus myosin VIIa (Myo7a), mRNA | 1.39 | 0,0066 |
| A\_51\_P188447 | X99253 | M.musculus mRNA for protein expressed at high levels in testis | 1.39 | 0,0224 |
| A\_51\_P437516 | NAP057202-1 | Mus musculus olfactory receptor MOR171-36P (MOR171-36P) pseudogene | 1.39 | 0,0010 |
| A\_51\_P344758 | AK005698 | hypothetical protein | 1.38 | 0,0029 |
| A\_51\_P247542 | NM\_172799 | hypothetical Tubulin-tyrosine ligase containing protein | 1.38 | 0,0128 |
| A\_51\_P326434 | NM\_025827 | Mus musculus RIKEN cDNA 1300002A08 gene (1300002A08Rik), mRNA | 1.38 | 0,0377 |
| A\_51\_P208534 | AK021185 | hypothetical protein | 1.38 | 0,0037 |
| A\_51\_P159641 | NM\_146486 | Mus musculus olfactory receptor MOR182-5 (MOR182-5), mRNA | 1.38 | 0,0113 |
| A\_51\_P102215 | NM\_001011777 | Mus musculus olfactory receptor MOR185-10 (MOR185-10) pseudogene | 1.38 | 0,0034 |
| A\_51\_P295606 | NM\_025567 | Mus musculus cytochrome c-1 (Cyc1), mRNA | 1.38 | 0,0096 |
| A\_51\_P251402 | NM\_029578 | similar to DTDP-D-GLUCOSE 4,6-DEHYDRATASE (EC 4.2.1.46) [Homo sapiens] | 1.38 | 0,0270 |
| A\_51\_P199778 | AK034273 | kinesin 7 | 1.38 | 0,0178 |
| A\_51\_P260912 | 2810416G20 | unknown EST | 1.38 | 0,0148 |
| A\_51\_P345937 | NM\_134011 | Mus musculus strain BALB/c unknown mRNA | 1.38 | 0,0014 |
| A\_51\_P418540 | NM\_010722 | Mus musculus lamin B2 (Lmnb2), mRNA | 1.38 | 0,0295 |
| A\_51\_P117604 | NM\_025887 | Mus musculus RAB5A, member RAS oncogene family (Rab5a), mRNA | 1.38 | 0,0241 |
| A\_51\_P343474 | NM\_010668 | M.musculus mk2e mRNA | 1.38 | 0,0426 |
| A\_51\_P127176 | NM\_175280 | hypothetical protein | 1.38 | 0,0253 |
| A\_51\_P491240 | AK011092 | weakly similar to RAT HEMOGLOBIN ALPHA CHAIN (FRAGMENT) [Rattus norvegicus] | 1.38 | 0,0116 |
| A\_51\_P129546 | NM\_009219 | Mus musculus somatostatin receptor 4 (Smstr4), mRNA | 1.38 | 0,0072 |
| A\_51\_P159612 | NM\_019487 | Mus musculus heme binding protein 2 (Hebp2), mRNA | 1.38 | 0,0141 |
| A\_51\_P372874 | NM\_011262 | Mus musculus requiem (Req), mRNA | 1.38 | 0,0132 |
| A\_51\_P378298 | NM\_026976 | weakly similar to ANTI-FAS-INDUCED APOPTOSIS (REGULATOR OF FAS-INDUCED APOPTOSIS) [Homo sapiens] | 1.38 | 0,0294 |
| A\_51\_P208472 | NM\_029901 | Mus musculus RIKEN cDNA 9430025F20 gene (9430025F20Rik), mRNA | 1.38 | 0,0365 |
| A\_51\_P314045 | AK220401 | hypothetical Calponin-homology domain, CH-domain structure containing protein | 1.38 | 0,0111 |
| A\_51\_P354419 | NM\_146995 | Mus musculus olfactory receptor MOR182-1 (MOR182-1), mRNA | 1.38 | 0,0078 |
| A\_51\_P155514 | NM\_011164 | Mus musculus prolactin (Prl), mRNA | 1.38 | 0,0078 |
| A\_51\_P480881 | NM\_021513 | Mus musculus hypothetical protein, MNCb-2032 (AB041579), mRNA | 1.38 | 0,0057 |
| A\_51\_P500814 | NM\_010708 | Mus musculus lectin, galactose binding, soluble 9 (Lgals9), mRNA | 1.38 | 0,0296 |
| A\_51\_P208511 | NM\_009924 | Mus musculus cannabinoid receptor 2 (macrophage) (Cnr2), mRNA | 1.38 | 0,0348 |
| A\_51\_P441837 | NM\_026837 | hypothetical protein | 1.38 | 0,0076 |
| A\_51\_P222453 | NM\_025311 | RIKEN cDNA 0610008K04 gene | 1.38 | 0,0474 |
| A\_51\_P371255 | NM\_016924 | ORF5 PROTEIN homolog [Mus musculus] | 1.38 | 0,0033 |
| A\_51\_P252819 | NM\_001011869 | Mus musculus olfactory receptor MOR257-5P (MOR257-5P) pseudogene | 1.38 | 0,0059 |
| A\_51\_P270213 | NM\_023289 | Mus musculus CEA-related cell adhesion molecule 11 (Ceacam11), mRNA | 1.38 | 0,0207 |
| A\_51\_P415247 | AK015886 | hypothetical protein | 1.38 | 0,0260 |
| A\_51\_P473288 | AK076599 | unknown EST | 1.38 | 0,0030 |
| A\_51\_P381988 | NM\_010445 | Mus musculus H6 homeo box 1 (Hmx1), mRNA | 1.38 | 0,0333 |
| A\_51\_P272414 | NM\_146762 | Mus musculus olfactory receptor MOR213-2 (MOR213-2), mRNA | 1.38 | 0,0091 |
| A\_51\_P248857 | NM\_026597 | hypothetical protein | 1.38 | 0,0270 |
| A\_51\_P375267 | NM\_013810 | Mus musculus drebrin-like (Dbnl), mRNA | 1.38 | 0,0453 |
| A\_51\_P305708 | NM\_018830 | Mus musculus N-acylsphingosine amidohydrolase 2 (Asah2), mRNA | 1.38 | 0,0223 |
| A\_51\_P210433 | NM\_146486 | Mus musculus olfactory receptor MOR182-5 (MOR182-5), mRNA | 1.37 | 0,0057 |
| A\_51\_P247211 | NM\_153776 | unknown EST | 1.37 | 0,0221 |
| A\_51\_P401337 | AK017242 | hypothetical protein | 1.37 | 0,0035 |
| A\_51\_P359454 | NM\_030561 | Mus musculus hypothetical protein, MGC:7550 (BC004004), mRNA | 1.37 | 0,0331 |
| A\_51\_P238009 | AK030047 | HYPOTHETICAL 76.6 KDA PROTEIN homolog [Macaca fascicularis] | 1.37 | 0,0426 |
| A\_51\_P382551 | NM\_019496 | Mus musculus Alport syndrome, mental retardation, midface hypoplasia and elliptocytosis chromosomal region gene 1 homolog (human) (Ammecr1), mRNA | 1.37 | 0,0185 |
| A\_51\_P427151 | NM\_138671 | Mus musculus putative inorganic polyphosphate/ATP-NAD kinase (MGC7589), mRNA | 1.37 | 0,0278 |
| A\_51\_P115738 | NM\_053248 | Mus musculus solute carrier family 5 (sodium iodide symporter), member 5 (Slc5a5), mRNA | 1.37 | 0,0070 |
| A\_51\_P153581 | AK016891 | hypothetical protein | 1.37 | 0,0070 |
| A\_51\_P289506 | AK032451 | SIMILAR TO MITOTIC CONTROL PROTEIN DIS3 HOMOLOG homolog [Homo sapiens] | 1.37 | 0,0282 |
| A\_51\_P413916 | XM\_130859 | MEMBRANE ASSOCIATED PROGESTERONE RECEPTOR COMPONENT 2 (PROGESTERONE MEMBRANE BINDING PROTEIN) (STEROID RECEPTOR PROTEIN DG6) homolog [Homo sapiens] | 1.37 | 0,0268 |
| A\_51\_P292230 | AK047481 | hypothetical Arginine-rich region containing protein | 1.37 | 0,0037 |
| A\_51\_P374869 | NM\_013850 | Mus musculus ATP-binding cassette, sub-family A (ABC1), member 7 (Abca7), mRNA | 1.37 | 0,0106 |
| A\_51\_P250848 | NM\_030264 | Mus musculus hypothetical protein, MGC:6623 (BC002118), mRNA | 1.37 | 0,0232 |
| A\_51\_P160272 | AK032445 | unclassifiable | 1.37 | 0,0072 |
| A\_51\_P167284 | NM\_025984 | Mus musculus small proline rich-like 3 (Sprrl3), mRNA | 1.37 | 0,0136 |
| A\_51\_P403578 | AK007907 | hypothetical protein | 1.37 | 0,0396 |
| A\_51\_P222973 | NM\_138949 | Mus musculus, similar to zinc finger protein 135 (clone pHZ-17), clone MGC:39058 IMAGE:5365187, mRNA, complete cds | 1.37 | 0,0042 |
| A\_51\_P382859 | AK049769 | hypothetical Phenylalanine-rich region containing protein | 1.37 | 0,0309 |
| A\_51\_P450411 | BC058768 | Mus musculus mucin glycoprotein MUC3 mRNA, partial cds | 1.37 | 0,0114 |
| A\_51\_P400490 | AK041423 | hypothetical Dbl domain (dbl/cdc24 rhoGEF family) containing protein | 1.37 | 0,0080 |
| A\_51\_P229816 | NM\_145445 | Mus musculus, Similar to eukaryotic translation initiation factor 2B, subunit 2 (beta, 39kD), clone MGC:7057 IMAGE:3156632, mRNA, complete cds | 1.37 | 0,0494 |
| A\_51\_P124499 | XM\_354566 | Mus musculus integral inner nuclear membrane protein MAN1 mRNA, partial cds | 1.37 | 0,0254 |
| A\_51\_P458748 | NM\_013552 | Mus musculus hyaluronan mediated motility receptor (RHAMM) (Hmmr), mRNA | 1.37 | 0,0023 |
| A\_51\_P271336 | NM\_197940 | unknown EST | 1.37 | 0,0228 |
| A\_51\_P276452 | NM\_178646 | hypothetical CENP-B protein containing protein | 1.37 | 0,0275 |
| A\_51\_P180862 | NM\_144892 | Mus musculus, Similar to coactivator independent of AF-2, clone MGC:28864 IMAGE:4511696, mRNA, complete cds | 1.37 | 0,0438 |
| A\_51\_P168092 | NM\_024205 | SIMILAR TO HYPOTHETICAL PROTEIN homolog [Mus musculus] | 1.37 | 0,0152 |
| A\_51\_P342535 | XM\_134599 | ZINC FINGER PROTEIN | 1.36 | 0,0371 |
| A\_51\_P387388 | NM\_146279 | Mus musculus olfactory receptor MOR224-12 (MOR224-12), mRNA | 1.36 | 0,0061 |
| A\_51\_P345643 | NM\_009336 | Mus musculus transcription factor-like 1 (Tcfl1), mRNA | 1.36 | 0,0090 |
| A\_51\_P380772 | NM\_178224 | Mus musculus cystathionine beta-synthase mRNA, 5 untranslated region | 1.36 | 0,0484 |
| A\_51\_P455861 | NM\_027105 | hypothetical protein | 1.36 | 0,0051 |
| A\_51\_P459091 | NM\_172550 | hypothetical Uncharacterized protein family UPF0054 containing protein | 1.36 | 0,0039 |
| A\_51\_P405304 | NM\_053172 | Mus musculus matrix extracellular phosphoglycoprotein with ASARM motif (bone) (Mepe), mRNA | 1.36 | 0,0293 |
| A\_51\_P206563 | AK008751 | CDNA FLJ13589 FIS, CLONE PLACE1009308, WEAKLY SIMILAR TO GLUCOSE REPRESSION MEDIATOR PROTEIN homolog [Homo sapiens] | 1.36 | 0,0109 |
| A\_51\_P183300 | NM\_007506 | Mus musculus ATP synthase, H+ transporting, mitochondrial F0 complex, subunit c (subunit 9), isoform 1 (Atp5g1), mRNA | 1.36 | 0,0243 |
| A\_51\_P289872 | NM\_172585 | weakly similar to C-MPL BINDING PROTEIN [Homo sapiens] | 1.36 | 0,0248 |
| A\_51\_P493264 | AK043682 | similar to PEROXISOME ASSEMBLY PROTEIN 10 (PEROXIN-10) [Homo sapiens] | 1.36 | 0,0048 |
| A\_51\_P501429 | AK081839 | CDNA FLJ13096 FIS, CLONE NT2RP3002166 homolog [Homo sapiens] | 1.36 | 0,0080 |
| A\_51\_P509881 | NM\_153798 | Mus musculus, polymerase (RNA) II (DNA directed) polypeptide B (140kD), clone IMAGE:3158185, mRNA | 1.36 | 0,0285 |
| A\_51\_P466448 | NM\_153407 | TGF-BETA INDUCED APOTOSIS PROTEIN 12 homolog [Homo sapiens] | 1.36 | 0,0346 |
| A\_51\_P342810 | NM\_027883 | KIAA0134 RNA HELICASE (HRH1) (FRAGMENT) homolog [Homo sapiens] | 1.36 | 0,0066 |
| A\_51\_P242918 | NM\_198675 | hypothetical Ferredoxin-fold anticodon binding domain containing protein | 1.36 | 0,0254 |
| A\_51\_P346614 | BC024687 | "Mus musculus, similar to loop tail associated protein� vang (van gogh)-like 2 (Drosophila)� loop tail, clone IMAGE:3669388, mRNA, partial cds" | 1.36 | 0,0186 |
| A\_51\_P471921 | A\_51\_P471921 | Mus musculus cDNA, 5 end | 1.36 | 0,0042 |
| A\_51\_P441822 | NM\_144878 | Mus musculus flavin-containing monooxygenase 4 mRNA, complete cds | 1.36 | 0,0075 |
| A\_51\_P476900 | AK053449 | Mus musculus PR domain containing 15 (Prdm15), mRNA | 1.36 | 0,0282 |
| A\_51\_P161902 | XM\_355476 | hypothetical protein | 1.36 | 0,0044 |
| A\_51\_P519791 | NM\_198605 | hypothetical protein | 1.36 | 0,0102 |
| A\_51\_P157223 | AK019057 | epiregulin | 1.36 | 0,0240 |
| A\_51\_P263983 | NM\_146908 | Mus musculus olfactory receptor MOR248-1 (MOR248-1), mRNA | 1.36 | 0,0132 |
| A\_51\_P417077 | NM\_011700 | Mus musculus partial mRNA for villin-like protein (Vill gene) | 1.36 | 0,0031 |
| A\_51\_P174505 | NM\_146336 | Mus musculus olfactory receptor MOR170-11 (MOR170-11), mRNA | 1.36 | 0,0014 |
| A\_51\_P322990 | NM\_175001 | CDNA FLJ20594 FIS, CLONE KAT08731 (SIMILAR TO MITOCHONDRIAL RIBOSOMAL PROTEIN L22) homolog [Homo sapiens] | 1.36 | 0,0291 |
| A\_51\_P489043 | NM\_011308 | Mus musculus nuclear receptor co-repressor 1 (Ncor1), mRNA | 1.36 | 0,0099 |
| A\_51\_P446098 | NM\_016799 | Mus musculus serine/arginine repetitive matrix 1 (Srrm1), mRNA | 1.36 | 0,0047 |
| A\_51\_P184969 | NM\_021430 | Mus musculus RIKEN cDNA 2900002H16 gene (2900002H16Rik), mRNA | 1.36 | 0,0057 |
| A\_51\_P311038 | NM\_001024139 | Mus musculus, Similar to a disintegrin and metalloproteinase with thrombospondin motifs 1 (ADAMTS-1), clone IMAGE:3491991, mRNA, partial cds | 1.36 | 0,0308 |
| A\_51\_P408100 | NM\_008011 | Mus musculus fibroblast growth factor receptor 4 (Fgfr4), mRNA | 1.36 | 0,0241 |
| A\_51\_P168392 | NM\_030188 | SIMILAR TO RIKEN CDNA 2510042P03 GENE homolog [Mus musculus] | 1.36 | 0,0056 |
| A\_51\_P124638 | NAP108490-1 | Mus musculus olfactory receptor GA\_x5J8B7W5KGR-202501-203873 (GA\_x5J8B7W5KGR-202501-203873) pseudogene | 1.36 | 0,0109 |
| A\_51\_P262410 | AK014745 | hypothetical protein | 1.35 | 0,0121 |
| A\_51\_P383063 | NM\_147060 | Mus musculus olfactory receptor MOR34-2 (MOR34-2), mRNA | 1.35 | 0,0419 |
| A\_51\_P472219 | NM\_199301 | Mus musculus, Similar to hypothetical protein BC004409, clone MGC:28365 IMAGE:4019717, mRNA, complete cds | 1.35 | 0,0423 |
| A\_51\_P482670 | AK079839 | unknown EST | 1.35 | 0,0341 |
| A\_51\_P287797 | AK016041 | hypothetical protein | 1.35 | 0,0155 |
| A\_51\_P315993 | AK080082 | hypothetical protein | 1.35 | 0,0028 |
| A\_51\_P465386 | AK008974 | hypothetical alpha/beta-Hydrolases structure containing protein | 1.35 | 0,0460 |
| A\_51\_P281079 | A\_51\_P281079 | Mus musculus cDNA, 3 end | 1.35 | 0,0109 |
| A\_51\_P453826 | NM\_146967 | Mus musculus olfactory receptor MOR233-2 (MOR233-2), mRNA | 1.35 | 0,0193 |
| A\_51\_P395694 | NM\_019481 | Mus musculus solute carrier family 13 (sodium/sulphate symporters), member 1 (Slc13a1), mRNA | 1.35 | 0,0261 |
| A\_51\_P269020 | AK006578 | hypothetical protein | 1.35 | 0,0054 |
| A\_51\_P465128 | NM\_029935 | B CELL RAG-ASSOCIATED PROTEIN | 1.35 | 0,0346 |
| A\_51\_P381239 | NM\_153563 | hypothetical Proline-rich region containing protein | 1.35 | 0,0428 |
| A\_51\_P460929 | NM\_153176 | Mus musculus paraplegin (Spg7), mRNA | 1.35 | 0,0179 |
| A\_51\_P508394 | NM\_146579 | Mus musculus olfactory receptor MOR199-1 (MOR199-1), mRNA | 1.35 | 0,0246 |
| A\_51\_P353772 | NM\_008126 | Mus musculus gap junction membrane channel protein beta 3 (Gjb3), mRNA | 1.35 | 0,0083 |
| A\_51\_P214755 | NM\_175201 | HYPOTHETICAL 43.9 KDA PROTEIN homolog [Homo sapiens] | 1.35 | 0,0420 |
| A\_51\_P485483 | NM\_020027 | Mus musculus HLA-B associated transcript 2 (Bat2), mRNA | 1.35 | 0,0088 |
| A\_51\_P120912 | AK048960 | ZETA-SARCOGLYCAN homolog [Homo sapiens] | 1.35 | 0,0071 |
| A\_51\_P143031 | NM\_133867 | Mus musculus expressed sequence AI504489 (AI504489), mRNA | 1.35 | 0,0090 |
| A\_51\_P248644 | BC065162 | TYROSYL-DNA PHOSPHODIESTERASE PROTEIN (FRAGMENT) homolog [Homo sapiens] | 1.35 | 0,0249 |
| A\_51\_P419599 | AK016764 | unclassifiable | 1.35 | 0,0462 |
| A\_51\_P458194 | NM\_009528 | Mus musculus wingless-related MMTV integration site 7B (Wnt7b), mRNA | 1.35 | 0,0214 |
| A\_51\_P245468 | AI838397 | Mus musculus cDNA, 3 end | 1.35 | 0,0101 |
| A\_51\_P107782 | BC023820 | FOLLICULAR VARIANT TRANSLOCATION PROTEIN 1 PRECURSOR (FVT-1) homolog [Homo sapiens] | 1.35 | 0,0199 |
| A\_51\_P458584 | NM\_008040 | Mus musculus formyl peptide receptor, related sequence 3 (Fpr-rs3), mRNA | 1.35 | 0,0063 |
| A\_51\_P383228 | NM\_009385 | Mus musculus thyroid transcription factor 1 (Titf1), mRNA | 1.35 | 0,0165 |
| A\_51\_P119659 | NM\_008059 | Mus musculus G0/G1 switch gene 2 (G0s2), mRNA | 1.35 | 0,0224 |
| A\_51\_P226134 | NM\_172422 | hypothetical protein | 1.35 | 0,0397 |
| A\_51\_P216605 | NM\_177993 | HMG-BOX CONTAINING PROTEIN 1 homolog [Rattus norvegicus] | 1.35 | 0,0069 |
| A\_51\_P199927 | NM\_133878 | Mus musculus RIKEN cDNA 4931417M11 gene (4931417M11Rik), mRNA | 1.35 | 0,0457 |
| A\_51\_P333594 | A\_51\_P333594 | Mus musculus cDNA, 3 end | 1.35 | 0,0136 |
| A\_51\_P488308 | NM\_139061 | Mus musculus VPS54-like (yeast) (Vps54l-pending), mRNA | 1.34 | 0,0169 |
| A\_51\_P211064 | NM\_020290 | Mus musculus odorant receptor S18 gene (Ors18), mRNA | 1.34 | 0,0019 |
| A\_51\_P119525 | NM\_054069 | Mus musculus prostatic steroid binding protein C1 (Psbpc1-pending), mRNA | 1.34 | 0,0020 |
| A\_51\_P124934 | AK031219 | U4/U6 SNRNP 60 KDA PROTEIN homolog [Homo sapiens] | 1.34 | 0,0099 |
| A\_51\_P358012 | NM\_009253 | Mus musculus serine protease inhibitor-2 related sequence 1 (Spi2-rs1), mRNA | 1.34 | 0,0226 |
| A\_51\_P482410 | AK049385 | hypothetical Immunoglobulin C-2 type/Immunoglobulin subtype containing protein | 1.34 | 0,0132 |
| A\_51\_P451696 | ENSMUST00000072368 | B4 OLFACTORY RECEPTOR | 1.34 | 0,0057 |
| A\_51\_P145010 | AK010095 | weakly similar to COLLAGEN-LIKE PROTEIN [Herpesvirus saimiri] | 1.34 | 0,0092 |
| A\_51\_P452153 | NM\_027222 | weakly similar to PROAPOPTOTIC CASPASE ADAPTOR PROTEIN [Homo sapiens] | 1.34 | 0,0190 |
| A\_51\_P153289 | NM\_026522 | Mus musculus RIKEN cDNA 3110023E09 gene (3110023E09Rik), mRNA | 1.34 | 0,0475 |
| A\_51\_P401001 | A\_51\_P401001 | Mus musculus cDNA, 3 end | 1.34 | 0,0325 |
| A\_51\_P188462 | NAP057293-1 | Mus musculus olfactory receptor MOR135-16P (MOR135-16P) pseudogene | 1.34 | 0,0178 |
| A\_51\_P321698 | NM\_021534 | Mus musculus peroxisomal membrane protein 4 (Pxmp4), mRNA | 1.34 | 0,0268 |
| A\_51\_P316446 | NM\_027588 | Mus musculus 5-nucleotidase, cytosolic IB (Nt5c1b), mRNA | 1.34 | 0,0447 |
| A\_51\_P208845 | NM\_178748 | hypothetical EGF-like domain, subtype 2/Laminin-G domain/Fibronectin type III domain/EGF-like domain/Calcium-binding EGF-like domain/Fibronectin type III repeat/Type II EGF-like signature containing protein | 1.34 | 0,0135 |
| A\_51\_P490296 | NM\_177472 | hypothetical protein | 1.34 | 0,0305 |
| A\_51\_P497882 | NM\_030080 | Mus musculus Attaches to Cre (Atce1) | 1.34 | 0,0144 |
| A\_51\_P237106 | NM\_001011804 | Mus musculus olfactory receptor MOR233-16P (MOR233-16P) pseudogene | 1.34 | 0,0051 |
| A\_51\_P499997 | BF583970 | BROMODOMAIN AND PHD FINGER CONTAINING PROTEIN | 1.34 | 0,0289 |
| A\_51\_P503719 | NM\_013554 | Mus musculus homeo box D10 (Hoxd10), mRNA | 1.34 | 0,0018 |
| A\_51\_P146837 | AK044171 | similar to IONOTROPIC GLUTAMATE RECEPTOR NMDA subunit NR3A[Rattus norvegicus] | 1.34 | 0,0040 |
| A\_51\_P484842 | NM\_007493 | Mus musculus asialoglycoprotein receptor 2 (Asgr2), mRNA | 1.34 | 0,0074 |
| A\_51\_P171602 | AF011416 | Mus musculus putative pheromone receptor (VR6) mRNA, partial cds | 1.34 | 0,0026 |
| A\_51\_P332081 | NM\_007619 | Mus musculus Casitas B-lineage lymphoma (Cbl), mRNA | 1.34 | 0,0315 |
| A\_51\_P308139 | AK031289 | PRO NEUREGULIN PRECURSOR PRO [CONTAINS: NEUREGULIN | 1.34 | 0,0321 |
| A\_51\_P413445 | AK016098 | unclassifiable | 1.34 | 0,0227 |
| A\_51\_P116687 | NM\_025851 | Mus musculus RIKEN cDNA 1700010I14 gene (1700010I14Rik), mRNA | 1.34 | 0,0098 |
| A\_51\_P121955 | AK051766 | CALCIUM-INDEPENDENT ALPHA-LATROTOXIN RECEPTOR 3 PRECURSOR (LATROPHILIN 3) (LRP3) (CIRL) (CL3) (LPH3) homolog [Rattus norvegicus] | 1.34 | 0,0099 |
| A\_51\_P109439 | NM\_146325 | Mus musculus olfactory receptor MOR25-1 (MOR25-1), mRNA | 1.34 | 0,0047 |
| A\_51\_P352076 | NM\_008399 | Mus musculus integrin, alpha E, epithelial-associated (Itgae), mRNA | 1.34 | 0,0419 |
| A\_51\_P412895 | NM\_146591 | Mus musculus olfactory receptor MOR179-3 (MOR179-3), mRNA | 1.34 | 0,0028 |
| A\_51\_P271644 | NM\_011246 | Mus musculus RAS guanyl releasing protein 1 (Rasgrp1), mRNA | 1.33 | 0,0228 |
| A\_51\_P483839 | NM\_016706 | Mus musculus coilin (Coil), mRNA | 1.33 | 0,0480 |
| A\_51\_P249414 | NM\_022408 | Mus musculus expressed sequence 2 embryonic lethal (Es2el), mRNA | 1.33 | 0,0331 |
| A\_51\_P515349 | AK049359 | PHOSPHATIDYLGLYCEROPHOSPHATE SYNTHASE homolog [Cricetulus griseus] | 1.33 | 0,0215 |
| A\_51\_P342418 | NM\_177661 | ARYLACETAMIDE DEACETYLASE EC 3.1.1.- | 1.33 | 0,0091 |
| A\_51\_P204492 | BC088739 | hypothetical Immunoglobulin and major histocompatibility complex domain/Cytochrome c family heme-binding site/Zinc finger, C2H2 type containing protein | 1.33 | 0,0405 |
| A\_51\_P398596 | NM\_144812 | Mus musculus, clone MGC:28739 IMAGE:4481333, mRNA, complete cds | 1.33 | 0,0123 |
| A\_51\_P179251 | NM\_177757 | KINESIN SUPERFAMILY PROTEIN 26B (FRAGMENT) | 1.33 | 0,0341 |
| A\_51\_P429046 | NM\_152810 | cell division cycle 5-like (S. pombe) | 1.33 | 0,0113 |
| A\_51\_P430552 | NM\_175935 | hypothetical PA-phosphatase related phosphoesterase protein | 1.33 | 0,0224 |
| A\_51\_P293048 | NM\_010759 | Mus musculus melanoma antigen, family B, 1 (Mageb1), mRNA | 1.33 | 0,0473 |
| A\_51\_P323579 | NM\_023794 | Mus musculus RIKEN cDNA 8430401F14 gene (8430401F14Rik), mRNA | 1.33 | 0,0153 |
| A\_51\_P349023 | NM\_026309 | Mus musculus RIKEN cDNA 1010001J12 gene (1010001J12Rik), mRNA | 1.33 | 0,0457 |
| A\_51\_P210021 | NM\_028735 | hypothetical Tetratricopeptide repeat (TPR) structure containing protein | 1.33 | 0,0075 |
| A\_51\_P370315 | NM\_011937 | Mus musculus glucosamine-6-phosphate deaminase (Gnpi), mRNA | 1.33 | 0,0024 |
| A\_51\_P488230 | AK036733 | unknown EST | 1.33 | 0,0047 |
| A\_51\_P425674 | AK005024 | isovaleryl coenzyme A dehydrogenase | 1.33 | 0,0109 |
| A\_51\_P486174 | NM\_009299 | Mus musculus seminal vesicle antigen (Sva), mRNA | 1.33 | 0,0416 |
| A\_51\_P371876 | NM\_027230 | similar to CTCL TUMOR ANTIGEN SE14-3 [Homo sapiens] | 1.33 | 0,0189 |
| A\_51\_P320606 | NM\_023746 | Mus musculus RIKEN cDNA 1600013P04 gene (1600013P04Rik), mRNA | 1.33 | 0,0050 |
| A\_51\_P133295 | NM\_028737 | hypothetical Immunoglobulin structure containing protein | 1.33 | 0,0056 |
| A\_51\_P306160 | AK051378 | similar to LEUCINE ZIPPER BEARING KINASE [Homo sapiens] | 1.33 | 0,0101 |
| A\_51\_P133215 | CB227689 | Mus musculus cDNA, 5 end | 1.33 | 0,0477 |
| A\_51\_P150802 | NM\_008622 | Mus musculus Mpv17 transgene, kidney disease mutant (Mpv17), mRNA | 1.33 | 0,0415 |
| A\_51\_P145020 | NM\_146245 | RETINA SPECIFIC PROTEIN PAL homolog [Rattus norvegicus] | 1.33 | 0,0195 |
| A\_51\_P256566 | NM\_175164 | Mus musculus cDNA, 5 end | 1.33 | 0,0078 |
| A\_51\_P418704 | NM\_134168 | Mus musculus vomeronasal 1 receptor, C13 (V1rc13), mRNA | 1.33 | 0,0270 |
| A\_51\_P435035 | NM\_025708 | Mus musculus RIKEN cDNA 4432406C05 gene (4432406C05Rik), mRNA | 1.33 | 0,0317 |
| A\_51\_P492346 | NM\_026685 | hypothetical protein | 1.33 | 0,0189 |
| A\_51\_P133247 | AK076014 | Mus musculus fatty acid transport protein 3 mRNA, partial cds | 1.33 | 0,0106 |
| A\_51\_P455528 | NM\_175644 | hypothetical Cystine-knot domain containing protein | 1.33 | 0,0030 |
| A\_51\_P122507 | NM\_146032 | SIGNAL RECOGNITION PARTICLE 68 KDA PROTEIN (SRP68) homolog [Canis familiaris] | 1.33 | 0,0306 |
| A\_51\_P307944 | NM\_010102 | Mus musculus endothelial differentiation, G-protein-coupled receptor 6 (Edg6), mRNA | 1.33 | 0,0045 |
| A\_51\_P468544 | NM\_009237 | Mus musculus SRY-box containing gene 3 (Sox3), mRNA | 1.33 | 0,0136 |
| A\_51\_P363258 | NM\_133731 | Mus musculus RIKEN cDNA 4733401N09 gene (4733401N09Rik), mRNA | 1.33 | 0,0193 |
| A\_51\_P381086 | NM\_178699 | BRAIN CDNA, CLONE MNCB-3966 (FRAGMENT) | 1.33 | 0,0066 |
| A\_51\_P517753 | NM\_013591 | Mus musculus mucosal vascular addressin cell adhesion molecule 1 (Madcam1), mRNA | 1.33 | 0,0319 |
| A\_51\_P244386 | AK086107 | MYOFERLIN (FER-1 LIKE PROTEIN 3) homolog [Homo sapiens] | 1.32 | 0,0051 |
| A\_51\_P301696 | A\_51\_P301696 | Mus musculus cDNA, 3 end | 1.32 | 0,0070 |
| A\_51\_P392604 | NM\_001011864 | Mus musculus olfactory receptor MOR168-2P (MOR168-2P) pseudogene | 1.32 | 0,0033 |
| A\_51\_P469476 | AK036529 | hypothetical protein | 1.32 | 0,0078 |
| A\_51\_P381157 | NM\_027420 | hypothetical protein | 1.32 | 0,0377 |
| A\_51\_P145171 | NM\_133729 | Mus musculus RIKEN cDNA 2610018G03 gene (2610018G03Rik), mRNA | 1.32 | 0,0028 |
| A\_51\_P262131 | NM\_146698 | Mus musculus olfactory receptor MOR202-8 (MOR202-8), mRNA | 1.32 | 0,0066 |
| A\_51\_P130459 | NM\_011694 | Mus musculus voltage-dependent anion channel 1 (Vdac1), mRNA | 1.32 | 0,0248 |
| A\_51\_P497560 | NM\_172269 | VACUOLAR PROTEIN SORTING PROTEIN 18 homolog [Homo sapiens] | 1.32 | 0,0309 |
| A\_51\_P246677 | NM\_020002 | Mus musculus meiotic cohesion Rec8 (Rec8-pending), mRNA | 1.32 | 0,0256 |
| A\_51\_P339154 | NM\_146058 | Mus musculus, Similar to lymphocyte antigen 6 complex, locus F, clone MGC:29251 IMAGE:5053382, mRNA, complete cds | 1.32 | 0,0052 |
| A\_51\_P234462 | NM\_133756 | Mus musculus RIKEN cDNA 2410004J02 gene (2410004J02Rik), mRNA | 1.32 | 0,0473 |
| A\_51\_P311105 | NM\_009134 | Mus musculus sodium channel, voltage-gated, type X, alpha polypeptide (Scn10a), mRNA | 1.32 | 0,0080 |
| A\_51\_P383194 | NM\_008804 | Mus musculus phosphodiesterase 9A (Pde9a), mRNA | 1.32 | 0,0472 |
| A\_51\_P206053 | NM\_010577 | Mus musculus integrin alpha 5 (fibronectin receptor alpha) (Itga5), mRNA | 1.32 | 0,0423 |
| A\_51\_P273326 | XM\_132230 | Mus musculus, Similar to RIKEN cDNA 1810024J13 gene, clone IMAGE:3594108, mRNA | 1.32 | 0,0336 |
| A\_51\_P182201 | NM\_028981 | Mouse neuroendocrine L-type calcium channel (CaCh3b) mRNA, partial cds | 1.32 | 0,0385 |
| A\_51\_P398943 | NM\_027614 | DMR PROTEIN homolog [Homo sapiens] | 1.32 | 0,0239 |
| A\_51\_P308625 | NM\_146759 | Mus musculus olfactory receptor MOR119-1 (MOR119-1), mRNA | 1.32 | 0,0102 |
| A\_51\_P116896 | NM\_146768 | Mus musculus olfactory receptor MOR206-3 (MOR206-3), mRNA | 1.32 | 0,0074 |
| A\_51\_P277139 | AK017078 | Mus musculus RIKEN cDNA 4933436E20 gene (4933436E20Rik), mRNA | 1.32 | 0,0175 |
| A\_51\_P402477 | AK173166 | hypothetical Esterase/lipase/thioesterase family active site containing protein | 1.32 | 0,0117 |
| A\_51\_P150845 | NM\_183289 | hypothetical WW/rsp5/WWP domain profile/WW / rsp5 / WWP domain containing protein | 1.32 | 0,0260 |
| A\_51\_P153734 | AK013939 | Mus musculus kinesin-like 5 (mitotic kinesin-like protein 1) (Knsl5), mRNA | 1.32 | 0,0290 |
| A\_51\_P380587 | NM\_028959 | hypothetical Outer arm dynein light chain 1 structure containing protein | 1.32 | 0,0151 |
| A\_51\_P111304 | NM\_173734 | weakly similar to CG17660 PROTEIN [Drosophila melanogaster] | 1.32 | 0,0225 |
| A\_51\_P364649 | NM\_021470 | Mus musculus ring finger protein 32 (Rnf32), mRNA | 1.32 | 0,0155 |
| A\_51\_P422388 | NM\_148935 | Mus musculus forkhead box containing protein N4 (Foxn4) mRNA, complete cds | 1.32 | 0,0207 |
| A\_51\_P115863 | AK019925 | RIKEN cDNA 2310066I18 gene | 1.32 | 0,0159 |
| A\_51\_P516268 | AK083404 | unknown EST | 1.32 | 0,0210 |
| A\_51\_P393086 | NM\_145502 | Mus musculus, similar to Caenorhabditis elegans protein C42C1.9, clone MGC:19110 IMAGE:4208238, mRNA, complete cds | 1.32 | 0,0125 |
| A\_51\_P139607 | NM\_146742 | Mus musculus olfactory receptor MOR245-1 (MOR245-1), mRNA | 1.32 | 0,0400 |
| A\_51\_P102333 | NM\_023478 | Mus musculus uroplakin 3 (Upk3), mRNA | 1.32 | 0,0079 |
| A\_51\_P506054 | AK038997 | weakly similar to BCL2/ADENOVIRUS E1B 19-KDA PROTEIN-INTERACTING PROTEIN 2 [Homo sapiens] | 1.32 | 0,0306 |
| A\_51\_P283818 | NM\_029614 | SERINE PROTEASE (HYPOTHETICAL 43.0 KDA PROTEIN) (PROTEASE, SERINE, 23) homolog [Homo sapiens] | 1.32 | 0,0494 |
| A\_51\_P441153 | NM\_144955 | Mus musculus homeodomain transcription factor (Nkx6-1) mRNA, complete cds | 1.32 | 0,0248 |
| A\_51\_P115234 | NM\_134210 | Mus musculus vomeronasal 1 receptor, G9 (V1rg9), mRNA | 1.32 | 0,0438 |
| A\_51\_P395373 | NM\_021464 | Mus musculus protein tyrosine phosphatase, receptor type, T (Ptprt), mRNA | 1.32 | 0,0382 |
| A\_51\_P310699 | NM\_178707 | HYPOTHETICAL ZINC FINGER PROTEIN KIAA0211 homolog [Homo sapiens] | 1.32 | 0,0337 |
| A\_51\_P424211 | NM\_008557 | Mus musculus FXYD domain-containing ion transport regulator 3 (Fxyd3), mRNA | 1.31 | 0,0346 |
| A\_51\_P307325 | NM\_009030 | Mus musculus retinoblastoma binding protein 4 (Rbbp4), mRNA | 1.31 | 0,0283 |
| A\_51\_P265173 | NM\_207238 | F BOX ONLY PROTEIN | 1.31 | 0,0481 |
| A\_51\_P338655 | AF135494 | DELTA NAIP PROTEIN (FRAGMENT) | 1.31 | 0,0156 |
| A\_51\_P329508 | NM\_001013392 | Mus musculus, Similar to ras responsive element binding protein 1, clone IMAGE:5321450, mRNA | 1.31 | 0,0181 |
| A\_51\_P336443 | AK036326 | weakly similar to reverse transcriptase-like protein (fragment) [Rattus norvegicus] | 1.31 | 0,0333 |
| A\_51\_P273657 | XM\_355958 | Mus musculus, clone IMAGE:5036118, mRNA | 1.31 | 0,0150 |
| A\_51\_P435487 | AK005717 | similar to DJ63M2.2 (SIMILAR TO ACTIN) (FRAGMENT) [Homo sapiens] | 1.31 | 0,0081 |
| A\_51\_P488028 | NAP057094-1 | Mus musculus olfactory receptor GA\_x5J8B7W62NC-668773-668438 (GA\_x5J8B7W62NC-668773-668438) pseudogene | 1.31 | 0,0028 |
| A\_51\_P358507 | NM\_147092 | Mus musculus olfactory receptor MOR14-2 (MOR14-2), mRNA | 1.31 | 0,0239 |
| A\_51\_P174244 | AI462521 | Mus musculus cDNA, 3 end | 1.31 | 0,0103 |
| A\_51\_P350073 | NM\_023668 | Mus musculus nuclear distribution gene E-like (Nudel-pending), mRNA | 1.31 | 0,0303 |
| A\_51\_P274506 | NM\_011573 | testis expressed gene 264 | 1.31 | 0,0089 |
| A\_51\_P334174 | NM\_145383 | Mus musculus, Similar to rhodopsin (opsin 2, rod pigment) (retinitis pigmentosa 4, autosomal dominant), clone MGC:21585 IMAGE:4500760, mRNA, complete cds | 1.31 | 0,0197 |
| A\_51\_P391727 | NM\_009205 | Mus musculus solute carrier family 3, member 1 (Slc3a1), mRNA | 1.31 | 0,0398 |
| A\_51\_P373770 | NAP057106-1 | Mus musculus olfactory receptor GA\_x5J8B7W5Q32-585040-584392 (GA\_x5J8B7W5Q32-585040-584392) pseudogene | 1.31 | 0,0122 |
| A\_51\_P475545 | NM\_001003685 | Mus musculus (clone pCM7) growth hormone-releasing factor receptor mRNA sequence | 1.31 | 0,0403 |
| A\_51\_P242695 | NM\_001011771 | Mus musculus olfactory receptor MOR193-1 (MOR193-1) pseudogene | 1.31 | 0,0034 |
| A\_51\_P324701 | NM\_025606 | Mus musculus mitochondrial ribosomal protein L16 (Mrpl16), mRNA | 1.31 | 0,0117 |
| A\_51\_P381449 | AK015845 | unclassifiable | 1.31 | 0,0032 |
| A\_51\_P288719 | NM\_146031 | Mus musculus, clone MGC:6956 IMAGE:3153901, mRNA, complete cds | 1.31 | 0,0045 |
| A\_51\_P346045 | NM\_053266 | Mus musculus RIKEN cDNA 1700012P16 gene (1700012P16Rik), mRNA | 1.31 | 0,0376 |
| A\_51\_P308148 | NM\_134208 | Mus musculus vomeronasal 1 receptor, G7 (V1rg7), mRNA | 1.30 | 0,0041 |
| A\_51\_P504832 | NM\_207583 | hypothetical Membrane attack complex components/perforin/complement C9 containing protein | 1.30 | 0,0493 |
| A\_51\_P381321 | NM\_199080 | Mus musculus, Similar to DEAD/H (Asp-Glu-Ala-Asp/His) box polypeptide 17 (72kD), clone MGC:38380 IMAGE:5345465, mRNA, complete cds | 1.30 | 0,0448 |
| A\_51\_P150242 | NM\_146744 | Mus musculus olfactory receptor MOR256-8 (MOR256-8), mRNA | 1.30 | 0,0086 |
| A\_51\_P398118 | NM\_134218 | Mus musculus vomeronasal 1 receptor, H9 (V1rh9), mRNA | 1.30 | 0,0350 |
| A\_51\_P329501 | AK037051 | MYOSIN-1D | 1.30 | 0,0080 |
| A\_51\_P356366 | NM\_177190 | SIMILAR TO TESTIS-SPECIFIC PROTEIN PBS13) homolog [Homo sapiens] | 1.30 | 0,0041 |
| A\_51\_P115487 | M36516 | Mouse zinc finger protein (mkr5) mRNA, 3 end | 1.30 | 0,0278 |
| A\_51\_P262616 | AK044521 | hypothetical RabGAP/TBC domain containing protein | 1.30 | 0,0312 |
| A\_51\_P446417 | NM\_026194 | hypothetical protein | 1.30 | 0,0210 |
| A\_51\_P373208 | AK079259 | KIAA0372 PROTEIN homolog [Homo sapiens] | 1.30 | 0,0097 |
| A\_51\_P372472 | NM\_016709 | Mus musculus AU RNA binding protein/enoyl-coenzyme A hydratase (Auh), mRNA | 1.30 | 0,0238 |
| A\_51\_P286399 | NM\_181075 | HISTOCOMPATIBILITY 2, CLASS II ANTIGEN E BETA | 1.30 | 0,0356 |
| A\_51\_P487155 | NM\_175031 | similar to FUSED SERINE/THREONINE KINASE [Homo sapiens] | 1.30 | 0,0403 |
| A\_51\_P299375 | NM\_025591 | Mus musculus RIKEN cDNA 2010309E21 gene (2010309E21Rik), mRNA | 1.30 | 0,0271 |
| A\_51\_P496966 | NM\_007770 | Mus musculus cone-rod homeobox containing gene (Crx), mRNA | 1.30 | 0,0222 |
| A\_51\_P128304 | NM\_146570 | Mus musculus olfactory receptor GA\_x5J8B7W2F7R-2521-3120 (GA\_x5J8B7W2F7R-2521-3120) pseudogene | 1.30 | 0,0212 |
| A\_51\_P157982 | NM\_029930 | Mus musculus 0 day neonate eyeball cDNA, RIKEN full-length enriched library, clone:E130103N08 product:hypothetical protein, full insert sequence. | 1.30 | 0,0478 |
| A\_51\_P137007 | NM\_012039 | Mus musculus, Similar to ZW10 (Drosophila) homolog, centromere/kinetochore protein, clone MGC:11681 IMAGE:3711269, mRNA, complete cds | 1.30 | 0,0344 |
| A\_51\_P392943 | NM\_178728 | hypothetical Metallo-hydrolase/oxidoreductase structure containing protein | 1.30 | 0,0319 |
| A\_51\_P230579 | AK007309 | 1700128E19RIK PROTEIN homolog [Mus musculus] | 1.30 | 0,0052 |
| A\_51\_P310963 | NM\_134170 | similar to VOMERONASAL RECEPTOR V1RC3 [Mus musculus] | 1.30 | 0,0421 |
| A\_51\_P433399 | NM\_139141 | zinc finger protein 192 | 1.30 | 0,0175 |
| A\_51\_P290626 | NM\_178747 | Mus musculus, clone MGC:37880 IMAGE:5101228, mRNA, complete cds | 1.30 | 0,0067 |
| A\_51\_P140811 | BC069869 | hypothetical G-protein beta WD-40 repeats containing protein | 1.30 | 0,0332 |
| A\_51\_P135732 | BC006583 | Mus musculus, Similar to hypothetical protein DKFZp434G156, clone MGC:8047 IMAGE:3587121, mRNA, complete cds | 1.30 | 0,0346 |
| A\_51\_P363644 | NM\_138581 | Mus musculus DNA segment, Human EST J0827E04 (ESTJ0827E04), mRNA | 1.30 | 0,0343 |
| A\_51\_P313602 | NM\_053222 | Mus musculus vomeronasal 1 receptor, A7 (V1ra7), mRNA | 1.30 | 0,0142 |
| A\_51\_P276334 | NAP108615-1 | Mus musculus olfactory receptor MOR162-11P (MOR162-11P) pseudogene | 1.30 | 0,0273 |
| A\_51\_P401924 | NAP057158-1 | Mus musculus olfactory receptor MOR136-18P (MOR136-18P) pseudogene | 1.30 | 0,0126 |
| A\_51\_P329975 | NM\_016718 | Mus musculus ninjurin 2 (Ninj2), mRNA | 1.30 | 0,0337 |
| A\_51\_P210210 | NM\_148947 | Mus musculus lymphocyte antigen 6 complex, locus G5C (Ly6g5c), mRNA | 1.30 | 0,0068 |
| A\_51\_P186374 | NM\_010815 | Mus musculus monocytic adaptor (Mona), mRNA | 1.30 | 0,0088 |
| A\_51\_P155073 | NM\_025399 | Mus musculus, RIKEN cDNA 1110030M18 gene, clone MGC:29286 IMAGE:3986887, mRNA, complete cds | 1.29 | 0,0132 |
| A\_51\_P289351 | NM\_032544 | Mus musculus RIKEN cDNA 2410009F13 gene (2410009F13Rik), mRNA | 1.29 | 0,0178 |
| A\_51\_P514730 | NM\_026615 | Mus musculus, RIKEN cDNA 2900073H19 gene, clone MGC:36675 IMAGE:5368627, mRNA, complete cds | 1.29 | 0,0072 |
| A\_51\_P270583 | AK087617 | inferred: unnamed protein product {Homo sapiens} | 1.29 | 0,0293 |
| A\_51\_P358700 | NM\_146902 | Mus musculus olfactory receptor MOR233-3 (MOR233-3), mRNA | 1.29 | 0,0339 |
| A\_51\_P192397 | NM\_020052 | Mus musculus CUB domain and EGF-like repeat containing 1 (Cegf1), mRNA | 1.29 | 0,0495 |
| A\_51\_P406539 | AK047455 | unknown EST | 1.29 | 0,0066 |
| A\_51\_P250123 | NM\_028543 | similar to MSZF26 (FRAGMENT) [Mus musculus] | 1.29 | 0,0322 |
| A\_51\_P329795 | NM\_145362 | Mus musculus, Similar to beta-1,4 mannosyltransferase, clone MGC:18946 IMAGE:3980821, mRNA, complete cds | 1.29 | 0,0436 |
| A\_51\_P362399 | NM\_176953 | DNA LIGASE IV (EC 6.5.1.1) (POLYDEOXYRIBONUCLEOTIDE SYNTHASE [ATP]) homolog [Homo sapiens] | 1.29 | 0,0386 |
| A\_51\_P151493 | NM\_007526 | Mus musculus BarH-like homeobox 1 (Barx1), mRNA | 1.29 | 0,0201 |
| A\_51\_P441687 | NM\_146242 | Mus musculus putative leucine-rich repeat protein LOC237560 (LOC237560), mRNA | 1.29 | 0,0277 |
| A\_51\_P433388 | AK006101 | hypothetical Glutamic acid-rich region containing protein | 1.29 | 0,0047 |
| A\_51\_P358825 | NM\_175532 | PAAD and NACHT containing protein | 1.29 | 0,0081 |
| A\_51\_P414815 | NM\_147102 | Mus musculus olfactory receptor MOR28-1 (MOR28-1), mRNA | 1.29 | 0,0050 |
| A\_51\_P483130 | AK046696 | hypothetical Histidine acid phosphatase containing protein | 1.29 | 0,0081 |
| A\_51\_P355829 | NM\_010504 | Mus musculus interferon alpha family, gene 4 (Ifna4), mRNA | 1.29 | 0,0071 |
| A\_51\_P235174 | Z83815 | M.musculus mRNA for axonemal dynein heavy chain (partial, ID mdhc7) | 1.29 | 0,0098 |
| A\_51\_P282210 | NM\_020490 | Mus musculus leukotriene B4 receptor 2 (Ltb4r2), mRNA | 1.29 | 0,0181 |
| A\_51\_P189151 | AK028396 | hypothetical BTB/POZ domain containing protein | 1.29 | 0,0192 |
| A\_51\_P322109 | NM\_177242 | T CELL ACTIVATION PROTEIN PHOSPHATASE | 1.29 | 0,0378 |
| A\_51\_P281673 | NM\_015741 | Mus musculus keratin associated protein 9-1 (Krtap9-1), mRNA | 1.29 | 0,0298 |
| A\_51\_P147445 | AK050154 | Leucine-rich repeat, containing protein, | 1.29 | 0,0178 |
| A\_51\_P334199 | NM\_010143 | Mus musculus, Similar to EphB3, clone MGC:18409 IMAGE:3673003, mRNA, complete cds | 1.29 | 0,0228 |
| A\_51\_P322851 | NM\_147083 | Mus musculus olfactory receptor MOR26-1 (MOR26-1), mRNA | 1.29 | 0,0132 |
| A\_51\_P265685 | NM\_026673 | hypothetical protein | 1.29 | 0,0470 |
| A\_51\_P493522 | NM\_178929 | BA108L7.1 (NOVEL INSULIN-LIKE GROWTH FACTOR BINDING TYPE PROTEIN WITH KAZAL-TYPE SERINE PROTEASE INHIBITOR DOMAIN) (FRAGMENT) homolog [Homo sapiens] | 1.29 | 0,0294 |
| A\_51\_P488108 | AF240172 | Mus musculus MRP9 mRNA, partial cds | 1.29 | 0,0110 |
| A\_51\_P361443 | AK028741 | similar to HYPOTHETICAL 21.1 KDA PROTEIN [Homo sapiens] | 1.28 | 0,0390 |
| A\_51\_P258493 | NM\_011067 | Mus musculus period homolog 3 (Drosophila) (Per3), mRNA | 1.28 | 0,0278 |
| A\_51\_P150023 | NM\_027970 | hypothetical protein | 1.28 | 0,0186 |
| A\_51\_P101856 | NM\_007862 | Mus musculus discs, large homolog 1 (Drosophila) (Dlgh1), mRNA | 1.28 | 0,0445 |
| A\_51\_P219527 | NM\_025308 | Mus musculus EST AA238765 (AA238765), mRNA | 1.28 | 0,0178 |
| A\_51\_P142343 | NM\_178912 | hypothetical ERCC4 domain containing protein | 1.28 | 0,0166 |
| A\_51\_P394441 | NM\_013619 | Mus musculus olfactory receptor 67 (Olfr67), mRNA | 1.28 | 0,0333 |
| A\_51\_P456294 | AF151717 | Mus musculus Usher syndrome 2A (autosomal recessive, mild) homolog (human) (Ush2a), mRNA | 1.28 | 0,0306 |
| A\_51\_P231099 | NM\_009996 | Mus musculus cytochrome P450, 24 (Cyp24), mRNA | 1.28 | 0,0162 |
| A\_51\_P450682 | NM\_199455 | hypothetical protein | 1.28 | 0,0335 |
| A\_51\_P303286 | NM\_153108 | Mus musculus mRNA for beta-defensin 8 | 1.28 | 0,0333 |
| A\_51\_P313093 | AK008991 | Mus musculus cDNA clone UI-M-CG0p-bdl-b-12-0-UI 5, mRNA sequence | 1.28 | 0,0225 |
| A\_51\_P423143 | NM\_176828 | hypothetical Zinc carboxypeptidases, carboxypeptidase A metalloprotease (M14) family containing protein | 1.28 | 0,0331 |
| A\_51\_P235856 | NM\_145425 | HOMOLOC-13 homolog [Mus musculus] | 1.28 | 0,0434 |
| A\_51\_P170536 | NM\_170671 | inferred: hypothetical protein {Homo sapiens} | 1.28 | 0,0132 |
| A\_51\_P458140 | NM\_177137 | GUANINE NUCLEOTIDE-BINDING PROTEIN G(OLF), ALPHA SUBUNIT (ADENYLATE CYCLASE-STIMULATING G ALPHA PROTEIN, OLFACTORY TYPE) homolog [Homo sapiens] | 1.28 | 0,0198 |
| A\_51\_P386688 | NM\_133359 | Mus musculus high-glycine/tyrosine protein type I E5 (LOC170939), mRNA | 1.28 | 0,0135 |
| A\_51\_P360262 | NM\_053122 | Mus musculus inner mitochondrial membrane peptidase 2-like (S. cerevisiae) (Immp2l-pending), mRNA | 1.28 | 0,0303 |
| A\_51\_P488068 | ENSMUST00000087819 | Mus musculus olfactory receptor MOR202-32P (MOR202-32P) pseudogene | 1.28 | 0,0191 |
| A\_51\_P366672 | NM\_153170 | similar to LYSOSOMAL AMINO ACID TRANSPORTER 1 [Rattus norvegicus] | 1.28 | 0,0315 |
| A\_51\_P343526 | NM\_008001 | Mus musculus faciogenital dysplasia homolog (Fgd1), mRNA | 1.28 | 0,0291 |
| A\_51\_P513653 | NM\_146738 | Mus musculus olfactory receptor MOR204-9 (MOR204-9), mRNA | 1.28 | 0,0306 |
| A\_51\_P211291 | NM\_025510 | Mus musculus RIKEN cDNA 2310004I24 gene (2310004I24Rik), mRNA | 1.28 | 0,0324 |
| A\_51\_P412325 | AK048533 | HRIHFB2007 PROTEIN (FRAGMENT) homolog [Homo sapiens] | 1.28 | 0,0391 |
| A\_51\_P151243 | NM\_207560 | OLFACTORY RECEPTOR | 1.28 | 0,0189 |
| A\_51\_P398633 | NM\_007418 | Mus musculus adrenergic receptor, alpha 2c (Adra2c), mRNA | 1.28 | 0,0104 |
| A\_51\_P182414 | AK044228 | similar to CAT EYE SYNDROME CRITICAL REGION PROTEIN ISOFORM 1 [Homo sapiens] | 1.28 | 0,0308 |
| A\_51\_P259584 | XM\_148595 | LIMKAIN B1 homolog [Rattus norvegicus] | 1.28 | 0,0165 |
| A\_51\_P196808 | BC038650 | PRE MRNA SPLICING FACTOR RNA HELICASE DEAH BOX PROTEIN | 1.28 | 0,0455 |
| A\_51\_P403243 | NM\_029779 | hypothetical protein | 1.27 | 0,0422 |
| A\_51\_P340603 | AK003717 | SIMILAR TO CHROMOSOME 11 OPEN READING FRAME2 (FRAGMENT) homolog [Homo sapiens] | 1.27 | 0,0348 |
| A\_51\_P463321 | NM\_133872 | hypothetical protein | 1.27 | 0,0494 |
| A\_51\_P353569 | NM\_001011837 | OLFACTORY RECEPTOR | 1.27 | 0,0236 |
| A\_51\_P434557 | NM\_008415 | Mus musculus jerky (Jrk), mRNA | 1.27 | 0,0123 |
| A\_51\_P119387 | NM\_029360 | similar to TRANSMEMBRANE 4 SUPERFAMILY, MEMBER 5 (TETRASPAN TRANSMEMBRANE PROTEIN L6H) [Homo sapiens] | 1.27 | 0,0279 |
| A\_51\_P321820 | AK076022 | hypothetical protein | 1.27 | 0,0209 |
| A\_51\_P388281 | NM\_009835 | Mus musculus chemokine (C-C) receptor 6 (Cmkbr6), mRNA | 1.27 | 0,0325 |
| A\_51\_P211740 | NM\_172825 | CDNA FLJ14454 FIS, CLONE HEMBB1001872, WEAKLY SIMILAR TO CELL SURFACE GLYCOPROTEIN EMR1 PRECURSOR homolog [Homo sapiens] | 1.27 | 0,0130 |
| A\_51\_P175442 | NM\_173024 | weakly similar to SPI2 PROTEINASE INHIBITOR [Mus musculus] | 1.27 | 0,0488 |
| A\_51\_P177310 | NM\_011775 | Mus musculus zona pellucida glycoprotein 2 (Zp2), mRNA | 1.27 | 0,0357 |
| A\_51\_P429770 | NM\_010184 | Mus musculus Fc receptor, IgE, high affinity I, alpha polypeptide (Fcer1a), mRNA | 1.27 | 0,0126 |
| A\_51\_P447743 | AK051581 | unclassifiable | 1.27 | 0,0172 |
| A\_51\_P491545 | NM\_147108 | Mus musculus olfactory receptor MOR223-1 (MOR223-1), mRNA | 1.27 | 0,0095 |
| A\_51\_P317887 | NM\_026907 | Mus musculus secreted and transmembrane 1 (Sectm1), mRNA | 1.27 | 0,0149 |
| A\_51\_P478825 | AK006591 | hypothetical ARM repeat structure containing protein | 1.27 | 0,0226 |
| A\_51\_P361788 | NM\_013933 | Mus musculus vesicle-associated membrane protein, associated protein A (33 kDa) (Vapa), mRNA | 1.27 | 0,0262 |
| A\_51\_P337935 | AK008024 | LARGE NEUTRAL AMINO ACIDS TRANSPORTER SMALL SUBUNIT L TYPE AMINO ACID TRANSPORTER | 1.27 | 0,0344 |
| A\_51\_P313729 | NM\_146334 | Mus musculus olfactory receptor MOR259-8 (MOR259-8), mRNA | 1.27 | 0,0376 |
| A\_51\_P403504 | NM\_146875 | Mus musculus olfactory receptor MOR170-1 (MOR170-1), mRNA | 1.27 | 0,0494 |
| A\_51\_P324484 | AK079063 | unknown EST | 1.27 | 0,0107 |
| A\_51\_P183261 | NM\_007474 | Mus musculus aquaporin 8 (Aqp8), mRNA | 1.27 | 0,0454 |
| A\_51\_P503883 | AK029859 | hypothetical PH domain profile/Dbl domain (dbl/cdc24 rhoGEF family)/Pleckstrin homology (PH) domain containing protein | 1.27 | 0,0364 |
| A\_51\_P464279 | NM\_009757 | Mus musculus bone morphogenetic protein 15 (Bmp15), mRNA | 1.27 | 0,0063 |
| A\_51\_P272303 | NM\_144790 | hypothetical Ankyrin repeat profile/Ankyrin-repeat/Ankyrin repeat region circular profile/Yeast DNA-binding domain containing protein | 1.27 | 0,0175 |
| A\_51\_P141560 | NAP057190-1 | Mus musculus olfactory receptor MOR175-8P (MOR175-8P) pseudogene | 1.27 | 0,0084 |
| A\_51\_P363137 | NM\_030147 | THYROID HORMONE RECEPTOR COACTIVATING PROTEIN homolog [Homo sapiens] | 1.27 | 0,0375 |
| A\_51\_P327713 | NM\_001012402 | HEPARAN SULFATE D GLUCOSAMINYL 3 O SULFOTRANSFERASE | 1.27 | 0,0240 |
| A\_51\_P275283 | AK047234 | similar to SORTING NEXIN 6 (TRAF4-ASSOCIATED FACTOR 2) [Homo sapiens] | 1.27 | 0,0125 |
| A\_51\_P280697 | NM\_008118 | Mus musculus gastric intrinsic factor (Gif), mRNA | 1.27 | 0,0105 |
| A\_51\_P366079 | NM\_146391 | Mus musculus olfactory receptor MOR190-3P (MOR190-3P) pseudogene | 1.27 | 0,0291 |
| A\_51\_P439413 | NAP057235-1 | Mus musculus olfactory receptor MOR234-4P (MOR234-4P) pseudogene | 1.27 | 0,0178 |
| A\_51\_P159213 | A\_51\_P159213 | Mus musculus cDNA, 3 end | 1.27 | 0,0241 |
| A\_51\_P468119 | NM\_024468 | Mus musculus ring finger protein 23 (Rnf23), mRNA | 1.27 | 0,0362 |
| A\_51\_P148102 | NM\_011234 | Mus musculus RAD51 homolog (S. cerevisiae) (Rad51), mRNA | 1.27 | 0,0252 |
| A\_51\_P269709 | NM\_008462 | Mus musculus killer cell lectin-like receptor, subfamily A, member 2 (Klra2), mRNA | 1.27 | 0,0322 |
| A\_51\_P138755 | NM\_177912 | similar to MLZE [Mus musculus] | 1.27 | 0,0117 |
| A\_51\_P402868 | AK046215 | weakly similar to GROUP III SECRETED PHOSPHOLIPASE A2 | 1.27 | 0,0201 |
| A\_51\_P120645 | NM\_027087 | Mus musculus RIKEN cDNA 2300006N05 gene (2300006N05Rik), mRNA | 1.27 | 0,0356 |
| A\_51\_P207275 | AK036825 | unclassifiable | 1.27 | 0,0383 |
| A\_51\_P265792 | NM\_146362 | Mus musculus olfactory receptor MOR245-20 (MOR245-20), mRNA | 1.27 | 0,0248 |
| A\_51\_P193116 | NM\_198642 | weakly similar to CDNA: FLJ21562 FIS, CLONE COL06420 [Homo sapiens] | 1.27 | 0,0262 |
| A\_51\_P229230 | AK005637 | hypothetical Immunoglobulin and major histocompatibility complex domain containing protein | 1.27 | 0,0458 |
| A\_51\_P490509 | NM\_009773 | Mus musculus budding uninhibited by benzimidazoles 1 homolog, beta (S. cerevisiae) (Bub1b), mRNA | 1.27 | 0,0325 |
| A\_51\_P380572 | AK016678 | hypothetical protein | 1.27 | 0,0208 |
| A\_51\_P475858 | NM\_008990 | Mus musculus poliovirus sensitivity (Pvs), mRNA | 1.27 | 0,0062 |
| A\_51\_P186294 | NM\_133203 | Mus musculus Ly-49Q mRNA for NK receptor Ly-49Q, complete cds | 1.27 | 0,0181 |
| A\_51\_P292221 | NM\_010608 | Mus musculus Kcnk3 channel mRNA, complete cds | 1.27 | 0,0174 |
| A\_51\_P169651 | NM\_028310 | similar to MY030 PROTEIN [Homo sapiens] | 1.27 | 0,0486 |
| A\_51\_P135532 | AK021182 | hypothetical protein | 1.27 | 0,0248 |
| A\_51\_P456196 | NM\_001011818 | Mus musculus olfactory receptor MOR285-3P (MOR285-3P) pseudogene | 1.27 | 0,0191 |
| A\_51\_P334459 | NM\_001002789 | hypothetical Vacuolar sorting protein 9 (VPS9) domain containing protein | 1.27 | 0,0095 |
| A\_51\_P168989 | NM\_018792 | Mus musculus histone H1-like protein in spermatids 1 (Hils1), mRNA | 1.27 | 0,0151 |
| A\_51\_P501235 | AK015689 | weakly similar to CDNA FLJ20514 FIS, CLONE KAT09756 (HYPOTHETICAL 28.6 KDA PROTEIN) (SIMILAR TO HYPOTHETICAL PROTEIN FLJ20514) [Homo sapiens] | 1.27 | 0,0395 |
| A\_51\_P370720 | NM\_024290 | Mus musculus tumor necrosis factor receptor superfamily, member 23 (Tnfrsf23), mRNA | 1.27 | 0,0120 |
| A\_51\_P519811 | NM\_018751 | Mus musculus sulfotransferase family 1A, phenol-preferring, member 2 (Sult1a2), mRNA | 1.27 | 0,0161 |
| A\_51\_P480796 | BC070476 | ANTISENSE RNA OVERLAPPING MCH PROTEIN [Rattus norvegicus] | 1.27 | 0,0225 |
| A\_51\_P168239 | NM\_001011814 | OLFACTORY RECEPTOR | 1.27 | 0,0390 |
| A\_51\_P107241 | AK079264 | weakly similar to MHC CLASS I T7 ANTIGEN (FRAGMENT) [Mus musculus] | 1.27 | 0,0245 |
| A\_51\_P201751 | NM\_146904 | Mus musculus olfactory receptor 72 gene (Olfr72), mRNA | 1.27 | 0,0102 |
| A\_51\_P302324 | AA739048 | Mus musculus mRNA for HGT keratin, partial cds | 1.27 | 0,0241 |
| A\_51\_P291860 | ENSMUST00000071587 | Mus musculus olfactory receptor MOR266-3 (MOR266-3), mRNA | 1.27 | 0,0309 |
| A\_51\_P446112 | AK078307 | hypothetical protein | 1.27 | 0,0472 |
| A\_51\_P450812 | NM\_133973 | Mus musculus component of oligomeric golgi complex 4 (Cog4), mRNA | 1.27 | 0,0204 |
| A\_51\_P385684 | NM\_010463 | Mus musculus HOXC12 mRNA, complete cds | 1.27 | 0,0369 |
| A\_51\_P367880 | NM\_008474 | Mus musculus type II 65kD keratin (Krt2-16) mRNA, complete cds | 1.27 | 0,0358 |
| A\_51\_P195124 | NM\_015756 | Mus musculus shroom (shrm), mRNA | 1.27 | 0,0247 |
| A\_51\_P351286 | NM\_008794 | Mus musculus proprotein convertase subtilisin/kexin type 7 (Pcsk7), mRNA | 1.27 | 0,0349 |
| A\_51\_P304743 | XM\_136692 | ATP-BINDING CASSETTE TRANSPORTER FAMILY A MEMBER 12 homolog [Homo sapiens] | 1.27 | 0,0188 |
| A\_51\_P243418 | NM\_027562 | Mus musculus RIKEN cDNA 4632413B12 gene (4632413B12Rik), mRNA | 1.27 | 0,0321 |
| A\_51\_P427132 | NM\_177076 | hypothetical F-box domain containing protein | 1.27 | 0,0304 |
| A\_51\_P411335 | NM\_146964 | Mus musculus olfactory receptor H7 mRNA, partial cds | 1.26 | 0,0063 |
| A\_51\_P107113 | NM\_016694 | Mus musculus parkin (Park2), mRNA | 1.26 | 0,0165 |
| A\_51\_P230155 | NM\_146543 | Mus musculus olfactory receptor MOR256-10 (MOR256-10), mRNA | 1.26 | 0,0275 |
| A\_51\_P416927 | NM\_008763 | Mus musculus olfactory receptor 16 (Olfr16), mRNA | 1.26 | 0,0300 |
| A\_51\_P332755 | AK006654 | hypothetical Lysine-rich region containing protein | 1.26 | 0,0329 |
| A\_51\_P271755 | AA867168 | Mus musculus, clone IMAGE:1380536, mRNA, partial cds | 1.26 | 0,0248 |
| A\_51\_P454313 | BC071238 | weakly similar to MSZF87 (FRAGMENT) [Mus musculus] | 1.26 | 0,0134 |
| A\_51\_P275163 | NM\_146354 | Mus musculus olfactory receptor MOR12-5 (MOR12-5), mRNA | 1.26 | 0,0163 |
| A\_51\_P181922 | XM\_134244 | "Mus musculus similar to apolipoprotein A-I binding protein� apoA-I binding protein [Homo sapiens] (LOC234365), mRNA" | 1.26 | 0,0423 |
| A\_51\_P231464 | NM\_205822 | OM2=cytoplasmically polyadenylated oocyte-specific maternal transcript {clone OM2b} [mice, ovary, mRNA, 727 nt] | 1.26 | 0,0113 |
| A\_51\_P333401 | A\_51\_P333401 | Mus musculus cDNA, 5 end | 1.26 | 0,0305 |
| A\_51\_P479138 | NM\_146547 | Mus musculus olfactory receptor MOR114-8 (MOR114-8), mRNA | 1.26 | 0,0142 |
| A\_51\_P158488 | AK053663 | hypothetical protein | 1.26 | 0,0347 |
| A\_51\_P194609 | NM\_178372 | weakly similar to tryptase (EC 3.4.21.59) III precursor [Homo sapiens] | 1.26 | 0,0241 |
| A\_51\_P276203 | AK087873 | unclassifiable | 1.26 | 0,0212 |
| A\_51\_P251617 | NM\_146897 | Mus musculus olfactory receptor MOR233-8 (MOR233-8), mRNA | 1.26 | 0,0144 |
| A\_51\_P446366 | NM\_031380 | Mus musculus follistatin-like 3 (Fstl3), mRNA | 1.26 | 0,0150 |
| A\_51\_P376333 | NM\_177059 | hypothetical Immunoglobulin and major histocompatibility complex domain containing protein | 1.26 | 0,0189 |
| A\_51\_P171347 | NM\_172937 | hypothetical SNF2 related domain/PHD-finger/DEAD/DEAH box helicase/Histone H1 and H5 family/Cytochrome c family heme-binding site containing protein | 1.26 | 0,0221 |
| A\_51\_P425175 | AK089738 | unknown EST | 1.26 | 0,0229 |
| A\_51\_P427812 | NM\_178638 | hypothetical protein | 1.26 | 0,0203 |
| A\_51\_P424905 | AK086185 | unclassifiable | 1.26 | 0,0459 |
| A\_51\_P344177 | NM\_007401 | a disintegrin and metalloprotease domain 5 | 1.26 | 0,0141 |
| A\_51\_P361858 | BM933347 | unknown EST | 1.26 | 0,0239 |
| A\_51\_P444364 | AK039639 | POU domain, class 4, transcription factor 3 | 1.26 | 0,0193 |
| A\_51\_P218362 | NAP057246-1 | Mus musculus olfactory receptor MOR261-7P (MOR261-7P) pseudogene | 1.26 | 0,0234 |
| A\_51\_P250319 | NM\_009470 | Mus musculus uromodulin mRNA, complete cds | 1.26 | 0,0248 |
| A\_51\_P203800 | NM\_172691 | Mus musculus RIKEN cDNA B230312A22 gene (B230312A22Rik), mRNA | 1.25 | 0,0343 |
| A\_51\_P363396 | NM\_008451 | Mus musculus kinesin light chain 2 (Klc2), mRNA | 1.25 | 0,0307 |
| A\_51\_P459988 | NAP108728-1 | Mus musculus olfactory receptor MOR248-12 (MOR248-12) pseudogene | 1.25 | 0,0193 |
| A\_51\_P517662 | NM\_146754 | Mus musculus olfactory receptor MOR41-1 (MOR41-1), mRNA | 1.25 | 0,0331 |
| A\_51\_P453456 | AK006510 | unknown EST | 1.25 | 0,0234 |
| A\_51\_P408644 | NM\_013719 | Mus musculus eukaryotic translation initiation factor 2 alpha kinase 4 (Eif2ak4), mRNA | 1.25 | 0,0385 |
| A\_51\_P362877 | NM\_021887 | Mus musculus interleukin 21 receptor (Il21r), mRNA | 1.25 | 0,0273 |
| A\_51\_P196862 | AK005066 | CDNA FLJ30055 FIS, CLONE ADRGL1000165, WEAKLY SIMILAR TO IMIDAZOLONEPROPIONASE (EC 3.5.2.7) homolog [Homo sapiens] | 1.25 | 0,0151 |
| A\_51\_P371695 | XM\_620559 | PHOSPHATIDYLINOSITOL 3-KINASE-RELATED PROTEIN KINASE homolog [Homo sapiens] | 1.25 | 0,0190 |
| A\_51\_P115147 | AK087419 | unclassifiable | 1.25 | 0,0464 |
| A\_51\_P292823 | AK019839 | similar to GUANINE NUCLEOTIDE REGULATORY PROTEIN (FRAGMENT) [Mus musculus] | 1.25 | 0,0367 |
| A\_51\_P104954 | NM\_173368 | Mus musculus, Similar to KIAA0308 protein, clone IMAGE:3482324, mRNA | 1.25 | 0,0494 |
| A\_51\_P115374 | AK045286 | hypothetical Nucleic acid-binding proteins structure containing protein | 1.25 | 0,0324 |
| A\_51\_P394612 | AK015851 | T-cell lymphoma invasion and metastasis 1 | 1.25 | 0,0465 |
| A\_51\_P493940 | AK006268 | hypothetical protein | 1.25 | 0,0348 |
| A\_51\_P228175 | XM\_486612 | hypothetical WW / rsp5 / WWP domain containing protein | 1.25 | 0,0242 |
| A\_51\_P480441 | NM\_146934 | Mus musculus olfactory receptor MOR253-10P (MOR253-10P) pseudogene | 1.25 | 0,0155 |
| A\_51\_P295787 | NM\_030741 | Mus musculus pheromone receptor V3R2 (V3R2), mRNA | 1.25 | 0,0335 |
| A\_51\_P230055 | NM\_146504 | ODORANT RECEPTOR M37 (OLFACTORY RECEPTOR MOR224-5) | 1.25 | 0,0153 |
| A\_51\_P274137 | AK009071 | hypothetical protein | 1.25 | 0,0196 |
| A\_51\_P329300 | AK041673 | PROTON MYO-INOSITOL TRANSPORTER homolog [Rattus norvegicus] | 1.25 | 0,0279 |
| A\_51\_P324191 | NM\_009050 | Mus musculus ret proto-oncogene (Ret), mRNA | 1.25 | 0,0495 |
| A\_51\_P136309 | AK014811 | RIKEN cDNA 4921504I02 gene | 1.25 | 0,0252 |
| A\_51\_P328416 | AK020388 | unknown EST | 1.25 | 0,0494 |
| A\_51\_P429715 | X90829 | Mus musculus lady bird-like homeobox 1 homolog, (Drosophila) (Lbx1h), mRNA | 1.25 | 0,0121 |
| A\_51\_P183401 | NM\_146851 | Mus musculus olfactory receptor MOR220-1 (MOR220-1), mRNA | 1.25 | 0,0254 |
| A\_51\_P497502 | AK033258 | mitochondria located 1 homolog (human) | 1.25 | 0,0334 |
| A\_51\_P130439 | NM\_009464 | Mus musculus uncoupling protein 3, mitochondrial (Ucp3), mRNA | 1.25 | 0,0491 |
| A\_51\_P329608 | A\_51\_P329608 | Mus musculus cDNA, 5 end | 1.25 | 0,0270 |
| A\_51\_P100396 | NM\_175475 | Mus musculus cytochrome P450, 26, retinoic acid B1 (Cyp26b1), mRNA | 1.25 | 0,0137 |
| A\_51\_P151732 | NM\_019645 | Mus musculus plakophilin 1 (Pkp1), mRNA | 1.25 | 0,0211 |
| A\_51\_P233801 | NM\_009626 | alcohol dehydrogenase 3 complex | 1.25 | 0,0141 |
| A\_51\_P358840 | NM\_028357 | hypothetical PH domain profile/Pleckstrin homology (PH) domain containing protein | 1.25 | 0,0333 |
| A\_51\_P120987 | AK081466 | phosphodiesterase 4D, cAMP specific | 1.25 | 0,0304 |
| A\_51\_P341688 | NM\_153061 | Mus musculus transforming growth factor beta 1-induced factor 2 mRNA, complete cds | 1.25 | 0,0250 |
| A\_51\_P107839 | NAP057180-1 | Mus musculus olfactory receptor MOR248-13P (MOR248-13P) pseudogene | 1.25 | 0,0337 |
| A\_51\_P153036 | NM\_020502 | Mus musculus candidate taste receptor T2R8 gene (T2r8), mRNA | 1.25 | 0,0194 |
| A\_51\_P517779 | NM\_147015 | Mus musculus olfactory receptor MOR180-1 (MOR180-1), mRNA | 1.25 | 0,0398 |
| A\_51\_P159755 | NM\_153393 | Mus musculus procollagen, type XXIII, alpha 1 (Col23a1), mRNA | 1.25 | 0,0411 |
| A\_51\_P422934 | NM\_146954 | Mus musculus olfactory receptor MOR253-7 (MOR253-7), mRNA | 1.25 | 0,0158 |
| A\_51\_P389255 | NM\_146318 | Mus musculus olfactory receptor MOR115-1 (MOR115-1), mRNA | 1.24 | 0,0346 |
| A\_51\_P167263 | NM\_007650 | Mus musculus CD5 antigen (Cd5), mRNA | 1.24 | 0,0305 |
| A\_51\_P183723 | XM\_283635 | weakly similar to SQUAMOUS CELL CARCINOMA ANTIGEN RECOGNIZED BY T CELL [Homo sapiens] | 1.24 | 0,0338 |
| A\_51\_P217360 | AK046418 | Mus musculus cDNA, 3 end | 1.24 | 0,0165 |
| A\_51\_P175549 | NM\_146567 | Mus musculus olfactory receptor MOR155-2 (MOR155-2), mRNA | 1.24 | 0,0312 |
| A\_51\_P328818 | NM\_207673 | Mus musculus olfactory receptor GA\_x5J8B7W5KGR-1200441-1200915 (GA\_x5J8B7W5KGR-1200441-1200915) pseudogene | 1.24 | 0,0113 |
| A\_51\_P253426 | NM\_146737 | Mus musculus olfactory receptor MOR204-10 (MOR204-10), mRNA | 1.24 | 0,0358 |
| A\_51\_P310387 | NM\_020613 | Mus musculus epidymal sperm gene (X99300), mRNA | 1.24 | 0,0311 |
| A\_51\_P460230 | AK005864 | hypothetical protein | 1.24 | 0,0143 |
| A\_51\_P510929 | NM\_207145 | OLFACTORY RECEPTOR MOR150-2 | 1.24 | 0,0105 |
| A\_51\_P410219 | NM\_027633 | HYPOTHETICAL 20.2 KDA PROTEIN homolog [Homo sapiens] | 1.24 | 0,0214 |
| A\_51\_P514439 | NM\_020274 | Mus musculus 5-hydroxytryptamine (serotonin) receptor 3B (Htr3b), mRNA | 1.24 | 0,0392 |
| A\_51\_P511329 | NM\_008966 | prostaglandin F receptor | 1.24 | 0,0294 |
| A\_51\_P437802 | AK037643 | unclassifiable | 1.24 | 0,0256 |
| A\_51\_P316661 | NM\_031192 | Mus musculus renin 1 structural (Ren1), mRNA | 1.24 | 0,0359 |
| A\_51\_P348922 | NM\_146921 | Mus musculus olfactory receptor MOR135-13 (MOR135-13), mRNA | 1.24 | 0,0479 |
| A\_51\_P388895 | NM\_178791 | hypothetical Immunoglobulin and major histocompatibility complex domain containing protein | 1.24 | 0,0424 |
| A\_51\_P184949 | NM\_021489 | Mus musculus coagulation factor XII (Hageman factor) (F12), mRNA | 1.24 | 0,0279 |
| A\_51\_P451226 | AK015074 | 3-phosphoglycerate dehydrogenase | 1.24 | 0,0400 |
| A\_51\_P257175 | AK036802 | similar to KERATIN, TYPE II CYTOSKELETAL 2 ORAL (CYTOKERATIN 2P) (K2P) (CK 2P) [Homo sapiens] | 1.24 | 0,0332 |
| A\_51\_P477618 | AK077101 | Mus musculus, clone MGC:25529 IMAGE:3586097, mRNA, complete cds | 1.24 | 0,0483 |
| A\_51\_P195534 | BC003460 | Mus musculus RIKEN cDNA 5430429D21 gene (5430429D21Rik), mRNA | 1.24 | 0,0448 |
| A\_51\_P261374 | NAP057088-1 | Mus musculus olfactory receptor GA\_x5J8B7W6337-1270568-1271367 (GA\_x5J8B7W6337-1270568-1271367) pseudogene | 1.24 | 0,0311 |
| A\_51\_P518014 | NM\_172258 | Mus musculus proton/amino acid transporter 3 (PAT3), mRNA | 1.24 | 0,0304 |
| A\_51\_P154272 | NM\_146349 | Mus musculus olfactory receptor MOR176-3 (MOR176-3), mRNA | 1.24 | 0,0298 |
| A\_51\_P486350 | NM\_001018013 | hypothetical Immunoglobulin structure containing protein | 1.24 | 0,0239 |
| A\_51\_P157118 | NM\_008458 | Mus musculus serine (or cysteine) proteinase inhibitor, clade A, member 3C (Serpina3c), mRNA | 1.24 | 0,0494 |
| A\_51\_P345306 | NM\_177142 | TRIACYLGLYCEROL LIPASE, PANCREATIC PRECURSOR EC 3.1.1.3 PANCREATIC LIPASE | 1.24 | 0,0479 |
| A\_51\_P216369 | L27105 | Mus musculus neurofibromatosis 2 (Nf2), mRNA | 1.24 | 0,0163 |
| A\_51\_P211171 | AK002608 | Mus musculus 3-hydroxyanthranilate 3,4-dioxygenase (Haao), mRNA | 1.24 | 0,0396 |
| A\_51\_P197910 | XM\_358375 | hypothetical protein | 1.23 | 0,0214 |
| A\_51\_P111234 | NM\_010077 | Mus musculus dopamine receptor 2 (Drd2), mRNA | 1.23 | 0,0243 |
| A\_51\_P306770 | AK018199 | hypothetical protein | 1.23 | 0,0274 |
| A\_51\_P173795 | NM\_146240 | PAM COOH-TERMINAL INTERACTOR PROTEIN 1 homolog [Rattus norvegicus] | 1.23 | 0,0311 |
| A\_51\_P160187 | S64154 | dystrophin {5 region, alternatively spliced} [mice, cerebellar Purkinje neurons, mRNA Partial, 330 nt] | 1.23 | 0,0286 |
| A\_51\_P283151 | NM\_028995 | hypothetical protein | 1.23 | 0,0110 |
| A\_51\_P151020 | NM\_009407 | Mus musculus transition protein 1 (Tnp1), mRNA | 1.23 | 0,0335 |
| A\_51\_P484859 | NM\_146951 | Mus musculus olfactory receptor MOR136-1 (MOR136-1), mRNA | 1.23 | 0,0457 |
| A\_51\_P237879 | NM\_027671 | Mus musculus syntrophin, gamma 1 (Sntg1), mRNA | 1.23 | 0,0227 |
| A\_51\_P401580 | NM\_010030 | Mus musculus defensin beta 2 (Defb2), mRNA | 1.23 | 0,0232 |
| A\_51\_P441807 | NM\_183137 | hypothetical Serine-rich region containing protein | 1.23 | 0,0263 |
| A\_51\_P497931 | NM\_175452 | Mus musculus gap junction membrane channel protein alpha 12 (Gja12-pending), mRNA | 1.23 | 0,0349 |
| A\_51\_P266695 | NM\_172835 | Mus musculus, Similar to pellino homolog 2 (Drosophila), clone MGC:30417 IMAGE:5037308, mRNA, complete cds | 1.23 | 0,0224 |
| A\_51\_P428832 | AK014640 | similar to HIGH-MOBILITY GROUP PROTEIN 2-LIKE 1 (HMGBCG PROTEIN) [Homo sapiens] | 1.23 | 0,0473 |
| A\_51\_P187782 | NM\_018879 | Mus musculus nitrogen permase homolog (S. cerevisiae) (Nprl2-pending), mRNA | 1.23 | 0,0357 |
| A\_51\_P504879 | NM\_146529 | Mus musculus olfactory receptor MOR230-10P (MOR230-10P) pseudogene | 1.23 | 0,0440 |
| A\_51\_P325889 | AK037440 | unknown EST | 1.23 | 0,0251 |
| A\_51\_P120027 | AK004863 | similar to ALCOHOL DEHYDROGENASE 2 (EC 1.1.1.1) [Peromyscus maniculatus] | 1.23 | 0,0142 |
| A\_51\_P349713 | NM\_022016 | Mus musculus interphotoreceptor matrix proteoglycan 1 (Impg1), mRNA | 1.23 | 0,0173 |
| A\_51\_P233855 | NM\_172750 | ADP RIBOSYLARGININE HYDROLASE EC 3.2.2.19 ADP RIBOSE L ARGININE CLEAVING | 1.23 | 0,0272 |
| A\_51\_P128913 | NM\_001005520 | Mus musculus olfactory receptor MOR105-4 (MOR105-4), mRNA | 1.23 | 0,0419 |
| A\_51\_P493627 | NM\_194334 | hypothetical RabGAP/TBC domain/Pleckstrin homology (PH) domain containing protein | 1.23 | 0,0248 |
| A\_51\_P504737 | NM\_146791 | Mus musculus olfactory receptor MOR231-10 (MOR231-10), mRNA | 1.23 | 0,0199 |
| A\_51\_P107985 | AK006597 | hypothetical protein | 1.23 | 0,0320 |
| A\_51\_P185693 | NM\_031197 | Mus musculus solute carrier family 2 (facilitated glucose transporter), member 2 (Slc2a2), mRNA | 1.23 | 0,0296 |
| A\_51\_P447946 | NM\_016859 | Mus musculus bystin-like (Bysl), mRNA | 1.23 | 0,0495 |
| A\_51\_P187184 | XM\_355941 | inferred: serine/threonine protein kinase TAO2 {Rattus norvegicus} | 1.23 | 0,0192 |
| A\_51\_P378990 | NM\_026100 | Mus musculus RIKEN cDNA 1700055O19 gene (1700055O19Rik), mRNA | 1.23 | 0,0382 |
| A\_51\_P136810 | NM\_001004025 | PP2B beta 2=calmodulin-dependent protein phosphatase regulatory subunit beta 2 isoform [mice, testis, mRNA, 784 nt] | 1.23 | 0,0466 |
| A\_51\_P457734 | NM\_010332 | Mus musculus endothelin receptor subtype A (ET-AR) mRNA, partial cds | 1.23 | 0,0335 |
| A\_51\_P140501 | A\_51\_P140501 | SELENIDE,WATER DIKINASE EC 2.7.9.3 SELENOPHOSPHATE SYNTHETASE SELENIUM DONOR PROTEIN | 1.23 | 0,0427 |
| A\_51\_P342117 | NM\_007675 | Mus musculus CEA-related cell adhesion molecule 10 (Ceacam10), mRNA | 1.22 | 0,0191 |
| A\_51\_P478748 | NM\_175647 | Mus musculus doublesex and mab-3 related transcription factor 4 (Dmrt4), mRNA | 1.22 | 0,0309 |
| A\_51\_P155582 | NM\_009013 | Mus musculus RAD51 associated protein 1 (Rad51ap1), mRNA | 1.22 | 0,0166 |
| A\_51\_P453303 | AK029737 | NEURABIN-I (NEURAL TISSUE-SPECIFIC F-ACTIN BINDING PROTEIN I) (PROTEIN PHOSPHATASE 1 REGULATORY SUBUNIT 9A) (P180) (PP1BP175) homolog [Rattus norvegicus] | 1.22 | 0,0295 |
| A\_51\_P388310 | NM\_009873 | Mus musculus cyclin-dependent kinase 6 (Cdk6), mRNA | 1.22 | 0,0478 |
| A\_51\_P230663 | AK044887 | GAMMA-TUBULIN COMPLEX COMPONENT GCP5 homolog [Homo sapiens] | 1.22 | 0,0454 |
| A\_51\_P506835 | AK033449 | POZ 56 PROTEIN homolog [Mus musculus] | 1.22 | 0,0290 |
| A\_51\_P118230 | NM\_028078 | Mus musculus, RIKEN cDNA 2010003D20 gene, clone MGC:7960 IMAGE:3584645, mRNA, complete cds | 1.22 | 0,0274 |
| A\_51\_P110791 | NM\_009212 | Mus musculus immunoglobulin mu binding protein 2 (Ighmbp2), mRNA | 1.22 | 0,0211 |
| A\_51\_P495550 | NM\_001011830 | Mus musculus olfactory receptor MOR263-7 (MOR263-7) pseudogene | 1.22 | 0,0265 |
| A\_51\_P388835 | NM\_009380 | Mus musculus thyroid hormone receptor beta (Thrb), mRNA | 1.22 | 0,0438 |
| A\_51\_P196995 | NM\_146064 | Mus musculus sterol O-acyltransferase 2 (Soat2), mRNA | 1.22 | 0,0472 |
| A\_51\_P487004 | NM\_027732 | similar to DOUBLESEX-MAB-3 (DM) DOMAIN (FRAGMENT) [Homo sapiens] | 1.22 | 0,0391 |
| A\_51\_P106169 | NM\_011829 | Mus musculus inosine 5-phosphate dehydrogenase 1 (Impdh1), mRNA | 1.22 | 0,0139 |
| A\_51\_P171086 | AK083525 | unknown EST | 1.22 | 0,0192 |
| A\_51\_P108812 | NM\_011312 | Mus musculus S100 calcium binding protein A5 (S100a5), mRNA | 1.22 | 0,0411 |
| A\_51\_P506843 | NM\_009504 | Mus musculus vitamin D receptor (Vdr), mRNA | 1.22 | 0,0495 |
| A\_51\_P103558 | XM\_146397 | unknown | 1.22 | 0,0355 |
| A\_51\_P142350 | AK016986 | unknown EST | 1.22 | 0,0216 |
| A\_51\_P478708 | NAP057242-1 | Mus musculus olfactory receptor MOR264-13P (MOR264-13P) pseudogene | 1.22 | 0,0241 |
| A\_51\_P477553 | TC1435668 | Mus musculus cDNA, 5 end | 1.22 | 0,0271 |
| A\_51\_P214552 | ENSMUST00000084596 | Mus musculus olfactory receptor GA\_x5J8B7W4TKW-7041228-7041658 (GA\_x5J8B7W4TKW-7041228-7041658) pseudogene | 1.22 | 0,0315 |
| A\_51\_P147662 | NM\_207553 | OLFACTORY RECEPTOR | 1.22 | 0,0311 |
| A\_51\_P449657 | AK005459 | weakly similar to ferritin heavy chain 2 (fragment) [Sus scrofa domestica] | 1.22 | 0,0421 |
| A\_51\_P510573 | BC030003 | Similar to: Mus musculus MAD homolog 2 (Drosophila) (Madh2), mRNA | 1.22 | 0,0317 |
| A\_51\_P148139 | AI303461 | Mus musculus cDNA, 3 end | 1.21 | 0,0309 |
| A\_51\_P211978 | NM\_146604 | Mus musculus olfactory receptor MOR260-2 (MOR260-2), mRNA | 1.21 | 0,0334 |
| A\_51\_P401073 | NM\_028636 | SIMILAR TO MANNOSIDASE, ALPHA, CLASS 2C, MEMBER 1 homolog [Mus musculus] | 1.21 | 0,0365 |
| A\_51\_P109643 | NAP057031-1 | Mus musculus ribosomal protein S12, pseudogene 1 (Rps12-ps1) on chromosome X | 1.21 | 0,0210 |
| A\_51\_P341540 | NM\_010068 | Mus musculus DNA methyltransferase 3B (Dnmt3b), mRNA | 1.21 | 0,0423 |
| A\_51\_P371647 | NM\_147037 | Mus musculus olfactory receptor MOR208-1 (MOR208-1), mRNA | 1.21 | 0,0359 |
| A\_51\_P241375 | NM\_052824 | Mus musculus FXYD domain-containing ion transport regulator 2 (Fxyd2), transcript variant c, mRNA | 1.21 | 0,0407 |
| A\_51\_P346252 | NM\_010352 | Mus musculus germ cell-specific gene 1 (Gsg1), mRNA | 1.21 | 0,0385 |
| A\_51\_P170725 | NM\_028788 | hypothetical protein | 1.21 | 0,0268 |
| A\_51\_P205028 | NM\_008007 | Mus musculus fibroblast growth factor 3 (Fgf3), mRNA | 1.21 | 0,0240 |
| A\_51\_P156740 | NM\_011851 | Mus musculus 5 nucleotidase, ecto (Nt5e), mRNA | 1.21 | 0,0457 |
| A\_51\_P177323 | NAP101619-1 | ALKALINE PHOSPHATASE, PRECURSOR EC 3.1.3.1 | 1.21 | 0,0324 |
| A\_51\_P426032 | NM\_177839 | FICOLIN PRECURSOR COLLAGEN/FIBRINOGEN DOMAIN CONTAINING PROTEIN FICOLIN FICOLIN | 1.21 | 0,0365 |
| A\_51\_P449644 | TC1427190 | Mus musculus cDNA, 5 end | 1.21 | 0,0240 |
| A\_51\_P378415 | AK032400 | similar to WUGSC:H\_RG122E10.2B PROTEIN [Homo sapiens] | 1.21 | 0,0484 |
| A\_51\_P252519 | NM\_053269 | Mus musculus Rad51 homolog c (S. cerevisiae) (Rad51c), mRNA | 1.21 | 0,0267 |
| A\_51\_P490879 | TC1521143 | Mus musculus cDNA, 3 end | 1.21 | 0,0391 |
| A\_51\_P504517 | NM\_027083 | similar to LYSOZYME HOMOLOG [Homo sapiens] | 1.21 | 0,0398 |
| A\_51\_P246181 | AK044844 | cytosolic 5 nucleotidase, type 1A | 1.21 | 0,0373 |
| A\_51\_P124603 | NM\_146934 | Mus musculus olfactory receptor 46 (Olfr46), mRNA | 1.21 | 0,0448 |
| A\_51\_P179051 | NM\_146809 | Mus musculus olfactory receptor MOR239-7 (MOR239-7) pseudogene | 1.21 | 0,0245 |
| A\_51\_P406193 | AK020389 | hypothetical protein | 1.21 | 0,0341 |
| A\_51\_P126258 | NM\_033584 | Mus musculus protocadherin gamma subfamily A, 1 (Pcdhga1), mRNA | 1.21 | 0,0287 |
| A\_51\_P114105 | BC052872 | MYOPALLADIN homolog [Homo sapiens] | 1.21 | 0,0382 |
| A\_51\_P393323 | A\_51\_P393323 | Mus musculus cDNA, 3 end | 1.21 | 0,0392 |
| A\_51\_P123314 | NM\_054091 | Mus musculus olfactory receptor 74 (Olfr74), mRNA | 1.21 | 0,0376 |
| A\_51\_P249909 | NM\_010702 | Mus musculus leukocyte cell-derived chemotaxin 2 (Lect2), mRNA | 1.21 | 0,0340 |
| A\_51\_P361675 | AK047998 | hypothetical protein | 1.21 | 0,0385 |
| A\_51\_P226831 | NM\_028628 | Mus musculus RIKEN cDNA 1110008K04 gene (1110008K04Rik), mRNA | 1.21 | 0,0415 |
| A\_51\_P393288 | NM\_146988 | Mus musculus olfactory receptor MOR261-1 (MOR261-1), mRNA | 1.21 | 0,0278 |
| A\_51\_P507644 | AW476024 | Mus musculus cDNA, 3 end | 1.21 | 0,0357 |
| A\_51\_P452367 | NM\_013675 | Mus musculus beta-spectrin 1 (Spnb1), mRNA | 1.21 | 0,0450 |
| A\_51\_P318140 | NM\_146943 | Mus musculus olfactory receptor MOR136-9 (MOR136-9), mRNA | 1.21 | 0,0409 |
| A\_51\_P417691 | BE992497 | Mus musculus cDNA, 3 end | 1.21 | 0,0468 |
| A\_51\_P359586 | XM\_284750 | M.musculus mRNA for 6-phosphofructo-2-kinase/fructose-2,6-bisphosphatase, clone 500bC6 | 1.21 | 0,0483 |
| A\_51\_P244879 | AB041806 | Mus musculus hypothetical protein, MNCb-2457 (AB041806), mRNA | 1.21 | 0,0396 |
| A\_51\_P205233 | NAP057132-1 | Mus musculus olfactory receptor GA\_x5J8B7W4CQV-104617-103730 (GA\_x5J8B7W4CQV-104617-103730) pseudogene | 1.21 | 0,0285 |
| A\_51\_P230122 | NM\_146777 | Mus musculus olfactory receptor MOR110-2 (MOR110-2), mRNA | 1.21 | 0,0337 |
| A\_51\_P283997 | AK051490 | unknown EST | 1.21 | 0,0322 |
| A\_51\_P204153 | NM\_010518 | Mus musculus insulin-like growth factor binding protein 5 (Igfbp5), mRNA | 1.21 | 0,0478 |
| A\_51\_P319527 | NAP057288-1 | Mus musculus olfactory receptor MOR177-11P (MOR177-11P) pseudogene | 1.20 | 0,0478 |
| A\_51\_P268812 | NAP057061-1 | Mus musculus olfactory receptor GA\_x5J8B7W89HK-6403592-6404251 (GA\_x5J8B7W89HK-6403592-6404251) pseudogene | 1.20 | 0,0497 |
| A\_51\_P472148 | NM\_011271 | Mus musculus ribonuclease 1, pancreatic (Rib1), mRNA | 1.20 | 0,0445 |
| A\_51\_P198800 | AK003363 | haemopoiesis related membrane protein 1 | 1.20 | 0,0423 |
| A\_51\_P415095 | AK083149 | synapsin I | 1.20 | 0,0415 |
| A\_51\_P413660 | AK041728 | unknown EST | 1.20 | 0,0493 |
| A\_51\_P196019 | NM\_011643 | Mus musculus transient receptor protein 1 (Trrp1), mRNA | 1.20 | 0,0361 |
| A\_51\_P362423 | NM\_010841 | Mus musculus metallothionein-like 5, testis-specific (tesmin) (Mtl5), mRNA | 1.20 | 0,0499 |
| A\_51\_P493649 | NM\_023135 | Mus musculus sulfotransferase, estrogen preferring (Ste), mRNA | 1.20 | 0,0392 |
| A\_51\_P180602 | NM\_146339 | Mus musculus mRNA for 18A olfactory receptor protein | 1.20 | 0,0349 |
| A\_51\_P320953 | NM\_153416 | Mus musculus, clone MGC:38135 IMAGE:5321013, mRNA, complete cds | 1.20 | 0,0309 |
| A\_51\_P474038 | TC1525739 | Mus musculus cDNA, 5 end | 1.20 | 0,0471 |
| A\_51\_P247472 | AK046664 | hypothetical protein | 1.19 | 0,0494 |
| A\_51\_P233338 | NM\_146934 | Mus musculus olfactory receptor F5 mRNA, partial cds | 1.19 | 0,0327 |
| A\_51\_P158238 | AK087696 | hypothetical CD9/CD37/CD63 antigens containing protein | 1.19 | 0,0346 |
| A\_51\_P358462 | NM\_133689 | Mus musculus RIKEN cDNA 4930579J09 gene (4930579J09Rik), mRNA | 1.19 | 0,0392 |
| A\_51\_P154637 | NM\_175290 | hypothetical Leucine rich repeat, ribonuclease inhibitor type containing protein | 1.19 | 0,0314 |
| A\_51\_P443958 | AI838745 | Mus musculus cDNA, 3 end | 1.19 | 0,0386 |
| A\_51\_P173801 | NM\_134178 | Mus musculus vomeronasal 1 receptor, C23 (V1rc23), mRNA | 1.19 | 0,0397 |
| A\_51\_P224938 | NM\_173372 | METABOTROPIC GLUTAMATE RECEPTOR PRECURSOR | 1.19 | 0,0319 |
| A\_51\_P459770 | AK048388 | REVERSE TRANSCRIPTASE homolog [Mus musculus] | 1.19 | 0,0422 |
| A\_51\_P483454 | NM\_177724 | hypothetical Glycine-rich region/Serine-rich region containing protein | 1.19 | 0,0494 |
| A\_51\_P388329 | NM\_010678 | Mus musculus mRNA similar to lymphoid nuclear protein related to AF4 (cDNA clone MGC:62452 IMAGE:5702935), complete cds | 1.19 | 0,0499 |
| A\_51\_P416278 | AK038734 | hypothetical protein | 1.19 | 0,0487 |
| A\_51\_P135357 | NM\_025679 | Mus musculus RIKEN cDNA 5730470L24 gene (5730470L24Rik), mRNA | 1.18 | 0,0441 |
| A\_51\_P103706 | NM\_007815 | Mus musculus cytochrome P450, 2c29 (Cyp2c29), mRNA | 1.18 | 0,0426 |
| A\_51\_P198453 | NM\_145710 | Mus musculus oocyte specific homeobox 6 (Obox6), mRNA | 1.17 | 0,0460 |
| A\_51\_P285651 | AK033341 | weakly similar to CYCLIN-DEPENDENT KINASE INHIBITOR 3 (EC 3.1.3.48) [Sus scrofa] | 1.17 | 0,0488 |
| A\_51\_P103129 | A\_51\_P103129 | Mus musculus cDNA, 3 end | 1.17 | 0,0494 |
| A\_51\_P105424 | NM\_029831 | similar to tissue kallikrein (EC 3.4.21.35), submandibular mGK-2 (fragment) [Mus musculus] | 0.85 | 0,0376 |
| A\_51\_P316042 | NM\_175362 | CARD-CONTAINING MAGUK PROTEIN CARMA1 homolog [Homo sapiens] | 0.84 | 0,0482 |
| A\_51\_P176549 | NM\_019774 | Mus musculus A kinase (PRKA) anchor protein 8 (Akap8), mRNA | 0.84 | 0,0416 |
| A\_51\_P234263 | NM\_011229 | Mus musculus RAB5B, member RAS oncogene family (Rab5b), mRNA | 0.84 | 0,0284 |
| A\_51\_P179194 | NM\_028562 | Mus musculus RIKEN cDNA 1700080E11 gene (1700080E11Rik), mRNA | 0.84 | 0,0444 |
| A\_51\_P343336 | XM\_355782 | IG KAPPA CHAIN V-V REGION K2 PRECURSOR (FRAGMENT). | 0.83 | 0,0263 |
| A\_51\_P461245 | AK079396 | myosin-light-chain kinase (EC 2.7.1.117), skeletal muscle homolog [Rattus norvegicus] | 0.83 | 0,0434 |
| A\_51\_P468054 | NM\_009762 | Mus musculus CD8beta opposite strand (Bop), mRNA | 0.83 | 0,0266 |
| A\_51\_P294402 | NM\_153790 | Mus musculus scavenger receptor class F, member 2 (Scarf2), mRNA | 0.83 | 0,0411 |
| A\_51\_P140042 | XM\_486166 | hypothetical Double-stranded RNA binding (DsRBD) domain/Adenosine-deaminase (editase) domain containing protein | 0.82 | 0,0429 |
| A\_51\_P261388 | NM\_172948 | hypothetical protein | 0.82 | 0,0420 |
| A\_51\_P219098 | NM\_027019 | Mus musculus RIKEN cDNA 1700011O04 gene (1700011O04Rik), mRNA | 0.82 | 0,0343 |
| A\_51\_P506915 | NM\_019762 | Mus musculus plakophilin 3 (Pkp3), mRNA | 0.82 | 0,0479 |
| A\_51\_P453312 | AU067806 | Mus musculus cDNA, 3 end | 0.82 | 0,0429 |
| A\_51\_P187444 | NM\_011242 | Mus musculus RAS, guanyl releasing protein 2 (Rasgrp2), mRNA | 0.82 | 0,0417 |
| A\_51\_P483739 | NM\_025824 | Mus musculus basic leucine zipper and W2 domains 1 (Bzw1), mRNA | 0.82 | 0,0235 |
| A\_51\_P465161 | NM\_026152 | Mus musculus RIKEN cDNA 0610010D20 gene (0610010D20Rik), mRNA | 0.82 | 0,0443 |
| A\_51\_P464710 | NM\_172133 | Mus musculus, Similar to Centaurin-alpha2 protein, clone MGC:36547 IMAGE:4951680, mRNA, complete cds | 0.82 | 0,0495 |
| A\_51\_P394973 | NM\_026598 | EMOPAMIL BINDING RELATED PROTEIN homolog [Mus musculus] | 0.82 | 0,0499 |
| A\_51\_P185627 | NM\_172412 | GLYPICAN-2 PRECURSOR (CEREBROGLYCAN) (HSPG M13) homolog [Rattus norvegicus] | 0.82 | 0,0388 |
| A\_51\_P318577 | AK010729 | weakly similar to SULFOTRANSFERASE [Gallus gallus] | 0.81 | 0,0477 |
| A\_51\_P412508 | NM\_018885 | Mus musculus iroquois related homeobox 4 (Drosophila) (Irx4), mRNA | 0.81 | 0,0409 |
| A\_51\_P169576 | A\_51\_P169576 | Mus musculus high mobility group box 1, related sequence 16 (Hmgb1-rs16) pseudogene | 0.81 | 0,0488 |
| A\_51\_P461902 | L22886 | Mus musculus rearranged IgH mRNA, V-region, cell line Cyd-1 | 0.81 | 0,0379 |
| A\_51\_P486610 | NM\_054070 | Mus musculus AFG3(ATPase family gene 3)-like 1 (yeast) (Afg3l1), mRNA | 0.81 | 0,0375 |
| A\_51\_P485421 | AK088666 | Mus musculus immunoglobulin heavy chain 6 (heavy chain of IgM), mRNA (cDNA clone MGC:18788 IMAGE:4189350), complete cds | 0.81 | 0,0382 |
| A\_51\_P384187 | NM\_011339 | Mus musculus small inducible cytokine subfamily B, member 15 (Scyb15), mRNA | 0.81 | 0,0466 |
| A\_51\_P268297 | AK039504 | weakly similar to KH TYPE SPLICING REGULATORY PROTEIN (FRAGMENT) [Homo sapiens] | 0.81 | 0,0494 |
| A\_51\_P443472 | NM\_025392 | Mus musculus RIKEN cDNA 1110013J05 gene (1110013J05Rik), mRNA | 0.81 | 0,0340 |
| A\_51\_P372156 | AK044443 | hypothetical protein | 0.81 | 0,0278 |
| A\_51\_P512899 | NM\_007551 | Mus musculus Burkitt lymphoma receptor 1 (Blr1), mRNA | 0.80 | 0,0388 |
| A\_51\_P372372 | NM\_146305 | Mus musculus olfactory receptor MOR105-10 (MOR105-10), mRNA | 0.80 | 0,0230 |
| A\_51\_P360274 | NM\_052977 | Mus musculus adenosine deaminase 3, RNA dependent (Adar3-pending), mRNA | 0.80 | 0,0472 |
| A\_51\_P360655 | NM\_008766 | Mus musculus solute carrier family 22 (organic anion transporter), member 6 (Slc22a6), mRNA | 0.80 | 0,0359 |
| A\_51\_P390531 | NM\_172663 | ENHANCER OF POLYCOMB 1 homolog [Homo sapiens] | 0.80 | 0,0496 |
| A\_51\_P123320 | AK019493 | hypothetical protein | 0.80 | 0,0236 |
| A\_51\_P248917 | NM\_007560 | Mus musculus bone morphogenetic protein receptor, type 1B (Bmpr1b), mRNA | 0.80 | 0,0408 |
| A\_51\_P389004 | NM\_011891 | Mus musculus sarcoglycan, delta (35kD dystrophin-associated glycoprotein) (Sgcd), mRNA | 0.80 | 0,0297 |
| A\_51\_P225263 | NM\_029792 | Mus musculus GlcAT-P mRNA for UDP-glucuronyltransferase-P, complete cds | 0.80 | 0,0392 |
| A\_51\_P408269 | NM\_013547 | Mus musculus homogentisate 1, 2-dioxygenase (Hgd), mRNA | 0.80 | 0,0181 |
| A\_51\_P381584 | NM\_021433 | Mus musculus syntaxin 6 (Stx6), mRNA | 0.80 | 0,0426 |
| A\_51\_P164127 | NM\_011711 | Mus musculus WW domain binding protein 3 mRNA, partial cds | 0.80 | 0,0271 |
| A\_51\_P380919 | NM\_027036 | hypothetical HMG1/2 (high mobility group) box containing protein | 0.80 | 0,0359 |
| A\_51\_P142100 | NM\_008057 | Mus musculus frizzled homolog 7 (Drosophila) (Fzd7), mRNA | 0.80 | 0,0441 |
| A\_51\_P507709 | NM\_173772 | SIALIDASE 3 EC 3.2.1.18 MEMBRANE SIALIDASE GANGLIOSIDE SIALIDASE N ACETYL ALPHA NEURAMINIDASE | 0.80 | 0,0262 |
| A\_51\_P333923 | NM\_133681 | Mus musculus RIKEN cDNA 9030418M05 gene (9030418M05Rik), mRNA | 0.80 | 0,0157 |
| A\_51\_P323180 | NM\_172777 | similar to GUANYLATE BINDING PROTEIN [Mus musculus] | 0.80 | 0,0393 |
| A\_51\_P321158 | NM\_017372 | Mus musculus lysozyme (Lyzs), mRNA | 0.80 | 0,0405 |
| A\_51\_P169415 | NM\_133167 | Mus musculus parvin, beta (Parvb), mRNA | 0.80 | 0,0263 |
| A\_51\_P278070 | BE980334 | Mus musculus cDNA clone UI-M-BG2-bch-e-03-0-UI 3. | 0.80 | 0,0283 |
| A\_51\_P279050 | NM\_008444 | Mus musculus kinesin family member 3b (Kif3b), mRNA | 0.80 | 0,0443 |
| A\_51\_P315941 | AK016167 | Mus musculus RIKEN cDNA 4930557O20 gene (4930557O20Rik), mRNA | 0.80 | 0,0356 |
| A\_51\_P385874 | NM\_011143 | Mouse Brn-3 gene POU-box region | 0.80 | 0,0104 |
| A\_51\_P343497 | XM\_129647 | "BIFUNCTIONAL AMINOACYL-TRNA SYNTHETASE [INCLUDES: GLUTAMYL-TRNA SYNTHETASE (EC 6.1.1.17) (GLUTAMATE--TRNA LIGASE)� PROLYL-TRNA SYNTHETASE (EC 6.1.1.15) (PROLINE--TRNA LIGASE)], homolog [Homo sapiens]" | 0.80 | 0,0246 |
| A\_51\_P219109 | NM\_008353 | Mus musculus interleukin 12 receptor, beta 1 (Il12rb1), mRNA | 0.80 | 0,0447 |
| A\_51\_P496741 | NM\_019459 | Mus musculus nephrosis 1 homolog, nephrin (human) (Nphs1), mRNA | 0.80 | 0,0328 |
| A\_51\_P305239 | AK016429 | hypothetical protein | 0.80 | 0,0392 |
| A\_51\_P332632 | NM\_172871 | hypothetical Kelch repeat containing protein | 0.80 | 0,0458 |
| A\_51\_P386189 | NM\_016788 | Mus musculus tyrosine kinase, non-receptor, 2 (Tnk2), mRNA | 0.80 | 0,0325 |
| A\_51\_P370082 | NM\_011549 | Mus musculus transcription factor EB (Tcfeb), mRNA | 0.80 | 0,0282 |
| A\_51\_P404275 | NM\_007410 | Mus musculus alcohol dehydrogenase 5 (Adh5), mRNA | 0.80 | 0,0481 |
| A\_51\_P510096 | NM\_172593 | MESODERM INDUCTION EARLY RESPONSE 1 | 0.80 | 0,0355 |
| A\_51\_P410576 | NM\_172914 | hypothetical protein | 0.79 | 0,0440 |
| A\_51\_P454286 | XM\_198225 | MYOSIN VC (MYOSIN 5C) homolog [Homo sapiens] | 0.79 | 0,0420 |
| A\_51\_P477716 | NM\_153112 | Mus musculus membrane glycoprotein (Tsll2) mRNA, complete cds | 0.79 | 0,0295 |
| A\_51\_P507290 | NM\_009769 | Mus musculus Kruppel-like factor 5 (Klf5), mRNA | 0.79 | 0,0372 |
| A\_51\_P256224 | NM\_146202 | similar to ZINC FINGER PROTEIN 11 (FRAGMENT) [Rattus norvegicus] | 0.79 | 0,0424 |
| A\_51\_P217465 | NM\_183148 | Mus musculus, similar to FLJ00074 protein, clone MGC:36549 IMAGE:4952810, mRNA, complete cds | 0.79 | 0,0294 |
| A\_51\_P455009 | AK220465 | similar to SPERM ANTIGEN [Homo sapiens] | 0.79 | 0,0216 |
| A\_51\_P223412 | NM\_015733 | Mus musculus caspase 9 (Casp9), mRNA | 0.79 | 0,0306 |
| A\_51\_P246693 | NM\_019834 | Mus musculus G protein-coupled receptor kinase-interactor 2 (Git2), mRNA | 0.79 | 0,0338 |
| A\_51\_P343369 | AK020843 | unknown EST | 0.79 | 0,0404 |
| A\_51\_P177897 | NM\_011665 | Mus musculus cDNA clone IMAGE:336206 | 0.79 | 0,0352 |
| A\_51\_P293753 | NM\_025611 | Mus musculus RIKEN cDNA 2510004L20 gene (2510004L20Rik), mRNA | 0.79 | 0,0248 |
| A\_51\_P422839 | NM\_133888 | Mus musculus RIKEN cDNA 1110054A24 gene (1110054A24Rik), mRNA | 0.79 | 0,0423 |
| A\_51\_P215849 | NM\_019508 | Mus musculus interleukin 17B (Il17b), mRNA | 0.79 | 0,0419 |
| A\_51\_P406306 | NM\_172655 | hypothetical C2 domain/C2-domain profile containing protein | 0.79 | 0,0247 |
| A\_51\_P137042 | NM\_175033 | weakly similar to SELENOPROTEIN W [Mus musculus] | 0.79 | 0,0132 |
| A\_51\_P386798 | AK006064 | hypothetical protein | 0.79 | 0,0140 |
| A\_51\_P247347 | A\_51\_P247347 | Mus musculus cDNA, 3 end | 0.79 | 0,0248 |
| A\_51\_P369461 | ENSMUST00000080904 | BUTYROPHILIN-LIKE (FRAGMENT) | 0.79 | 0,0423 |
| A\_51\_P253834 | NM\_013603 | Mus musculus metallothionein 3 (Mt3), mRNA | 0.79 | 0,0104 |
| A\_51\_P480858 | NM\_021453 | Mus musculus pepsinogen F (Pepf-pending), mRNA | 0.78 | 0,0276 |
| A\_51\_P493570 | NM\_146505 | Mus musculus olfactory receptor MOR224-1 (MOR224-1), mRNA | 0.78 | 0,0427 |
| A\_51\_P388857 | NM\_009395 | Mus musculus tumor necrosis factor, alpha-induced protein 1 (endothelial) (Tnfaip1), mRNA | 0.78 | 0,0339 |
| A\_51\_P172054 | NM\_019521 | Mus musculus growth arrest specific 6 (Gas6), mRNA | 0.78 | 0,0453 |
| A\_51\_P204121 | AK086019 | weakly similar to NUCLEOSIDE DIPHOSPHATE-LINKED MOIETY X MOTIF 6 (PROTEIN GFG) [Rattus norvegicus] | 0.78 | 0,0487 |
| A\_51\_P391585 | A\_51\_P391585 | Mus musculus carnitine O-octanoyltransferase (Crot), mRNA | 0.78 | 0,0261 |
| A\_51\_P506284 | NM\_026131 | Mus musculus RIKEN cDNA 1110003B01 gene (1110003B01Rik), mRNA | 0.78 | 0,0439 |
| A\_51\_P238094 | NM\_024444 | Mus musculus RIKEN cDNA 1810054N16 gene (1810054N16Rik), mRNA | 0.78 | 0,0187 |
| A\_51\_P483499 | NM\_011453 | Mus musculus serine protease inhibitor 11 (Spi11), mRNA | 0.78 | 0,0426 |
| A\_51\_P413147 | NM\_008693 | Mouse mRNA for gamma-7S nerve growth factor (y-NGF) fragment | 0.78 | 0,0242 |
| A\_51\_P443686 | NM\_010319 | Mus musculus oocyte G protein gamma 7 subunit mRNA, partial cds | 0.78 | 0,0308 |
| A\_51\_P334331 | NM\_027397 | Mus musculus RIKEN cDNA 3110001N10 gene (3110001N10Rik), mRNA | 0.78 | 0,0201 |
| A\_51\_P175758 | NM\_026566 | Mus musculus, RIKEN cDNA 9430023L20 gene, clone MGC:18665 IMAGE:4166443, mRNA, complete cds | 0.78 | 0,0369 |
| A\_51\_P473438 | XM\_620278 | hypothetical protein | 0.78 | 0,0271 |
| A\_51\_P441970 | NM\_175162 | hypothetical protein | 0.78 | 0,0384 |
| A\_51\_P156866 | NM\_022024 | Mus musculus glia maturation factor, gamma (Gmfg), mRNA | 0.78 | 0,0372 |
| A\_51\_P430202 | NM\_175380 | similar to GLYCEROL-3-PHOSPHATE DEHYDROGENASE [NAD+], CYTOPLASMIC (EC 1.1.1.8) (GPD-C) (GPDH-C) [Fugu rubripes] | 0.78 | 0,0386 |
| A\_51\_P470475 | AK016931 | hypothetical P-loop containing nucleotide triphosphate hydrolases structure containing protein | 0.78 | 0,0494 |
| A\_51\_P338027 | NM\_009961 | Mus musculus protocadherin alpha 10 (Pcdha10), mRNA | 0.78 | 0,0382 |
| A\_51\_P347154 | NM\_010084 | Mus musculus a disintegrin and metalloprotease domain 18 (Adam18), mRNA | 0.78 | 0,0310 |
| A\_51\_P390937 | BF579422 | IG KAPPA CHAIN V-V REGION L6 PRECURSOR (FRAGMENT) | 0.78 | 0,0142 |
| A\_51\_P381015 | NM\_007966 | Mus musculus even skipped homeotic gene 1 homolog (Evx1), mRNA | 0.78 | 0,0443 |
| A\_51\_P414606 | NM\_007698 | Mus musculus cholinergic receptor, muscarinic 1, CNS (Chrm1), mRNA | 0.78 | 0,0120 |
| A\_51\_P387868 | AK005842 | weakly similar to TEKTIN A1 [Strongylocentrotus purpuratus] | 0.78 | 0,0178 |
| A\_51\_P375670 | NM\_009326 | Mus musculus transcription elongation factor A (SII), 2 (Tcea2), mRNA | 0.78 | 0,0224 |
| A\_51\_P123604 | NM\_172807 | weakly similar to LD39850P [Drosophila melanogaster] | 0.78 | 0,0239 |
| A\_51\_P275930 | NM\_194355 | Mus musculus RIKEN cDNA 6030430B19 gene, mRNA (cDNA clone MGC:51665 IMAGE:5318015), complete cds | 0.78 | 0,0238 |
| A\_51\_P183746 | NM\_009116 | Mus musculus paired related homeobox 2 (Prrx2), mRNA | 0.78 | 0,0212 |
| A\_51\_P477419 | NM\_026756 | NUCLEAR FACTOR 1 C-TYPE (NUCLEAR FACTOR 1/C) (NF1-C) (NFI-C) (NF-I/C) (CCAAT-BOX BINDING TRANSCRIPTION FACTOR) (CTF) (TGGCA-BINDING PROTEIN) | 0.78 | 0,0197 |
| A\_51\_P454207 | NM\_008679 | Mus musculus nuclear receptor coactivator 3 (Ncoa3), mRNA | 0.78 | 0,0247 |
| A\_51\_P182116 | NM\_019466 | Mus musculus Down syndrome critical region homolog 1 (human) (Dscr1), mRNA | 0.78 | 0,0329 |
| A\_51\_P269045 | A\_51\_P269045 | Mus musculus RIKEN cDNA 4930517G15 gene (4930517G15Rik), mRNA | 0.78 | 0,0294 |
| A\_51\_P438399 | NM\_010049 | unknown EST | 0.78 | 0,0111 |
| A\_51\_P460143 | NM\_015750 | Mus musculus neuraminidase 2 (Neu2), mRNA | 0.78 | 0,0343 |
| A\_51\_P334563 | AK013746 | unclassifiable | 0.78 | 0,0203 |
| A\_51\_P497768 | XM\_130488 | hypothetical protein | 0.78 | 0,0357 |
| A\_51\_P222733 | NM\_010381 | Mus musculus histocompatibility 2, class II antigen E alpha (H2-Ea), mRNA | 0.78 | 0,0168 |
| A\_51\_P410853 | NM\_025949 | RIBOSOMAL PROTEIN S6 KINASE ALPHA 6 (EC 2.7.1.-) (S6K-ALPHA 6) (90 KDA RIBOSOMAL PROTEIN S6 KINASE 6) (P90-RSK 6) (RIBOSOMAL S6 KINASE 4) (RSK-4) (PP90RSK4) homolog [Homo sapiens] | 0.78 | 0,0311 |
| A\_51\_P437707 | NM\_019815 | Mus musculus claudin 18 (Cldn18), mRNA | 0.78 | 0,0202 |
| A\_51\_P284716 | AK029593 | weakly similar to CDNA: FLJ23584 FIS, CLONE LNG14307 (HYPOTHETICAL 25.7 KDA PROTEIN) [Homo sapiens] | 0.78 | 0,0324 |
| A\_51\_P244856 | NM\_007504 | SARCOPLASMIC/ENDOPLASMIC RETICULUM CALCIUM ATPASE 2 EC 3.6.3.8 CALCIUM PUMP 2 SERCA2 SR CA 2+ ATPASE 2 CALCIUM TRANSPORTING ATPASE SARCOPLASMIC RETICULUM TYPE, SLOW TWITCH SKELETAL MUSCLE ISOFORM ENDOPLASMIC RETICULUM CLASS 1/2 CA 2+ | 0.78 | 0,0128 |
| A\_51\_P307427 | XM\_135033 | Mus musculus, Similar to KIAA1009 protein, clone IMAGE:1332852, mRNA | 0.78 | 0,0379 |
| A\_51\_P162553 | AK018093 | hypothetical Pleckstrin putative G-protein interacting domain containing protein | 0.78 | 0,0224 |
| A\_51\_P241933 | NM\_013921 | Mus musculus distal intestinal serine protease (Disp-pending), mRNA | 0.77 | 0,0494 |
| A\_51\_P237418 | AK014752 | hypothetical protein | 0.77 | 0,0263 |
| A\_51\_P345867 | NM\_011939 | Mus musculus heat shock transcription factor 4 (Hsf4), mRNA | 0.77 | 0,0346 |
| A\_51\_P372050 | AK014229 | hypothetical protein | 0.77 | 0,0459 |
| A\_51\_P484880 | NM\_207680 | Mus musculus BCL2-like 11 (apoptosis facilitator) (Bcl2l11), mRNA | 0.77 | 0,0106 |
| A\_51\_P250191 | NM\_144841 | M.musculus Otx2 mRNA | 0.77 | 0,0458 |
| A\_51\_P327141 | NM\_009539 | Mus musculus zeta-chain (TCR) associated protein kinase (70kD) (Zap70), mRNA | 0.77 | 0,0145 |
| A\_51\_P143212 | AK048650 | SMC4 PROTEIN homolog [Microtus arvalis] | 0.77 | 0,0418 |
| A\_51\_P279062 | NM\_027763 | hypothetical Immunoglobulin subtype containing protein | 0.77 | 0,0150 |
| A\_51\_P107807 | NM\_147111 | Mus musculus olfactory receptor GA\_x5J8B7W5P47-202028-201789 (GA\_x5J8B7W5P47-202028-201789) pseudogene | 0.77 | 0,0190 |
| A\_51\_P205779 | NM\_009690 | Mus musculus apoptosis inhibitory 6 (Api6), mRNA | 0.77 | 0,0075 |
| A\_51\_P381060 | NM\_133209 | Mus musculus immunoglobulin-like cell surface receptor FDFACT, activating counterpart (FDFACT), mRNA | 0.77 | 0,0335 |
| A\_51\_P301499 | AK007620 | weakly similar to NADH-UBIQUINONE OXIDOREDUCTASE 13 KDA-A SUBUNIT, MITOCHONDRIAL PRECURSOR (EC 1.6.5.3) (EC 1.6.99.3) (COMPLEX I-13KD-A) (CI-13KD-A) [Homo sapiens] | 0.77 | 0,0181 |
| A\_51\_P513741 | AK173242 | Mus musculus cDNA clone IMAGE:6335382 5 | 0.77 | 0,0153 |
| A\_51\_P240703 | NM\_181543 | hypothetical Rhodopsin-like GPCR superfamily containing protein | 0.77 | 0,0484 |
| A\_51\_P467483 | NM\_173443 | Mus musculus, clone IMAGE:3591498, mRNA | 0.77 | 0,0478 |
| A\_51\_P255395 | NM\_007738 | Mus musculus procollagen, type VII, alpha 1 (Col7a1), mRNA | 0.77 | 0,0210 |
| A\_51\_P199318 | AK047403 | unknown EST | 0.77 | 0,0194 |
| A\_51\_P325664 | NM\_146927 | Mus musculus olfactory receptor MOR114-2 (MOR114-2), mRNA | 0.77 | 0,0224 |
| A\_51\_P377528 | NM\_011011 | Mouse kappa opioid receptor mRNA, complete cds | 0.77 | 0,0251 |
| A\_51\_P236588 | AK078159 | hypothetical protein | 0.77 | 0,0342 |
| A\_51\_P128248 | NM\_198640 | Mus musculus Ig rearranged anti-Sm hybridoma mRNA V-region sequence | 0.77 | 0,0450 |
| A\_51\_P146407 | NM\_138598 | Mus musculus DNA segment, Chr 11, Wayne State University 99, expressed (D11Wsu99e), mRNA | 0.77 | 0,0350 |
| A\_51\_P225232 | NM\_029727 | similar to GASDERMIN [Mus musculus] | 0.77 | 0,0291 |
| A\_51\_P442759 | NM\_172520 | weakly similar to RHO GUANINE NUCLEOTIDE EXCHANGE FACTOR 5 (GUANINE NUCLEOTIDE REGULATORY PROTEIN TIM) (ONCOGENE TIM) (P60 TIM) (TRANSFORMING IMMORTALIZED MAMMARY ONCOGENE) [Homo sapiens] | 0.77 | 0,0455 |
| A\_51\_P390239 | NM\_032396 | Mus musculus kringle containing transmembrane protein (Kremen), mRNA | 0.77 | 0,0096 |
| A\_51\_P444162 | NM\_144939 | Mus musculus, Similar to suc1-associated neurotrophic factor target 2 (FGFR signalling adaptor), clone MGC:25496 IMAGE:4506982, mRNA, complete cds | 0.77 | 0,0187 |
| A\_51\_P359891 | NM\_011426 | Mus musculus sialoadhesin (Sn), mRNA | 0.77 | 0,0251 |
| A\_51\_P410205 | NM\_008289 | Mus musculus hydroxysteroid 11-beta dehydrogenase 2 (Hsd11b2), mRNA | 0.77 | 0,0193 |
| A\_51\_P369185 | NM\_028625 | Mus musculus small proline rich-like 2 (Sprrl2), mRNA | 0.77 | 0,0369 |
| A\_51\_P433157 | XM\_284439 | similar to DJ620E11.1.1 (NOVEL HELICASE C-TERMINAL DOMAIN AND SNF2 N-TERMINAL DOMAINS CONTAINING PROTEIN, SIMILAR TO KIAA0308 (ISOFORM 1)) (FRAGMENT) [Homo sapiens] | 0.77 | 0,0411 |
| A\_51\_P162474 | AF285577 | Mus musculus sex comb on midleg-like 2 (Drosophila) (Scml2), mRNA | 0.77 | 0,0291 |
| A\_51\_P132620 | NM\_053262 | Mus musculus hydroxysteroid 17-beta dehydrogenase 11 (Hsd17b11), mRNA | 0.77 | 0,0298 |
| A\_51\_P268154 | NM\_019477 | Mus musculus fatty acid-Coenzyme A ligase, long chain 4 (Facl4), mRNA | 0.77 | 0,0243 |
| A\_51\_P278721 | NM\_053069 | Mus musculus autophagy 5-like (S. cerevisiae) (Apg5l), mRNA | 0.77 | 0,0296 |
| A\_51\_P362348 | NM\_020280 | Mus musculus melanoma antigen, family A, 4 (Magea4), mRNA | 0.77 | 0,0093 |
| A\_51\_P219025 | NM\_026898 | hypothetical Trp-Asp (WD) repeats profile/Trp-Asp (WD) repeats circular profile/G-protein beta WD-40 repeats containing protein | 0.77 | 0,0183 |
| A\_51\_P508469 | NM\_146833 | Mus musculus olfactory receptor MOR250-3 (MOR250-3), mRNA | 0.77 | 0,0397 |
| A\_51\_P125265 | NM\_008091 | Mus musculus GATA binding protein 3 (Gata3), mRNA | 0.77 | 0,0283 |
| A\_51\_P156962 | NM\_026435 | Mus musculus RIKEN cDNA 1810045K17 gene (1810045K17Rik), mRNA | 0.77 | 0,0332 |
| A\_51\_P324871 | NM\_201351 | unknown | 0.77 | 0,0254 |
| A\_51\_P462385 | NM\_008061 | Mus musculus glucose-6-phosphatase, catalytic (G6pc), mRNA | 0.77 | 0,0259 |
| A\_51\_P152670 | AF357512 | Mus musculus clone MBII-223 miscellaneous RNA, partial sequence | 0.77 | 0,0141 |
| A\_51\_P483329 | NM\_172713 | weakly similar to MYSTERY 45A [Drosophila melanogaster] | 0.77 | 0,0242 |
| A\_51\_P295210 | NM\_178309 | similar to BRCA1-BINDING HELICASE-LIKE PROTEIN BACH1 [Homo sapiens] | 0.77 | 0,0145 |
| A\_51\_P520066 | NM\_020273 | Mus musculus glucocorticoid modulatory element binding protein 1 (Gmeb1), mRNA | 0.77 | 0,0136 |
| A\_51\_P245393 | NM\_145993 | H-L(3)MBT-LIKE PROTEIN (HYPOTHETICAL 79.1 KDA PROTEIN) homolog [Homo sapiens] | 0.77 | 0,0234 |
| A\_51\_P381916 | AK042951 | unknown EST | 0.77 | 0,0459 |
| A\_51\_P332717 | NM\_011673 | Mus musculus UDP-glucose ceramide glucosyltransferase (Ugcg), mRNA | 0.77 | 0,0108 |
| A\_51\_P309488 | AK007900 | hypothetical protein | 0.77 | 0,0087 |
| A\_51\_P231113 | NM\_147100 | OLFACTORY RECEPTOR MOR20-1 | 0.77 | 0,0266 |
| A\_51\_P331827 | NM\_175333 | weakly similar to CDNA FLJ30339 FIS, CLONE BRACE2007401, MODERATELY SIMILAR TO ORYCTOLAGUS CUNICULUS PEROXISOMAL CA-DEPENDENT SOLUTE CARRIER MRNA [Homo sapiens] | 0.77 | 0,0331 |
| A\_51\_P515412 | NAP002111-001 | ZINC FINGER | 0.77 | 0,0441 |
| A\_51\_P302336 | NM\_023670 | Mus musculus insulin-like growth factor 2, binding protein 3 (Igf2bp3), mRNA | 0.77 | 0,0398 |
| A\_51\_P263033 | NM\_010739 | Mus musculus lymphocyte antigen 64 (Ly64), mRNA | 0.76 | 0,0135 |
| A\_51\_P189497 | NM\_009225 | Mus musculus small nuclear ribonucleoprotein B (Snrpb), mRNA | 0.76 | 0,0309 |
| A\_51\_P152841 | NM\_153557 | hypothetical protein | 0.76 | 0,0045 |
| A\_51\_P284937 | NM\_138591 | Mus musculus G elongation factor (Gfm), mRNA | 0.76 | 0,0179 |
| A\_51\_P310949 | NM\_008911 | Mus musculus protoporphyrinogen oxidase (Ppox), mRNA | 0.76 | 0,0045 |
| A\_51\_P174264 | NM\_198614 | hypothetical C2 domain containing protein | 0.76 | 0,0194 |
| A\_51\_P489367 | NM\_019792 | M.musculus mRNA for cytochrome P450IIIA25 | 0.76 | 0,0253 |
| A\_51\_P346608 | A\_51\_P346608 | Mus musculus meiosis-specific nuclear structural protein 1 (Mns1), mRNA | 0.76 | 0,0203 |
| A\_51\_P115346 | NM\_011028 | Mus musculus purinergic receptor P2X-like 1, orphan receptor (P2rxl1), mRNA | 0.76 | 0,0170 |
| A\_51\_P333891 | NM\_146471 | OLFACTORY RECEPTOR | 0.76 | 0,0353 |
| A\_51\_P391716 | NM\_013848 | Mus musculus erythroblast membrane-associated protein (Ermap), mRNA | 0.76 | 0,0059 |
| A\_51\_P412391 | NM\_011998 | Mus musculus carbohydrate (chondroitin 6/keratan) sulfotransferase 4 (Chst4), mRNA | 0.76 | 0,0142 |
| A\_51\_P392593 | AK049893 | X-LIKE 1 PROTEIN homolog [Homo sapiens] | 0.76 | 0,0312 |
| A\_51\_P233002 | NM\_007859 | Mus musculus DNA fragmentation factor, 40 kD, beta subunit (Dffb), mRNA | 0.76 | 0,0206 |
| A\_51\_P326417 | NM\_019712 | Mus musculus ring-box 1 (Rbx1), mRNA | 0.76 | 0,0169 |
| A\_51\_P199593 | XM\_131409 | unknown EST | 0.76 | 0,0410 |
| A\_51\_P406835 | AY270177 | hypothetical ARM repeat structure containing protein | 0.76 | 0,0376 |
| A\_51\_P182464 | NM\_010688 | Mus musculus LIM and SH3 protein 1 (Lasp1), mRNA | 0.76 | 0,0319 |
| A\_51\_P287241 | AK005888 | hypothetical protein | 0.76 | 0,0360 |
| A\_51\_P245405 | NM\_008915 | Mus musculus protein phosphatase 3, catalytic subunit, gamma isoform (Ppp3cc), mRNA | 0.76 | 0,0407 |
| A\_51\_P277336 | NM\_138741 | Mus musculus serum deprivation response (Sdpr), mRNA | 0.76 | 0,0225 |
| A\_51\_P496273 | NM\_023502 | Mus musculus RIKEN cDNA 2610036A20 gene (2610036A20Rik), mRNA | 0.76 | 0,0198 |
| A\_51\_P256902 | NM\_031884 | Mus musculus ATP-binding cassette, sub-family G (WHITE), member 5 (Abcg5), mRNA | 0.76 | 0,0197 |
| A\_51\_P272123 | NM\_024197 | Mus musculus RIKEN cDNA 2900053E13 gene (2900053E13Rik), mRNA | 0.76 | 0,0288 |
| A\_51\_P450505 | NM\_001012266 | Mus musculus olfactory receptor MOR150-3 (MOR150-3) pseudogene | 0.76 | 0,0365 |
| A\_51\_P178825 | NM\_010776 | Mus musculus mannose binding lectin, serum (C) (Mbl2), mRNA | 0.76 | 0,0460 |
| A\_51\_P256549 | AK015138 | similar to DJ1068E13.2 (NOVEL PROTEIN SIMILAR TO BOVINE SCP2 (STEROL CARRIER PROTEIN 2) AND PART OF HSD17B4 (HYDROXYSTEROID (17-BETA) DEHYDROGENASE 4)) [Homo sapiens] | 0.76 | 0,0247 |
| A\_51\_P465449 | NM\_008653 | Mus musculus myosin binding protein C, cardiac (Mybpc3), mRNA | 0.76 | 0,0066 |
| A\_51\_P380013 | NM\_177410 | Mus musculus B-cell leukemia/lymphoma 2 (Bcl2), mRNA | 0.76 | 0,0116 |
| A\_51\_P293968 | NM\_001003918 | Mus musculus cDNA, 5 end | 0.76 | 0,0372 |
| A\_51\_P502608 | NM\_133784 | Mus musculus RIKEN cDNA 2310058J06 gene (2310058J06Rik), mRNA | 0.76 | 0,0112 |
| A\_51\_P139751 | U26472 | Mus musculus nucleosome-reactive monoclonal antibody MGC23, Ig light chain variable region mRNA, partial cds | 0.76 | 0,0162 |
| A\_51\_P251588 | NM\_026000 | Mus musculus proteasome (prosome, macropain) 26S subunit, non-ATPase, 9 (Psmd9), mRNA | 0.76 | 0,0472 |
| A\_51\_P464600 | NM\_182840 | Mus musculus, clone IMAGE:3485728, mRNA | 0.76 | 0,0050 |
| A\_51\_P464490 | A\_51\_P464490 | Mus musculus cDNA, 5 end | 0.76 | 0,0483 |
| A\_51\_P273096 | AK042259 | inferred: putative {Mus musculus} | 0.76 | 0,0237 |
| A\_51\_P186510 | NM\_008759 | Mus musculus OG9 homeobox gene (Og9x), mRNA | 0.76 | 0,0283 |
| A\_51\_P434759 | BC042793 | hypothetical protein | 0.76 | 0,0441 |
| A\_51\_P211088 | AK037397 | unclassifiable | 0.76 | 0,0499 |
| A\_51\_P454736 | NM\_009612 | Mus musculus activin A receptor, type II-like 1 (Acvrl1), mRNA | 0.76 | 0,0311 |
| A\_51\_P129435 | AK007269 | hypothetical AT-rich interaction domain (ARID) containing protein | 0.76 | 0,0441 |
| A\_51\_P353703 | NM\_010237 | Mus musculus B-cell src-homology tyrosine kinase (Frk), mRNA | 0.76 | 0,0072 |
| A\_51\_P137640 | AK011315 | RIKEN cDNA 2610005D18 gene | 0.76 | 0,0440 |
| A\_51\_P246345 | NM\_022879 | Mus musculus myosin light chain, regulatory A (Mylc2a), mRNA | 0.76 | 0,0371 |
| A\_51\_P471219 | NM\_175486 | similar to G20 PROTEIN [Homo sapiens] | 0.76 | 0,0087 |
| A\_51\_P133576 | BC036332 | hypothetical Adenine nucleotide alpha hydrolases structure containing protein | 0.76 | 0,0350 |
| A\_51\_P410312 | XM\_619801 | Mus musculus mRNA for motor domain of KIF16A, partial cds | 0.76 | 0,0306 |
| A\_51\_P324535 | NM\_019835 | Mus musculus UDP-Gal:betaGlcNAc beta 1,4-galactosyltransferase, polypeptide 5 (B4galt5), mRNA | 0.76 | 0,0238 |
| A\_51\_P223489 | AK040351 | unclassifiable | 0.76 | 0,0037 |
| A\_51\_P469401 | NM\_009400 | Mus musculus tumor necrosis factor receptor superfamily, member 18 (Tnfrsf18), mRNA | 0.76 | 0,0079 |
| A\_51\_P135379 | NM\_025703 | Mus musculus RIKEN cDNA 3930402F23 gene (3930402F23Rik), mRNA | 0.76 | 0,0362 |
| A\_51\_P229480 | AK013604 | unknown | 0.76 | 0,0247 |
| A\_51\_P501622 | NM\_026628 | Mus musculus RIKEN cDNA 1700120K04 gene (1700120K04Rik), mRNA | 0.76 | 0,0239 |
| A\_51\_P285020 | AK054516 | hypothetical protein | 0.76 | 0,0167 |
| A\_51\_P259773 | NM\_020258 | Mus musculus solute carrier family 37 (glycerol-3-phosphate transporter), member 1 (Slc37a1), mRNA | 0.76 | 0,0167 |
| A\_51\_P292168 | NM\_027698 | Mus musculus RIKEN cDNA 4933424N09 gene (4933424N09Rik), mRNA | 0.76 | 0,0243 |
| A\_51\_P234893 | NM\_021306 | Mus musculus endothelin converting enzyme-like 1 (Ecel1), mRNA | 0.76 | 0,0299 |
| A\_51\_P378387 | XM\_355790 | weakly similar to RSEC15 [Rattus norvegicus] | 0.76 | 0,0202 |
| A\_51\_P159792 | NM\_009928 | Mus musculus procollagen, type XV (Col15a1), mRNA | 0.76 | 0,0079 |
| A\_51\_P357313 | NM\_023055 | Mus musculus solute carrier family 9 (sodium/hydrogen exchanger), isoform 3 regulator 2 (Slc9a3r2), mRNA | 0.75 | 0,0369 |
| A\_51\_P136028 | AK046838 | unclassifiable | 0.75 | 0,0157 |
| A\_51\_P386983 | NM\_009406 | Mus musculus troponin I, cardiac (Tnni3), mRNA | 0.75 | 0,0153 |
| A\_51\_P380136 | AK018651 | syntrophin associated serine/threonine kinase | 0.75 | 0,0239 |
| A\_51\_P375187 | NM\_023323 | Mus musculus RIKEN cDNA 2810470K21 gene (2810470K21Rik), mRNA | 0.75 | 0,0371 |
| A\_51\_P200223 | XM\_619497 | hypothetical protein | 0.75 | 0,0154 |
| A\_51\_P390628 | NM\_146247 | Mus musculus, clone MGC:38788 IMAGE:5359405, mRNA, complete cds | 0.75 | 0,0228 |
| A\_51\_P328060 | NM\_019929 | Mus musculus SMT3 (supressor of mif two, 3) homolog 1 (S. cerevisiae) (Smt3h1), mRNA | 0.75 | 0,0176 |
| A\_51\_P354548 | AK013182 | hypothetical protein | 0.75 | 0,0314 |
| A\_51\_P205129 | AB030188 | Mus musculus hypothetical protein, clone 1-53 (AB030188), mRNA | 0.75 | 0,0239 |
| A\_51\_P278675 | NM\_009643 | M.musculus mRNA for desmoyokin, partial | 0.75 | 0,0080 |
| A\_51\_P430014 | AK032547 | PLATELET ACTIVATING FACTOR RECEPTOR | 0.75 | 0,0397 |
| A\_51\_P216679 | NM\_011922 | annexin A10 | 0.75 | 0,0079 |
| A\_51\_P294328 | NM\_172727 | hypothetical protein | 0.75 | 0,0445 |
| A\_51\_P273863 | NM\_172919 | similar to MSZF23-1 (FRAGMENT) [Mus musculus] | 0.75 | 0,0162 |
| A\_51\_P136269 | NM\_027874 | Mus musculus casein kinase 1, delta (Csnk1d), transcript variant 1, mRNA | 0.75 | 0,0043 |
| A\_51\_P375285 | AK083796 | weakly similar to ENVELOPE PROTEIN (FRAGMENT) [Friend spleen focus-forming virus] | 0.75 | 0,0113 |
| A\_51\_P475689 | NM\_026972 | Mus musculus CD209b antigen (Cd209b-pending), mRNA | 0.75 | 0,0330 |
| A\_51\_P339803 | NM\_146227 | Mus musculus TSP50 (Tsp50), mRNA | 0.75 | 0,0167 |
| A\_51\_P332547 | AK017723 | Mus musculus RIKEN cDNA 5730493B19 gene (5730493B19Rik), mRNA | 0.75 | 0,0261 |
| A\_51\_P311866 | AK031314 | hypothetical Reprolysin family propeptide containing protein | 0.75 | 0,0169 |
| A\_51\_P339331 | AK018683 | HOMEOBOX PROTEIN NKX 6 | 0.75 | 0,0295 |
| A\_51\_P119358 | AK005633 | hypothetical Apoptosis regulator protein, Bcl-2 family BH domain containing protein | 0.75 | 0,0083 |
| A\_51\_P204831 | NM\_007763 | Mus musculus cysteine rich intestinal protein (Crip), mRNA | 0.75 | 0,0495 |
| A\_51\_P473051 | AK089344 | arachidonate 5-lipoxygenase | 0.75 | 0,0117 |
| A\_51\_P291741 | NM\_021337 | Mus musculus superkiller viralicidic activity 2-like (S. cerevisiae ) (Skiv2l), mRNA | 0.75 | 0,0129 |
| A\_51\_P260051 | NM\_007476 | Mus musculus ADP-ribosylation factor 1 (Arf1), mRNA | 0.75 | 0,0124 |
| A\_51\_P147906 | AK014870 | RIKEN cDNA 4921511H03 gene | 0.75 | 0,0241 |
| A\_51\_P166198 | BC004575 | Mus musculus, Similar to TEMO, clone IMAGE:3586444, mRNA, partial cds | 0.75 | 0,0103 |
| A\_51\_P418913 | BC053449 | unknown EST | 0.75 | 0,0216 |
| A\_51\_P151983 | NM\_019744 | Mus musculus nuclear receptor coactivator 4 (Ncoa4), mRNA | 0.75 | 0,0206 |
| A\_51\_P323011 | AK045005 | hypothetical CBL proto-oncogene N-terminal domain containing protein | 0.75 | 0,0440 |
| A\_51\_P163639 | NM\_027055 | hypothetical protein | 0.75 | 0,0231 |
| A\_51\_P304490 | NM\_206896 | Mus musculus olfactory receptor MOR208-5 (MOR208-5) pseudogene | 0.75 | 0,0389 |
| A\_51\_P163222 | NM\_133752 | Mus musculus optic atrophy 1 homolog (human) (Opa1), mRNA | 0.75 | 0,0230 |
| A\_51\_P170675 | NM\_011386 | Mus musculus ski/sno related (Skir), mRNA | 0.75 | 0,0128 |
| A\_51\_P201450 | AK014425 | hypothetical Histidine-rich region containing protein | 0.75 | 0,0048 |
| A\_51\_P293110 | NM\_010818 | Mus musculus antigen identified by monoclonal antibody MRC OX-2 (Mox2), mRNA | 0.75 | 0,0336 |
| A\_51\_P190604 | NM\_134010 | Mus musculus, clone IMAGE:3497824, mRNA, partial cds | 0.75 | 0,0143 |
| A\_51\_P464539 | NM\_153412 | hypothetical Serine-rich region containing protein | 0.75 | 0,0309 |
| A\_51\_P381957 | AK006312 | protein phosphatase 2, regulatory subunit B (B56), gamma isoform | 0.75 | 0,0296 |
| A\_51\_P426902 | NM\_024470 | Mus musculus killer cell lectin-like receptor subfamily A, member 23 (Klra23), mRNA | 0.75 | 0,0494 |
| A\_51\_P507965 | NM\_029690 | similar to ACTIN RELATED PROTEIN [Homo sapiens] | 0.75 | 0,0093 |
| A\_51\_P231721 | NM\_011944 | Mus musculus mitogen activated protein kinase kinase 7 (Map2k7), mRNA | 0.75 | 0,0424 |
| A\_51\_P378682 | XM\_620428 | CEA15 PROTEIN (FRAGMENT) | 0.75 | 0,0232 |
| A\_51\_P243641 | BC032281 | Mus musculus, clone IMAGE:3989034, mRNA | 0.75 | 0,0143 |
| A\_51\_P487832 | XM\_354752 | DJ259A10.1 (SSDNA BINDING PROTEIN (SEB4D)) homolog [Homo sapiens] | 0.75 | 0,0120 |
| A\_51\_P420803 | NM\_011675 | Mus musculus uridine kinase mRNA, partial cds | 0.75 | 0,0266 |
| A\_51\_P167713 | BC068151 | WD-CONTAINING PROTEIN (FRAGMENT) homolog [Rattus norvegicus] | 0.75 | 0,0229 |
| A\_51\_P345416 | M64608 | Yamaguchi sarcoma viral (v-yes-1) oncogene homolog | 0.75 | 0,0088 |
| A\_51\_P378754 | NM\_017467 | Mus musculus zinc finger protein 316 (Zfp316), mRNA | 0.75 | 0,0196 |
| A\_51\_P150175 | BC025043 | Mus musculus ubiquitination factor E4A, UFD2 homolog (S. cerevisiae) (Ube4a), mRNA | 0.75 | 0,0485 |
| A\_51\_P239737 | NM\_011082 | Mus musculus polymeric immunoglobulin receptor (Pigr), mRNA | 0.75 | 0,0223 |
| A\_51\_P374437 | NM\_139154 | Mus musculus Rab40c, member RAS oncogene family (Rab40c), mRNA | 0.75 | 0,0391 |
| A\_51\_P438692 | NM\_016800 | Mus musculus vesicle transport through interaction with t-SNAREs 1b homolog (Vti1b-pending), mRNA | 0.75 | 0,0189 |
| A\_51\_P409153 | AK044431 | PUTATIVE FOUR REPEAT ION CHANNEL homolog [Rattus norvegicus] | 0.75 | 0,0058 |
| A\_51\_P274907 | AK029583 | Rab6-interacting protein 2 | 0.75 | 0,0142 |
| A\_51\_P513934 | NM\_144901 | Mus musculus, clone MGC:19174 IMAGE:4224466, mRNA, complete cds | 0.75 | 0,0248 |
| A\_51\_P297011 | NM\_028243 | similar to LYSOSOMAL PRO-X CARBOXYPEPTIDASE PRECURSOR (EC 3.4.16.2) (PROLYLCARBOXYPEPTIDASE) (PRCP) (PROLINE CARBOXYPEPTIDASE) (ANGIOTENSINASE C) (LYSOSOMAL CARBOXYPEPTIDASE C) [Homo sapiens] | 0.75 | 0,0203 |
| A\_51\_P346679 | XM\_138377 | DNA sequence of a peptide which has a inhibition activity of binding ICAM-1 and LFA-1 | 0.75 | 0,0091 |
| A\_51\_P349244 | AA688787 | Mus musculus cDNA, 5 end | 0.75 | 0,0448 |
| A\_51\_P327405 | NM\_029509 | Mus musculus RIKEN cDNA 5830443L24 gene (5830443L24Rik), mRNA | 0.75 | 0,0266 |
| A\_51\_P340936 | BC006909 | Mus musculus, clone MGC:6888 IMAGE:2651775, mRNA, complete cds | 0.75 | 0,0455 |
| A\_51\_P496045 | AK020592 | mitochondria located 1 homolog (human) | 0.75 | 0,0177 |
| A\_51\_P488739 | NM\_030701 | Mus musculus interferon-gamma inducible gene, Puma-g (Pumag-pending), mRNA | 0.75 | 0,0082 |
| A\_51\_P133078 | NM\_008942 | Mus musculus puromycin-sensitive aminopeptidase (Psa), mRNA | 0.75 | 0,0423 |
| A\_51\_P446978 | NM\_011801 | Mus musculus craniofacial development protein 1 (Cfdp), mRNA | 0.75 | 0,0177 |
| A\_51\_P308750 | NM\_025546 | hypothetical Ribosomal protein L1 containing protein | 0.75 | 0,0197 |
| A\_51\_P106011 | NM\_133947 | similar to NUCLEAR MITOTIC APPARATUS PROTEIN (FRAGMENT) [Homo sapiens] | 0.75 | 0,0176 |
| A\_51\_P464420 | AK014845 | hypothetical protein | 0.74 | 0,0074 |
| A\_51\_P245208 | NM\_010317 | Mus musculus guanine nucleotide binding protein (G protein), gamma 4 subunit (Gng4), mRNA | 0.74 | 0,0418 |
| A\_51\_P205035 | AK042573 | hypothetical protein | 0.74 | 0,0181 |
| A\_51\_P430903 | XM\_486298 | hypothetical protein | 0.74 | 0,0301 |
| A\_51\_P358256 | NM\_009803 | Mus musculus nuclear receptor subfamily 1, group I, member 3 (Nr1i3), mRNA | 0.74 | 0,0115 |
| A\_51\_P275915 | AY550908 | PROGESTIN INDUCED PROTEIN homolog [Homo sapiens] | 0.74 | 0,0409 |
| A\_51\_P179933 | TC1501700 | Mus musculus hypertension-related, calcium regulated gene (Hcarg-pending), mRNA | 0.74 | 0,0176 |
| A\_51\_P469261 | NAP057024-1 | Mus musculus thioredoxin peroxidase, pseudogene 2 (Tdpx-ps2) | 0.74 | 0,0444 |
| A\_51\_P218202 | NM\_144514 | Mouse Murr1 mRNA, exon | 0.74 | 0,0156 |
| A\_51\_P346243 | NM\_010326 | Mus musculus glycoprotein 1b, alpha polypeptide (Gp1ba), mRNA | 0.74 | 0,0409 |
| A\_51\_P445211 | NM\_021311 | Mus musculus piwi like homolog 1 (Drosophila) (Piwil1), mRNA | 0.74 | 0,0076 |
| A\_51\_P467889 | NM\_025400 | Mus musculus RIKEN cDNA 1110028N05 gene (1110028N05Rik), mRNA | 0.74 | 0,0063 |
| A\_51\_P329094 | BC087920 | Mus musculus RIKEN cDNA 4930550L24 gene (4930550L24Rik), mRNA | 0.74 | 0,0050 |
| A\_51\_P223124 | XM\_127381 | Mus musculus, clone IMAGE:1067377, mRNA | 0.74 | 0,0359 |
| A\_51\_P356467 | NM\_009903 | Mus musculus claudin 4 (Cldn4), mRNA | 0.74 | 0,0092 |
| A\_51\_P407074 | NM\_025982 | Mus musculus RIKEN cDNA 2700085A14 gene (2700085A14Rik), mRNA | 0.74 | 0,0237 |
| A\_51\_P231687 | NM\_011879 | IK cytokine | 0.74 | 0,0264 |
| A\_51\_P300038 | NM\_027570 | weakly similar to PUTATIVE D-LACTATE DEHYDROGENASE (CYTOCHROME) OXIDOREDUCTASE PROTEIN (EC 1.1.2.4) [Ralstonia solanacearum] | 0.74 | 0,0148 |
| A\_51\_P234245 | AK081677 | Mus musculus cDNA, 3 end | 0.74 | 0,0081 |
| A\_51\_P469531 | NM\_019652 | Mus musculus arsA (bacterial) arsenite transporter, ATP-binding, homolog 1 (Asna1), mRNA | 0.74 | 0,0044 |
| A\_51\_P471830 | NM\_153075 | Mus musculus putative ion channel protein CATSPER2 mRNA, complete cds | 0.74 | 0,0182 |
| A\_51\_P479570 | AI840556 | Mus musculus cDNA, 3 end | 0.74 | 0,0464 |
| A\_51\_P381801 | NM\_007480 | Mus musculus ADP-ribosylation factor 5 (Arf5), mRNA | 0.74 | 0,0311 |
| A\_51\_P273489 | AK029819 | weakly similar to CENTAURIN, BETA 1 [Homo sapiens] | 0.74 | 0,0188 |
| A\_51\_P217906 | NM\_007799 | Mus musculus cathepsin E (Ctse), mRNA | 0.74 | 0,0387 |
| A\_51\_P335151 | XM\_484161 | unknown | 0.74 | 0,0202 |
| A\_51\_P314691 | AF108215 | Mus musculus 5-AMP-activated protein kinase beta subunit mRNA, partial cds | 0.74 | 0,0163 |
| A\_51\_P428483 | NM\_181849 | fibrinogen, B beta polypeptide | 0.74 | 0,0096 |
| A\_51\_P146593 | NM\_146773 | Mus musculus olfactory receptor MOR204-6 (MOR204-6), mRNA | 0.74 | 0,0293 |
| A\_51\_P373043 | NM\_013898 | Mus musculus translocase of inner mitochondrial membrane 8 homolog a (yeast) (Timm8a), mRNA | 0.74 | 0,0312 |
| A\_51\_P259445 | AK007933 | hypothetical protein | 0.74 | 0,0244 |
| A\_51\_P131277 | NM\_172857 | hypothetical 3-5 exonuclease containing protein | 0.74 | 0,0097 |
| A\_51\_P179864 | NM\_016979 | Mus musculus, Similar to putative serine/threonine kinase, clone MGC:11638 IMAGE:3596013, mRNA, complete cds | 0.74 | 0,0356 |
| A\_51\_P119749 | BB096820 | CDNA FLJ12921 FIS, CLONE NT2RP2004600 homolog [Homo sapiens] | 0.74 | 0,0090 |
| A\_51\_P235835 | AK075797 | DNA segment, Chr 12, ERATO Doi 647, expressed | 0.74 | 0,0111 |
| A\_51\_P497484 | NM\_172923 | hypothetical Cation channels (non-ligand gated) containing protein | 0.74 | 0,0040 |
| A\_51\_P503757 | AK008094 | immunoglobulin lambda chain, variable 1 | 0.74 | 0,0224 |
| A\_51\_P130110 | NM\_130884 | Mus musculus isocitrate dehydrogenase 3 (NAD+) beta (Idh3b), mRNA | 0.74 | 0,0283 |
| A\_51\_P358812 | AK081685 | DNA segment, Chr 5, Brigham & Womens Genetics 0676 expressed | 0.74 | 0,0057 |
| A\_51\_P462271 | NM\_007424 | Mus musculus aggrecan 1 (Agc1), mRNA | 0.74 | 0,0161 |
| A\_51\_P209135 | NM\_206924 | Mus musculus mJTB mRNA, complete cds | 0.74 | 0,0058 |
| A\_51\_P472730 | BC048726 | hypothetical KRAB box containing protein | 0.74 | 0,0307 |
| A\_51\_P208793 | NM\_144938 | Mus musculus, Similar to complement component 1, s subcomponent, clone MGC:19094 IMAGE:4196654, mRNA, complete cds | 0.74 | 0,0329 |
| A\_51\_P371923 | NM\_011519 | Mus musculus syndecan 1 (Sdc1), mRNA | 0.74 | 0,0268 |
| A\_51\_P450518 | BC042740 | HYPOTHETICAL 39.4 KDA PROTEIN homolog [Homo sapiens] | 0.74 | 0,0111 |
| A\_51\_P312956 | AK034271 | similar to ORF4 PROTEIN [Rattus norvegicus] | 0.74 | 0,0363 |
| A\_51\_P414442 | XM\_132099 | hypothetical SAM domain (Sterile alpha motif)/Pleckstrin homology (PH) domain containing protein | 0.74 | 0,0083 |
| A\_51\_P464691 | NM\_028648 | hypothetical protein | 0.74 | 0,0362 |
| A\_51\_P185478 | A\_51\_P185478 | Mus musculus cDNA, 5 end | 0.74 | 0,0372 |
| A\_51\_P146208 | AI841821 | Mus musculus cDNA, 3 end | 0.74 | 0,0283 |
| A\_51\_P138648 | NM\_010562 | Mus musculus integrin linked kinase (Ilk), mRNA | 0.74 | 0,0104 |
| A\_51\_P426195 | NM\_008726 | Mus musculus natriuretic peptide precursor type B (Nppb), mRNA | 0.74 | 0,0408 |
| A\_51\_P365287 | NM\_177574 | hypothetical protein | 0.74 | 0,0402 |
| A\_51\_P312497 | NM\_009726 | Mus musculus ATPase, Cu++ transporting, alpha polypeptide (Atp7a), mRNA | 0.74 | 0,0097 |
| A\_51\_P475612 | NM\_029629 | similar to CGI-105 PROTEIN [Homo sapiens] | 0.74 | 0,0335 |
| A\_51\_P332822 | NM\_015751 | Mus musculus ATP-binding cassette, sub-family E (OABP), member 1 (Abce1), mRNA | 0.74 | 0,0371 |
| A\_51\_P192085 | NM\_178788 | deoxycytidylate deaminase homolog [Homo sapiens] | 0.74 | 0,0326 |
| A\_51\_P115159 | NM\_027342 | Mus musculus, RIKEN cDNA 2310056P07 gene, clone MGC:19151 IMAGE:4219531, mRNA, complete cds | 0.74 | 0,0485 |
| A\_51\_P501299 | NM\_029203 | Mus musculus RIKEN cDNA 4930539I12 gene (4930539I12Rik), mRNA | 0.74 | 0,0110 |
| A\_51\_P288549 | BC016255 | Mus musculus phospholipase A2, group IVB (cytosolic) (Pla2g4b), mRNA | 0.74 | 0,0146 |
| A\_51\_P207754 | NM\_008189 | Mus musculus guanylate cyclase activator 1a (retina) (Guca1a), mRNA | 0.73 | 0,0495 |
| A\_51\_P185902 | NM\_025659 | similar to NESH (NESH PROTEIN) [Homo sapiens] | 0.73 | 0,0382 |
| A\_51\_P217697 | AK006127 | hypothetical protein | 0.73 | 0,0122 |
| A\_51\_P436767 | NM\_146911 | Mus musculus olfactory receptor MOR129-1 (MOR129-1), mRNA | 0.73 | 0,0412 |
| A\_51\_P334903 | NM\_181665 | SEROLOGICALLY DEFINED COLON CANCER ANTIGEN | 0.73 | 0,0342 |
| A\_51\_P314472 | NM\_139234 | ACTIN-BINDING PROTEIN FRABIN-GAMMA homolog [Mus musculus] | 0.73 | 0,0463 |
| A\_51\_P197923 | NM\_009559 | Mus musculus zinc finger protein 57 (Zfp57), mRNA | 0.73 | 0,0182 |
| A\_51\_P315193 | NM\_178400 | unknown EST | 0.73 | 0,0247 |
| A\_51\_P478238 | NM\_031387 | Mus musculus tudor domain containing 1 (Tdrd1), mRNA | 0.73 | 0,0070 |
| A\_51\_P491366 | NM\_026112 | ZINC FINGER PROTEIN 328 homolog [Homo sapiens] | 0.73 | 0,0303 |
| A\_51\_P477830 | XM\_144142 | hypothetical Galactose oxidase, central domain structure containing protein | 0.73 | 0,0143 |
| A\_51\_P201346 | NM\_010786 | Mus musculus transformed mouse 3T3 cell double minute 2 (Mdm2), mRNA | 0.73 | 0,0239 |
| A\_51\_P167198 | NM\_146985 | Mus musculus olfactory receptor MOR227-1 (MOR227-1), mRNA | 0.73 | 0,0432 |
| A\_51\_P205739 | NM\_016666 | Mus musculus aryl-hydrocarbon receptor-interacting protein (Aip), mRNA | 0.73 | 0,0186 |
| A\_51\_P350403 | NM\_009148 | Mus musculus SEC8 (S. cerevisiae) (Sec8), mRNA | 0.73 | 0,0190 |
| A\_51\_P408537 | NM\_009054 | Mus musculus tripartite motif protein 27 (Trim27), mRNA | 0.73 | 0,0240 |
| A\_51\_P408249 | AK013187 | unknown EST | 0.73 | 0,0043 |
| A\_51\_P279683 | AK005991 | RIKEN cDNA 1700015F03 gene | 0.73 | 0,0325 |
| A\_51\_P450547 | NM\_011060 | Mus musculus peptidyl arginine deiminase, type III (Pdi3), mRNA | 0.73 | 0,0382 |
| A\_51\_P356883 | NM\_008728 | Mus musculus natriuretic peptide receptor 3 (Npr3), mRNA | 0.73 | 0,0258 |
| A\_51\_P473509 | NM\_026423 | Mus musculus RIKEN cDNA 2410018C20 gene (2410018C20Rik), mRNA | 0.73 | 0,0153 |
| A\_51\_P453909 | NM\_007817 | Mus musculus cytochrome P450, 2f2 (Cyp2f2), mRNA | 0.73 | 0,0400 |
| A\_51\_P199674 | Y00746 | Mus musculus phosphodiesterase 6G, cGMP-specific, rod, gamma (Pde6g), mRNA | 0.73 | 0,0319 |
| A\_51\_P464900 | BC054735 | Mus musculus gamma-aminobutyric acid (GABA-B) receptor, 1 (Gabbr1), mRNA | 0.73 | 0,0231 |
| A\_51\_P505738 | NM\_145635 | Mus musculus SMAF1 mRNA, complete cds | 0.73 | 0,0424 |
| A\_51\_P486240 | NM\_011606 | Mus musculus tetranectin (plasminogen binding protein) (Tna), mRNA | 0.73 | 0,0319 |
| A\_51\_P134030 | NM\_145210 | Mus musculus 2-5 oligoadenylate synthetase 1E (Oas1e), mRNA | 0.73 | 0,0042 |
| A\_51\_P397454 | AK038921 | GAMMA TUBULIN RING COMPLEX PROTEIN homolog [Homo sapiens] | 0.73 | 0,0348 |
| A\_51\_P249544 | NM\_029621 | Mus musculus RIKEN cDNA 2410004L22 gene (2410004L22Rik), mRNA | 0.73 | 0,0207 |
| A\_51\_P380239 | BC014761 | Mus musculus, Similar to leucine rich repeat (in FLII) interacting protein 2, clone MGC:25637 IMAGE:4217995, mRNA, complete cds | 0.73 | 0,0037 |
| A\_51\_P252328 | AK076417 | HYPOTHETICAL 28.4 KDA PROTEIN homolog [Homo sapiens] | 0.73 | 0,0111 |
| A\_51\_P278353 | AK006745 | hypothetical protein | 0.73 | 0,0220 |
| A\_51\_P436530 | NM\_008971 | Mus musculus protein tyrosine kinase 9 (Ptk9), mRNA | 0.73 | 0,0315 |
| A\_51\_P102106 | NM\_146364 | Mus musculus olfactory receptor MOR204-37 (MOR204-37), mRNA | 0.73 | 0,0047 |
| A\_51\_P424990 | AK045732 | hypothetical Kelch repeat containing protein | 0.73 | 0,0236 |
| A\_51\_P409101 | AK006216 | hypothetical Cadherin structure containing protein | 0.73 | 0,0027 |
| A\_51\_P405074 | AK015402 | hypothetical protein | 0.73 | 0,0316 |
| A\_51\_P377171 | NM\_183264 | hypothetical protein | 0.73 | 0,0287 |
| A\_51\_P172935 | NM\_026092 | Mus musculus RIKEN cDNA 1700038F02 gene (1700038F02Rik), mRNA | 0.73 | 0,0341 |
| A\_51\_P475138 | XM\_130483 | DUAL OXIDASE homolog [Homo sapiens] | 0.73 | 0,0375 |
| A\_51\_P267861 | NM\_021542 | Mus musculus potassium channel, subfamily K, member 5 (Kcnk5), mRNA | 0.73 | 0,0212 |
| A\_51\_P268625 | NM\_007992 | Mus musculus fibulin 2 (Fbln2), mRNA | 0.73 | 0,0372 |
| A\_51\_P191463 | AY302216 | MHC NONCLASSICAL CLASS I THY19.4 GENE (H-2 D HAPLOTYPE) (FRAGMENT) | 0.73 | 0,0281 |
| A\_51\_P183165 | NM\_146129 | Mus musculus RIKEN cDNA F730014I05 gene (F730014I05Rik), mRNA | 0.73 | 0,0201 |
| A\_51\_P199148 | NM\_025946 | Mus musculus RIKEN cDNA 2010100O12 gene (2010100O12Rik), mRNA | 0.73 | 0,0280 |
| A\_51\_P365694 | NM\_019491 | Mus musculus v-ral simian leukemia viral oncogene homolog A (ras related) (Rala), mRNA | 0.73 | 0,0411 |
| A\_51\_P178887 | NM\_008642 | Mus musculus microsomal triglyceride transfer protein (Mttp), mRNA | 0.73 | 0,0189 |
| A\_51\_P494491 | NM\_008888 | Mus musculus paired mesoderm homeobox 2b (Pmx2b), mRNA | 0.73 | 0,0314 |
| A\_51\_P151373 | AK016539 | nonhistone chromosomal protein HMG-1 homolog [Bos primigenius taurus] | 0.73 | 0,0486 |
| A\_51\_P310910 | A\_51\_P310910 | NEURONAL | 0.73 | 0,0493 |
| A\_51\_P358683 | NM\_183031 | EBV-INDUCED G PROTEIN-COUPLED RECEPTOR 2 (EBI2) homolog [Homo sapiens] | 0.73 | 0,0168 |
| A\_51\_P266717 | XM\_125542 | CGI 130 | 0.73 | 0,0237 |
| A\_51\_P377948 | NM\_174960 | weakly similar to HUMAN IMMUNITY ASSOCIATED PROTEIN 1 [Homo sapiens] | 0.73 | 0,0081 |
| A\_51\_P189361 | NM\_027950 | similar to PREGNANCY-INDUCED GROWTH INHIBITOR [Homo sapiens] | 0.73 | 0,0331 |
| A\_51\_P234092 | A\_51\_P234092 | Mus musculus cDNA, 3 end | 0.73 | 0,0367 |
| A\_51\_P479181 | NM\_009704 | amphiregulin | 0.73 | 0,0315 |
| A\_51\_P443508 | NM\_026438 | Mus musculus RIKEN cDNA 2010317E03 gene (2010317E03Rik), mRNA | 0.73 | 0,0081 |
| A\_51\_P385478 | AK019589 | hypothetical protein | 0.73 | 0,0032 |
| A\_51\_P448608 | NM\_008738 | Mus musculus neurturin (Nrtn), mRNA | 0.73 | 0,0094 |
| A\_51\_P453736 | XM\_283314 | hypothetical protein | 0.73 | 0,0491 |
| A\_51\_P194293 | NM\_020264 | Mus musculus seminal vesicle protein, secretion 7 (Svs7), mRNA | 0.73 | 0,0386 |
| A\_51\_P465918 | NM\_172721 | weakly similar to F-BOX PROTEIN FBX29 (FRAGMENT) [Homo sapiens] | 0.73 | 0,0011 |
| A\_51\_P394921 | NM\_025492 | RIKEN cDNA 1700020L24 gene | 0.73 | 0,0222 |
| A\_51\_P235139 | AK078669 | KELCH LIKE PROTEIN | 0.73 | 0,0163 |
| A\_51\_P121485 | NM\_146625 | OLFACTORY RECEPTOR | 0.73 | 0,0022 |
| A\_51\_P360809 | NM\_144783 | Mouse Wilms tumor mRNA, complete cds | 0.73 | 0,0254 |
| A\_51\_P396752 | NM\_024191 | Mus musculus RIKEN cDNA 6330544B05 gene (6330544B05Rik), mRNA | 0.73 | 0,0491 |
| A\_51\_P115905 | AK036480 | inferred: KIAA1742 protein {Homo sapiens} | 0.73 | 0,0234 |
| A\_51\_P299795 | AK033746 | hypothetical protein | 0.73 | 0,0382 |
| A\_51\_P298455 | NM\_175427 | weakly similar to UNKNOWN (PROTEIN FOR MGC:16664) [Homo sapiens] | 0.73 | 0,0083 |
| A\_51\_P521106 | NM\_011652 | TITIN homolog [Homo sapiens] | 0.73 | 0,0118 |
| A\_51\_P347764 | NM\_021315 | Mus musculus unknown mRNA | 0.73 | 0,0072 |
| A\_51\_P151271 | NM\_145625 | EUKARYOTIC INITIATION FACTOR 4B homolog [Homo sapiens] | 0.73 | 0,0301 |
| A\_51\_P120295 | NM\_029562 | Mus musculus RIKEN cDNA 1300006E06 gene (1300006E06Rik), mRNA | 0.73 | 0,0147 |
| A\_51\_P167403 | NM\_031368 | Mus musculus, Similar to bone gamma-carboxyglutamate protein, related sequence 1, clone MGC:7287 IMAGE:3485278, mRNA, complete cds | 0.73 | 0,0488 |
| A\_51\_P468329 | NM\_011187 | Mus musculus proteasome (prosome, macropain) subunit, beta type 7 (Psmb7), mRNA | 0.73 | 0,0164 |
| A\_51\_P149699 | NM\_013534 | Mus musculus gene rich cluster, B gene (Grcb), mRNA | 0.73 | 0,0167 |
| A\_51\_P284608 | NM\_010545 | H-2 CLASS II HISTOCOMPATIBILITY ANTIGEN, GAMMA CHAIN (MHC CLASS II(IA) ASSOCIATED INVARIANT CHAIN) | 0.73 | 0,0278 |
| A\_51\_P114037 | AK041084 | unknown EST | 0.73 | 0,0061 |
| A\_51\_P231549 | NM\_153760 | Mus musculus MHC I - like leukocyte 2 (Mill2), mRNA | 0.73 | 0,0203 |
| A\_51\_P184162 | NM\_178755 | hypothetical Zn-dependent exopeptidases structure containing protein | 0.73 | 0,0211 |
| A\_51\_P334344 | AK047044 | Mus musculus mRNA similar to CGI-72 protein (cDNA clone MGC:28361 IMAGE:4019308), complete cds | 0.73 | 0,0021 |
| A\_51\_P376050 | NM\_029495 | Mus musculus, RIKEN cDNA 5033415K03 gene, clone MGC:7873 IMAGE:3581940, mRNA, complete cds | 0.73 | 0,0348 |
| A\_51\_P219786 | AY989880 | H-2 CLASS I HISTOCOMPATIBILITY ANTIGEN, D-B ALPHA CHAIN PRECURSOR (H-2D(B)) | 0.73 | 0,0304 |
| A\_51\_P465512 | NM\_008708 | Mus musculus N-myristoyltransferase 2 (Nmt2), mRNA | 0.73 | 0,0260 |
| A\_51\_P178591 | NM\_053158 | Mus musculus mitochondrial ribosomal protein L1 (Mrpl1), mRNA | 0.73 | 0,0113 |
| A\_51\_P435497 | NM\_008637 | Mus musculus nudix (nucleoside diphosphate linked moiety X)-type motif 1 (Nudt1), mRNA | 0.73 | 0,0120 |
| A\_51\_P300726 | NM\_011014 | Mus musculus opioid receptor, sigma 1 (Oprs1), mRNA | 0.73 | 0,0239 |
| A\_51\_P131093 | BF783780 | Mus musculus cDNA, 5 end | 0.73 | 0,0495 |
| A\_51\_P176797 | NM\_172397 | EPITHELIAL PROTEIN LOST IN | 0.73 | 0,0180 |
| A\_51\_P425372 | NM\_028047 | CDNA FLJ12886 FIS, CLONE NT2RP2004041, WEAKLY SIMILAR TO SYNAPSINS IA AND IB homolog [Homo sapiens] | 0.73 | 0,0136 |
| A\_51\_P188155 | NM\_080644 | Mus musculus voltage-dependent calcium channel gamma-5 subunit (Cacng5) mRNA, complete cds | 0.73 | 0,0070 |
| A\_51\_P418644 | AK087566 | hypothetical Gram-positive cocci surface protein anchoring hexapeptide containing protein | 0.73 | 0,0204 |
| A\_51\_P306049 | NM\_024206 | Mus musculus RIKEN cDNA 1110003H02 gene (1110003H02Rik), mRNA | 0.73 | 0,0254 |
| A\_51\_P397437 | NM\_183103 | hypothetical Serine proteases, trypsin family containing protein | 0.73 | 0,0319 |
| A\_51\_P337350 | NM\_012016 | Mus musculus endoplasmic reticulum (ER) to nucleus signalling 2 (Ern2), mRNA | 0.73 | 0,0126 |
| A\_51\_P290191 | NM\_008243 | Mus musculus hepatocyte growth factor-like (Hgfl), mRNA | 0.72 | 0,0222 |
| A\_51\_P445479 | NM\_013524 | Mus musculus fucosyltransferase 7 (Fut7), mRNA | 0.72 | 0,0260 |
| A\_51\_P279704 | BC024679 | Mus musculus, Similar to hypothetical gene LOC133157, clone IMAGE:3991705, mRNA, partial cds | 0.72 | 0,0120 |
| A\_51\_P305003 | AK081588 | neurotrophic tyrosine kinase, receptor, type 1 | 0.72 | 0,0088 |
| A\_51\_P279888 | NM\_001029933 | Mus musculus zinc finger protein ZFP110 mRNA, partial cds | 0.72 | 0,0063 |
| A\_51\_P264468 | BC026492 | Mus musculus RIKEN cDNA 2200005K02 gene (2200005K02Rik), mRNA | 0.72 | 0,0087 |
| A\_51\_P227202 | AK021081 | hypothetical protein | 0.72 | 0,0127 |
| A\_51\_P506714 | AK012596 | similar to PUTATIVE RNA BINDING PROTEIN [Homo sapiens] | 0.72 | 0,0378 |
| A\_51\_P415635 | NM\_146554 | Mus musculus olfactory receptor MOR111-3 (MOR111-3), mRNA | 0.72 | 0,0398 |
| A\_51\_P221886 | NM\_172381 | hypothetical ARM repeat structure containing protein | 0.72 | 0,0126 |
| A\_51\_P406077 | NM\_025363 | Mus musculus RIKEN cDNA 1110001J03 gene (1110001J03Rik), mRNA | 0.72 | 0,0225 |
| A\_51\_P132491 | NM\_175008 | "hypothetical EF-hand/Guanine nucleotide exchange factor for Ras-like GTPases� N-terminal motif containing protein" | 0.72 | 0,0074 |
| A\_51\_P345975 | NM\_022883 | Mus musculus lipin 3 (Lpin3), mRNA | 0.72 | 0,0030 |
| A\_51\_P403393 | NM\_026099 | Mus musculus RIKEN cDNA 1700065I17 gene (1700065I17Rik), mRNA | 0.72 | 0,0141 |
| A\_51\_P175842 | NM\_013528 | Mus musculus glutamine fructose-6-phosphate transaminase 1 (Gfpt1), mRNA | 0.72 | 0,0095 |
| A\_51\_P352068 | D12885 | Mus musculus pituitary specific transcription factor 1 (Pit1), mRNA | 0.72 | 0,0392 |
| A\_51\_P379037 | AF201683 | Mus musculus DiGeorge syndrome critical region gene 8 (Dgcr8), mRNA | 0.72 | 0,0212 |
| A\_51\_P476767 | NM\_010924 | Mus musculus nicotinamide N-methyltransferase (Nnmt), mRNA | 0.72 | 0,0122 |
| A\_51\_P411370 | NAP057215-1 | Mus musculus olfactory receptor MOR121-4P (MOR121-4P) pseudogene | 0.72 | 0,0185 |
| A\_51\_P384306 | NM\_015788 | Mus musculus sacsin (Sacs), mRNA | 0.72 | 0,0165 |
| A\_51\_P341571 | NM\_027117 | Mus musculus, RIKEN cDNA 2310022K15 gene, clone MGC:11736 IMAGE:3968615, mRNA, complete cds | 0.72 | 0,0381 |
| A\_51\_P482488 | NAP108772-1 | Mus musculus acyl-CoA-binding protein ACBP pseudogene (Dbi-ps) on chromosome 11 | 0.72 | 0,0343 |
| A\_51\_P227342 | NM\_007876 | Mus musculus dipeptidase 1 (renal) (Dpep1), mRNA | 0.72 | 0,0097 |
| A\_51\_P321643 | NM\_173371 | "similar to GDH/6PGL ENDOPLASMIC BIFUNCTIONAL PROTEIN PRECURSOR [INCLUDES: GLUCOSE 1-DEHYDROGENASE (EC 1.1.1.47) (HEXOSE-6-PHOSPHATE DEHYDROGENASE)� 6- PHOSPHOGLUCONOLACTONASE (EC 3.1.1.31) (6PGL)] [Homo sapiens]" | 0.72 | 0,0040 |
| A\_51\_P410187 | BC037267 | Mus musculus RIKEN cDNA 4632415L05 gene (4632415L05Rik), mRNA | 0.72 | 0,0400 |
| A\_51\_P199104 | NM\_009813 | Mus musculus calsequestrin 1 (Casq1), mRNA | 0.72 | 0,0084 |
| A\_51\_P385639 | NM\_010291 | Mus musculus gap junction membrane channel protein beta 5 (Gjb5), mRNA | 0.72 | 0,0072 |
| A\_51\_P192991 | A\_51\_P192991 | Mus musculus cDNA, 5 end | 0.72 | 0,0031 |
| A\_51\_P482289 | NM\_023525 | "CAD PROTEIN [INCLUDES: GLUTAMINE DEPENDENT CARBAMOYL PHOSPHATE SYNTHASE EC 6.3.5.5�� ASPARTATE CARBAMOYLTRANSFERASE EC 2.1.3.- 2� DIHYDROOROTASE EC 3.5.-.- 2 3" | 0.72 | 0,0276 |
| A\_51\_P496668 | NM\_028938 | hypothetical IQ calmodulin-binding motif/Protein splicing (intein)/Leucine-rich repeat containing protein | 0.72 | 0,0077 |
| A\_51\_P487308 | NM\_177609 | hypothetical P-loop containing nucleotide triphosphate hydrolases structure containing protein | 0.72 | 0,0466 |
| A\_51\_P413075 | NM\_175524 | hypothetical Rhodopsin-like GPCR superfamily containing protein | 0.72 | 0,0241 |
| A\_51\_P344531 | NM\_027660 | similar to CDNA FLJ32828 FIS, CLONE TESTI2003117, WEAKLY SIMILAR TO TEKTIN A1 [Homo sapiens] | 0.72 | 0,0264 |
| A\_51\_P507571 | NM\_013638 | Mus musculus protamine 3 (Prm3), mRNA | 0.72 | 0,0031 |
| A\_51\_P321930 | AK002366 | hypothetical protein | 0.72 | 0,0089 |
| A\_51\_P495719 | XM\_484943 | hypothetical Alanine-rich region containing protein | 0.72 | 0,0368 |
| A\_51\_P372302 | XM\_355182 | Mus musculus, clone IMAGE:2811941, mRNA | 0.72 | 0,0074 |
| A\_51\_P465582 | NM\_024257 | hypothetical Haloacid dehalogenase/epoxide hydrolase family containing protein | 0.72 | 0,0044 |
| A\_51\_P355301 | NM\_007818 | Mus musculus cytochrome P450, family 3, subfamily a, polypeptide 11 (Cyp3a11), mRNA | 0.72 | 0,0362 |
| A\_51\_P246667 | NM\_146097 | DOPAMINE RESPONSIVE PROTEIN homolog [Rattus norvegicus] | 0.72 | 0,0168 |
| A\_51\_P184300 | NM\_010087 | Mus musculus dystrobrevin alpha (Dtna), mRNA | 0.72 | 0,0323 |
| A\_51\_P505792 | NM\_145391 | NAD P TRANSHYDROGENASE, MITOCHONDRIAL PRECURSOR EC 1.6.1.2 PYRIDINE NUCLEOTIDE TRANSHYDROGENASE NICOTINAMIDE NUCLEOTIDE | 0.72 | 0,0216 |
| A\_51\_P240760 | NM\_028189 | Mus musculus UDP-GlcNAc:betaGal beta-1,3-N-acetylglucosaminyltransferase 3 (B3gnt3), mRNA | 0.72 | 0,0287 |
| A\_51\_P296737 | NM\_027211 | Mus musculus annexin A13 (Anxa13), mRNA | 0.72 | 0,0168 |
| A\_51\_P120201 | NM\_181582 | Mus musculus, eukaryotic translation initiation factor 5A, clone MGC:25474 IMAGE:4482804, mRNA, complete cds | 0.72 | 0,0309 |
| A\_51\_P251465 | NM\_025644 | 3-5 EXORIBONUCLEASE CSL4 HOMOLOG (EC 3.1.13.-) (CGI-108) homolog [Homo sapiens] | 0.72 | 0,0414 |
| A\_51\_P422030 | NM\_029865 | Mus musculus, Similar to RIKEN cDNA 9430098E02 gene, clone MGC:30323 IMAGE:5151818, mRNA, complete cds | 0.72 | 0,0396 |
| A\_51\_P482990 | NM\_145996 | similar to MODULATOR RECOGNITION FACTOR I (FRAGMENT) [Homo sapiens] | 0.72 | 0,0151 |
| A\_51\_P222092 | NM\_008784 | Mus musculus alpha 4 protein mRNA, 5 end | 0.72 | 0,0138 |
| A\_51\_P471057 | AF357403 | Mus musculus clone MBI-100 H/ACA box snoRNA, partial sequence | 0.72 | 0,0081 |
| A\_51\_P259778 | XM\_143619 | RETINAL SHORT CHAIN DEHYDROGENASE | 0.72 | 0,0029 |
| A\_51\_P504672 | NM\_133665 | Mus musculus myocyte enhancer factor 2D (Mef2d), mRNA | 0.72 | 0,0209 |
| A\_51\_P428977 | BC023444 | musculus, clone MGC:32491 IMAGE:5053834, mRNA, complete cds | 0.72 | 0,0022 |
| A\_51\_P163901 | NM\_146899 | Mus musculus olfactory receptor MOR233-6 (MOR233-6), mRNA | 0.72 | 0,0105 |
| A\_51\_P494196 | NM\_009709 | Mus musculus aryl hydrocarbon receptor nuclear translocator (Arnt), mRNA | 0.72 | 0,0479 |
| A\_51\_P159171 | NM\_172161 | similar to interleukin-1 receptor-associated kinase 2 [Homo sapiens] | 0.72 | 0,0364 |
| A\_51\_P119266 | NM\_011768 | Mus musculus zinc finger protein X-linked (Zfx), mRNA | 0.72 | 0,0193 |
| A\_51\_P425279 | AK018541 | hypothetical protein | 0.72 | 0,0259 |
| A\_51\_P214786 | NM\_053079 | Mus musculus solute carrier family 15 (oligopeptide transporter), member 1 (Slc15a1), mRNA | 0.72 | 0,0035 |
| A\_51\_P415945 | NM\_008944 | Mus musculus proteasome (prosome, macropain) subunit, alpha type 2 (Psma2), mRNA | 0.72 | 0,0260 |
| A\_51\_P120680 | AK082760 | weakly similar to KRUPPEL-RELATED ZINC FINGER PROTEIN F80-L [Mus musculus] | 0.72 | 0,0135 |
| A\_51\_P491648 | NM\_153786 | TRANSCRIPTION COFACTOR VESTIGIAL-LIKE 2 PROTEIN homolog [Homo sapiens] | 0.72 | 0,0305 |
| A\_51\_P215489 | NM\_153062 | GLYCEROL-3-PHOSPHATE TRANSPORTER (G-3-P TRANSPORTER) (G-3-P PERMEASE) homolog [Homo sapiens] | 0.72 | 0,0128 |
| A\_51\_P234716 | AK005533 | Mus musculus CEA-related cell adhesion molecule 14 (Ceacam14), mRNA | 0.72 | 0,0428 |
| A\_51\_P486207 | AK032580 | weakly similar to REVERSE TRANSCRIPTASE (FRAGMENT) [Sheep pulmonary adenomatosis virus] | 0.72 | 0,0142 |
| A\_51\_P297244 | AK016107 | weakly similar to PUTATIVE KERATIN-ASSOCIATED PROTEIN (KERATIN-ASSOCIATED PROTEIN 13) [Mus musculus] | 0.72 | 0,0431 |
| A\_51\_P246627 | AK030188 | Kinesin superfamily protein 2B | 0.72 | 0,0030 |
| A\_51\_P446315 | NM\_025950 | Mus musculus cell division cycle 37 homolog (S. cerevisiae)-like (Cdc37l), mRNA | 0.72 | 0,0019 |
| A\_51\_P326152 | NM\_011377 | Mus musculus single-minded 2 (Sim2), mRNA | 0.72 | 0,0182 |
| A\_51\_P453079 | NM\_148943 | Mus musculus mRNA for putative ubiquitin-specific protease (Usp9y gene) | 0.72 | 0,0412 |
| A\_51\_P206704 | NM\_053115 | Mus musculus acyl-Coenzyme A oxidase 2, branched chain (Acox2), mRNA | 0.72 | 0,0434 |
| A\_51\_P301215 | NM\_023197 | Mus musculus RIKEN cDNA 2310008H09 gene (2310008H09Rik), mRNA | 0.72 | 0,0495 |
| A\_51\_P452016 | NM\_033570 | Mus musculus cyclin M4 (Cnnm4), mRNA | 0.71 | 0,0022 |
| A\_51\_P477779 | NM\_013646 | Mus musculus RAR-related orphan receptor alpha (Rora), mRNA | 0.71 | 0,0310 |
| A\_51\_P377154 | NM\_009997 | Mus musculus cytochrome P450, 2a4 (Cyp2a4), mRNA | 0.71 | 0,0129 |
| A\_51\_P153029 | XM\_144855 | hypothetical Ankyrin repeat profile/Ankyrin-repeat/Ankyrin repeat region circular profile/Yeast DNA-binding domain containing protein | 0.71 | 0,0185 |
| A\_51\_P457658 | NM\_010010 | Mus musculus cytochrome P450, 46 (cholesterol 24-hydroxylase) (Cyp46), mRNA | 0.71 | 0,0382 |
| A\_51\_P148421 | NM\_029082 | Mus musculus RIKEN cDNA 5830411J07 gene (5830411J07Rik), mRNA | 0.71 | 0,0091 |
| A\_51\_P175611 | NM\_033614 | Mus musculus phosphodiesterase 6C, cGMP specific, cone, alpha prime (Pde6c), mRNA | 0.71 | 0,0022 |
| A\_51\_P353118 | AK014334 | hypothetical Eukaryotic protein kinase containing protein | 0.71 | 0,0145 |
| A\_51\_P461040 | NM\_028798 | similar to NICE-1 PROTEIN [Homo sapiens] | 0.71 | 0,0161 |
| A\_51\_P147527 | NM\_022030 | Mus musculus synaptic vesicle glycoprotein 2 a (Sv2a), mRNA | 0.71 | 0,0065 |
| A\_51\_P161890 | NM\_010189 | Mus musculus Fc receptor, IgG, alpha chain transporter (Fcgrt), mRNA | 0.71 | 0,0206 |
| A\_51\_P394207 | NM\_007986 | Mus musculus fibroblast activation protein (Fap), mRNA | 0.71 | 0,0073 |
| A\_51\_P445985 | NM\_019415 | Mus musculus solute carrier family 12, member 3 (Slc12a3), mRNA | 0.71 | 0,0104 |
| A\_51\_P196973 | NM\_013733 | Mus musculus chromatin assembly factor 1, subunit A (p150) (Chaf1a), mRNA | 0.71 | 0,0356 |
| A\_51\_P293789 | NM\_007498 | Mus musculus activating transcription factor 3 (Atf3), mRNA | 0.71 | 0,0113 |
| A\_51\_P517952 | NM\_146206 | hypothetical Calcium and sodium channel pore region (S4-S6)/Cation channels TM region (not potassium)/Cation channels (non-ligand gated) containing protein | 0.71 | 0,0385 |
| A\_51\_P251892 | NM\_010760 | Mus musculus mago-nashi homolog, proliferation-associated (Drosophila) (Magoh), mRNA | 0.71 | 0,0324 |
| A\_51\_P476706 | NM\_175526 | weakly similar to C-TYPE LECTIN-LIKE RECEPTOR-1 [Homo sapiens] | 0.71 | 0,0297 |
| A\_51\_P359272 | NM\_009655 | Mus musculus activated leukocyte cell adhesion molecule CD166 (ALCAM) mRNA, complete cds | 0.71 | 0,0043 |
| A\_51\_P254181 | NM\_029629 | unknown | 0.71 | 0,0106 |
| A\_51\_P251977 | NM\_134184 | Mus musculus vomeronasal 1 receptor, C29 (V1rc29), mRNA | 0.71 | 0,0322 |
| A\_51\_P187716 | AK003350 | unclassifiable | 0.71 | 0,0056 |
| A\_51\_P327418 | NM\_025438 | weakly similar to SPLICING FACTOR, ARGININE/SERINE-RICH 6 (PRE-MRNA SPLICING FACTOR SRP55) (FRAGMENT) [Oryctolagus cuniculus] | 0.71 | 0,0125 |
| A\_51\_P376979 | NM\_025789 | Mus musculus RIKEN cDNA 4930524H12 gene (4930524H12Rik), mRNA | 0.71 | 0,0131 |
| A\_51\_P172251 | NM\_025903 | Mus musculus interferon-related developmental regulator 2 (Ifrd2), mRNA | 0.71 | 0,0033 |
| A\_51\_P391694 | NM\_009163 | Mus musculus sphingosine phosphate lyase 1 (Sgpl1), mRNA | 0.71 | 0,0106 |
| A\_51\_P180108 | NM\_028809 | Mus musculus, RIKEN cDNA 2010015J01 gene, clone MGC:37396 IMAGE:4977391, mRNA, complete cds | 0.71 | 0,0284 |
| A\_51\_P487950 | XM\_483962 | Mus musculus brush border myosin-I (BBM-I) mRNA, partial cds | 0.71 | 0,0113 |
| A\_51\_P395041 | NM\_009759 | Mus musculus BMX non-receptor tyrosine kinase (Bmx), mRNA | 0.71 | 0,0156 |
| A\_51\_P461703 | NM\_031188 | Mus musculus major urinary protein 1 (Mup1), mRNA | 0.71 | 0,0459 |
| A\_51\_P377641 | NM\_028434 | hypothetical protein | 0.71 | 0,0248 |
| A\_51\_P256427 | AK021165 | unclassifiable | 0.71 | 0,0057 |
| A\_51\_P223583 | NM\_134257 | PDZ-RGS3 PROTEIN | 0.71 | 0,0121 |
| A\_51\_P325281 | XM\_129595 | similar to INTERFERON-ACTIVATABLE PROTEIN 204 (IFI-204) (INTERFERON-INDUCIBLE PROTEIN P204) [Mus musculus] | 0.71 | 0,0074 |
| A\_51\_P484010 | NM\_007941 | Mus musculus epimorphin (Epim), mRNA | 0.71 | 0,0077 |
| A\_51\_P346304 | NM\_198169 | Mus musculus hypothetical protein LOC229004, mRNA (cDNA clone IMAGE:4953646), partial cds | 0.71 | 0,0017 |
| A\_51\_P515452 | NM\_019873 | Mus musculus FK506 binding protein-like (Fkbpl), mRNA | 0.71 | 0,0252 |
| A\_51\_P311965 | NM\_009298 | Mus musculus surfeit gene 6 (Surf6), mRNA | 0.71 | 0,0231 |
| A\_51\_P337517 | NM\_019421 | Mus musculus hypothetical protein 425O18-1 (425O18-1), mRNA | 0.71 | 0,0134 |
| A\_51\_P512783 | NM\_025476 | Mus musculus RIKEN cDNA 2410005O16 gene (2410005O16Rik), mRNA | 0.71 | 0,0443 |
| A\_51\_P189208 | AK016844 | similar to SEC13-RELATED PROTEIN [Homo sapiens] | 0.71 | 0,0056 |
| A\_51\_P161268 | NM\_009138 | Mus musculus small inducible cytokine A25 (Scya25), mRNA | 0.71 | 0,0260 |
| A\_51\_P205209 | NM\_018729 | Mus musculus non MHC restricted killing associated (Nmrk), mRNA | 0.71 | 0,0081 |
| A\_51\_P445882 | AK018014 | musculus adult male thymus cDNA, RIKEN full-length enriched library, clone:5830455I16:T-cell receptor beta, joining region, full insert sequence | 0.71 | 0,0077 |
| A\_51\_P497061 | NM\_026391 | Mus musculus protein phosphatase 2A B regulatory subunit delta isoform mRNA, complete cds | 0.71 | 0,0314 |
| A\_51\_P449334 | AK007945 | ubiquitin-activating enzyme E1, Chr Y 1 | 0.71 | 0,0322 |
| A\_51\_P367934 | NM\_130877 | Mus musculus mRNA for Edr protein | 0.71 | 0,0115 |
| A\_51\_P317417 | AK087383 | unknown EST | 0.71 | 0,0474 |
| A\_51\_P205390 | NM\_028491 | hypothetical protein | 0.71 | 0,0390 |
| A\_51\_P107315 | NM\_145423 | Mus musculus similar to Sodium/iodide cotransporter (Na(+)/I(-) cotransporter) (Sodium-iodide symporter) (Na+/I-symporter) (LOC216225), mRNA | 0.71 | 0,0032 |
| A\_51\_P286073 | AK015500 | hypothetical protein | 0.71 | 0,0259 |
| A\_51\_P108479 | NM\_026826 | similar to MITOCHONDRIAL 28S RIBOSOMAL PROTEIN S18-1 (MRP-S18-1) [Homo sapiens] | 0.71 | 0,0082 |
| A\_51\_P403137 | NM\_028981 | Mus musculus calcium channel, voltage-dependent, L type, alpha 1D subunit (Cacna1d), mRNA | 0.71 | 0,0403 |
| A\_51\_P412955 | AK044842 | hypothetical Phosphatidylinositol-specific phospholipase C, X domain containing protein | 0.71 | 0,0074 |
| A\_51\_P385749 | NM\_026902 | MULTIPLE COPIES IN A T-CELL MALIGNANCIES (MCT-1 PROTEIN) homolog [Homo sapiens] | 0.71 | 0,0287 |
| A\_51\_P308948 | XM\_132806 | similar to HYPOTHETICAL 35.9 KDA PROTEIN [Homo sapiens] | 0.71 | 0,0106 |
| A\_51\_P252157 | NM\_011623 | Mus musculus topoisomerase (DNA) II alpha (Top2a), mRNA | 0.71 | 0,0028 |
| A\_51\_P368660 | NM\_026483 | Mus musculus, Similar to M-phase phosphoprotein 10 (U3 small nucleolar ribonucleoprotein), clone IMAGE:3979017, mRNA | 0.71 | 0,0019 |
| A\_51\_P223363 | NM\_025783 | NEUROENDOCRINE DIFFERENTIATION FACTOR homolog [Homo sapiens] | 0.71 | 0,0137 |
| A\_51\_P235784 | NM\_008099 | Mus musculus germinal center expressed transcript (Gcet), mRNA | 0.71 | 0,0417 |
| A\_51\_P139280 | NM\_007773 | Mus musculus crystallin, beta B2 (Crybb2), mRNA | 0.71 | 0,0034 |
| A\_51\_P112627 | NM\_009180 | Mus musculus sialyltransferase 7 ((alpha-N-acetylneuraminyl 2,3-beta-galactosyl-1,3)-N-acetyl galactosaminde alpha-2,6-sialyltransferase) B (Siat7b), mRNA | 0.71 | 0,0072 |
| A\_51\_P320304 | NM\_011122 | Mus musculus procollagen-lysine, 2-oxoglutarate 5-dioxygenase 1 (Plod1), mRNA | 0.71 | 0,0084 |
| A\_51\_P323615 | AF335543 | Mus musculus histocompatibility 47 (H47), mRNA | 0.71 | 0,0079 |
| A\_51\_P286780 | BC028808 | Mus musculus, clone IMAGE:1244192, mRNA | 0.71 | 0,0199 |
| A\_51\_P430563 | NM\_008098 | Mus musculus granule cell differentiation protein (Gcdp), mRNA | 0.71 | 0,0420 |
| A\_51\_P353360 | NM\_011811 | phenylalanine-tRNA synthetase-like | 0.71 | 0,0383 |
| A\_51\_P190849 | AK081974 | unclassifiable | 0.71 | 0,0283 |
| A\_51\_P231697 | XM\_112192 | INTEGRIN ALPHA-10 PRECURSOR homolog [Homo sapiens] | 0.71 | 0,0423 |
| A\_51\_P476008 | NM\_013854 | Mus musculus ATP-binding cassette protein (Abcf1) mRNA, partial cds | 0.71 | 0,0038 |
| A\_51\_P468939 | NM\_007831 | Mus musculus deleted in colorectal carcinoma (Dcc), mRNA | 0.71 | 0,0487 |
| A\_51\_P485688 | NM\_007666 | Mus musculus cadherin 6 (Cdh6), mRNA | 0.71 | 0,0378 |
| A\_51\_P415546 | NM\_054074 | Mus musculus defensin beta 6 (Defb6), mRNA | 0.71 | 0,0053 |
| A\_51\_P290974 | XM\_125706 | BCR PROTEIN (FRAGMENT) | 0.71 | 0,0176 |
| A\_51\_P233145 | NM\_023546 | Mus musculus RIKEN cDNA 1700029H17 gene (1700029H17Rik), mRNA | 0.71 | 0,0142 |
| A\_51\_P468658 | NM\_153543 | Mus musculus RIKEN cDNA D330038I09 gene (D330038I09Rik), mRNA | 0.71 | 0,0386 |
| A\_51\_P119670 | NM\_010265 | Mus musculus glucosaminyl (N-acetyl) transferase 1, core 2 (Gcnt1), mRNA | 0.71 | 0,0420 |
| A\_51\_P162671 | NM\_008035 | Mus musculus folate receptor 2 (fetal) (Folr2), mRNA | 0.71 | 0,0025 |
| A\_51\_P403277 | NM\_025816 | Mus musculus RIKEN cDNA 1200003J11 gene (1200003J11Rik), mRNA | 0.70 | 0,0097 |
| A\_51\_P148512 | AK079640 | unknown EST | 0.70 | 0,0321 |
| A\_51\_P149189 | NM\_028459 | Mus musculus mRNA for N-WASP protein | 0.70 | 0,0127 |
| A\_51\_P317272 | NM\_001024919 | hypothetical protein | 0.70 | 0,0417 |
| A\_51\_P106235 | AK085937 | Mus musculus proteasome (prosome, macropain) subunit, alpha type 4 (Psma4), mRNA | 0.70 | 0,0264 |
| A\_51\_P195365 | BC080205 | HYPOTHETICAL 46.9 KDA PROTEIN homolog [Homo sapiens] | 0.70 | 0,0363 |
| A\_51\_P346153 | NM\_009720 | Mus musculus ATX1 (antioxidant protein 1) homolog 1 (yeast) (Atox1), mRNA | 0.70 | 0,0085 |
| A\_51\_P460279 | XM\_131470 | hypothetical protein | 0.70 | 0,0079 |
| A\_51\_P468418 | NM\_145618 | Mus musculus NMDA receptor-regulated gene 2 (Narg2), mRNA | 0.70 | 0,0323 |
| A\_51\_P309370 | NM\_133754 | Mus musculus RIKEN cDNA 2410043F08 gene (2410043F08Rik), mRNA | 0.70 | 0,0074 |
| A\_51\_P212938 | NM\_011930 | Mus musculus chloride channel 7 (Clcn7), mRNA | 0.70 | 0,0244 |
| A\_51\_P368088 | NM\_016754 | Mus musculus myosin light chain, phosphorylatable, fast skeletal muscle (Mylpf), mRNA | 0.70 | 0,0109 |
| A\_51\_P352994 | NM\_146580 | Mus musculus olfactory receptor MOR201-2 (MOR201-2), mRNA | 0.70 | 0,0263 |
| A\_51\_P194099 | NM\_009381 | Mus musculus thyroid hormone responsive SPOT14 homolog (Rattus) (Thrsp), mRNA | 0.70 | 0,0103 |
| A\_51\_P330870 | AK016162 | homeodomain interacting protein kinase 1 | 0.70 | 0,0099 |
| A\_51\_P206734 | NM\_133657 | Mus musculus cytochrome P450, 2a12 (Cyp2a12), mRNA | 0.70 | 0,0201 |
| A\_51\_P191865 | NM\_008481 | Mus musculus laminin-2 alpha2 chain mRNA, complete cds | 0.70 | 0,0264 |
| A\_51\_P291027 | XM\_355528 | hypothetical ARM repeat structure containing protein | 0.70 | 0,0027 |
| A\_51\_P155174 | AK008471 | inferred: Mus musculus, Similar to zinc finger protein 135 (clone pHZ-17), clone MGC:7841 IMAGE:3500812, mRNA, complete cds | 0.70 | 0,0106 |
| A\_51\_P203277 | NM\_134230 | Mus musculus vomeronasal 1 receptor, E11 (V1re11), mRNA | 0.70 | 0,0349 |
| A\_51\_P333349 | NM\_172541 | TRANSMEMBRANE PROTEIN INDUCED BY TUMOR NECROSIS FACTOR ALPHA homolog [Homo sapiens] | 0.70 | 0,0126 |
| A\_51\_P255952 | NM\_021290 | Mus musculus urocortin (Ucn), mRNA | 0.70 | 0,0434 |
| A\_51\_P339822 | NM\_138630 | Mus musculus Rho GTPase activating protein 4 (Arhgap4), mRNA | 0.70 | 0,0299 |
| A\_51\_P146382 | AV242801 | Mus musculus CAG trinucleotide repeat mRNA, partial sequence | 0.70 | 0,0392 |
| A\_51\_P147748 | NM\_010257 | Mus musculus gastrin (Gast), mRNA | 0.70 | 0,0045 |
| A\_51\_P448545 | AK013012 | hypothetical BTB/POZ domain/Kelch repeat containing protein | 0.70 | 0,0142 |
| A\_51\_P427425 | NM\_144793 | hypothetical Mitochondrial energy transfer proteins (carrier protein) containing protein | 0.70 | 0,0084 |
| A\_51\_P326263 | AA623690 | "Knowles Solter mouse 2 cell Mus musculus cDNA clone IMAGE:961976 3 similar to WP:F46F11.4 CE10602 ." | 0.70 | 0,0135 |
| A\_51\_P495876 | NM\_022328 | Mus musculus myeloid/lymphoid or mixed lineage-leukemia translocation to 4 homolog (Drosophila) (Mllt1), mRNA | 0.70 | 0,0207 |
| A\_51\_P353042 | NM\_181589 | weakly similar to ORF FOR OVERLAPPING PROTEIN [Kennedya yellow mosaic virus] | 0.70 | 0,0378 |
| A\_51\_P273596 | NM\_146126 | Mus musculus sorbitol dehydrogenase precursor mRNA, partial cds | 0.70 | 0,0047 |
| A\_51\_P311096 | AK031878 | Mus musculus, Similar to RIKEN cDNA 2410003K15 gene, clone IMAGE:4167150, mRNA | 0.70 | 0,0379 |
| A\_51\_P473506 | NM\_173398 | PROBABLE G PROTEIN-COUPLED RECEPTOR H963 homolog [Homo sapiens] | 0.70 | 0,0097 |
| A\_51\_P145276 | AJ409488 | Mus musculus RNA binding site for Dazl protein, clone dd1 | 0.70 | 0,0369 |
| A\_51\_P340003 | NM\_011516 | M.musculus mRNA for synaptonemal complex protein 1 | 0.70 | 0,0454 |
| A\_51\_P418935 | AK004524 | hypothetical SOCS domain, C-terminus of STAT-inhibitors containing protein | 0.70 | 0,0037 |
| A\_51\_P258359 | NM\_153072 | Mus musculus HUS1B mRNA, complete cds | 0.70 | 0,0421 |
| A\_51\_P226882 | NM\_021309 | Mus musculus SH2 domain protein 2A (Sh2d2a), mRNA | 0.70 | 0,0050 |
| A\_51\_P352572 | NM\_019701 | Mus musculus chloride channel K1-like (Clcnk1l-pending), mRNA | 0.70 | 0,0281 |
| A\_51\_P520262 | AK018056 | hypothetical protein | 0.70 | 0,0269 |
| A\_51\_P429633 | AK005867 | hypothetical protein | 0.70 | 0,0171 |
| A\_51\_P258381 | BC010717 | Mus musculus, clone IMAGE:4009470, mRNA | 0.70 | 0,0023 |
| A\_51\_P519002 | BC028549 | Mus musculus, clone IMAGE:1380624, mRNA | 0.70 | 0,0192 |
| A\_51\_P386448 | AK040480 | serine/threonine kinase 33 | 0.70 | 0,0420 |
| A\_51\_P314941 | NM\_013862 | Mus musculus hematopoietic, heart, liver (Hhl-pending), mRNA | 0.70 | 0,0267 |
| A\_51\_P113578 | AK015033 | unknown EST | 0.70 | 0,0207 |
| A\_51\_P363914 | NM\_007636 | Mus musculus chaperonin subunit 2 (beta) (Cct2), mRNA | 0.70 | 0,0304 |
| A\_51\_P140415 | NM\_008265 | Murine mRNA for Hox-1.4 protein | 0.70 | 0,0014 |
| A\_51\_P385415 | NM\_053208 | Mus musculus EGL nine homolog 2 (C. elegans) (Egln2), mRNA | 0.70 | 0,0185 |
| A\_51\_P175146 | NM\_027769 | copine III | 0.70 | 0,0055 |
| A\_51\_P151433 | NM\_026554 | Mus musculus nuclear cap binding protein subunit 2, 20kDa (Ncbp2), mRNA | 0.70 | 0,0238 |
| A\_51\_P287952 | AK040892 | unclassifiable | 0.70 | 0,0356 |
| A\_51\_P459820 | AK006200 | hypothetical protein | 0.70 | 0,0191 |
| A\_51\_P150195 | NM\_028860 | Mus musculus, Similar to myotubularin related protein 3, clone IMAGE:4972572, mRNA, partial cds | 0.70 | 0,0435 |
| A\_51\_P498558 | NM\_146028 | Mus musculus, similar to src homology three (SH3) and cysteine rich domain, clone MGC:38869 IMAGE:5361431, mRNA, complete cds | 0.70 | 0,0359 |
| A\_51\_P439311 | AK129415 | unknown EST | 0.70 | 0,0055 |
| A\_51\_P335916 | NM\_023331 | Mus musculus RIKEN cDNA 3110052F15 gene (3110052F15Rik), mRNA | 0.70 | 0,0043 |
| A\_51\_P487964 | NM\_147033 | Mus musculus olfactory receptor MOR263-2 (MOR263-2), mRNA | 0.70 | 0,0465 |
| A\_51\_P464234 | NM\_008020 | Mus musculus FK506 binding protein 2 (13 kDa) (Fkbp2), mRNA | 0.70 | 0,0037 |
| A\_51\_P312121 | NM\_011723 | Mus musculus xanthine dehydrogenase (Xdh), mRNA | 0.70 | 0,0063 |
| A\_51\_P129299 | NM\_013635 | Mus musculus pantophysin (Pphn), mRNA | 0.70 | 0,0153 |
| A\_51\_P462774 | A\_51\_P462774 | Mus musculus cDNA, 3 end | 0.70 | 0,0162 |
| A\_51\_P125385 | NM\_031248 | Mus musculus mitogen activated protein binding protein interacting protein (Mapbpip-pending), mRNA | 0.70 | 0,0137 |
| A\_51\_P390117 | NM\_023256 | keratin 21, type I, cytoskeletal homolog [Rattus norvegicus] | 0.70 | 0,0078 |
| A\_51\_P454696 | BC028441 | hypothetical protein | 0.70 | 0,0073 |
| A\_51\_P230987 | NM\_025951 | Mus musculus phosphatidylinositol 4-kinase type 2 beta (Pi4k2b-pending), mRNA | 0.70 | 0,0184 |
| A\_51\_P426872 | NM\_024228 | Mus musculus RIKEN cDNA 1110015E22 gene (1110015E22Rik), mRNA | 0.69 | 0,0125 |
| A\_51\_P182896 | NM\_028502 | Mus musculus RIKEN cDNA 1700013N18 gene (1700013N18Rik), mRNA | 0.69 | 0,0130 |
| A\_51\_P169693 | NM\_198095 | Mus musculus, Similar to bone marrow stromal cell antigen 2, clone MGC:28276 IMAGE:4009434, mRNA, complete cds | 0.69 | 0,0199 |
| A\_51\_P366542 | NM\_011925 | Mus musculus CD97 antigen (Cd97), mRNA | 0.69 | 0,0051 |
| A\_51\_P456266 | NM\_019392 | Mus musculus TYRO3 protein tyrosine kinase 3 (Tyro3), mRNA | 0.69 | 0,0044 |
| A\_51\_P104920 | NM\_008895 | pro-opiomelanocortin-alpha | 0.69 | 0,0098 |
| A\_51\_P334785 | NM\_175026 | Similar to interferon activated gene 203 [Mus musculus] | 0.69 | 0,0468 |
| A\_51\_P375870 | NM\_198214 | Mus musculus mRNA for mKIAA0374 protein | 0.69 | 0,0164 |
| A\_51\_P234907 | XM\_488509 | centrosomal protein 1 | 0.69 | 0,0248 |
| A\_51\_P137675 | AF357361 | Mus musculus clone MBII-407 C/D box snoRNA, partial sequence | 0.69 | 0,0105 |
| A\_51\_P515908 | NM\_175451 | similar to P63 PROTEIN [Homo sapiens] | 0.69 | 0,0279 |
| A\_51\_P113413 | NAP057085-1 | Mus musculus olfactory receptor GA\_x5J8B7W6B6J-189947-189022 (GA\_x5J8B7W6B6J-189947-189022) pseudogene | 0.69 | 0,0155 |
| A\_51\_P497594 | AK037109 | hypothetical protein | 0.69 | 0,0047 |
| A\_51\_P376883 | NM\_029601 | hypothetical protein | 0.69 | 0,0074 |
| A\_51\_P364168 | NM\_008513 | Mus musculus low density lipoprotein receptor-related protein 5 (Lrp5), mRNA | 0.69 | 0,0080 |
| A\_51\_P503696 | NM\_174996 | hypothetical protein | 0.69 | 0,0058 |
| A\_51\_P334739 | NM\_021339 | Mus musculus cell adhesion molecule-related/down-regulated by oncogenes (Cdon), mRNA | 0.69 | 0,0330 |
| A\_51\_P472574 | NM\_023304 | Mus musculus fibroblast growth factor 22 (Fgf22), mRNA | 0.69 | 0,0146 |
| A\_51\_P110381 | NM\_144943 | Mus musculus mRNA for C type lectin (langerin gene) | 0.69 | 0,0068 |
| A\_51\_P161503 | XM\_622761 | similar to VACUOLAR ATP SYNTHASE SUBUNIT F (EC 3.6.3.14) (V-ATPASE F SUBUNIT) (VACUOLAR PROTON PUMP F SUBUNIT) (V-ATPASE 14 KDA SUBUNIT) [Rattus norvegicus] | 0.69 | 0,0334 |
| A\_51\_P151038 | BC087943 | MITOCHONDRIAL PROCESSING PEPTIDASE BETA SUBUNIT, MITOCHONDRIAL PRECURSOR (EC 3.4.24.64) (BETA-MPP) (P-52) | 0.69 | 0,0182 |
| A\_51\_P377596 | AK078481 | unclassifiable | 0.69 | 0,0099 |
| A\_51\_P181615 | NM\_178712 | similar to G-PROTEIN-COUPLED RECEPTOR HE6 PRECURSOR homolog [Homo sapiens] | 0.69 | 0,0387 |
| A\_51\_P467798 | NM\_022018 | Mus musculus niban protein (Niban), mRNA | 0.69 | 0,0476 |
| A\_51\_P494675 | NM\_028071 | Mus musculus coactosin-like protein (Clp-pending), mRNA | 0.69 | 0,0147 |
| A\_51\_P504162 | NM\_017390 | Mus musculus seminal vesicle protein, secretion 2 (Svs2), mRNA | 0.69 | 0,0444 |
| A\_51\_P190881 | BC049187 | Mus musculus, Similar to RIKEN cDNA 1100001D10 gene, clone IMAGE:3582399, mRNA | 0.69 | 0,0286 |
| A\_51\_P289486 | NAP108603-1 | Mus musculus olfactory receptor MOR190-4P (MOR190-4P) pseudogene | 0.69 | 0,0283 |
| A\_51\_P117903 | BE306420 | Mus musculus cDNA clone NIA:K0746F08 IMAGE:30078019 5 | 0.69 | 0,0124 |
| A\_51\_P514085 | NM\_013606 | Mus musculus myxovirus (influenza virus) resistance 2 (Mx2), mRNA | 0.69 | 0,0294 |
| A\_51\_P245989 | NM\_009915 | Mus musculus chemokine (C-C) receptor 2 (Cmkbr2), mRNA | 0.69 | 0,0120 |
| A\_51\_P221014 | NM\_010581 | Mus musculus integrin-associated protein (Itgp), mRNA | 0.69 | 0,0082 |
| A\_51\_P473409 | NM\_134117 | Mus musculus, clone IMAGE:5134400, mRNA, partial cds | 0.69 | 0,0026 |
| A\_51\_P214269 | NM\_023580 | Eph receptor A1 | 0.69 | 0,0228 |
| A\_51\_P483231 | NM\_008413 | Mus musculus Janus kinase 2 (Jak2), mRNA | 0.69 | 0,0071 |
| A\_51\_P183025 | NM\_133893 | Mus musculus 2-5 oligoadenylate synthetase 1D (Oas1d), mRNA | 0.69 | 0,0042 |
| A\_51\_P375550 | NM\_146866 | Mus musculus olfactory receptor MOR187-3 (MOR187-3), mRNA | 0.69 | 0,0203 |
| A\_51\_P318012 | M79303 | Mus musculus cDNA clone IMAGE:4016802 5 | 0.69 | 0,0182 |
| A\_51\_P355617 | AK002346 | CASPASE RECRUITMENT DOMAIN PROTEIN 11 (CARD-CONTAINING MAGUK PROTEIN 3) (CARMA 1) homolog [Homo sapiens] | 0.69 | 0,0098 |
| A\_51\_P472405 | NM\_019443 | Mus musculus NADH dehydrogenase (ubiquinone) 1 alpha subcomplex, 1 (Ndufa1), mRNA | 0.69 | 0,0203 |
| A\_51\_P388374 | NM\_026141 | DJ12G14.1 (NOVEL CYCLOPHILIN TYPE PEPTIDYL-PROLYL CIS-TRANS ISOMERASE) (FRAGMENT) homolog [Homo sapiens] | 0.69 | 0,0225 |
| A\_51\_P267971 | NM\_011259 | Mus musculus regenerating islet-derived 3 alpha (Reg3a), mRNA | 0.69 | 0,0040 |
| A\_51\_P138990 | BC019741 | Mus musculus regulator of G-protein signaling 11 mRNA, partial cds | 0.69 | 0,0071 |
| A\_51\_P401673 | NM\_053072 | Mus musculus ethanol decreased 4 (Etohd4), mRNA | 0.69 | 0,0116 |
| A\_51\_P248965 | NM\_023121 | Mus musculus G protein gamma 8 subunit, partial cds | 0.69 | 0,0428 |
| A\_51\_P330353 | NM\_026226 | POLY A BINDING PROTEIN, CYTOPLASMIC 1 homolog [Mus musculus] | 0.69 | 0,0041 |
| A\_51\_P339417 | NM\_013905 | Mus musculus hairy/enhancer-of-split related with YRPW motif-like (Heyl), mRNA | 0.69 | 0,0084 |
| A\_51\_P113322 | NM\_172896 | Similar to PAN2 protein | 0.69 | 0,0076 |
| A\_51\_P158822 | NM\_026002 | Mus musculus lyric (Lyric-pending), mRNA | 0.69 | 0,0415 |
| A\_51\_P506813 | NM\_009442 | Mus musculus transcription termination factor 1 (Ttf1), mRNA | 0.69 | 0,0023 |
| A\_51\_P110814 | NM\_028794 | nudix (nucleoside diphosphate linked moiety X)-type motif 9 | 0.69 | 0,0182 |
| A\_51\_P329251 | NM\_025366 | Mus musculus RIKEN cDNA 1110001O19 gene (1110001O19Rik), mRNA | 0.69 | 0,0263 |
| A\_51\_P146560 | NM\_018857 | Mus musculus mesothelin (Msln), mRNA | 0.69 | 0,0394 |
| A\_51\_P258443 | BE914468 | Mus musculus cDNA, 5 end | 0.69 | 0,0228 |
| A\_51\_P368394 | NM\_019964 | Mus musculus DnaJ (Hsp40) homolog, subfamily B, member 8 (Dnajb8), mRNA | 0.69 | 0,0037 |
| A\_51\_P292160 | NM\_177772 | hypothetical Lipid-binding serum glycoprotein containing protein | 0.69 | 0,0171 |
| A\_51\_P194976 | AK004984 | weakly similar to CYTOCHROME P450 MONOOXYGENASE (FRAGMENT) [Brachydanio rerio] | 0.69 | 0,0016 |
| A\_51\_P215356 | NM\_178080 | MYOMEGALIN homolog [Rattus norvegicus] | 0.69 | 0,0327 |
| A\_51\_P247121 | NM\_008199 | Mus musculus histocompatibility 2, blastocyst (H2-Bl), mRNA | 0.69 | 0,0158 |
| A\_51\_P301274 | NM\_026606 | hypothetical protein | 0.69 | 0,0188 |
| A\_51\_P342786 | AK029786 | weakly similar to DNA MISMATCH REPAIR PROTEIN MLH3 (MUTL PROTEIN HOMOLOG 3) [Homo sapiens] | 0.69 | 0,0053 |
| A\_51\_P154692 | NM\_207157 | Mus musculus olfactory receptor MOR259-3P (MOR259-3P) pseudogene | 0.69 | 0,0138 |
| A\_51\_P329198 | NM\_020521 | Mus musculus vomeronasal 1 receptor, B5 (V1rb5), mRNA | 0.69 | 0,0274 |
| A\_51\_P128229 | AK013201 | inferred: HN1 like {Homo sapiens} | 0.69 | 0,0128 |
| A\_51\_P282523 | NM\_054044 | Mus musculus RIKEN cDNA 9530074E10 gene (9530074E10Rik), mRNA | 0.69 | 0,0199 |
| A\_51\_P259694 | AK009778 | X-ray repair complementing defective repair in Chinese hamster cells 1 | 0.69 | 0,0042 |
| A\_51\_P291037 | AK129175 | PLEXIN A3 PRECURSOR PLEXIN 4 TRANSMEMBRANE PROTEIN | 0.69 | 0,0173 |
| A\_51\_P343147 | AK006280 | weakly similar to PROTEIN PHOSPHATASE INHIBITOR 2 (IPP-2) [Rattus norvegicus] | 0.69 | 0,0192 |
| A\_51\_P423732 | NM\_029035 | Mus musculus RIKEN cDNA 4930422J18 gene (4930422J18Rik), mRNA | 0.69 | 0,0058 |
| A\_51\_P495379 | NM\_177699 | Mus musculus, Similar to FH1/FH2 domain-containing protein, clone IMAGE:4503930, mRNA | 0.69 | 0,0028 |
| A\_51\_P444015 | A\_51\_P444015 | PROBABLE UBIQUITIN CARBOXYL-TERMINAL HYDROLASE FAF-X (EC 3.1.2.15) (UBIQUITIN THIOLESTERASE FAF-X) (UBIQUITIN-SPECIFIC PROCESSING PROTEASE FAF-X) (DEUBIQUITINATING ENZYME FAF-X) (FAT FACETS PROTEIN RELATED, X-LINKED) (UBIQUIT | 0.69 | 0,0017 |
| A\_51\_P216085 | NM\_027026 | Mus musculus, RIKEN cDNA 1700006D24 gene, clone MGC:31551 IMAGE:4503387, mRNA, complete cds | 0.69 | 0,0162 |
| A\_51\_P134829 | NM\_008272 | Mus musculus homeo box C9 (Hoxc9), mRNA | 0.69 | 0,0163 |
| A\_51\_P268068 | X80339 | M.musculus Six1 mRNA | 0.69 | 0,0329 |
| A\_51\_P175303 | NM\_019725 | Mus musculus transducin-like enhancer of split 2, homolog of Drosophila E(spl) (Tle2), mRNA | 0.69 | 0,0239 |
| A\_51\_P382524 | XM\_283873 | Mus musculus putative E1-E2 ATPase mRNA, partial cds | 0.69 | 0,0138 |
| A\_51\_P364609 | NM\_019699 | Mus musculus fatty acid desaturase 2 (Fads2), mRNA | 0.69 | 0,0078 |
| A\_51\_P333669 | NM\_013751 | Mus musculus Harvey rat sarcoma virus oncogene relatedsuppressor (Hrasrs-pending), mRNA | 0.69 | 0,0118 |
| A\_51\_P363194 | AK019693 | similar to EIF4GII [Homo sapiens] | 0.68 | 0,0066 |
| A\_51\_P188271 | NM\_054042 | Mus musculus tumor endothelial marker 1 precursor (Tem1-pending), mRNA | 0.68 | 0,0035 |
| A\_51\_P184314 | NM\_026390 | Mus musculus, Similar to RIKEN cDNA 1300013G12 gene, clone IMAGE:3582146, mRNA | 0.68 | 0,0279 |
| A\_51\_P138548 | NM\_008253 | Mus musculus high mobility group box 3 (Hmgb3), mRNA | 0.68 | 0,0263 |
| A\_51\_P230537 | XM\_133435 | Mus musculus, clone IMAGE:4216925, mRNA, partial cds | 0.68 | 0,0096 |
| A\_51\_P342318 | NM\_026409 | ATP DEPENDENT RNA HELICASE DEAD BOX PROTEIN | 0.68 | 0,0319 |
| A\_51\_P420100 | NM\_025736 | Mus musculus RIKEN cDNA 4921531G14 gene (4921531G14Rik), mRNA | 0.68 | 0,0141 |
| A\_51\_P208031 | NM\_178854 | hypothetical Leucine-rich repeat, typical subtype containing protein | 0.68 | 0,0272 |
| A\_51\_P194628 | NM\_173037 | hypothetical ARM repeat structure containing protein | 0.68 | 0,0371 |
| A\_51\_P408310 | XM\_138431 | hypothetical Vitamin B12 dependent methionine synthase activation domain containing protein | 0.68 | 0,0064 |
| A\_51\_P385940 | AK220117 | Mus musculus cDNA, 3 end | 0.68 | 0,0104 |
| A\_51\_P138731 | NM\_080457 | Mus musculus mucin (Muc4) mRNA, partial cds | 0.68 | 0,0139 |
| A\_51\_P355427 | NM\_080639 | Mus musculus tissue inhibitor of metalloproteinase 4 (Timp4), mRNA | 0.68 | 0,0010 |
| A\_51\_P271637 | A\_51\_P271637 | Mus musculus cDNA, 3 end | 0.68 | 0,0075 |
| A\_51\_P510782 | NM\_130859 | Mus musculus caspase recruitment domain family, member 10 (Card10), mRNA | 0.68 | 0,0268 |
| A\_51\_P296361 | NM\_001017429 | Mus musculus cDNA clone IMAGE:476100 5. | 0.68 | 0,0104 |
| A\_51\_P191199 | NM\_027863 | weakly similar to GLYCOPHORIN C (PAS-2) (GLYCOPROTEIN BETA) (GLPC) (GLYCOCONNECTIN) (SIALOGLYCOPROTEIN D) (GLYCOPHORIN D) (GPD) [Homo sapiens] | 0.68 | 0,0154 |
| A\_51\_P205833 | NM\_007531 | Mus musculus B-cell receptor-associated protein 37 (Bcap37), mRNA | 0.68 | 0,0410 |
| A\_51\_P384946 | NM\_010885 | Mus musculus NADH dehydrogenase (ubiquinone) 1 alpha subcomplex 2 (Ndufa2), mRNA | 0.68 | 0,0342 |
| A\_51\_P509264 | NM\_010455 | Mus musculus homeo box A7 (Hoxa7), mRNA | 0.68 | 0,0021 |
| A\_51\_P390857 | AK054376 | unclassifiable | 0.68 | 0,0099 |
| A\_51\_P473154 | NM\_011633 | Mus musculus Tnf receptor-associated factor 5 (Traf5), mRNA | 0.68 | 0,0105 |
| A\_51\_P201354 | AK048370 | L1 repeat, Tf subfamily, member 30 | 0.68 | 0,0095 |
| A\_51\_P213765 | AF177144 | Mus musculus inositol hexaphosphate kinase 1 (Ihpk1), mRNA | 0.68 | 0,0050 |
| A\_51\_P442890 | AF289178 | Mus musculus anti-DNA monoclonal autoantibody G4-20 heavy chain variable region mRNA, partial cds | 0.68 | 0,0028 |
| A\_51\_P313067 | AK035153 | weakly similar to oncofetal-laminin binding collagen (fragments) [Homo sapiens] | 0.68 | 0,0238 |
| A\_51\_P445924 | A\_51\_P445924 | Mus musculus cDNA, 3 end | 0.68 | 0,0388 |
| A\_51\_P175567 | AK077691 | Mus musculus thymus expressed gene 3 (Thyex3-pending), mRNA | 0.68 | 0,0491 |
| A\_51\_P412369 | NM\_028087 | similar to BETA-1,6-N-ACETYLGLUCOSAMINYLTRANSFERASE (GLUCOSAMINYL (N-ACETYL) TRANSFERASE 3, MUCIN TYPE) [Homo sapiens] | 0.68 | 0,0111 |
| A\_51\_P193935 | NM\_008650 | Mus musculus methylmalonyl-Coenzyme A mutase (Mut), mRNA | 0.68 | 0,0316 |
| A\_51\_P213087 | NM\_175101 | Mus musculus, clone MGC:7080 IMAGE:3157147, mRNA, complete cds | 0.68 | 0,0223 |
| A\_51\_P489578 | AK009384 | P11F3 homolog [Xenopus laevis] | 0.68 | 0,0455 |
| A\_51\_P395646 | NM\_144961 | Mus musculus myosin, heavy polypeptide 2, skeletal muscle, adult (Myh2), mRNA | 0.68 | 0,0232 |
| A\_51\_P497953 | NM\_026666 | UBINUCLEIN homolog [Homo sapiens] | 0.68 | 0,0411 |
| A\_51\_P115027 | BC036999 | Mus musculus, Similar to AFG3 ATPase family gene 3-like 2 (yeast), clone IMAGE:5043529, mRNA | 0.68 | 0,0097 |
| A\_51\_P328014 | NM\_011991 | Mus musculus COP9 (constitutive photomorphogenic) homolog, subunit 3 (Arabidopsis thaliana) (Cops3), mRNA | 0.68 | 0,0262 |
| A\_51\_P423762 | NM\_175211 | similar to RAL GUANINE NUCLEOTIDE EXCHANGE FACTOR RALGPS1A [Homo sapiens] | 0.68 | 0,0314 |
| A\_51\_P320249 | AK122579 | musculus adult male colon cDNA, RIKEN full-length enriched library, clone:9030612M13:unclassifiable transcript, full insert sequence | 0.68 | 0,0053 |
| A\_51\_P459741 | NM\_026081 | PER1 INTERACTING PROTEIN OF THE SUPRACHIAMATIC NUCLEUS homolog [Rattus norvegicus] | 0.68 | 0,0240 |
| A\_51\_P223776 | NM\_145434 | SIMILAR TO NUCLEAR RECEPTOR SUBFAMILY 1, GROUP D, MEMBER 1 homolog [Mus musculus] | 0.68 | 0,0143 |
| A\_51\_P153805 | BC026852 | Mus musculus similar to 60S ribosomal protein L21 (LOC278335), mRNA | 0.68 | 0,0263 |
| A\_51\_P131442 | NM\_013874 | Mus musculus neuronal d4 domain family member (Neud4), mRNA | 0.68 | 0,0223 |
| A\_51\_P499530 | NM\_133971 | Mus musculus expressed sequence AW549277 (AW549277), mRNA | 0.68 | 0,0083 |
| A\_51\_P272184 | NM\_026201 | weakly similar to CDNA FLJ10590 FIS, CLONE NT2RP2004392, WEAKLY SIMILAR TO MNN4 PROTEIN [Homo sapiens] | 0.68 | 0,0277 |
| A\_51\_P257789 | AK036636 | unknown EST | 0.68 | 0,0031 |
| A\_51\_P142196 | AK003142 | Mus musculus H19 fetal liver mRNA (H19), mRNA | 0.68 | 0,0383 |
| A\_51\_P257309 | AK122224 | unknown EST | 0.68 | 0,0028 |
| A\_51\_P365037 | AK019847 | NY-REN-18 antigen | 0.68 | 0,0134 |
| A\_51\_P335969 | NM\_010043 | Mouse desmin mRNA | 0.68 | 0,0030 |
| A\_51\_P210885 | NM\_172463 | hypothetical EGF-like domain, subtype 2/EGF-like domain/Type II EGF-like signature containing protein | 0.68 | 0,0445 |
| A\_51\_P293369 | NM\_009488 | Mus musculus putative pheromone receptor (VR1) mRNA, complete cds | 0.68 | 0,0243 |
| A\_51\_P105021 | NM\_011236 | Mus musculus RAD52 homolog, (S. cerevisiae) (Rad52), mRNA | 0.68 | 0,0489 |
| A\_51\_P205301 | NAP057258-1 | Mus musculus olfactory receptor MOR202-25P (MOR202-25P) pseudogene | 0.68 | 0,0049 |
| A\_51\_P209258 | AK080354 | unclassifiable | 0.68 | 0,0079 |
| A\_51\_P372463 | NM\_008053 | Mus musculus fragile X mental retardation gene 1, autosomal homolog (Fxr1h), mRNA | 0.68 | 0,0102 |
| A\_51\_P379373 | NM\_145381 | Similar to: Mus musculus CGI-83 protein (Cgi-83-pending), mRNA | 0.68 | 0,0022 |
| A\_51\_P156222 | NM\_175522 | hypothetical RNI-like structure containing protein | 0.68 | 0,0048 |
| A\_51\_P460302 | NM\_130863 | Mus musculus, Similar to adrenergic, beta, receptor kinase 1, clone IMAGE:3586085, mRNA, partial cds | 0.68 | 0,0072 |
| A\_51\_P138744 | NM\_019933 | Mus musculus protein tyrosine phosphatase, non-receptor type 4 (Ptpn4), mRNA | 0.68 | 0,0175 |
| A\_51\_P165960 | NM\_019766 | telomerase binding protein, p23 | 0.68 | 0,0024 |
| A\_51\_P478311 | NM\_133787 | Mus musculus expressed sequence C87860 (C87860), mRNA | 0.68 | 0,0298 |
| A\_51\_P393747 | NM\_172689 | Mus musculus, Similar to RNA helicase, clone IMAGE:4950144, mRNA | 0.68 | 0,0021 |
| A\_51\_P145433 | NM\_011699 | Mus musculus expressed sequence AU019331 (AU019331), mRNA | 0.68 | 0,0334 |
| A\_51\_P261835 | NM\_025376 | Mus musculus RIKEN cDNA 1110002H13 gene (1110002H13Rik), mRNA | 0.68 | 0,0455 |
| A\_51\_P481934 | NM\_007662 | Mus musculus cadherin 15 (Cdh15), mRNA | 0.68 | 0,0143 |
| A\_51\_P455240 | NM\_144857 | Mus musculus, clone MGC:19067 IMAGE:4192711, mRNA, complete cds | 0.68 | 0,0219 |
| A\_51\_P488411 | NM\_022028 | Mus musculus WW domain-containing protein 3 (Wwp3-pending), mRNA | 0.68 | 0,0283 |
| A\_51\_P410687 | NM\_018826 | Mus musculus iroquois related homeobox 5 (Drosophila) (Irx5), mRNA | 0.68 | 0,0100 |
| A\_51\_P319244 | D89080 | Mus musculus fibroblast growth factor 10 (Fgf10), mRNA | 0.68 | 0,0120 |
| A\_51\_P470029 | NM\_027592 | Mus musculus, Similar to adrenal gland protein AD-004, clone MGC:28404 IMAGE:4024616, mRNA, complete cds | 0.68 | 0,0341 |
| A\_51\_P316616 | NM\_174992 | Mus musculus, Similar to hypothetical protein from clone 643, clone MGC:7903 IMAGE:3582955, mRNA, complete cds | 0.68 | 0,0138 |
| A\_51\_P124477 | NM\_133231 | Mus musculus regulatory factor X-associated protein (Rfxap), mRNA | 0.68 | 0,0260 |
| A\_51\_P368705 | NM\_026574 | Mus musculus RIKEN cDNA 4632409L19 gene (4632409L19Rik), mRNA | 0.68 | 0,0027 |
| A\_51\_P152873 | NM\_207670 | M.musculus mRNA for HCMV-interacting protein, clone pJS3 | 0.68 | 0,0065 |
| A\_51\_P148374 | AK018327 | SUBSTANCE K RECEPTOR SKR NEUROKININ A RECEPTOR NK 2 RECEPTOR NK | 0.68 | 0,0064 |
| A\_51\_P322910 | NM\_026340 | similar to RIBONUCLEASES P/MRP PROTEIN SUBUNIT POP1 (EC 3.1.26.5) (HPOP1) [Homo sapiens] | 0.68 | 0,0423 |
| A\_51\_P184484 | NM\_008607 | Mus musculus matrix metalloproteinase 13 (Mmp13), mRNA | 0.68 | 0,0098 |
| A\_51\_P304643 | NM\_011718 | Mus musculus wingless related MMTV integration site 10b (Wnt10b), mRNA | 0.68 | 0,0436 |
| A\_51\_P417096 | NM\_011728 | Mus musculus xeroderma pigmentosum, complementation group A (Xpa), mRNA | 0.68 | 0,0065 |
| A\_51\_P378531 | A\_51\_P378531 | Similar to: Mus musculus intracisternal A particles (Iap), mRNA | 0.68 | 0,0405 |
| A\_51\_P104710 | NM\_173428 | Mus musculus SCO-spondin (LOC243369), mRNA | 0.68 | 0,0075 |
| A\_51\_P488888 | NM\_013632 | Mus musculus purine-nucleoside phosphorylase (Pnp), mRNA | 0.68 | 0,0046 |
| A\_51\_P408561 | NM\_207219 | Mus musculus, clone MGC:31023 IMAGE:3989373, mRNA, complete cds | 0.68 | 0,0216 |
| A\_51\_P381397 | XM\_131770 | hypothetical protein | 0.68 | 0,0007 |
| A\_51\_P260428 | NM\_008976 | Mus musculus protein tyrosine phosphatase, non-receptor type 14 (Ptpn14), mRNA | 0.68 | 0,0299 |
| A\_51\_P256759 | NM\_212470 | similar to APOPTOSIS RELATED PROTEIN APR-3 [Homo sapiens] | 0.68 | 0,0168 |
| A\_51\_P368855 | NM\_010385 | Mus musculus H2-K region expressed gene 2 (H2-Ke2), mRNA | 0.68 | 0,0101 |
| A\_51\_P352027 | TC1436661 | Mus musculus cDNA, 5 end | 0.68 | 0,0030 |
| A\_51\_P288643 | BC006028 | Mus musculus, clone MGC:7391 IMAGE:3487966, mRNA, complete cds | 0.68 | 0,0103 |
| A\_51\_P409729 | NM\_021307 | Mus musculus zinc finger protein 112 (Zfp112), mRNA | 0.68 | 0,0191 |
| A\_51\_P459368 | NM\_025825 | Mus musculus, RIKEN cDNA 1300003O07 gene, clone MGC:29424 IMAGE:5065139, mRNA, complete cds | 0.68 | 0,0318 |
| A\_51\_P476859 | NAP057262-1 | Mus musculus olfactory receptor MOR125-3P (MOR125-3P) pseudogene | 0.67 | 0,0283 |
| A\_51\_P447495 | NM\_027580 | hypothetical protein | 0.67 | 0,0224 |
| A\_51\_P374651 | AK047427 | CHROMATIN | 0.67 | 0,0384 |
| A\_51\_P377228 | NM\_175447 | hypothetical protein | 0.67 | 0,0473 |
| A\_51\_P415558 | NM\_009669 | Mus musculus amylase 2, pancreatic (Amy2), mRNA | 0.67 | 0,0293 |
| A\_51\_P255899 | NM\_011578 | Mus musculus betaglycan mRNA, complete cds | 0.67 | 0,0293 |
| A\_51\_P149209 | AK015560 | weakly similar to developmental control protein Kr2 [Mus musculus] | 0.67 | 0,0307 |
| A\_51\_P131025 | NM\_026890 | hypothetical protein | 0.67 | 0,0307 |
| A\_51\_P309328 | NM\_013752 | Mus musculus nibrin (Nbn), mRNA | 0.67 | 0,0484 |
| A\_51\_P242024 | NM\_018753 | Mus musculus tyrosine 3-monooxygenase/tryptophan 5-monooxygenase activation protein, beta polypeptide (Ywhab), mRNA | 0.67 | 0,0016 |
| A\_51\_P215599 | NM\_028104 | Mus musculus RIKEN cDNA 2010107K19 gene (2010107K19Rik), mRNA | 0.67 | 0,0045 |
| A\_51\_P234113 | NM\_172729 | similar to CASPASE RECRUITMENT DOMAIN PROTEIN 4 (NOD1 PROTEIN) [Homo sapiens] | 0.67 | 0,0258 |
| A\_51\_P398647 | NM\_175558 | weakly similar to SIMILAR TO HYPOTHETICAL PROTEIN FLJ20626 [Homo sapiens] and weakly similar to ZINC FINGER 202 M3 SPLICE VARIANT [Mus musculus] | 0.67 | 0,0075 |
| A\_51\_P438537 | NM\_011352 | Mus musculus sema domain, immunoglobulin domain (Ig), and GPI membrane anchor, (semaphorin) 7A (Sema7a), mRNA | 0.67 | 0,0031 |
| A\_51\_P140434 | NM\_027600 | hypothetical protein | 0.67 | 0,0053 |
| A\_51\_P281670 | NM\_172660 | endonuclease G | 0.67 | 0,0026 |
| A\_51\_P293901 | NM\_026819 | Mus musculus RIKEN cDNA 1110029G07 gene (1110029G07Rik), mRNA | 0.67 | 0,0475 |
| A\_51\_P170647 | S79463 | M-Sema F=a factor in neural network development [mice, neonatal brain, mRNA, 3503 nt] | 0.67 | 0,0180 |
| A\_51\_P339987 | NM\_175344 | hypothetical protein | 0.67 | 0,0056 |
| A\_51\_P218556 | AK016089 | hypothetical protein | 0.67 | 0,0053 |
| A\_51\_P166339 | NM\_009635 | Mus musculus advillin (Advil-pending), mRNA | 0.67 | 0,0196 |
| A\_51\_P131261 | A\_51\_P131261 | Mus musculus cDNA, 3 end | 0.67 | 0,0124 |
| A\_51\_P361557 | NM\_138680 | Mus musculus CGI-74-like SR-rich (LOC192196), mRNA | 0.67 | 0,0108 |
| A\_51\_P351765 | NM\_025584 | hypothetical Glycine-rich region containing protein | 0.67 | 0,0128 |
| A\_51\_P269553 | NM\_010439 | Mus musculus high mobility group box 1 (Hmgb1), mRNA | 0.67 | 0,0095 |
| A\_51\_P208922 | NM\_011491 | Mus musculus stanniocalcin 2 (Stc2), mRNA | 0.67 | 0,0010 |
| A\_51\_P418781 | NAP057153-1 | Mus musculus olfactory receptor GA\_x5J8B7W31JB-148149-147210 (GA\_x5J8B7W31JB-148149-147210) pseudogene | 0.67 | 0,0464 |
| A\_51\_P516896 | NM\_019411 | Mus musculus protein phosphatase 2a, catalytic subunit, alpha isoform (Ppp2ca), mRNA | 0.67 | 0,0185 |
| A\_51\_P120277 | NM\_017378 | Mus musculus protocadherin 12 (Pcdh12), mRNA | 0.67 | 0,0427 |
| A\_51\_P121236 | NM\_022654 | Mus musculus leucine-rich and death domain containing (Lrdd), mRNA | 0.67 | 0,0411 |
| A\_51\_P305787 | NM\_029781 | RAB36, member RAS oncogene family | 0.67 | 0,0113 |
| A\_51\_P318551 | NM\_145431 | Mus musculus, hypothetical protein similar to beta-transducin family, clone MGC:25690 IMAGE:3491925, mRNA, complete cds | 0.67 | 0,0055 |
| A\_51\_P199688 | NAP057232-1 | Mus musculus olfactory receptor MOR206-5P (MOR206-5P) pseudogene | 0.67 | 0,0359 |
| A\_51\_P436469 | NM\_144821 | similar to DJ1112D6.1 (PUTATIVE NOVEL PROTEIN SIMILAR TO BACTERIAL NARK (NITRITE EXTRUSION PROTEIN, NITRITE FACILITATOR)) (FRAGMENT) [Homo sapiens] | 0.67 | 0,0071 |
| A\_51\_P364890 | NM\_054089 | Mus musculus nuclear receptor coactivator 6 interacting protein (Ncoa6ip), mRNA | 0.67 | 0,0199 |
| A\_51\_P415647 | NM\_145556 | Mus musculus similar to TAR DNA-binding protein-43 (TDP-43) (LOC230908), mRNA | 0.67 | 0,0192 |
| A\_51\_P453145 | AK077387 | hypothetical Regulator of chromosome condensation (RCC1) profile containing protein | 0.67 | 0,0055 |
| A\_51\_P454666 | NM\_144509 | Mus musculus ARL-6 interacting protein-4 (Aip-4) mRNA, partial cds | 0.67 | 0,0302 |
| A\_51\_P107738 | NM\_028175 | hypothetical protein | 0.67 | 0,0020 |
| A\_51\_P328963 | NM\_011672 | Mus musculus ubiquitin fusion degradation 1 like (Ufd1l), mRNA | 0.67 | 0,0264 |
| A\_51\_P517982 | NM\_026693 | similar to GANGLIOSIDE EXPRESSION FACTOR 2 (GEF-2) (GENERAL PROTEIN TRANSPORT FACTOR P16) (GATE-16) [Homo sapiens] | 0.67 | 0,0199 |
| A\_51\_P353606 | NM\_172126 | Mus musculus fertilin alpha precursor (ADAM 1) mRNA, partial cds | 0.67 | 0,0187 |
| A\_51\_P439426 | NM\_133360 | Mus musculus clone 677 acetyl-CoA carboxylase 265 mRNA, partial cds | 0.67 | 0,0346 |
| A\_51\_P473734 | NM\_010386 | Mus musculus histocompatibility 2, class II, locus DMa (H2-DMa), mRNA | 0.67 | 0,0020 |
| A\_51\_P330428 | NM\_007918 | Mus musculus eukaryotic translation initiation factor 4E binding protein 1 (Eif4ebp1), mRNA | 0.67 | 0,0263 |
| A\_51\_P326709 | NM\_001025613 | ZINC FINGER PROTEIN CEZANNE homolog [Homo sapiens] | 0.67 | 0,0096 |
| A\_51\_P322138 | NM\_024441 | Mus musculus heat shock 27kD protein 2 (Hspb2), mRNA | 0.67 | 0,0322 |
| A\_51\_P230600 | NM\_201376 | Mus musculus squamous cell carcinoma antigen 2 (Scca2), mRNA | 0.67 | 0,0056 |
| A\_51\_P345896 | NM\_133880 | Mus musculus expressed sequence AI507170 (AI507170), mRNA | 0.67 | 0,0248 |
| A\_51\_P284703 | NM\_018814 | Mus musculus pecanex homolog (Drosophila) (Pcnx), mRNA | 0.67 | 0,0359 |
| A\_51\_P191700 | NM\_009929 | Mus musculus procollagen, type XVIII, alpha 1 (Col18a1), mRNA | 0.67 | 0,0148 |
| A\_51\_P308726 | NM\_025516 | Mus musculus serologically defined breast cancer antigen 84 (Sdbcag84), mRNA | 0.67 | 0,0082 |
| A\_51\_P182774 | NM\_018863 | Mus musculus prodynorphin (Pdyn), mRNA | 0.67 | 0,0081 |
| A\_51\_P348022 | NM\_026623 | Mus musculus cleavage and polyadenylation specific factor 5, 25 kD subunit (Cpsf5), mRNA | 0.67 | 0,0008 |
| A\_51\_P294550 | NM\_019829 | Mus musculus syntaxin 5A (Stx5a), mRNA | 0.67 | 0,0230 |
| A\_51\_P304437 | NM\_173019 | "6-PHOSPHOFRUCTO-2-KINASE/FRUCTOSE-2,6-BIPHOSPHATASE 4 (6PF-2-K/FRU- 2,6-P2ASE TESTIS-TYPE ISOZYME) [INCLUDES: 6-PHOSPHOFRUCTO-2-KINASE (EC 2.7.1.105)� FRUCTOSE-2,6-BISPHOSPHATASE (EC 3.1.3.46)] homolog [Rattus norvegicus]" | 0.67 | 0,0026 |
| A\_51\_P223656 | NM\_008484 | Mus musculus laminin, beta 3 (Lamb3), mRNA | 0.67 | 0,0356 |
| A\_51\_P275983 | BC027414 | Mus musculus RNA component of mitochondrial RNAase P, 1 (Rmrp1), misc RNA | 0.67 | 0,0427 |
| A\_51\_P334072 | NM\_009989 | Mus musculus cytochrome c, testis (Cyct), mRNA | 0.67 | 0,0128 |
| A\_51\_P467573 | NM\_011669 | ubiquitin hydrolyzing enzyme 1 | 0.67 | 0,0374 |
| A\_51\_P465148 | NM\_007798 | cathepsin B | 0.67 | 0,0374 |
| A\_51\_P441455 | AK032831 | hypothetical PPR repeats containing protein | 0.67 | 0,0125 |
| A\_51\_P219453 | AK005740 | hypothetical Serine proteases, trypsin family containing protein | 0.67 | 0,0079 |
| A\_51\_P161612 | NM\_009831 | Mus musculus cyclin G (Ccng), mRNA | 0.67 | 0,0117 |
| A\_51\_P368696 | NM\_009684 | Mus musculus apoptotic protease activating factor 1 (Apaf1), mRNA | 0.67 | 0,0056 |
| A\_51\_P172872 | NM\_026456 | Mus musculus RIKEN cDNA 2610301I15 gene (2610301I15Rik), mRNA | 0.67 | 0,0061 |
| A\_51\_P354062 | NM\_011197 | Mus musculus prostaglandin F2 receptor negative regulator (Ptgfrn), mRNA | 0.67 | 0,0042 |
| A\_51\_P422629 | NM\_011603 | Mus musculus TATA box binding protein-like protein (Tlp), mRNA | 0.67 | 0,0408 |
| A\_51\_P386526 | NM\_033572 | Mus musculus RIKEN cDNA 5730496C04 gene (5730496C04Rik), mRNA | 0.67 | 0,0143 |
| A\_51\_P334404 | A\_51\_P334404 | Mus musculus pyridoxal (pyridoxine, vitamin B6) kinase, pseudogene (Pdxk-ps) on chromosome 17 | 0.67 | 0,0008 |
| A\_51\_P178911 | NM\_001011769 | Mus musculus olfactory receptor MOR256-47 (MOR256-47) pseudogene | 0.67 | 0,0018 |
| A\_51\_P507344 | NM\_001003915 | SODIUM/IODIDE COTRANSPORTER NA + /I COTRANSPORTER SODIUM IODIDE SYMPORTER NA+/I | 0.66 | 0,0312 |
| A\_51\_P224054 | NM\_009459 | Mus musculus ubiquitin-conjugating enzyme E2H (Ube2h), mRNA | 0.66 | 0,0264 |
| A\_51\_P215780 | NM\_029572 | Mus musculus endoplasmic reticulum resident protein 44kDa (Erp44-pending), mRNA | 0.66 | 0,0037 |
| A\_51\_P280971 | NM\_008948 | Mus musculus proteasome (prosome, macropain) 26S subunit, ATPase 3 (Psmc3), mRNA | 0.66 | 0,0243 |
| A\_51\_P515585 | NM\_026017 | Mus musculus, clone MGC:25664 IMAGE:4486464, mRNA, complete cds | 0.66 | 0,0087 |
| A\_51\_P215237 | NM\_207105 | Mus musculus histocompatibility 2, class II antigen A, beta 1 (H2-Ab1), mRNA | 0.66 | 0,0044 |
| A\_51\_P305770 | NM\_145158 | Mus musculus elastin microfibril interface located protein 2 (Emilin2-pending), mRNA | 0.66 | 0,0195 |
| A\_51\_P239924 | NM\_009090 | Mus musculus RNA polymerase II 3 (Rpo2-3), mRNA | 0.66 | 0,0220 |
| A\_51\_P133097 | AA867022 | hypothetical Cysteine-rich region containing protein | 0.66 | 0,0145 |
| A\_51\_P439612 | NM\_020266 | Mus musculus DnaJ (Hsp40) homolog, subfamily B, member 10 (Dnajb10), mRNA | 0.66 | 0,0096 |
| A\_51\_P370445 | NM\_138748 | Mus musculus protein phosphatase 2A, regulatory subunit B (PR 53) (Ppp2r4), mRNA | 0.66 | 0,0401 |
| A\_51\_P485203 | NM\_147201 | Mus musculus HLS7-interacting protein kinase mRNA, complete cds | 0.66 | 0,0065 |
| A\_51\_P200291 | NM\_008146 | Mus musculus golgi autoantigen, golgin subfamily a, 3 (Golga3), mRNA | 0.66 | 0,0103 |
| A\_51\_P103779 | NM\_145512 | hypothetical protein | 0.66 | 0,0102 |
| A\_51\_P324037 | BG294955 | Mus musculus cDNA, 5 end | 0.66 | 0,0248 |
| A\_51\_P210634 | NM\_023547 | Mus musculus RIKEN cDNA 2510009I23 gene (2510009I23Rik), mRNA | 0.66 | 0,0309 |
| A\_51\_P389358 | NM\_021356 | Mus musculus growth factor receptor bound protein 2-associated protein 1 (Gab1), mRNA | 0.66 | 0,0391 |
| A\_51\_P332303 | NM\_007901 | Mus musculus endothelial differentiation sphingolipid G-protein-coupled receptor 1 (Edg1), mRNA | 0.66 | 0,0430 |
| A\_51\_P477641 | NM\_008883 | Mus musculus plexin 3 (Plxn3), mRNA | 0.66 | 0,0101 |
| A\_51\_P180952 | NM\_011612 | Mus musculus tumor necrosis factor receptor superfamily, member 9 (Tnfrsf9), mRNA | 0.66 | 0,0383 |
| A\_51\_P472937 | TC1536721 | Mus musculus cDNA clone IMAGE:5681032 3 | 0.66 | 0,0149 |
| A\_51\_P448757 | NM\_013698 | Mus musculus TXK tyrosine kinase (Txk), mRNA | 0.66 | 0,0096 |
| A\_51\_P403443 | NM\_053008 | Mus musculus oligodendrocyte transcription factor 3 (Olig3), mRNA | 0.66 | 0,0161 |
| A\_51\_P321136 | NM\_146222 | Mus musculus, Similar to hypothetical protein FLJ23342, clone MGC:37388 IMAGE:4977181, mRNA, complete cds | 0.66 | 0,0010 |
| A\_51\_P250465 | BC027547 | Mus musculus, Similar to HSPC133 protein, clone MGC:41295 IMAGE:1531973, mRNA, complete cds | 0.66 | 0,0170 |
| A\_51\_P453635 | NM\_010784 | Mus musculus midkine pseudogene 1 (Mdk-ps1) on chromosome 11 | 0.66 | 0,0177 |
| A\_51\_P338594 | NM\_025387 | Mus musculus RIKEN cDNA 1110021D01 gene (1110021D01Rik), mRNA | 0.66 | 0,0009 |
| A\_51\_P268529 | AF045741 | Mus musculus deoxyribonuclease II alpha (Dnase2a), mRNA | 0.66 | 0,0055 |
| A\_51\_P269002 | NM\_080793 | Mus musculus SET domain-containing protein 7 (Set7), mRNA | 0.66 | 0,0058 |
| A\_51\_P425511 | NM\_013903 | Mus musculus matrix metalloproteinase 20 (enamelysin) (Mmp20), mRNA | 0.66 | 0,0480 |
| A\_51\_P140607 | NM\_138757 | Mus musculus hypothetical protein MGC28965 (MGC28965), mRNA | 0.66 | 0,0043 |
| A\_51\_P460890 | NM\_009059 | Mus musculus ral guanine nucleotide dissociation stimulator,-like 2 (Rgl2), mRNA | 0.66 | 0,0192 |
| A\_51\_P323583 | NM\_134111 | Mus musculus expressed sequence AW048865 (AW048865), mRNA | 0.66 | 0,0008 |
| A\_51\_P285462 | NM\_146276 | Mus musculus olfactory receptor MOR280-1 (MOR280-1), mRNA | 0.66 | 0,0018 |
| A\_51\_P372762 | NM\_010127 | Mus musculus POU domain, class 6, transcription factor 1 (Pou6f1), mRNA | 0.66 | 0,0149 |
| A\_51\_P116838 | NM\_010024 | Mus musculus dopachrome tautomerase (Dct), mRNA | 0.66 | 0,0352 |
| A\_51\_P511985 | NM\_010092 | Mus musculus dual-specificity tyrosine-(Y)-phosphorylation regulated kinase 1b (Dyrk1b), mRNA | 0.66 | 0,0172 |
| A\_51\_P481742 | XM\_139909 | SOMATOSTATIN RECEPTOR TYPE 5 (SS5R) | 0.66 | 0,0080 |
| A\_51\_P227332 | NM\_023372 | Mus musculus RIKEN cDNA 0610025G13 gene (0610025G13Rik), mRNA | 0.66 | 0,0348 |
| A\_51\_P211732 | NM\_008977 | Mus musculus protein tyrosine phosphatase, non-receptor type 2 (Ptpn2), mRNA | 0.66 | 0,0214 |
| A\_51\_P369443 | NM\_148939 | Mus musculus lymphocyte antigen 6 complex, locus G5B (Ly6g5b), mRNA | 0.66 | 0,0348 |
| A\_51\_P282663 | NM\_010421 | Mus musculus hexosaminidase A (Hexa), mRNA | 0.66 | 0,0008 |
| A\_51\_P384248 | XM\_620559 | PI-3-KINASE-RELATED KINASE SMG-1 homolog [Homo sapiens] | 0.66 | 0,0303 |
| A\_51\_P396225 | NM\_146811 | Mus musculus olfactory receptor MOR165-3 (MOR165-3), mRNA | 0.66 | 0,0225 |
| A\_51\_P207324 | NM\_011881 | Mus musculus rhodopsin kinase (Rhok), mRNA | 0.66 | 0,0081 |
| A\_51\_P170350 | XM\_619920 | Mus musculus, Similar to RIKEN cDNA 1500041I23 gene, clone IMAGE:5012813, mRNA | 0.66 | 0,0224 |
| A\_51\_P178561 | NM\_033042 | Mus musculus tumor necrosis factor receptor superfamily, member 12 (Tnfrsf12), mRNA | 0.66 | 0,0283 |
| A\_51\_P421294 | NM\_008132 | Mus musculus glutamine repeat protein 1 (Glrp1), mRNA | 0.66 | 0,0114 |
| A\_51\_P159132 | XM\_128552 | similar to PROTEIN DISULFIDE ISOMERASE PDIP [Homo sapiens] | 0.66 | 0,0258 |
| A\_51\_P193424 | NM\_025614 | SMALL ANDROGEN RECEPTOR-INTERACTING PROTEIN homolog [Rattus norvegicus] | 0.66 | 0,0196 |
| A\_51\_P248122 | NM\_133234 | Mus musculus PUMA/JFY1 protein (PUMA), mRNA | 0.66 | 0,0468 |
| A\_51\_P297579 | NM\_008102 | Mus musculus GTP cyclohydrolase 1 (Gch), mRNA | 0.66 | 0,0025 |
| A\_51\_P118742 | NM\_021354 | Mus musculus developmentally regulated GTP binding protein 2 (Drg2), mRNA | 0.66 | 0,0171 |
| A\_51\_P460004 | NM\_009404 | Mus musculus tumor necrosis factor (ligand) superfamily, member 9 (Tnfsf9), mRNA | 0.66 | 0,0301 |
| A\_51\_P387845 | AK172909 | Mus musculus, clone IMAGE:3597827, mRNA, partial cds | 0.66 | 0,0046 |
| A\_51\_P245103 | NM\_008021 | Mus musculus forkhead box M1 (Foxm1), mRNA | 0.66 | 0,0019 |
| A\_51\_P390310 | NM\_026248 | Mus musculus RIKEN cDNA 4930430A15 gene (4930430A15Rik), mRNA | 0.66 | 0,0082 |
| A\_51\_P118300 | NM\_011430 | Mus musculus synuclein, gamma (Sncg), mRNA | 0.66 | 0,0458 |
| A\_51\_P411079 | AK045374 | enhancer of zeste homolog 1 (Drosophila) | 0.66 | 0,0047 |
| A\_51\_P280055 | NAP057112-1 | Mus musculus olfactory receptor GA\_x5J8B7W5M25-134931-135139 (GA\_x5J8B7W5M25-134931-135139) pseudogene | 0.66 | 0,0395 |
| A\_51\_P273433 | NM\_008926 | Mus musculus protein kinase, cGMP-dependent, type II (Prkg2), mRNA | 0.66 | 0,0371 |
| A\_51\_P295846 | AK046831 | BRAIN-SPECIFIC ANGIOGENESIS INHIBITOR 1 PRECURSOR homolog [Homo sapiens] | 0.66 | 0,0248 |
| A\_51\_P436689 | XM\_137955 | Similar to: Mus musculus, Similar to apolipoprotein B (including Ag(x) antigen), clone IMAGE:5052989, mRNA, partial cds | 0.66 | 0,0083 |
| A\_51\_P195825 | NM\_024445 | TRAX-INTERACTING PROTEIN 1 (FRAGMENT) homolog [Mus musculus] | 0.66 | 0,0140 |
| A\_51\_P231226 | NM\_027238 | hypothetical Tetratricopeptide repeat (TPR) structure containing protein | 0.66 | 0,0185 |
| A\_51\_P441745 | AK021396 | hypothetical protein | 0.66 | 0,0070 |
| A\_51\_P317512 | AK017758 | hypothetical protein | 0.66 | 0,0045 |
| A\_51\_P277075 | NM\_027650 | Mus musculus RIKEN cDNA 4933405P08 gene (4933405P08Rik), mRNA | 0.66 | 0,0071 |
| A\_51\_P502906 | NM\_177544 | Mus musculus angiogenin pseudogene 1 (Ang-ps1) | 0.66 | 0,0127 |
| A\_51\_P152765 | NM\_011488 | Mus musculus signal transducer and activator of transcription 5A (Stat5a), mRNA | 0.66 | 0,0112 |
| A\_51\_P135296 | XM\_127913 | similar to HYPOTHETICAL 66.1 KDA PROTEIN [Macaca fascicularis] | 0.66 | 0,0009 |
| A\_51\_P131119 | NM\_178697 | similar to CALCIUM-ACTIVATED CHLORIDE CHANNEL-2 [Homo sapiens] | 0.66 | 0,0081 |
| A\_51\_P341949 | NM\_007749 | Mus musculus, Similar to cytochrome c oxidase, subunit VIIc, clone MGC:18637 IMAGE:4216583, mRNA, complete cds | 0.66 | 0,0116 |
| A\_51\_P478419 | NM\_178680 | similar to UNC45-RELATED PROTEIN [Brachydanio rerio] | 0.66 | 0,0178 |
| A\_51\_P252127 | NAP057276-1 | Mus musculus olfactory receptor MOR111-8P (MOR111-8P) pseudogene | 0.66 | 0,0306 |
| A\_51\_P211798 | NM\_020599 | Mus musculus retinaldehyde binding protein 1 (Rlbp1), mRNA | 0.66 | 0,0049 |
| A\_51\_P137560 | NM\_007959 | Mus musculus ets related protein 71 (Etsrp71), mRNA | 0.66 | 0,0150 |
| A\_51\_P249104 | NM\_177844 | hypothetical protein | 0.66 | 0,0117 |
| A\_51\_P293729 | NM\_054102 | Mus musculus Nd1 (Nd1-pending), mRNA | 0.66 | 0,0284 |
| A\_51\_P224617 | NM\_001001932 | ENDOSOMAL PROTEIN homolog [Homo sapiens] | 0.66 | 0,0185 |
| A\_51\_P342276 | NM\_172868 | Mus musculus clone pEN70 unknown mRNA, partial cds | 0.66 | 0,0033 |
| A\_51\_P140541 | BC090963 | Mus musculus mRNA for protein phosphatase 1B2 (ppm1b2 gene) | 0.66 | 0,0252 |
| A\_51\_P444565 | AK083376 | weakly similar to H-2 CLASS I HISTOCOMPATIBILITY ANTIGEN, D-37 ALPHA CHAIN PRECURSOR [Mus musculus], full insert sequence. | 0.66 | 0,0248 |
| A\_51\_P317031 | NM\_025779 | Mus musculus RIKEN cDNA 9030408N13 gene (9030408N13Rik), mRNA | 0.66 | 0,0150 |
| A\_51\_P231979 | NM\_013472 | Mus musculus annexin A6 (Anxa6), mRNA | 0.66 | 0,0402 |
| A\_51\_P366704 | NM\_015729 | Mus musculus acyl-Coenzyme A oxidase 1, palmitoyl (Acox1), mRNA | 0.66 | 0,0037 |
| A\_51\_P508510 | Z11886 | Mus musculus Notch gene homolog 1, (Drosophila) (Notch1), mRNA | 0.66 | 0,0316 |
| A\_51\_P257686 | NM\_011490 | Mus musculus staufen (RNA binding protein) homolog 1 (Drosophila) (Stau1), mRNA | 0.66 | 0,0031 |
| A\_51\_P476757 | NM\_198640 | Mouse mRNA sequence, partial cds | 0.66 | 0,0295 |
| A\_51\_P177819 | NM\_030743 | Mus musculus zinc finger protein 313 (Zfp313), mRNA | 0.66 | 0,0053 |
| A\_51\_P391996 | BC011329 | Mus musculus, RIKEN cDNA 0610042A05 gene, clone MGC:18955 IMAGE:3984654, mRNA, complete cds | 0.66 | 0,0073 |
| A\_51\_P414752 | NM\_139222 | Mus musculus mRNA for putative beta defensin (defb11 gene) | 0.66 | 0,0104 |
| A\_51\_P183252 | NM\_080442 | Mus musculus testis-specific serine/threonine kinase 3b (Tssk3b) mRNA, complete cds | 0.66 | 0,0047 |
| A\_51\_P321331 | NM\_011479 | Mus musculus serine palmitoyltransferase, long chain base subunit 2 (Sptlc2), mRNA | 0.66 | 0,0045 |
| A\_51\_P379171 | AK007819 | hypothetical protein | 0.66 | 0,0239 |
| A\_51\_P307463 | NM\_207210 | similar to DUAL-SPECIFICITY TYROSINE-PHOSPHORYLATION REGULATED KINASE 4 (EC 2.7.1.-) (FRAGMENT) [Homo sapiens] | 0.66 | 0,0050 |
| A\_51\_P441086 | A\_51\_P441086 | "similar to TR:G1109823 G1109823 COSMID C16A3. " | 0.66 | 0,0204 |
[truncated: 204,885 more chars]
