# Supplementary material for: The functional genome of CA1 and CA3 neurons under native conditions and in response to ischemia
Source: BMC Genomics. 2007 Oct 15;8:370. doi: 10.1186/1471-2164-8-370 (PMC2194787; doi:10.1186/1471-2164-8-370)
Supplement: Additional file 6 — List of all significantly regulated genes in CA3 vs CA1 (ischemic state). HTML file containing all significantly regulated genes in CA3 vs CA1 in the ischemic state. Given are Agilent probe numbers, accession numbers, gene names, enrichment factors ("M (CA3/CA1")), and false-discovery-rate corrected p-values. [file 1471-2164-8-370-S6.htm]

Additional file 6


| Agilent probe# | Accession# | gene name | M (CA3/CA1) | P.fdr |
| A\_51\_P142972 | NM\_029947 | Mus musculus PR domain containing 8 (Prdm8), mRNA | 9.65 | 0.01685 |
| A\_51\_P239750 | NM\_008380 | Mus musculus inhibin beta-A (Inhba), mRNA | 9.10 | 0.02056 |
| A\_51\_P373082 | NM\_016778 | Mus musculus Bcl-2-related ovarian killer protein (Bok), mRNA | 8.29 | 0.00726 |
| A\_51\_P417998 | NM\_013757 | Mus musculus synaptotagmin-like 4 (Sytl4), mRNA | 7.12 | 0.00726 |
| A\_51\_P260288 | AK016705 | Mus musculus adult male testis cDNA, RIKEN full-length enriched library, clone:4933406O10 product:RIKEN cDNA 4933406O10 gene, full insert sequence. | 6.33 | 0.01262 |
| A\_51\_P254855 | NM\_011198 | Mus musculus prostaglandin-endoperoxide synthase 2 (Ptgs2), mRNA | 6.30 | 0.00996 |
| A\_51\_P191669 | NM\_007694 | Mus musculus chromogranin B (Chgb), mRNA | 6.26 | 0.00726 |
| A\_51\_P496569 | NM\_178804 | Mus musculus slit homolog 2 (Drosophila) (Slit2), mRNA | 5.40 | 0.02047 |
| A\_51\_P274488 | NM\_021543 | Mus musculus protocadherin 8 (Pcdh8), mRNA | 5.14 | 0.02414 |
| A\_51\_P147766 | NM\_175079 | Mus musculus RIKEN cDNA A730016F12 gene (A730016F12Rik), transcript variant 2, mRNA | 4.93 | 0.04086 |
| A\_51\_P305508 | NM\_181988 | Mus musculus RAS-like, estrogen-regulated, growth-inhibitor (Rerg), mRNA | 4.14 | 0.04396 |
| A\_51\_P125009 | NM\_021495 | Mus musculus poliovirus receptor-related 3 (Pvrl3), transcript variant alpha, mRNA | 4.07 | 0.01475 |
| A\_51\_P311919 | NM\_008731 | Mus musculus neuropeptide Y receptor Y2 (Npy2r), mRNA | 4.03 | 0.02441 |
| A\_51\_P384993 | NM\_175481 | Mus musculus glutamate receptor, ionotropic, kainate 4 (Grik4), mRNA | 4.00 | 0.04050 |
| A\_51\_P206144 | NM\_133733 | Mus musculus RIKEN cDNA 9030425E11 gene (9030425E11Rik), mRNA | 3.76 | 0.00614 |
| A\_51\_P276943 | NM\_016696 | Mus musculus glypican 1 (Gpc1), mRNA | 3.75 | 0.01043 |
| A\_51\_P298837 | NM\_009262 | Mus musculus sparc/osteonectin, cwcv and kazal-like domains proteoglycan 1 (Spock1), mRNA | 3.52 | 0.03357 |
| A\_51\_P370423 | NM\_022319 | Mus musculus calsyntenin 2 (Clstn2), mRNA | 3.24 | 0.00546 |
| A\_51\_P189962 | NM\_026380 | Mus musculus regulator of G-protein signaling 8 (Rgs8), mRNA | 3.14 | 0.00546 |
| A\_51\_P118237 | NM\_011361 | Mus musculus serum/glucocorticoid regulated kinase (Sgk), mRNA | 3.04 | 0.00726 |
| A\_51\_P222882 | NM\_010595 | Mus musculus potassium voltage-gated channel, shaker-related subfamily, member 1 (Kcna1), mRNA | 2.99 | 0.04676 |
| A\_51\_P199580 | NM\_028185 | Mus musculus U7 snRNP-specific Sm-like protein LSM11 (Lsm11), mRNA | 2.96 | 0.04439 |
| A\_51\_P224164 | NM\_011867 | Mus musculus solute carrier family 26, member 4 (Slc26a4), mRNA | 2.85 | 0.01475 |
| A\_51\_P125629 | NM\_173402 | Mus musculus regulator of G-protein signaling 12 (Rgs12), mRNA | 2.83 | 0.04163 |
| A\_51\_P481494 | NM\_011248 | Mus musculus roundabout homolog 3 (Drosophila) (Robo3), mRNA | 2.75 | 0.01030 |
| A\_51\_P396331 | NM\_013681 | Mus musculus synapsin II (Syn2), mRNA | 2.74 | 0.02209 |
| A\_51\_P267634 | NM\_178751 | Mus musculus RIKEN cDNA A730041O15 gene (A730041O15Rik), mRNA | 2.72 | 0.03882 |
| A\_51\_P337230 | NM\_027864 | Mus musculus UDP-N-acetyl-alpha-D-galactosamine:polypeptide N-acetylgalactosaminyltransferase 14 (Galnt14), mRNA | 2.71 | 0.04086 |
| A\_51\_P347312 | BC019747 | Mus musculus opioid growth factor receptor-like 1, mRNA (cDNA clone MGC:30347 IMAGE:4485439), complete cds. | 2.70 | 0.02441 |
| A\_51\_P267986 | NM\_145951 | Mus musculus cytosolic ovarian carcinoma antigen 1 (Cova1), mRNA | 2.62 | 0.04676 |
| A\_51\_P308844 | NM\_153529 | Mus musculus neuritin 1 (Nrn1), mRNA | 2.60 | 0.03365 |
| A\_51\_P171200 | NM\_027307 | Mus musculus golgi phosphoprotein 2 (Golph2), mRNA | 2.50 | 0.00726 |
| A\_51\_P167313 | NM\_171824 | Mus musculus piggyBac transposable element derived 5 (Pgbd5), mRNA | 2.39 | 0.01933 |
| A\_51\_P408071 | XM\_132322 | PREDICTED: similar to mKIAA0166 protein | 2.34 | 0.00614 |
| A\_51\_P360615 | NM\_008730 | Mus musculus neuronal pentraxin 1 (Nptx1), mRNA | 2.29 | 0.03709 |
| A\_51\_P483168 | AW536275 | G0102E08-3 NIA Mouse E7.5 Embryonic Portion cDNA Library Mus musculus cDNA clone G0102E08 3'. | 2.25 | 0.04439 |
| A\_51\_P346488 | BC085129 | Mus musculus synapsin II, mRNA (cDNA clone MGC:113746 IMAGE:5701861), complete cds. | 2.04 | 0.03330 |
| A\_51\_P280125 | NM\_176860 | Mus musculus RIKEN cDNA 2810457I06 gene (2810457I06Rik), mRNA | 1.90 | 0.03020 |
| A\_51\_P303620 | NM\_001008791 | Mus musculus whirlin (Whrn), transcript variant 2, mRNA | 1.90 | 0.04439 |
| A\_51\_P229875 | AK122457 | Mus musculus mRNA for mKIAA1147 protein | 1.85 | 0.02364 |
| A\_51\_P262773 | NM\_007664 | Mus musculus cadherin 2 (Cdh2), mRNA | 1.85 | 0.03580 |
| A\_51\_P517198 | NM\_009959 | Mus musculus protocadherin alpha 5 (Pcdha5), mRNA | 1.80 | 0.03882 |
| A\_51\_P110978 | AK079102 | Mus musculus adult male diencephalon cDNA, RIKEN full-length enriched library, clone:9330189H06 product:RIBOSOMAL PROTEIN S6 KINASE ALPHA 3 (EC 2.7.1.-) (S6K-ALPHA 3) (90 KDA RIBOSOMAL PROTEIN S6 KINASE 3) (P90-RSK 3) (RIBOSOMAL S6 KINASE 2) (RSK-2) ... | 1.77 | 0.03813 |
| A\_51\_P392943 | NM\_178728 | Mus musculus cDNA sequence AB112350 (AB112350), mRNA | 1.76 | 0.00726 |
| A\_51\_P303868 | XM\_126847 | PREDICTED: similar to mKIAA1921 protein | 1.66 | 0.02283 |
| A\_51\_P332286 | NM\_153525 | Mus musculus transmembrane protein 41B (Tmem41b), mRNA | 1.55 | 0.04676 |
| A\_51\_P438841 | NM\_009819 | Mus musculus catenin (cadherin associated protein), alpha 2 (Ctnna2), transcript variant 2, mRNA | 1.52 | 0.02770 |
| A\_51\_P477419 | NM\_026756 | Mus musculus RIKEN cDNA 1110019L22 gene (1110019L22Rik), mRNA | 1.43 | 0.04086 |
| A\_51\_P300456 | AK122223 | Mus musculus mRNA for mKIAA0230 protein | 1.34 | 0.02770 |
| A\_51\_P488422 | BC050879 | Mus musculus RIKEN cDNA 4930578F03 gene, mRNA (cDNA clone MGC:63264 IMAGE:5687835), complete cds. | 0.72 | 0.04676 |
| A\_51\_P238094 | NM\_024444 | Mus musculus cytochrome P450, family 4, subfamily f, polypeptide 18 (Cyp4f18), mRNA | 0.68 | 0.02439 |
| A\_51\_P225263 | NM\_029792 | Mus musculus beta-1,3-glucuronyltransferase 1 (glucuronosyltransferase P) (B3gat1), mRNA | 0.68 | 0.03357 |
| A\_51\_P235835 | AK075797 | Mus musculus 10 day old male pancreas cDNA, RIKEN full-length enriched library, clone:1810049E24 product:DNA segment, Chr 12, ERATO Doi 647, expressed, full insert sequence. | 0.67 | 0.03896 |
| A\_51\_P327778 | NM\_011070 | Mus musculus prefoldin 2 (Pfdn2), mRNA | 0.66 | 0.04086 |
| A\_51\_P375036 | NM\_010888 | Mus musculus NADH dehydrogenase (ubiquinone) Fe-S protein 6 (Ndufs6), mRNA | 0.65 | 0.03896 |
| A\_51\_P335077 | NM\_010888 | Mus musculus NADH dehydrogenase (ubiquinone) Fe-S protein 6 (Ndufs6), mRNA | 0.65 | 0.03709 |
| A\_51\_P502872 | AK033738 | Mus musculus adult male cecum cDNA, RIKEN full-length enriched library, clone:9130604L22 product:weakly similar to HAI-2 RELATED SMALL PROTEIN (IMMORTALIZATION-UPREGULATED PROTEIN 1) (HEPATOCYTE GROWTH FACTOR ACTIVATOR INHIBITOR TYPE 2-RELATED SMALL... | 0.64 | 0.02056 |
| A\_51\_P103209 | NM\_028639 | Mus musculus tetratricopeptide repeat domain 7 (Ttc7), mRNA | 0.64 | 0.02348 |
| A\_51\_P467431 | Z83816 | M.musculus mRNA for axonemal dynein heavy chain (partial, ID mdhc8). | 0.63 | 0.04439 |
| A\_51\_P499673 | NM\_025401 | Mus musculus ubiquitin-like 5 (Ubl5), mRNA | 0.62 | 0.01030 |
| A\_51\_P133811 | XM\_618738 | PREDICTED: Mus musculus large tumor supressor (Lats1), mRNA | 0.62 | 0.04118 |
| A\_51\_P465042 | NM\_025882 | Mus musculus polymerase (DNA-directed), epsilon 4 (p12 subunit) (Pole4), mRNA | 0.62 | 0.02408 |
| A\_51\_P157554 | NM\_012047 | Mus musculus bromodomain containing 7 (Brd7), mRNA | 0.61 | 0.04439 |
| A\_51\_P494675 | NM\_028071 | Mus musculus coactosin-like 1 (Dictyostelium) (Cotl1), mRNA | 0.61 | 0.04118 |
| A\_51\_P180108 | NM\_028809 | Mus musculus actin related protein 2/3 complex, subunit 5-like (Arpc5l), mRNA | 0.60 | 0.04620 |
| A\_51\_P368885 | NM\_001001804 | Mus musculus abhydrolase domain containing 7 (Abhd7), mRNA | 0.60 | 0.04676 |
| A\_51\_P489192 | NM\_015784 | Mus musculus periostin, osteoblast specific factor (Postn), mRNA | 0.59 | 0.03730 |
| A\_51\_P296878 | NM\_173746 | Mus musculus hypothetical protein C130086A10 (C130086A10), mRNA | 0.57 | 0.02056 |
| A\_51\_P343598 | AK012407 | Mus musculus 11 days embryo whole body cDNA, RIKEN full-length enriched library, clone:2700050C19 product:unknown EST, full insert sequence | 0.56 | 0.04439 |
| A\_51\_P115471 | AK046043 | Mus musculus adult male corpora quadrigemina cDNA, RIKEN full-length enriched library, clone:B230337H21 product:inferred: Similar to apolipoprotein L {Homo sapiens}, full insert sequence | 0.55 | 0.00726 |
| A\_51\_P403704 | AK011787 | Mus musculus 10 days embryo whole body cDNA, RIKEN full-length enriched library, clone:2610100L16 product:unknown EST, full insert sequence. | 0.55 | 0.01816 |
| A\_51\_P104077 | NM\_001013024 | Mus musculus ubiquitin specific protease 13 (isopeptidase T-3) (Usp13), mRNA | 0.54 | 0.01043 |
| A\_51\_P196774 | AK045646 | Mus musculus adult male corpora quadrigemina cDNA, RIKEN full-length enriched library, clone:B230218B10 product:MESENCHYMAL STEM CELL PROTEIN DSC54 homolog | 0.54 | 0.02056 |
| A\_51\_P290059 | NM\_008083 | Mus musculus growth associated protein 43 (Gap43), mRNA | 0.54 | 0.02936 |
| A\_51\_P187067 | AK018920 | Mus musculus adult male testis cDNA, RIKEN full-length enriched library, clone:1700091G21 product:hypothetical protein, full insert sequence. | 0.53 | 0.04118 |
| A\_51\_P484200 | NM\_153777 | Mus musculus RIKEN cDNA 5730427C23 gene (5730427C23Rik), mRNA | 0.53 | 0.03905 |
| A\_51\_P309328 | NM\_013752 | Mus musculus nibrin (Nbn), mRNA | 0.51 | 0.04211 |
| A\_51\_P338886 | NM\_026950 | Mus musculus OCIA domain containing 2 (Ociad2), mRNA | 0.48 | 0.00614 |
| A\_51\_P215077 | NM\_025569 | Mus musculus microsomal glutathione S-transferase 3 (Mgst3), mRNA | 0.46 | 0.00546 |
| A\_51\_P475785 | NM\_207264 | Mus musculus cDNA sequence BC052040 (BC052040), mRNA | 0.43 | 0.02106 |
| A\_51\_P383032 | NM\_010819 | Mus musculus C-type lectin domain family 4, member d (Clec4d), mRNA | 0.43 | 0.02524 |
| A\_51\_P423484 | NM\_011254 | Mus musculus retinol binding protein 1, cellular (Rbp1), mRNA | 0.42 | 0.01315 |
| A\_51\_P272993 | NM\_172290 | Mus musculus neurotrimin (Hnt), mRNA | 0.42 | 0.00996 |
| A\_51\_P383524 | NM\_181728 | Mus musculus ADP-ribosyltransferase 3 (Art3), mRNA | 0.38 | 0.00726 |
| A\_51\_P274852 | NM\_028133 | Mus musculus EGL nine homolog 3 (C. elegans) (Egln3), mRNA | 0.37 | 0.00546 |
| A\_51\_P104985 | AI391295 | mb65f07.y1 Soares mouse p3NMF19.5 Mus musculus cDNA clone IMAGE:334309 5'. | 0.36 | 0.03645 |
| A\_51\_P421908 | NM\_145711 | Mus musculus thymocyte selection-associated HMG box gene (Tox), mRNA | 0.35 | 0.03580 |
| A\_51\_P307979 | NM\_007960 | Mus musculus ets variant gene 1 (Etv1), mRNA | 0.30 | 0.00996 |
| A\_51\_P388517 | NM\_010698 | Mus musculus LIM domain binding 2 (Ldb2), mRNA | 0.30 | 0.03730 |
| A\_51\_P497395 | NM\_145100 | Mus musculus Ly6/Plaur domain containing 1 (Lypdc1), mRNA | 0.23 | 0.03379 |
| A\_51\_P151909 | NM\_145584 | Mus musculus spondin 1, (f-spondin) extracellular matrix protein (Spon1), mRNA | 0.22 | 0.04010 |
| A\_51\_P273609 | NM\_146125 | Mus musculus inositol 1,4,5-trisphosphate 3-kinase A (Itpka), mRNA | 0.21 | 0.02387 |
| A\_51\_P356052 | NM\_175012 | Mus musculus gastrin releasing peptide (Grp), mRNA | 0.19 | 0.03379 |
| A\_51\_P284849 | XM\_131258 | PREDICTED: calcium-independent alpha-latrotoxin receptor homolog 2 | 0.18 | 0.04676 |
| A\_51\_P374752 | NM\_011255 | Mus musculus retinol binding protein 4, plasma (Rbp4), mRNA | 0.18 | 0.03580 |
| A\_51\_P296846 | NM\_172610 | Mus musculus DNA segment, Chr 15, Brigham & Women's Genetics 0669 expressed (D15Bwg0669e), mRNA | 0.14 | 0.03091 |
| A\_51\_P422429 | NM\_010825 | Mus musculus myeloid ecotropic viral integration site-related gene 1 (Mrg1), mRNA | 0.14 | 0.02936 |
| A\_51\_P458638 | NM\_183136 | Mus musculus RIKEN cDNA C630041L24 gene (C630041L24Rik), mRNA | 0.08 | 0.00546 |
|  |  |  |  |  |
