# Supplementary material for: The functional genome of CA1 and CA3 neurons under native conditions and in response to ischemia
Source: BMC Genomics. 2007 Oct 15;8:370. doi: 10.1186/1471-2164-8-370 (PMC2194787; doi:10.1186/1471-2164-8-370)
Supplement: Additional file 7 — Comparison of regulation factors derived from array analysis and quantitative PCR. Shown are 8 genes that show differential expression between CA3 and CA1 in the native or ischemic state with the regulation factor derived from array analysis, and quantitative PCR: There is a good correlation between both assessments. The Pearson correlation coefficient from these 8 comparisons is 0.75. [file 1471-2164-8-370-S7.pdf]

	Accession#	gene name	Array	qPCR
**CA3 vs CA1 sham**	NM_009788	Calbindin 28K (Calb1)	0.07	0.08 ± 0.01
	NM_181988	RAS-like, estrogen-regulated, growth-inhibitor (Rerg)	21.49	4.94 ± 0.35
	NM_008731	Neuropeptide Y receptor Y2 (Npy2r)	5.78	1.76 ± 0.32
**CA3 vs CA1 isch**	NM_011255	Retinol binding protein 4 (Rbp4)	0.18	0.29 ± 0.01
	NM_013757	Synaptotagmin-like 4 (Sytl4)	7.12	3.53 ± 0.81
	NM_021543	Protocadherin 8 (Pcdh8)	5.14	5.21± 1.25
	NM_153529	Neuritin 1 (Nrn1)	2.60	2.03 ± 0.87
	NM_145584	Spondin 1 (Spon1)	0.22	0.32 ± 0.17
