# Supplementary material for: The functional genome of CA1 and CA3 neurons under native conditions and in response to ischemia
Source: BMC Genomics. 2007 Oct 15;8:370. doi: 10.1186/1471-2164-8-370 (PMC2194787; doi:10.1186/1471-2164-8-370)
Supplement: Additional file 10 — Genes that show a significantly different CA3/CA1 distribution under normoxic and ischemic conditions. Shown are 15 genes which had significantly differing distribution to CA3 and CA1 in the normoxic versus the ischemic state. Given are the accession number, gene name, the relative CA3/CA1 ratio shift from normoxic to ischemic conditions (negative values indicate the reverse CA1/CA3 ratio), the raw P values and the FDR-corrected P-values for the siginificance of different behaviour under the two conditions), and the CA3/CA1 ratios under normoxic and ischemic conditions with the respective FDR-corrected P-values. Changes in most genes are caused by an attenuation of differential expression by ischemia. The only gene which shows a true reversal of distribution preference is Inhibin beta A (from CA1 in sham to CA3 in ischemia). [file 1471-2164-8-370-S10.htm]

| acc. no. | Agilent ID | gene name | fold CA3/CA1 ratio shift | P | P.fdr | CA3/CA1 in sham | P.fdr | CA3/CA1 in ischemia | P.fdr |
| NM\_008380 | A\_51\_P239750 | Inhibin beta-A (Inhba) | 25.93 | 0.000025 | 0.042 | -2.85 | 0.169629 | 9.10 | 0.020556 |
| NM\_001002927 | A\_51\_P102987 | Preproenkephalin 1 (Penk1) | 12.90 | 0.000010 | 0.033 | -44.05 | 0.000004 | -3.41 | 0.070075 |
| NM\_010483 | A\_51\_P322115 | 5-hydroxytryptamine (serotonin) receptor 5B (Htr5b) | 5.92 | 0.000019 | 0.042 | -12.79 | 0.000008 | -2.16 | 0.129711 |
| NM\_172632 | A\_51\_P245895 | Mitogen-activated protein kinase 4 (MAPK4) | 4.21 | 0.000009 | 0.033 | -5.88 | 0.000012 | -1.40 | 0.467282 |
| NM\_015777 | A\_51\_P168905 | Immunoglobulin (CD79A) binding protein 1b (Igbp1b) | 3.44 | 0.000022 | 0.042 | -3.56 | 0.000089 | -1.04 | 0.968626 |
| NM\_139228 | A\_51\_P267877 | Rhomboid, veinlet-like 3 (Drosophila) (Rhbdl3) | 3.32 | 0.000022 | 0.042 | -5.52 | 0.000009 | -1.66 | 0.148843 |
| NM\_173405 | A\_51\_P114634 | Archaemetzincin-1 | 2.78 | 0.000030 | 0.042 | -3.29 | 0.000043 | -1.18 | 0.700678 |
| NM\_178888 | A\_51\_P340987 | GTPase activating RANGAP domain-like 3 (Garnl3) | 2.64 | 0.000008 | 0.033 | -3.97 | 0.000004 | -1.50 | 0.107084 |
| NM\_011799 | A\_51\_P520019 | Cell division cycle 6 homolog (S. cerevisiae) (Cdc6) | 2.49 | 0.000027 | 0.042 | -3.02 | 0.000030 | -1.21 | 0.571274 |
| NM\_022565 | A\_51\_P435990 | N-deacetylase/N-sulfotransferase (heparin glucosaminyl) 4 (Ndst4) | 1.79 | 0.000028 | 0.042 | -1.95 | 0.000046 | -1.09 | 0.745666 |
| NM\_011324 | A\_51\_P213691 | Sodium channel, nonvoltage-gated, type I, alpha (Scnn1a) | -2.24 | 0.000016 | 0.042 | 2.97 | 0.000009 | 1.33 | 0.234626 |
| NM\_013820 | A\_51\_P204080 | Hexokinase 2 (Hk2) | -2.42 | 0.000010 | 0.033 | 2.80 | 0.000019 | 1.15 | 0.663895 |
| NM\_016677 | A\_51\_P487360 | Hippocalcin-like 1 (Hpcal1) | -3.07 | 0.000009 | 0.033 | 3.71 | 0.000016 | 1.21 | 0.630261 |
| NM\_027391 | A\_51\_P411200 | Iodotyrosine deiodinase (Iyd) | -8.74 | 0.000003 | 0.033 | 21.28 | 0.000004 | 2.44 | 0.091459 |
| XM\_887785 | A\_51\_P132530 | Claudin 22 | -26.46 | 0.000000 | 0.007 | 69.40 | 0.000002 | 2.62 | 0.153699 |
|  |  |  |  |  |  |  |  |  |  |
